# Asymmetric Azidation under Hydrogen Bonding Phase-Transfer Catalysis: A Combined Experimental and Computational Study

**DOI:** 10.1021/jacs.1c13434

**Published:** 2022-03-01

**Authors:** Jimmy Wang, Matthew A. Horwitz, Alexander B. Dürr, Francesco Ibba, Gabriele Pupo, Yuan Gao, Paolo Ricci, Kirsten E. Christensen, Tejas P. Pathak, Timothy D. W. Claridge, Guy C. Lloyd-Jones, Robert S. Paton, Véronique Gouverneur

**Affiliations:** † Chemistry Research Laboratory, University of Oxford, 12 Mansfield Road, Oxford OX1 3TA, U.K.; ‡ School of Chemistry, University of Edinburgh, Edinburgh EH9 3FJ, U.K.; § Novartis Institutes for Biomedical Research, 22 Windsor Street, Cambridge, Massachusetts 02139, United States; ∥ Department of Chemistry, Colorado State University, Fort Collins, Colorado 80528, United States

## Abstract

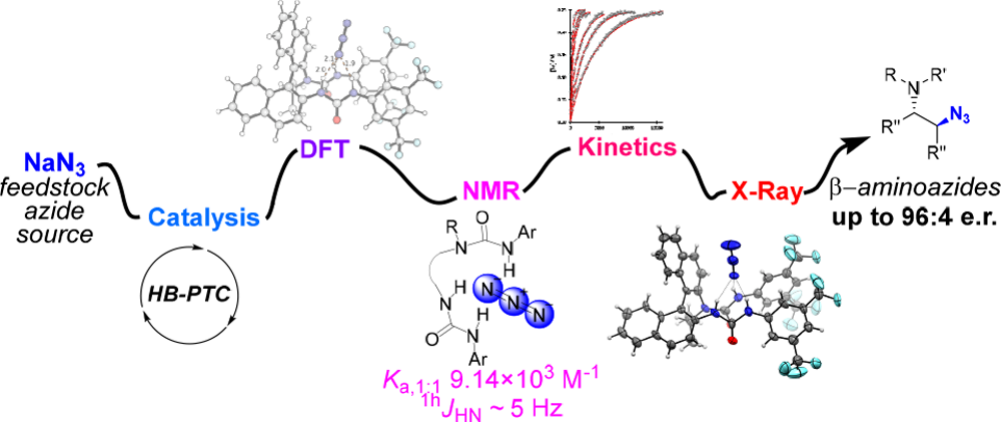

Asymmetric catalytic azidation has increased in importance to access enantioenriched nitrogen containing molecules, but methods that employ inexpensive sodium azide remain scarce. This encouraged us to undertake a detailed study on the application of hydrogen bonding phase-transfer catalysis (HB-PTC) to enantioselective azidation with sodium azide. So far, this phase-transfer manifold has been applied exclusively to insoluble metal alkali fluorides for carbon–fluorine bond formation. Herein, we disclose the asymmetric ring opening of *meso* aziridinium electrophiles derived from β-chloroamines with sodium azide in the presence of a chiral bisurea catalyst. The structure of novel hydrogen bonded azide complexes was analyzed computationally, in the solid state by X-ray diffraction, and in solution phase by ^1^H and ^14^N/^15^N NMR spectroscopy. With *N*-isopropylated BINAM-derived bisurea, end-on binding of azide in a tripodal fashion to all three NH bonds is energetically favorable, an arrangement reminiscent of the corresponding dynamically more rigid trifurcated hydrogen-bonded fluoride complex. Computational analysis informs that the most stable transition state leading to the major enantiomer displays attack from the hydrogen-bonded end of the azide anion. All three H-bonds are retained in the transition state; however, as seen in asymmetric HB-PTC fluorination, the H-bond between the nucleophile and the monodentate urea lengthens most noticeably along the reaction coordinate. Kinetic studies corroborate with the turnover rate limiting event resulting in a chiral ion pair containing an aziridinium cation and a catalyst-bound azide anion, along with catalyst inhibition incurred by accumulation of NaCl. This study demonstrates that HB-PTC can serve as an activation mode for inorganic salts other than metal alkali fluorides for applications in asymmetric synthesis.

## Introduction

Griess, Curtius, and Tiemann were the first to investigate the chemistry of metal and organic azides at the end of the 19th century,^1^ with greater interest in azidation chemistry emerging in the 1960s.^2^ Today, organic azides have established themselves as highly versatile intermediates for synthetic, material, and biological applications because they participate in diverse transformations including 1,3-dipolar cycloadditions, aza-Wittig reactions, Staudinger reductions and ligations, as well as C–H bond aminations.^3^ Various protocols for asymmetric azidation have been developed, often requiring toxic and volatile reagents such as hydrazoic acid or azidotrimethylsilane.^4^ Crystalline 1-azido-1,2-benziodoxol-3(1*H*)-one has also been used, but this azide source is of poor atom economy, and is prepared from azidotrimethylsilane.^5^ In contrast, only a few enantioenriched organic azides are obtainable directly from sodium azide, an inexpensive reagent compared to all aforementioned azide sources. Phase-transfer catalysis with chiral ammonium salts is the most successful approach for azidation with sodium azide, although details on how the azide ion interacts with the catalyst-substrate complex are scarce.^6^

**Figure 1:**
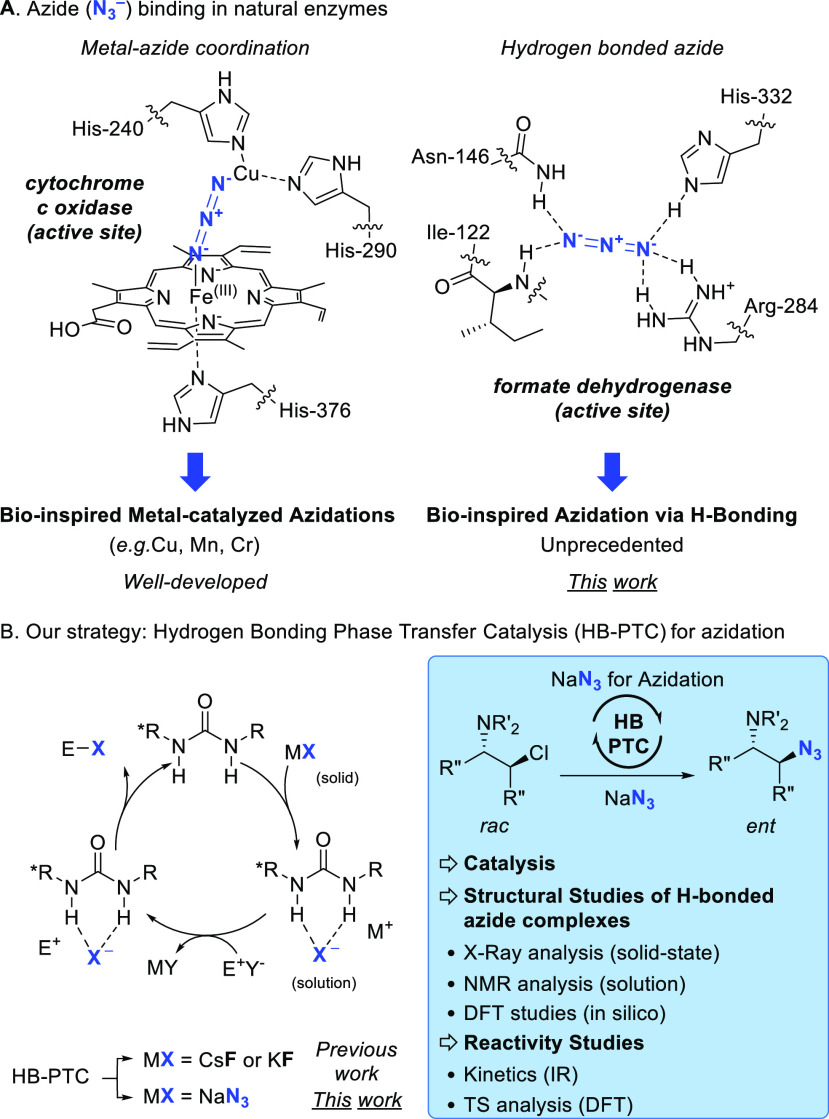
Molecular principles of catalysis and inhibition featuring hydrogen bonding interactions in natural enzymes; translational design strategies for synthetic organocatalysts. (A) Azide binding in natural enzymes. (B) Azidation with sodium azide under hydrogen bonding phase-transfer catalysis (this work).

In biology, the azide ion serves as an inhibitor of many enzymes, including cytochrome oxidases involved in the electron transport chain, and formate dehydrogenase for CO_2_ fixation or nicotinamide recycling (Figure 1A).^7^ Enzyme inhibition results from coordination of the azide ion to the metal or through H-bonding interactions as observed in the azide-bound NAD-dependent dehydrogenase complex (PDB ID 2NAD). The terminal nitrogen atoms of the azide anion can engage in H-bond contacts with several residues of the enzyme, a binding profile that enables azide to serve as a bridging ion between molecular fragments. As well as acting as an inhibitor, the azide ion has been used as a nucleophile in biocatalytic azidations. Janssen and co-workers reported the kinetic resolution of racemic epoxides by azidolysis with NaN_3_ in the presence of the halohydrin dehalogenase from *Agrobacterium radiobacter* AD1, an enzyme class that typically promotes (de)halogenation, with the exception of fluoride, in epoxide chemistry.^8^ This enzyme displays nucleophile promiscuity for a range of monovalent, linear anions other than N_3_^-^ including cyanide, cyanate, and isocyanate ions. More recently, C–H azidation at aliphatic carbons enabled by an iron-dependent halogenase was disclosed, a process highlighting coordination of the azide anion to the enzyme’s Fe(II) cofactor.^9^

The metal-coordinating ability of the azide anion has been amply exploited for catalytic azidation.^10^ For example, Groves and co-workers have reported a manganese-catalyzed aliphatic C–H azidation reaction featuring a Mn-bound intermediate in the azido transfer step.^11^ In contrast, the ability of the azide anion to engage in H-bonding interactions has not been harnessed for catalytic azidation with NaN_3_. We however note that theoretical studies have suggested that H-bonds in methylpentynol-azide clusters may influence the regiochemical outcome of 1,3-dipolar cycloaddition reactions.^12^

This state of play encouraged an in-depth investigation into the coordination chemistry of the azide ion with hydrogen bond donors (HBD) for applications in asymmetric catalysis (Figure 1B). The study detailed herein focuses on the H-bonding of azide with ureas, a class of HBD widely used in catalysis for a wide range of (asymmetric) transformations other than azidations. Mechanistically, we envisaged a scenario based on anion binding catalysis whereby the urea-bound azide ion would intercept a cationic electrophile (E^+^) in the enantiodetermining step. In this approach, H-bonding interactions to the azide anion would enable the HBD urea to function as a phase-transfer catalyst and bring NaN_3_ into solution. These interactions would attenuate the nucleophilicity of the azide. In our previous work applying this mechanistic scenario for enantioselective fluorination, background reactivity was suppressed by using an insoluble metal alkali fluoride with the urea HBD serving as phase-transfer catalyst (hydrogen bonding phase-transfer catalysis, HB-PTC).^13^ This manuscript addresses whether HB-PTC is viable for enantioselective azidation with NaN_3_. Specifically, we demonstrate C(sp^3^)–N_3_ bond formation under catalytic conditions and report the successful application of a chiral BINAM-derived bisurea catalyst to promote asymmetric azidation with sodium azide for the synthesis of enantioenriched β-amino azides. Detailed information is provided on the structure and characterization of a diverse range of (a)chiral urea-azide complexes in the solid state and in solution. Moreover, X-ray diffraction analysis, quantum chemical calculations, and NMR spectroscopy provide insight on the coordination chemistry of the azide ion to a chiral BINAM-derived bisurea catalyst. The catalytic cycle has been interrogated using a combination of kinetics and computational studies.

## Results and Discussion

### 1 Catalytic Azidation under HB-PTC

**Scheme 1:**
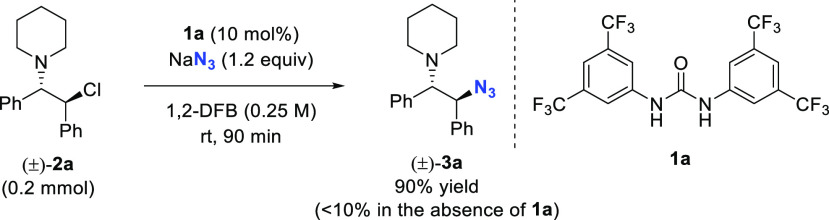
Azidation of β-Chloroamine (±)-**2a** Using NaN_3_ in 1,2-Difluorobenzene with(out) Schreiner’s Urea **1a**

We started our investigation by employing β-chloroamines as substrates as these were previously found to be reactive under HB-PTC conditions with alkali metal fluorides.^13^ When treated with NaN_3_ in 1,2-difluorobenzene in the absence of a hydrogen bond donor, model substrate (±)-**2a** afforded (±)-**3a** in less than 10% yield after 1 h (Scheme 1). When 10 mol % of Schreiner’s urea **1a**(14) was added to the reaction mixture and under otherwise identical conditions, the yield of this reaction increased to 90%.

The demonstration that the hydrogen bond donor urea **1a** catalyzes azidation of (±)-**2a** prompted the development of an enantioselective variant of this reaction. We selected the chiral BINAM-derived bisurea catalyst (*S*)**-1k** that was highly successful for fluorination, well aware that the strength and binding mode of azide anion with (*S*)**-1k** may differ significantly from fluoride (Table 1). The reaction of (±)-**2a** with NaN_3_ in 1,2-difluorobenzene in the presence of (*S*)-**1k** (5 mol %) at room temperature provided **3a** in 80% yield and 85:15 e.r., thereby demonstrating the feasibility of enantioselective azidation under HB-PTC (Table 1, entry 1). The nonalkylated (*S*)-BINAM catalyst (*S*)-**1l** with four instead of three sites for hydrogen bonding with azide afforded β-aminoazide **3a** in 74% yield, but with significantly decreased e.r., a result highlighting the crucial effect of *N*-alkylation on enantiocontrol (Table 1, entry 2).^13^*N*-Methylated catalyst (*S*)-**1m** (Table 1, entry 3) or a switch to other solvents (Table 1, entries 4 and 5) led to decreased enantiocontrol. Enantioselectivity was improved by reducing the temperature, although this required increasing both the azide and catalyst loading to achieve full conversion. Use of catalyst (*S*)-**1k** (10 mol %) with 2.4 equiv NaN_3_ at −20 °C for 72 h yielded β-amino azide (*S*,*S*)-**3a** in 74% yield and 93.5:6.5 e.r. (Table 4, entry 6).^15^ The reaction of soluble tetrabutylammonium azide in the presence of (*S*)-**1k** (5 mol %) resulted in racemic product, suggesting that phase-transfer is essential for enantioinduction (Table 1, entry 7).

**Table 1:**
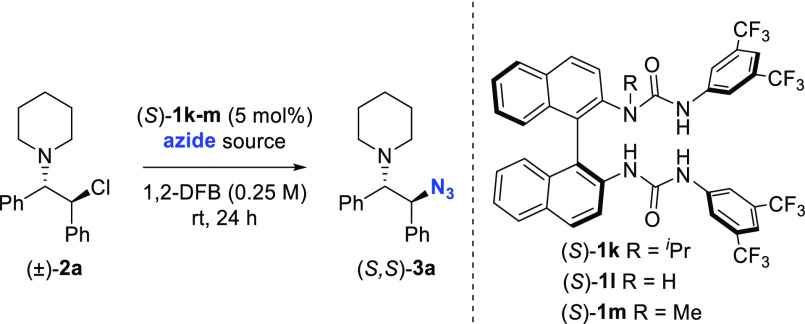
Optimization of the Reaction Conditions

entry	cat.	azide source	solvent	yield^a^ (%)	e.r.^b^
1	(*S*)-**1k**	NaN_3_ (1.2 equiv)	1,2-DFB	80	85:15
2	(*S*)-**1l**	NaN_3_ (1.2 equiv)	1,2-DFB	74	58:42
3	(*S*)-**1m**	NaN_3_ (1.2 equiv)	1,2-DFB	80	83:17
4	(*S*)-**1k**	NaN_3_ (1.2 equiv)	CHCl_3_	86	77:23
5	(*S*)-**1k**	NaN_3_ (1.2 equiv)	CH_2_Cl_2_	91	81:19
6^c^	(*S*)-**1k**	NaN_3_ (2.4 equiv)	1,2-DFB	74	93.5:6.5
7	(*S*)-**1k**	Bu_4_N·N_3_ (1.2 equiv)	1,2-DFB	90	50:50

a Yield determined by ^1^H NMR with Ph_3_CH (0.5 equiv) internal standard.

b e.r. determined after prep TLC.

c (*S*)-**1k** (10 mol %), 72 h, −20 °C. 1,2-DFB = 1,2-difluorobenzene.

**Scheme 2:**
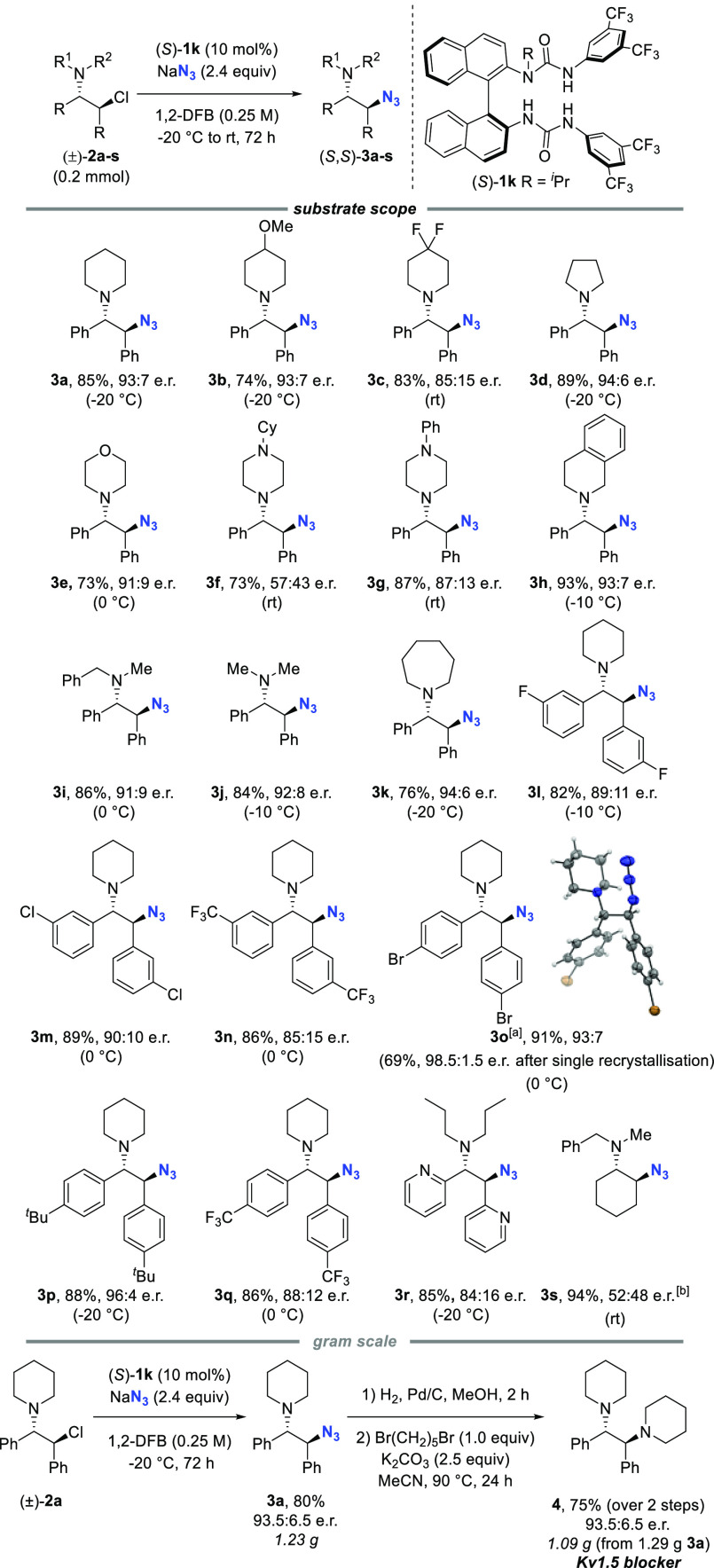
Substrate Scope, Scale-up, and Derivatization 1.09 mmol scale. Absolute configuration of major enantiomer not determined.

The optimized conditions were successfully applied to a range of *meso*-aziridinium precursors (Scheme 2). Products containing pharmaceutically relevant saturated heterocycles ,^16^ including substituted piperidines **3a**–**c**, pyrrolidine **3d**, morpholine **3e**, piperazine **3f**–**g**, and tetrahydroisoquinoline **3h** motifs were all formed with high enantioselectivity. Unsymmetrically substituted amine derivatives also performed well in this reaction to give **3h**–**i**, despite the possibility for formation of two diastereomeric *meso*-aziridinium intermediates. The reaction was found to be tolerant of *meta*- and *para*-halogen substituents (**3l**, **3m**, **3o**), trifluoromethyl groups (**3n**, **3q**), and larger alkyl groups (**3p**). The absolute configuration of **3o** was determined by single-crystal X-ray diffraction and was used to assign the absolute configuration of **3a**–**n** and **3p**–**r** by analogy. The bis-pyridyl azide **3r** was obtained in good yield and enantioselectivity. A cycloalkyl amino chloride successfully furnished product **3s** in high yield but with no enantiocontrol. The model reaction was carried out on gram scale, yielding 1.23 g of (*S*,*S*)-**3a** in 80% yield and 93.5:6.5 e.r. No measures were taken to avoid moisture or oxygen, emphasizing the operational simplicity of reactions performed under HB-PTC. Reduction of azide (*S*,*S*)-**3a** by hydrogenation and subsequent *bis*-alkylation with 1,5-dibromopentane afforded 1.1 g of enantioenriched Kv1.5 blocker **4** from (±)-**2a**.^17^

The successful application of HB-PTC to NaN_3_ encouraged a detailed analysis of how the azide ion interacts with hydrogen bond donors such as ureas in the solid state and in solution, and further mechanistic investigation based on kinetics combined with computational studies.

### 2 Insight on the Structure of Urea-Azide Complexes from Experimental and Computational Studies

**Figure 2:**
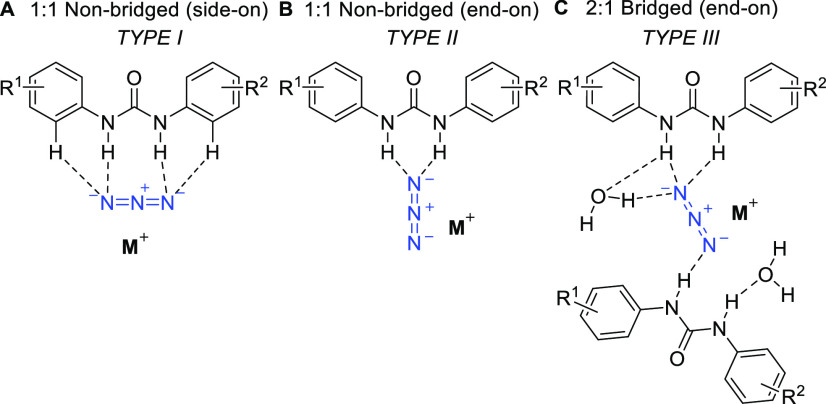
Coordination diversity of achiral urea-azide complexes. M^+^ = tetrabutylammonium or Na[15-crown-5].

**Figure 3:**
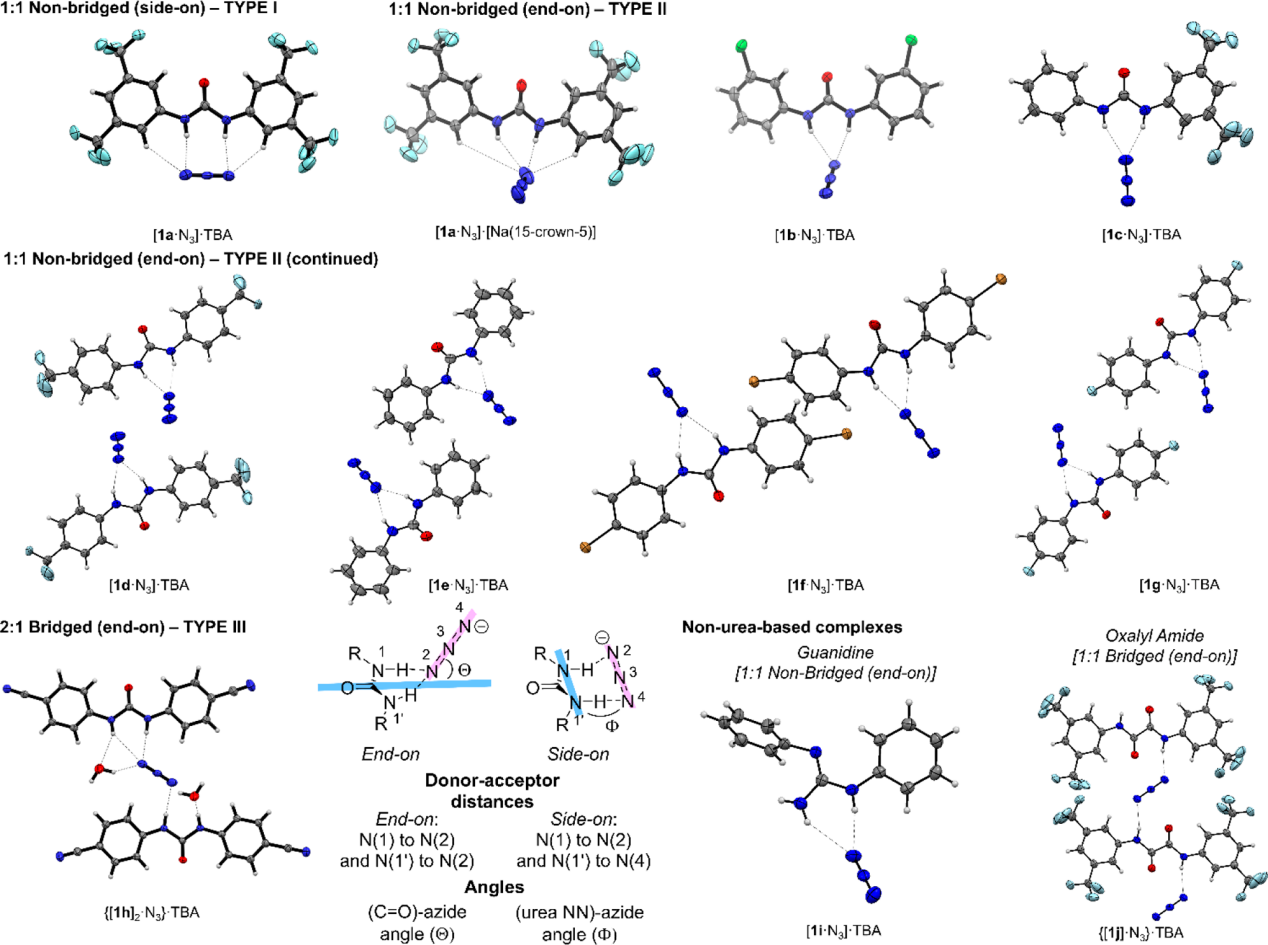
Achiral hydrogen bonded donor-azide complexes. Counter cations and crown ethers are omitted for clarity.

Studies on H-bonded azide complexes have been reported,^18^ with a single example of azide anion encapsulated in a urea receptor.^18l^ This limited knowledge on the binding modes of azide with urea motifs prompted us to prepare and characterize H-bonded azide complexes derived, at first instance, from a range of achiral hydrogen bond donors.^19^ A set of variously substituted 1,3-diarylureas was selected to examine how steric and electronic effects may influence the structures of azide complexes in the solid-state, well aware that hydrogen bond directionality and/or packing effects may be at play. All complexes were synthesized from either tetrabutylammonium (TBA) azide or a combination of sodium azide and 15-crown-5 (>95% yield). The azide salt was stirred overnight with an equimolar amount of hydrogen bond donor in acetonitrile (0.1 M), followed by evaporation of solvent to dryness. The resulting complexes were characterized by ^1^H NMR, ^13^C NMR, and IR spectroscopy and subsequently recrystallized to obtain samples suitable for X-ray analysis.^15^ A diverse set of structures was obtained, revealing distinct binding modes which were categorized depending on (i) the type of donor–acceptor interaction (side-on or end-on),^20^ (ii) whether the complex exists as a bridged or nonbridged structure—in the end-on binding mode, the azide could act as a bridging ion between two hydrogen bond donors, and (iii) the stoichiometry of the complex with a HBD:azide ratio of either 1:1 or 2:1. Figure 2 illustrates the coordination diversity of the urea-azide complexes that were successfully characterized by single-crystal X-ray diffraction analysis and distinguishes between 1:1 nonbridged, side-on (type I, Figure 2A), 1:1 nonbridged, end-on (type II, Figure 2B), and 2:1 bridged, end-on (type III, Figure 2C) complexes. Figure 3 and Table 2 highlight some key parameters for these complexes, such as donor–acceptor (D–A) distances and the angles θ and Φ, which indicate the extent to which the azide lies outside of the urea NC(=O)N plane in end-on and side-on complexes.^19^

**Table 2:** Main Structural Features of Achiral Hydrogen-Bonded Urea Complexes

entry	HBD	complex type	complex	D–A distance^22^	θ or Φ^22^
1^a^^,^^b^	**1a**, R^1^ = R^2^ = 3,5-CF_3_	1:1 Non-bridged (side-on)	[**1a**·N_3_]·TBA	2.966(4), 2.974(4), 2.962(4), 3.069(4)	1.15(14), 15.79(7)
2^c^	**1a**, R^1^ = R^2^ = 3,5-CF_3_	1:1 Non-bridged (end-on)	[**1a**·N_3_]·[Na(15-crown-5)]	2.780(9), 3.332(9)	62.40(30)
3	**1b**, R^1^ = R^2^ = 3-Cl	1:1 Non-bridged (end-on)	[**1b**·N_3_]·TBA	2.876(4), 3.000(4)	51.90(14)
4	**1c**, R^1^ = 3,5-CF_3_; R^2^ = H	1:1 Non-bridged (end-on)	[**1c**·N_3_]·TBA	2.852(9), 2.946(9), 2.834(9), 2.940(9)^c^	27.60(19), 34.21(12)^c^
5	**1d**, R^1^ = R^2^ = 4-CF_3_	1:1 Non-bridged (end-on)	[**1d**·N_3_]·TBA	2.838(5), 2.905(5)	46.53(8)
6	**1e**, R^1^ = R^2^ = H	1:1 Non-bridged (end-on)	[**1e**·N_3_]·TBA	2.870(2), 2.996(2)	7.43(14)
7	**1f**, R^1^ = R^2^ = 4-Br	1:1 Non-bridged (end-on)	[**1f**·N_3_]·TBA	2.909(8), 2.923(8)	24.90(3)
8	**1g**, R^1^ = R^2^ = 4-F	1:1 Non-bridged (end-on)	[**1g**·N_3_]·TBA	2.833(3), 2.912(3)	37.13(6)
9^d^	**1h**, R^1^ = R^2^ = 4-CN	2:1 Bridged (end-on)	{[**1h**]_2_·N_3_·2H_2_O}·TBA	2.860(2), 2.976(2)	nd
10^e^	**1i**, R^1^ = R^2^ = H	1:1 Non-bridged (end-on)	[**1i**·N_3_]·TBA	2.842(2), 3.202(2)	nd
11^f^	**1j**, R^1^ = R^2^ = 3,5-CF_3_	1:1 Bridged (end-on)	{[**1j**]_2_·N_3_}·TBA	2.923(3)^d^	nd

a Calculated for the two crystallographically distinct motifs.

b This complex is side-on. The values correspond to N(1) to N(2) and N(1′) to N(4) distances, respectively.

c Under slightly different crystallization conditions, a 2:1 complex was also obtained (see Supporting Information for details).

d The second HB donor involves a single N–H in binding (N–H–N_3_ = 2.144(2) Å).

e The hydrogen bond donor is a guanidine;

f The hydrogen bond donor is an oxalyl amide, and the terminal nitrogen of the azide anion is bound to each HB donor via a single N–H. TBA = tetrabutylammonium.

The [**1a**·N_3_]·TBA complex derived from Schreiner’s urea **1a** (entry 1, Table 2) features two crystallographically distinct motifs (see Supporting Information for details) and is a 1:1 complex with the azide bound side-on. This arrangement is likely favored due to the electron-deficient 3,5-bis(trifluoromethyl)phenyl groups which allow for an additional interaction between the two terminal nitrogens of the azide and the weakly acidic aryl *ortho* C–H bonds (D–A distance: 3.364(4) Å). An additional point of interest resides in weak long-range interactions between the azide and α-C–H bonds of the tetrabutylammonium countercation.^17^ In both crystallographic motifs, the azide lies in the plane of the NC(=O)N motif of the urea (1.15(14)°; 15.79(7)°). When the same urea was bound to azide but featured Na^+^(15-crown-5) as the countercation, an end-on 1:1 complex of type II was obtained with the azide out of the urea plane (θ = 62.40(30)°, ([**1a**·azide]·[Na(15-crown-5)], entry 2, Table 2). This result underlines the role of the cation in influencing the coordination mode of the azide in the solid-state. Complex [**1a**·azide]·[Na(15-crown-5)] features the shortest and longest D–A distances observed among all complexes examined in this study (2.780(9) Å and 3.332(9) Å). Similar coordination modes were observed for [**1b**·N_3_]·TBA and [**1c**·N_3_]·TBA (entries 3 and 4, Table 2) derived from symmetrical urea **1b** featuring 3-Cl substituents, and unsymmetrical urea **1c** substituted with 3,5-bis(trifluoromethyl) group on a single aryl ring, respectively. Complexes formed with **1d**–**1g** presented two different packing arrangements, a dimeric structure whereby the urea N–H bonds point toward each other with two linking azides (entries 5 and 6, Table 2, [**1d**·N_3_]·TBA and [**1e**·N_3_]·TBA), and a structure in which the NHs of each urea point in the same direction thus forming an extended chain (entries 7 and 8, Table 2, [**1f**·N_3_]·TBA and [**1g**· N_3_]·TBA). Interestingly, [**1f**·N_3_]·TBA revealed the possibility of halogen bond interactions (3.140(2) and 3.131(2) Å) between the C–Br and the terminal nitrogen of the azide.^21^ Both urea **1f** and **1g** led to the formation of symmetry-related interdigitated antiparallel chains. For complexes derived from **1d**–**1g**, the azide is out of the NC(=O)N plane of the urea with angles in the range of θ = 24–62°, but to a lesser extend for [**1e**· N_3_]·TBA (θ = 7.43(14)). A single 2:1 urea-azide complex was obtained, which included water of crystallization (type III, entry 9, {[**1h**]_2_· N_3_·2H_2_O}·TBA). In this complex, each urea with one of its NH binds the azide while the other NH is coordinated to water, which presumably originated from TBAF·3H_2_O.

Given the range of structures that are accessible within a narrow family of urea-derived complexes, the question of whether the urea unit could be replaced by another hydrogen bonding entity arose. These queries encouraged the synthesis of additional azide complexes, two of which successfully crystallized. The guanidine-based complex [**1i**·N_3_]·TBA (type II, entry 10) crystallized as the amino rather than imino tautomer whereby both NH_2_ and NH interact with N_3_^–^ with NH_2_ binding significantly more weakly than NH (D–A distance: 3.202(2) Å versus 2.842(2) Å). The C=NPh forces the phenyl ring to bend, thus giving an angle between the two aryls of ∼63° (average values for ureas in this set: ∼6–30°). Finally, a near symmetrical complex {[**1j**]·N_3_}·TBA (entry 11) was obtained with diphenyloxalamide as HB donor. In this structure, the presence of two carbonyl groups sets the two aryl units of diphenyloxalamide in plane with the two N–H bonds that are oriented *anti* to each other. This generates a 1:1 bridged complex which is distinct from all others and in which each oxalamide unit binds a different terminal nitrogen of the azide anion (D–A distance: 2.923(3) Å).

The binding properties of **1a**–**i** with N_3_^–^ in solution were also investigated by ^1^H NMR spectroscopy. ^1^H NMR titrations were carried out by adding increasing amounts of tetrabutylammonium azide (TBA·N_3_) to a solution of HBD (CH_3_CN/CD_3_CN 8:2, at 2 mM concentration). Deshielding and broadening of ^1^H resonances ascribed to the NH groups was observed, an indicator of H-bonding interactions between azide and urea. The chemical shift variation of the aromatic signals was plotted against the concentration of added TBA·N_3_, and association constants extrapolated from nonlinear least-squares regression using Bindfit.^23^ Titration data were fitted to 1:1 and 2:1 binding isotherms; for **1a** and **1d**, the fitting was optimal when accounting for the formation of a 2:1 complex, while the 1:1 binding mode resulted in a better fit for **1c**, **1g**, **1e**, and **1i**.^15^ The association constants (*K*_a(1:1)_) for 1:1 urea-azide complexes ranged between 10^2^–10^3^ M^–1^ with the more electron-deficient diarylureas resulting in stronger binding to azide, which is consistent with the enhanced acidity of the NH groups (Table 3). The two most acidic ureas **1a** and **1d** featuring 3,5-bis(trifluoromethyl) or 4-trifluoromethyl substituents gave binding constants *K*_a(1:1)_ = 1.57 ± 0.06 × 10^3^ M^–1^, *K*_a(2:1)_ = 7 ± 3 × 10^1^ M^–1^ and *K*_a(1:1)_ = 1.25 ± 0.12 × 10^3^, *K*_a(2:1)_ = 1.3 ± 0.5 × 10^2^, respectively. Ureas **1c** (R^1^ = 3,5-CF_3_; R^2^ = H), **1g** (R^1^ = R^2^ = 4-F), and **1e** (R^1^ = R^2^ = H) presented progressively reduced binding affinity, as expected from their electronic properties. Diphenyl guanidine **1i** displayed the weakest binding among all receptors studied.

**Table 3:**
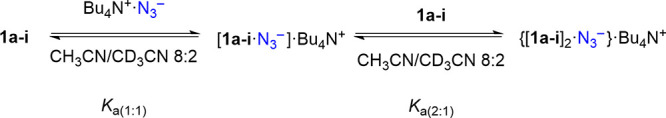
Association Constants for the Formation of 1:1, *K*_a(1:1)_, and 2:1, *K*_a(2:1)_, Complexes between Receptor **1a**–**i** and TBA·N_3_ (CH_3_CN/CD_3_CN, 2 mM), Ordered by Decreasing Strength

entry	HBD	*K*_a(1:1)_ (M^–1^)	*K*_a(2:1)_ (M^–1^)
1	**1a**	1.57 ± 0.06 × 10^3^	7 ± 3 × 10^1^
2	**1d**	1.25 ± 0.12 × 10^3^	1.3 ± 0.5 × 10^2^
3	**1c**	9.4 ± 1.7 × 10^2^	–
4	**1g**	4.82 ± 0.05 × 10^2^	–
5	**1e**	3.14 ± 0.03 × 10^2^	–
6	**1i**	1.4 ± 0.3 × 10^2^	–

These data confirm the ability of azide to engage in hydrogen bonding interactions with dual HBD donors in solution. The predominant binding mode is 1:1, with an additional weaker 2:1 binding mode observed only for the strongest donors **1a** and **1d**. Compared with complexes derived from fluoride,^24^ the binding affinity is substantially reduced, a measure of the lower propensity of azide to engage in hydrogen bonding interaction (*cf*. **1d**·F^–^ ∼ 10^5^ M^–1^, **1d**·N_3_^–^ ∼ 10^3^ M^–1^). The weaker binding of azide compared to fluoride has implications in the development of a catalytic method aimed at bringing insoluble azide salts into solution via complexation with hydrogen bond donors; while the phase-transfer of azide salts may differ from that of fluoride salts, the nucleophilicity of soluble bound urea-azide complex is expected to be less attenuated as a consequence of weaker binding to the HBD catalyst. Also, urea **1a** binds chloride (*K*_a(1:1)_ = 4.7 ± 1.6 × 10^4^ M^–1^; *K*_a(2:1)_ = 1.7 ± 0.9 × 10^2^ M^–1^) more effectively than azide (*K*_a(1:1)_ = 1.57 ± 0.06 × 10^3^ M^–1^; *K*_a(2:1)_ = 7 ± 3 × 10^1^ M^–1^), raising awareness of a possible inhibition pathway.

**Figure 4:**
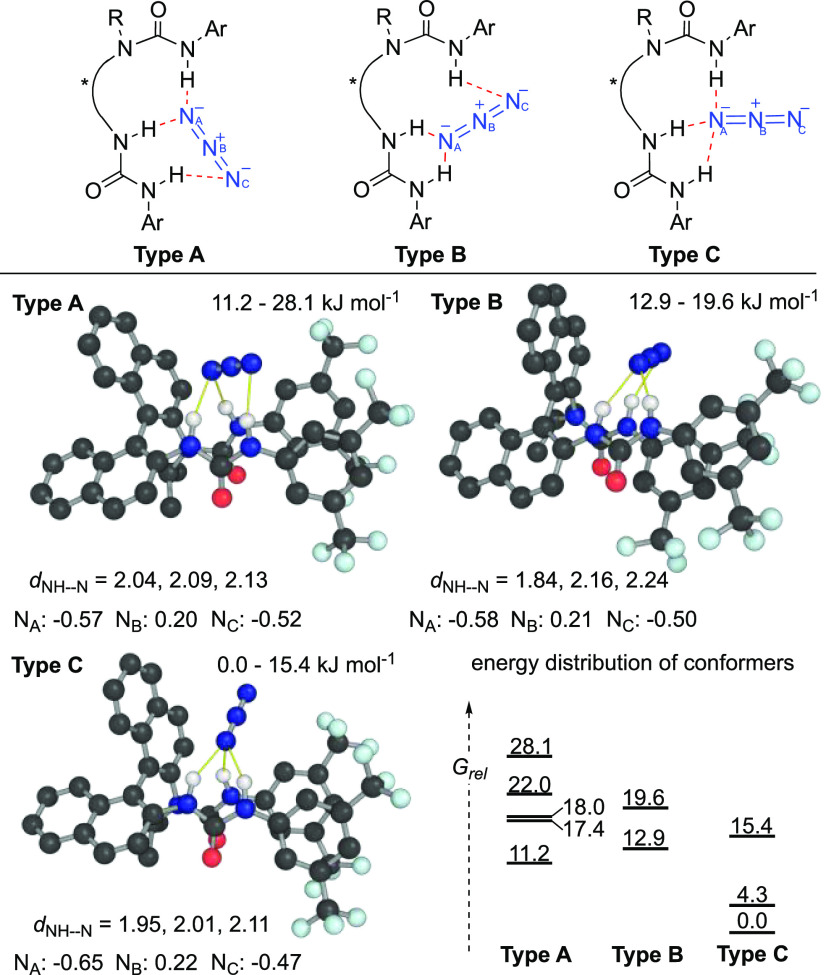
DFT computed conformers and relative Gibbs energies (kJ·mol^–1^) of the [(*S*)-**1k**·N_3_]^−^ complex. N–H distances (Å) and natural charges on azide N atoms (au) also shown.

Next, we focused on the characterization of the chiral BINAM-derived bisurea-azide complex that led to successful enantioselective azidation with sodium azide. The proposed hydrogen-bonded association between azide and (*S*)-**1k** was investigated computationally and experimentally. Conformational analysis of the solution-phase structure of a 1:1 complex formed between (*S*)-**1k** and azide was performed computationally. While our previous studies on fluoride complexation focused on the use of explicitly solvated classical molecular dynamics,^25^ here we used semiempirical GFN2-xTB calculations and the iMTD-GC workflow implemented in Grimme’s CREST for sampling including implicit solvation for dichloromethane,^26^ followed by DFT optimizations of the low energy conformers.^27^ Low-lying conformers were obtained with three NH-azide H-bonding interactions, which can be further categorized into three distinct catalyst-azide binding-modes: (i) Type A conformers show side-on binding in which the azide termini form H-bonds with proximal N–H groups in the catalyst; (ii) Type B conformers show side-on binding in which the azide termini form H-bonds to distal N–H groups; (iii) Type C conformers show end-on binding in a tripodal fashion to all three N–H bonds in **1k** (Figure 4). The *syn*,*anti*-conformation with respect to the catalyst *N*-isopropylated urea is found in these low-lying conformers.

The end-on binding mode in Type C conformers is energetically most favorable by over 10 kJ·mol^–1^, displaying the shortest average N–H distance (2.02 Å). Additionally, the quadrupole moment of the azide anion is polarized upon binding, with the terminus coordinated to the highest possible number of N–H bonds (3 for end-on; 2 for side-on), and N_A_, bearing the largest residual negative charge. In the Type C conformation, this effect is largest.

**Figure 5:**
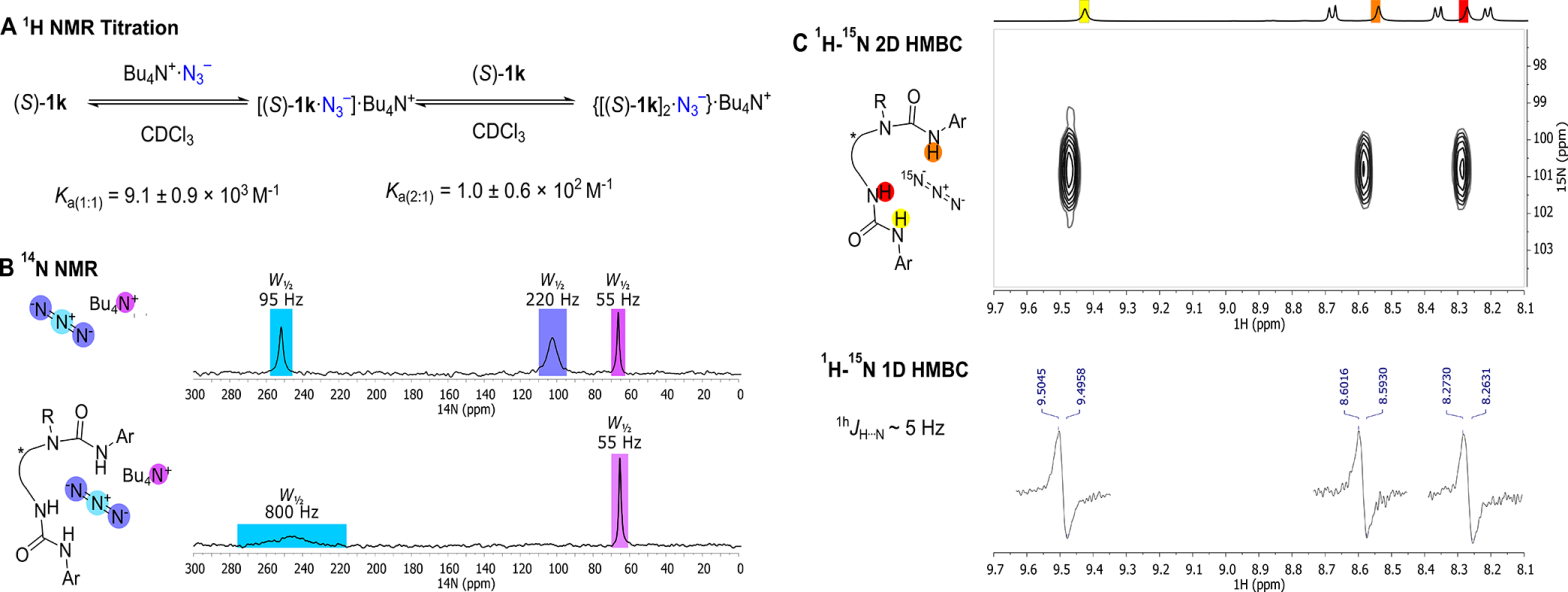
(A) *K*_a(1:1)_ and *K*_a(2:1)_ for the complexes of TBA·N_3_ with (*S*)-**1k** in CDCl_3_ (2 mM). (B) ^14^N spectra of and TBA·N_3_ complexed to 1 equiv of (*S*)-**1k** in CDCl_3_ (25 mM). (C) 1D and 2D ^1^H–^15^N HMBC spectra of (*S*)-**1k**·[1-^15^N]N_3_ (CDCl_3_, 25 mM, 213 K).

Next, ^1^H NMR titrations were conducted using TBA·N_3_ (CDCl_3_ at 2 mM concentration). Similar to achiral ureas **1a** and **1d**, a 1:1 binding model was insufficient to provide an accurate description of the system. The inclusion of a 2:1 complex ([(*S*)-**1k**]_2_·N_3^–^_) resulted in improved fits, leading to a *K*_a(1:1)_ of 9.14 ± 0.9 × 10^3^ M^–1^ and a *K*_a(2:1)_ of 1.0 ± 0.6 × 10^2^ M^–1^ (Figure 5A). This finding is analogous to fluoride, where 2:1 urea-fluoride complexes were also observed in solution.^13d^ The *K*_a(1:1)_ and *K*_a(2:1)_ for the complexes of (*S*)-**1k** with fluoride are 1.43 ± 0.04 × 10^6^ M^–1^ and 3.1 ± 0.9 × 10^3^ M^–1^ in CH_2_Cl_2_, approximately 2 orders of magnitude higher than azide. ^14^N NMR spectroscopy provided further insight. The highly symmetric environment of unbound TBAN_3_ (CDCl_3_, 25 mM) gives three signals in ^14^N NMR spectrum corresponding to the tetrabutylammonium cation (66 ppm), to the central azide nitrogen (251 ppm), and to the terminal one (102 ppm). In an equimolar mixture of (*S*)-**1k** and TBAN_3_ (CDCl_3_, 25 mM), the central azide nitrogen appears significantly broader and the signal of the terminal azide nitrogen is broadened beyond detection; negligible change is observed for the tetrabutylammonium cation (Figure 5B). ^14^N is a quadrupolar nucleus which shows sharp signals only in symmetric environments;^28^ the extreme line broadening observed for (*S*)-**1k**·TBA·N_3_ is thus consistent with a lack of symmetry of the azide anion likely resulting from an interaction with (*S*)-**1k**. Further analysis was performed using isotopically enriched tetrabutylammonium [1-^15^N]azide (TBA·[1-^15^N]N_3_). At room temperature, a sample prepared by mixing TBA·[1-^15^N]N_3_ and 1 equiv of (*S*)-**1k** (CDCl_3_, 25 mM) exhibited broad lines in ^1^H NMR supporting the existence of multiple equilibrating species present in solution, ascribed to the unbound ligand, the 1:1 [(*S*)-**1k**]·N_3^–^_ and 2:1 [(*S*)-**1k**]_2_· complexes. ^15^N NMR at room temperature shows one resonance at 101.8 ppm. Steady-state heteronuclear ^1^H–^15^N NOE experiments provided evidence of azide binding to the NH groups of (*S*)-**1k**. Selective irradiation of the urea NH protons resulted in a decrease of ^15^N signal intensities, whereas irradiation of the CH protons gave no evidence of heteronuclear NOEs, supporting the proposal that the urea NH protons are responsible for azide coordination.^15^ At 213 K a sample prepared using (*S*)-**1k** (CDCl_3_, 25 mM) and two equivalents of TBA[1-^15^N]N_3_ shows sharp resonances in ^1^H NMR, with a major species assigned to the 1:1 complex **1k**·[1-^15^N]azide and a minor species which is consistent with the 2:1 complex [(*S*)-**1k**]_2_·azide, as noted in the course of the ^1^H NMR titrations. At this temperature, applying a ^1^H–^15^N HMBC sequence, it was possible to detect couplings across hydrogen bonding (^1h^*J*_NH_) between the three NH groups and [1-^15^N]N_3_^–^ (Figure 5C). The magnitude of ^1h^*J*_NH_, measured using 1D ^1^H^15^N HMBC, was found to be around 5 Hz, although accurate measurement was hindered by the line width of the cross-peaks. This value is consistent with those reported for ^15^N-labeled DNA duplex, which are typically in the range of 1–4 Hz.^29^ The data obtained by ^1^H–^15^N NOE and HMBC experiments unambiguously indicate coordination of the azide with all three NH hydrogen bond donors in solution.

**Figure 6:**
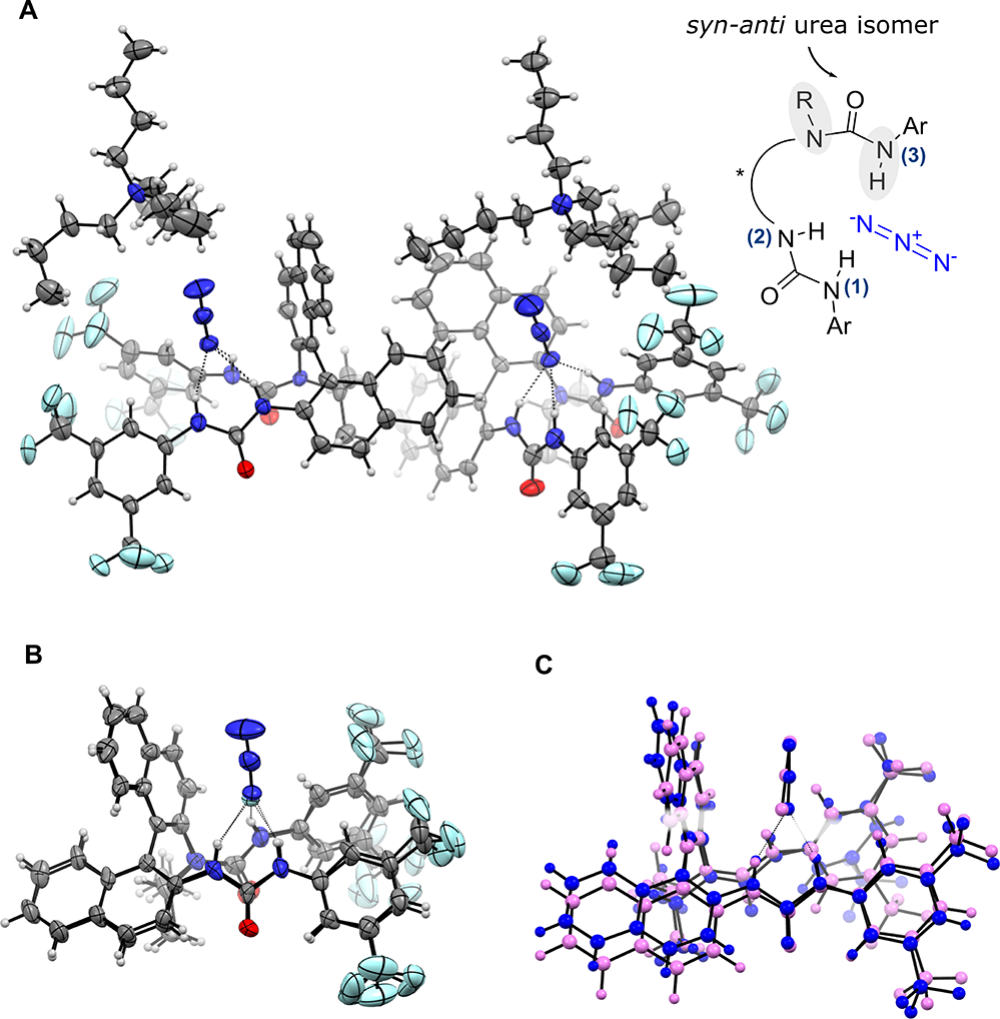
(A) Asymmetric unit of a Z′ = 2 crystal structure consisting of both (*R*)-**1k** and (*S*)-**1k** complexed to tetrabutylammonium azide. (B) View of (*S*)**-1k** complexed to azide. Distances provided in Ångstroms, displacement ellipsoids drawn at 50% probability level. (C) Overlay of [**1k**·N_3_]^−^ (DFT vs X-ray).

Further insight on the nature of the complexation of (±)-**1k** with azide was obtained in the solid-state. A sample of (±)-**1k** complexed to TBAN_3_ (1:1 ratio) was prepared by stirring both components in MeCN (0.1 M) and subsequently evaporating the mixture to dryness. Crystals of (±)-**1k**·TBAN_3_ suitable for single crystal X-ray diffraction were successfully grown by slow evaporation of a saturated solution of the amorphous solid in hot hexane and EtOAc. In a single asymmetric unit cell, both enantiomers (*R*)- and (*S*)-**1k** were observed, each complexed to azide. In both enantiomeric complexes, the azide anion is coordinated at one terminus by three hydrogen bonds from the NH groups of one catalyst unit. As previously observed for the corresponding fluoride complex,^13a−13d^ the *N*-isopropylated urea adopts a *syn-anti* conformation with the *i*Pr group pointing away from the chiral pocket, thus allowing for azide to interact with the three NHs (Figure 6A). Comparison with (*S*)-**1k**·tetrabutylammonium fluoride revealed similar geometries, although the donor–acceptor distances^22a^ for the azide complex were consistently longer (by ∼0.2–0.4 Å) than observed for fluoride (Figure 6B, Table 4). In (*S*)-**1k**·TBAF, the relative N(H)···F donor–acceptor distances were NH(3)···F ∼ NH(1)···*F* < NH(2)···F; a reversal of these distances is found in (*S*)-**1k**·TBAN_3_, with NH(1)···N_3_ < NH(2)···N_3_ < NH(3)···N_3_ (Table 4). The crystal structure is analogous to the computed Type C coordination mode, which shows end-on binding of the azide (Figure 6C, Table 4).

**Table 4:** Donor–Acceptor N(H)···X^–^ Bond Distances^22a^ of **1k**·TBAN_3_

NH···X^–^	X-ray (±)-**1k**·N_3_^–^*d* N···N_3_^–^ (Å)	X-ray (*S*)-**1k**·F^–^*d* N···F^–^ (Å)^13a^	DFT (*S*)-**1k**·N_3_^–^*d* N···N_3_^–^ (Å)
**1**	2.81(2) (*S*), 2.81(2) (*R*)	2.667(2)	2.90(4)
**2**	2.94(2) (*S*), 2.91(2) (*R*)	2.690(2)	2.95(2)
**3**	2.98(2) (*S*), 3.02(2) (*R*)	2.662(2)	3.03(2)

### 3 Mechanistic Insight from Kinetic and Computational Studies

**Figure 7:**
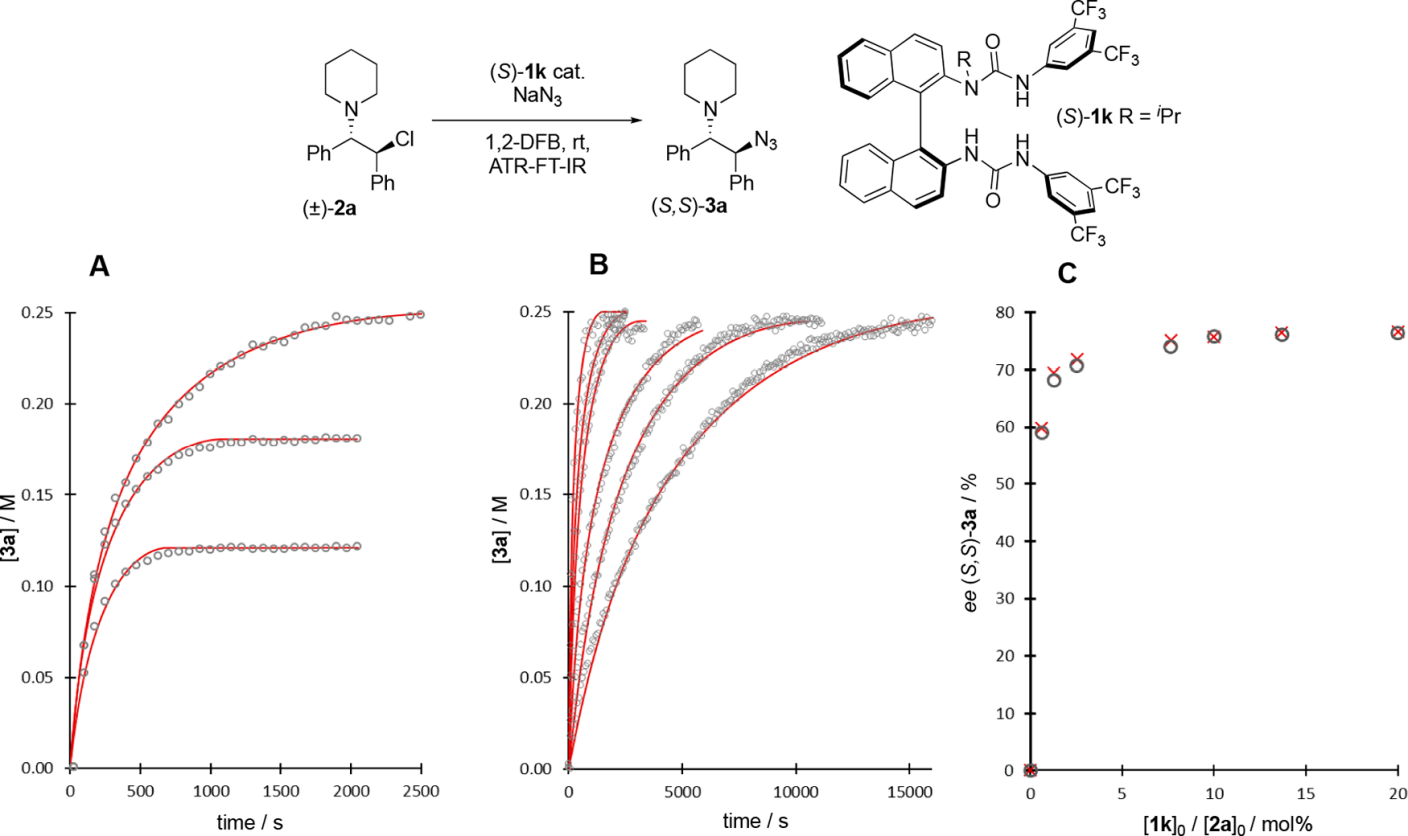
In situ ATR-FTIR analysis of the reaction of **2a** with {NaN_3_}_s_ in 1,2-difluorobenzene, catalyzed by (*S*)-**1k**. Data, open circles. Kinetic model (eq 2), solid red lines/crosses. (A) Temporal growth of [**3a**] from [**2a**]_0_ = 0.25, 0.18, and 0.13 M, at [(S)-**1k**]_0_ = 0.025M. (B) Temporal growth of [**3a**] from [**2a**]_0_ = 0.25 M, at catalyst (S)-**1k** loadings (mol %) indicated. (C) Net enantiomeric excess of (*S*,*S*)-**3a** at catalyst (S)-**1k** loadings (0.6, 1.3, 2.6, 7.7, 10, and 20 mol %). Constants used for fitting eq 2: *a* = 0.081(±0.018) M^–0.5^ s^–1^; *b* = 2.1(±0.9); *c* = 1.8(±0.5) × 10^–5^ M^0.5^ s^–1^; *r* = {NaN_3_}_s_/{NaCl}_s_. Enantioselectivity employed in all fits as *a*_S,S_/*a*_R,R_ = 7.94 (e.r. = 88.8:11.2) and *c*_S,S_/*c*_R,R_ = 1.00 (e.r. = 50:50).

To shed light on the reaction mechanism, we explored the kinetics of the reaction of β-chloroamine (±)-**2a** with NaN_3_ in 1,2-difluorobenzene, in the presence and absence of catalyst (*S*)-**1k** at ambient temperature. The growth of the substitution product (**3a**) was monitored by in situ ATR-FT-IR, analyzing the absolute intensity of the signal arising from organoazide stretching band at 2100 cm^–1^. After some initial optimization of conditions,^15^ the reactions gave kinetics that were sufficiently reproducible for further analysis (Figure 7). The temporal concentration profiles for product **3a** obtained at a series of different initial concentrations of **2a** and **1k** were investigated using a series of simple models that included the net enantioselectivity.^15^ Detailed kinetic analysis was precluded by the absence of information on catalyst speciation from the in situ FT-IR spectra, and by the solid-phase form of the sodium azide reactant and sodium chloride coproduct; {NaN_3_}_s_ and {NaCl}_s_, from the overall reaction, eq 1. Nonetheless, three key features that govern the reaction evolution emerged: (i) the rate of turnover has a first-order dependency on the initial concentration of catalyst, [(*S*)-**1k**]_0_; (ii) the rate of turnover has a fractional order (∼0.5) dependency on the temporal concentration of the substrate, [**2a**]_*t*_; and (iii) as the reactions proceed, the rate of turnover is attenuated to a greater degree than dictated by the progressive reduction in the quantities of the reactants (**2a** and {NaN_3_}_s_). The latter is consistent with inhibition by accumulation of {NaCl}_s_.^15^
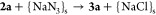
1
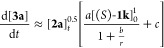
2

**Figure 8:**
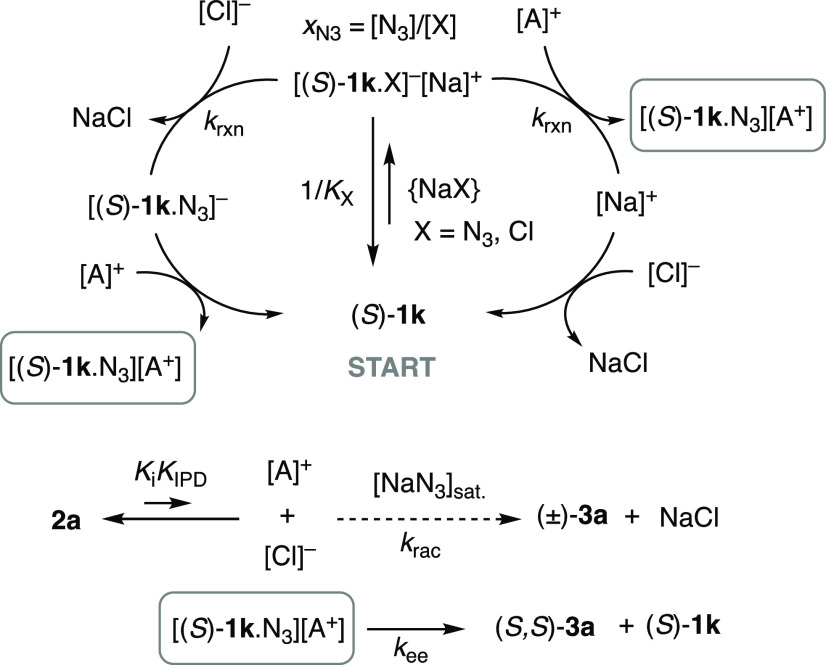
Two pathways for generation of **3a** from NaN_3_ and **2a** catalyzed by **1k**, in competition with a racemic background reaction. The pathways are kinetically equivalent in the context of eq 2.^15^*x*_N3_ is the mole fraction of {[**1k**·X]^−^[Na^+^]}in which X is azide.

The temporal concentration profiles for [**3a**] can be satisfactorily correlated (Figure 7A and B) using the simple empirical relationship shown in eq 2. When *a*_S,S_/*a*_R,R_ = 7.94 (e.r. = 88.8:11.2) and *c*_S,S_/*c*_R,R_ = 1.00 (e.r. = 50:50), eq 2 also correctly predicts the net enantioselectivity for (*S*,*S*)-**3a** as a function of catalyst loading (Figure 7C). eq 2 is consistent with two processes operating in parallel: one enantioselective, and one a background racemic reaction. Their relative flux, and thus the net enantioselectivity, is governed by the initial concentrations of substrate **2a** and catalyst **1k**, the proportions of reactants, the extent of conversion, and the magnitude of constants *a*, *b*, and *c*. Two kinetically equivalent processes that are consistent with the empirical eq 2 are shown in Figure 8. Both processes involve competing complexation (*K*_X_; X = N_3_ or Cl) of catalyst **1k** with either azide or chloride ion, and a pre-equilibrium (*K*_i_*K*_IPD_) involving **2a** that generates ion-pair separated aziridinium ([A]^+^) cation and chloride anion. The two pathways diverge in the sequence of their reaction of {[**1k**·X]^−^[Na^+^]} where X = N_3_, with [A]^+^ and [Cl]^−^. But in both cases, they lead to the same key species: an aziridinium/catalyst-bound azide ion pair {[**1k**·N_3_][A^+^]}. This rapidly and irreversibly generates (*k*_ee_) the product **3a** in 89:11 e.r. The fitting parameter “*a*” reflects the series of equilibria and reactions that lead to {[**1k**·N_3_][A^+^]}. The fitting parameter “*b*” reports the differential binding of chloride over azide to the catalyst, in an equilibrium that is limited by the common-ion Na^+^. The term “*r*” reflects the evolving stoichiometry ratio {NaN_3_}_s_/{NaCl}_s_. The fitting parameter “*c*” reports on the rate of the competing background racemic (e.r. = 50:50) process involving direct reaction (*k*_rac_) of the aziridinium ([A]^+^) cation with azide.

Alternative approaches involving more complex models and holistic simulations were also effective, but did not prove advantageous, or allow elucidation of any discrete kinetic constants. Conducting reactions in the presence of exogenous NaCl led to the expected changes in rate and enantioselectivity.^15^ Other general mechanisms, where catalyst **1k** interacts first with the substrate **2a**, in its neutral or ionized forms, are inconsistent with eq 2.^15^ Overall, the kinetics support a process where the turnover rate limiting event directly, or indirectly, results in the generation of an ion pair {[**1k**·N_3_][A^+^]} containing an aziridinium cation and a catalyst-bound azide anion. The substitution product (*S*,*S*)-**3a** is then generated in excess over (*R*,*R*)-**3a** through enantiocontrol by ligand ((*S*)-**1k**) that is coordinated to the azide being delivered to the aziridinium cation as the ion-pair collapses.

**Figure 9:**
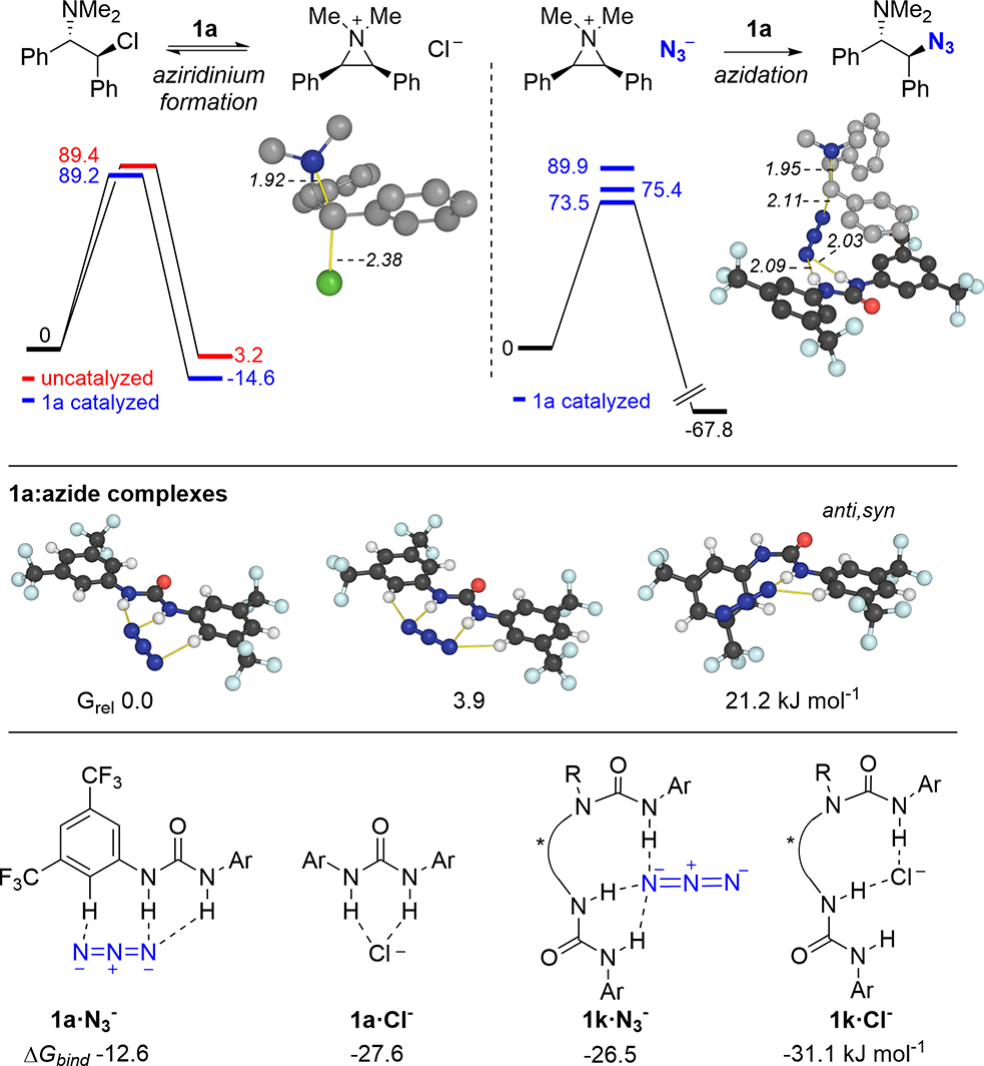
DFT computed transition structures for aziridinium formation, azidation with **1a**·N_3_^–^, and binding of azide vs chloride to catalysts **1a** and **1k**.^30^ Distances in Å and energies in kJ·mol^–1^.

**Figure 10:**
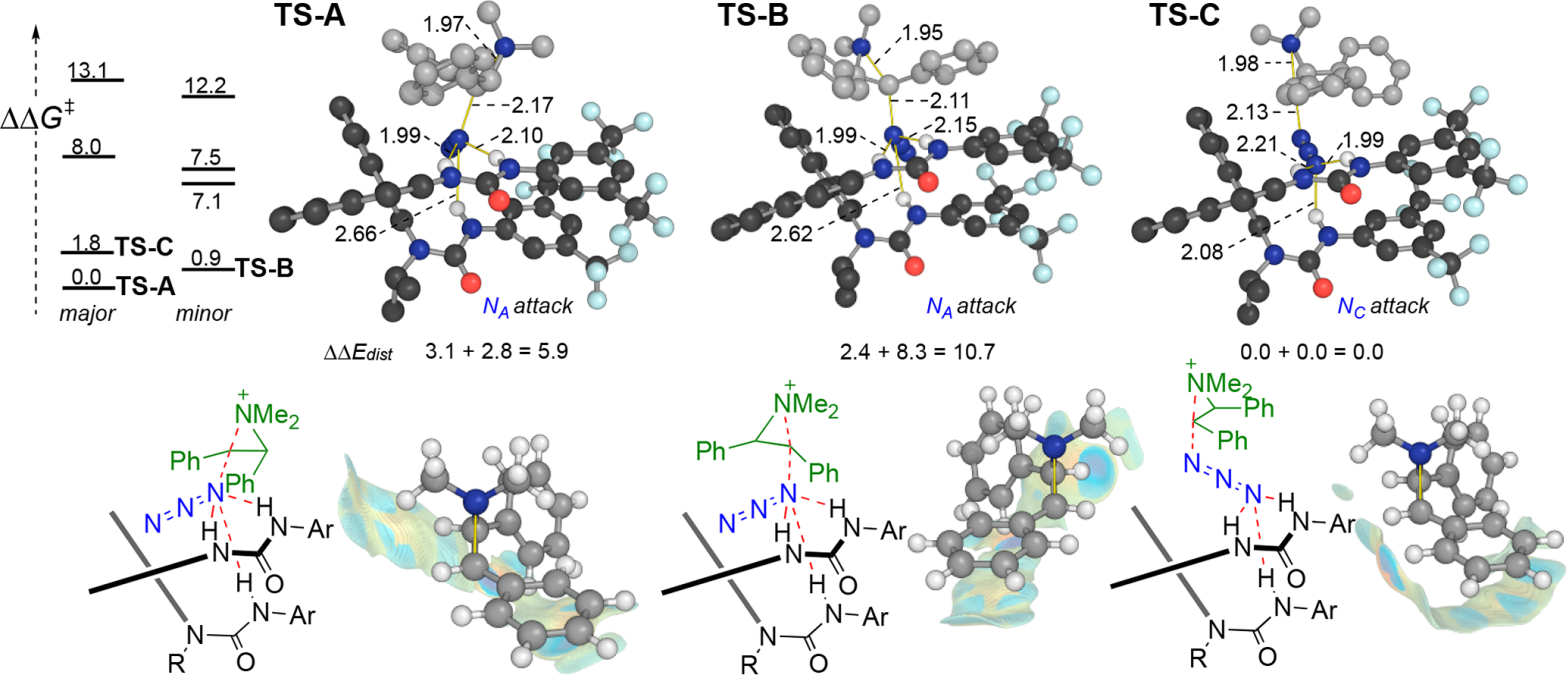
Low-lying enantiodetermining azidation TSs with (*S*)-**1k**. Relative distortion energies of aziridinium and [(*S*)-**1k**·N_3_]^−^ fragment in each TS shown. The reduced density gradient isosurface (RDG = 0.3) around the substrate is shown to indicate qualitatively the extent of substrate-catalyst noncovalent interactions in each TS.

Computationally, we studied several elementary steps in the proposed azidation mechanism. First, we considered the achiral transformation with catalyst **1a** (Figure 9) and subsequently the enantioselective reaction with **1k** (Figure 10).

Transition structures (TSs) were located for aziridinium formation in the absence and presence of the achiral urea catalyst **1a**. Little energetic difference (0.2 kJ·mol^–1^) was found between these pathways, which corroborates previous computational studies.^13^ Consistent with the kinetic model above, aziridinium formation by autoionization is computed to be feasible and reversible, with ion-pair formation endergonic by 3 kJ·mol^–1^. Azidation TSs were also located for the addition of the urea-bound azide anion. Barrier heights are lower than for aziridinium formation by >15 kJ·mol^–1^, and the addition of azide is computed to occur irreversibly, with product formation exergonic by 68 kJ·mol^–1^. We also considered the relative stabilities of chloride and azide bound urea catalyst. For **1a**, we located three distinct azide binding modes, of which the end-on structure is most stable. Chloride binding, however, is computed to be more favorable by 15 kJ·mol^–1^ (−27.6 vs −12.6 kJ·mol^–1^). Interestingly, the differential binding of chloride over azide for catalyst **1k** is reduced to 4 kJ·mol^–1^, since azide anion is able to form three N–H bonds. This value is consistent with the kinetic model developed for **1k**.

In order to understand the origins of asymmetric induction in azidation promoted by (*S*)-**1k**, we computed the competing TSs for the enantiodetermining step. We manually located a TS for the formation of major and minor enantiomer products, followed by a constrained conformational search with CREST. This produced 168 major and 219 minor structures,^15^ from which we finally obtained eight DFT-optimized (using the same methodology described above) energetically low-lying TSs within 14 kJ·mol^–1^ of the most stable structure (Figure 10). The two most stable competing TSs (TS-A and TS-B) involve attack from the H-bonded end of the azide anion (N_A_), while the remaining six higher energy structures are characterized by the distal nitrogen N_C_ as the reactive center of the nucleophile, of which TS-C is the most stable example. Attack from N_C_ results in a bridged structure in which there is comparatively little geometric distortion of the catalyst and substrate in the TS (relative distortion energies are shown in Figure 10). However, in the two most stable structures, attack from N_A_ results in more significant noncovalent interactions between the substrate and catalyst—as can be seen qualitatively from the extent of the RDG isosurface produced by NCI plot in each of the TSs. These arise from several dispersive interactions between aromatic rings (both face-to-face and edge-to-face are evident) as well as CH(substrate)-π(catalyst) interactions. All three H-bonds are retained in the TS, however, as seen in asymmetric HB-PTC fluorination, the H-bond between nucleophile and the monodentate urea most noticeably lengthens along the reaction coordinate. The Boltzmann-averaged enantioselectivity arising from these low-lying structures is 69:31 in favor of the major enantiomer observed experimentally (Table 1, entry 5).

## Conclusion

In this work, we have described the expansion of hydrogen-bonding phase-transfer catalysis and the privileged BINAM-derived bisurea catalyst scaffolds to the recognition of an anion other than fluoride for applications in catalysis. By employing a linear anion such as azide rather than the spherical charge-dense fluoride anion, we have demonstrated that the bisurea catalyst acts as an azide receptor and enables enantioselective azidation of β-chloroamine-derived *meso* aziridinium electrophiles using sodium azide. Kinetic studies support a process with the turnover rate limiting event that directly or indirectly generates an ion pair containing an aziridinium cation and a catalyst-bound azide anion, with catalyst inhibition incurred by accumulation of NaCl. Structural data in the solid state and in solution of a range of hydrogen bonded azide complexes inform that azide end-on binding is more often observed. For the chiral monoalkylated bisurea catalyst, ^1^H–^15^N NOE and HMBC experiments in solution as well as data in the solid state arising from single crystal X-ray diffraction analysis indicate coordination of the azide with all three NH hydrogen bond donors. Computationally, azide end-on bound to all three NH bonds of the BINAM urea in a tripodal fashion is found to be energetically most favorable. This binding mode induces polarization of the azide ion with the bound nitrogen bearing the largest negative charge, which indicates that this nitrogen is amenable to electrophilic attack. This analysis corroborates with the features of the most stable transition state leading to the major enantiomer. More generally, this study highlights the potential of hydrogen bonding phase transfer (HB-PTC) catalysis beyond fluorination and as a general activation mode for abundant alkali metal salts as reagents in asymmetric synthesis.

## Supporting Information Available

Additional data (TXT)
Experimental details, kinetics, characterization data, NMR spectra, and full computational details (PDF)

## Accession Codes

CCDC 2129236–2129248 contain the supplementary crystallographic data for this paper. These data can be obtained free of charge via www.ccdc.cam.ac.uk/data_request/cif, or by emailing data_request@ccdc.cam.ac.uk, or by contacting The Cambridge Crystallographic Data Centre, 12 Union Road, Cambridge CB2 1EZ, UK; fax: +44 1223 336033.
