# Supplementary material for: Asymmetric Azidation under Hydrogen Bonding Phase-Transfer Catalysis: A Combined Experimental and Computational Study
Source: J Am Chem Soc. 2022 Mar 1;144(10):4572–84. doi: 10.1021/jacs.1c13434 (PMC8931729; doi:10.1021/jacs.1c13434)
Supplement: Supplementary file 2 — ja1c13434_si_002.pdf [file ja1c13434_si_002.pdf]

Asymmetric Azidation under Hydrogen Bonding Phase-Transfer Catalysis: A Combined Experimental and Computational Study

Supporting Information

Jimmy Wang,[1‡] Matthew A. Horwitz,[1‡] Alexander B. Dürr,[1‡] Francesco Ibba,[1] Gabriele Pupo,[1] Yuan Gao,[2] Paolo Ricci,[1] Kirsten E. Christensen,[1] Tejas P. Pathak,[3] Timothy D. W. Claridge,[1*] Guy C. Lloyd-Jones,[2*] Robert S. Paton,[4*] Véronique Gouverneur[1*]

[1]Chemistry Research Laboratory, University of Oxford, 12 Mansfield Road, Oxford, OX1 3TA, U.K.

[2]School of Chemistry, University of Edinburgh, Edinburgh EH9 3FJ, U.K.

[3]Novartis Institutes for Biomedical Research, 22 Windsor Street, Cambridge, MA 02139, USA.

[4]Department of Chemistry, Colorado State University, Fort Collins, CO 80528, USA.

‡ These authors contributed equally to this work.

*To whom correspondence should be addressed:

tim.claridge@chem.ox.ac.uk

guy.lloyd-jones@ed.ac.uk

robert.paton@colostate.edu

veronique.gouverneur@chem.ox.ac.uk

Contents

Contents ii

General Considerations S3

Synthesis and characterisation for structural studies S5

Synthesis of hydrogen bond donors and acceptors S5

Synthesis of hydrogen bonded azide complexes S7

Reactivity study of selected achiral urea catalysts and comparison with their corresponding $K_{a(1:1)}$ binding constants S13

(*S*)-1k ^{1}H assignment ($CDCl_3$) S14

^{1}H NMR Titrations S21

^{14}N Studies S40

Study of (*S*)-1k·[1-^{15}N]N_3·Bu_4N S44

General procedures for enantioselective azidation S72

Reaction screening/optimization S74

Synthesis of substrates S75

Synthesis of products S89

Product derivatization S100

Non-linear effect study S102

ReactIR data collection S103

Development and Evaluation of Empirical Rate Equation S105

Control experiments with additional NaCl S118

Computational Methods S119

NMR spectra S137

HPLC traces S282

References S304

General Considerations

All reagents and solvents were purchased from commercial suppliers and used without further purification unless stated otherwise. Tetrabutylammonium azide was purchased from Sigma-Aldrich, dried over P_2O_5 and stored under nitrogen. Dry THF, CH_2Cl_2, and MeCN were obtained from a MBRAUN SPS-5 bench-top unit. $CDCl_3$ was purchased from Sigma-Aldrich and stored over K_2CO_3.

Reactions requiring prolonged refrigeration were kept cold with an ethanol or isopropyl alcohol bath cooled using a Thermo Scientific HAAKE EK immersion cooler.

Melting points were measured with a Gallenkamp melting point apparatus equipped with a mercury 76 mm partial immersion thermometer.

Infrared spectra were recorded on a Bruker Tensor 27 FT-IR spectrometer equipped with a diamond ATR module.

NMR spectra for characterisation were obtained on Bruker AVANCE III HD 400 and 500 MHz spectrometers. Chemical shifts are reported in ppm and coupling constants are reported in Hz and rounded to the nearest 0.5 Hz. ^{1}H and ^{13}C chemical shifts are referenced to the appropriate residual solvent signal.[1] ^{19}F chemical shifts were observed directly and referenced to external $CFCl_3$. ^{15}N chemical shifts were observed directly and referenced to external $NH_{3(l)}$.

High resolution mass spectra were recorded by the University of Oxford, Department of Chemistry High Resolution Sample Submission Service. High resolution values are calculated to four decimal places from the molecular formula, and all values are within a tolerance of 5 ppm.

Specific rotations were recorded on a UniPol L2000 Polarimeter with a path length of 10 cm using the sodium D-line (589 nm). Specific rotations are reported in units of ° $dm^2\ g^{-1}$. Concentration (c) is given in g/100 mL.

Analytical chiral HPLC was performed on a SHIMADZU Prominence-*i* LC2030-LT instrument.

Azide quench

Glassware and equipment contaminated with azide (from NaN_3, $Bu_4N{\cdot}N_3$ etc.) were washed with water into a glass flask and quenched according to a modified literature procedure.[2]

The collected washings were diluted such that the azide concentration was <5% (w/w). A >40% excess (with respect to azide) of $NaNO_2$ (20% in H_2O) was added, followed by careful addition of H_2SO_4 (20% in H_2O) with gentle stirring. This generates nitrous acid *in situ*, which reacts readily with sodium azide to form N_2O and N_2 gas.[3] The order of addition is important to avoid releasing hydrazoic acid. After gas evolution ceased, the solution was checked with litmus and starch-iodide paper to ensure it was acidic and contained excess nitrite. The mixture was then disposed of as standard aqueous waste.

Synthesis and characterisation for structural studies

General procedure 1 (GP1, synthesis of azide complexes)

Dry MeCN (0.10 M) was added to tetrabutylammonium azide (1.00 equiv) and hydrogen bond donor (1.00 equiv). The reaction mixture stirred at rt for 24 h. The reaction mixture was evaporated to dryness to afford an amorphous solid. The solids obtained were used without further purification.

General procedure 2 (GP2, achiral reaction screening)

1,2-difluorobenzene (0.20 mL, 0.25 M) was added to a 1.75 mL vial charged sequentially with **2a** (15 mg, 0.05 mmol, 1.00 equiv), catalyst (0.10 equiv), and sodium azide (3.9 mg, 0.06 mmol, 1.20 equiv), and in a 1.75 mL vial. The reaction mixture was stirred at rt and 1200 rpm for 90 min. The reaction mixture was filtered through silica, eluted with Et_2O, and concentrated. Yield was determined by quantitative ^1H NMR with Ph_3CH (6.1 mg, 0.025 mmol, 0.50 equiv) as internal standard.

Synthesis of hydrogen bond donors and acceptors

1a,[4] **1b**,[5] **1c**,[4] **1d**,[6] **1f**,[6] **1g**,[6] **1h**,[7] and (*S*)-**1k**,[8] were prepared according to the reported literature procedures.

N^1,*N*^{2}-bis(3,5-bis(trifluoromethyl)phenyl)oxalamide (1j)

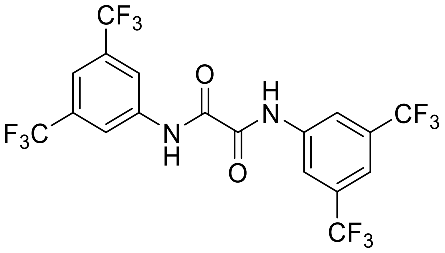

Oxalyl chloride (0.43 mL, 5.1 mmol, 0.51 equiv) was added to a solution of 3,5-bis(trifluoromethyl)aniline (1.56 mL, 10 mmol, 1.00 equiv) in dry THF (20 mL) under N_2. The reaction mixture was heated to reflux for 18 h. The reaction mixture was concentrated to dryness to afford crude product. The crude solid was washed with pentane, then dried to afford the title compound (2.04 g, 3.98 mmol, 80%) as a white solid.

mp 263-265 °C; ν_{max} (neat) /cm^{-1} 2161, 2033, 1676, 1543, 1375, 1274, 1129, 891, 681; **^{1}H NMR** (400 MHz, Acetone-d_6) δ 10.73 (s, 2H), 8.61 (s, 4H), 7.84 (s, 2H); **^{19}F NMR** (377 MHz, Acetone-d_6) δ -63.72; **^{13}C NMR** (101 MHz, Acetone-d_6) δ 159.2, 140.3, 132.7 (q, J = 33.5 Hz), 124.3 (q, J = 272.0 Hz), 121.2 (q, J = 4.0 Hz), 118.8 (p, J = 4.0 Hz); **HRMS** (ESI$^-$) calc. for $C_{18}H_7F_{12}N_2O_2^+$ ([M-H]$^-$): 511.0321; found: 511.0317.

(±)-N^2-isopropyl-[1,1'-binaphthalene]-2,2'-diamine

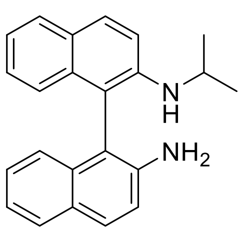

Prepared according to the literature procedure for (*S*)-N^2-isopropyl-[1,1'-binaphthalene]-2,2'-diamine using (±)-1,1'-bi(2-naphthylamine) instead of (*S*)-1,1'-bi(2-naphthylamine).[8]

NB: All spectra identical to (*S*)-N^2-isopropyl-[1,1'-binaphthalene]-2,2'-diamine.[8]

(±)-3-(3,5-bis(trifluoromethyl)phenyl)-1-(2'-(3-(3,5-bis(trifluoromethyl)phenyl)ureido)-[1,1'-binaphthalen]-2-yl)-1-isopropylurea ((±)-1k)

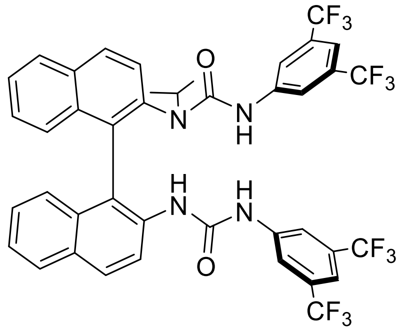

Prepared according to the literature procedure for (*S*)-**1k** with (±)-N^2-isopropyl-[1,1'-binaphthalene]-2,2'-diamine instead of (*S*)-N^2-isopropyl-[1,1'-binaphthalene]-2,2'-diamine.[8]

NB: All spectra identical to (*S*)-**1k**.[8]

Tetrabutylammonium [1-^{15}N]azide

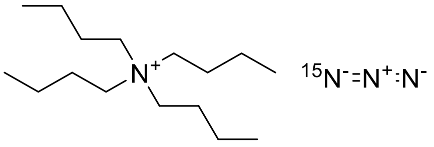

Prepared according to an adapted literature procedure.[9] Tetrabutylammonium hydroxide (40% in H_2O, 2.76 mL, 4.24 mmol, 1.1 equiv) was washed with CH_2Cl_2 (2 mL). The aqueous layer was added to a solution of sodium [1-^{15}N]azide (250 mg, 3.85 mmol, 1 equiv) in H_2O (1 mL) in a separatory funnel. The aqueous layer was extracted with $CHCl_3$ (3×5 mL). The organic layer was dried over $MgSO_4$, filtered and concentrated to afford crude product. The crude product was concentrated under reduced pressure, then evaporated to dryness under reduced pressure (0.05 mbar) over P_2O_5 to afford the title compound as a white solid, used without further purification (1.03 g, 3.61 mmol, 94%). The title compound was stored under nitrogen.

ν_{max} (neat) /cm^{-1} 2959, 2873, 1983, 1489, 885, 743; **^{1}H NMR** (500 MHz, $CDCl_3$) δ 3.40 – 3.24 (m, 8H), 1.74 – 1.62 (m, 8H), 1.52 – 1.37 (m, 8H), 1.02 (t, *J* = 7.5 Hz, 12H); **^{13}C NMR** (126 MHz, $CDCl_3$) δ 58.8, 24.1, 19.9, 13.8; **^{15}N NMR** (51 MHz, $CDCl_3$) δ 101.2.

NB: Only ^{15}N label on terminal azide observed on ^{15}N NMR.

Synthesis of hydrogen bonded azide complexes

1,3-bis(3,5-bis(trifluoromethyl)phenyl)urea·tetrabutylammonium azide (1a·N_3·Bu_4N)

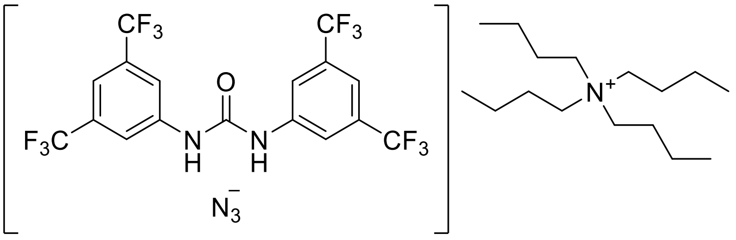

Prepared according to GP1 with 1,3-bis(3,5-bis(trifluoromethyl)phenyl)urea (484 mg, 1.00 mmol, 1.00 equiv) to afford the title compound as a yellow solid (732 mg, 0.95 mmol, 95%). Single crystals of [**1a**·N_3]·TBA suitable for X-ray diffraction were grown by vapour diffusion of cyclohexane into a saturated solution of the title compound in EtOAc.

mp 100-102 °C; ν_{max} (neat) /cm^{-1} 2961, 2004, 1706, 1574, 1471, 1376, 1273, 1229, 1170, 1124, 927, 880, 682; **^{1}H NMR** (400 MHz, Acetone-*d*$_6$) δ 10.62 (s, 2H), 8.23 (s, 4H), 7.57 (s, 2H), 3.51 – 3.35 (m, 8H), 1.81 (p, *J* = 8.0 Hz, 8H), 1.40 (h, *J* = 7.5 Hz, 8H), 0.95 (td, *J* = 7.5, 1.2 Hz, 12H); **^{19}F NMR** (377 MHz, Acetone-*d*$_6$) δ -63.55; **^{13}C NMR** (101 MHz, Acetone-*d*$_6$) δ 153.9, 143.0, 132.6 (q, *J* = 33.0 Hz), 124.6 (q, *J* = 272.0 Hz), 118.8 (d, *J* = 4.0 Hz), 115.5 (p, *J* = 4.0 Hz), 59.4 (t, *J* = 3.0 Hz), 24.4, 20.4, 13.9.

1,3-bis(3,5-bis(trifluoromethyl)phenyl)urea·sodium azide·15-crown-5 (1a·N_3·Na·15-crown-5)

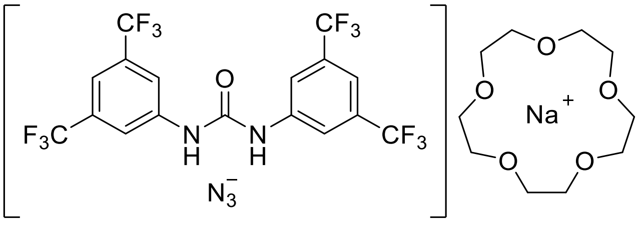

15-crown-5 (0.099 mL, 0.50 mmol, 1.00 equiv) was added to sodium azide (32.5 mg, 0.50 mmol, 1.00 equiv) and 1,3-bis(3,5-bis(trifluoromethyl)phenyl)urea (1.00 mmol, 2.00 equiv) in dry MeCN (5 mL). The reaction mixture was stirred at rt for 24 h. The reaction mixture was evaporated to dryness to afford the title compound (385 mg, 0.50 mmol, *quant*) as a white solid. Single crystals of the [**1a**·N_3]·[Na(15-crown-5)] suitable for X-ray diffraction were grown by layering cyclohexane onto a saturated solution of the title compound in THF. Single crystals of {[**1a**]$_2$·N_3}·[Na(15-crown-

5)] suitable for X-ray diffraction were grown by layering PhMe onto a saturated solution of the title compound in MeCN.

mp 126-128 °C; **ν_{max}** (neat) /cm^{-1} 2921, 2068, 1706, 1471, 1280, 1113, 927, 682; **^{1}H NMR** (500 MHz, Acetone-*d*$_6$) δ 10.40 (br s, 2H), 8.23 (s, 4H), 7.58 (s, 2H), 3.70 (s, 20H); **^{19}F NMR** (470 MHz, Acetone-*d*$_6$) δ -63.56; **^{13}C NMR** (126 MHz, Acetone-*d*$_6$) δ 158.0, 147.1, 136.8 (q, *J* = 33.0 Hz), 128.8 (q, *J* = 272.0 Hz), 123.9 – 122.5 (m), 120.2 – 119.4 (m), 74.1.

1,3-bis(3-chlorophenyl)urea·tetrabutylammonium azide (1b·N_3·Bu_4N)

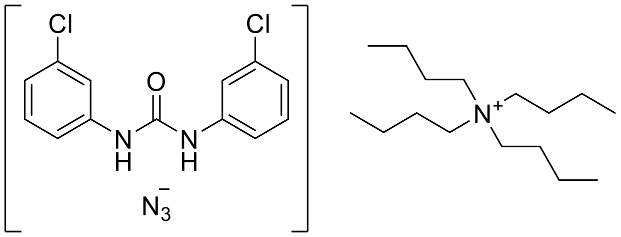

Prepared according to GP1 with 1,3-bis(3-chlorophenyl)urea (140 mg, 0.50 mmol, 1.00 equiv) to afford the title compound as a yellow solid (281 mg, 0.50 mmol, *quant*.). Single crystals suitable for X-ray diffraction were grown by layering cyclohexane onto a saturated solution of the title compound in EtOAc.

mp 67-69 °C; **ν_{max}** (neat) /cm^{-1} 2961, 2015, 1709, 1587, 1537, 1195, 879, 783, 713; **^{1}H NMR** (400 MHz, CD_3CN) δ 9.33 (s, 2H), 7.72 (t, *J* = 2.0 Hz, 2H), 7.31 (ddd, *J* = 8.5, 2.0, 1.0 Hz, 2H), 7.24 (t, *J* = 8.0 Hz, 2H), 6.97 (ddd, *J* = 8.0, 2.0, 1.0 Hz, 2H), 3.11 – 3.01 (m, 8H), 1.67 – 1.49 (m, 8H), 1.33 (h, *J* = 7.5 Hz, 8H), 0.95 (t, *J* = 7.5 Hz, 12H); **^{13}C NMR** (101 MHz, CD_3CN) δ 154.0, 142.5, 134.8, 131.2, 122.5, 118.8, 117.6, 59.3 (t, *J* = 3.0 Hz), 24.3, 20.3 (t, *J* = 1.5 Hz), 13.8.

1-(3,5-bis(trifluoromethyl)phenyl)-3-phenylurea·tetrabutylammonium azide (1c·N_3·Bu_4N)

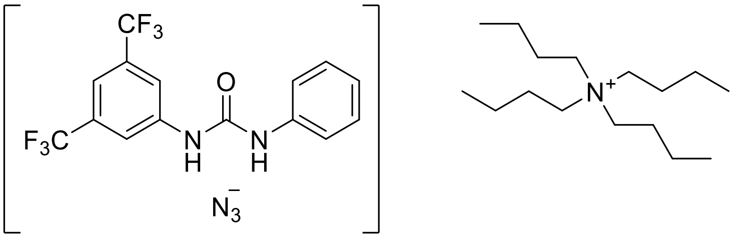

Prepared according to GP1 with 1-(3,5-bis(trifluoromethyl)phenyl)-3-phenylurea (174 mg, 0.50 mmol, to afford the title compound as a white solid (316 mg, 0.50 mmol, *quant*.). Single crystals of [**1c**·N_3]·TBA suitable for X-ray diffraction were grown by layering cyclohexane onto a saturated solution of the title compound in THF.

mp 86-88 °C; **ν_{max}** (neat) /cm^{-1} 2963, 2023, 1708, 1582, 1551, 1473, 1447, 1392, 1307, 1276, 1232, 1204, 1186, 1168, 1152, 1123, 1084, 1024, 877, 755, 681; **^{1}H NMR** (400 MHz, CD_3CN) δ 9.82 (s, 1H), 9.04 (s, 1H), 8.15 – 8.05 (m, 2H), 7.55 – 7.48 (m, 3H), 7.36 – 7.23 (m, 2H), 7.08 – 6.95 (m, 1H),

3.12 – 3.01 (m, 8H), 1.66 – 1.52 (m, 8H), 1.33 (h, *J* = 7.5 Hz, 8H), 0.95 (t, *J* = 7.5 Hz, 12H); **^{19}F NMR** (377 MHz, CD_3CN) -63.58; **^{13}C NMR** (151 MHz, CD_3CN) δ 154.1, 143.3, 140.5, 132.4 (q, *J* = 33.0 Hz), 129.9, 124.7 (q, *J* = 272.0 Hz), 123.5, 119.7, 118.9 (q, *J* = 3.5 Hz), 115.4 (p, *J* = 4.0 Hz), 59.4 (t, *J* = 3.0 Hz), 24.3, 20.3, 13.8.

1,3-bis(4-trifluoromethylphenyl)urea·tetrabutylammonium azide (1d·N_3·Bu_4N)

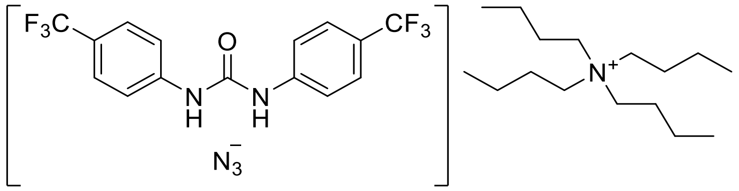

Prepared according to GP1 with 1,3-bis(4-trifluoromethyl)urea (174 mg, 0.50 mmol, 1.00 equiv) to afford the title compound as an off-white solid (321 mg, 0.49 mmol, 99%). Single crystals of [**1d**·N_3]·TBA suitable for X-ray diffraction were grown by slow diffusion of PhMe into a saturated solution of the title compound in MeCN.

mp 99-101 °C; **ν_{max}** (neat) /cm^{-1} 2963, 2006, 1711, 1603, 858; **^{1}H NMR** (400 MHz, Acetone-*d*$_6$) δ 10.11 (s, 2H), 7.78 (d, *J* = 8.5 Hz, 4H), 7.60 (d, *J* = 8.5 Hz, 4H), 3.53 – 3.31 (m, 8H), 1.86 – 1.75 (m, 8H), 1.41 (h, *J* = 7.5 Hz, 8H), 0.96 (t, *J* = 7.5 Hz, 12H); **^{19}F NMR** (376 MHz, Acetone-*d*$_6$) δ -62.06; **^{13}C NMR** (101 MHz, Acetone-*d*$_6$) δ 153.8, 144.9, 126.8 (q, *J* = 4.0 Hz), 125.7 (q, *J* = 270.0 Hz), 123.5 (q, *J* = 32.0 Hz), 118.7, 59.4 (t, *J* = 3.0 Hz), 24.5, 20.4 (t, *J* = 1.5 Hz), 13.8.

1,3-diphenylurea·tetrabutylammonium azide (1e·N_3·Bu_4N)

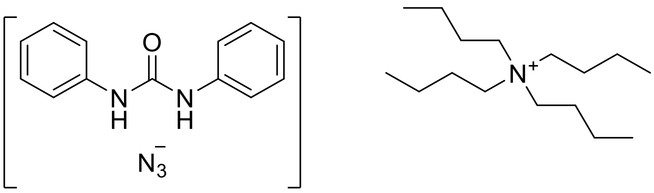

Prepared according to GP1 with 1,3-diphenylurea (106 mg, 0.50 mmol, 1 equiv) to afford the title compound as a white solid (248 mg, 0.50 mmol, *quant.*). Single crystals of [**1e**·N_3]·TBA suitable for X-ray diffraction were grown by layering cyclohexane onto a saturated solution of the title compound in EtOAc.

mp 69-71 °C; **ν_{max}** (neat) /cm^{-1} 3279, 2962, 2022 ,1706, 1594, 1545, 1488, 1445, 1384, 1297, 1201, 1173, 892, 754, 696; **^{1}H NMR** (400 MHz, Acetone-*d*$_6$) δ 9.41 (s, 2H), 7.67 – 7.53 (m, 4H), 7.31 – 7.15 (m, 4H), 6.90 (tt, *J* = 7.5, 1.0 Hz, 2H), 3.49 – 3.35 (m, 8H), 1.80 (p, *J* = 8.0 Hz, 8H), 1.41 (h, *J* = 7.5 Hz, 8H), 0.96 (t, *J* = 7.5 Hz, 12H); **^{13}C NMR** (101 MHz, Acetone-*d*$_6$) δ 141.8, 129.4, 122.0, 118.9, 118.8, 59.3, 30.4, 24.4, 20.4, 13.9.

1,3-bis(4-bromophenyl)urea·tetrabutylammonium azide (1f·N_3·Bu_4N)

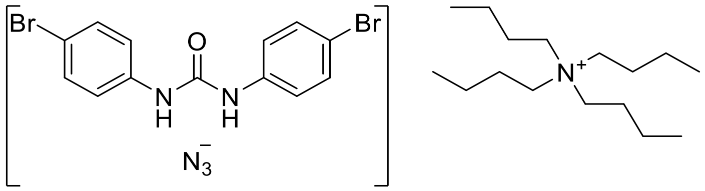

Prepared according to GP1 with 1,3-bis(4-bromophenyl)urea (185 mg, 0.50 mmol, 1.00 equiv) to afford the title compound as an off-white solid (326 mg, 0.50 mmol, *quant.*). Single crystals of [**1f**·N_3]·TBA suitable for X-ray diffraction were grown by layering cyclohexane onto a saturated solution of the title compound in THF.

mp 118-120 °C; **ν_{max}** (neat) /cm^{-1} 2961, 2020, 1704, 1485, 1174, 817; **^{1}H NMR** (400 MHz, DMSO-d_6) δ 8.95 (s, 2H), 7.64 – 7.29 (m, 8H), 3.21 – 3.00 (m, 8H), 1.56 (td, *J* = 11.5, 10.0, 6.0 Hz, 8H), 1.30 (h, *J* = 7.5 Hz, 8H), 0.93 (t, *J* = 7.5 Hz, 12H); **^{13}C NMR** (101 MHz, DMSO-d_6) δ 152.3, 139.0, 131.5, 120.2, 113.3, 57.5 (t, *J* = 3.0 Hz), 23.1, 19.2 (t, *J* = 3.0 Hz), 13.5.

1,3-bis(4-fluorophenyl)urea·tetrabutylammonium azide (1g·N_3·Bu_4N)

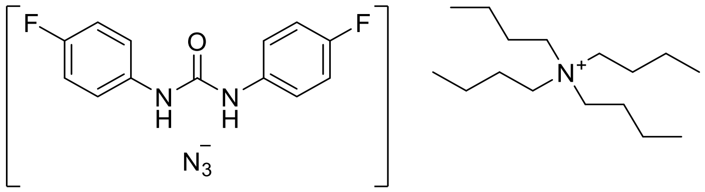

Prepared according to GP1 with 1,3-bis(4-fluorophenyl)urea (248 mg, 1.00 mmol, 1.00 equiv) to afford the title compound as an off-white solid (504 mg, 0.95 mmol, 95%). Single crystals of [**1g**·N_3]·TBA suitable for X-ray diffraction were grown by layering cyclohexane onto a saturated solution of the title compound in EtOAc.

mp 88-90 °C; **ν_{max}** (neat) /cm^{-1} 2961, 2021, 1705, 1613, 1561, 1503, 1305, 1200, 737; **^{1}H NMR** (400 MHz, CD_3CN) δ 8.68 (s, 2H), 7.51 – 7.42 (m, 4H), 7.08 – 6.97 (m, 4H), 3.12 – 3.02 (m, 8H), 1.59 (ddd, *J* = 12.0, 10.0, 6.5 Hz, 8H), 1.34 (h, *J* = 7.5 Hz, 8H), 0.96 (t, *J* = 7.5 Hz, 12H); **^{19}F NMR** (376 MHz, CD_3CN) δ -123.40; **^{13}C NMR** (101 MHz, CD_3CN) δ 159.0 (d, *J* = 238.0 Hz), 154.4, 137.4 (d, *J* = 2.0 Hz), 121.2 (d, *J* = 7.5 Hz), 116.1 (d, *J* = 22.5 Hz), 59.3 (t, *J* = 3.0 Hz), 24.3, 20.3 (t, *J* = 1.5 Hz), 13.8.

1,3-bis(4-cyanophenyl)urea·tetrabutylammonium azide (1h·N_3·Bu_4N)

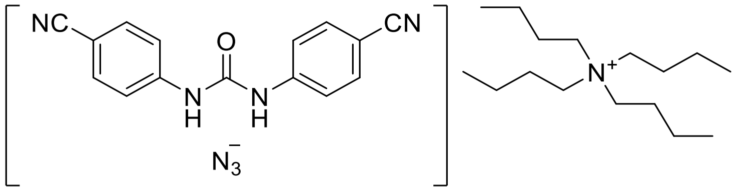

Prepared according to GP1 with 1,3-bis(4-cyanophenyl)urea (131 mg, 0.50 mmol, 1.00 equiv) to afford the title compound as an off-white solid (272 mg, 0.50 mmol, *quant.*). Single crystals of

{[**1h**]$_2$·N_3·2H_2O}·TBA suitable for X-ray diffraction were grown by layering cyclohexane onto a saturated solution of the title compound in THF.

mp 96-98 °C; **ν$_{max}$** (neat) /cm^{-1} 2962, 2222, 2014, 1719, 1590, 1170; **^{1}H NMR** (400 MHz, Acetone-*d*$_6$) δ 10.33 (s, 2H), 7.78 – 7.71 (m, 4H), 7.71 – 7.62 (m, 4H), 3.50 – 3.32 (m, 8H), 1.99 – 1.70 (m, 8H), 1.42 (h, *J* = 7.5 Hz, 8H), 0.96 (t, *J* = 7.5 Hz, 8H); **^{13}C NMR** (101 MHz, Acetone-*d*$_6$) δ 152.6, 144.5, 133.0, 119.0, 118.2, 104.2, 58.5 (t, *J* = 3.0 Hz), 23.5, 19.5 (t, *J* = 1.5 Hz), 13.0.

diphenylguanidine·tetrabutylammonium azide (1i·N$_3$·Bu$_4$N)

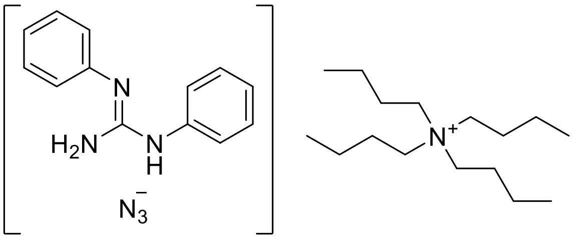

Prepared according to GP1 with diphenylguanidine (106 mg, 0.50 mmol, 1.00 equiv) to afford the title compound as a white solid (248 mg, 0.50 mmol, *quant.*). Single crystals of [**1i**·N_3]·TBA suitable for X-ray diffraction were grown by layering cyclohexane onto a saturated solution of the title compound in acetone.

mp 93-95 °C; **ν$_{max}$** (neat) /cm^{-1} 2960, 2011, 1665, 1554, 700; **^{1}H NMR** (400 MHz, $CDCl_3$) δ 7.26 – 7.20 (m, 8H), 6.98 – 6.90 (m, 2H), 3.30 – 3.14 (m, 8H), 1.58 (dq, *J* = 12.0, 8.0 Hz, 8H), 1.40 (h, *J* = 7.5 Hz, 8H), 0.98 (t, *J* = 7.5 Hz, 12H); **^{13}C NMR** (101 MHz, $CDCl_3$) δ 149.1, 145.3, 129.2, 122.0, 121.8, 58.6, 23.9, 19.8, 13.8.

***N*^{1},*N*^{2}-bis(3,5-bis(trifluoromethyl)phenyl)oxalamide·tetrabutylammonium azide (1j·N$_3$·Bu$_4$N)**

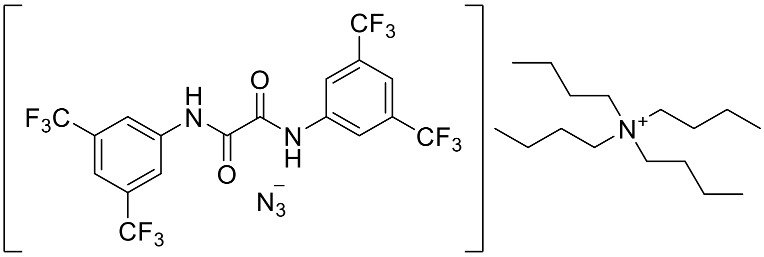

Prepared according to GP1 with *N*^{1},*N*^{2}-bis(3,5-bis(trifluoromethyl)phenyl)oxalamide (256 mg, 0.50 mmol, 1.00 equiv) to afford the title compound as an off-white solid (398 mg, 0.50 mmol, *quant.*). Single crystals of [**1j**·N_3]·TBA suitable for X-ray diffraction were grown by layering cyclohexane onto a saturated solution of the title compound in THF.

mp 140-142 °C; **ν$_{max}$** (neat) /cm^{-1} 2964, 2033, 1683, 1537, 1373, 1129, 890; **^{1}H NMR** (500 MHz, Acetone-*d*$_6$) δ 8.66 (s, 4H), 7.81 (s, 2H), 4.20 – 2.96 (m, 8H), 1.82 (dq, *J* = 12.0, 7.5 Hz, 8H), 1.43 (h, *J* = 7.5 Hz, 8H), 0.97 (t, *J* = 7.5 Hz, 12H); **^{19}F NMR** (470 MHz, Acetone-*d*$_6$) δ -63.58; **^{13}C NMR** (126

MHz, Acetone-d_6) δ 159.6, 141.0, 132.6 (q, *J* = 33.0 Hz), 124.4 (q, *J* = 272.0 Hz), 121.8 – 121.1 (m), 119.0 – 117.8 (m), 59.3 (t, *J* = 3.0 Hz), 24.4, 20.4 (t, *J* = 1.5 Hz), 13.9.

(±)-3-(3,5-bis(trifluoromethyl)phenyl)-1-(2'-(3-(3,5-bis(trifluoromethyl)phenyl)ureido)-[1,1'-binaphthalen]-2-yl)-1-isopropylurea·tetrabutylammonium azide ((±)-1k·N_3·Bu_4N)

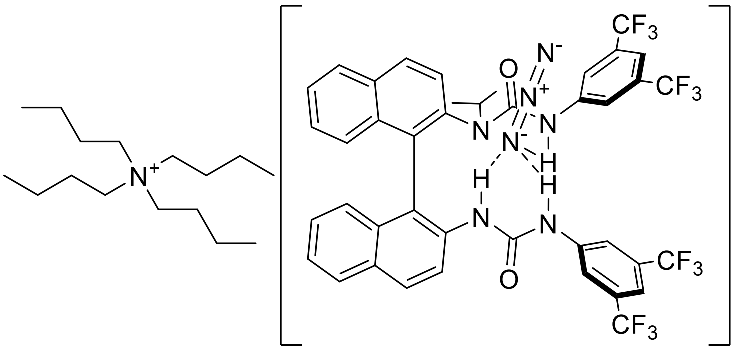

MeCN (2.5 mL) was added to (±)-**1k** (209 mg, 0.25 mmol, 1 equiv) and tetrabutylammonium azide (71.2 mg, 0.25 mmol, 1 equiv). The reaction mixture was stirred at rt for 24 h. The reaction mixture was evaporated to dryness to afford the title compound as a white solid (277 mg, 0.25 mmol, 99%).

Single crystals of [(±)-**1k**·N_3]·TBA suitable for X-ray diffraction were grown by slow evaporation of a saturated solution of the title compound in hot hexane with a minimal quantity of EtOAc.

mp 85-87 °C; **ν_{max}** (neat) /cm^{-1} 2034, 1709, 1666, 1388, 1277, 1175, 1121, 880, 747, 681; **^{1}H NMR** (500 MHz, $CDCl_3$) δ 9.27 (br s, 1H), 8.84 (d, *J* = 8.0 Hz, 1H), 8.28 (br s, 1H), 8.03 – 7.80 (m, 7H), 7.51 – 7.40 (m, 4H), 7.32 (t, *J* = 7.5 Hz, 1H), 7.20 – 7.14 (m, 4H), 7.04 (s, 1H), 6.77 (d, *J* = 8.5 Hz, 1H), 3.12 – 3.02 (m, 9H), 1.57 – 1.51 (m, 8H), 1.38 – 1.30 (m, 8H), 1.20 (d, *J* = 6.5 Hz, 3H), 0.95 (t, *J* = 7.5 Hz, 12H), 0.36 (d, *J* = 6.5 Hz, 3H); **^{19}F NMR** (471 MHz, $CDCl_3$) δ -62.82, -63.02; **^{13}C NMR** (126 MHz, $CDCl_3$) δ 153.9, 153.0, 141.6, 141.0, 140.6, 136.8, 134.8, 133.4×2, 131.9, 131.6, 131.4 (q, *J* = 33.0 Hz), 131.1 (q, *J* = 33.0 Hz), 130.0, 129.1, 128.7, 128.2, 127.4, 127.1, 126.7, 126.5, 126.4, 125.3, 124.0, 123.4 (q, *J* = 272.5 Hz), 123.4 (q, *J* = 273.0 Hz), 120.8 (q, *J* = 5.0 Hz), 120.3, 119.7, 117.4 (q, *J* = 5.0 Hz), 114.8, 114.5, 58.9, 56.4, 23.9, 20.0, 19.7, 19.1, 13.6.

Reactivity study of selected achiral urea catalysts and comparison with their corresponding $K_{a(1:1)}$ binding constants

Screening according to general procedure 2 (GP2)

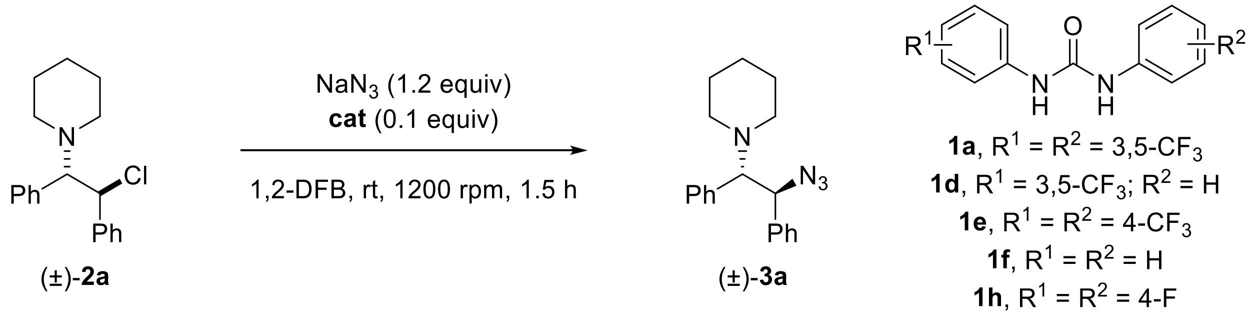

entry	cat	$K_{a(1:1)}$ (M^{-1})	NMR yield
1	**1a**, $R^1 = R^2$ = 3,5-CF_3	$1.57 \pm 0.06 \times 10^3$	90%
2	**1d**, R^1 = 3,5-CF_3; R^2 = H	$9.4 \pm 1.7 \times 10^2$	64%
3	**1e**, $R^1 = R^2$ = 4-CF_3	$1.25 \pm 0.12 \times 10^3$	40%
4	**1f**, $R^1 = R^2$ = H	$3.14 \pm 0.03 \times 10^2$	11%
5	**1h**, $R^1 = R^2$ = 4-F	$4.82 \pm 0.05 \times 10^2$	2%

NMRy determined with Ph_3CH internal standard. 1,2-DFB = 1,2-difluorobenzene.

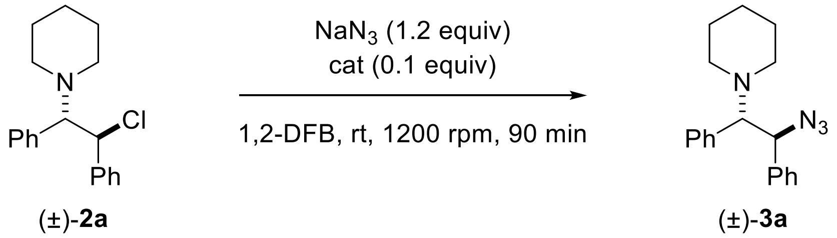

entry	cat	NMR yield
1	**1a**, $R^1 = R^2$ = 3,5-CF_3	90%
2	**1b**, $R^1 = R^2$ = 3-Cl	12%
3	**1c**, R^1 = 3,5-CF_3; R^2 = H	64%
4	**1d**, $R^1 = R^2$ = 4-CF_3	40%
5	**1e**, $R^1 = R^2$ = H	11%
6	**1f**, $R^1 = R^2$ = 4-Br	3%
7	**1g**, $R^1 = R^2$ = 4-F	2%
8	**1h**, $R^1 = R^2$ = 4-CN	16%
10	**1i**	37%
11	**1j**	10%

NMRy determined with Ph_3CH internal standard. 1,2-DFB = 1,2-difluorobenzene.

(*S*)-1k ^{1}H assignment ($CDCl_3$)

Full characterisation of (*S*)-**1k** with ^{1}H, ^{19}F, and ^{13}C spectra in $CDCl_3$ has been reported.[8] Full assignment of (*S*)-**1k** in CD_2Cl_2 has been reported.[10] The ^{1}H assignment of (*S*)-**1k** in $CDCl_3$ was assisted with 2D ^{1}H-^{1}H COSY, ^{1}H-^{1}H NOESY, ^{1}H-1^{5}N HSQC, and ^{1}H-^{15}N HMBC spectra, and is in good agreement with the ^{1}H assignment of (*S*)-**1k** in CD_2Cl_2.

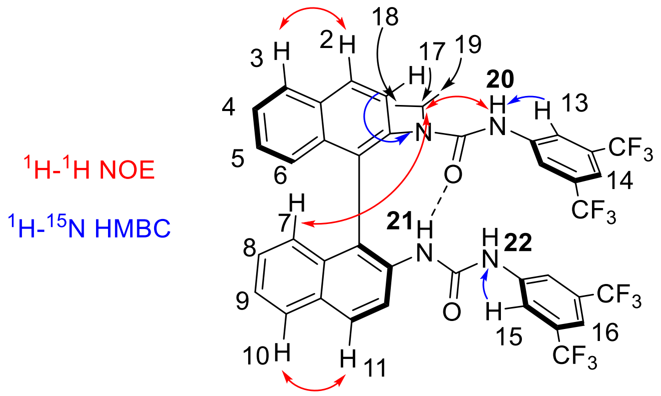

^{1}H NMR (500 MHz, $CDCl_3$) δ 8.54 (d, *J* = 9.0 Hz, 1H, **H12**), 8.14 (d, *J* = 9.0 Hz, 1H, **H2**), 8.02 (d, *J* = 9.0 Hz, 1H, **H11**), 7.99 (d, *J* = 8.0 Hz, 1H, **H3**), 7.92 (d, *J* = 8.0 Hz, 1H, **H10**), 7.82 (s, 2H, **H13**), 7.61 (s, 2H, **H15**), 7.56-7.51 (m, 4 H, **H4, H1, H14, H21**), 7.43 – 7.38 (m, 2H, **H9, H16**), 7.30 (ddd, *J* = 8.0, 6.5, 1.0 Hz, 1H, **H5**), 7.26 – 7.22 (m, 1H, **H8**), 7.17 (d, *J* = 8.5 Hz, 1H, **H6**), 7.06 (s, 1H, **H22**), 6.90 (d, *J* = 8.5 Hz, 1H, **H7**), 6.80 (s, 1H, **H20**), 3.66 (sept, *J* = 7.0 Hz, 1H, **H17**), 1.06 (d, *J* = 7.0 Hz, 3H, **H19**), 0.67 (d, *J* = 7.0 Hz, 3H, **H18**).

^{1}H NMR (500 MHz, $CDCl_3$)

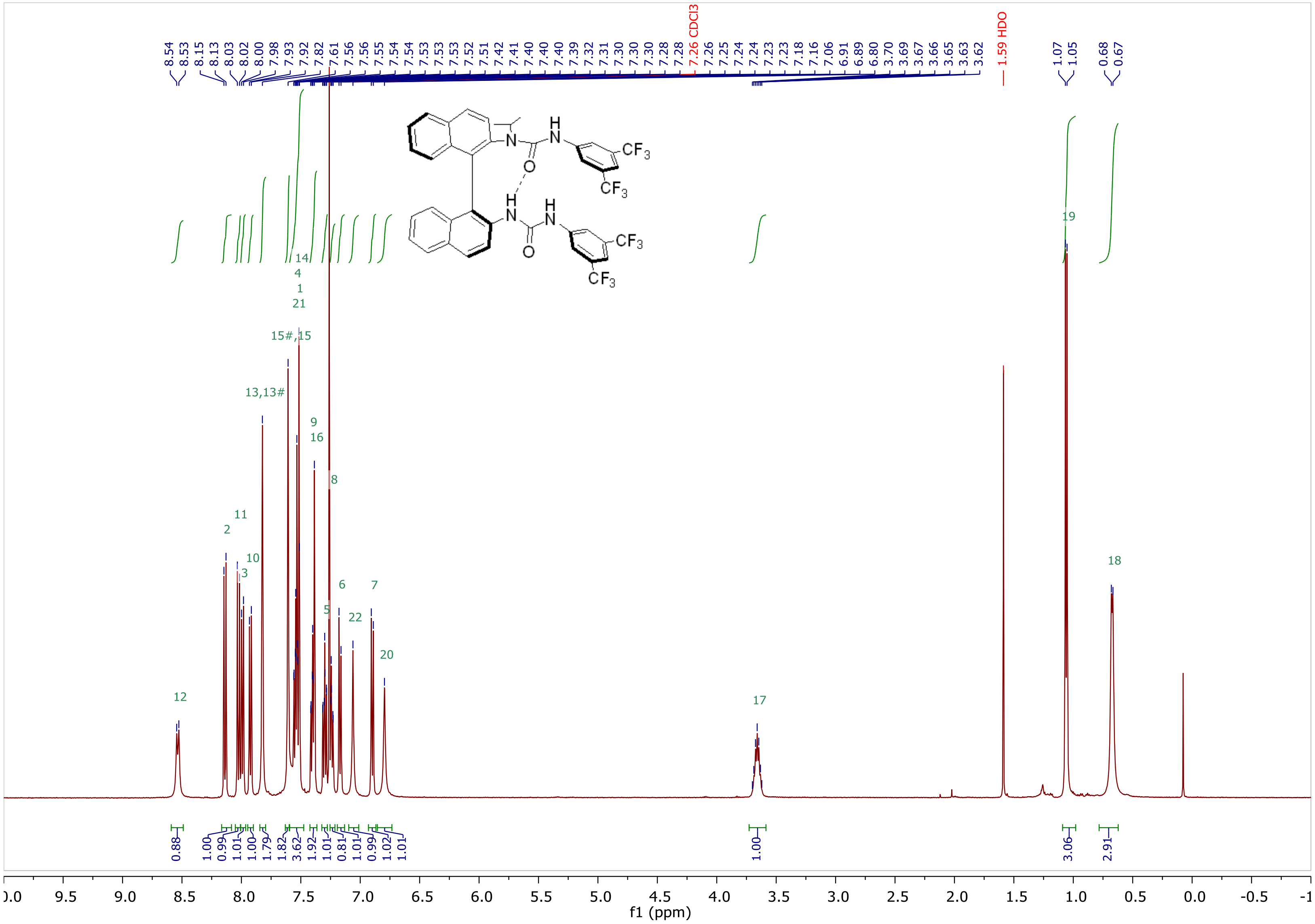

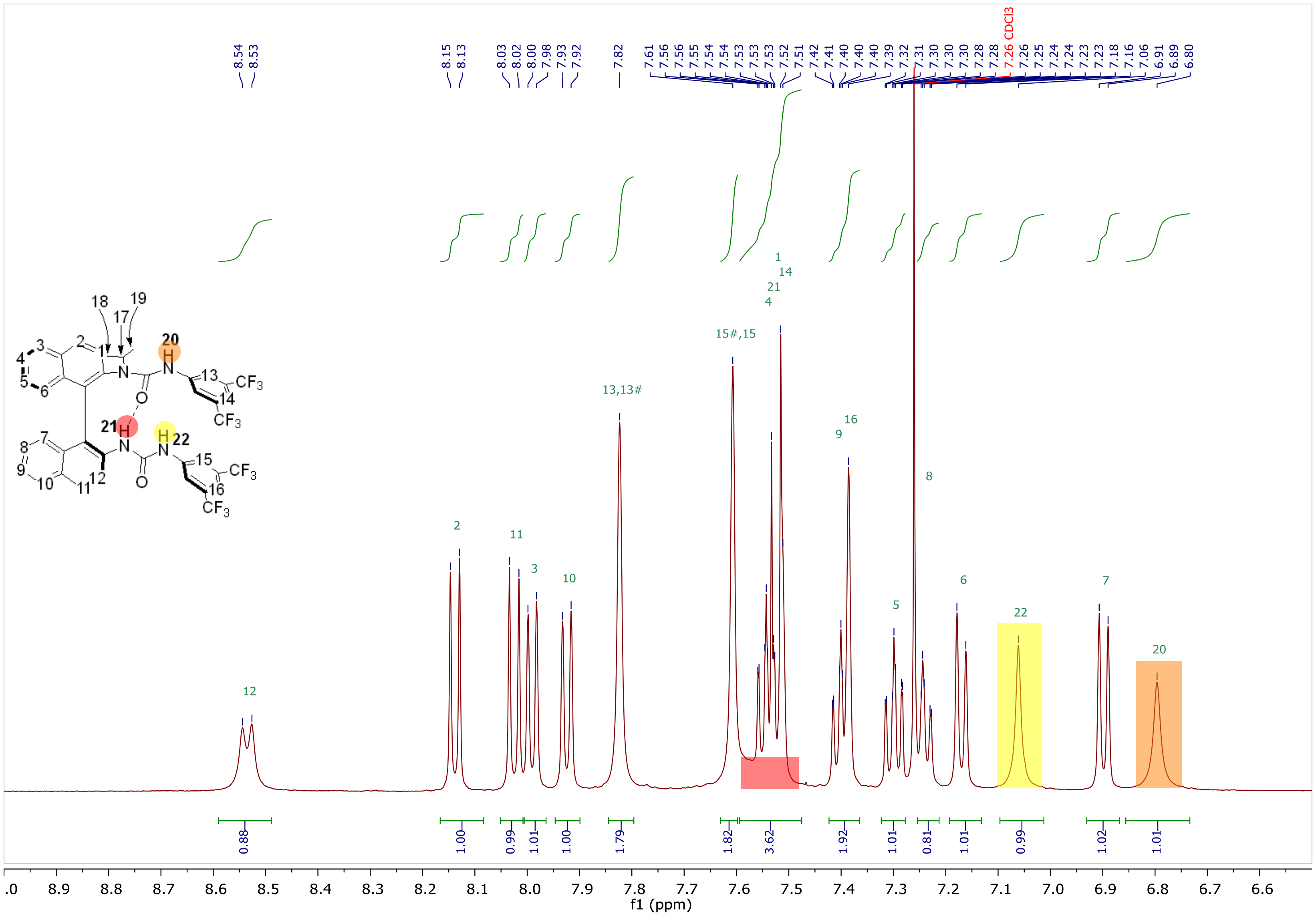
8.54
8.53
8.15
8.13
8.03
8.02
8.00
7.98
7.93
7.92
7.82
7.26 CDCl3
12
2
11
3
10
13,13#
15#,15
1
14
21
4
16
9
8
5
6
22
7
20
0.88
1.00
0.99
1.01
1.00
1.79
1.82
3.62
1.92
1.01
0.81
1.01
0.99
1.02
1.01
f1 (ppm)

^{1}H-^{1}H COSY (500 MHz, $CDCl_3$)

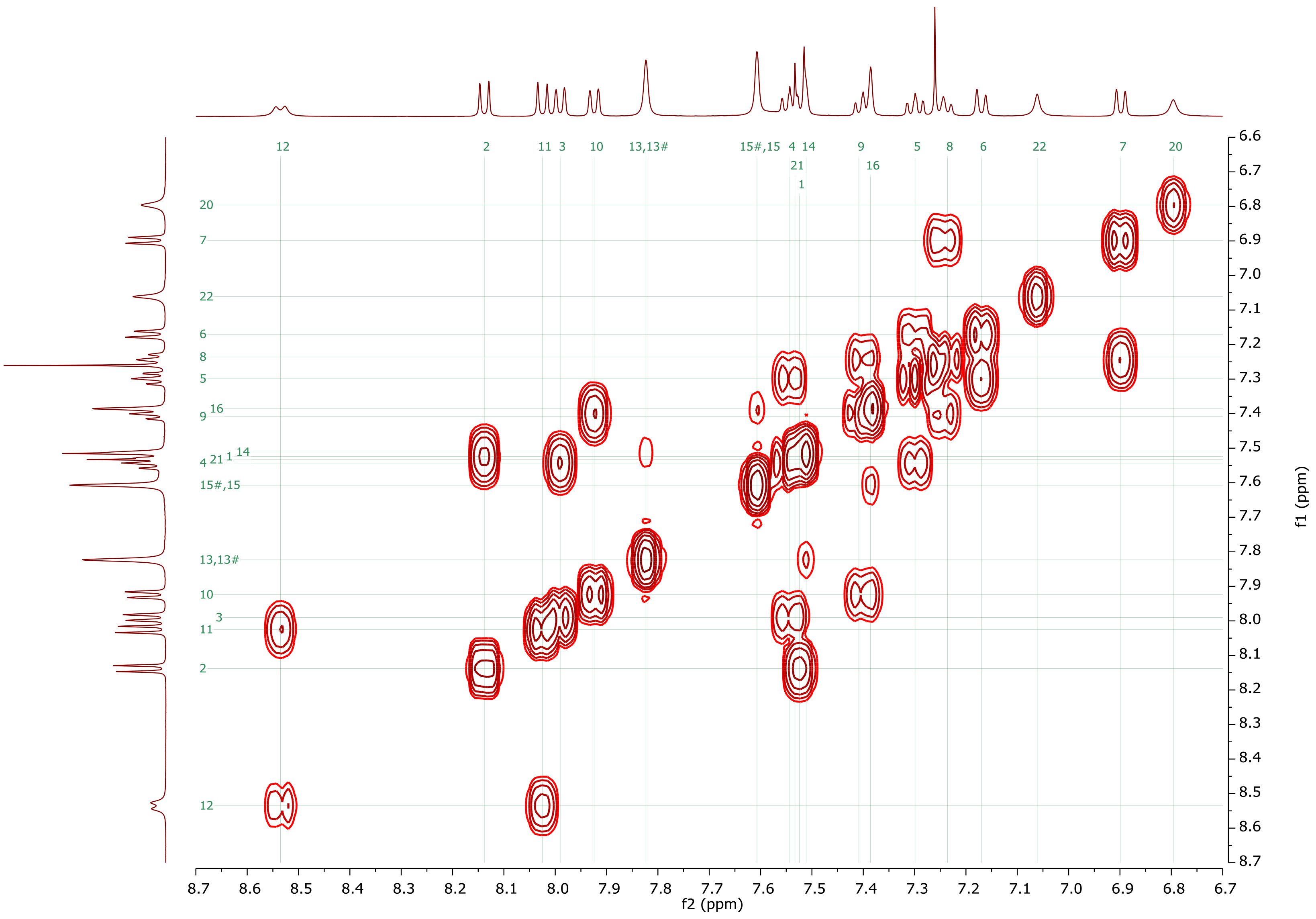

^{1}H-^{1}H NOESY (500 MHz, $CDCl_3$)

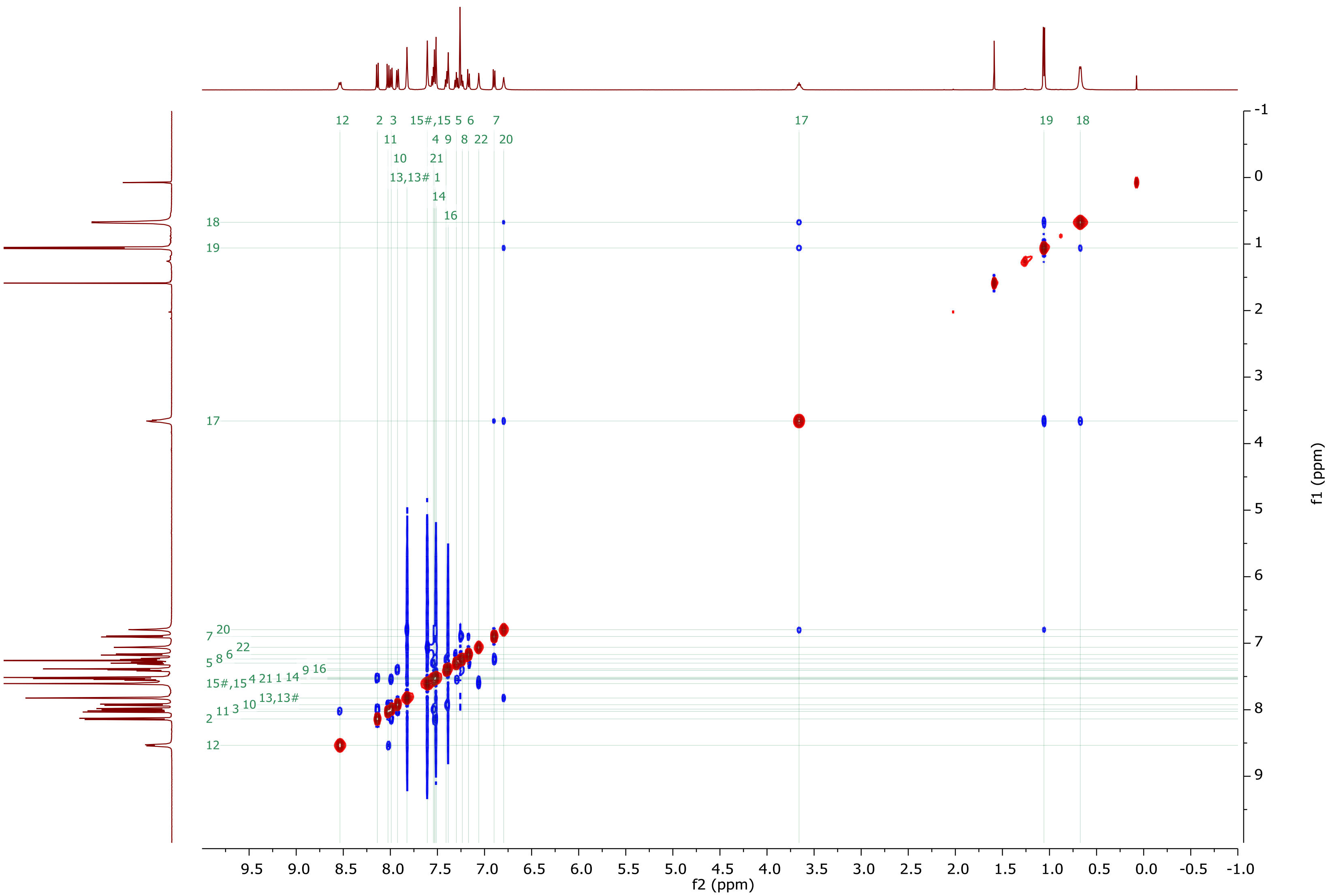

Expansion of above ^1H-^1H NOESY

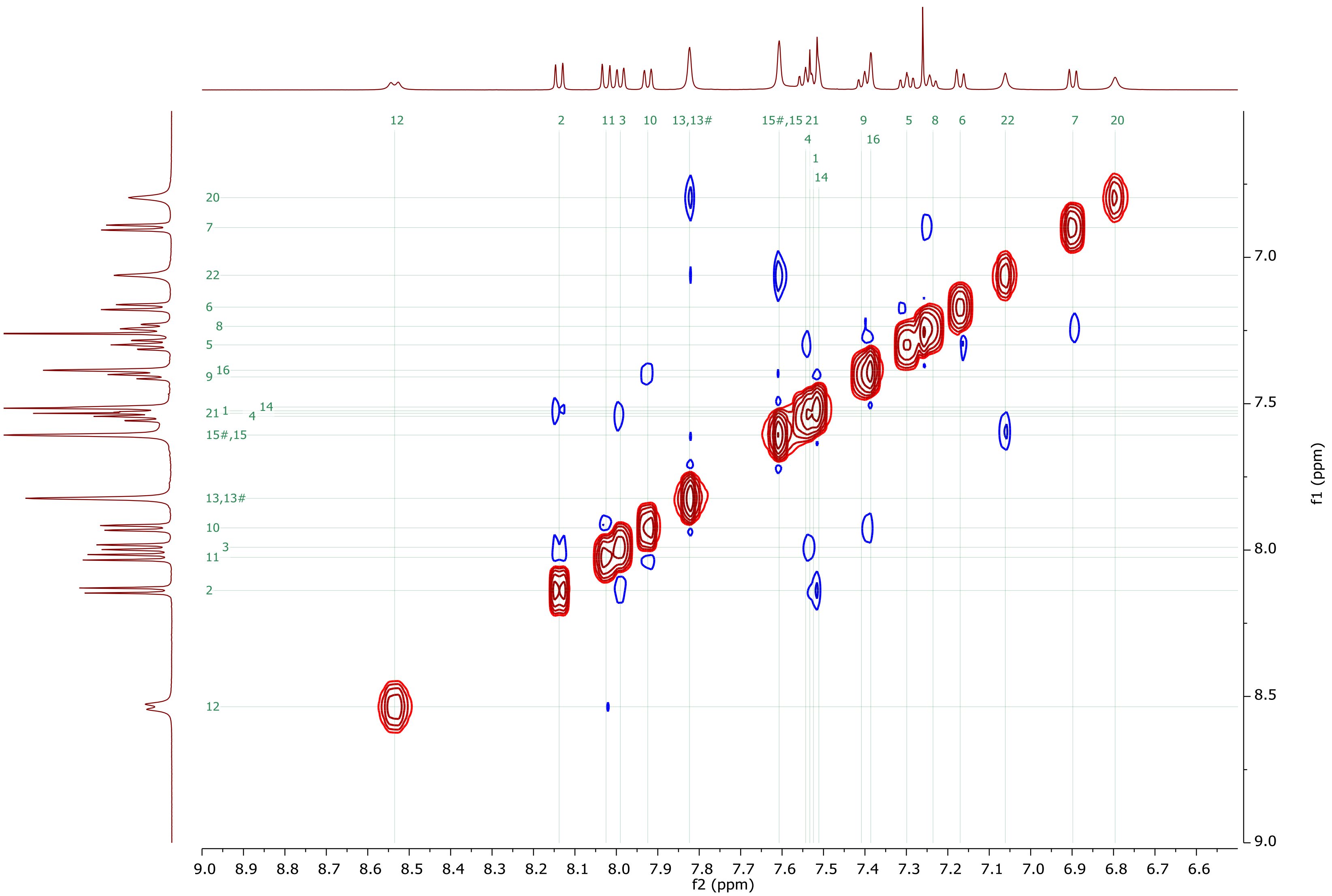

Superimposed ^{1}H-^{15}N HSQC (red) and ^{1}H-^{15}N HMBC (green) ($CDCl_3$)

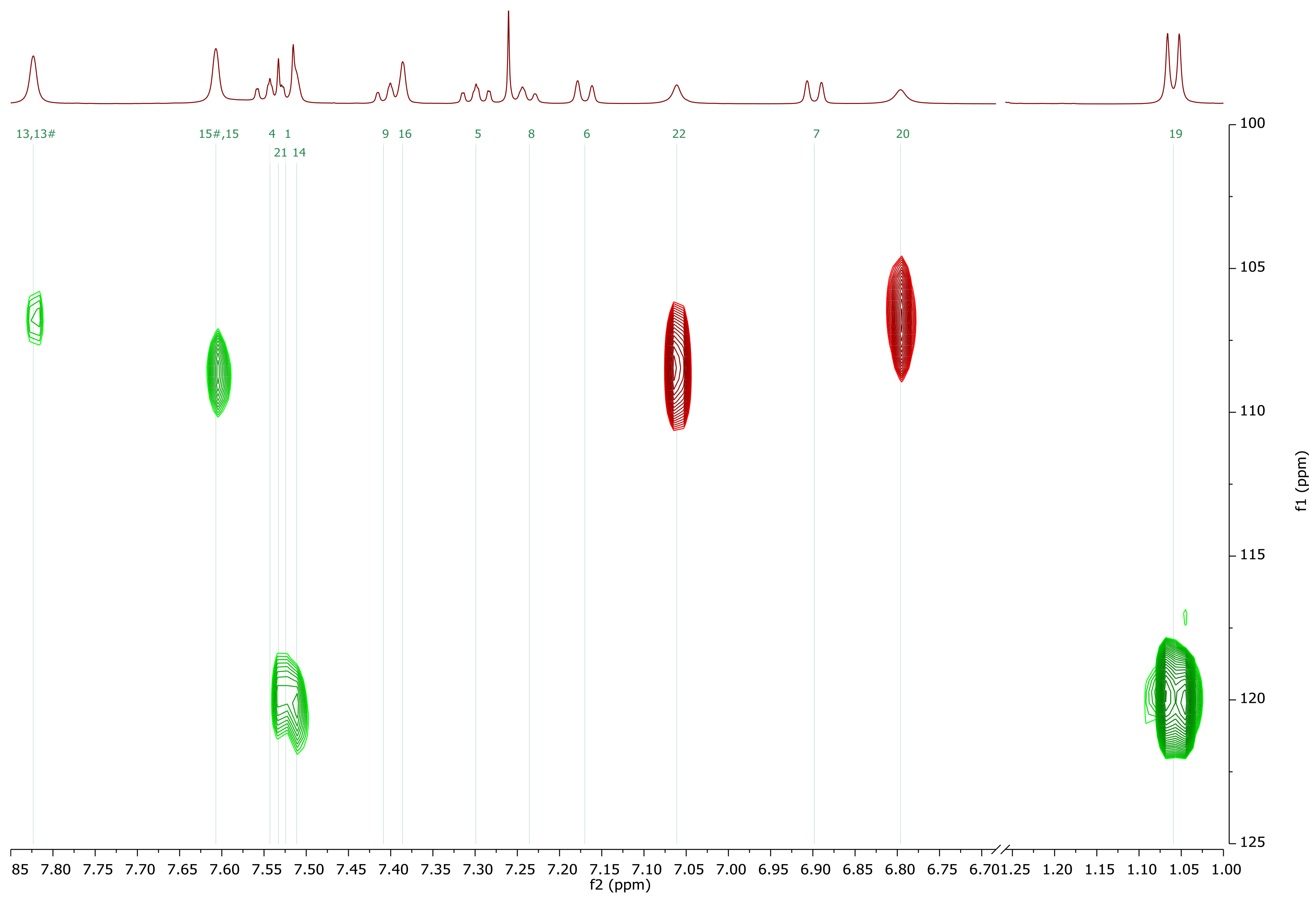

^{1}H NMR Titrations

Titration procedure

^{1}H NMR spectra were recorded on Bruker AVANCE III HD 500 MHz spectrometers at 298 K. A solution of host (0.5 mL, 2 mM) was placed in an NMR tube. A ^{1}H NMR spectrum was recorded followed by stepwise addition (0.0 equiv, 0.2 equiv, 0.4 equiv, 0.6 equiv, 0.8 equiv, 1.0 equiv, 1.2 equiv, 1.4 equiv, 1.6 equiv, 1.8 equiv, 2.0 equiv, 2.5 equiv, 3.0 equiv, 4.0 equiv, 5.0 equiv, 7.0 equiv, 10.0 equiv) of a solution of the selected salt (100 mM) using a 25 or 50 μL Hamilton Microlitre syringe. After each addition, the sample was shaken thoroughly.

Data analysis & model fitting

Data was analysed with BindFit v0.5,[11] enabling a global analysis approach to improve the quality of the resultant fits.[11,12] Errors in association constants are the standard deviation from three independent repetitions. Details of BindFit v0.5,[11] derivation of equations,[12] and descriptions of models implemented and respective equations are published.[13,14]

The appropriate binding model (1:1 or full 2:1 model) was determined by comparing the 'cov_{fit} factor' of the two models.[13] The cov_{fit} (covariance of the fit) was generated by BindFit v0.5,[11] and is calculated by dividing the covariance of the residual with the covariance of the experimental data. The 'cov_{fit} factor' was calculated by dividing the cov_{fit} from the to 1:1 binding model with the cov_{fit} from the model under study.[13]

1a and tetrabutylammonium azide

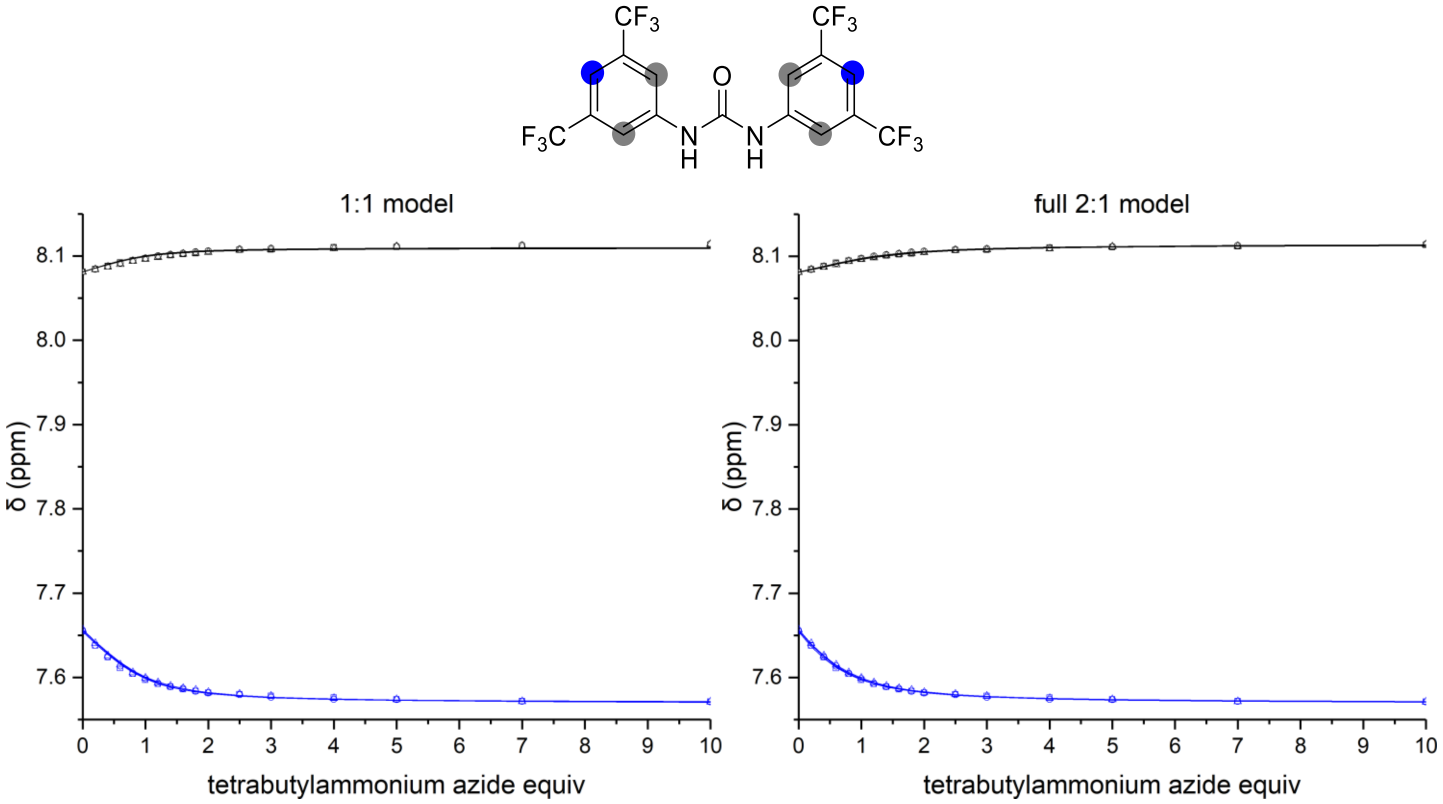

Figure S1 ^{1}H NMR titration data of **1a** with tetrabutylammonium azide. One set of symbols (□, ○, △) refers to experimental data from one set of measurements. Lines are the calculated isotherms of the described model. (2 mM **1a**, CH_3CN/CD_3CN 8:2, 500 MHz, 298 K).

Table S1 Comparison of binding models

model	entry	cov_{fit} (10^{-3})	cov_{fit} factor	$K_{a(1:1)}$ (M^{-1})	$K_{a(2:1)}$ (M^{-1})	$\Delta G_{(1:1)}$ (kJ mol^{-1})	$\Delta G_{(2:1)}$ (kJ mol^{-1})
1:1	1	2.26	1	2.86×10^3	-		
	2	1.81	1	2.68×10^3	-		
	3	2.04	1	2.45×10^3	-		
	**mean**	**2.04**	**1**	**$2.66 \pm 0.18 \times 10^3$**	**-**	**-19.54 ± 0.17**	**-**
**full 2:1**	1	0.14	16.1	1.55×10^3	1.06×10^2		
	2	0.062	29.2	1.65×10^3	6.62×10^1		
	3	0.083	24.6	1.50×10^3	3.23×10^1		
	**mean**	**0.095**	**23.3**	**$1.57 \pm 0.06 \times 10^3$**	**$7 \pm 3 \times 10^1$**	**-18.23 ± 0.09**	**-10.4 ± 1.1**

cov_{fit} factor is cov_{fit} for the 1:1 model divided by the cov_{fit} for the binding model under study.[13] Values calculated with BindFit v0.5,[11] error is the standard deviation from 3 independent replicas.

The mean cov_{fit} factor for the full 2:1 model is significantly higher than the mean cov_{fit} for the 1:1 model, suggesting this system is best described by the full 2:1 model.

1c and tetrabutylammonium azide

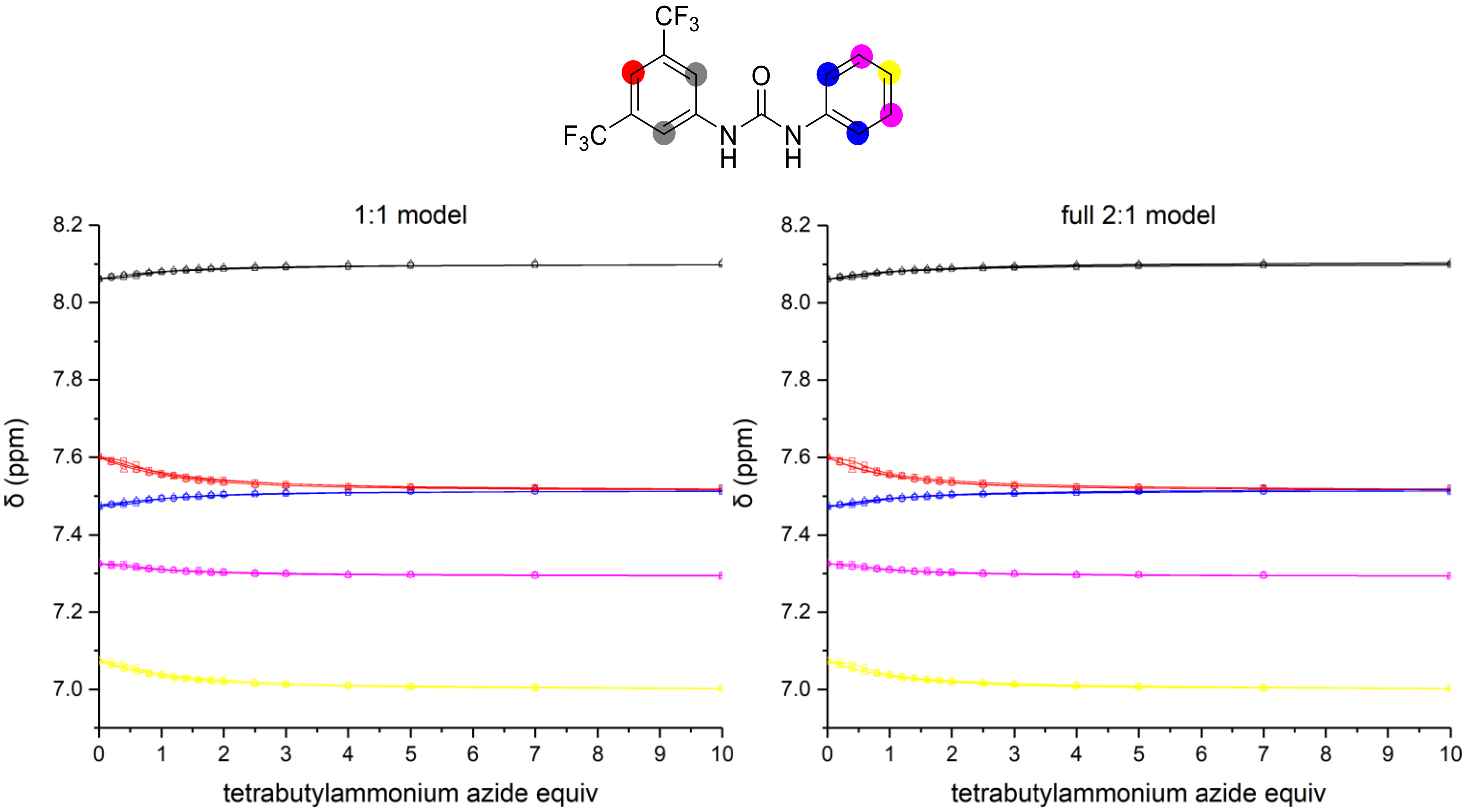

Figure S2 ^{1}H NMR titration data of **1c** with tetrabutylammonium azide. One set of symbols (□, ○, △) refers to experimental data from one set of measurements. Lines are the calculated isotherms of the described model. (2 mM **1c**, CH_3CN/CD_3CN 8:2, 500 MHz, 298 K).

Table S2 Comparison of binding models

model	entry	cov_{fit} (10^{-3})	cov_{fit} factor	$K_{a(1:1)}$ (M^{-1})	$K_{a(2:1)}$ (M^{-1})	$\Delta G_{(1:1)}$ (kJ mol^{-1})	$\Delta G_{(2:1)}$ (kJ mol^{-1})
**1:1**	1	6.43	1	6.93×10^2	-		
	2	1.11	1	1.06×10^3	-		
	3	1.62	1	1.06×10^3	-		
	**mean**	**3.05**	**1**	**$9.4 \pm 1.7 \times 10^2$**	**-**	**-17.0 ± 0.4**	**-**
full 2:1	1	1.54	4.2	4.57×10^5	2.37×10^5		
	2	0.74	1.5	2.77×10^2	-5.95×10^1		
	3	0.84	1.9	4.75×10^2	6.84×10^1		
	**mean**	**1.04**	**2.5**	**$2 \pm 2 \times 10^4$**	**$8 \pm 11 \times 10^3$**	**-24.5 ± 24.5**	**-16.6 ± 3.4**

cov_{fit} factor is cov_{fit} for the 1:1 model divided by the cov_{fit} for the binding model under study.[13] Values calculated with BindFit v0.5,[11] error is the standard deviation from 3 independent replicas.

The mean cov_{fit} factor for the full 2:1 model is not significantly higher than the mean cov_{fit} for the 1:1 model, suggesting this system is best described by the 1:1 model. In addition the $K_{a(2:1)}$ cannot be negative, hence it is likely the fits from the full 2:1 models do not need to be considered.

1d and tetrabutylammonium azide

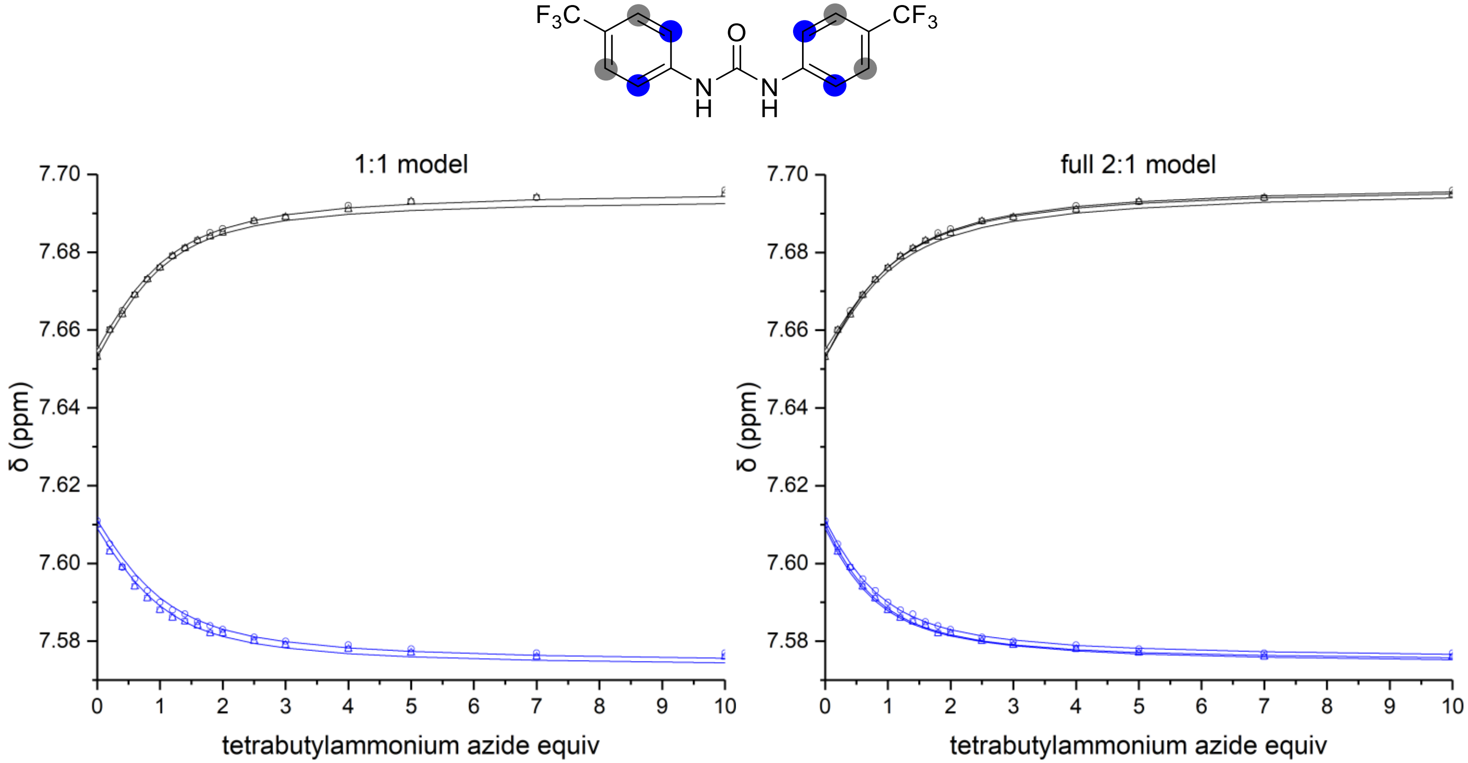

Figure S3 ^{1}H NMR titration data of **1d** with tetrabutylammonium azide. One set of symbols (□, ○, △) refers to experimental data from one set of measurements. Lines are the calculated isotherms of the described model. (2 mM **1d**, CH_3CN/CD_3CN 8:2, 500 MHz, 298 K)..

Table S3 Comparison of binding models

model	entry	cov_{fit} (10^{-3})	cov_{fit} factor	$K_{a(1:1)}$ (M^{-1})	$K_{a(2:1)}$ (M^{-1})	$\Delta G_{(1:1)}$ (kJ mol^{-1})	$\Delta G_{(2:1)}$ (kJ mol^{-1})
1:1	1	1.08	1	1.39×10^3	-		
	2	0.94	1	1.24×10^3	-		
	3	0.94	1	1.48×10^3	-		
	**mean**	**0.99**	**1**	**$1.4 \pm 0.1 \times 10^3$**	**-**	**-17.95 ± 0.18**	**-**
**full 2:1**	1	0.16	6.8	1.31×10^3	1.99×10^2		
	2	0.16	5.9	1.37×10^3	1.20×10^2		
	3	0.15	6.3	1.08×10^3	7.47×10^1		
	**mean**	**0.16**	**6.3**	**$1.25 \pm 0.12 \times 10^3$**	**$1.3 \pm 0.5 \times 10^2$**	**-17.7 ± 0.2**	**-12 ± 1**

cov_{fit} factor is cov_{fit} for the 1:1 model divided by the cov_{fit} for the binding model under study.[13] Values calculated with BindFit v0.5,[11] error is the standard deviation from 3 independent replicas.

The mean cov_{fit} factor for the full 2:1 model is significantly higher than the mean cov_{fit} for the 1:1 model, suggesting this system is best described by the full 2:1 model.

1e and tetrabutylammonium azide

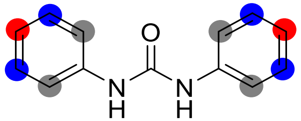

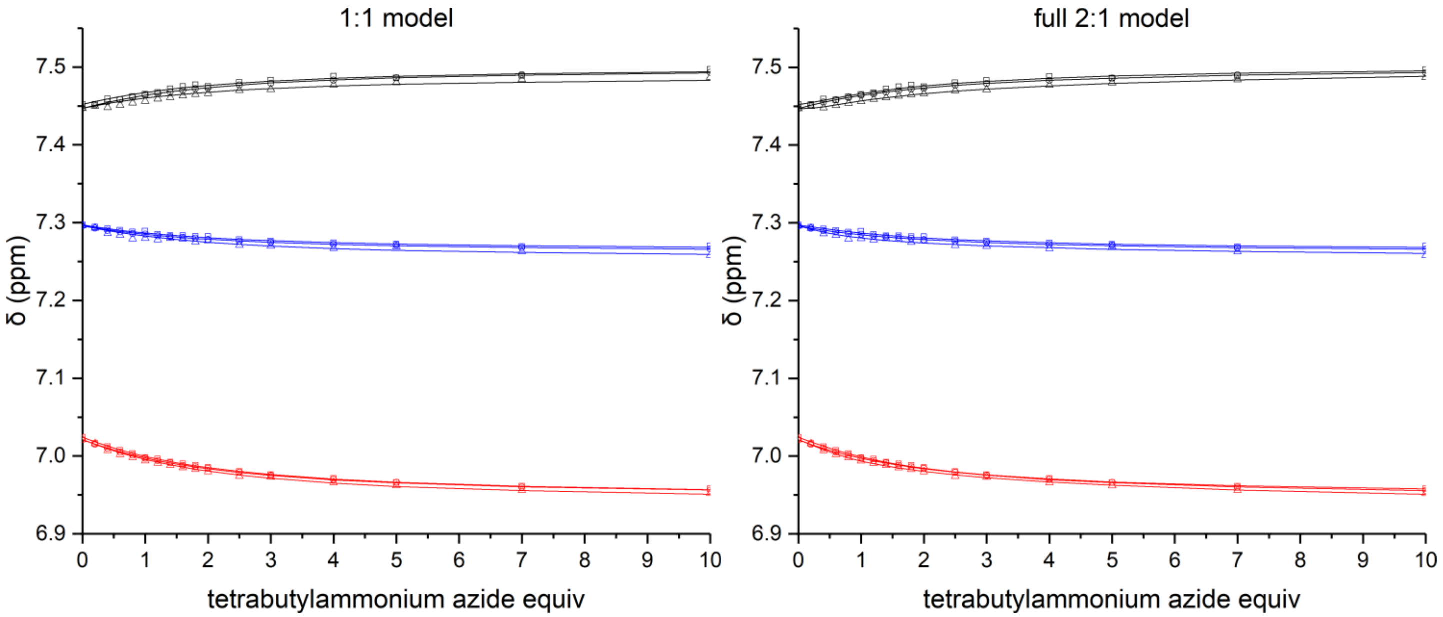

Figure S4 ^{1}H NMR titration data of **1e** with tetrabutylammonium azide. One set of symbols (□, ○, △) refers to experimental data from one set of measurements. Lines are the calculated isotherms of the described model. (2 mM **1e**, CH_3CN/CD_3CN 8:2, 500 MHz, 298 K).

Table S4 Comparison of binding models

model	entry	cov_{fit} (10^{-3})	cov_{fit} factor	$K_{a(1:1)}$ (M^{-1})	$K_{a(2:1)}$ (M^{-1})	$\Delta G_{(1:1)}$ (kJ mol^{-1})	$\Delta G_{(2:1)}$ (kJ mol^{-1})
**1:1**	1	2.95	1	3.14×10^2	-		
	2	0.18	1	3.17×10^2	-		
	3	7.09	1	3.10×10^2	-		
	**mean**	**3.41**	**1**	**$3.14 \pm 0.03 \times 10^2$**	**-**	**-14.3 ± 0.2**	**-**
full 2:1	1	2.45	1.2	6.67×10^2	2.88×10^2		
	2	0.093	1.9	3.64×10^2	1.51×10^2		
	3	2.51	2.8	8.38×10^2	1.65×10^3		
	**mean**	**1.68**	**2.0**	**$6 \pm 2 \times 10^2$**	**$7 \pm 7 \times 10^2$**	**-15.8 ± 0.8**	**-11 ± 2**

cov_{fit} factor is cov_{fit} for the 1:1 model divided by the cov_{fit} for the binding model under study.[13] Values calculated with BindFit v0.5,[11] error is the standard deviation from 3 independent replicas.

The mean cov_{fit} factor for the full 2:1 model is not significantly higher than the mean cov_{fit} for the 1:1 model, suggesting this system is best described by the 1:1 model.

1g and tetrabutylammonium azide

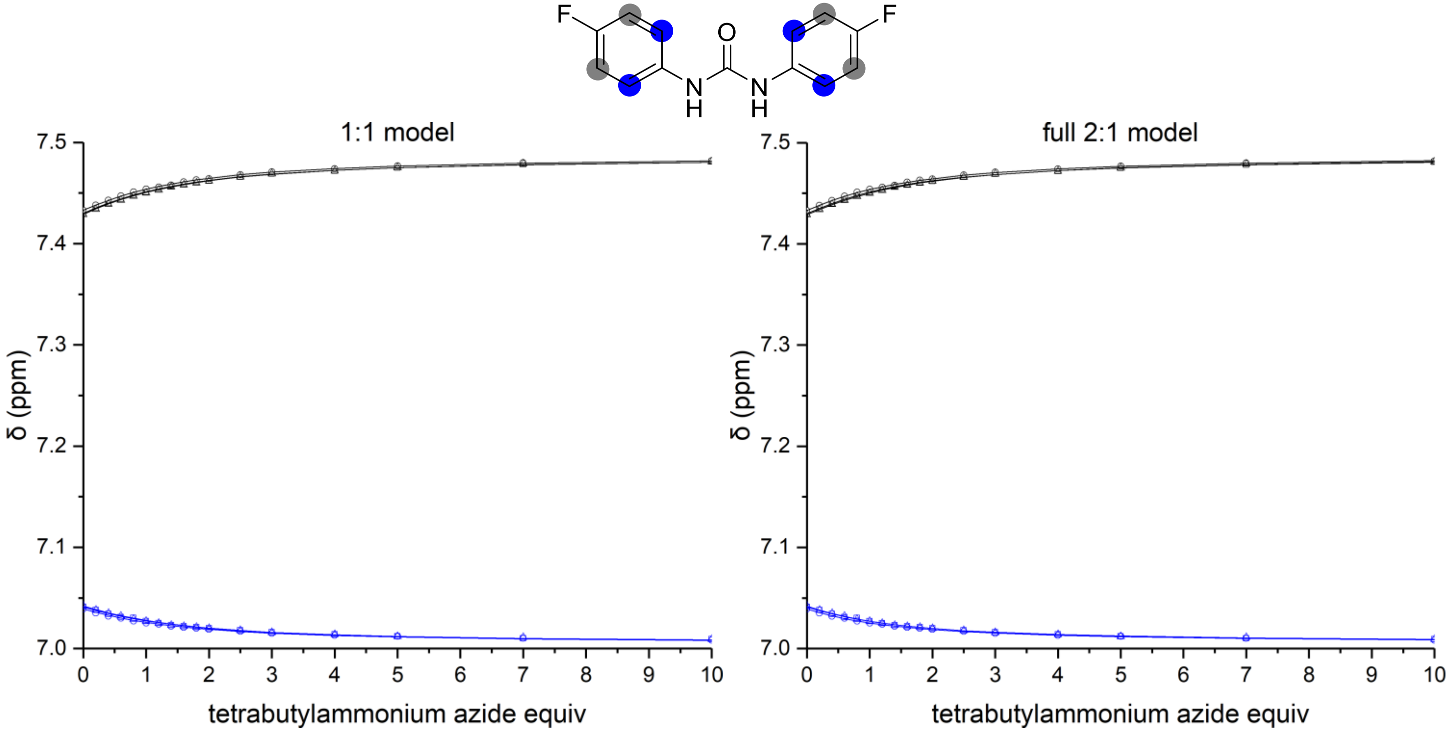

Figure S5 ^{1}H NMR titration data of **1g** with tetrabutylammonium azide. One set of symbols (□, ○, △) refers to experimental data from one set of measurements. Lines are the calculated isotherms of the described model. (2 mM **1g**, CH_3CN/CD_3CN 8:2, 500 MHz, 298 K).

Table S5 Comparison of binding models

model	entry	cov_{fit} (10^{-3})	cov_{fit} factor	$K_{a(1:1)}$ (M^{-1})	$K_{a(2:1)}$ (M^{-1})	$\Delta G_{(1:1)}$ (kJ mol^{-1})	$\Delta G_{(2:1)}$ (kJ mol^{-1})
**1:1**	1	0.19	1	4.81×10^2	-		
	2	0.29	1	4.88×10^2	-		
	3	0.40	1	4.77×10^2	-		
	**mean**	**0.29**	**1**	**$4.82 \pm 0.05 \times 10^2$**	**-**	**-15.31 ± 0.03**	**-**
full 2:1	1	0.14	1.4	7.85×10^2	2.72×10^2		
	2	0.16	1.8	4.56×10^2	6.5×10^1		
	3	0.091	4.4	6.11×10^2	8.9×10^1		
	**mean**	**0.13**	**2.5**	**$6.17 \pm 0.13 \times 10^2$**	**$1.4 \pm 0.9 \times 10^2$**	**-15.92 ± 0.05**	**-12.2 ± 1.6**

cov_{fit} factor is cov_{fit} for the 1:1 model divided by the cov_{fit} for the binding model under study.[13] Values calculated with BindFit v0.5,[11] error is the standard deviation from 3 independent replicas.

The mean cov_{fit} factor for the full 2:1 model is not significantly higher than the mean cov_{fit} for the 1:1 model, suggesting this system is best described by the 1:1 model.

1i and tetrabutylammonium azide

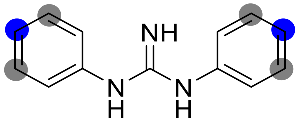

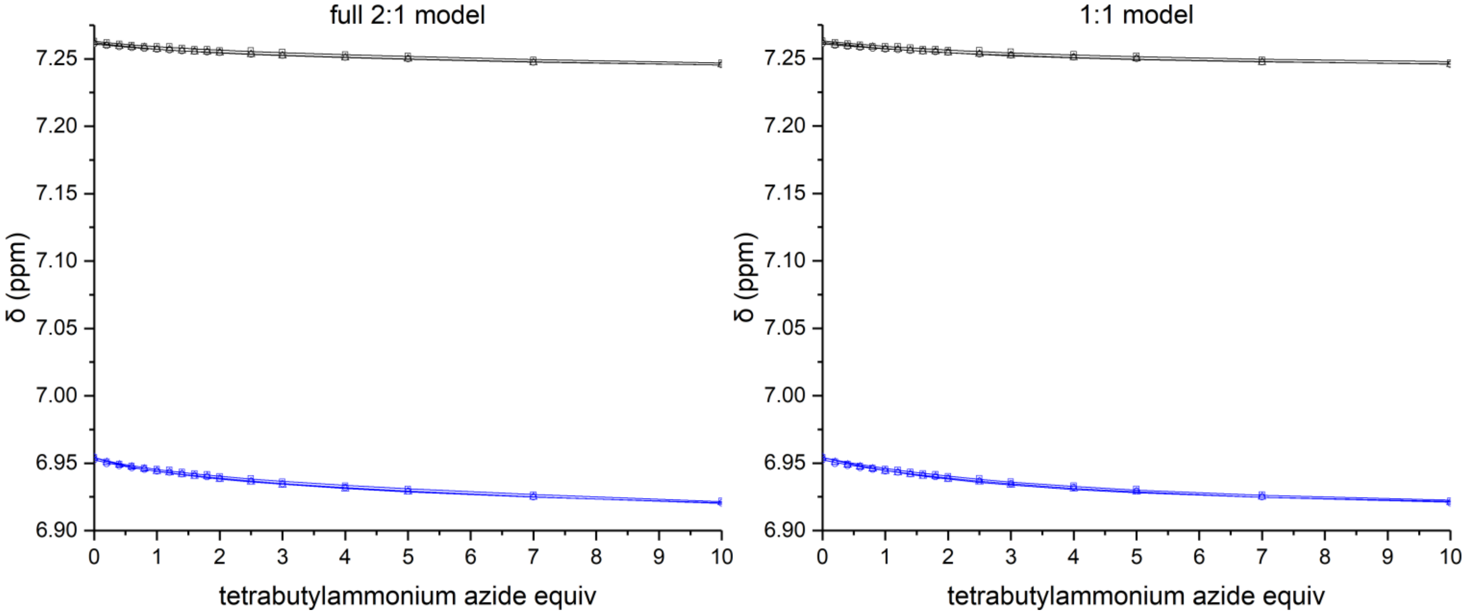

Figure S6 ^{1}H NMR titration data of **1i** with tetrabutylammonium azide. One set of symbols (□, ○, △) refers to experimental data from one set of measurements. Lines are the calculated isotherms of the described model. (2 mM **1i**, CH_3CN/CD_3CN 8:2, 500 MHz, 298 K).

Table S6 Comparison of binding models

model	entry	cov_{fit} (10^{-3})	cov_{fit} factor	$K_{a(1:1)}$ (M^{-1})	$K_{a(2:1)}$ (M^{-1})	$\Delta G_{(1:1)}$ (kJ mol^{-1})	$\Delta G_{(2:1)}$ (kJ mol^{-1})
**1:1**	1	6.70	1	1.23×10^{2}	-		
	2	2.29	1	1.17×10^{2}	-		
	3	4.48	1	1.79×10^{2}	-		
	**mean**	**4.49**	**1**	**$1.4 \pm 0.3 \times 10^{2}$**	**-**	**-12.2 ± 0.5**	**-**
full 2:1	1	2.59	2.6	1.73×10^{1}	7.40×10^{3}		
	2	0.55	4.2	6.34×10^{1}	1.04×10^{3}		
	3	1.86	2.4	9.24×10^{1}	9.53×10^{2}		
	**mean**	**1.67**	**3.1**	**$6 \pm 3 \times 10^{1}$**	**$3 \pm 3 \times 10^{3}$**	**-10.1 ± 1.2**	**-20 ± 2**

cov_{fit} factor is cov_{fit} for the 1:1 model divided by the cov_{fit} for the binding model under study.[13] Values calculated with BindFit v0.5,[11] error is the standard deviation from 3 independent replicas.

The mean cov_{fit} factor for the full 2:1 model is not significantly higher than the mean cov_{fit} for the 1:1 model, suggesting this system is best described by the 1:1 model.

1a and tetrabutylammonium chloride

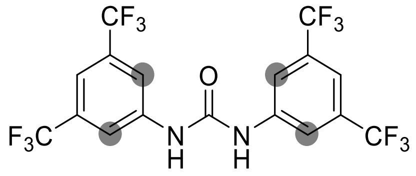

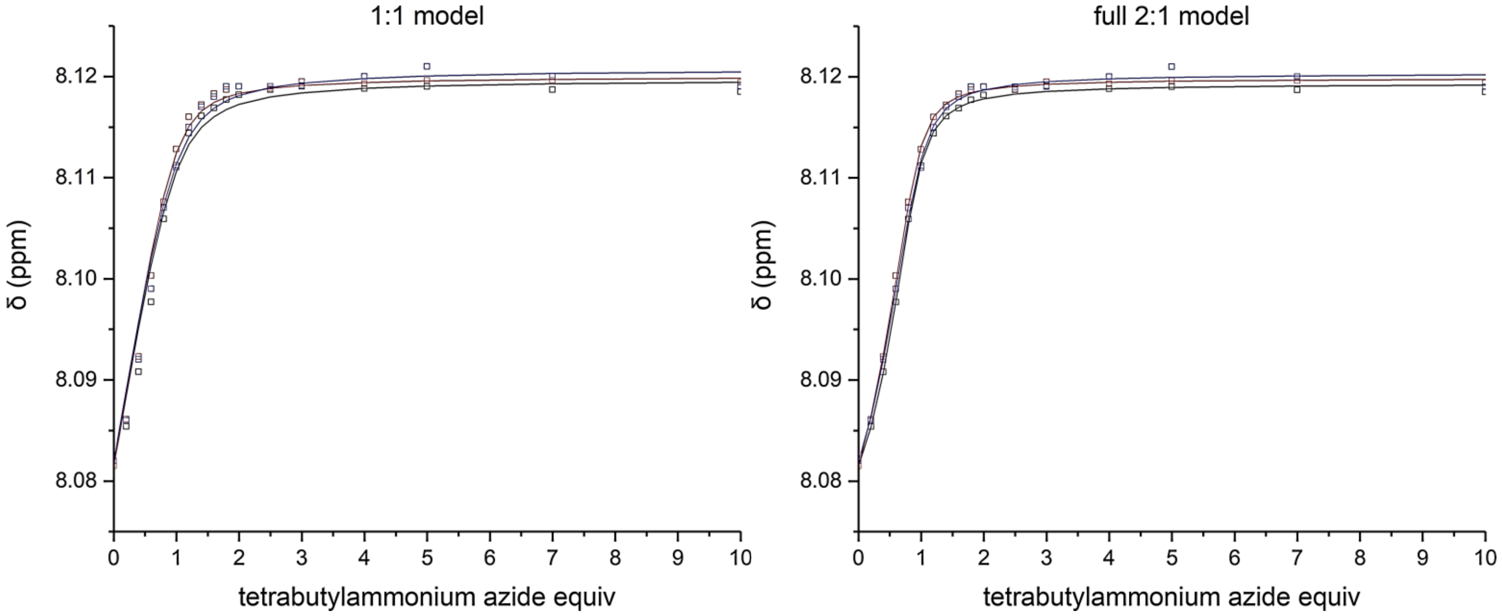

Figure S7 ^{1}H NMR titration data of **1a** with tetrabutylammonium chloride. One set of symbols (□, ○, △) refers to experimental data from one set of measurements. Lines are the calculated isotherms of the described model. (2 mM **1a**, CH_3CN/CD_3CN 8:2, 500 MHz, 298 K).

Table S7 Comparison of binding models

model	entry	cov_{fit} (10^{-3})	cov_{fit} factor	$K_{a(1:1)}$ (M^{-1})	$K_{a(2:1)}$ (M^{-1})	ΔG$_{(1:1)}$ (kJ mol^{-1})	ΔG$_{(2:1)}$ (kJ mol^{-1})
1:1	1	18.31	1	6.77×10^3	-		
	2	10.05	1	1.06×10^4	-		
	3	15.53	1	6.48×10^3	-		
	**mean**	**14.63**	**1**	**$8.0 \pm 1.9 \times 10^3$**	**-**	**-22.3 ± 0.6**	**-**
**full 2:1**	1	0.60	11.0	5.24×10^4	1.02×10^2		
	2	0.46	21.8	6.41×10^4	1.05×10^2		
	3	1.95	8.0	2.54×10^4	3.05×10^2		
	**mean**	**1.00**	**13.6**	**$4.7 \pm 1.6 \times 10^4$**	**$1.7 \pm 0.9 \times 10^2$**	**-26.7 ± 0.8**	**-12.7 ± 1.3**

cov_{fit} factor is cov_{fit} for the 1:1 model divided by the cov_{fit} for the binding model under study.[13] Values calculated with BindFit v0.5,[11] error is the standard deviation from 3 independent replicas.

The mean cov_{fit} factor for the full 2:1 model is significantly higher than the mean cov_{fit} for the 1:1 model, suggesting this system is best described by the full 2:1 model.

(*S*)-1k and tetrabutylammonium azide

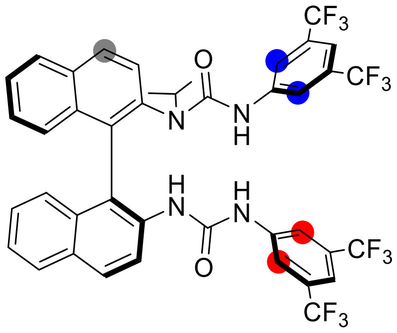

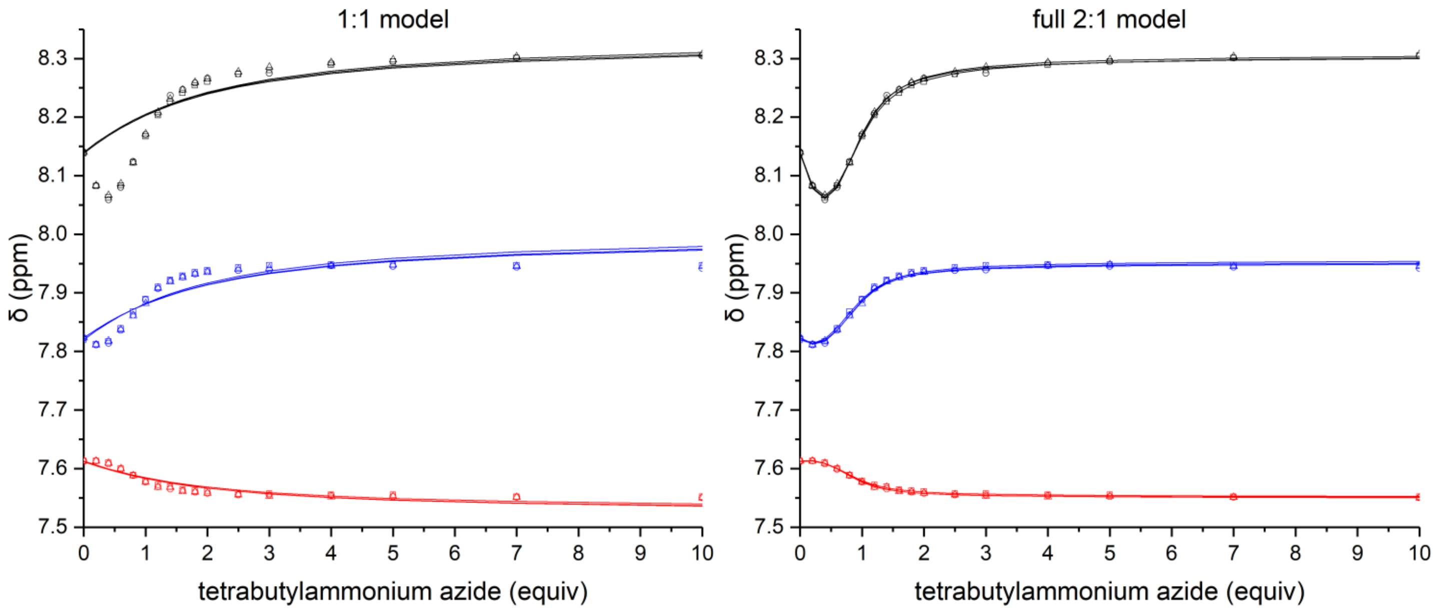

Figure S8 ^{1}H NMR titration data of (*S*)-**1k** with tetrabutylammonium azide. One set of symbols (□, ○, △) refers to experimental data from one set of measurements. Lines are the calculated isotherms of the described model. (2 mM (*S*)-**1k**, $CDCl_3$ 8:2, 500 MHz, 298 K).

Table S8 Comparison of binding models

model	entry	cov_{fit} (10^{-3})	cov_{fit} factor	$K_{a(1:1)}$ (M^{-1})	$K_{a(2:1)}$ (M^{-1})	$\Delta G_{(1:1)}$ (kJ mol^{-1})	$\Delta G_{(2:1)}$ (kJ mol^{-1})
1:1	1	126.72	1	3.77×10^2	-		
	2	137.94	1	3.68×10^2	-		
	3	127.35	1	3.58×10^2	-		
	**mean**	**130.67**	**1**	**$3.68 \pm 0.08 \times 10^2$**	**-**	**-14.64 ± 0.05**	**-**
**full 2:1**	1	0.67	189.1	9.17×10^3	1.67×10^2		
	2	0.96	143.7	1.03×10^4	1.16×10^2		
	3	0.53	240.3	7.95×10^3	1.69×10^1		
	**mean**	**0.72**	**191.0**	**$9.1 \pm 0.9 \times 10^3$**	**$1.0 \pm 0.6 \times 10^2$**	**-22.7 ± 0.2**	**-11.4 ± 1.5**

cov_{fit} factor is cov_{fit} for the 1:1 model divided by the cov_{fit} for the binding model under study.[13] Values calculated with BindFit v0.5,[11] error is the standard deviation from 3 independent replicas.

The mean cov_{fit} factor for the full 2:1 model is significantly higher than the mean cov_{fit} for the 1:1 model, suggesting this system is best described by the full 2:1 model.

(*S*)-1k and tetrabutylammonium chloride

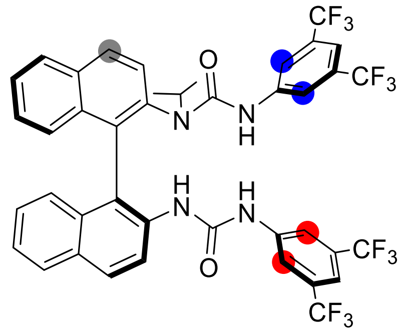

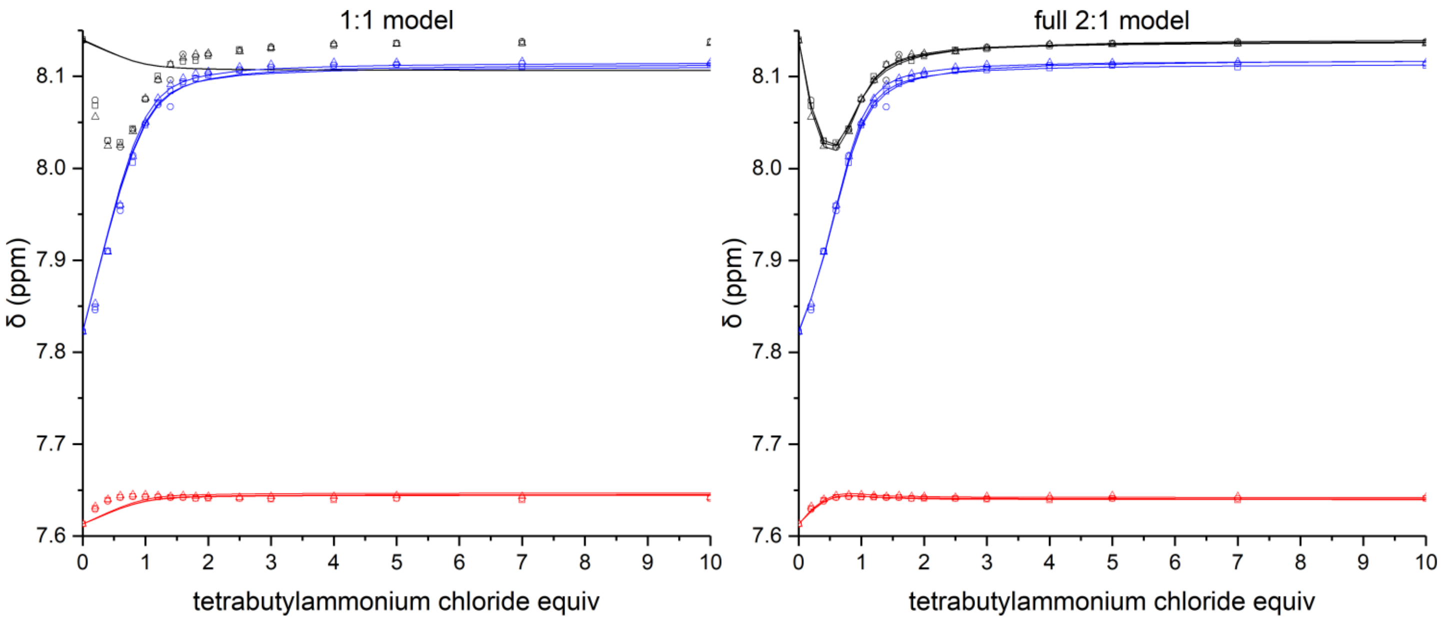

Figure S9 ¹H NMR titration data of (*S*)-**1k** with tetrabutylammonium chloride. One set of symbols (□, ○, △) refers to experimental data from one set of measurements. Lines are the calculated isotherms of the described model. (2 mM (*S*)-**1k**, $CDCl_3$ 8:2, 500 MHz, 298 K).

Table S9 Comparison of binding models

model	entry	*cov*$_{fit}$ (10^{-3})	*cov*$_{fit}$ factor	$K_{a(1:1)}$ (M^{-1})	$K_{a(2:1)}$ (M^{-1})	$\Delta G_{(1:1)}$ (kJ mol^{-1})	$\Delta G_{(2:1)}$ (kJ mol^{-1})
1:1	1	46.11	1	9.03×10^3	-		
	2	48.29	1	7.67×10^3	-		
	3	49.37	1	9.44×10^3	-		
	**mean**	**47.92**	**1**	**$8.7 \pm 0.8 \times 10^3$**	**-**	**-22.5 ± 0.2**	**-**
**full 2:1**	1	0.31	148.7	1.85×10^4	1.90×10^2		
	2	1.11	43.5	2.66×10^4	9.87×10^2		
	3	0.28	176.3	1.96×10^4	1.47×10^2		
	**mean**	**0.57**	**122.8**	**$2.2 \pm 0.4 \times 10^4$**	**$4 \pm 3 \times 10^2$**	**-24.8 ± 0.5**	**-15 ± 2**

cov$_{fit}$ factor is *cov*$_{fit}$ for the 1:1 model divided by the *cov*$_{fit}$ for the binding model under study.[13] Values calculated with BindFit v0.5,[11] error is the standard deviation from 3 independent replicas.

The mean *cov*$_{fit}$ factor for the full 2:1 model is significantly higher than the mean *cov*$_{fit}$ for the 1:1 model, suggesting this system is best described by the full 2:1 model.

^{14}N Studies

^{14}N NMR spectra were obtained on a Bruker AVANCE III 500 MHz spectrometer equipped with a 5 mm z-gradient broadband X-^{19}F/^{1}H BBFO SMART probe. An anti-ringing proton decoupled pulse sequence 'aringdec' was used. Chemical shifts referenced to external $NH_{3(l)}$.

25 mM solutions of tetrabutylammonium azide, (*S*)-**1k,** and (*S*)-**1k**·N_3·Bu_4N were measured. The (*S*)-**1k**·N_3·Bu_4N sample was prepared by dissolving tetrabutylammonium azide (5 mg) with a solution of (*S*)-**1k** in $CDCl_3$ (0.7 mL, 25 mM).

Tetrabutylammonium azide

^{14}N NMR (36 MHz, $CDCl_3$)

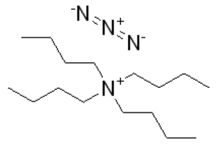

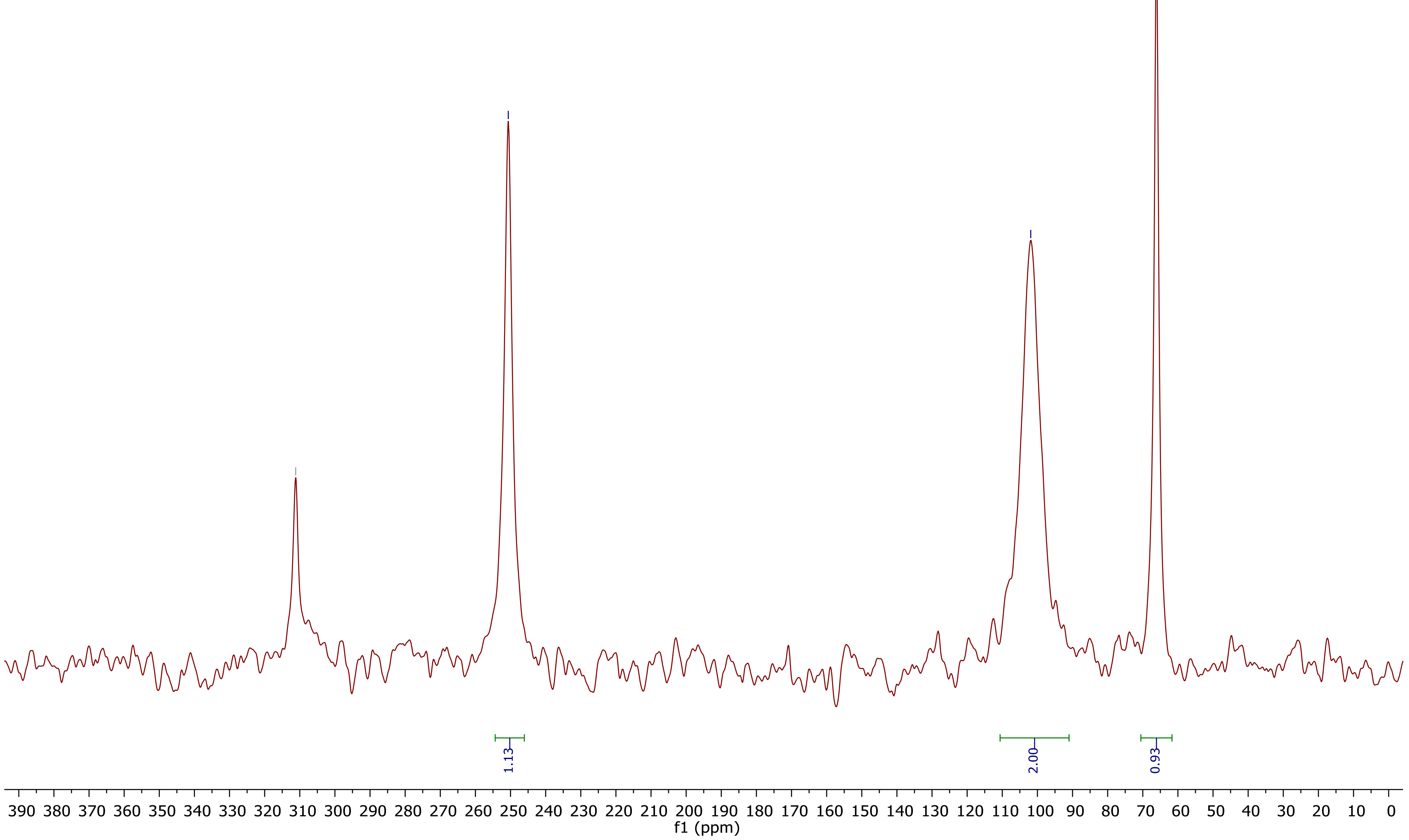

(*S*)-1k

[14]N NMR (36 MHz, $CDCl_3$)

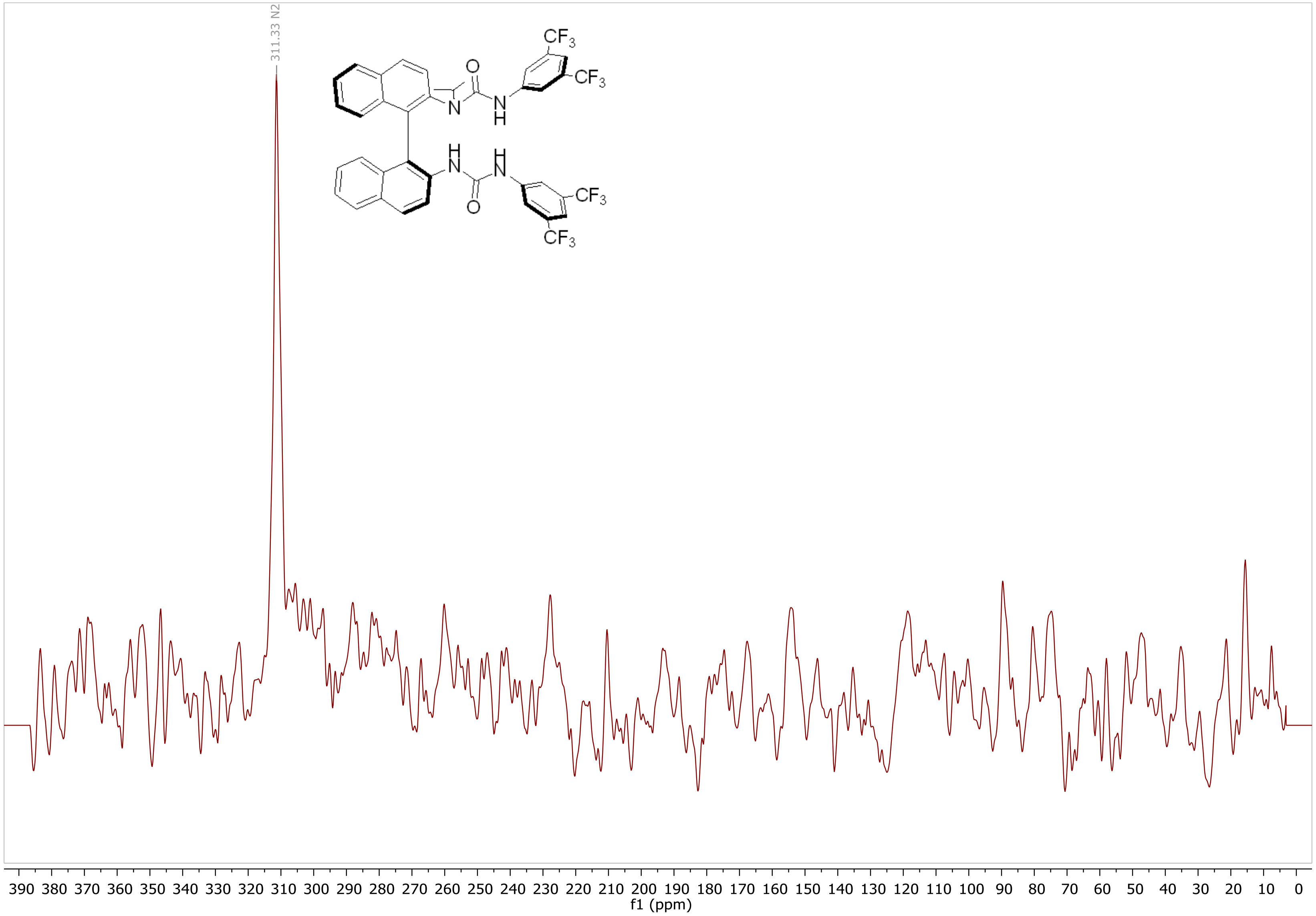

(*S*)-1k·N_3·Bu_4N

^{14}N NMR (36 MHz, $CDCl_3$)

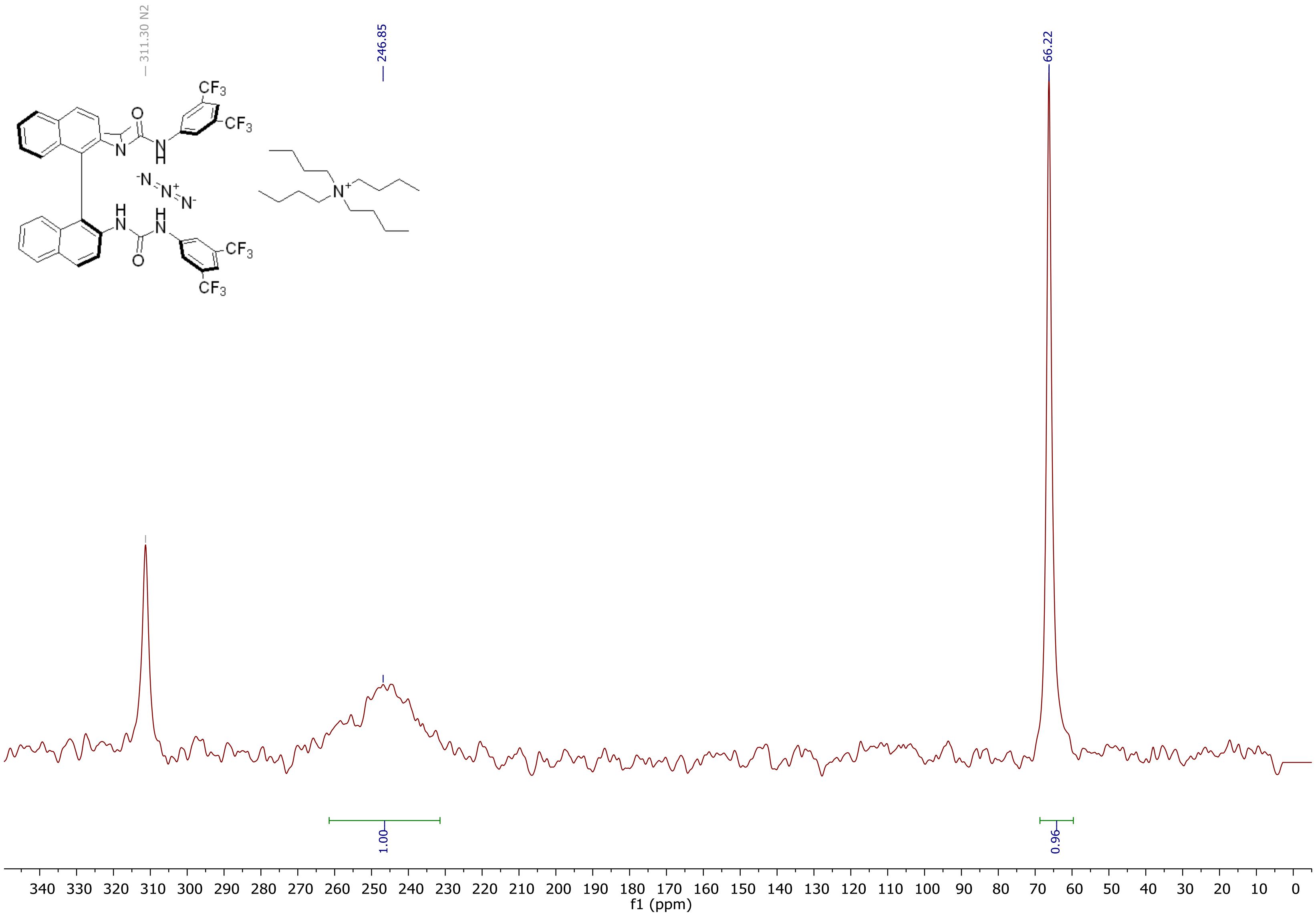

Study of (*S*)-1k·[1-^{15}N]N_3·Bu_4N

Concentration dependence of spectra

A 1:1 mixture of (*S*)-**1k**·[1-^{15}N]N_3·Bu_4N at 100 mM was prepared by dissolving (*S*)-**1k** (83.7 mg) and tetrabutylammonium [1-^{15}N]azide (28.5 mg) with $CDCl_3$ in a 1 mL volumetric flask.

A 25 mM solution was prepared with 250 μL of (*S*)-**1k**·[1-^{15}N]N_3·Bu_4N (100 mM) and $CDCl_3$ in a 1 mL volumetric flask.

A 2.5 mM solution was prepared with 100 μL of (*S*)-**1k**·[1-^{15}N]N_3·Bu_4N (25 mM) and $CDCl_3$ in a 1 mL volumetric flask.

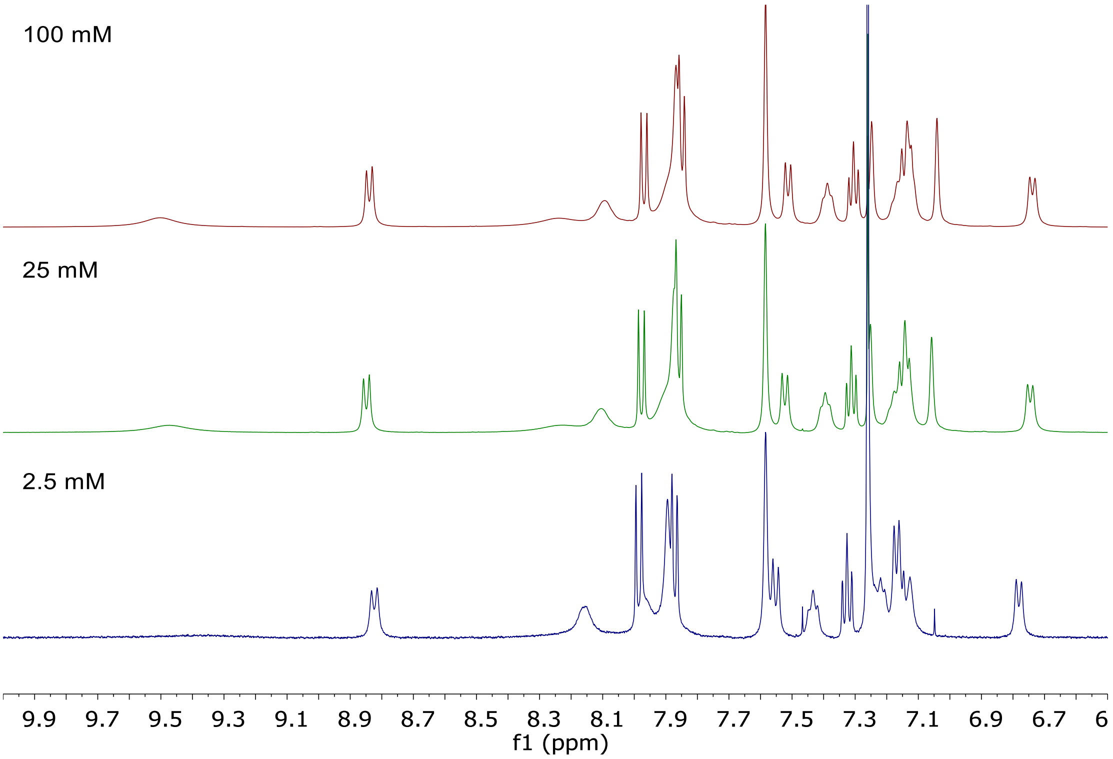

Figure S10 Partial ^{1}H spectra of (*S*)-**1k**·Bu_4N·[1-^{15}N]N_3 at 100 mM, 25 mM, and 2.5 mM (*S*)-**1k**, $CDCl_3$, 500 MHz, 298 K).

Sample preparation

The sample was prepared by weighing tetrabutylammonium [1-^{15}N]azide (5.2 mg, 1 equiv) into an NMR tube equipped with a J. Young valve under N_2. A solution of (*S*)-**1k** in $CDCl_3$ (0.73 mL, 0.25 mM, 1 equiv) was added, and the sample was frozen with liquid N_2, evacuated, and flushed with N_2 once.

(*S*)-1k·[1-^{15}N]N_3·Bu_4N ($CDCl_3$, 25 mM)

The assignment of the ^{1}H shifts of (*S*)-**1k**·[1-^{15}N]N_3·Bu_4N in $CDCl_3$ at rt was achieved by tracking ^{1}H CH signals along the titration, with additional assistance from 2D ^{1}H-^{1}H COSY and ^{1}H-^{1}H NOESY spectra.

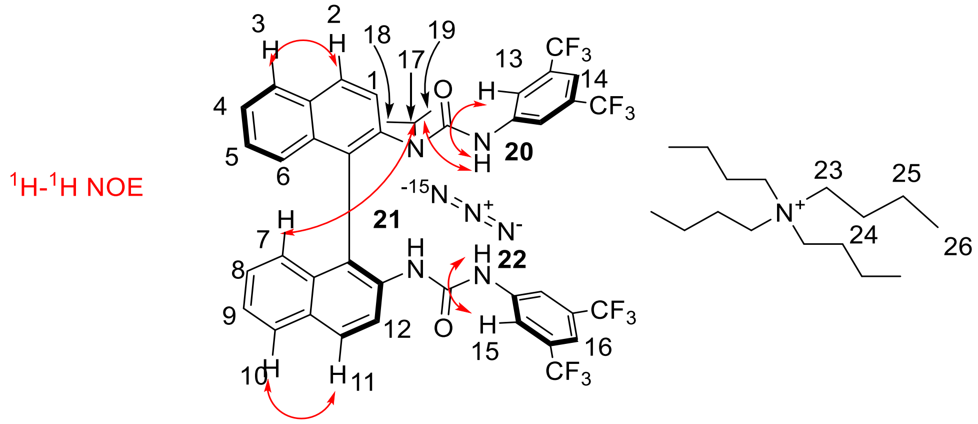

Additional support for NH assignments was provided by variable temperature NMR of this sample, and variable temperature (VT) NMR studies of a sample containing excess tetrabutylammonium [1-^{15}N]azide *vida infra*.

^{1}H NMR (600 MHz, $CDCl_3$) δ 9.49 (s, 1H, **H22**), 8.85 (d, *J* = 9.0 Hz, 1H, **H12**), 8.23 (s, 1H, **H20**), 8.10 (br s, 1H, **H2**), 7.98 (d, *J* = 9.0 Hz, 1H, **H11**), 7.94 – 7.80 (m, 4H, **H3**, **H10**, **H21**, **H13**), 7.59 (s, 2H, **H15**), 7.52 (d, *J* = 8.5 Hz, 1H, **H1**), 7.39 (br s, 1H, **H4**), 7.31 (t, *J* = 7.5 Hz, 1H, **H8**), 7.25 (s, 1H, **H16**), 7.21 – 7.10 (m, 3H, **H5**, **H6**, **H9**), 7.06 (s, 1H, **H14**), 6.75 (d, *J* = 8.0 Hz, 1H, **H7**), 3.14 – 2.98 (m, 9H, **H23**, **H17**), 1.62 – 1.41 (m, 8H, **H24**), 1.34 (sept, *J* = 7.5 Hz, 8H, **H25**), 1.18 (d, *J* = 6.5 Hz, 3H, **H19**), 0.95 (t, *J* = 7.5 Hz, 12H, **H26**), 0.37 (d, *J* = 6.5 Hz, 3H, **H18**).

^{1}H NMR (600 MHz, $CDCl_3$)

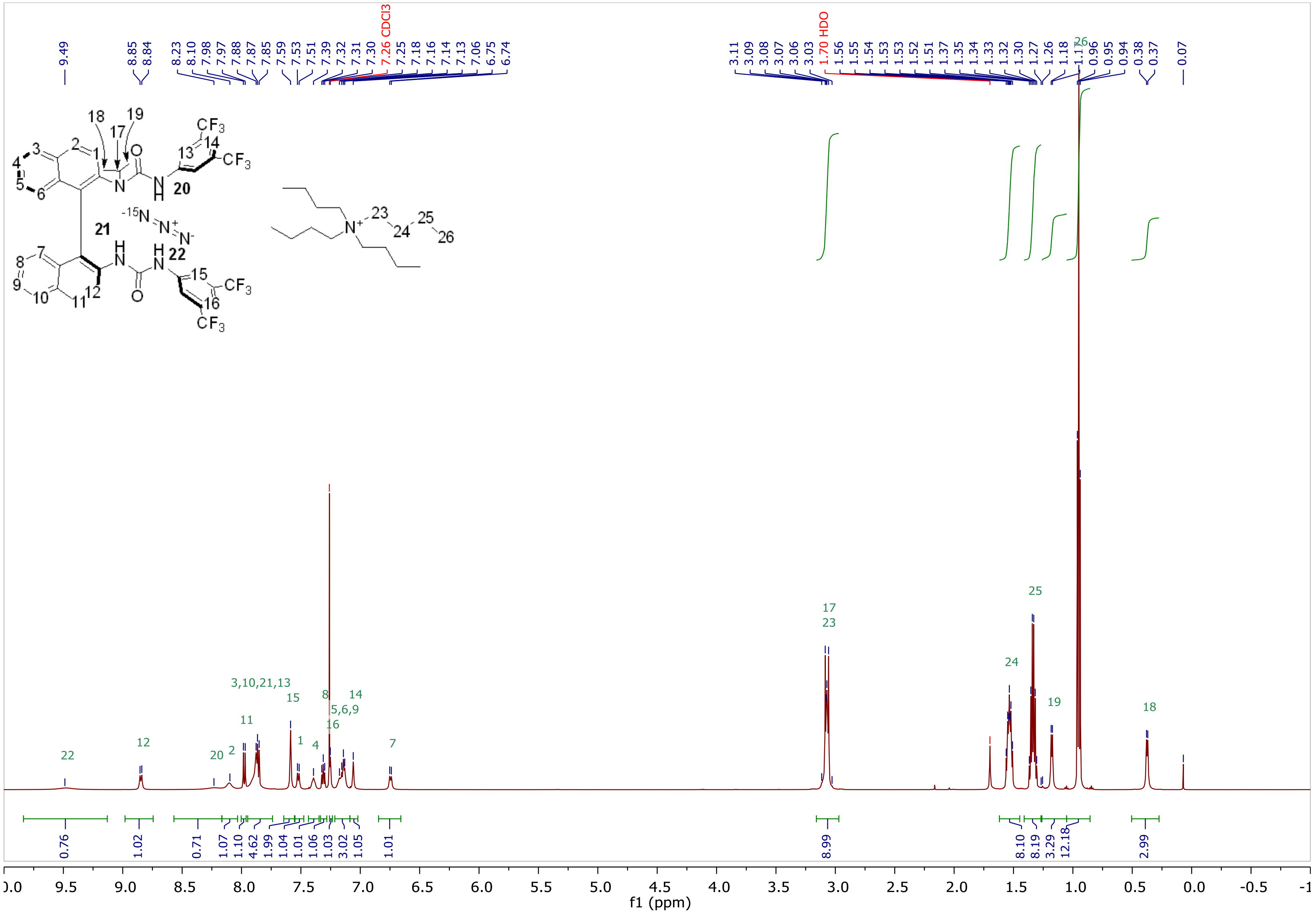

Expansion of above ^{1}H NMR

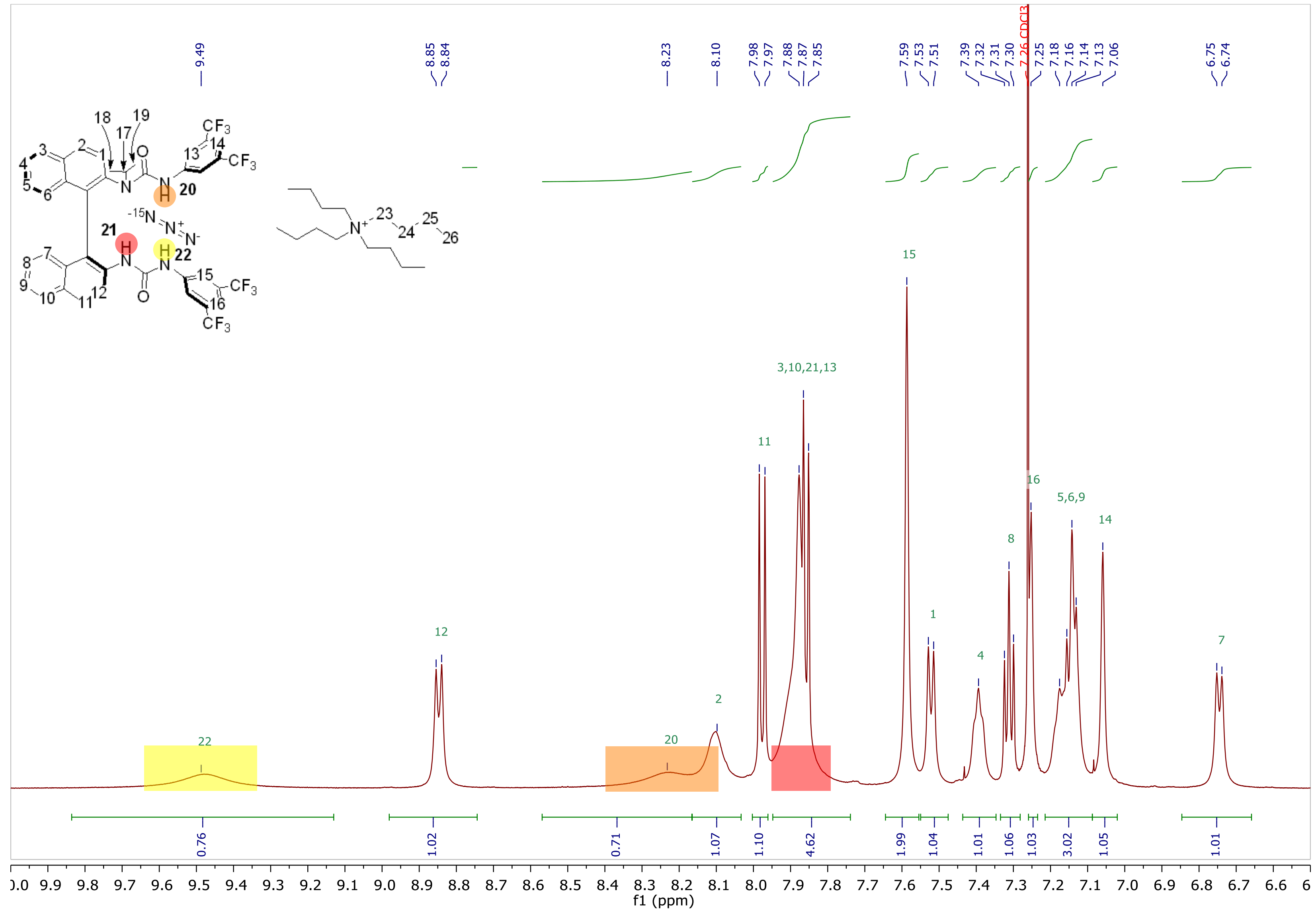

^{1}H-^{1}H COSY (600 MHz, $CDCl_3$)

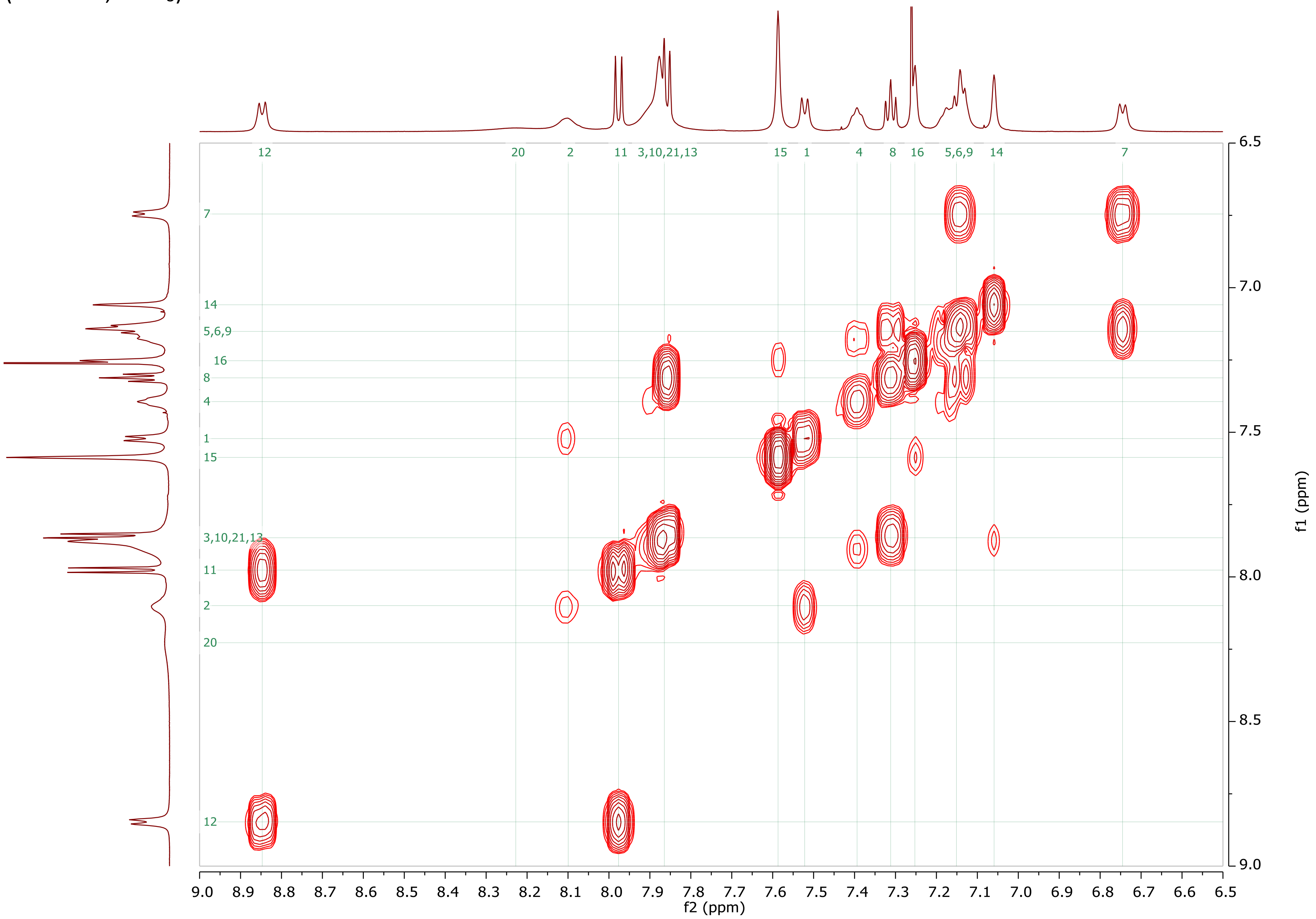

^{1}H-^{1}H NOESY (600 MHz, $CDCl_3$)

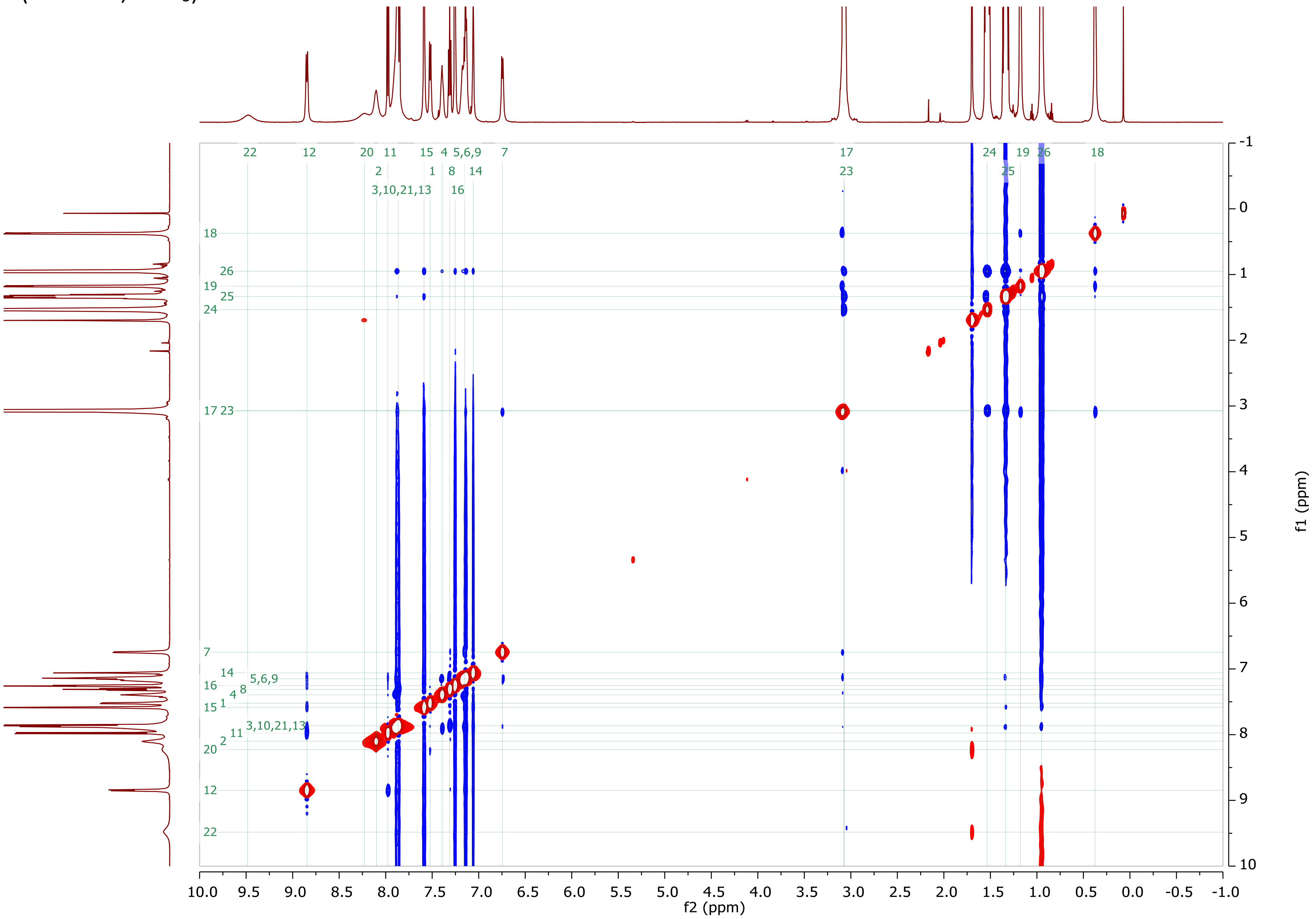

Expansion of above **^{1}H-^{1}H NOESY**

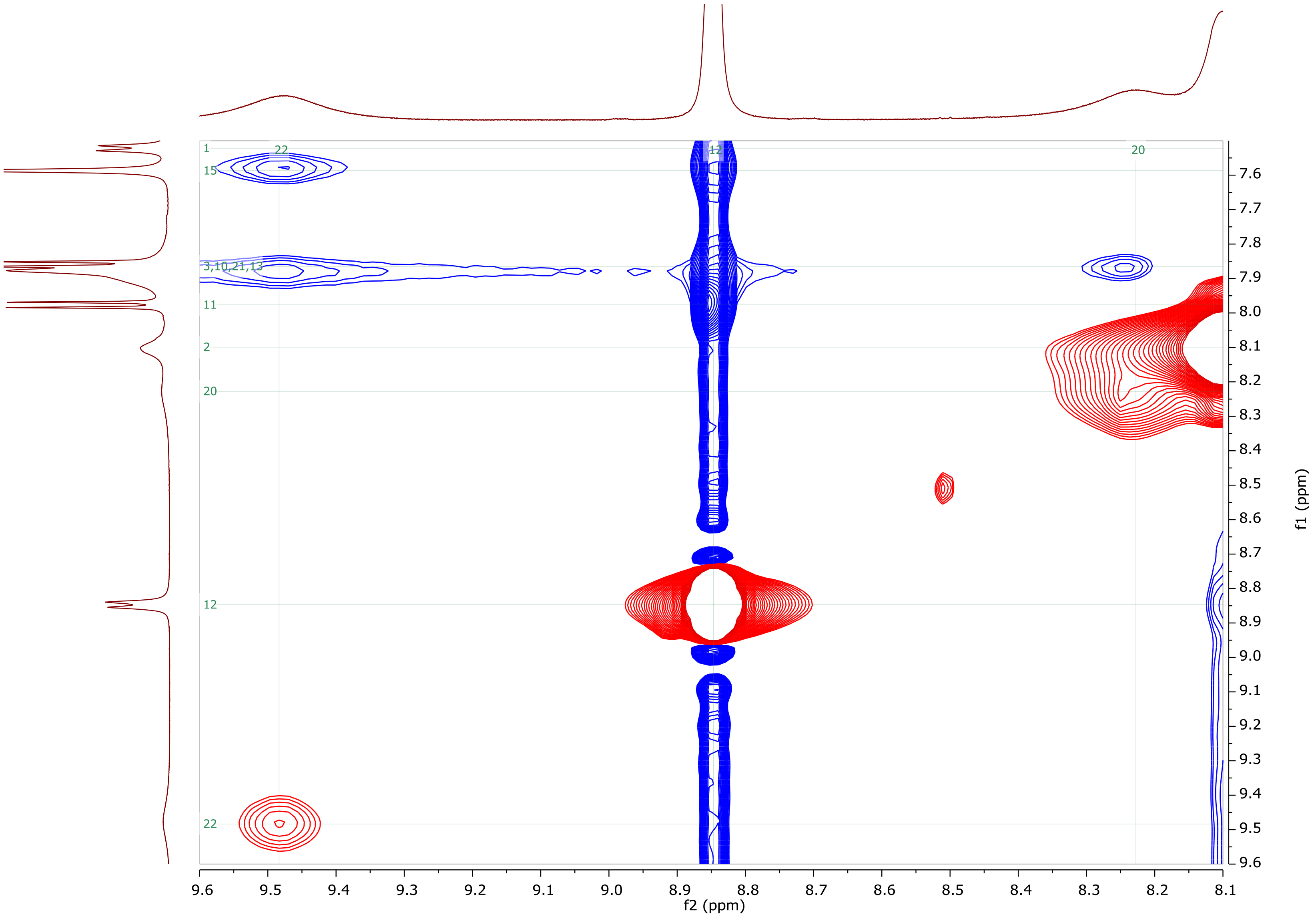

^{15}N [1-^{15}N]-Azide T_1 Measurement

The previously prepared 25 mM sample of (*S*)-**1k**·[1-^{15}N]N_3·Bu_4N in $CDCl_3$ was studied. A 25 mM sample of tetrabutylammonium [1-^{15}N]azide in $CDCl_3$ was also prepared.

^{15}N T_1 measurements were conducted on a Bruker AVIII HD 600 MHz spectrometer equipped with a Prodigy N_2 broadband cryoprobe. T_1 measurements used the inversion-recovery method.

The data were fitted to eqn (1) using TopSpin 4.0.2 in order to determine the ^{15}N [1-^{15}N]azide T_1 of each sample. *I*[0] is the relative equilibrium peak intensity (ideal = 1) and *P* is the inverted peak intensity factor that is variable in the fit to allow for an imperfect initial inversion condition (*P* = -2·*I*[0] for ideal inversion).

$$I[t] = I[0] + Pe^{-\frac{t}{T_1}} \tag{1}$$

Table S10 [1-^{15}N]-azide T_1 acquisition parameters and values.

entry	sample	F1 (slices/scans)	*t* (s)	T_1 (s)
1	(*S*)-**1k**·[1-^{15}N]N_3·Bu_4N	10/8	0.001, 1, 3, 5, 8, 10, 15, 20, 25, 50	3.5
2	Bu_4N·[1-^{15}N]N_3	8/8	0.001, 10, 50, 80, 120, 180, 240, 300	39

^{1}H-^{15}N Heteronuclear NOE

The previously prepared 25 mM sample of (*S*)-**1k**·[1-^{15}N]N_3·Bu_4N in $CDCl_3$ was studied.

^{1}H-^{15}N NOE measurements were conducted on a Bruker AVIII HD 600 MHz spectrometer equipped with a Prodigy N_2 broadband cryoprobe. ^{1}H chemical shifts are referenced to residual $CHCl_3$ and ^{15}N chemical shifts are referenced to external $NH_{3(l)}$.

Steady-state ^{1}H to ^{15}N NOEs were generated using selective presaturation of ^{1}H resonances followed by acquisition of the ^{15}N 1D spectrum (**Figure S11A**). Control experiments were performed by off-resonance irradiation at ±20 ppm for all measurements. Due to crowding of the ^{1}H resonances, various B_1 *rf* field strengths for ^{1}H presaturation were investigated, providing a balance of selectivity versus extent of ^{1}H saturation, as directly observed in ^{1}H spectra following selective presaturation (**Figure S11B**). Series of NOE experiments were recorded using B_1 *rf* fields of 25 and 5 Hz, the former providing stronger NOEs but with spill over of saturation to neighbouring resonances in the ^{1}H spectrum, whereas the latter provided for greater selectivity but weaker NOEs. Total ^{1}H irradiation times of up to 40 s (>5×T_1) were employed.

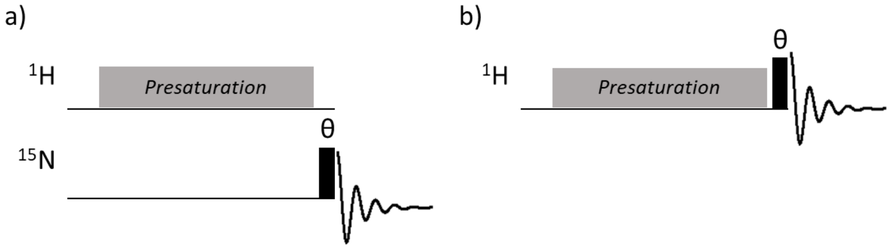

Figure S11 (A) Schematic NMR sequences for ^{1}H-^{15}N steady state NOE observations. (B) Schematic NMR sequences for optimisation of ^{1}H presaturation conditions. Grey box indicates presaturation of a ^{1}H resonance during relaxation delay and black rectangle indicates excitation pulse for acquisition (β = 30° or 90°).

NOE effects in small molecules are expected to lead to a reduction in the ^{15}N resonance intensity due to the negative magnetogyric ratio of ^{15}N. In the case of the azide complexes, any NOE effect will be moderated by exchange between free and bound states of the azide, and from the fact that this is labelled only at one end of the molecule with a single ^{15}N isotope label, leading to a statistical distribution of bound orientations. NOE intensity changes were quantified by direct comparison with off-resonance control spectra and expressed as percentage changes.

Irradiation selectivity at 25 Hz rf

Irradiation at 9.48 ppm.

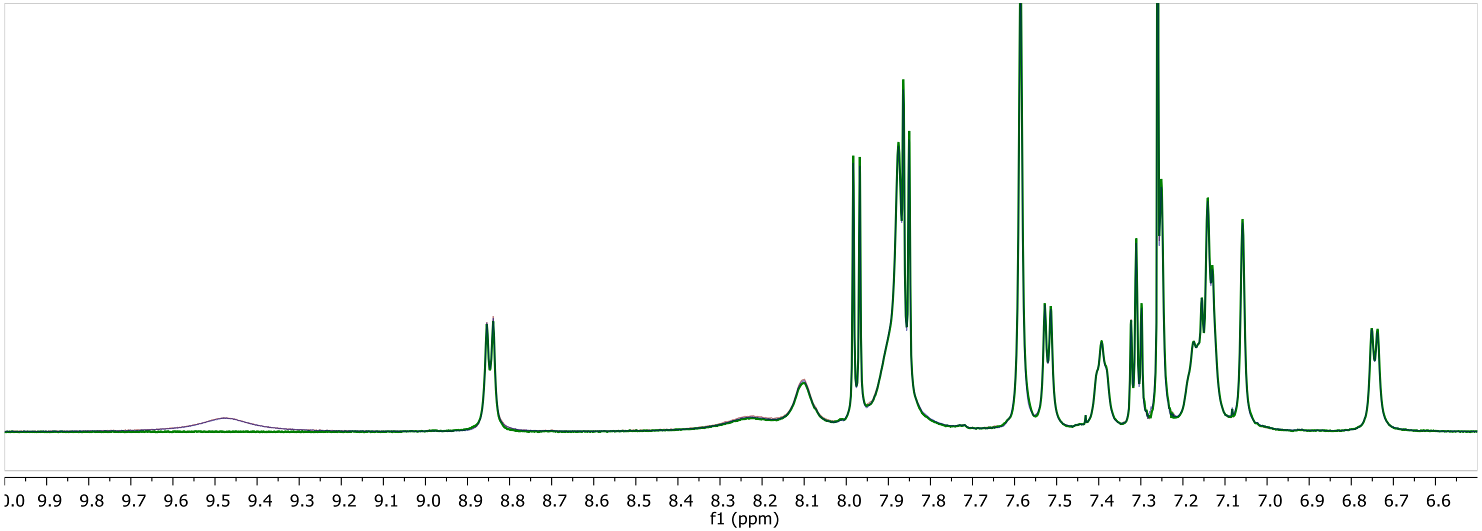

Irradiation at 8.85 ppm.

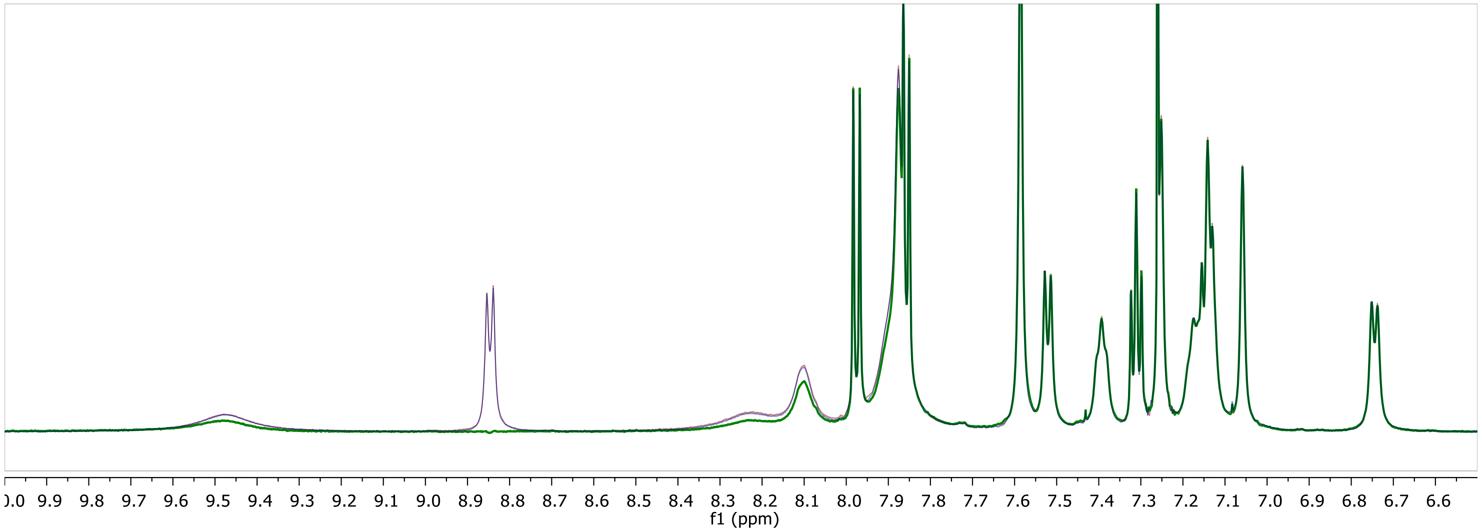

Irradiation at 8.23 ppm.

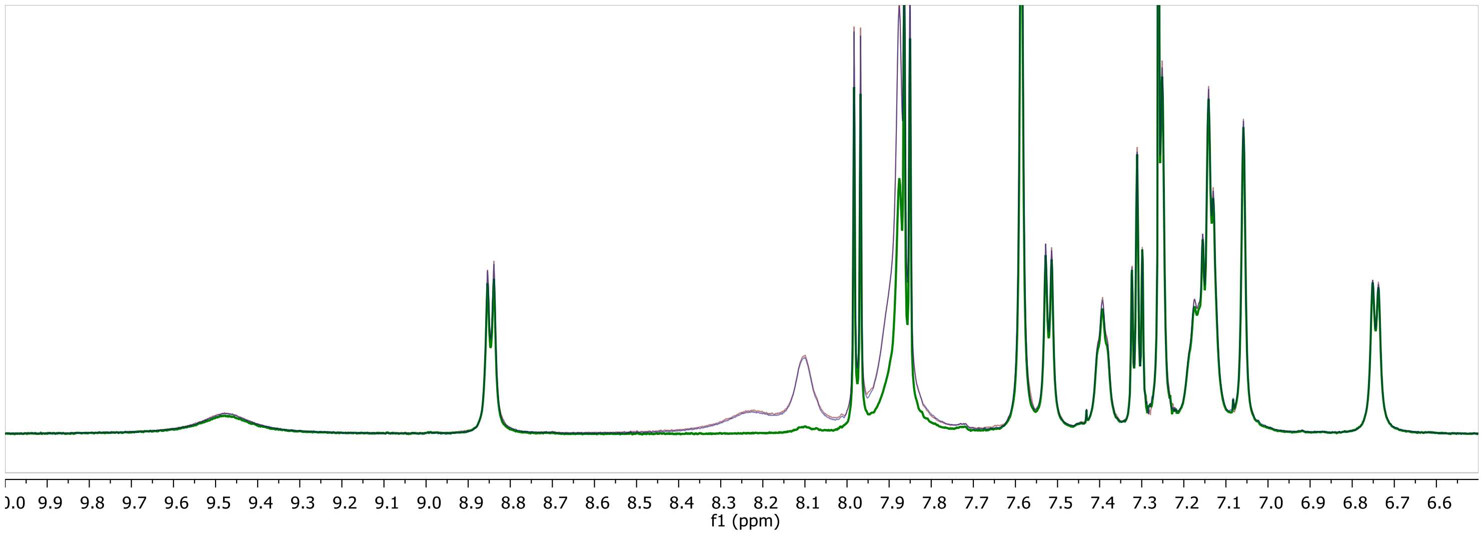

Irradiation at 7.90 ppm.

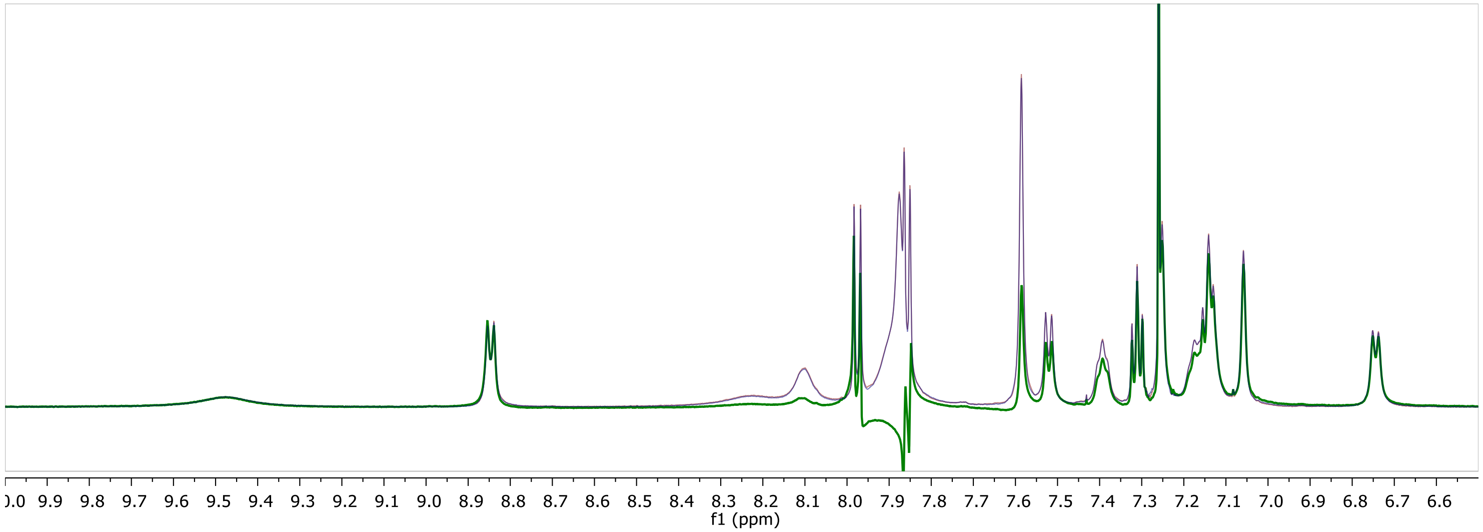

Irradiation at 6.75 ppm.

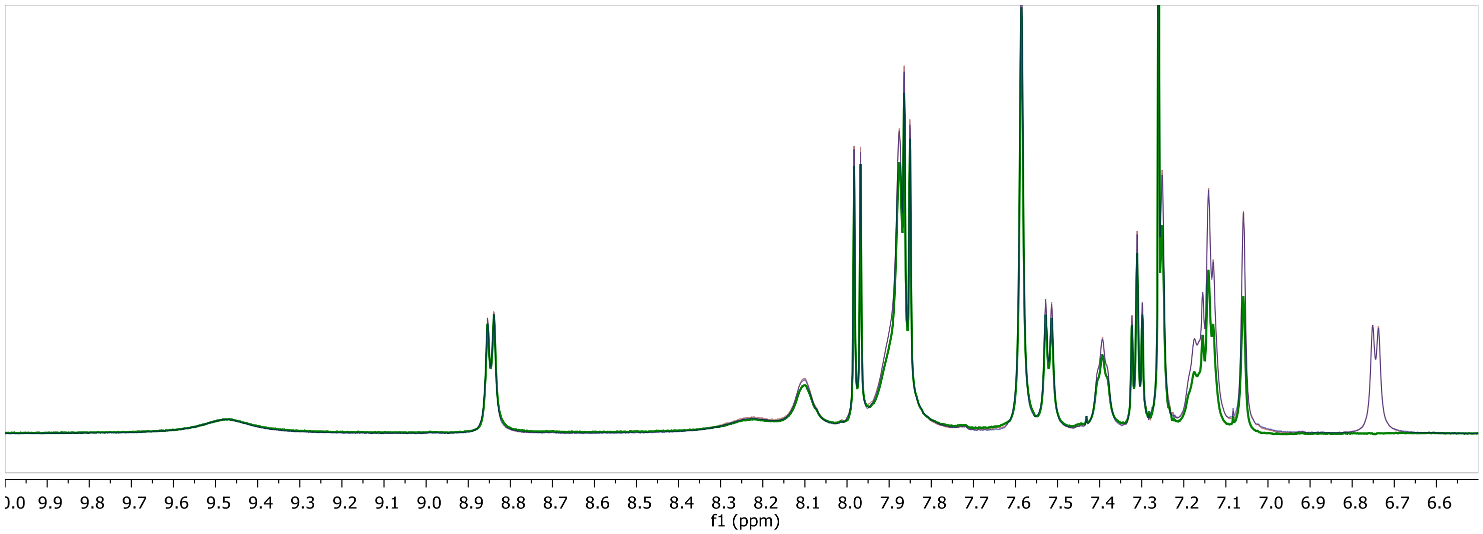

^{1}H-^{15}N NOEs at 25 Hz rf

O1P (ppm)	20	9.48	8.85[a]	8.23	7.90	6.75	3.07	1.53	1.18	0.38	-20
	off-resonance control	**H22**	H12	**H20**	**H21**, H10, H3, H13	H7	H17, H20	H21	H19	H18	off-resonance control
Δ*I*	0%	-12%	-8%	-13%	-14%	-3%	3%	0%	-2%	-3%	0%

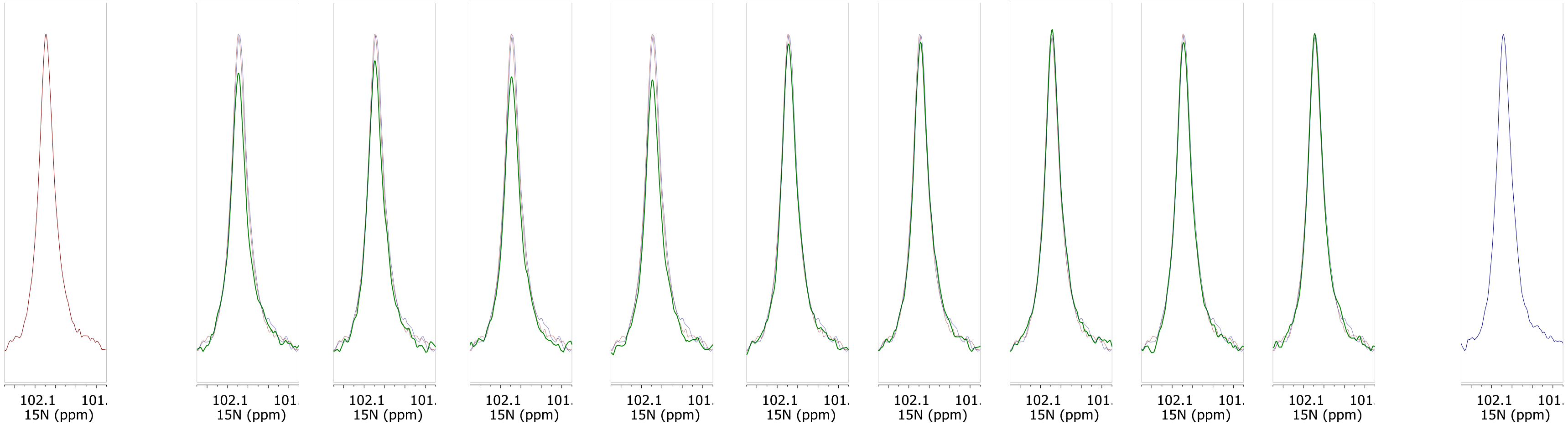

Figure S12 Change in ^{15}N signal intensity (Δ*I*) caused by irradiation at indicated chemical shift (δH). The ^{1}H rf field strength (B_1) was 25 Hz and the presaturation time was 40 s. [a]Note irradiation at 8.85 ppm led to partial saturation of the neighbouring NH at 9.48 ppm which likely causes the observed NOE.

Irradiation selectivity at 5 Hz rf

Irradiation at 9.48 ppm.

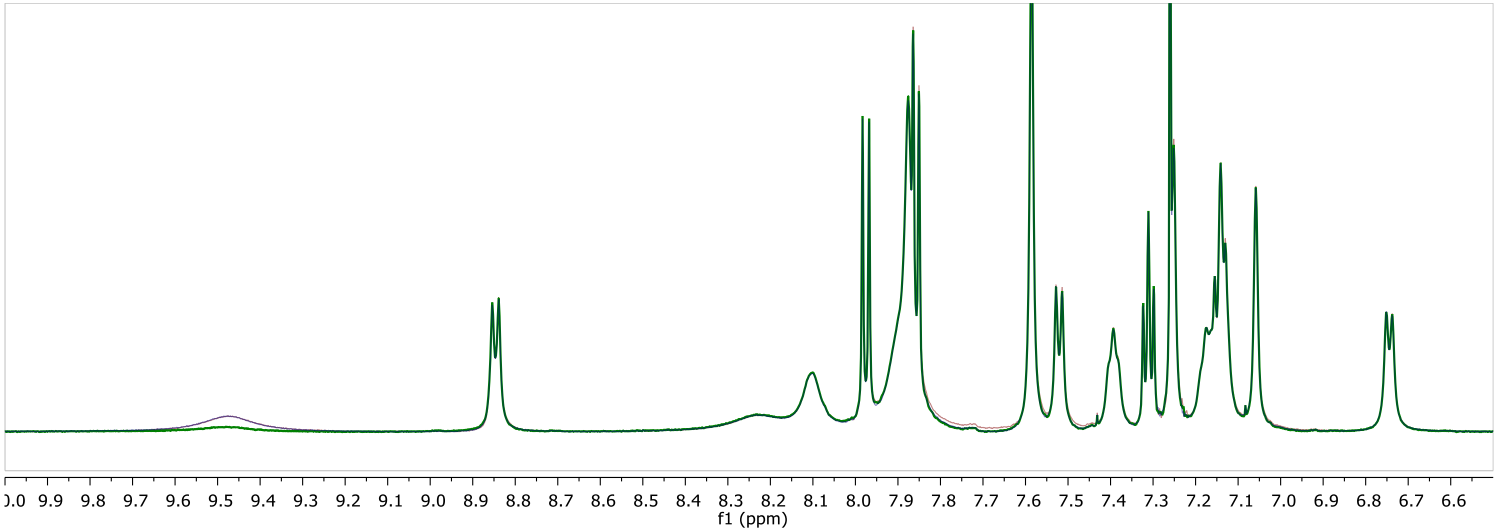

Irradiation at 8.85 ppm.

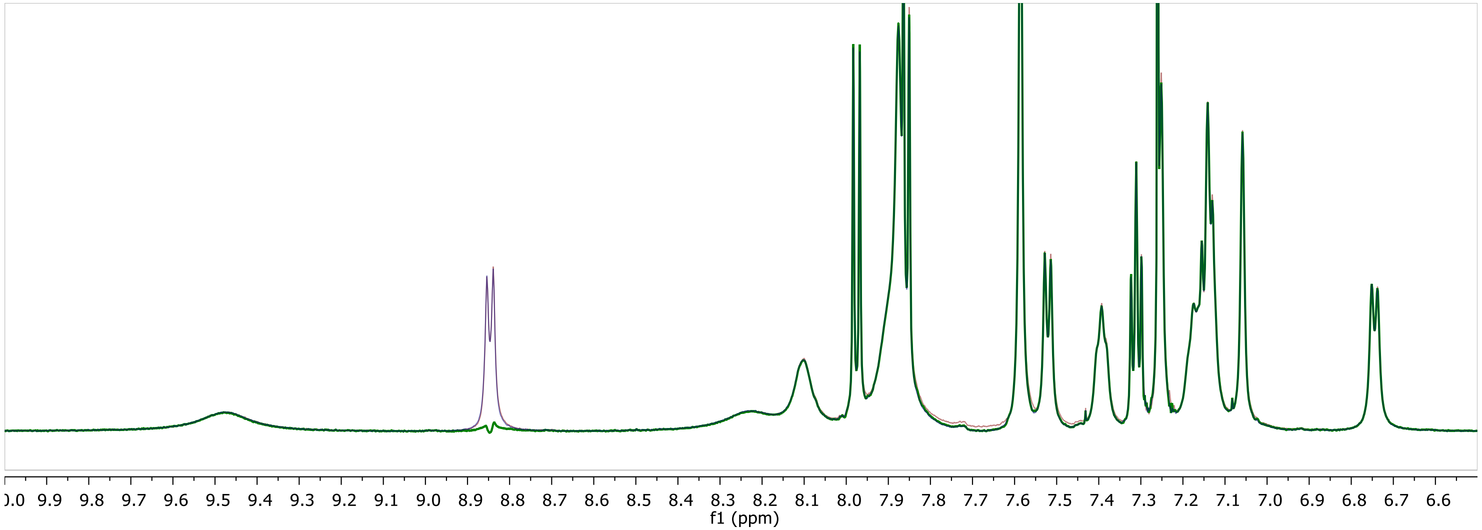

Irradiation at 8.23 ppm.

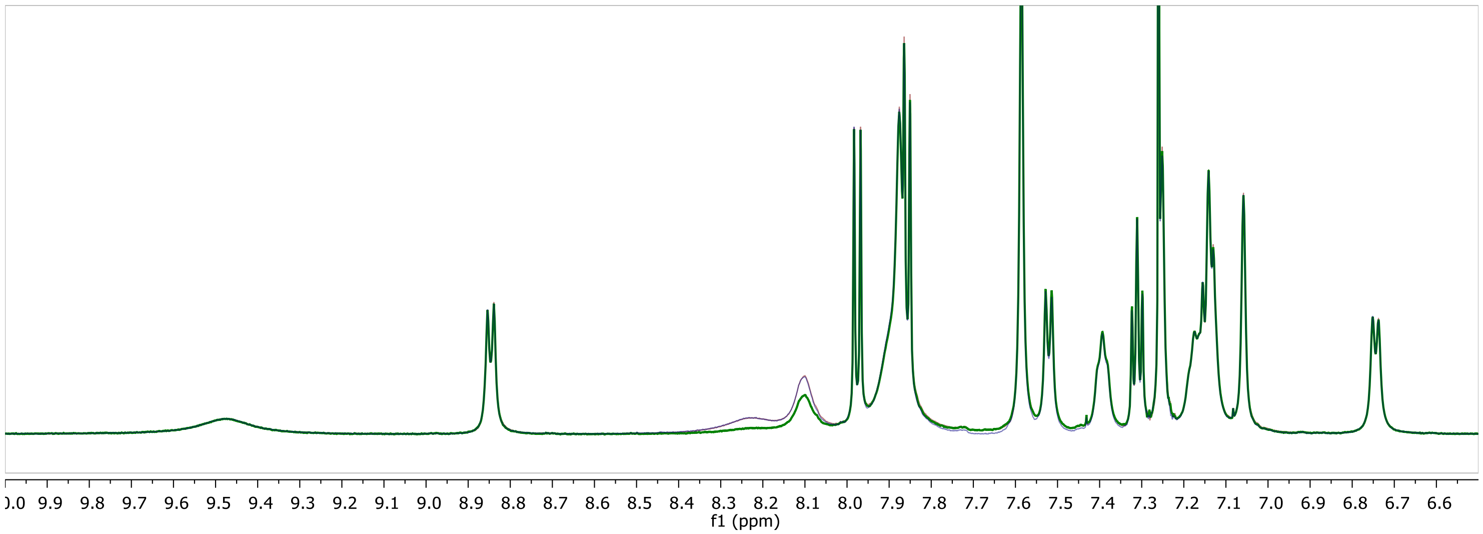

Irradiation at 7.90 ppm.

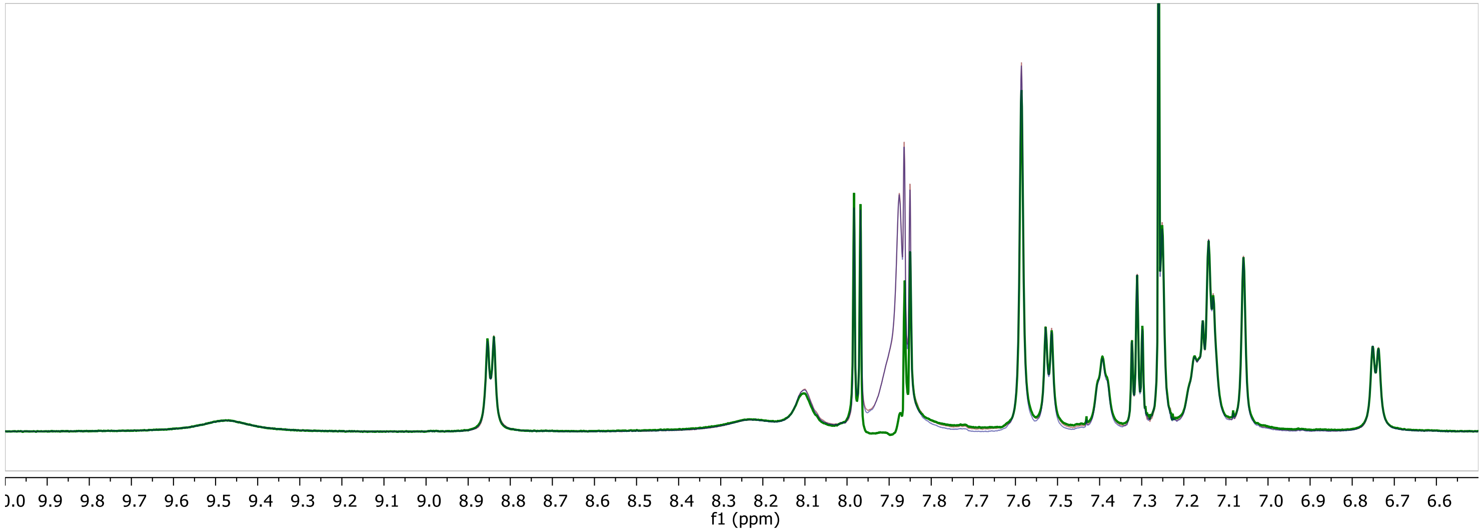

Irradiation at 6.75 ppm.

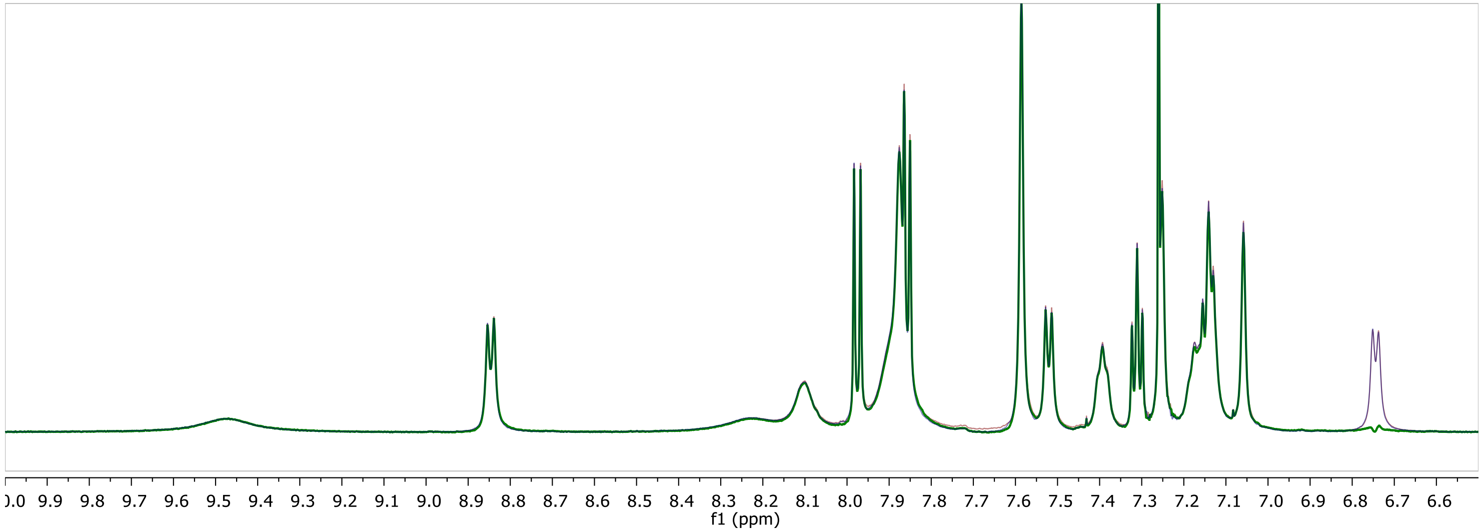

^1H-^{15}N NOEs at 5 Hz rf

O1P (ppm)	20	9.48	8.85[a]	8.23	7.90	6.75	3.07	1.53	1.18	0.38	-20
	off-resonance control	**H22**	H12	**H20**	**H21**, H10, H3, H13	H7	H17, H20	H21	H19	H18	off-resonance control
Δ*I*	0%	-7%	0%	-6%	-7%	0%	0%	0%	0%	0%	0%

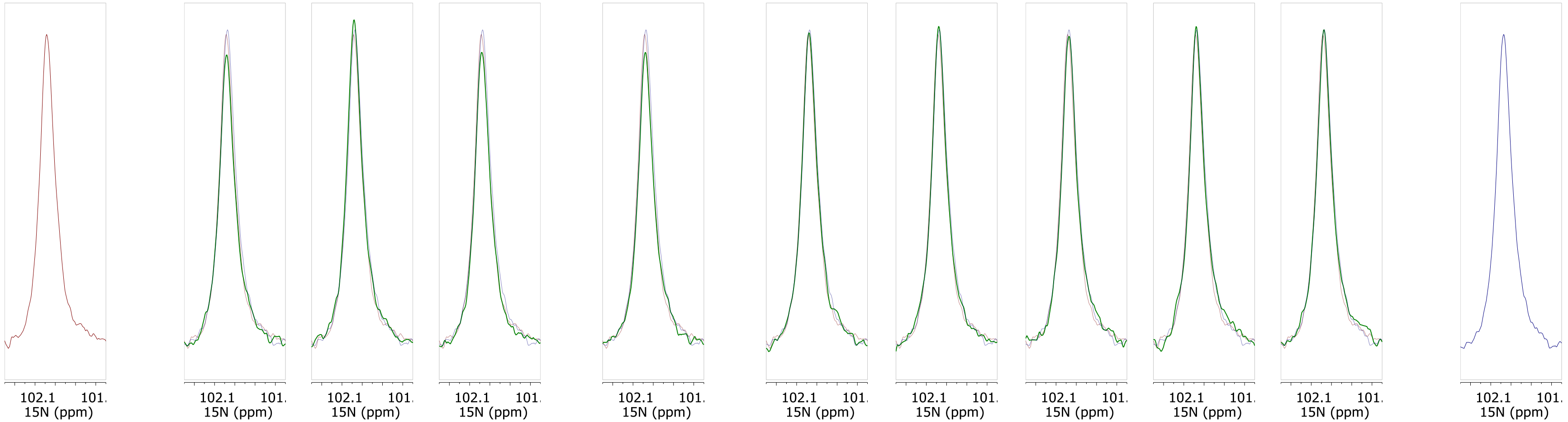

Figure S13 Change in ^{15}N signal intensity (ΔI) caused by irradiation at indicated chemical shift (δH). The ^1H rf field strength (B_1) was 5 Hz and the presaturation time was 40 s.

Variable temperature studies of (*S*)-1k·[1-^{15}N]N_3·Bu_4N

The previously prepared 25 mM sample of (*S*)-1k·[1-^{15}N]N_3·Bu_4N in $CDCl_3$ was studied.

VT studies were conducted on a Bruker AVANCE III 500 MHz spectrometer.

A ^{1}H spectra was taken at 298 K, then the temperature was reduced from 283 K to 213 K in 10 K decrements, and a ^{1}H spectra was acquired at every decrement.

At 213 K, decoalescence of an additional minor species was observed. The major species was designated the 1:1 [(*S*)-**1k**·[1-^{15}N]N_3]·TBA complex, and the minor species was tentatively designated as a 2:1 {[(*S*)-**1k**]$_2$·[1-^{15}N]N_3}·TBA complex, supported by the titration data. The presence of this species was suppressed in the sample of (*S*)-**1k** containing excess azide, further supporting this hypothesis.

^{1}H NMR (500 MHz, $CDCl_3$)

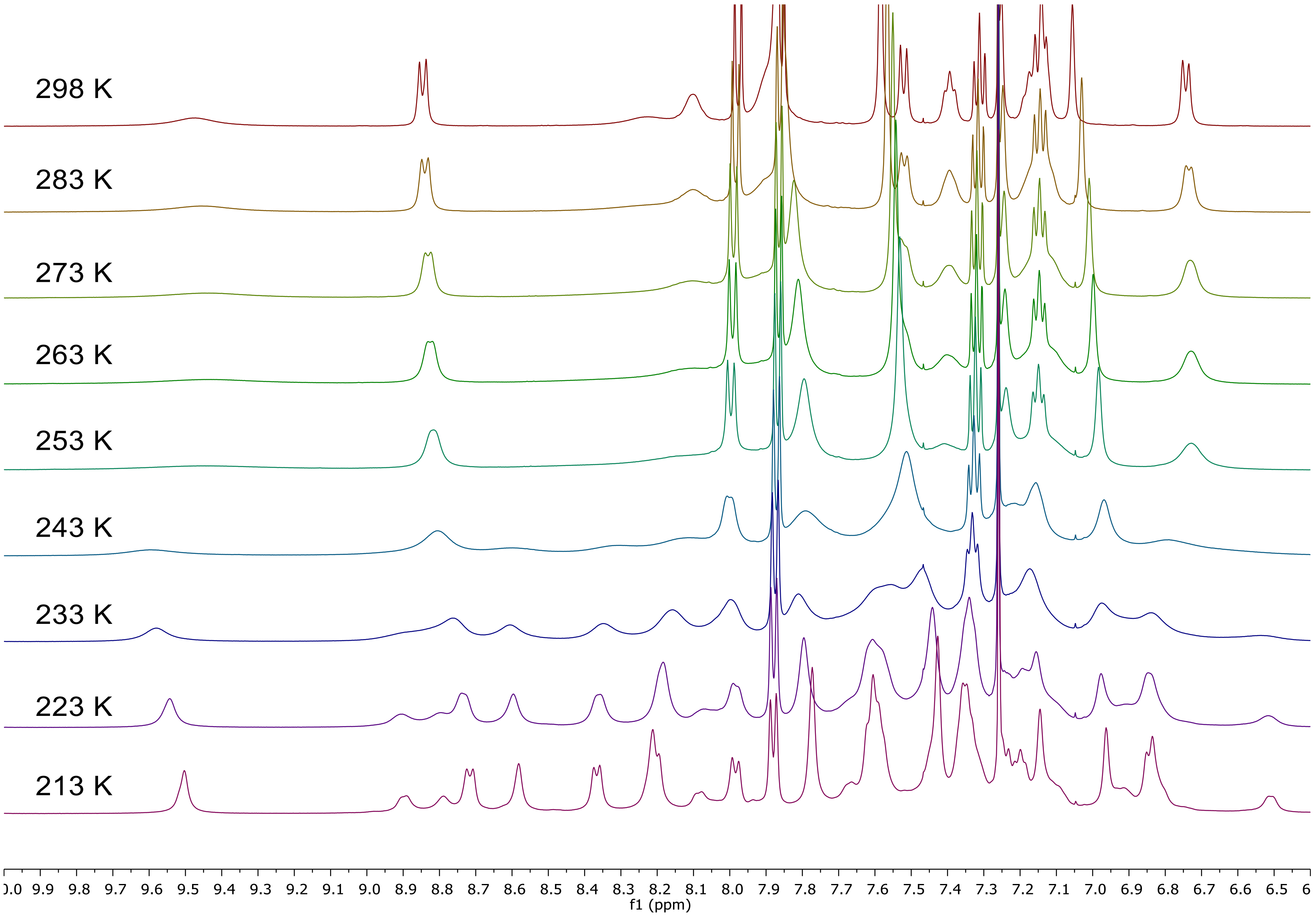

Variable temperature studies of (*S*)-1k with excess tetrabutylammonium [1-^{15}N]azide

$CDCl_3$ was further dried over activated 4 Å molecular sieves. The sample was prepared by weighing tetrabutylammonium [1-^{15}N]azide (10.4 mg, 2 equiv) and (*S*)-**1k** (15.2 mg, 1 equiv) into a flame dried NMR tube equipped with a J. Young valve under N_2. Dry $CDCl_3$ (0.73 mL, 0.25 mM) was added, and the sample was frozen with liquid N_2, evacuated, and flushed with N_2 once.

VT studies were conducted on a Bruker AVANCE III 500 MHz spectrometer.

A ^{1}H spectra was taken at 298 K, then the temperature was reduced from 283 K to 213 K in 10 K decrements, and a ^{1}H spectra was acquired at every decrement.

A significantly reduced quantity of the previously observed minor species at 213 K was observed, supporting the hypothesis those signals corresponded to a 2:1 {[(*S*)-**1k**]$_2$·[1-^{15}N]N_3}·TBA complex.

The assignment of the ^{1}H shifts of [(*S*)-**1k**·[1-^{15}N]N_3]·TBA in $CDCl_3$ at 213 K was achieved by tracking ^{1}H signals along the titration and temperature change, with additional assistance from 2D ^{1}H-^{1}H COSY and ^{1}H-^{1}H NOESY spectra, and supported the assignment at rt.

$^{1h}J_{HN}$ coupling constants was studied by 1D ^{1}H-^{15}N HMBC.

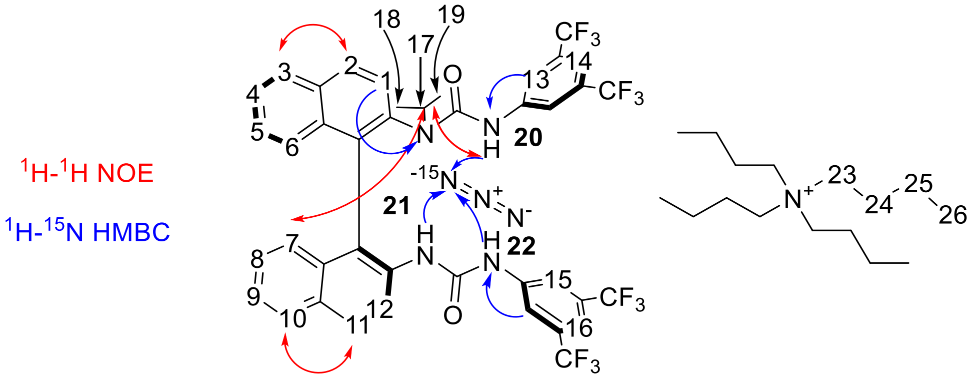

^{1}H NMR (500 MHz, $CDCl_3$, 213 K) δ 9.43 (s, 1H, **H22**), 8.69 (d, *J* = 9.0 Hz, 1H, **H12**), 8.55 (s, 1H, **H20**), 8.37 (d, *J* = 8.5 Hz, 1H, **H2**), 8.28 (s, 1H, **H21**), 8.22 (d, *J* = 8.5 Hz, 1H, **H3**), 7.98 (d, *J* = 9.0 Hz, 1H, **H11**), 7.87 (d, *J* = 8.0 Hz, 1H, **H10**), 7.73 (s, 2H, **H13**), 7.64 – 7.55 (m, 2H, **H4, H1**), 7.41 (s, 2H, **H15**), 7.39 – 7.32 (m, 2H, **H5, H9**), 7.26 – 7.22 (m, 1H, **H6**), 7.22 – 7.16 (m, 1H, **H8**), 7.12 (s, 1H, **H16**), 6.93 (s, 1H, **H14**), 6.84 (d, *J* = 8.5 Hz, 1H, **H7**), 3.22 – 3.13 (m, 16H, **H23**), 3.02 – 2.94 (m, 1H, **H17**), 1.57 (s, 16H, **H24**), 1.41 – 1.27 (m, 16H, **H25**), 1.21 (d, *J* = 6.5 Hz, 1H, **H19**), 0.96 (t, *J* = 7.0 Hz, 24H, **H26**), 0.27 (d, *J* = 6.5 Hz, 1H, **H18**).

^{1}H NMR (500 MHz, $CDCl_3$)

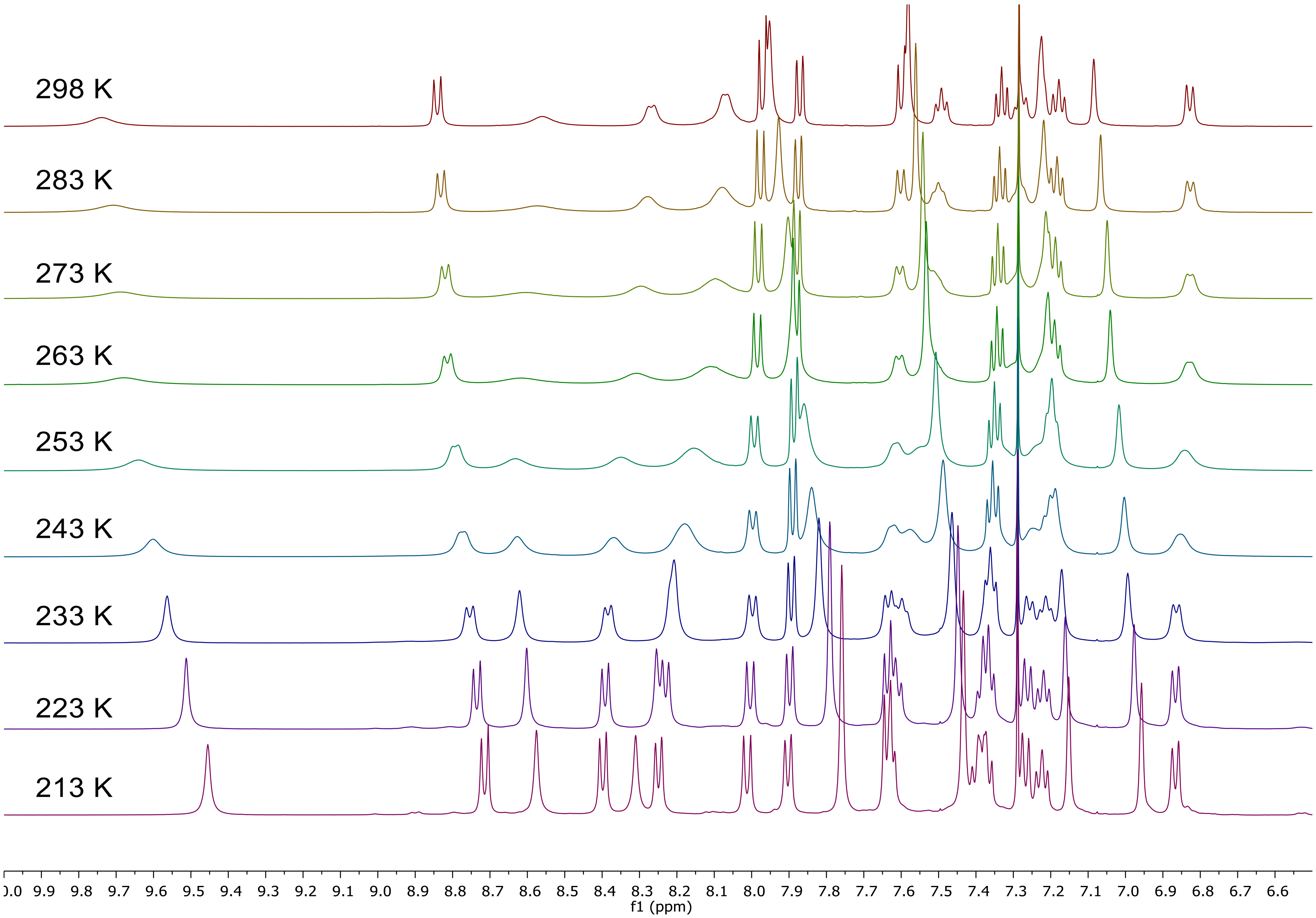

^{1}H NMR (500 MHz, $CDCl_3$, 213 K)

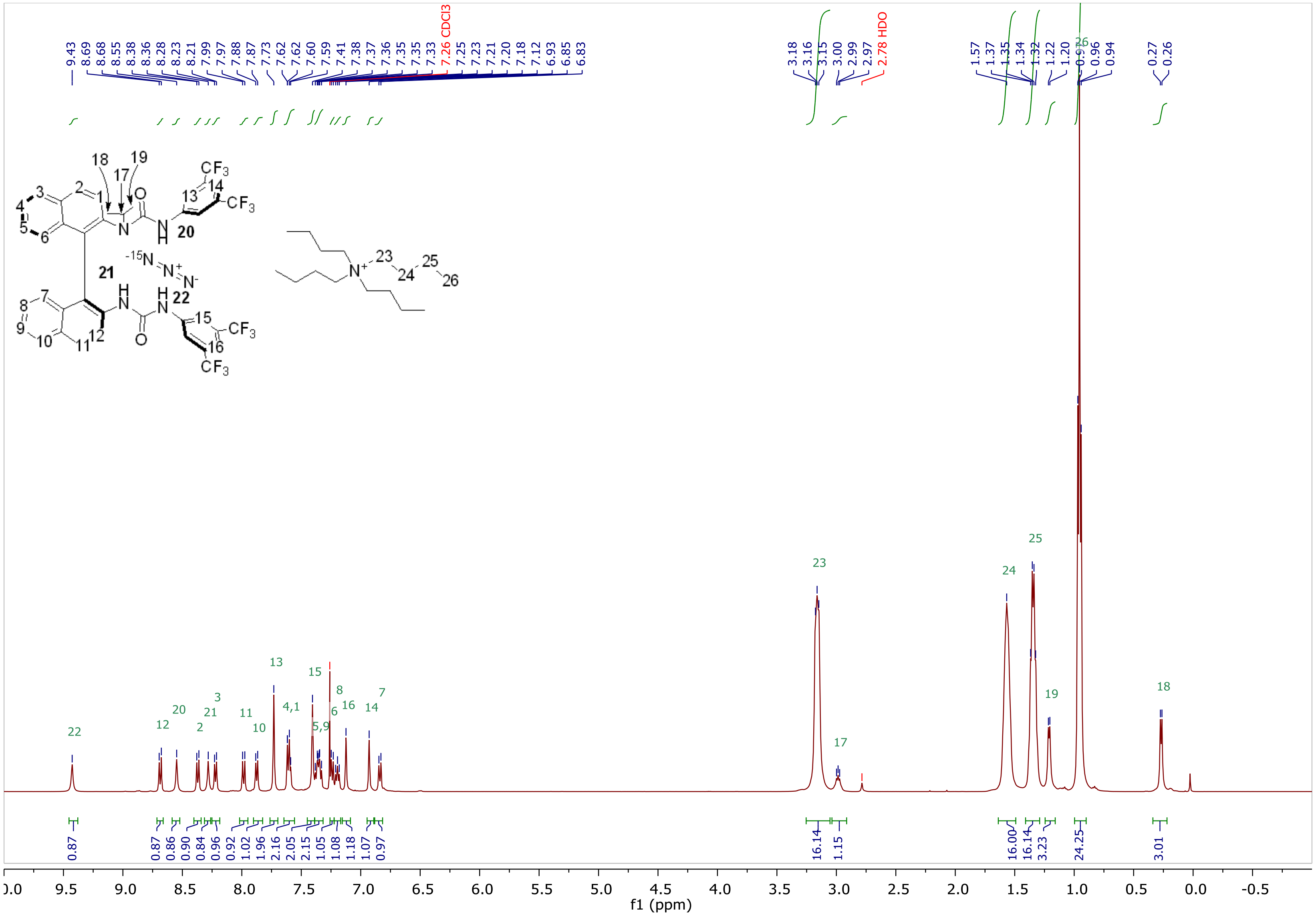

Expansion of above ^{1}H NMR

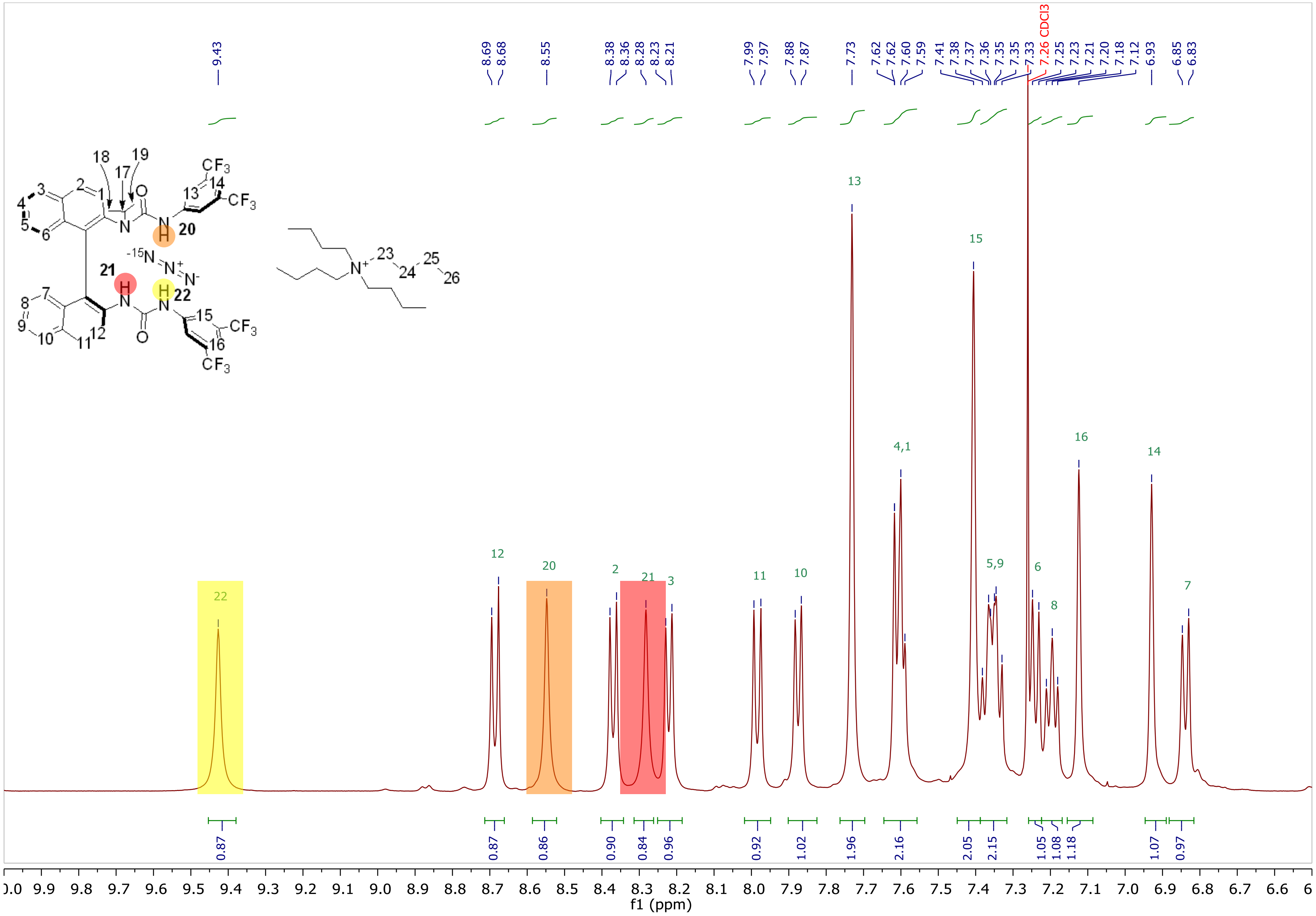

^{1}H-^{1}H **COSY** (500 MHz, $CDCl_3$, 213 K)

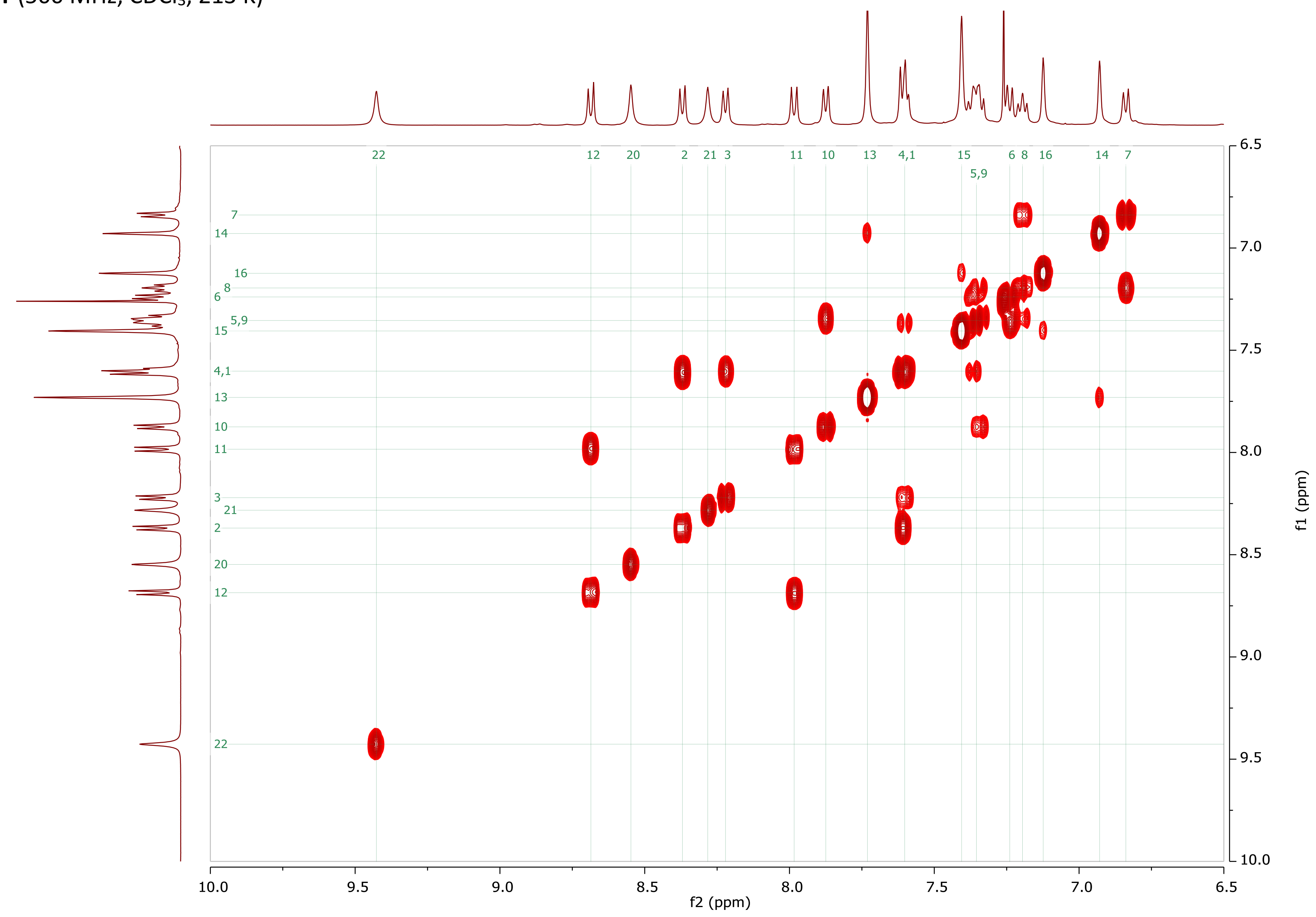

$^{1}H-^{1}H$ **NOESY** (500 MHz, $CDCl_3$, 213 K)

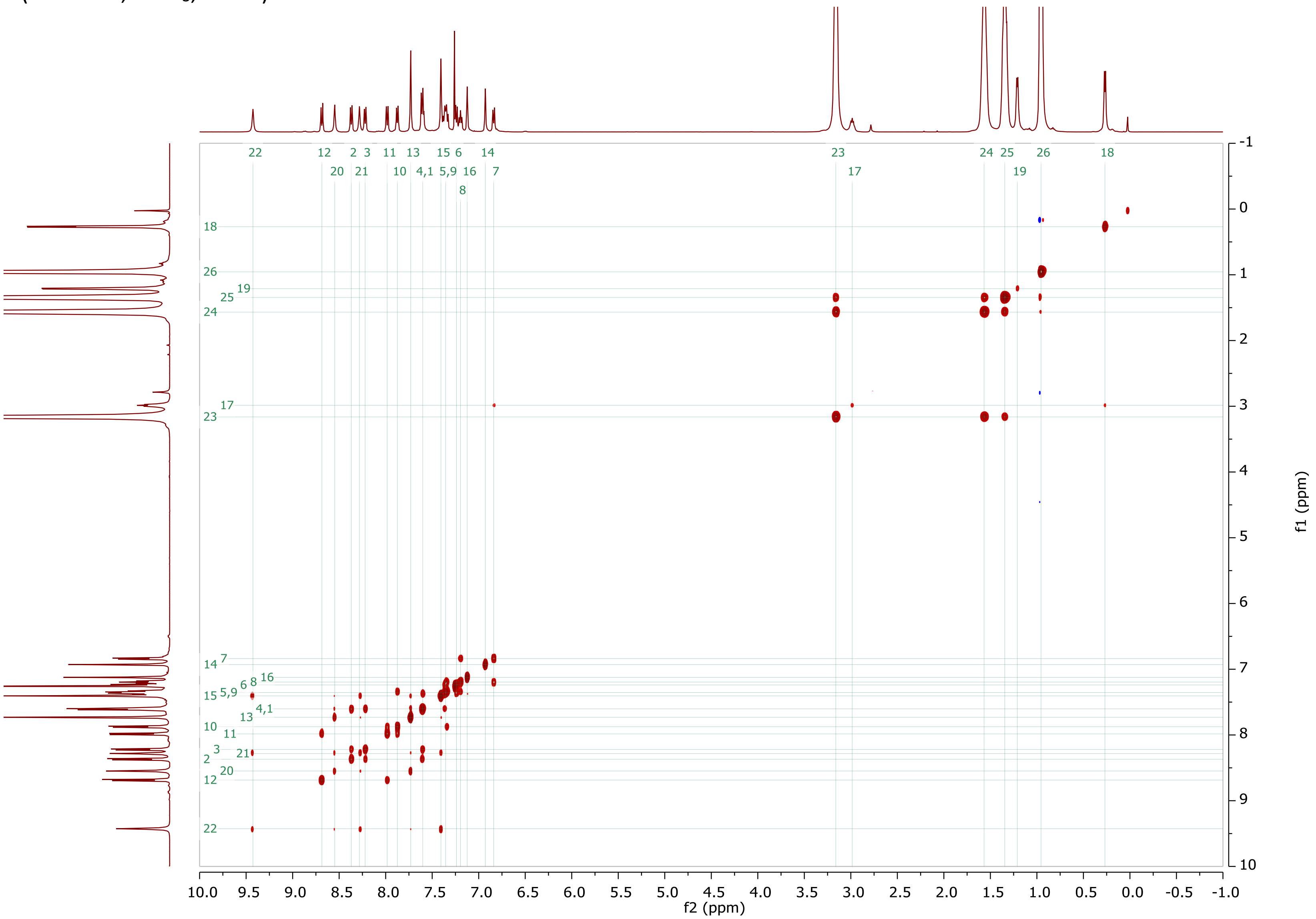

Expansion of above **^{1}H-^{1}H NOESY**

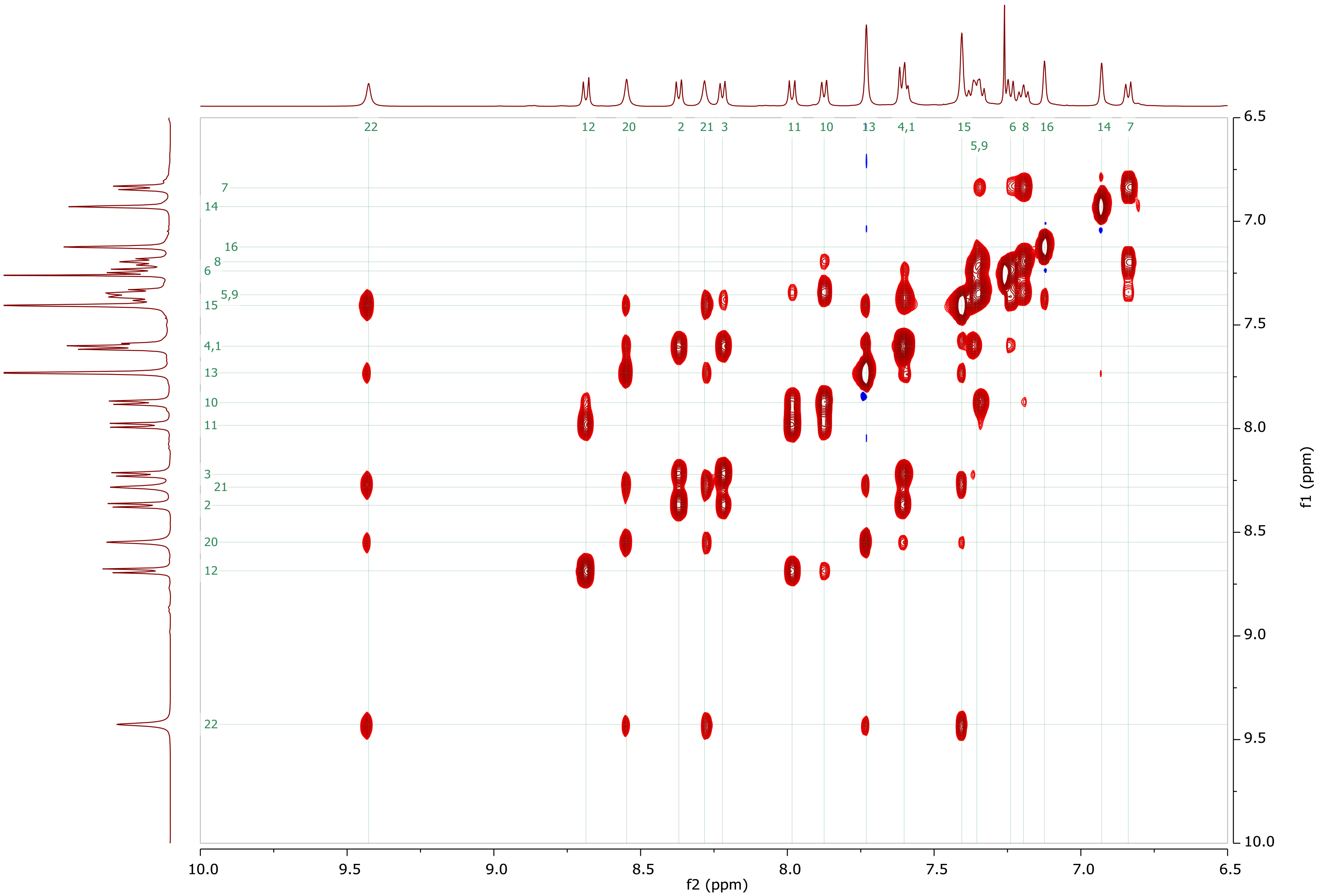

Superimposed ^{1}H-^{15}N **HSQC** (red) and ^{1}H-^{15}N **HMBC** (green) ($CDCl_3$, 213 K)

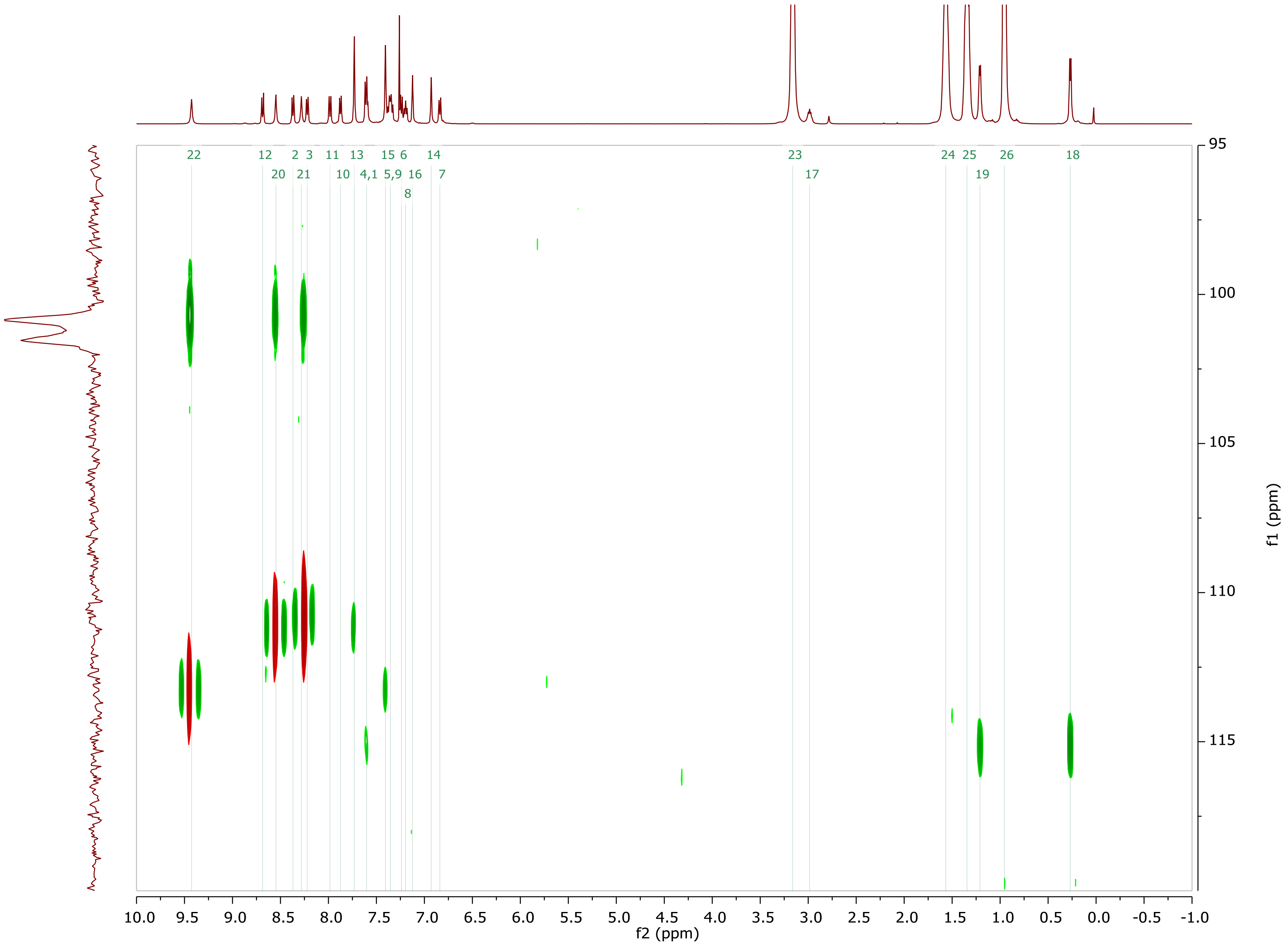

^{1}H-^{15}N 1D HMBC

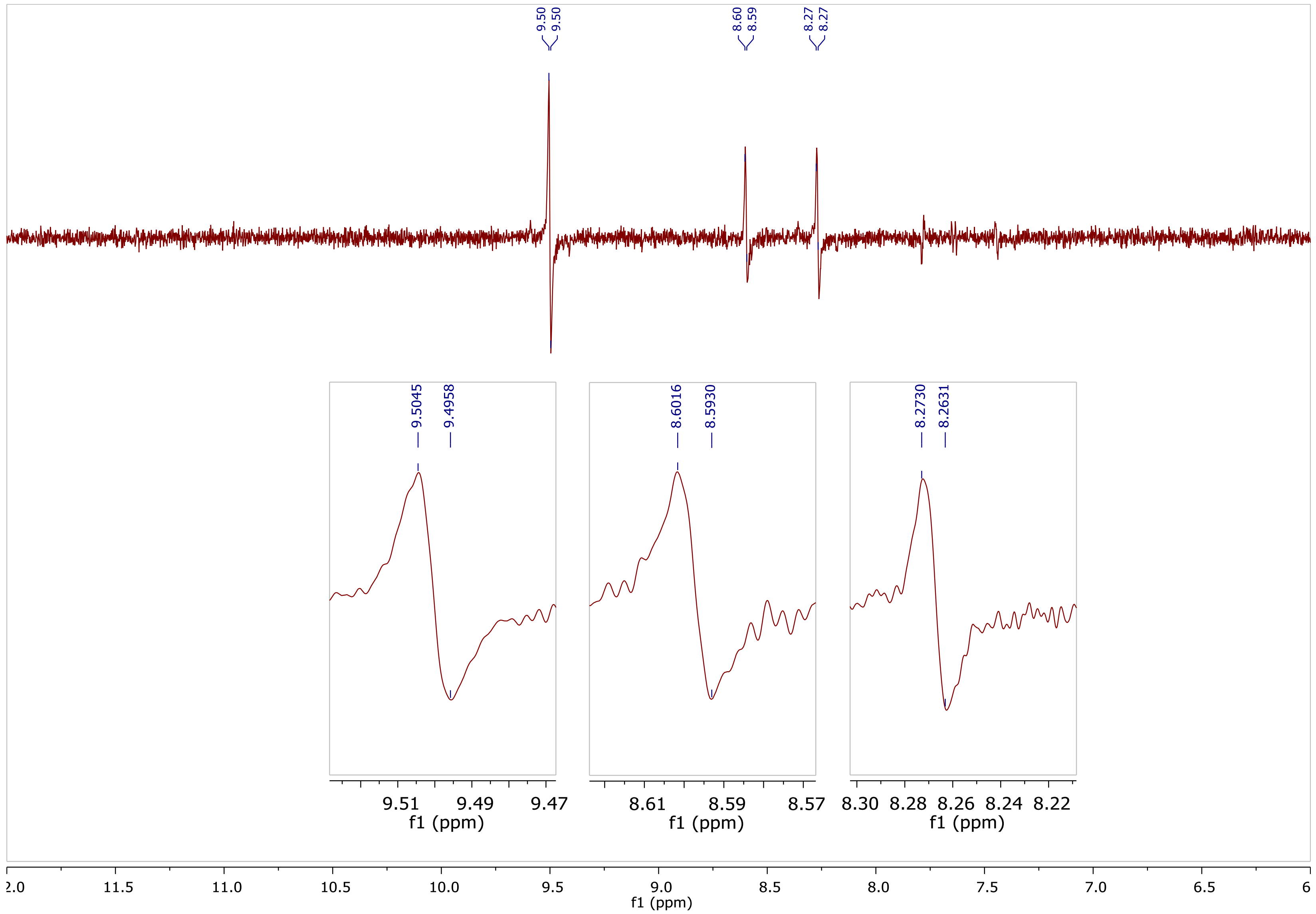

General procedures for enantioselective azidation

(*S*)-**1k-m** were prepared according to reported literature procedures.[8]

General procedure 3 (GP3, synthesis of racemic reference samples)

1,2-difluorobenzene (0.40 mL, 0.25 M) was added to a 1.75 mL vial charged sequentially with substrate (0.10 mmol, 1.00 equiv), **1a** (4.8 mg, 0.01 mmol, 0.10 equiv), and sodium azide (7.8 mg, 1.20 mmol, 1.20 equiv). The reaction mixture was stirred at rt and 1200 rpm for 24 h. The reaction mixture was purified by flash silica chromatography.

NB: Reference for **3s** required 50 °C.

General procedure 4 (GP4, reaction optimization)

1,2-difluorobenzene (0.40 mL, 0.25 M) was added to a 1.75 mL vial charged sequentially with **2a** (30 mg, 0.10 mmol, 1.00 equiv), catalyst, and azide source. The suspension was stirred at the indicated temperature at 1200 rpm for the indicated time. The reaction mixture was applied directly to a silica column and purified by flash silica chromatography (0 to 10% Et_2O in pentane) to afford **3a**. **HPLC** DAICEL CHIRALPAK® IB-3, 0.1% $BuNH_2$ and 0.9% IPA in heptane, 1 mL min^{-1}, t_1 = 3.23, t_2 = 3.73.

General procedure 5 (GP5, synthesis of β-amino alcohols)

The amine and epoxide (1.00 equiv) were stirred in a pressure tube at 100 °C for the indicated time. The reaction mixture was concentrated to afford crude product. The crude product was purified as indicated.

General procedure 6 (GP6, synthesis of β-amino chlorides)

Methanesulfonyl chloride (1.50 equiv) was added dropwise to a solution of the β-amino alcohol (1.00 equiv) and triethylamine (1.50 equiv) in CH_2Cl_2 (0.2 M) at 0 °C under N_2. The reaction mixture was stirred at rt for 2 h. The reaction mixture was washed with sat. $NaHCO_3$ and sat. brine, dried with $MgSO_4$, filtered, and concentrated to afford crude product. The crude product was purified as indicated. All β-amino chlorides were stored in the freezer.

NB: Degradation of β-amino chlorides was observed upon prolonged exposure to silica.

General procedure 7 (GP7, synthesis of enantioenriched β-amino azides)

Standard scale: A 2 mL Schlenk tube (7 mm internal diameter) was charged sequentially with substrate (0.20 mmol, 1.00 equiv), (*S*)-**1k** (16.7 mg, 0.02 mmol, 0.10 equiv), and sodium azide (31.2 mg, 0.48 mmol, 2.40 equiv) under air. 1,2-difluorobenzene (0.8 mL, 0.25 M) was added to the Schlenk tube. The suspension was stirred at the indicated temperature at 1200 rpm for 72 h. The reaction mixture was diluted with Et_2O (2 mL) and washed with H_2O (2 mL). **Aqueous washings containing the excess azide were quenched according to the method described *vida supra*.** The

organic layer was dried with $MgSO_4$, filtered, and concentrated to afford crude product. The crude product was purified as indicated.

Reported yields are the mean of two independent reactions.

Reaction screening/optimization

Reaction optimization used GP4.

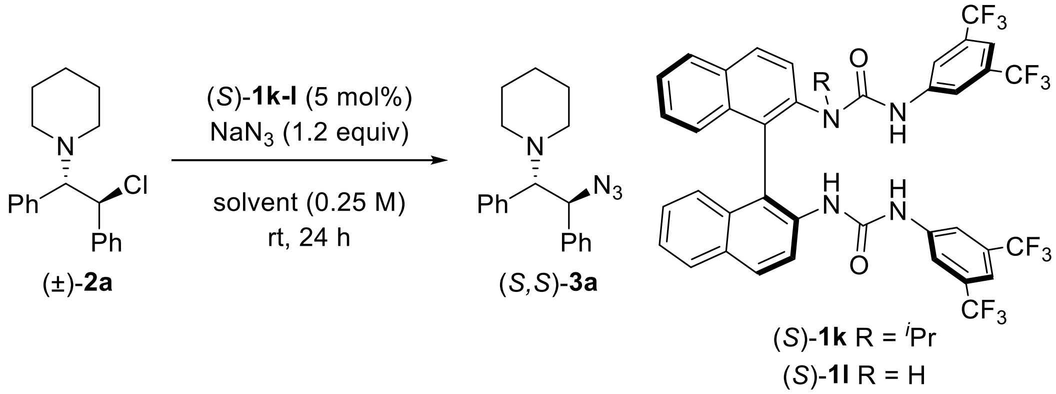

entry	R	solvent	yield	e.r.
1	**H**	CH_2Cl_2	97%	57:43
2	**H**	1,2-difluorobenzene	80%	58:42
3	**H**	PhMe	83%	59:41
4	**H**	MeCN	69%	50:50
5	**^{i}Pr**	CH_2Cl_2	91%	81:19
6	**^{i}Pr**	1,2-difluorobenzene	80%	85:15
7	**^{i}Pr**	PhMe	83%	81:19
8	**^{i}Pr**	MeCN	83%	52:48
9	**^{i}Pr**	$CHCl_3$	86%	77:23

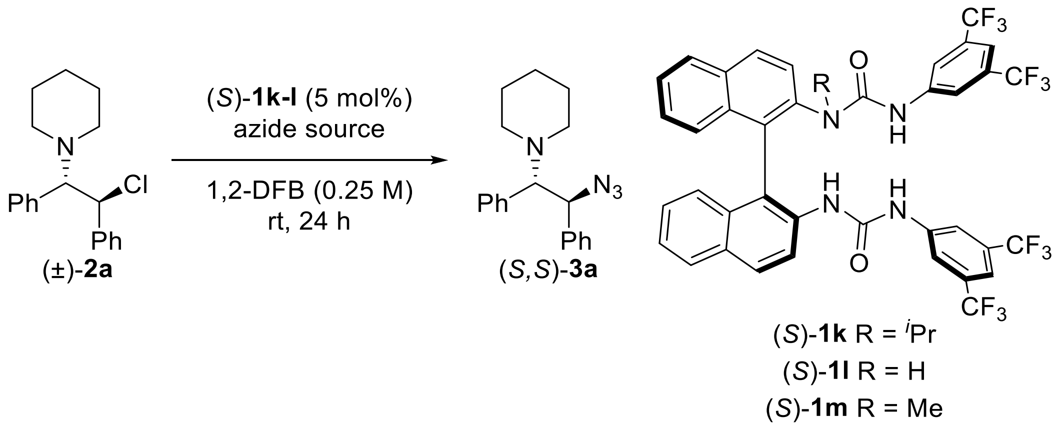

entry	cat.	azide source	temp	yield	e.r.
1	(*S*)-**1l**	NaN_3 (1.2 equiv)	rt	74%	58:42
2	(*S*)-**1m**	NaN_3 (1.2 equiv)	rt	80%	83:17
3	(*S*)-**1k**	NaN_3 (1.2 equiv)	rt	80%	85:15
**4**[a]	(*S*)-**1k**	**NaN_3 (2.4 equiv)**	**-20 °C**	**74%**	**93.5:6.5**
6	(*S*)-**1k**	$Bu_4N{\cdot}N_3$ (1.2 equiv)	rt	90%	50:50

[a]0.1 equiv (*S*)-**1k**, 72 h. 1,2-DFB = 1,2-difluorobenzene.

Synthesis of substrates

Substrates **2a**, **2d**, **2e**, **2f**, **2h**, and **2j** were prepared according to reported literature procedures.[15]

(±)-2-(4-methoxypiperidin-1-yl)-1,2-diphenylethan-1-ol

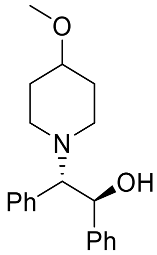

Prepared according to GP5 (48 h) with *cis*-stilbene oxide (2.00 g, 10.19 mmol, 1.00 equiv) and 4-methoxy-piperidine (5.87 g, 50.96 mmol, 5.00 equiv). The crude product was purified by flash silica chromatography (elution gradient 0 to 20% Et_2O in pentane) to afford the title compound as a white solid (2.96 g, 9.50 mmol, 93%).

mp 115-117 °C; ν_{max} (thin film) /cm^{-1} 2926, 2360, 1452, 1084, 701; **^{1}H NMR** (400 MHz, $CDCl_3$) δ 7.26 – 7.18 (m, 5H), 7.17 – 7.05 (m, 5H), 5.19 (s, 1H), 5.02 (d, *J* = 10.5 Hz, 1H), 3.58 (d, *J* = 10.5 Hz, 1H), 3.27 (s, 3H), 3.08 (quin, *J* = 4.5 Hz, 1H), 3.03 – 2.91 (m, 1H), 2.76 – 2.62 (m, 1H), 2.41 (t, *J* = 10.5 Hz, 1H), 2.05 – 1.92 (m, 3H), 1.75 – 1.57 (m, 2H); **^{13}C NMR** (101 MHz, $CDCl_3$) δ 141.6, 133.3, 130.0, 128.1, 128.0, 127.9, 127.5, 127.4, 76.6, 70.7, 55.7, 45.3, 31.8, 31.5; **HRMS** (ESI^+) calc. for $C_{20}H_{26}O_2N$ ($[M+H]^+$): 312.1958; found: 321.1958.

NB: Two fewer ^{13}C resonances observed due to overlapping signals from diastereotopic carbons.

(±)-1-(2-chloro-1,2-diphenylethyl)-4-methoxypiperidine (2b)

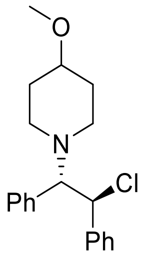

Prepared according to GP6 with (±)-2-(4-methoxypiperidin-1-yl)-1,2-diphenylethan-1-ol (2.90 g, 9.31 mmol, 1.00 equiv). The crude product was purified by flash silica chromatography (elution gradient 0 to 20% Et_2O in pentane) to afford **2b** as a white solid (2.15 g, 6.52 mmol, 70%).

mp 97-99 °C; ν_{max} (thin film) /cm^{-1} 2940, 2819, 2361, 1452, 1139, 1090, 697; **^{1}H NMR** (400 MHz, $CDCl_3$) δ 7.23 – 7.06 (m, 8H), 6.97 – 6.92 (m, 2H), 5.37 (d, *J* = 10.5 Hz, 1H), 4.08 (d, *J* = 10.5 Hz, 1H), 3.28 (s, 3H), 3.07 (tt, *J* = 8.5, 4.5 Hz, 1H), 2.97 – 2.88 (m, 1H), 2.80 – 2.71 (m, 1H), 2.23 (td, *J* = 10.5, 3.0 Hz, 1H), 2.10 (td, *J* = 10.5, 3.0 Hz, 1H), 2.03 – 1.85 (m, 2H), 1.77 – 1.56 (m, 2H); **^{13}C NMR** (101

MHz, $CDCl_3$) δ 140.0, 135.0, 129.2, 128.3, 128.2, 128.0, 127.9, 127.4, 76.9, 74.9, 63.1, 55.6, 48.9, 45.4, 31.6, 31.4; **HRMS** (ESI^+) calc. for $C_{20}H_{25}ON^{35}Cl$ ($[M+H]^+$): 330.1619; found: 330.1618.

(±)-2-(4,4-difluoropiperidin-1-yl)-1,2-diphenylethan-1-ol

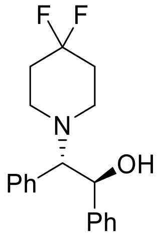

cis-Stilbene oxide (1.00 g, 5.10 mmol, 1.00 equiv) was added to a mixture of 4,4-difluoropiperidine hydrochloride (2.41 g, 15.29 mmol, 3.00 equiv) and NaOH (1.22 g, 30.57 mmol, 6.00 equiv) in ^{i}PrOH (12.75 mL) and H_2O (12.75 mL). The reaction mixture was stirred at 90 °C in a sealed tube for 60 h. The reaction mixture was cooled to rt, diluted with EtOAc (50 mL), washed with H_2O (25 mL), sat. brine (25 mL), dried with $MgSO_4$, filtered, and concentrated to afford crude product. The crude product was purified by flash silica chromatography (elution gradient 0 to 20% Et_2O in pentane) to afford the title compound as a white solid (1.26 g, 3.97 mmol, 78%).

mp 123-124 °C; $\mathbf{\nu_{max}}$ (thin film) /cm^{-1} 2845, 1366, 1155, 1129, 1072, 1005, 701; ^{1}**H NMR** (500 MHz, DMSO-d_6) δ 7.29 – 7.24 (m, 2H), 7.24 – 7.11 (m, 7H), 7.11 – 7.05 (m, 1H), 5.14 (d, *J* = 10.0 Hz, 1H), 4.90 (d, *J* = 1.0 Hz, 1H), 3.80 (d, *J* = 10.0 Hz, 1H), 2.66 (br s, 2H), 2.37 (br s, 2H), 2.16 – 1.94 (m, 4H); ^{19}**F NMR** (471 MHz, DMSO-d_6) δ -95.32 (br s); ^{13}**C NMR** (126 MHz, DMSO-d_6) δ 142.3, 133.8, 129.6, 127.8, 127.6×2, 127.2, 127.0, 122.8 (t, *J* = 241.0 Hz), 74.2, 70.3, 45.5, 33.7 (t, *J* = 22.0 Hz); **HRMS** (ESI^+) calc. for $C_{19}H_{22}ONF_2$ ($[M+H]^+$): 318.1664; found: 318.1662.

(±)-1-(2-chloro-1,2-diphenlethyl)-4,4-difluoropiperidine (2c)

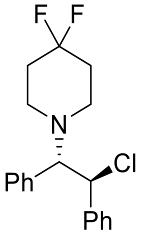

Prepared according to GP6 with (±)-2-(4,4-difluoropiperidin-1-yl)-1,2-diphenylethan-1-ol (1.18 g, 3.72 mmol, 1.00 equiv). The crude product was purified by flash silica chromatography (elution gradient 0 to 10% Et_2O in pentane) to afford **2c** as a white solid (1.16 g, 3.45 mmol, 93%).

mp 120-121 °C; $\mathbf{\nu_{max}}$ (thin film) /cm^{-1} 2832, 2361, 1453, 1362, 1125, 1081, 946, 697; ^{1}**H NMR** (400 MHz, $CDCl_3$) δ 7.25 – 7.08 (m, 8H), 7.03 – 6.89 (m, 2H), 5.36 (d, *J* = 11.0 Hz, 1H), 4.13 (d, *J* = 11.0 Hz, 1H), 2.78 – 2.61 (m, 2H), 2.61 – 2.42 (m, 2H), 2.22 – 1.92 (m, 4H); ^{19}**F NMR** (377 MHz, $CDCl_3$) δ -97.34 (br s); ^{13}**C NMR** (101 MHz, $CDCl_3$) δ 139.7, 134.6, 128.9, 128.4, 128.2, 128.2, 128.1, 127.8,

122.3 (t, J = 241.5 Hz), 74.5, 63.1, 40.0, 34.6 (t, J = 22.5 Hz); **HRMS** (ESI$^+$) calc. for $C_{19}H_{21}N^{35}ClF_2$ ([M+H]$^+$): 336.1325; found: 336.1324.

(±)-1,2-diphenyl-2-(4-phenylpiperazin-1-yl)ethan-1-ol

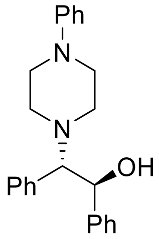

Prepared according to GP5 (48 h) with *cis*-stilbene oxide (1.00 g, 5.10 mmol, 1.00 equiv) and 1-phenylpiperazine (3.89 g, 25.50 mmol, 5.00 equiv). The crude product was purified by flash silica chromatography (elution gradient 0 to 30% Et_2O in pentane) to afford the title compound as a white solid (1.46 g, 4.07 mmol, 80%).

mp 174-176 °C; $\mathbf{v_{max}}$ (thin film) /cm^{-1} 2836, 1599, 1239, 761, 749, 701, 690; **^{1}H NMR** (400 MHz, $CDCl_3$) δ 7.20 – 6.99 (m, 12H), 6.84 – 6.73 (m, 3H), 5.53 – 4.75 (m, 2H), 3.58 (d, J = 10.5 Hz, 1H), 3.25 – 3.08 (m, 4H), 2.81 – 2.71 (m, 2H), 2.53 – 2.42 (m, 2H); **^{13}C NMR** (101 MHz, $CDCl_3$) δ 151.3, 141.3, 132.8, 130.0, 129.2, 128.1×2, 128.0, 127.6, 127.4, 120.2, 116.4, 76.5, 70.6, 49.9, 49.1; **HRMS** (ESI$^+$) calc. for $C_{24}H_{27}ON_2$ ([M+H]$^+$): 359.2129; found: 359.2120.

(±)-1-(2-chloro-1,2-diphenylethyl)-4-phenylpiperazine (2g)

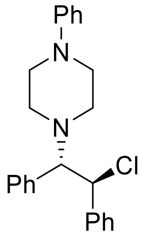

Prepared according to GP6 with (±)-1,2-diphenyl-2-(4-phenylpiperazin-1-yl)ethan-1-ol (1.42 g, 3.96 mmol, 1.00 equiv). The crude product was purified by flash silica chromatography (elution gradient 0 to 20% EtOAc in CH_2Cl_2) to afford **2g** as white solid (1.10 g, 3.96 mmol, 74%).

mp 135-137 °C; $\mathbf{v_{max}}$ (thin film) /cm^{-1} 2824, 2361, 1598, 1233, 694; **^{1}H NMR** (400 MHz, $CDCl_3$) δ 7.21 – 7.13 (m, 4H), 7.13 – 6.99 (m, 6H), 6.96 – 6.86 (m, 2H), 6.87 – 6.79 (m, 2H), 6.79 – 6.68 (m, 1H), 5.36 (d, J = 10.5 Hz, 1H), 4.07 (d, J = 10.5 Hz, 1H), 3.25 – 3.08 (m, 4H), 2.73 – 2.50 (m, 4H); **^{13}C NMR** (101 MHz, $CDCl_3$) δ 151.6, 139.7, 134.5, 129.3, 129.2, 128.4, 128.2, 128.1, 128.0, 127.6, 119.9, 116.4, 75.0, 62.7, 49.8, 49.3; **HRMS** (ESI$^+$) calc. for $C_{24}H_{26}N_2{}^{35}Cl$ ([M+H]$^+$): 377.1779; found: 377.1782.

(±)-2-(benzyl(methyl)amino)-1,2-diphenylethan-1-ol

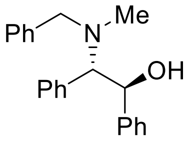

Yttrium(III) trifluoromethanesulfonate (1.09 g, 2.04 mmol, 0.20 equiv) was added to a solution of *N*-benzylmethylamine (1.32 mL, 10.19 mmol, 1.00 equiv) and *cis*-stilbene oxide (2.00 g, 10.19 mmol, 1.00 equiv) in dry THF (20 mL) under N_2. The reaction mixture was heated under reflux for 18 h. The reaction mixture was cooled to rt, diluted with Et_2O (50 mL), washed with sat. $NaHCO_3$ (50 mL), sat. brine (50 mL), dried with $MgSO_4$, filtered, and concentrated to afford crude product. The crude product was purified by flash silica chromatography (elution gradient 0 to 50% Et_2O in pentane) to afford the title compound as a white solid (2.17 g, 6.84 mmol, 67%).

mp 119-120 °C; **ν_{max}** (thin film) /cm^{-1} 3029, 1494, 1452, 1399, 1079, 1053, 913, 879, 758, 737, 699; **^{1}H NMR** (400 MHz, $CDCl_3$) δ 7.41 – 7.34 (m, 4H), 7.34 – 7.26 (m, 4H), 7.19 – 7.07 (m, 7H), 5.27 (s, 1H), 5.11 (d, *J* = 10.5 Hz, 1H), 3.74 (d, *J* = 10.5 Hz, 1H), 3.65 (d, *J* = 13.0 Hz, 1H), 3.41 (d, *J* = 13.0 Hz, 1H), 2.21 (s, 3H); **^{13}C NMR** (101 MHz, $CDCl_3$) δ 141.5, 138.5, 133.0, 130.3, 129.2, 128.7, 128.1, 128.0, 127.9, 127.5×3, 74.3, 71.2, 58.8, 36.9; **HRMS** (ESI$^+$) calc. for $C_{22}H_{24}ON$ ($[M+H]^+$): 318.1852; found 318.1851.

(±)-*N*-benzyl-2-chloro-*N*-methyl-1,2-diphenylethan-1-amine (2i)

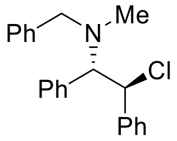

Prepared according to GP6 with (±)-2-(benzyl(methyl)amino)-1,2-diphenylethan-1-ol (2.15 g, 6.77 mmol, 1.00 equiv). The crude product was triturated with CH_2Cl_2. The solids were collected and dried to afford **2i** as white solid (1.17 g, 3.45 mmol, 51%).

mp 156-157 °C; **ν_{max}** (thin film) /cm^{-1} 3028, 2361, 1492, 1452, 761, 703; **^{1}H NMR** (400 MHz, $CDCl_3$) δ 7.52 – 7.45 (m, 2H), 7.39 – 7.33 (m, 2H), 7.31 – 7.26 (m, 1H), 7.24 – 7.06 (m, 8H), 7.01 – 6.95 (m, 2H), 5.49 (d, *J* = 11.0 Hz, 1H), 4.24 (d, *J* = 11.0 Hz, 1H), 3.80 (d, *J* = 13.5 Hz, 1H), 3.33 (d, *J* = 13.5 Hz, 1H), 2.25 (s, 3H); **^{13}C NMR** (101 MHz, $CDCl_3$) δ 140.0, 139.5, 134.3, 129.3, 129.0, 128.4×2, 128.2, 128.1, 128.0, 127.5, 127.1, 73.0, 63.4, 58.0, 37.6; **HRMS** (ESI$^+$) calc. for $C_{22}H_{23}N^{35}Cl$ ($[M+H]^+$): 412.1514; found: 412.1512.

(±)-2-(azepan-1-yl)-1,2-diphenylethan-1-ol

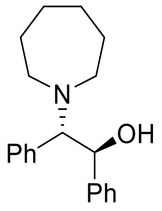

Prepared according to GP5 (72 h) with *cis*-stilbene oxide (2.00 g, 10.19 mmol, 1.00 equiv) and azepane (5.74 mL, 50.96 mmol, 5.00 equiv). The crude product was purified by flash silica chromatography (elution gradient 0 to 20% EtOAc in pentane), then triturated with pentane to afford the title compound as a white solid (2.05 g, 6.94 mmol, 68%).

mp 81-83 °C; **ν_{max}** (thin film) /cm^{-1} 2923, 2852, 1451, 1047, 759, 699; **^{1}H NMR** (400 MHz, $CDCl_3$) δ 7.25 – 7.18 (m, 5H), 7.17 – 7.08 (m, 5H), 5.34 (s, 1H), 4.98 (d, *J* = 10.5 Hz, 1H), 3.64 (d, *J* = 10.5 Hz, 1H), 2.89 – 2.74 (m, 2H), 2.60 – 2.45 (m, 2H), 1.80 – 1.65 (m, 4H), 1.62 – 1.56 (m, 4H); **^{13}C NMR** (101 MHz, $CDCl_3$) δ 141.7, 135.0, 129.8, 128.0×2, 127.7, 127.5, 127.4, 77.6, 71.3, 52.1, 29.4, 26.7; **HRMS** (ESI$^+$) calc. for $C_{20}H_{26}ON$ ([M+H]$^+$): 296.2009; found 296.2007.

(±)-2-chloro-1,2-diphenylethyl)azepane (2k)

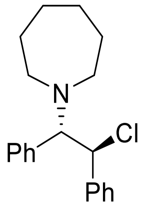

Prepared according to GP6 with (±)-2-(azepan-1-yl)-1,2-diphenylethan-1-ol (2.05 g, 6.94 mmol, 1.00 equiv). The crude product was purified by flash silica chromatography (elution gradient 0 to 20% Et_2O in pentane) to afford **2k** as a white solid (1.47 g, 4.68 mmol, 67%).

mp 77-78 °C; **ν_{max}** (thin film) /cm^{-1} 2924, 2361, 1494, 1451, 1158, 1076, 695; **^{1}H NMR** (400 MHz, $CDCl_3$) δ 7.24 – 6.84 (m, 10H), 5.29 (d, *J* = 10.5 Hz, 1H), 4.08 (d, *J* = 10.5 Hz, 1H), 2.87 – 2.65 (m, 2H), 2.65 – 2.42 (m, 2H), 1.67 – 1.55 (m, 4H), 1.52 – 1.41 (m, 4H); **^{13}C NMR** (101 MHz, $CDCl_3$) δ 140.3, 137.4, 128.7, 128.3, 128.2, 127.9, 127.8, 127.0, 75.5, 64.5, 51.6, 29.7, 27.2; **HRMS** (ESI$^+$) calc. for $C_{20}H_{25}N^{35}Cl$ ([M+H]$^+$): 314.1670; found 314.1668.

(±)-1,2-bis(3-fluorophenyl)-2-(piperidin-1-yl)ethan-1-ol

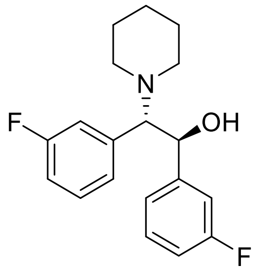

Prepared according to GP5 (30 h) with *cis*-2,3-bis(3-fluorophenyl)oxirane[16] (1.30 g, 5.60 mmol, 1.00 equiv) and piperidine (2.76 mL, 28.00 mmol, 5.00 equiv). The crude product was purified by flash silica chromatography (elution gradient 0 to 20% Et_2O in pentane) to afford the title compound as a white solid (1.42 g, 4.48 mmol, 80%).

mp 108-110 °C; **ν_{max}** (thin film) /cm^{-1} 2936, 1590, 1488, 1445, 1250, 1136, 1157, 780, 732, 706, 695; **^{1}H NMR** (500 MHz, $CDCl_3$) δ 7.23 (td, *J* = 8.0, 6.0 Hz, 1H), 7.09 (td, *J* = 8.0, 6.0 Hz, 1H), 7.00 (dt, *J* = 10.0, 2.0 Hz, 1H), 6.97 – 6.91 (m, 2H), 6.86 (d, *J* = 7.5 Hz, 1H), 6.84 – 6.79 (m, 2H), 5.29 (s, 1H), 4.97 (d, *J* = 10.5 Hz, 1H), 3.47 (d, *J* = 10.5 Hz, 1H), 2.62 (br s, 2H), 2.28 (br s, 2H), 1.70 – 1.59 (m, 4H), 1.37 (br s, 2H); **^{19}F NMR** (471 MHz, $CDCl_3$) δ -113.00, -113.44; **^{13}C NMR** (126 MHz, $CDCl_3$) δ 162.8 (d, *J* = 245.0 Hz), 162.4 (d, *J* = 246.5 Hz), 144.3 (d, *J* = 7.0 Hz), 135.9 (d, *J* = 6.5 Hz), 129.5 (d, *J* = 8.0 Hz), 129.4 (d, *J* = 8.0 Hz), 125.7 (d, *J* = 3.0 Hz), 123.1 (d, *J* = 3.0 Hz), 116.7 (d, *J* = 21.0 Hz), 114.9 (d, *J* = 21.0 Hz), 114.4 (d, *J* = 21.0 Hz), 114.0 (d, *J* = 22.0 Hz), 76.8 (d, *J* = 1.5 Hz), 70.0 (d, *J* = 2.0 Hz), 50.4, 26.6, 24.3; **HRMS** (ESI$^+$) calc. for $C_{19}H_{22}ONF_2$ ([M+H]$^+$): 318.1664 found: 318.1662.

(±)-1-(2-chloro-1,2-bis(3-fluorophenyl)ethyl)piperidine (2l)

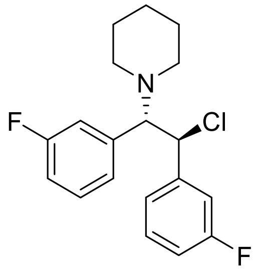

Prepared according to GP6 with (±)-1-(2-chloro-1,2-bis(3-fluorophenyl)ethyl)piperidine (1.36 g, 4.29 mmol, 1.00 equiv). The crude product was purified by flash silica chromatography (elution gradient 0 to 20% Et_2O in pentane) to afford **2l** as a white solid (1.10 g, 3.26 mmol, 76%).

mp 120-122 °C; **ν_{max}** (thin film) /cm^{-1} 2934, 1590, 1488, 1447, 1245, 878, 773; **^{1}H NMR** (500 MHz, $CDCl_3$) δ 7.19 – 7.06 (m, 2H), 7.00 – 6.92 (m, 2H), 6.89 – 6.78 (m, 2H), 6.73 (d, *J* = 7.5 Hz, 1H), 6.67 (d, *J* = 9.5 Hz, 1H), 5.30 (d, *J* = 10.5 Hz, 1H), 3.97 (d, *J* = 10.5 Hz, 1H), 2.56 – 2.43 (m, 2H), 2.42 – 2.27 (m, 2H), 1.74 – 1.63 (m, 2H), 1.63 – 1.52 (m, 2H), 1.41 – 1.31 (m, 2H); **^{19}F NMR** (471 MHz, $CDCl_3$) δ -112.67, -113.13; **^{13}C NMR** (126 MHz, $CDCl_3$) δ 162.6 (d, *J* = 246.5 Hz), 162.4 (d, *J* = 246.0 Hz), 142.2

(d, J = 7.5 Hz), 137.5 (d, J = 6.0 Hz), 129.9 (d, J = 8.5 Hz), 129.3 (d, J = 8.0 Hz), 124.9 (d, J = 3.0 Hz), 124.0 (d, J = 3.0 Hz), 115.9 (d, J = 21.0 Hz), 115.2 (d, J = 21.0 Hz), 115.2 (d, J = 22.5 Hz), 114.5 (d, J = 21.0 Hz), 75.2, 61.6, 50.6, 26.5, 24.7; **HRMS** (ESI$^+$) calc. for $C_{19}H_{21}N^{35}ClF_2$ ([M+H]$^+$): 336.1325 found: 336.1325.

(±)-1,2-bis(3-chlorophenyl)-2-(piperidin-1-yl)ethan-1-ol

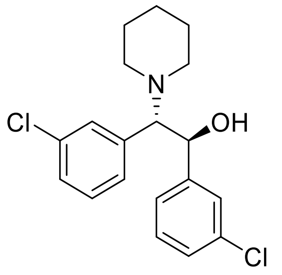

Prepared according to GP5 (48 h) with *cis*-2,3-bis(3-chlorophenyl)oxirane[17] (1.48 g, 7.54 mmol, 1 equiv) and piperidine (3.72 mL, 50.90 mmol, 6.75 equiv). The crude product was purified by flash silica chromatography (elution gradient 0 to 10% Et_2O in pentane) to afford the title compound as a white solid (1.67 g, 4.77 mmol, 63%).

mp 99-101 °C; **ν_{max}** (thin film) /cm^{-1} 2936, 2360, 1596, 1572, 1475, 1429, 1191, 1157, 1055, 883, 733, 695; **^{1}H NMR** (400 MHz, $CDCl_3$) δ 7.34 – 7.29 (m, 1H), 7.26 – 7.18 (m, 2H), 7.13 – 7.02 (m, 3H), 7.00 – 6.92 (m, 2H), 5.27 (s, 1H), 4.94 (d, J = 10.5 Hz, 1H), 3.44 (d, J = 10.5 Hz, 1H), 2.60 (br s, 2H), 2.27 (br s, 2H), 1.76 – 1.59 (m, 4H), 1.37 (br s, 2H); **^{13}C NMR** (101 MHz, $CDCl_3$) δ 143.7, 135.3, 134.2, 134.1, 129.9, 129.3×2, 128.2, 128.0, 127.8, 127.3, 125.8, 76.8, 70.0, 50.3, 26.6, 24.3; **HRMS** (ESI$^+$) calc. for $C_{19}H_{22}ON^{35}Cl_2$ ([M+H]$^+$): 350.1073; found: 350.1076.

(±)-1-(2-chloro-1,2-bis(3-chlorophenyl)ethyl)piperidine (2m)

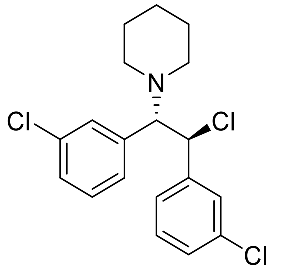

Prepared according to GP6 with (±)-1,2-bis(3-chlorophenyl)-2-(piperidin-1-yl)ethan-1-ol (1.65 g, 4.71 mmol, 1.00 equiv). The crude product was purified by flash silica chromatography (elution gradient 0 to 10% Et_2O in pentane) to afford **2m** as a white solid (1.22 g, 3.30 mmol, 70%).

mp 140-142 °C; **ν_{max}** (thin film) /cm^{-1} 2933, 2360, 1573, 1429, 1098, 1079, 717, 694; **^{1}H NMR** (400 MHz, $CDCl_3$) δ 7.25 – 7.21 (m, 1H), 7.13 – 7.04 (m, 5H), 6.96 – 6.93 (m, 1H), 6.87 – 6.80 (m, 1H), 5.28 (d, J = 10.5 Hz, 1H), 3.94 (d, J = 10.5 Hz, 1H), 2.50 (ddd, J = 11.0, 7.0, 3.5 Hz, 2H), 2.34 (ddd, J = 11.0, 7.0, 3.5 Hz, 2H), 1.72 – 1.56 (m, 4H), 1.42 – 1.32 (m, 2H); **^{13}C NMR** (101 MHz, $CDCl_3$) δ 141.7,

136.9, 134.3, 134.0, 129.7, 129.1×2, 128.4×2, 127.8, 127.3, 126.4, 75.2, 61.5, 50.7, 26.4, 24.7; **HRMS** (ESI$^+$) calc. for $C_{19}H_{21}N^{35}Cl_3$ ([M+H]$^+$): 368.0734; found: 368.0735.

(±)-2-(piperidin-1-yl)-1,2-bis(3-(trifluoromethyl)phenyl)ethan-1-ol

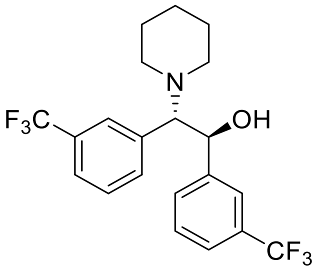

Prepared according to GP5 (48 h) with 2,3-bis(3-(trifluoromethyl)phenyl)oxirane[17] (1.00 g, 3.01 mmol, 1.00 equiv) and piperidine (1.44 mL, 15.05 mmol, 5.00 equiv). The crude product was purified by flash silica chromatography (elution gradient 0 to 20% Et_2O in pentane) to afford the title compound as a pale-yellow oil (0.95 g, 2.29 mmol, 76%).

ν_{max} (thin film) /cm^{-1} 2939, 1446, 1326, 1160, 1120, 1073, 900, 804, 704, 665; **^{1}H NMR** (400 MHz, $CDCl_3$) δ 7.55 – 7.48 (m, 2H), 7.43 – 7.36 (m, 2H), 7.34 – 7.21 (m, 4H), 5.27 (s, 1H), 5.08 (d, *J* = 10.5 Hz, 1H), 3.53 (d, *J* = 10.5 Hz, 1H), 2.63 (br s, 2H), 2.30 (br s, 2H), 1.76 – 1.54 (m, 4H), 1.38 (s, 2H); **^{19}F NMR** (377 MHz, $CDCl_3$) δ -62.72, -62.81; **^{13}C NMR** (101 MHz, $CDCl_3$) δ 142.5, 134.3, 133.3, 130.7, 130.6 (q, *J* = 32.5 Hz), 130.6 (q, *J* = 32.5 Hz), 128.6, 128.6, 126.4 (q, *J* = 4.0 Hz), 125.0 (q, *J* = 4.0 Hz), 124.5 (q, *J* = 4.0 Hz), 124.2 (q, *J* = 272.0 Hz), 124.1 (q, *J* = 272.5 Hz), 124.0 (q, *J* = 4.0 Hz), 77.1, 70.2, 50.4, 26.6, 24.3; **HRMS** (ESI$^+$) calc. for $C_{21}H_{22}ONF_6$ ([M+H]$^+$): 418.1600; found: 418.1589.

(±)-1-(2-chloro-1,2-bis(3-(trifluoromethyl)phenyl)ethyl)piperidine (2n)

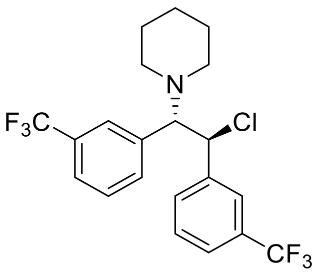

Prepared according to GP6 with (±)-2-(piperidin-1-yl)-1,2-bis(3-(trifluoromethyl)phenyl)ethan-1-ol (902 mg, 2.16 mmol, 1.00 equiv). The crude product was purified by flash silica chromatography (elution gradient 0 to 20% Et_2O in pentane) to afford **2n** as a white solid (687 mg, 1.58 mmol, 73%).

mp 89-90 °C; **ν_{max}** (thin film) /cm^{-1} 2937, 1446, 1327, 1162, 1120, 1073, 700; **^{1}H NMR** (500 MHz, $CDCl_3$) δ 7.46 – 7.37 (m, 4H), 7.35 – 7.27 (m, 2H), 7.18 (br s, 1H), 7.16 – 7.12 (m, 1H), 5.47 (d, *J* = 9.5 Hz, 1H), 4.06 (d, *J* = 9.5 Hz, 1H), 2.63 – 2.46 (m, 2H), 2.46 – 2.37 (m, 2H), 1.76 – 1.61 (m, 4H), 1.48 – 1.34 (m, 2H); **^{19}F NMR** (471 MHz, $CDCl_3$) δ -62.84, -62.96; **^{13}C NMR** (126 MHz, $CDCl_3$) δ

140.4, 136.0, 132.6, 131.4, 130.7 (q, *J* = 32.5 Hz), 130.5 (d, *J* = 32.5 Hz), 128.9, 128.4, 125.7 (q, *J* = 4.0 Hz), 125.2 (q, *J* = 4.0 Hz), 125.0 (d, *J* = 4.0 Hz), 124.5 (q, *J* = 4.0 Hz), 124.1 (q, *J* = 272.5 Hz), 123.8 (q, *J* = 272.5 Hz), 75.8, 61.3, 51.0, 26.4, 24.6; **HRMS** (ESI^+) calc. for $C_{21}H_{21}N^{35}ClF_6$ ($[M+H]^+$): 436.1261; found: 436.1269.

(±)-1,2-bis(4-bromophenyl)-2-(piperidin-1-yl)ethan-1-ol

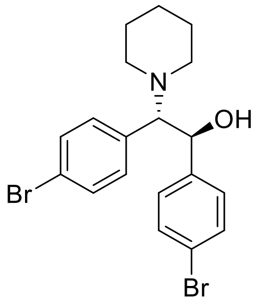

Prepared according to GP5 (48 h) with 2,3-bis(4-bromophenyl)oxirane[18] (2.25 g, 6.36 mmol, 1.00 equiv). The crude product was purified by flash silica chromatography (elution gradient 0 to 15% Et_2O in pentane), then triturated with pentane to afford the title compound as a white solid (1.46 g, 3.32 mmol, 52%).

mp 130-132 °C; $\mathbf{v_{max}}$ (thin film) /cm^{-1} 2934, 2361, 1591, 1488, 1395, 1071, 1010, 908, 871, 822, 728; **^{1}H NMR** (400 MHz, $CDCl_3$) δ 7.40 (d, *J* = 8.5 Hz, 2H), 7.28 (d, *J* = 8.5 Hz, 2H), 7.06 (d, *J* = 8.5 Hz, 2H), 6.93 (d, *J* = 8.5 Hz, 2H), 5.26 (s, 1H), 4.92 (d, *J* = 10.5 Hz, 1H), 3.41 (d, *J* = 10.5 Hz, 1H), 2.58 (br s, 2H), 2.25 (br s, 2H), 1.80 – 1.51 (m, 4H), 1.36 (s, 2H); **^{13}C NMR** (101 MHz, $CDCl_3$) δ 140.7, 132.2, 131.5, 131.3, 131.2, 129.0, 122.1, 121.4, 76.7, 70.0, 50.5, 26.6, 24.3; **HRMS** (ESI^+) calc. for $C_{19}H_{22}ON^{79}Br_2$ ($[M+H]^+$): 438.0063; found: 438.0059.

(±)-1-(1,2-bis(4-bromophenyl)-2-chloroethyl)piperidine (2o)

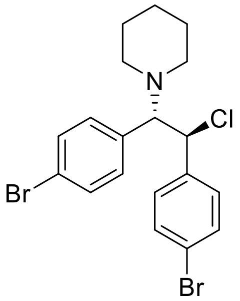

Prepared according to GP6 with (±)-1,2-bis(4-bromophenyl)-2-(piperidin-1-yl)ethan-1-ol (1.62 g, 3.69 mmol, 1.00 equiv). The crude product was purified by flash silica chromatography (elution gradient 0 to 10% Et_2O in pentane) to afford **2o** as a white solid (1.05 g, 2.29 mmol, 62%).

mp 119-120 °C; $\mathbf{v_{max}}$ (thin film) /cm^{-1} 2993, 1488, 1103, 1074, 1010, 750; **^{1}H NMR** (400 MHz, $CDCl_3$) δ 7.34 – 7.27 (m, 4H), 7.10 – 6.98 (m, 2H), 6.87 – 6.75 (m, 2H), 5.29 (d, *J* = 10.5 Hz, 1H), 3.93 (d, *J* = 10.5 Hz, 1H), 2.53 – 2.40 (m, 2H), 2.40 – 2.27 (m, 2H), 1.73 – 1.52 (m, 4H), 1.47 – 1.30 (m, 2H); **^{13}C**

NMR (101 MHz, $CDCl_3$) δ 138.8, 133.8, 131.6, 131.1, 130.7, 129.9, 122.1, 121.5, 75.0, 61.6, 50.6, 26.4, 24.7; **HRMS** (ESI$^+$) calc. for $C_{19}H_{21}N^{79}Br_2{}^{35}Cl$ ([M+H]$^+$): 455.9724; found 455.9722.

(*Z*)-1,2-bis(4-(*tert*-butyl)phenyl)ethene

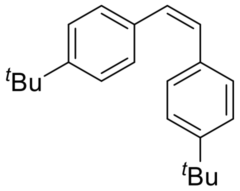

A solution of ^{n}BuLi in hexane (27.5 mL, 1.6 M, 44.08 mmol, 4.00 equiv) was added dropwise over 5 min to a solution of titanium(IV) isopropoxide (6.53 mL, 22.04 mmol, 2.00 equiv) and 1,2-bis(4-(*tert*-butyl)phenyl)ethyne[19,20] (3.20 g, 11.02 mmol, 1.00 equiv) in dry THF (44 mL, 0.25 M) under N_2 at -78 °C. The reaction mixture was stirred at rt for 2 h. The reaction mixture was cooled to 0 °C, then diluted with sat. NH_4Cl (50 mL). The reaction mixture was extracted with Et_2O (50 mL), washed with sat. brine (50 mL), dried with $MgSO_4$, filtered, and concentrated to afford crude product. The crude product was purified by filtration through a silica plug (elution with 100% pentane) to afford the title compound as a pale-yellow oil (2.34 g, 8.00 mmol, 73%).

ν_{max} (thin film) /cm^{-1} 2961, 2361, 1511, 1463, 1363, 1269, 1108, 1018, 883, 842, 824; **^{1}H NMR** (400 MHz, $CDCl_3$) δ 7.18 – 7.16 (m, 8H), 6.42 (s, 2H), 1.23 (s, 18H); **^{13}C NMR** (101 MHz, $CDCl_3$) δ 150.2, 134.7, 129.6, 128.7, 125.2, 34.7, 31.5; **HRMS** (EI) calc. for $C_{22}H_{28}$ ([M]$^+$): 292.2186; found 292.2191.

NB: Both (*E*)-1,2-bis(4-(tert-butyl)phenyl)ethene and a mixture of (*E*)-1,2-bis(4-(tert-butyl)phenyl)ethene and (*Z*)-1,2-bis(4-(tert-butyl)phenyl)ethene have been previously characterised.[21,22]

2,3-bis(4-(*tert*-butyl)phenyl)oxirane

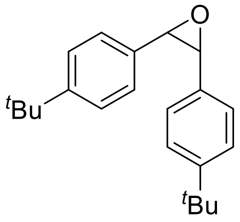

*m*CPBA (2.69 g, 12.00 mmol, 1.50 equiv) was added to (*Z*)-1,2-bis(4-(*tert*-butyl)phenyl)ethene (2.34 g, 8.00 mmol, 1.00 equiv) in CH_2Cl_2 (16 mL, 0.5 M) at 0 °C. The reaction mixture was stirred at rt for 24 h. The reaction mixture was filtered through celite. The filtrate was washed with sat. Na_2SO_3 (20 mL), sat. brine (20 mL), dried with $MgSO_4$, filtered, and concentrated to afford crude product. The crude product was purified by flash silica chromatography (elution gradient 0 to 5% Et_2O in pentane) to afford the title compound as a pale-yellow oil (2.30 g, 7.46 mmol, 93%).

ν$_{max}$ (thin film) /cm^{-1} 2962, 2361, 1517, 1392, 1268, 805; **^{1}H NMR** (400 MHz, $CDCl_3$) δ 7.24 – 7.19 (m, 4H), 7.15 – 7.08 (m, 4H), 4.31 (s, 2H), 1.25 (s, 18H); **^{13}C NMR** (101 MHz, $CDCl_3$) δ 150.5, 131.7, 126.9, 124.8, 60.1, 34.6, 31.4; **HRMS** (ESI$^+$) calc. for $C_{22}H_{29}O$ ([M+H]$^+$): 309.2213; found: 309.2214.

(±)-1,2-bis(4-(*tert*-butyl)phenyl)-2-(piperidin-1-yl)ethan-1-ol

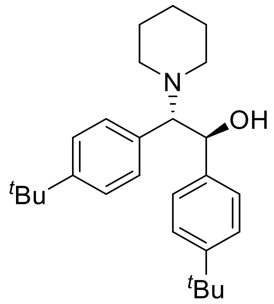

Prepared according to GP5 (72 h) with 2,3-bis(4-(*tert*-butyl)phenyl)oxirane (737 mg, 2.39 mmol, 1.00 equiv). The crude product was purified by flash silica chromatography (elution gradient 0 to 20% Et_2O in pentane) to afford the title compound as a white solid (823 mg, 2.09 mmol, 88%).

mp 125-127 °C; **ν$_{max}$** (thin film) /cm^{-1} 2961, 2360, 1364, 1271, 1110, 828; **^{1}H NMR** (400 MHz, $CDCl_3$) δ 7.27 – 7.22 (m, 2H), 7.20 – 7.12 (m, 4H), 7.05 – 7.00 (m, 2H), 5.48 (s, 1H), 5.01 (d, *J* = 10.5 Hz, 1H), 3.54 (d, *J* = 10.5 Hz, 1H), 2.62 (br s, 2H), 2.24 (br s, 2H), 1.80 – 1.49 (m, 4H), 1.40 – 1.31 (m, 2H), 1.28 (s, 9H), 1.22 (s, 9H); **^{13}C NMR** (101 MHz, $CDCl_3$) δ 150.3, 149.9, 139.0, 130.7, 129.7, 127.0, 124.9, 124.6, 76.1, 69.9, 50.4, 34.6, 34.5, 31.5, 31.4, 26.7, 24.4; **HRMS** (ESI$^+$) calc. for $C_{27}H_{40}ON$ ([M+H]$^+$): 394.3104; found: 394.3099.

(±)-1-(1,2-bis(4-(*tert*-butyl)phenyl)-2-chloroethyl)piperidine (2p)

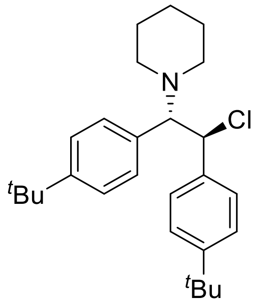

Prepared according to GP6 with (±)-1,2-bis(4-(*tert*-butyl)phenyl)-2-(piperidin-1-yl)ethan-1-ol (832 mg, 1.89 mmol, 1.00 equiv). The crude product was purified by flash silica chromatography (elution gradient 0 to 10% Et_2O in pentane) to afford **2p** as a white solid (289 mg, 0.70 mmol, 35%).

mp 117-119 °C; **ν$_{max}$** (thin film) /cm^{-1} 2962, 2360, 908, 731, 697; **^{1}H NMR** (400 MHz, $CDCl_3$) δ 7.17 – 6.95 (m, 6H), 6.85 – 6.71 (m, 2H), 5.29 (d, *J* = 10.5 Hz, 1H), 3.93 (d, *J* = 10.5 Hz, 1H), 2.47 (br s, 2H), 2.30 (br s, 2H), 1.67 – 1.37 (m, 4H), 1.34 – 1.20 (m, 2H), 1.14 (s, 9H), 1.11 (s, 9H); **^{13}C NMR** (101

MHz, $CDCl_3$) δ 150.6, 149.8, 137.3, 132.3, 129.0, 127.8, 125.0, 124.4, 75.1, 63.2, 50.6, 34.5, 34.4, 31.4, 31.3, 26.5, 24.8; **HRMS** (ESI^+) calc. for $C_{27}H_{39}N^{35}Cl$ ($[M+H]^+$): 412.2766; found: 412.2764.

(±)-2-(piperidin-1-yl)-1,2-bis(4-(trifluoromethyl)phenyl)ethan-1-ol

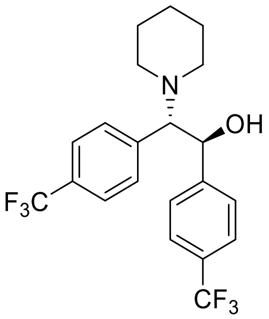

Prepared according to GP5 (48 h) with 2,3-bis(3-(trifluoromethyl)phenyl)oxirane[17] (1.00 g, 3.01 mmol, 1.00 equiv) and piperidine (1.44 mL, 15.05 mmol, 5.00 equiv). The crude product was purified by flash silica chromatography (elution gradient 0 to 20% Et_2O in pentane) to afford the title compound as a white solid (0.84 g, 2.02 mmol, 67%).

mp 118-119 °C; **ν_{max}** (thin film) /cm^{-1} 2939, 1322, 1161, 1109, 1067, 1018, 873, 836; **^{1}H NMR** (500 MHz, $CDCl_3$) δ 7.55 (d, *J* = 8.0 Hz, 2H), 7.42 (d, *J* = 8.0 Hz, 2H), 7.31 (d, *J* = 8.0 Hz, 2H), 7.21 (d, *J* = 8.0 Hz, 2H), 5.33 (s, 1H), 5.08 (d, *J* = 10.5 Hz, 1H), 3.54 (d, *J* = 10.5 Hz, 1H), 2.62 (br s, 2H), 2.28 (br s, 2H), 1.74 – 1.59 (m, 4H), 1.38 (br s, 2H); **^{19}F NMR** (471 MHz, $CDCl_3$) δ -62.56, -62.60; **^{13}C NMR** (126 MHz, $CDCl_3$) δ 145.6, 137.2, 130.3 (q, *J* = 32.5 Hz), 130.2, 129.8 (q, *J* = 32.5 Hz), 127.6, 125.2 (q, *J* = 4.0 Hz), 125.1 (q, *J* = 3.5 Hz), 124.2 (q, *J* = 272.0 Hz), 124.1 (q, *J* = 272.0), 76.9, 70.0, 50.5, 26.6, 24.2; **HRMS** (ESI$^+$) calc. for $C_{21}H_{22}ONF_6$ ([M+H]$^+$): 418.1600; found: 418.1596.

NB: In the ^{13}C spectra, one resonance of the quartet at 124.1 overlaps with the quartet at 125.2.

(±)-1-(2-chloro-1,2-bis(4-(trifluoromethyl)phenyl)ethyl)piperidine (2q)

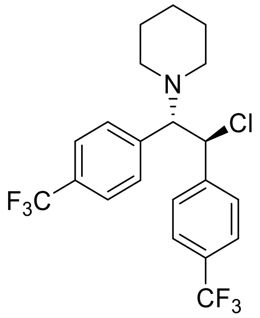

Prepared according to GP6 with (±)-2-(piperidin-1-yl)-1,2-bis(4-(trifluoromethyl)phenyl)ethan-1-ol (792 mg, 1.89 mmol, 1.00 equiv). The crude product was purified by flash silica chromatography (elution gradient 0 to 20% Et_2O in pentane) to afford **2q** as a white solid (559 mg, 1.29 mmol, 68%).

mp 125-127 °C; **ν_{max}** (thin film) /cm^{-1} 2937, 1323, 1164, 1110, 1068, 1018, 877, 848, 760, 720, 671; **^{1}H NMR** (500 MHz, $CDCl_3$) δ 7.47 – 7.41 (m, 4H), 7.33 (d, *J* = 8.0 Hz, 2H), 7.07 (d, *J* = 8.0 Hz, 2H), 5.43 (d, *J* = 10.0 Hz, 1H), 4.07 (d, *J* = 10.0 Hz, 1H), 2.54 – 2.46 (m, 2H), 2.40 – 2.32 (m, 2H), 1.69 –

1.58 (m, 4H), 1.41 – 1.33 (m, 2H); **^{19}F NMR** (471 MHz, $CDCl_3$) δ -62.59, -62.76; **^{13}C NMR** (126 MHz, $CDCl_3$) δ 143.4, 138.7, 130.4 (q, *J* = 32.5 Hz), 129.8 (q, *J* = 32.5 Hz), 129.4, 128.6, 125.5 (q, *J* = 4.0 Hz), 124.9 (q, *J* = 3.5 Hz), 124.1 (q, *J* = 272.0 Hz), 123.9 (q, *J* = 272.5 Hz), 75.2, 61.3, 50.1, 26.4, 24.6; **HRMS** (ESI$^+$) calc. for $C_{21}H_{21}N^{35}ClF_6$ ([M+H]$^+$): 436.1261; found: 436.1250.

NB: In the ^{13}C spectra, one resonance of the quartet at 124.1 overlaps with the quartet at 124.9.

(±)-*N*-(2-chloro-1,2-di(pyridin-2-yl)ethyl)-*N*-propylpropan-1-amine (2r)

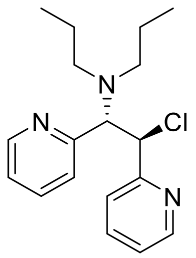

Prepared according to GP6 with (±)-2-(dipropylamino)-1,2-di(pyridin-2-yl)ethan-1-ol[23] (550 mg, 1.84 mmol, 1.00 equiv). The crude product was purified by flash silica chromatography (elution gradient 0 to 100% Et_2O in pentane) to afford **2r** as a yellow oil (461 mg, 1.45 mmol, 79%).

ν_{max} (thin film) /cm^{-1} 2958, 1589, 1570, 1470, 1434, 746; **^{1}H NMR** (400 MHz, $CDCl_3$) δ 8.49 – 8.23 (m, 2H), 7.49 – 7.37 (m, 2H), 7.23 (dt, *J* = 8.0, 1.0 Hz, 1H), 7.04 – 6.86 (m, 3H), 5.78 (d, *J* = 10.5 Hz, 1H), 4.68 (d, *J* = 10.5 Hz, 1H), 2.85 (ddd, *J* = 13.0, 8.5, 7.0 Hz, 2H), 2.35 (ddd, *J* = 13.0, 8.5, 5.0 Hz, 2H), 1.69 – 1.36 (m, 4H), 0.92 (t, *J* = 7.5 Hz, 6H); **^{13}C NMR** (101 MHz, $CDCl_3$) δ 159.1, 158.1, 149.4, 148.4, 136.3, 135.5, 124.6, 123.9, 122.5, 121.7, 69.2, 63.9, 53.0, 22.3, 12.0; **HRMS** (ESI$^+$) calc. for $C_{18}H_{25}N_3{}^{35}Cl$ ([M+H]$^+$): 318.1732; found: 318.1733.

(±)-*N*-benzyl-2-chloro-*N*-methylcyclohexan-1-amine (2s)

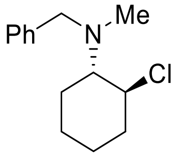

Prepared according to GP6 with (±)-2-(benzyl(methyl)amino)cyclohexan-1-ol[24] (500 mg, 2.28 mmol, 1.00 equiv). The crude product was purified by flash silica chromatography (elution gradient 0 to 10% Et_2O in pentane) to afford **2s** as a white solid (273 mg, 1.14 mmol, 50%).

mp 37-38 °C; **ν_{max}** (thin film) /cm^{-1} 2936, 1450, 734, 698; **^{1}H NMR** (400 MHz, $CDCl_3$) δ 7.44 – 7.38 (m, 2H), 7.34 – 7.28 (m, 2H), 7.25 – 7.19 (m, 1H), 3.96 (ddd, *J* = 11.0, 10.5, 4.5 Hz, 1H), 3.81 (d, *J* = 13.5 Hz, 1H), 3.59 (d, *J* = 13.5 Hz, 1H), 2.61 (ddd, *J* = 11.5, 10.5, 3.5 Hz, 1H), 2.35 – 2.28 (m, 1H), 2.26 (s, 3H), 2.03 – 1.95 (m, 1H), 1.81 – 1.63 (m, 3H), 1.36 – 1.21 (m, 3H); **^{13}C NMR** (101 MHz, $CDCl_3$) δ 140.4, 128.8, 128.3, 126.9, 68.3, 62.3, 58.3, 37.8, 36.8, 26.4×2, 25.2; **HRMS** (ESI$^+$) calc. for $C_{14}H_{21}N^{35}Cl$ ([M+H]$^+$): 238.1357; found: 238.1358.

Synthesis of products

1-((1*S*,2*S*)-2-azido-1,2-diphenylethyl)piperidine (3a)

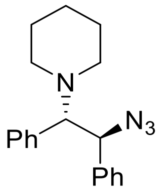

Standard scale: Prepared according to GP7 from **2a**. The reaction mixture was stirred at -20 °C. The crude product was purified by flash silica chromatography (elution gradient 0 to 10% Et_2O in pentane) to afford **3a** as a white solid (52 mg, 0.17 mmol, 85%, 93:7 e.r.).

Gram scale: A 50 mL pear-shaped Schlenk flask was charged sequentially with **2a** (1.50 g, 5.00 mmol, 1.00 equiv), sodium azide (781 mg, 12.00 mmol, 2.40 equiv), and (*S*)-**1k** (419 mg, 0.50 mmol, 0.10 equiv) under air. 1,2-difluorobenzene (20 mL, 0.25 M) was added to the Schlenk flask. The suspension was stirred at -20 °C at 900 rpm for 72 h. The reaction mixture was diluted with Et_2O (25 mL) and washed with H_2O (25 mL). **Aqueous washings containing the excess azide were quenched according to the method described *vida supra*.** The organic layer was dried with $MgSO_4$, filtered, and concentrated to afford crude product. The crude product was purified by flash silica chromatography (elution gradient 0 to 10% Et_2O in pentane) to afford **3a** as an off-white solid (1.23 g, 4.01 mmol, 80%, 93.5:6.5 e.r.). Continued elution (20% EtOAc in pentane) enabled recovery of crude catalyst (*S*)-**1k**. The crude catalyst was purified by recrystallization, layering pentane over a saturated solution of crude catalyst in Et_2O overnight. The solids were collected and dried to afford recovered (*S*)-**1k** (304 mg, 0.36 mmol, 73%) as a white solid.

mp 44-45 °C; ν_{max} (thin film) /cm^{-1} 3031, 2933, 2852, 2805, 2094, 698; **^{1}H NMR** (400 MHz, $CDCl_3$) δ 7.21 – 7.06 (m, 8H), 7.00 – 6.93 (m, 2H), 5.01 (d, *J* = 11.0 Hz, 1H), 3.94 (d, *J* = 11.0 Hz, 1H), 2.65 – 2.50 (m, 2H), 2.35 (br s, 2H), 1.72 – 1.60 (m, 4H), 1.34 (quin, *J* = 6.0 Hz, 2H); **^{13}C NMR** (101 MHz, $CDCl_3$) δ 138.2, 133.7, 129.5, 128.4, 128.0, 127.9, 127.7, 127.3, 75.1, 65.0, 50.9, 26.2, 24.7; **HRMS** (ESI$^+$) calc. for $C_{19}H_{23}N_4$ ($[M+H]^+$): 307.1917; found: 307.1917; $[\alpha]_D^{25}$ +30.6° (*c* 1.00, $CHCl_3$); **HPLC** DAICEL CHIRALPAK® IB-3, 0.1% $BuNH_2$ and 0.9% IPA in heptane, 1 mL min^{-1}, t_{major} = 3.73, t_{minor} = 3.23.

1-((1*S*,2*S*)-2-azido-1,2-diphenylethyl)-4-methoxypiperidine (3b)

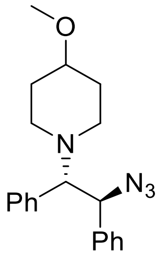

Prepared according to GP7 from **2b**. The reaction mixture was stirred at -20 °C. The crude product was purified by flash silica chromatography (elution gradient 0 to 10% Et_2O in pentane) to afford **3b** as a viscous oil (50 mg, 0.15 mmol, 74%, 93:7 e.r.).

ν_{max} (thin film) /cm^{-1} 2941, 2821, 2093, 1452, 1087, 946, 751, 698; **^{1}H NMR** (400 MHz, $CDCl_3$) δ 7.21 – 7.05 (m, 8H), 7.01 – 6.92 (m, 2H), 4.98 (d, *J* = 10.5 Hz, 1H), 3.98 (d, *J* = 10.5 Hz, 1H), 3.27 (s, 3H), 3.08 (ddt, *J* = 13.0, 8.5, 4.0 Hz, 1H), 3.02 – 2.91 (m, 1H), 2.77 (dt, *J* = 10.0, 4.5 Hz, 1H), 2.33 (ddd, *J* = 11.5, 10.0, 3.0 Hz, 1H), 2.16 – 2.04 (m, 1H), 2.04 – 1.92 (m, 2H), 1.83 – 1.59 (m, 2H); **^{13}C NMR** (101 MHz, $CDCl_3$) δ 138.1, 133.7, 129.4, 128.4, 128.0×2, 127.8, 127.4, 76.7, 74.3, 65.3, 55.5, 49.1, 45.8, 31.4, 30.8; **HRMS** (ESI^+) calc. for $C_{20}H_{25}ON_4$ ($[M+H]^+$): 337.2023; found: 337.2022; $[\alpha]_D^{25}$ +14.4° (*c* 1.00, $CHCl_3$); **HPLC** DAICEL CHIRALPAK® IB-3, 0.1% $BuNH_2$ and 0.9% IPA in heptane, 1 mL min^{-1}, t_{major} = 6.41, t_{minor} = 5.07.

1-((1*S*,2*S*)-2-azido-1,2-diphenylethyl)-4,4-difluoropiperidine (3c)

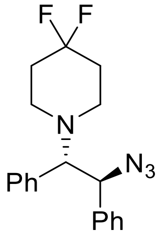

Prepared according to GP7 from **2c**. The reaction mixture was stirred at rt. The crude product was purified by flash silica chromatography (elution gradient 0 to 10% Et_2O in pentane) to afford **3c** as a white solid (59 mg, 0.17 mmol, 86%, 84:16 e.r.).

mp 82-84 °C; **ν_{max}** (thin film) /cm^{-1} 2093, 1363, 1160, 1082, 938, 699; **^{1}H NMR** (400 MHz, $CDCl_3$) δ 7.24 – 7.09 (m, 8H), 7.03 – 6.95 (m, 2H), 4.98 (d, *J* = 10.5 Hz, 1H), 4.05 (d, *J* = 10.5 Hz, 1H), 2.82 – 2.71 (m, 2H), 2.59 – 2.49 (m, 2H), 2.19 – 2.00 (m, 4H); **^{19}F NMR** (377 MHz, $CDCl_3$) δ -97.43 (br s); **^{13}C NMR** (101 MHz, $CDCl_3$) δ 137.7, 133.4, 129.1, 128.5, 128.2, 128.1, 127.9, 127.8, 122.0 (t, *J* = 241.5 Hz), 73.9, 65.4, 46.5, 34.3 (t, *J* = 22.5 Hz); **HRMS** (ESI^+) calc. for $C_{19}H_{21}N_4F_2$ ($[M+H]^+$): 343.1729; found: 343.1730; $[\alpha]_D^{25}$ +10.9° (*c* 1.00, $CHCl_3$); **HPLC** DAICEL CHIRALPAK® IB-3, 1% IPA in heptane, 1 mL min^{-1}, t_{major} = 7.13, t_{minor} = 5.34.

1-((1*S*,2*S*)-2-azido-1,2-diphenylethyl)pyrrolidine (3d)

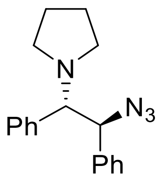

Prepared according to GP7 from **2d**. The reaction mixture was stirred at -20 °C. The crude product was purified by flash silica chromatography (elution gradient 0 to 10% Et_2O in pentane) to afford **3d** as a viscous oil (52 mg, 0.18 mmol, 89%, 94:6 e.r.).

ν_{max} (thin film) /cm^{-1} 2968, 2803, 2094, 1453, 1252, 757, 698; **^{1}H NMR** (400 MHz, $CDCl_3$) δ 7.21 – 7.11 (m, 6H), 7.11 – 7.07 (m, 2H), 7.02 – 6.97 (m, 2H), 5.03 (d, *J* = 8.5 Hz, 1H), 3.94 (d, *J* = 8.5 Hz, 1H), 2.68 – 2.55 (m, 4H), 1.80 – 1.70 (m, 4H); **^{13}C NMR** (101 MHz, $CDCl_3$) δ 137.6, 136.0, 129.6, 128.2, 128.1, 127.9, 127.7, 127.3, 72.0, 67.9, 50.5, 23.1; **HRMS** (ESI$^+$) calc. for $C_{18}H_{21}N_4$ ([M+H]$^+$): 293.1716; found: 293.1757; $[\alpha]_D^{25}$ +73.6° (*c* 1.00, $CHCl_3$); **HPLC** DAICEL CHIRALPAK® IA-3, 0.1% $BuNH_2$ and 0.15% IPA in heptane, 1 mL min^{-1}, t_{major} = 4.77, t_{minor} = 6.12.

4-((1*S*,2*S*)-2-azido-1,2-diphenylethyl)morpholine (3e)

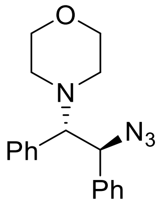

Prepared according to GP7 from **2e**. The reaction mixture was stirred at 0 °C. The crude product was purified by flash silica chromatography (elution gradient 0 to 20% Et_2O in pentane) to afford **3e** as a white solid (45 mg, 0.15 mmol, 73%, 91:9 e.r.).

mp 96-98 °C; **ν_{max}** (thin film) /cm^{-1} 2861, 2090, 1494, 1452, 1251, 1113, 1002, 881, 759, 699; **^{1}H NMR** (400 MHz, $CDCl_3$) δ 7.22 – 7.10 (m, 8H), 7.01 – 6.97 (m, 2H), 5.01 (d, *J* = 10.5 Hz, 1H), 3.95 (d, *J* = 10.5 Hz, 1H), 3.85 – 3.71 (m, 4H), 2.70 – 2.60 (m, 2H), 2.52 – 2.44 (m, 2H); **^{13}C NMR** (101 MHz, $CDCl_3$) δ 137.7, 133.4, 129.4, 128.4, 128.1, 127.9×2, 127.7, 74.6, 67.1, 64.9, 50.1; **HRMS** (ESI$^+$) calc. for $C_{18}H_{21}ON_4$ ([M+H]$^+$): 309.1710; found: 309.1710; $[\alpha]_D^{25}$ +28.9° (*c* 1.00, $CHCl_3$); **HPLC** DAICEL CHIRALPAK® IB-3, 0.1% $BuNH_2$ and 0.9% IPA in heptane, 1 mL min^{-1}, t_{major} = 12.13, t_{minor} = 8.67.

1-((1*S*,2*S*)-2-azido-1,2-diphenylethyl)-4-cyclohexylpiperazine (3f)

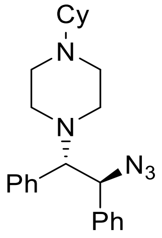

Prepared according to GP7 from **2f**. The reaction mixture was stirred at rt. The crude product was purified by flash silica chromatography (elution gradient 0 to 20% EtOAc in CH_2Cl_2) to afford **3f** as a white solid (57 mg, 0.15 mmol, 73%, 57:43 e.r.).

mp 78-80 °C; $\mathbf{v_{max}}$ (thin film) /cm^{-1} 2928, 2094, 1452, 755, 731, 698; **^{1}H NMR** (500 MHz, $CDCl_3$) δ 7.18 – 7.05 (m, 8H), 7.01 – 6.92 (m, 2H), 5.01 (d, *J* = 10.5 Hz, 1H), 3.95 (d, *J* = 10.5 Hz, 1H), 2.68 (s, 6H), 2.48 (s, 2H), 2.26 – 2.07 (m, 1H), 1.90 – 1.80 (m, 2H), 1.80 – 1.70 (m, 2H), 1.66 – 1.53 (m, 1H), 1.29 – 1.01 (m, 5H); **^{13}C NMR** (126 MHz, $CDCl_3$) δ 138.0, 133.4, 129.5, 128.4, 128.0, 128.0, 127.9, 127.4, 74.2, 65.0, 63.6, 49.6, 49.2, 29.2, 29.1, 26.4, 26.0×2; **HRMS** (ESI$^+$) calc. for $C_{24}H_{32}N_5$ ([M+H]$^+$): 390.2652; found: 390.2645; $[\alpha]_D^{25}$ +6.1° (*c* 1.00, $CHCl_3$); **HPLC** DAICEL CHIRALPAK® IB-3, 0.1% $BuNH_2$ and 0.9% IPA in heptane, 1 mL min^{-1}, t_{major} = 4.58, t_{minor} = 3.89.

1-((1*S*,2*S*)-2-azido-1,2-diphenylethyl)-4-phenylpiperazine (3g)

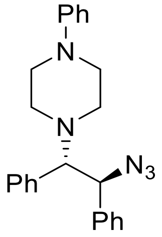

Prepared according to GP7 from **2g**. The reaction mixture was stirred at rt. The crude product was purified by flash silica chromatography (elution gradient 0 to 20% Et_2O in pentane) to afford **3g** as a white solid (67 mg, 0.17 mmol, 87%, 87:13 e.r.).

mp 103-105 °C; $\mathbf{v_{max}}$ (thin film) /cm^{-1} 2827, 2095, 1599, 1234, 755, 695; **^{1}H NMR** (400 MHz, $CDCl_3$) δ 7.18 – 7.11 (m, 2H), 7.11 – 7.00 (m, 8H), 6.94 – 6.89 (m, 2H), 6.83 – 6.78 (m, 2H), 6.78 – 6.72 (m, 1H), 4.95 (d, *J* = 10.5 Hz, 1H), 3.95 (d, *J* = 10.5 Hz, 1H), 3.25 – 3.11 (m, 4H), 2.76 – 2.68 (m, 2H), 2.57 – 2.49 (m, 2H); **^{13}C NMR** (101 MHz, $CDCl_3$) δ 151.5, 137.8, 133.4, 129.4, 129.1, 128.5, 128.1, 128.0, 127.9, 127.6, 119.8, 116.3, 74.3, 65.1, 49.6, 49.5; **HRMS** (ESI$^+$) calc. for $C_{26}H_{26}N_5$ ([M+H]$^+$): 384.2194; found: 384.2184; $[\alpha]_D^{25}$ +15.6° (*c* 1.00, $CHCl_3$); **HPLC** DAICEL CHIRALPAK® IB-3, 1% IPA in heptane, 1 mL min^{-1}, t_{major} = 11.71, t_{minor} =8.38.

2-((1*S*,2*S*)-2-azido-1,2-diphenylethyl)-1,2,3,4-tetrahydroisoquinoline (3h)

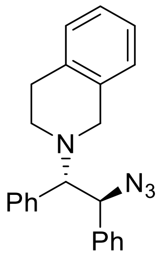

Prepared according to GP7 from **2h**. The reaction mixture was stirred at -10 °C. The crude product was purified by flash silica chromatography (elution gradient 0 to 10% Et_2O in pentane) to afford **3h** as a viscous oil (66 mg, 0.19 mmol, 93%, 93:7 e.r.).

ν_{max} (thin film) /cm^{-1} 2360, 2095, 1495, 1454, 1251, 1095, 741, 697; **^{1}H NMR** (400 MHz, $CDCl_3$) δ 7.26 – 7.09 (m, 13H), 7.08 – 7.01 (m, *J* = 3.0 Hz, 1H), 5.16 (d, *J* = 10.5 Hz, 1H), 4.19 (d, *J* = 10.5 Hz, 1H), 3.85 – 3.73 (m, 2H), 3.21 – 3.05 (m, 2H), 3.03 – 2.90 (m, 1H), 2.68 – 2.59 (m, 1H); **^{13}C NMR** (101 MHz, $CDCl_3$) δ 137.9, 135.1, 134.5, 133.8, 129.5, 128.8, 128.5, 128.1, 128.0, 128.0, 127.6, 126.6×2, 125.6, 73.6, 65.6, 52.8, 46.6, 29.6; **HRMS** (ESI$^+$) calc. for $C_{23}H_{23}N_4$ ($[M+H]^+$): 355.1917; found: 355.1911; $[\alpha]_D^{25}$ +31.3° (*c* 1.00, $CHCl_3$); **HPLC** DAICEL CHIRALPAK® IB-3, 1% IPA in heptane, 1 mL min^{-1}, t_{major} = 7.04, t_{minor} = 5.44.

(1*S*,2*S*)-2-azido-*N*-benzyl-*N*-methyl-1,2-diphenylethan-1-amine (3i)

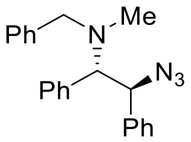

Prepared according to GP7 from **2i**. The reaction mixture was stirred at 0 °C. The crude product was purified by flash silica chromatography (elution gradient 0 to 10% Et_2O in pentane) to afford **3i** as a white solid (59 mg, 0.17 mmol, 86%, 91:9 e.r.).

mp 131-132 °C; **ν_{max}** (thin film) /cm^{-1} 2962, 2095, 1492, 1451, 1225, 1014, 701; **^{1}H NMR** (500 MHz, $CDCl_3$) δ 7.53 – 7.48 (m, 2H), 7.44 – 7.39 (m, 2H), 7.36 – 7.30 (m, 1H), 7.28 – 7.23 (m, 2H), 7.23 – 7.13 (m, 6H), 7.08 – 7.04 (m, 2H), 5.09 (d, *J* = 11.0 Hz, 1H), 4.17 (d, *J* = 11.0 Hz, 1H), 3.75 (d, *J* = 13.0 Hz, 1H), 3.47 (d, *J* = 13.0 Hz, 1H), 2.30 (s, 3H); **^{13}C NMR** (126 MHz, $CDCl_3$) δ 139.2, 137.9, 133.7, 129.5, 129.0, 128.5×2, 128.1, 128.0, 127.9, 127.5, 127.2, 71.4, 65.7, 58.9, 37.4; **HRMS** (ESI$^+$) calc. for $C_{22}H_{23}N_4$ ($[M+H]^+$): 343.1917; found: 343.1914; $[\alpha]_D^{25}$ +43.1° (*c* 1.00, $CHCl_3$); **HPLC** DAICEL CHIRALPAK® IB-3, 1% IPA in heptane, 1 mL min^{-1}, t_{major} = 5.06, t_{minor} = 4.23.

(1*S*,2*S*)-2-azido-*N*,*N*-dimethyl-1,2-diphenylethan-1-amine (3j)

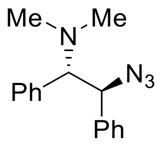

Prepared according to GP7 from **2j**. The reaction mixture was stirred at -10 °C. The crude product was purified by flash silica chromatography (elution gradient 0 to 20% EtOAc in CH_2Cl_2) to afford **3j** as a viscous oil (45 mg, 0.17 mmol, 84%, 92:8 e.r.).

ν_{max} (thin film) /cm^{-1} 2936, 2785, 2096, 1453, 1256, 752, 698; **^{1}H NMR** (500 MHz, $CDCl_3$) δ 7.22 – 7.06 (m, 8H), 7.02 – 6.92 (m, 2H), 4.99 (d, *J* = 11.0 Hz, 1H), 3.90 (d, *J* = 11.0 Hz, 1H), 2.29 (s, 6H); **^{13}C NMR** (126 MHz, $CDCl_3$) δ 137.6, 133.1, 129.6, 128.5, 128.2, 128.1, 127.8, 127.5, 73.1, 65.9, 41.2; **HRMS** (ESI$^+$) calc. for $C_{16}H_{19}N_4$ ([M+H]$^+$): 267.1604; found 267.1604; $[\alpha]_D^{25}$ +61.4° (*c* 1.00, $CHCl_3$); **HPLC** DAICEL CHIRALPAK® IB-3, 0.1% $BuNH_2$ and 0.9% IPA in heptane, 1 mL min^{-1}, t_{major} = 4.92, t_{minor} = 3.83.

1-((1*S*,2*S*)-2-azido-1,2-diphenylethyl)azepane (3k)

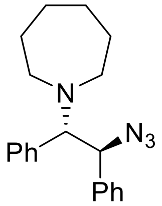

Prepared according to GP7 from **2k**. The reaction mixture was stirred at -20 °C. The crude product was purified by flash silica chromatography (elution gradient 0 to 10% Et_2O in pentane) to afford **3k** as a viscous oil (49 mg, 0.15 mmol, 76%, 94:6 e.r.).

ν_{max} (thin film) /cm^{-1} 2925, 2361, 2093, 1452, 1249, 758, 696; **^{1}H NMR** (400 MHz, $CDCl_3$) δ 7.19 – 7.09 (m, 8H), 7.06 – 7.01 (m, 2H), 4.87 (d, *J* = 11.0 Hz, 1H), 4.07 (d, *J* = 11.0 Hz, 1H), 2.86 (ddd, *J* = 11.5, 6.0, 4.5 Hz, 2H), 2.64 (ddd, *J* = 12.0, 7.5, 4.0 Hz, 2H), 1.77 – 1.64 (m, 4H), 1.62 – 1.54 (m, 4H); **^{13}C NMR** (101 MHz, $CDCl_3$) δ 138.5, 136.2, 129.1, 128.5, 128.0, 127.9, 127.8, 127.1, 74.2, 66.4, 52.2, 29.3, 27.0; **HRMS** (ESI$^+$) calc. for $C_{20}H_{25}N_4$ ([M+H]$^+$): 321.2074; found: 321.2070; $[\alpha]_D^{25}$ -9.1° (*c* 1.00, $CHCl_3$); **HPLC** DAICEL CHIRALPAK® IB-3, 0.1% $BuNH_2$ and 0.9% IPA in heptane, 1 mL min^{-1}, t_{major} = 3.46, t_{minor} = 3.03.

1-((1*S*,2*S*)-2-azido-1,2-bis(3-fluorophenyl)ethyl)piperidine (3l)

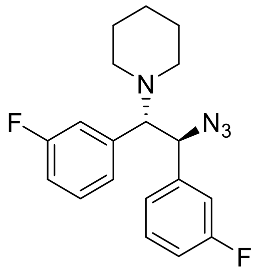

Prepared according to GP7 from **2l**. The reaction mixture was stirred at -10 °C. The crude product was purified by flash silica chromatography (elution gradient 0 to 10% Et_2O in pentane) to afford **3l** as a white solid (56 mg, 0.16 mmol, 82%, 89:11 e.r.).

mp 67-68 °C; **ν_{max}** (thin film) /cm^{-1} 2936, 2100, 1615, 1591, 1489, 1449, 1249, 1142, 777, 695; **^{1}H NMR** (500 MHz, $CDCl_3$) δ 7.19 – 7.09 (m, 2H), 6.93 – 6.80 (m, 4H), 6.78 – 6.73 (m, 1H), 6.69 (dt, *J* = 10.0, 2.0 Hz, 1H), 4.95 (d, *J* = 10.5 Hz, 1H), 3.88 (d, *J* = 10.5 Hz, 1H), 2.70 – 2.53 (m, 2H), 2.53 – 2.29 (m, 2H), 1.71 – 1.48 (m, 4H), 1.36 (quin, *J* = 6.0 Hz, 2H); **^{19}F NMR** (471 MHz, $CDCl_3$) δ -112.46, -113.16; **^{13}C NMR** (126 MHz, $CDCl_3$) δ 162.7 (d, *J* = 246.5 Hz), 162.4 (d, *J* = 246.0 Hz), 142.2 (d, *J* = 7.5 Hz), 137.5 (d, *J* = 6.0 Hz)., 130.0 (d, *J* = 8.5 Hz), 129.3 (d, *J* = 8.0 Hz), 125.1 (d, *J* = 3.0 Hz), 123.8 (d, *J* = 3.0 Hz), 116.1 (d, *J* = 21.0 Hz), 115.1 (d, *J* = 21.0 Hz), 114.9 (d, *J* = 22.0 Hz), 114.5 (d, *J* = 21.0 Hz), 74.9 (d, *J* = 1.5 Hz), 64.2 (d, *J* = 2.0 Hz), 51.0, 26.1, 24.6; **HRMS** (ESI$^+$) calc. for $C_{19}H_{21}F_2N_4$ ($[M+H]^+$): 343.1729; found: 343.1729; $[\alpha]_D^{25}$ +12.5° (*c* 1.00, $CHCl_3$, 89:11 e.r.); **HPLC** DAICEL CHIRALPAK® IB-3, 1% IPA in heptane, 1 mL min^{-1}, t_{major} = 4.55, t_{minor} = 3.72.

NB: Recrystallisation of **3l** (45 mg, 0.13 mmol) from hot hexanes afforded **3l** (14 mg, 0.04 mmol, 30%, 98:2 e.r.).

1-((1*S*,2*S*)-2-azido-1,2-bis(3-chlorophenyl)ethyl)piperidine (3m)

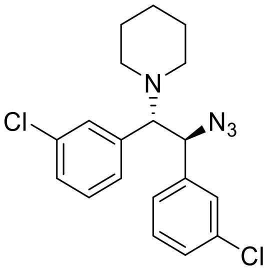

Prepared according to GP7 from **2m**. The reaction mixture was stirred at 0 °C. The crude product was purified by flash silica chromatography (elution gradient 0 to 10% Et_2O in pentane) to afford **3m** as a viscous oil (67 mg, 0.18 mmol, 89%, 90:10 e.r.).

ν_{max} (thin film) /cm^{-1} 2962, 2095, 1270, 785, 714, 693; **^{1}H NMR** (400 MHz, $CDCl_3$) δ 7.18 – 7.06 (m, 5H), 7.02 – 6.95 (m, 2H), 6.88 – 6.83 (m, 1H), 4.93 (d, *J* = 10.5 Hz, 1H), 3.84 (d, *J* = 10.5 Hz, 1H), 2.58 (dt, *J* = 11.0, 5.0 Hz, 2H), 2.33 (dt, *J* = 9.5, 5.0 Hz, 2H), 1.71 – 1.62 (m, 4H), 1.36 (quin, *J* = 6.0 Hz,

2H); **^{13}C NMR** (101 MHz, $CDCl_3$) δ 139.9, 135.6, 134.4, 133.9, 129.7, 129.3, 129.1, 128.3, 128.1, 127.7, 127.5, 126.2, 74.9, 64.2, 51.0, 26.0, 24.6; **HRMS** (ESI$^+$) calc. for $C_{19}H_{21}N_4{}^{35}Cl_2$ ([M+H]$^+$): 375.1138; found: 375.1142; $[\alpha]_D^{25}$ -5.3° (*c* 1.00, $CHCl_3$); **HPLC** DAICEL CHIRALPAK® IA-3, 1% IPA in heptane, 1 mL min^{-1}, t_{major} = 6.96, t_{minor} = 5.49.

1-((1*S*,2*S*)-2-azido-1,2-bis(3-(trifluoromethyl)phenyl)ethyl)piperidine (3n)

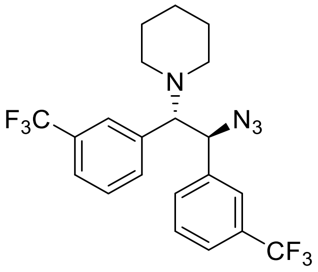

Prepared according to GP7 from **2n**. The reaction mixture was stirred at 0 °C. The crude product was purified by flash silica chromatography (elution gradient 0 to 10% Et_2O in pentane) to afford **3n** as a white solid (76 mg, 0.17 mmol, 86%, 85:15 e.r.).

mp 43-44 °C; **ν_{max}** (thin film) /cm^{-1} 2938, 2100, 1326, 1162, 1120, 1074, 702; **^{1}H NMR** (400 MHz, $CDCl_3$) δ 7.40 – 7.22 (m, 6H), 7.20 – 7.15 (m, 1H), 7.12 – 7.07 (m, 1H), 5.07 (d, *J* = 10.0 Hz, 1H), 3.90 (d, *J* = 10.0 Hz, 1H), 2.69 – 2.48 (m, 2H), 2.48 – 2.21 (m, 2H), 1.70 – 1.61 (m, 4H), 1.41 – 1.28 (m, 2H); **^{19}F NMR** (377 MHz, $CDCl_3$) δ -62.85, -62.99; **^{13}C NMR** (101 MHz, $CDCl_3$) δ 138.8, 134.7, 132.8, 131.3, 130.9 (q, *J* = 32.5 Hz), 130.3 (q, *J* = 32.5 Hz), 129.0, 128.5, 125.8 (q, *J* = 4.0 Hz), 125.0 (q, *J* = 4.0 Hz), 124.9 (q, *J* = 4.0 Hz), 124.4 (q, *J* = 3.7 Hz), 124.1 (q, *J* = 272.5 Hz), 123.9 (q, *J* = 272.5 Hz), 75.6, 64.3, 51.2, 26.0, 24.5; **HRMS** (ESI$^+$) calc. for $C_{21}H_{21}N_4F_6$ ([M+H]$^+$): 443.1665; found: 443.1653; $[\alpha]_D^{25}$ +13.9° (*c* 1.00, $CHCl_3$); **HPLC** DAICEL CHIRALPAK® IB-3, 0.5% IPA in heptane, 1 mL min^{-1}, t_{major} = 4.95, t_{minor} = 3.79.

1-((1*S*,2*S*)-2-azido-1,2-bis(4-bromophenyl)ethyl)piperidine (3o)

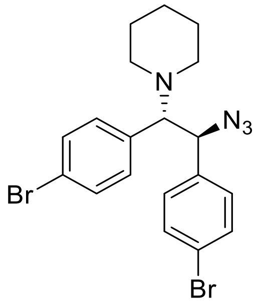

Standard scale: Prepared according to GP7 from **2o**. The reaction mixture was stirred at 0 °C. The crude product was purified by flash silica chromatography (elution gradient 0 to 10% Et_2O in pentane) to afford **3o** as a white solid (73 mg, 0.16 mmol, 79%, 93:7 e.r.).

Half-gram scale: A Schlenk flask was charged sequentially with **2o** (0.50 g, 1.09 mmol, 1.00 equiv), sodium azide (170 mg, 2.62 mmol, 2.40 equiv), and (*S*)-**1k** (91 mg, 0.11 mmol, 0.10 equiv) under air. 1,2-difluorobenzene (4.36 mL, 0.25 M) was added to the Schlenk flask. The suspension was stirred at 0 °C at 900 rpm for 72 h. The reaction mixture was diluted with Et_2O (10 mL) and washed with H_2O (10 mL). **Aqueous washings containing the excess azide were quenched according to the method described *vida supra*.** The organic layer was dried with $MgSO_4$, filtered, and concentrated to afford crude product. The crude product was purified by flash silica chromatography (elution gradient 0 to 10% Et_2O in pentane) to afford an off-white solid (459 mg, 0.99 mmol, 91%, 93:7 e.r.). A sample of this (144 mg, 0.31 mmol, 1.00 equiv) was recrystallized from hot hexane to afford **3o** as an off-white crystalline solid, suitable for X-ray diffraction (110 mg, 0.24 mmol, 76%, 98.5:1.5 e.r.).

mp 120-122 °C; $\mathbf{v_{max}}$ (thin film) /cm^{-1} 2934, 2098, 1488, 1097, 1010, 908, 815, 728; **^{1}H NMR** (400 MHz, $CDCl_3$) δ 7.34 – 7.27 (m, 4H), 7.01 – 6.96 (m, 2H), 6.85 – 6.79 (m, 2H), 4.92 (d, *J* = 10.5 Hz, 1H), 3.83 (d, *J* = 10.5 Hz, 1H), 2.59 – 2.42 (m, 2H), 2.42 – 2.13 (m, 2H), 1.71 – 1.57 (m, 4H), 1.49 – 1.24 (m, 2H); **^{13}C NMR** (101 MHz, $CDCl_3$) δ 137.0, 132.5, 131.7, 131.1, 130.9, 129.6, 122.1, 121.5, 74.6, 64.1, 50.9, 26.0, 24.6; **HRMS** (ESI$^+$) calc. for $C_{19}H_{21}N_4{}^{79}Br_2$ ($[M+H]^+$): 463.0128; found: 463.0125; $[\alpha]_D^{25}$ -2.6° (*c* 1.00, $CHCl_3$, 95.5:1.5 e.r.); **HPLC** DAICEL CHIRALPAK® OJ-3, 1% EtOH in heptane, 1 mL min^{-1}, t_{major} = 14.00, t_{minor} = 9.03.

1-((1*S*,2*S*)-2-azido-1,2-bis(4-(*tert*-butyl)phenyl)ethyl)piperidine (3p)

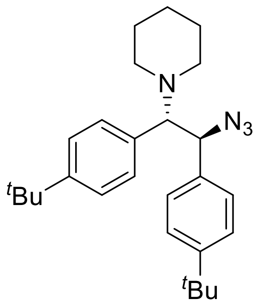

Prepared according to GP7 from **2p**. The reaction mixture was stirred at -20 °C. The crude product was purified by flash silica chromatography (elution gradient 0 to 10% Et_2O in pentane) to afford **3p** as a white solid (74 mg, 0.18 mmol, 88%, 96:4 e.r.).

mp 62-63 °C; $\mathbf{v_{max}}$ (thin film) /cm^{-1} 2935, 2097, 1270, 1110, 909, 823, 733; **^{1}H NMR** (400 MHz, $CDCl_3$) δ 7.20 – 7.10 (m, 4H), 7.09 – 7.03 (m, 2H), 6.95 – 6.87 (m, 2H), 4.96 (d, *J* = 10.5 Hz, 1H), 3.92 (d, *J* = 10.5 Hz, 1H), 2.75 – 2.54 (m, 2H), 2.54 – 2.25 (m, 2H), 1.74 – 1.57 (m, 4H), 1.43 – 1.33 (m, 2H), 1.24 (s, 9H), 1.21 (s, 9H); **^{13}C NMR** (101 MHz, $CDCl_3$) δ 150.5, 149.8, 135.3, 131.1, 129.2, 127.6, 125.2, 124.4, 74.4, 65.1, 51.0, 34.5, 34.4, 31.4, 31.3, 26.3, 24.7; **HRMS** (ESI$^+$) calc. for $C_{27}H_{39}N_4$

([M+H]+): 419.3169; found: 419.3174; $[\alpha]_D^{25}$ +11.2° (*c* 1.00, $CHCl_3$); **HPLC** DAICEL CHIRALPAK® IA-3 0.5% IPA in heptane, 1 mL min^{-1}, t_{major} = 6.96, t_{minor} = 5.49.

1-((1*S*,2*S*)-2-azido-1,2-bis(4-(trifluoromethyl)phenyl)ethyl)piperidine (3q)

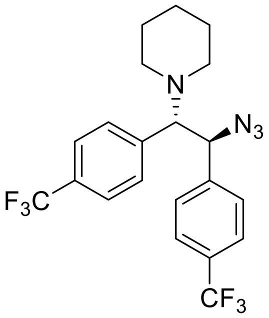

Prepared according to GP7 from **2q**. The reaction mixture was stirred at 0 °C. The crude product was purified by flash silica chromatography (elution gradient 0 to 10% Et_2O in pentane) to afford **3q** as a white solid (76 mg, 0.17 mmol, 86%, 88:12 e.r.).

mp 60-62 °C; $\mathbf{v_{max}}$ (thin film) /cm^{-1} 2938, 2102, 1322, 1163, 1109, 1067, 829; **^{1}H NMR** (400 MHz, $CDCl_3$) δ 7.46 – 7.37 (m, 4H), 7.25 – 7.19 (m, 2H), 7.10 – 7.03 (m, 2H), 5.04 (d, *J* = 10.5 Hz, 1H), 3.94 (d, *J* = 10.5 Hz, 1H), 2.61 – 2.50 (m, 2H), 2.37 – 2.26 (m, 2H), 1.70 – 1.60 (m, 4H), 1.38 – 1.28 (m, 2H); **^{19}F NMR** (377 MHz, $CDCl_3$) δ -62.62, -62.78; **^{13}C NMR** (101 MHz, $CDCl_3$) δ 141.7, 137.4, 130.4 (q, *J* = 32.5 Hz), 129.8 (q, *J* = 32.5 Hz), 129.5, 128.4, 125.6 (q, *J* = 4.0 Hz), 124.9 (q, *J* = 4.0 Hz), 124.1 (q, *J* = 272.0 Hz), 123.9 (q, *J* = 272.5 Hz), 75.0, 64.2, 51.1, 26.0, 24.5; **HRMS** (ESI+) calc. for $C_{21}H_{21}N_4F_6$ ([M+H]+): 443.1665; found: 443.1650; $[\alpha]_D^{25}$ +11.3° (*c* 1.00, $CHCl_3$); **HPLC** DAICEL CHIRALPAK® OJ-3, 1% EtOH in heptane, 1 mL min^{-1}, t_{major} = 16.23, t_{minor} = 6.52.

***N*-((1*R*,2*R*)-2-azido-1,2-di(pyridin-2-yl)ethyl)-*N*-propylpropan-1-amine (3r)**

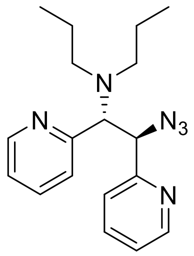

NaN_3 (31.2 mg, 0.48 mmol, 2.40 equiv) was added to a solution of **2r** (64 mg, 0.2 mmol, 1.00 equiv) and (*S*)-**1k** (16.7 mg, 0.02 mmol, 0.1.00 equiv) in 1,2-difluorobenzene (0.8 mL, 0.25 M) in a 2 mL Schlenk tube (7 mm internal diameter) under air. The suspension was stirred at -20 °C at 1200 rpm for 72 h. The reaction mixture was diluted with Et_2O (2 mL) and washed with H_2O (2 mL). **Aqueous washings containing the excess azide were quenched according to the method described *vida supra*.** The organic layer was dried with $MgSO_4$, filtered, and concentrated to afford crude product.

The crude product was purified by flash silica chromatography (elution gradient 0 to 20% EtOAc in CH_2Cl_2) to afford **3r** as a clear oil (55 mg, 0.17 mmol, 85%, 84:16 e.r.).

ν_{max} (thin film) /cm^{-1} 2959, 2095, 1589, 1570, 1470, 1434, 996, 782, 747; **^{1}H NMR** (400 MHz, $CDCl_3$) δ 8.47 – 8.35 (m, 2H), 7.43 – 7.34 (m, 2H), 7.08 (dt, *J* = 8.0, 1.0 Hz, 1H), 7.00 – 6.90 (m, 3H), 5.21 (d, *J* = 10.5 Hz, 1H), 4.58 (d, *J* = 10.5 Hz, 1H), 2.81 (ddd, *J* = 13.0, 9.5, 6.5 Hz, 2H), 2.36 – 2.25 (m, 2H), 1.64 – 1.42 (m, 4H), 0.89 (t, *J* = 7.5 Hz, 6H); **^{13}C NMR** (101 MHz, $CDCl_3$) δ 158.1, 157.0, 149.4, 148.5, 136.1, 135.5, 124.7, 123.9, 122.5, 121.8, 68.1, 65.6, 53.0, 21.9, 11.9; **HRMS** (ESI^+) calc. for $C_{18}H_{25}N_6$ ($[M+H]^+$): 325.2135; found: 325.2139; $[\alpha]_D^{25}$ +37.2° (*c* 1.00, $CHCl_3$); **HPLC** DAICEL CHIRALPAK® IC-3, 0.1% $BuNH_2$ and 0.9% IPA in heptane, 1 mL min^{-1}, t_{major} = 6.48, t_{minor} = 5.52.

2-azido-*N*-benzyl-*N*-methylcyclohexan-1-amine (3s)

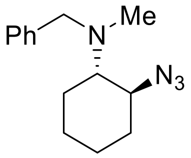

Prepared according to GP7 from **2s**. The reaction mixture was stirred at rt. The crude product was purified by flash silica chromatography (elution gradient 0 to 10% Et_2O in pentane) to afford **3s** as a clear oil (46 mg, 0.19 mmol, 94%, 52:48 e.r.).

ν_{max} (thin film) /cm^{-1} 2933, 2089, 1451, 1460, 737, 698; **^{1}H NMR** (400 MHz, $CDCl_3$) δ 7.40 – 7.35 (m, 2H), 7.32 – 7.26 (m, 2H), 7.24 – 7.18 (m, 1H), 3.71 (d, *J* = 13.5 Hz, 1H), 3.60 (d, *J* = 13.5 Hz, 1H), 3.29 (td, *J* = 10.5, 4.5 Hz, 1H), 2.50 (ddd, *J* = 11.5, 10.5, 3.5 Hz, 1H), 2.20 (s, 3H), 2.08 – 1.98 (m, 1H), 1.96 – 1.86 (m, 1H), 1.79 – 1.65 (m, 2H), 1.33 – 1.11 (m, 4H); **^{13}C NMR** (101 MHz, $CDCl_3$) δ 139.9, 128.8, 128.3, 127.0, 66.3, 61.5, 59.0, 36.2, 32.4, 25.1, 25.0, 24.2; **HRMS** (ESI^+) calc. for $C_{14}H_{21}N_4$ ($[M+H]^+$): 245.1761; found: 245.1761; **HPLC** DAICEL CHIRALPAK® IF-3 0.1% IPA in heptane, 1 mL min^{-1}, t_{major} = 7.57, t_{minor} = 6.51.

NB: Absolute configuration of major enantiomer not determined.[15]

Product derivatization

(1*S*,2*S*)-1,2-diphenyl-2-(piperidin-1-yl)ethan-1-amine

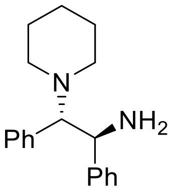

Pd/C (10 wt. %, 224 mg, 0.21 mmol, 0.05 equiv) was added to a suspension of **3a** (1.29 g, 4.21 mmol, 1.00 equiv, 93.5:6.5 e.r.) in MeOH (16.8 mL) at rt under N_2. The reaction mixture was purged with H_2 (balloon), then stirred at rt for 2 h under H_2 (1 atm). The reaction mixture was filtered through celite and concentrated to afford crude product. The crude product was purified by flash silica chromatography (elution gradient 20 to 60% EtOAc in pentane) to afford the title compound as a white solid (0.94 g, 3.35 mmol, 80%, 93.5:6.5 e.r.).

mp 76-78 °C; ν_{max} (neat) /cm^{-1} 2933, 1490, 1451, 1303, 696; **^{1}H NMR** (400 MHz, $CDCl_3$) δ 7.25 – 7.20 (m, 2H), 7.19 – 7.07 (m, 5H), 7.07 – 6.96 (m, 3H), 4.49 (d, *J* = 10.5 Hz, 1H), 3.62 (d, *J* = 10.5 Hz, 1H), 2.55 (br s, 2H), 2.28 (br s, 2H), 1.95 (br s, 2H), 1.74 – 1.61 (m, 2H), 1.61 – 1.47 (m, 2H), 1.39 – 1.26 (m, 2H); **^{13}C NMR** (101 MHz, $CDCl_3$) δ 143.5, 135.0, 129.7, 128.2, 128.1, 127.4, 126.9, 126.8, 76.6, 55.1, 50.6, 26.9, 24.8; **HRMS** (ESI$^+$) calc. for $C_{19}H_{25}N_2$ ([M+H]$^+$): 281.2012; found: 281.2012; $[\alpha]_D^{25}$ -22.0° (*c* 0.5, EtOH); **HPLC** DAICEL CHIRALPAK® IA-3, 0.1% $BuNH_2$ and 4.9% IPA in heptane, 1 mL min^{-1}, t_{major} = 6.43, t_{minor} = 5.40.

NB: Synthesis of (1*R*,2*R*)-1,2-diphenyl-2-(piperidin-1-yl)ethan-1-amine from commercial (1*R*,2*R*)-(+)-1,2-diphenylethane-1,2-diamine according to the literature procedure[25] provided a sample with $[\alpha]_D^{25}$ +30.8° (*c* 0.5, EtOH), providing further confirmation of absolute configuration.

(1*S*,2*S*)-1,2-diphenyl-1,2-di(piperidin-1-yl)ethane (4)

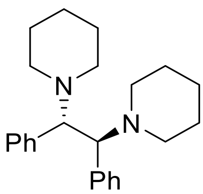

1,5-dibromopentane (0.50 mL, 3.69 mmol, 1.10 equiv) was added to a suspension of (1*S*,2*S*)-1,2-diphenyl-2-(piperidin-1-yl)ethan-1-amine (935 mg, 3.33 mmol, 1.00 equiv, 93.5:6.5 e.r.) and K_2CO_3 (1.15 mg, 8.38 mmol, 2.50 equiv) in MeCN (26.8 mL). The reaction mixture was heated under reflux for 24 h. The reaction mixture was filtered through celite and concentrated to afford crude product. The crude product was purified by flash silica chromatography (elution gradient 0 to 100% Et_2O in pentane) to afford **4** as a white solid (1.09 g, 3.13 mmol, 94%).

mp 164-165 °C; ν_{max} (thin film) /cm^{-1} 2928, 1451, 1161, 874, 696; **^{1}H NMR** (400 MHz, $CDCl_3$) δ 7.15 – 7.09 (m, 4H), 7.09 – 7.00 (m, 6H), 4.18 (s, 2H), 2.69 – 2.52 (m, 4H), 2.46 – 2.26 (m, 4H), 1.67 – 1.56 (m, 8H), 1.38 – 1.20 (m, 4H); **^{13}C NMR** (101 MHz, $CDCl_3$) δ 137.2, 129.4, 127.5, 126.5, 69.2, 50.3, 27.1, 25.2; **HRMS** (ESI$^+$) calc. for $C_{24}H_{33}N_2$ ([M+H]$^+$): 349.2638; found: 349.2638; $[\alpha]_D^{25}$ -13.8° (*c* 1.0, $CHCl_3$).

NB: Enantiomeric ratio was determined by ^{1}H NMR analysis with (*R*)-(−)-1,1′-Binaphthyl-2,2′-diyl hydrogenphosphate as chiral shift reagent.[26] **4** (3.5 mg) and (*R*)-(−)-1,1′-Binaphthyl-2,2′-diyl hydrogenphosphate (3.5 mg) was dissolved in $CDCl_3$ (1 mL). 0.1 mL of this solution was diluted 10-fold to 1 mM. Sample analysis was conducted on a Bruker AVANCE III 500 MHz spectrometer cooled to 233 K. ^{1}H NMR spectra were collected with 512 scans. Data was processed with MestReNova 12.0.0. qGSD (quantitative global spectrum deconvolution) of the signals at 5.02 (minor) and 4.97 (major) provided the enantiomeric ratio.

Non-linear effect study

Non-linear effect study used GP4 with scalemic mixtures of catalyst **1k** and sodium azide (7.8 mg, 0.12 mmol, 1.20 equiv). **HPLC** DAICEL CHIRALPAK® IB-3, 0.1% $BuNH_2$ and 0.9% IPA in heptane, 1 mL min^{-1}, t_1 = 3.23, t_2 = 3.73.

Scalemic mixtures of catalyst **1k** were prepared by mixing (*S*)-**1k** with (±)-**1k**, dissolving in CH_2Cl_2, then evaporating the mixture to dryness. Enantiopurity of each catalyst batch was determined by HPLC. **HPLC** DAICEL CHIRALPAK® IH, 2% EtOH in heptane, 1 mL min^{-1}, t_{major} = 6.06, t_{minor} = 9.16.

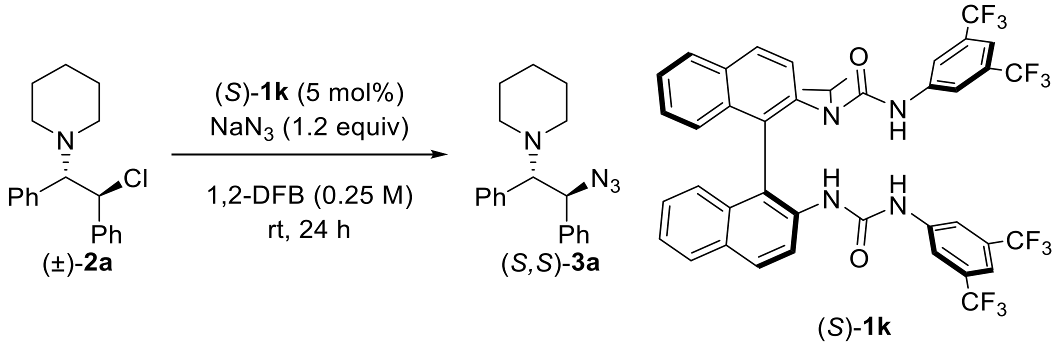

entry	ee of **1k**	ee of **3a**[a]
1	100.0	72.8
2	79.4	61.4
3	56.2	39.2
4	37.8	27.6
5	19.2	12.8
6	7.6	4.0
7	0.0	-0.2

[a]Mean of two runs, major enantiomer of **1k** in scalemic mixtures is (*S*).

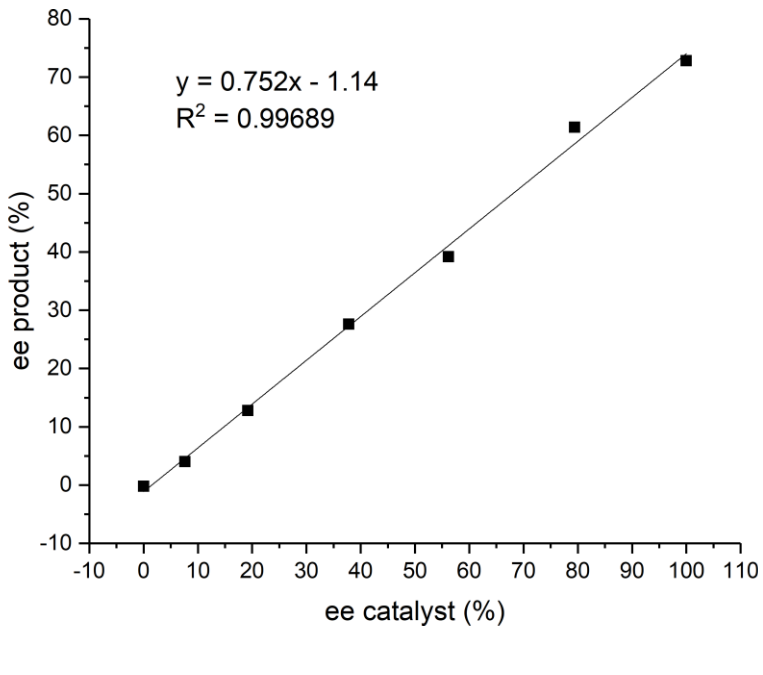

ReactIR data collection

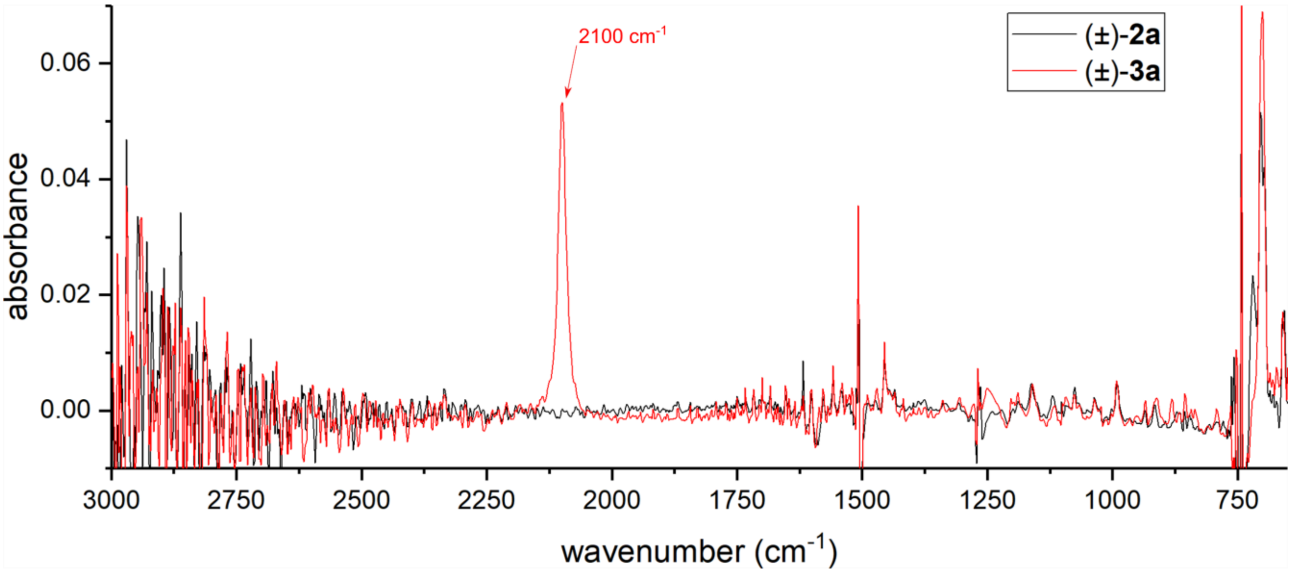

Figure S14 Reference spectra of (±)-**2a** and (±)-**3a.**

Reaction monitoring by *in situ* infra-red spectroscopy was conducted with a Mettler Toledo ReactIR 15 equipped with a 6.3 mm silicon composite probe. Reactions were conducted in a 5 cm tall test tube with a B12 adaptor made from medium (1.5 ± 0.25 mm) walled tubing. Reactions were stirred with an 8×3 mm magnetic stir bar.

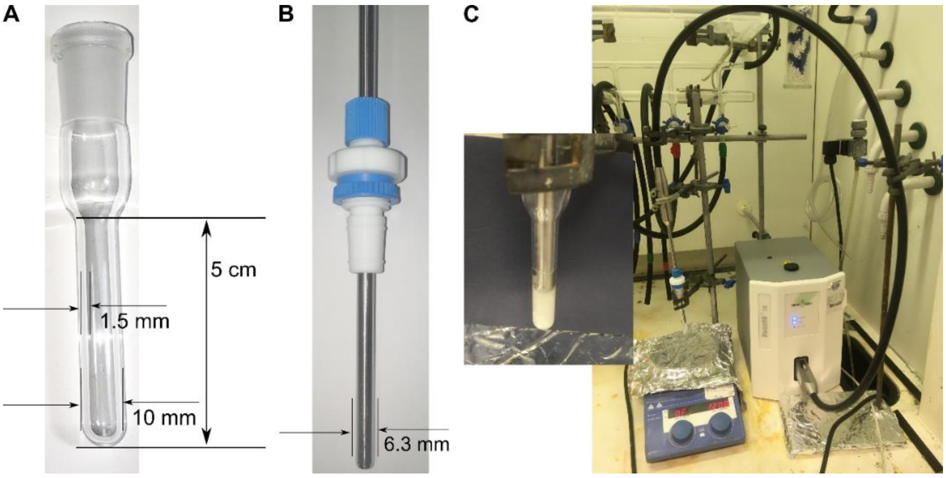

Figure S15 (A) Dimensions of reaction vessel used. (B) Dimension of silicon composite probe used. (C) Reaction setup inset with stirred reaction mixture at 1200 rpm under standard conditions.

A stock solution containing substrate and internal standard was prepared by making up a 1mL volumetric flask. Catalyst and NaN_3 was added to the selected reaction vessel charged with an 8×3 mm stir bar. 1,2-difluorobenzene (0.4 mL) was slowly injected. The probe was lowered, and the stirring and acquisition was started to pre-stir the reaction mixture. After 15 minutes, the substrate stock solution was carefully injected. The probe was lifted such that it was just touching the surface of the reaction mixture. The reaction was stirred for the desired time. The reaction was quenched by filtration through a plug of silica and eluted with Et_2O. The reaction mixture was

filtered through silica, eluted with Et_2O and concentrated. Full conversion was confirmed by ^1H NMR. Enantiomeric excess was determined on a sample purified by pTLC.

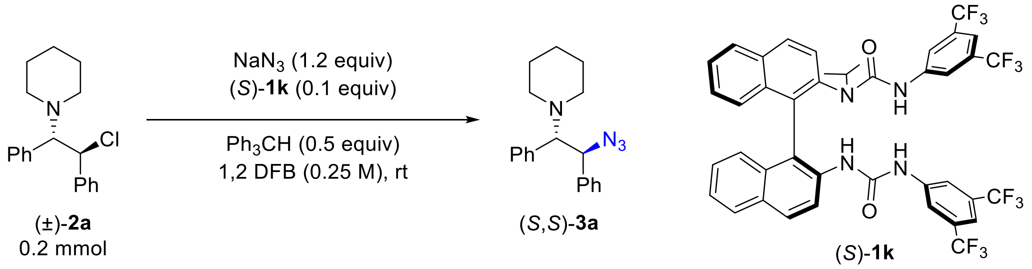

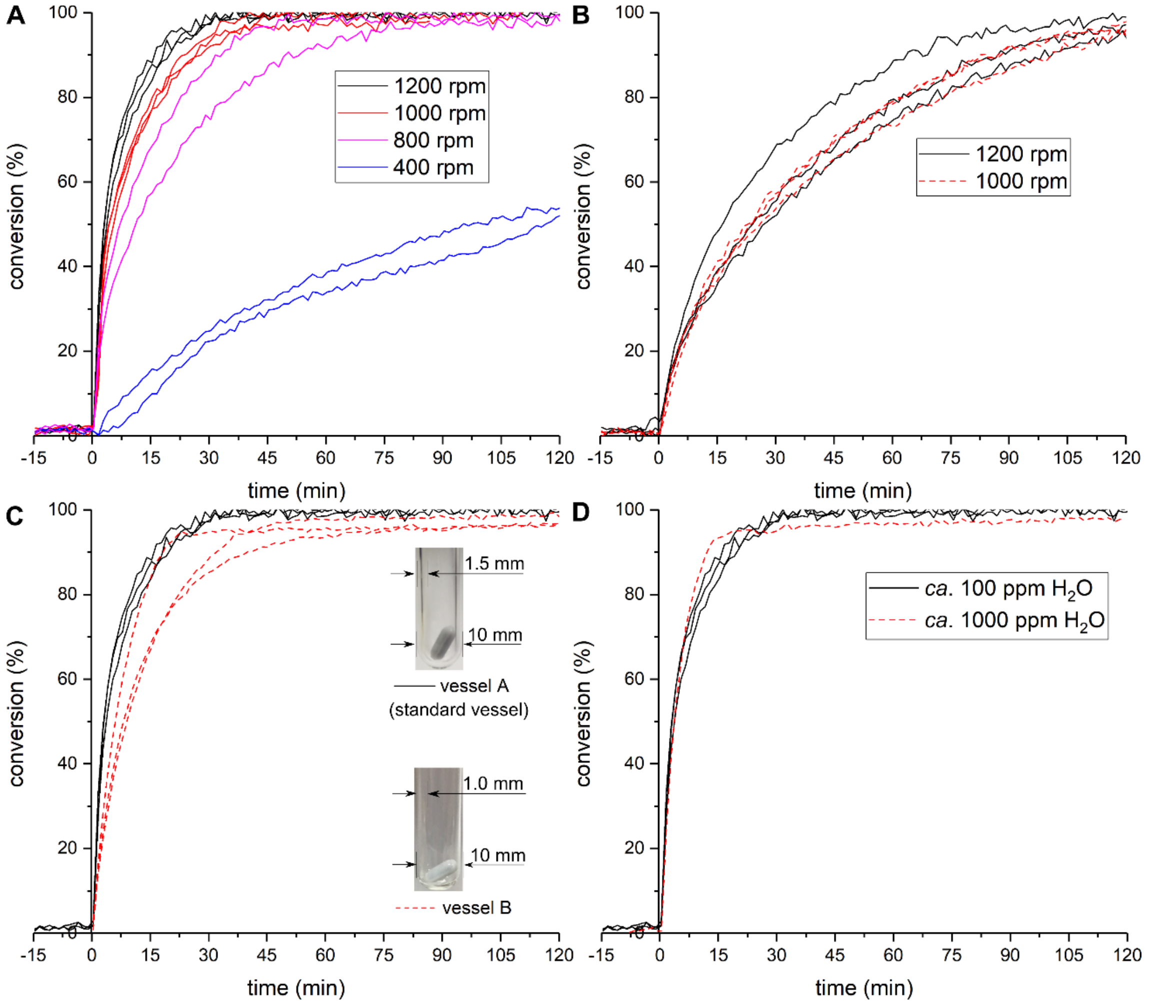

Figure S16 (A) Conversion at 1200, 1000, 800, and 400 rpm with 0.1 equiv (*S*)-**1k**. (B) Conversion at 1200 and 1000 rpm with 0.0125 equiv (*S*)-**1k**. (C) Conversion with alternate reaction vessel and 0.1 equiv (*S*)-**1k**, with images demonstrating the reaction vessels and angle of the 8×3 mm stir bar used. (D) Overlay of standard reaction, and reaction in 1,2-DFB saturated with water. 1,2-DFB = 1,2-difluorobenzene.

Conducting the background reaction with 1,2-DFB saturated with water resulted in visibly different reactions with the azide suspension appearing much less fine and coming together in small clumps on the sides of the reaction vessel.

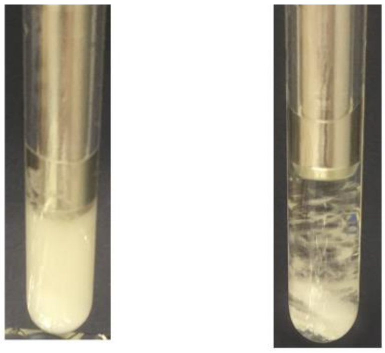

Figure S17 Differences in the appearance of the transformation when conducted with 1,2-DFB saturated with water

Development and Evaluation of Empirical Rate Equation

A series of generic models (A to H, **Figure S18, S19 and S20**) were considered for the reaction of **2a** with NaN_3 that is catalyzed by **1k**. These models were used to develop and rationalize an empirical relationship for the kinetics established by in situ ATR-FT-IR analysis of the rate of generation of product **3a** as a function of [**2a**]$_t$, [**1k**]$_{tot}$, and {NaN_3}{NaCl}. The latter is treated as a quasi-homogeneous mixture of two salts, with proportions that vary systematically as the reactions evolve.

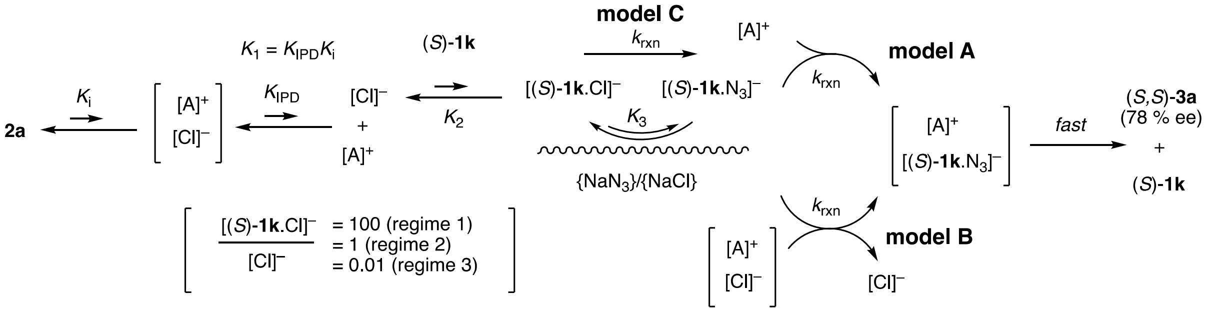

Figure S18 Models A, B, C and regimes 1, 2, 3, employing eqn (2) to (10).

In models A, B and C, **Figure S18**, the catalyst (**1k**) interacts with the chloride ion liberated by ionization of substrate **2a**. Ionization of **2a** can generate (K_i) a chloride aziridinium ion pair (A^+Cl^-) which can then dissociate (K_{IPD}) to free ions (Cl^- and A^+; $K_1 = K_iK_{IPD}$). The free chloride ion binds ($1/K_2$) in a separate equilibrium with the catalyst to generate an H-bonded chloride anion, [**1k**·Cl]$^-$. The latter can also be generated by direct reaction of **1k** with the ion-paired chloride ([A^+Cl^-]). Both routes lead to the same equilibrium concentrations of [**1k**], [**1k**·Cl]$^-$, [Cl^-], [A^+], ([A^+Cl^-]), and [**2a**]; eqn (2) to (5).

$$K_i[\mathbf{2a}] = [\mathrm{A^+Cl^-}] \tag{2}$$

$$K_{IPD}[\mathrm{A^+Cl^-}] = [\mathrm{A^+}][\mathrm{Cl^-}] \tag{3}$$

$$K_1[\mathbf{2a}] = [\mathrm{A^+}][\mathrm{Cl^-}] \tag{4}$$

$$K_2[\mathbf{1k}\cdot\mathrm{Cl}^-] = [\mathbf{1k}][\mathrm{Cl}^-] \quad (5)$$

The catalyst bearing H-bonded chloride anion, [**1k**·Cl]$^-$ exchanges its anion at, near, or in, the solution-surface boundary of the solid reagent {NaN_3}/{NaCl}. The H-bonded azide anion, [**1k**·N_3]$^-$ arising from the ion exchange (k_3 or K_3) then reacts in solution, or at, near, or in, the phase boundary, with free aziridinium ion, to generate the product **3a**. In models A and B, anion exchange (K_3) is at equilibrium in the phase boundary, leading to an azide-bound mol-fraction (x_{N3}) of the catalyst-anion eqn (6) and (7). The quantity of [**1k**·X]$^-$ present in the phase boundary is assumed to be low enough to not impact on the solution equilibria (eqn (5)). The reaction product **3a** is produced in the turnover-limiting event by reaction of [**1k**·N_3]$^-$ with either the fully dissociated aziridinium ion (A^+, model A), or with ion-paired aziridinium ([A^+Cl^-], model B). In model C, the anion exchange (k_3) is the turnover-limiting event, and the reaction of [**1k**·N_3]$^-$ with either the fully dissociated or ion-paired aziridinium ion is rapid. Analytical solutions[27] to the cubic equation for concentrations of all species arising in competitive binding of two ligands (in this case A^+ and **1k**) for one species (in this case Cl^-) were then applied (eqn (8) to (17)) to explore conditions relevant to the kinetics, where [**2a**]$_{tot}$ = 0 to 0.25 M, [**1k**] $_{tot}$ = 0 to 10 mol %, and the extent of ionization to generate free ions (Cl^- and A^+) was constrained to be ≤0.1 % at [**2a**]$_{tot}$ = 0.25 M ($K_1 \leq 2.5 \times 10^{-7}$ M) in the absence of catalyst **1k** (eqn (17) and (18)). The background reaction for models A to C involves an analogous process in which chloride ion, liberated via K_1, exchanges with azide ion, and thus generates **3a** by reaction with [A^+Cl^-] or A^+.

$$K_3[\mathbf{1k}\cdot\mathrm{Cl}^-]\{\mathrm{NaN_3}\} = [\mathbf{1k}\cdot\mathrm{N_3}]\{\mathrm{NaCl}\} \quad (6)$$

$$x_{N3} = \frac{1}{1 + \dfrac{\{\mathrm{NaCl}\}}{K_3\,\{\mathrm{NaN_3}\}}} \quad (7)$$

$$[\mathbf{2a}] = \frac{d}{3} + \frac{2}{3}\sqrt{(d^2 - 3e)}cos\frac{\theta}{3} \quad (8)$$

$$[\mathrm{Cl}^-] = \frac{[\mathbf{2a}]_{tot}\{2\sqrt{(d^2-3e)}\cos(\emptyset/3) - d\}}{3K_1 + \{2\sqrt{(d-3e)}\cos(\emptyset/3) - d\}} \quad (9)$$

$$[\mathrm{A}^+] = [\mathbf{2a}]_{tot} - [\mathbf{2a}] \quad (10)$$

$$[\mathbf{1k}\cdot\mathrm{Cl}]^- = \frac{[\mathbf{1k}]_{tot}\{2\sqrt{(d^2-3e)}\cos(\emptyset/3) - d\}}{3K_2 + \{2\sqrt{(d^2-3e)}\cos(\emptyset/3) - d\}} \quad (11)$$

$$d = K_1 + K_2 + [\mathbf{1}\boldsymbol{k}]_{tot} \quad (12)$$

$$e = K_1K_2 + K_1([\mathbf{1}\boldsymbol{k}]_{tot} - [\mathbf{2a}]_{tot}) \quad (13)$$

$$f = -K_1K_2[\mathbf{2a}]_{tot} \quad (14)$$

$$\emptyset = arccos \frac{-2d^3 + 9de - 27f}{2\sqrt{(d^2 - 3e)^3}} \quad (15)$$

$$[\mathrm{A}^+] = [\mathrm{Cl}^-] = \frac{\sqrt{K_1{}^2 + 4K_1[\mathbf{2a}]_{tot}} - K_1}{2} \quad (16)$$

$$[\mathbf{2a}] = [\mathbf{2a}]_{tot} - [\mathrm{A}^+] \quad (17)$$

Three regimes were explored for models A, B, C in which K_2/K_1 was set to control the ratio of complexed versus free chloride ion, as shown below.

Regime 1.	$[\mathbf{1k}\cdot\mathrm{Cl}]^-/[\mathrm{Cl}^-] = 100$	($K_1/K_2 = 9.20 \times 10^2$); $[\mathbf{1k}\cdot\mathrm{Cl}]^- \approx [\mathrm{A}^+] >> [\mathrm{Cl}^-]$
Regime 2.	$[\mathbf{1k}\cdot\mathrm{Cl}]^-/[\mathrm{Cl}^-] = 1$	($K_1/K_2 = 9.96 \times 10^4$); $[\mathbf{1k}\cdot\mathrm{Cl}]^-$ |= $[\mathrm{Cl}^-] \approx 2[\mathrm{A}^+]$
Regime 3.	$[\mathbf{1k}\cdot\mathrm{Cl}]^-/[\mathrm{Cl}^-] = 0.01$	($K_1/K_2 = 9.96 \times 10^6$); $[\mathrm{A}^+] \approx [\mathrm{Cl}^-] >> [\mathbf{1k}\cdot\mathrm{Cl}]^-$

$$\frac{d[\boldsymbol{3a}]}{dt} \approx k_{rxn}\, x_{N3}[\mathbf{1k}\cdot\mathrm{Cl}^-][\mathrm{A}^+]\,;\ \text{model A} \quad (18)$$

$$\frac{d[\boldsymbol{3a}]}{dt} \approx k_{rxn}\, x_{N3}[\mathbf{1k}\cdot\mathrm{Cl}^-][\mathrm{A}^+\mathrm{Cl}^-];\ \text{model B} \quad (19)$$

$$\frac{d[\boldsymbol{3a}]}{dt} \approx k_{rxn}\, [\mathbf{1k}\cdot\mathrm{Cl}^-]\{\mathrm{NaN_3}\}\,;\ \text{model C} \quad (20)$$

Graphical analysis of models A, B and C, by way of linearization of plots of rate versus $[\mathbf{2a}]^x[\mathbf{1k}]^y$, using the rate relationships given in eqn (18) to (20) with equilibrium concentrations of $[\mathbf{1k}\cdot\mathrm{Cl}]^-$, $[\mathrm{A}^+]$, and $[\mathrm{A}^+\mathrm{Cl}^-]$ calculated from eqn (8) to (11), under regimes 1 to 3, gave the rate laws shown in **Table S11**, entries 1 to 7.

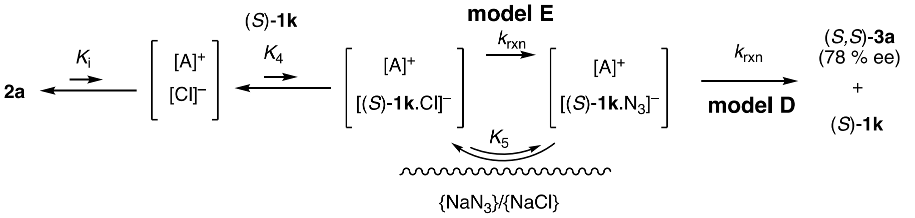

Figure S19 Models D and E, employing eqn (2), (3), and (21) to (26).

In models D and E, **Figure S19**, the catalyst **1k** abstracts chloride ion from the ion pair $[\mathrm{A}^+\mathrm{Cl}^-]$ to generate an equilibrium (K_4) with a second ion-pair containing the aziridinium and the H-bonded anion, [**1k**·**2a**], eqn (21) and (22). The extent of ion pair generation was constrained to be ≤0.1% ($K_i \leq 1 \times 10^{-3}$) in the absence of catalyst **1k**, and the ion paired catalyst constrained to be ≤5 % of the total catalyst ($K_4/K_i \leq 2 \times 10^5$ M^{-1}). In model D, the H-bonded anion-cation pair [**1k**·**2a**] exchanges (K_5) chloride for azide at, near, or in, the phase boundary, to generate an azide-bound mol-fraction

(x_{N3}) eqn (23) and (24), from which product **3a** and **1k** are then liberated. In model E, the anion exchange is irreversible due to rapid generation of **3a** and **1k**.

$$[\mathbf{1k}\cdot\mathbf{2a}] = [\mathbf{2a}]_{tot} - [\mathbf{2a}] \tag{21}$$

$$[\mathbf{1k}\cdot\mathbf{2a}] = \frac{1 + K_i + K_iK_4([\mathbf{2a}]_{tot} + [\mathbf{1k}]_{tot}) - \sqrt{(-1 - K_i - K_iK_4([\mathbf{2a}]_{tot} + [\mathbf{1k}]_{tot}))^2 - 4(K_iK_4)^2[\mathbf{1k}]_{tot}[\mathbf{2a}]_{tot}}}{2K_iK_4} \tag{22}$$

$$K_5[\mathbf{1k}\cdot\mathbf{2a}]\{\mathrm{NaN_3}\} = [\mathbf{1k}\cdot\mathrm{A^+N_3^-}]\{\mathrm{NaCl}\} \tag{23}$$

$$x_{N3} = \frac{1}{1 + \dfrac{\{\mathrm{NaCl}\}}{K_5\,\{\mathrm{NaN_3}\}}} \tag{24}$$

$$\frac{d[\boldsymbol{3a}]}{dt} \approx k_{rxn}\, x_{N3}[\mathbf{1k}\cdot\mathbf{2a}];\ \text{model D} \tag{25}$$

$$\frac{d[\boldsymbol{3a}]}{dt} \approx k_{rxn}[\mathbf{1k}\cdot\mathbf{2a}]\{\mathrm{NaN_3}\}\,;\ \text{model E} \tag{26}$$

Graphical analysis of models D and E, by way of linearization of plots of rate versus $[\mathbf{2a}]^x[\mathbf{1k}]^y$, using the rate relationships given in eqn (25) and (26) gave the rate laws shown in **Table S11**, entries 1 and 8.

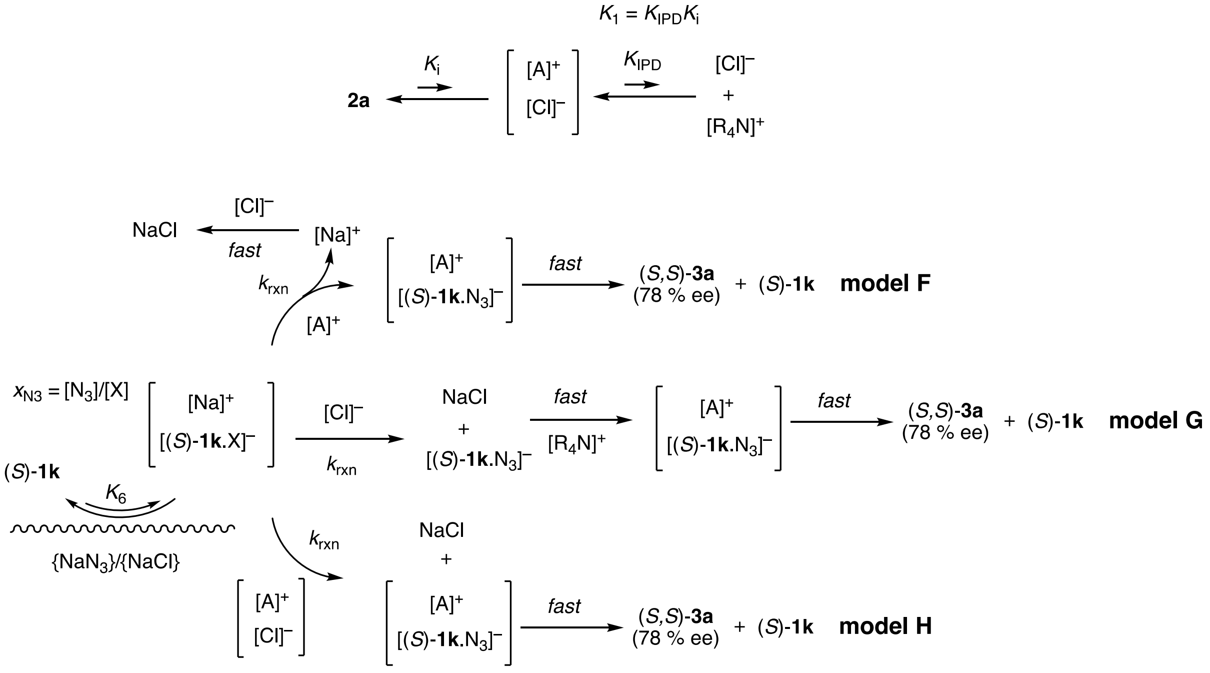

Figure S20 Models F, G and H, employing eqn (2) to (3), eqn (16) to (17), and eqn (27) to (31).

In models F, G, and H, **Figure S20**, the catalyst **1k** complexes with NaN_3 to form an $\{[\mathbf{1k}\cdot N_3^-][Na^+]\}$ ion pair. The anion binding is assumed to be at equilibrium (K_6) in the phase boundary, at a concentration dictated by the common ion Na^+, leading to an azide-bound mol-fraction (x_{N3}) eqn (27) to (28).

$$K_6[\mathbf{1k}]\{\mathrm{NaX}\} = \{[\mathbf{1k}\cdot\mathrm{X^-}][\mathrm{Na^+}]\} \tag{27}$$

$$x_{N3} = \frac{1}{1 + \frac{\{\mathrm{NaCl}\}}{K_6\,\{\mathrm{NaN_3}\}}} \tag{28}$$

In model F, {[**1k**·N_3^-][Na^+]} reacts in solution, or at, near, or in, the phase boundary, with aziridinium ion [A^+], to generate {[**1k**·N_3^-][A^+]} and Na^+; the latter reacts rapidly chloride ion to generate NaCl. The ion pair {[**1k**·N_3^-][A^+]} collapses to generate product **3a** and free catalyst **1k**. In model G, {[**1k**·N_3^-][Na^+]} reacts in solution, or at, near, or in, the phase boundary, with chloride ion [Cl^-] to generate NaCl and [**1k**·N_3]$^-$; the latter reacts rapidly with an aziridinium ion to generate {[**1k**·N_3^-][A^+]} which collapses to generate product **3a** and free catalyst **1k**. In model H, {[**1k**·N_3^-][Na^+]} reacts in solution, or at, near, or in, the phase boundary, with the ion pair [A^+Cl^-] to generate NaCl and the ion pair {[**1k**·N_3^-][A^+]}. The latter collapses to generate product **3a** and free catalyst **1k**.

$$\frac{d[\boldsymbol{3a}]}{dt} \approx k_{rxn}\, x_{N3}\{[\mathbf{1k}\cdot \mathrm{X}^-][\mathrm{Na}^+]\}[\mathrm{A}^+];\ \text{model F} \tag{29}$$

$$\frac{d[\boldsymbol{3a}]}{dt} \approx k_{rxn}\, x_{N3}\{[\mathbf{1k}\cdot \mathrm{X}^-][\mathrm{Na}^+]\}[\mathrm{Cl}^-];\ \text{model G} \tag{30}$$

$$\frac{d[\boldsymbol{3a}]}{dt} \approx k_{rxn}\, x_{N3}\{[\mathbf{1k}\cdot \mathrm{X}^-][\mathrm{Na}^+]\}[\mathrm{A}^+\mathrm{Cl}^-];\ \text{model H} \tag{31}$$

Graphical analysis of models F, G, and H, by way of linearization of plots rate versus [**2a**]^x[**1k**]^y, using concentrations for [A^+], [Cl^-] and [A^+Cl^-] given by eqn (16) and (3), and the rate relationships given in eqn (29) to (31) gave the rate laws shown in **Table S11**, entries 1 and 9.

Table S11 Models A to H and associated rate laws; see text for full discussion.

entry	model	constraint	rate law for catalytic process
1	A and H<br>D	none<br>$K_i \leq 1\times10^{-3}$<br>$K_4/K_i \leq 2\times10^5$	$rate \approx \dfrac{a[\mathbf{2a}]^1[\mathbf{1k}]^1}{1 + \dfrac{b\{\mathrm{NaCl}\}}{\{\mathrm{NaN_3}\}}}$
2	B - regime 1	$[\mathbf{1k}{\cdot}\mathrm{Cl}]^-/[\mathrm{Cl}^-] = 100$	$rate \approx \dfrac{a[\mathbf{2a}]^{1.5}[\mathbf{1k}]^{0.5}}{1 + \dfrac{b\{\mathrm{NaCl}\}}{\{\mathrm{NaN_3}\}}}$
3	B - regime 2	$[\mathbf{1k}{\cdot}\mathrm{Cl}]^-/[\mathrm{Cl}^-] = 1$	$rate \approx \dfrac{a[\mathbf{2a}]^{1.5}[\mathbf{1k}]^{0.75}}{1 + \dfrac{b\{\mathrm{NaCl}\}}{\{\mathrm{NaN_3}\}}}$
4	B - regime 3	$[\mathbf{1k}{\cdot}\mathrm{Cl}]^-/[\mathrm{Cl}^-] = 0.01$	$rate \approx \dfrac{a[\mathbf{2a}]^{1.5}[\mathbf{1k}]^{1}}{1 + \dfrac{b\{\mathrm{NaCl}\}}{\{\mathrm{NaN_3}\}}}$
5	C - regime 1	$[\mathbf{1k}{\cdot}\mathrm{Cl}]^-/[\mathrm{Cl}^-] = 100$	$rate \approx a[\mathbf{2a}]^{0.5}[\mathbf{1k}]^{0.5}\{\mathrm{NaN_3}\}$
6	C - regime 2	$[\mathbf{1k}{\cdot}\mathrm{Cl}]^-/[\mathrm{Cl}^-] = 1$	$rate \approx a[\mathbf{2a}]^{0.5}[\mathbf{1k}]^{0.75}\{\mathrm{NaN_3}\}$
7	C - regime 3	$[\mathbf{1k}{\cdot}\mathrm{Cl}]^-/[\mathrm{Cl}^-] = 0.01$	$rate \approx a[\mathbf{2a}]^{0.5}[\mathbf{1k}]^{1}\{\mathrm{NaN_3}\}$
8	E	$K_i \leq 1\times10^{-3}$<br>$K_4/K_i \leq 2\times10^5$	$rate \approx a[\mathbf{2a}]^{1}[\mathbf{1k}]^{1}\{\mathrm{NaN_3}\}$
9	F and G	none	$rate \approx \dfrac{a[\mathbf{2a}]^{0.5}[\mathbf{1k}]^1}{1 + \dfrac{b\{\mathrm{NaCl}\}}{\{\mathrm{NaN_3}\}}}$

The generic rate laws shown in **Table S11** were evaluated for their ability to correlate temporal concentrations of $[\mathbf{3a}]_t$, and net enantioselectivity, obtained from 8 different starting conditions (initial concentrations of $[\mathbf{2a}]_0$ and $[\mathbf{1k}]_0$), **Figure S21**. The analysis, see **Tables S12-S21** indicates that of models A to H, the best fit is obtained with the equation associated with models F and G (**Table S11**, entry 9) which gives kinetic orders of $[\mathbf{2a}]^{0.5}$ and $[\mathbf{1k}]^1$, progressive inhibition by chloride ion, and the correct enantioselectivity when a background racemic reaction is included, eqn (32). As shown in **Figure S21B**, the rate of the background reactions varies between runs. When fitted with eqn (32) with data points up to 20000 s, c = (1.3 ± 0.5) x 10^{-5}. It is noted that model C, regime 3 (**Table S11**, entry 7) does not account for the progressive inhibition by accumulating {NaCl}, or for how the enantioselective catalyzed process is able to compete with the racemic background reaction in the conversion of **2a** to **3a**, when $[\mathbf{1k}{\cdot}\mathrm{Cl}]^-/[\mathrm{Cl}^-] << 1$.

$$\frac{d[(S,S)\text{-}\mathbf{3a}]}{dt} = [\mathbf{2a}]^{0.5}\left[\frac{0.8882a[\mathbf{1k}]^1}{1 + \frac{b\{\mathrm{NaCl}\}}{\{\mathrm{NaN_3}\}}} + 0.5c\right];\ \frac{d[(R,R)\text{-}\mathbf{3a}]}{dt} = [\mathbf{2a}]^{0.5}\left[\frac{0.1118a[\mathbf{1k}]^1}{1 + \frac{b\{\mathrm{NaCl}\}}{\{\mathrm{NaN_3}\}}} + 0.5c\right] \quad (32)$$

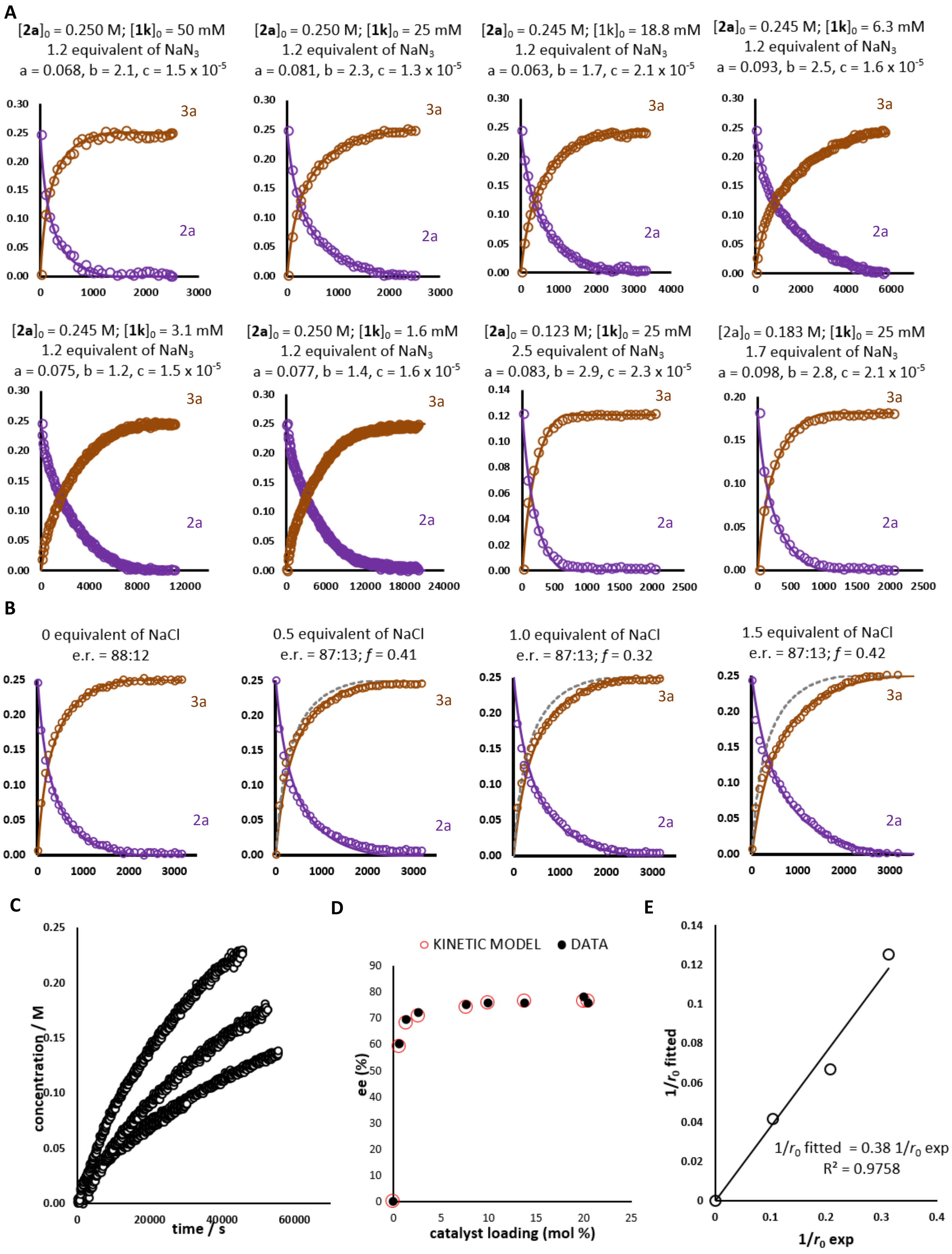

Figure S21 (A) Kinetic simulations (solid lines; employing eqn (32), with values for *a*, *b*, and *c* as shown) of $[\mathbf{2a}]_t$ and, $[\mathbf{3a}]_t$ across a series of variations in initial concentrations of chloride (±**2a**) and bis-urea catalyst ((*S*)-**1k**) as compared to values determined experimentally (open circles; by *in situ* infra-red spectroscopy). (B) The effect of exogenous NaCl ($[\mathbf{2a}]_0$ = 0.250 M; 10 mol% **1k**) using constant values for *a*, *b*, *c* and a fitting parameter *f*. When when $f = 0$ (see dashed line) there is no inhibition, when $f = 1$, exogenous NaCl behaves identically to endogenous. The values are consistent with the exogenous NaCl being of a different 'form' (particle size etc.) to the endogenous NaCl' however pre-grinding

NaCl with NaN_3 was considered too hazardous. (C) Concentration profile for background reactions, [**2a**]$_0$ = 0.250 M, [**1k**]$_0$ = 0 M in 1,2-difluorobenzene. (D) Enantiomeric excess of product at different catalyst loading predicted by kinetic simulations (open red circle; employing eqn (32) as compared to values determined experimentally (black circle; by chiral HPLC after prep TLC). (E) correlation between $1/r_0$ fitted and $1/r_0$ exp, where $1/r_0$ = initial NaCl/NaN_3; the gradient is the average fitting parameter *f*.

A series of rate equations with different order in catalyst and substrate concentration, with (eqn (33)) or without (eqn (34)) chloride inhibition, were fitted to the experimental data by using Excel Solver (setting *a*, *b* and *c* as variables; then minimizing the sum of least squares of difference in experimental and simulated values of concentration of substrate and two enantiomers of product). The results are summarized in **Table S12**, with fitting details shown in **Tables S13 to S21**.

$$\frac{d[\mathbf{3a}]}{dt} \approx [\mathbf{2a}]^x \left[\frac{a[\mathbf{1k}]^y}{1 + \frac{b\{\mathrm{NaCl}\}}{\{\mathrm{NaN_3}\}}} + c \right] \tag{33}$$

$$\frac{d[\mathbf{3a}]}{dt} \approx [\mathbf{2a}]^x \left[\frac{a[\mathbf{1k}]^y}{1 + \frac{b}{\{\mathrm{NaN_3}\}}} + c \right] \tag{34}$$

Table S12 Values of *a*, *b* and *c* that provide best fit (Excel Solver) equations with different order in catalyst (**1k**) and substrate (**2a**) concentration, with (eqn (33)) or without (eqn (34)) chloride inhibition.

entry	[**2a**]^x (*x*)	[**1k**]^y (*y*)	rate eqn	*a*	*b*	*c* x 10^5
**1**	**0.5**	**1**	**eqn (33)**	**0.081 ± 0.018 (22%)**	**2.1± 0.9 (43%)**	**1.8 ± 0.5 (28%)**
2	1	1	eqn (33)	0.17 ± 0.06 (35%)	0.32 ± 0.32 (100%)	1.7 ± 0.3 (18%)
3	1.5	0.5	eqn (33)	0.078 ± 0.062 (79%)	0	0.75 ± 0.75 (100%)
4	1.5	0.75	eqn (33)	0.21 ± 0.13 (62%)	0	0.75 ± 0.75 (100%)
5	1.5	1	eqn (33)	0.6 ± 0.3 (50%)	0	0.85 ± 0.85 (100%)
6	0.5	0.5	eqn (34)	/	/	/
7	0.5	0.75	eqn (34)	340 ± 310 (91%)	5800 ± 5300 (35%)	1.1 ± 1.1 (100%)
8	0.5	1	eqn (34)	0.25 ± 0.11 (44%)	0.25 ± 0.25 (100%)	1.6 ± 0.2 (13%)
9	1	1	eqn (34)	0.26 ± 0.12 (46%)	0.27 ± 0.27 (100%)	1.7 ± 0.3 (15%)

Table S13 Best-fit values of *a*, *b* and *c* employed for error analysis **Table S12**, entry 1.

$$\frac{d[\mathbf{3a}]}{dt} \approx [\mathbf{2a}]^{0.5}\left[\frac{a[\mathbf{1k}]^{1}}{1 + \frac{b\{\mathrm{NaCl}\}}{\{\mathrm{NaN_3}\}}} + c\right]$$

entry	a	b	c x10^5	ex	ee	exp ee
1	0.068	2.1	1.5	0.2	76.5	78
2	0.081	2.3	1.3	0.2	75.9	76
3	0.063	1.7	2.1	0.22	74.0	75
4	0.093	2.5	1.6	0.22	70.8	72
5	0.075	1.2	1.5	0.22	68.2	69.5
6	0.077	1.4	1.6	0.2	59.1	60
8	0.083	2.9	2.3	1.48	76.1	76
9	0.098	2.8	2.1	0.66	76.1	76

Table S14 Best-fit values of *a*, *b* and *c* employed for error analysis **Table S12**, entry 2.

$$\frac{d[\mathbf{3a}]}{dt} \approx [\mathbf{2a}]^{1}\left[\frac{a[\mathbf{1k}]^{1}}{1 + \frac{b\{\mathrm{NaCl}\}}{\{\mathrm{NaN_3}\}}} + c\right]$$

entry	a	b	c x 10^5	ex	ee	exp ee
1	0.11	0.43	1.4	0.2	76.5	78
2	0.14	0.64	1.4	0.2	75.6	76
3	0.11	0.23	1.8	0.22	74.2	75
4	0.14	0.35	1.4	0.22	71.9	72
5	0.14	0.034	1.4	0.22	68.1	69.5
6	0.14	0.0085	1.8	0.2	55.8	60
8	0.22	0	1.7	1.48	76.5	76
9	0.20	0.42	1.9	0.66	76.1	76

An error in solver is encountered if the restriction b >1 is made; manual variation of initial fitting required.

Table S15 Best-fit values of *a*, *b* and *c* employed for error analysis **Table S12**, entry 3.

$$\frac{d[\mathbf{3a}]}{dt} \approx [\mathbf{2a}]^{1.5}\left[\frac{a[\mathbf{1k}]^{0.5}}{1 + \frac{b\{\mathrm{NaCl}\}}{\{\mathrm{NaN_3}\}}} + c\right]$$

entry	a	b	c x 10^5	ex	ee	exp ee
1	0.056	0	0	0.2	76.6	78
2	0.043	0	1.4	0.2	75.7	76
3	0.037	0	0.75	0.22	76.1	75
4	0.026	0	1.0	0.22	73.3	72
5	0.022	0	0.98	0.22	70.0	69.5
6	0.016	0	1.5	0.2	55.3	60
8	0.14	0	0	1.48	77.6	76
9	0.089	0	0	0.66	77.6	76

poor correlation with experimental data.

Table S16 Best-fit values of *a*, *b* and *c* employed for error analysis **Table S12**, entry 4.

$$\frac{d[\mathbf{3a}]}{dt} \approx [\mathbf{2a}]^{1.5}\left[\frac{a[\mathbf{1k}]^{0.75}}{1 + \frac{b\{\mathrm{NaCl}\}}{\{\mathrm{NaN_3}\}}} + c\right]$$

entry	a	b	c x 10^5	ex	ee	exp ee
1	0.12	0	0	0.2	77.6	78
2	0.11	0	1.4	0.2	75.7	76
3	0.10	0	0.75	0.22	76.1	75
4	0.093	0	1.0	0.22	73.0	72
5	0.091	0	0.93	0.22	70.3	69.5
6	0.082	0	1.5	0.2	55.4	60
8	0.34	0	0	1.48	77.6	76
9	0.23	0	0	0.66	77.6	76

poor correlation with experimental data.

Table S17 Best-fit values of *a*, *b* and *c* employed for error analysis **Table S12**, entry 5.

$$\frac{d[\mathbf{3a}]}{dt} \approx [\mathbf{2a}]^{1.5}\left[\frac{a[\mathbf{1k}]^{1}}{1 + \frac{b\{\mathrm{NaCl}\}}{\{\mathrm{NaN_3}\}}} + c\right]$$

entry	a	b	c x 10^5	ex	ee	exp ee
1	0.25	0	0	0.2	76.6	78
2	0.27	0	1.4	0.2	75.7	76
3	0.27	0	0.7	0.22	74.2	75
4	0.33	0	1.0	0.22	73.0	72
5	0.39	0	1.0	0.22	70.0	69.5
6	0.41	0	1.5	0.2	55.5	60
8	0.86	0	0	1.48	77.6	76
9	0.57	0	0	0.66	77.6	76

poor correlation with experimental data.

Table S18 Best-fit values of *a*, *b* and *c* employed for error analysis **Table S12**, entry 6.

$$\frac{d[\mathbf{3a}]}{dt} \approx [\mathbf{2a}]^{0.5}\left[\frac{a[\mathbf{1k}]^{0.5}}{1 + \frac{b}{\{\mathrm{NaN_3}\}}} + c\right]$$

entry	a	b	c x 10^5	ex	ee	exp ee
1				0.2		78
2	12	420	1.2	0.2	76.2	76
3				0.22		75
4				0.22		72
5				0.22		69.5
6	6.5	721	1.8	0.2	57.3	60
8				1.48		76
9				0.66		76

1,3,4,5,8,9: do not converge or run into an error

Table S19 Best-fit values of *a*, *b* and *c* employed for error analysis **Table S12**, entry 7.

$$\frac{d[\mathbf{3a}]}{dt} \approx [\mathbf{2a}]^{0.5}\left[\frac{a[\mathbf{1k}]^{0.75}}{1 + \frac{b}{\{\mathrm{NaN_3}\}}} + c\right]$$

entry	a	b	c x 10^5	ex	ee	exp ee
1	310	4000	0	0.2	77.6	78
2	38	540	1.5	0.2	75.8	76
3	39	630	2.1	0.22	74.3	75
4	640	11000	1.5	0.22	71.8	72
5	37	680	1.5	0.22	68.5	69.5
6	33	730	1.8	0.2	58.0	60
8	47	500	2.1	1.48	76.5	76
9				0.66		76

4: poor correlation with experimental data. 9: run into an error.

Table S20 Best-fit values of *a*, *b* and *c* employed for error analysis **Table S12**, entry 8.

$$\frac{d[\mathbf{3a}]}{dt} \approx [\mathbf{2a}]^{0.5}\left[\frac{a[\mathbf{1k}]^{1}}{1 + \frac{b}{\{\mathrm{NaN_3}\}}} + c\right]$$

entry	a	b	c × 10^5	ex	ee	exp ee
1				0.2		78
2	0.36	0.49	1.5	0.2	75.6	76
3	0.14	0.08	1.8	0.22	74.2	75
4	0.21	0.15	1.4	0.22	71.8	72
5	0.14	0.0093	1.4	0.22	68.1	69.5
6	0.14	0.0015	1.8	0.2	55.9	60
8	0.21	0	1.4	1.48	76.6	76
9	0.31	0.19	1.8	0.66	76.1	76

An error in solver is encountered if the restriction b >1 is made; manual variation of initial fitting required.

Table S21 Best-fit values of *a*, *b* and *c* employed for error analysis Table S12, entry 9.

$$\frac{d[\mathbf{3a}]}{dt} \approx [\mathbf{2a}]^1 \left[\frac{a[\mathbf{1k}]^1}{1 + \frac{b}{\{\mathrm{NaN_3}\}}} + c \right]$$

entry	a	b	c × 10^5	ex	ee	exp ee
1	0.20	0.23	1.7	0.2	76.3	78
2	0.38	0.54	1.5	0.2	75.6	76
3	0.14	0.088	1.8	0.22	74.2	75
4	0.21	0.16	1.4	0.22	71.9	72
5	0.14	0.011	1.4	0.22	68.1	69.5
6	0.14	0.0026	1.8	0.2	55.8	60
8	0.22	0	1.7	1.48	76.5	76
9	0.34	0.21	1.9	0.66	76.1	76

An error in solver is encountered if the restriction b >1 is made; manual variation of initial fitting required.

Table S22 Best-fit values of *a*, *b* and *c* employed for error analysis Table S12, entry 10.

$$\mathrm{rate} = [\mathrm{S}]^{1.5} \left[\frac{\mathrm{a}\,[\mathrm{Cat}]_0^1}{1 + \frac{\mathrm{b}}{\mathrm{ex}[\mathrm{S}]_0 + [\mathrm{S}]_\mathrm{t}}} + \mathrm{c} \right]$$

entry	a	b	c × 10^5	ex	ee	exp ee
1	0.26	0.00065	1.4	0.2	76.5	78
2	0.27	0	1.4	0.2	75.6	76
3	0.28	0.00080	1.4	0.22	74.8	75
4	0.34	0.00061	1.4	0.22	71.2	72
5	0.41	0.00094	1.4	0.22	67.3	69.5
6	0.41	0	1.5	0.2	55.5	60
8	0.86	0	0	1.48	77.6	76
9	0.57	0	0	0.66	77.6	76

An error in solver is encountered if the restriction b >1 is made; manual variation of initial fitting required.

Control experiments with additional NaCl

The rate of the model reaction was monitored in the presence of increasing quantities of NaCl.

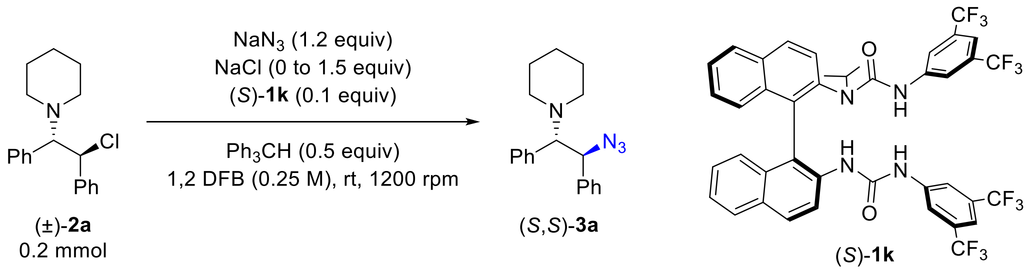

entry	NaCl	conversion	e.r.
1	0 equiv	>99%	88:12
2	0.5 equiv	>99%	87:13
3	1 equiv	>99%	87:13
4	1.5 equiv	>99%	87:13

[a]Mean of two runs.

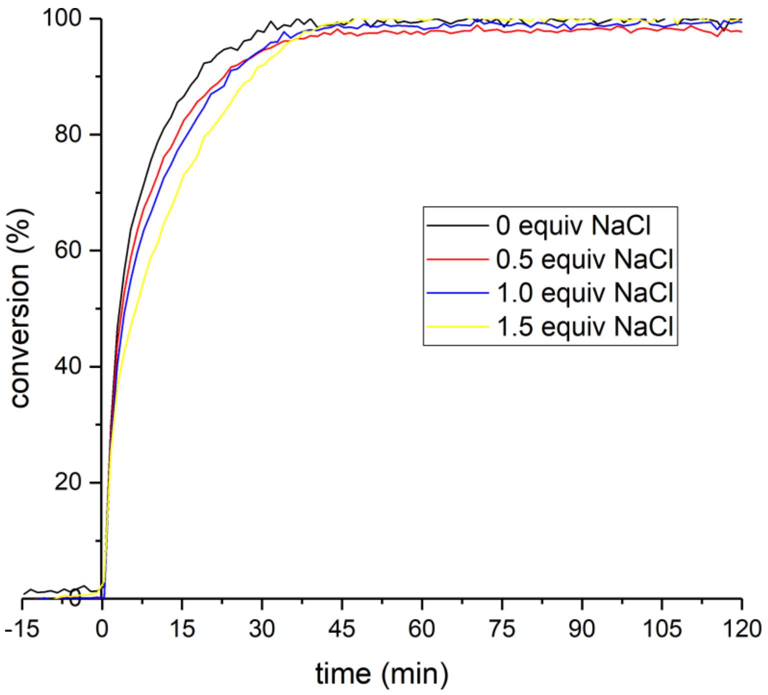

Computational Methods

We performed all geometry optimizations and frequency calculations with Gaussian 09, Revision D.01.[28] We used Truhlar's meta-hybrid M06-2X functional[29] and mixed basis sets from the Ahlrich family. The triple-zeta def2-TZVPD basis set was used for all heteroatoms without the diffuse function for fluorine (def2-TZVP).[30,31] Carbon and hydrogen atoms were described by the double-zeta def2-SVP basis set.[30] We employed the ultrafine grid with the int=ultrafine keyword and considered solvent effects implemented in the CPCM solvent model for CH_2Cl_2.[32,33]

Single point energies were obtained with ORCA 4.2.0[34] utilizing the range separated ωB97x-D3 functional[35] including Grimme's D3 dispersion correction[36] along with the ma-def2-TZVPP[37] basis set on heteroatoms and the def2-TZVPP basis set on C,H. As for optimizations, solvent effects were accounted for by employing the CPCM solvent model for CH_2Cl_2. Weinhold's Natural Bond Orbital analysis was performed with NBO version 6.0.[38]

Thermochemical corrections were applied using GoodVibes,[39] following Grimme's recommendation[40] of a free-rotor to describe low-frequency normal modes. A cut-off value of 100 cm^{-1} was used. Gibbs energies were evaluated at 298.15 K and 1 M concentration unless stated otherwise.

The protocol for obtaining optimized geometries and their corresponding electronic and Gibbs Free energies are based upon previous work in our group and have been validated *vide infra* for the systems discussed within the framework of this paper.[8,15]

We performed conformational sampling of the pre-transition states by utilizing Grimme's **C**onformer-**R**otamer **E**nsemble **S**ampling **T**ool (CREST) versions 2.6 and 2.8.[41,42] Here, the conformer/rotamer ensemble is obtained by implementation of the iMTD-GC workflow, where semi-empirical DFT calculations based on the tight-binding approach GFN-xTB (**g**eometry, **f**requency, **n**on-covalent interactions, e**x**tended **t**ight **b**inding) are combined with meta-dynamics (MTD) and genetic Z-matrix crossing (GC). The collective variables are defined by a biasing Gaussian-type potential, representing previously located minima on the potential energy hypersurface.

NCIPLOT version 4.0 was used to generate promolecular densities from which the reduced density gradient (RDG) isosurface was generated (RDG = 0.5) with color code reflecting the value of sign(λ2)ρ (-0.03 blue, 0.03 red).[43,44]

Method Validation

Density Functional: We assessed the M06-2X functional for a model complex consisting of the azide anion H-bonded to two unsubstituted urea motifs and evaluated key geometric parameters against an MP2[45] benchmark (**Table S23**). We found good agreement for all H-bond distances and one of the dihedral angles and a moderate accuracy for the remaining dihedral angle between DFT and MP2.

Table S23 Comparison of key parameters for a model urea-azide system.

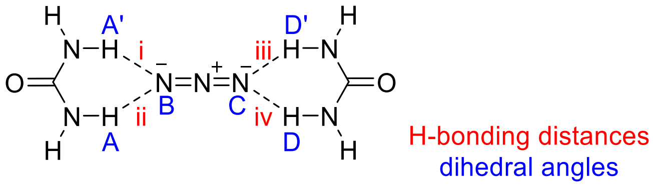

parameter	MP2	M06-2X
i,ii,iii,iv	2.06 Å	2.09 Å
θ(ABCD)	155.2°	148.1°
θ(A'BCD')	3.3°	24.7°

M06-2X/def2SVP(TZVPPD)/CPCM(CH_2Cl_2) and MP2/def2-TZVPD/CPCM(CH_2Cl_2)

Basis set: We found a strong dependence of the choice of the basis set within Ahlrich's def2-family on the geometric arrangement of the model system (**Table S24**). While the H bond distances are largely unaffected, the two dihedral angles do vary. BS2 and BS8 show the largest deviation for these two parameters, with the former exhibiting a complete lack of diffuse functions (def2-SVP (C,H); def2-TZVP (O,N)) and the latter lacking triple zeta basis sets (def2-SVP (C,H); def2-SVPD (O,N)). Less severe deviation from the dihedral angle obtained with BS1 (def2-SVP(TZVPPD)), was observed when employing BS3-5 which all contain a triple zeta basis set with diffuse functions on N (def2-TZVPPD (N)) BS3 does contain any diffuse functions on C,H,O however a triple zeta basis set is used on O (def2-SVP (C,H); def2-TZVP (O); def2-TZVPPD (N)). BS4 differs from BS3 by removal of the triple zeta treatment for O (def2-SVP (C,H,O); def2-TZVPPD (N)) and finally, the triple zeta treatment on O is removed and substituted for by a less costly split valence treatment including diffuse functions in BS5 (def2-SVP (C,H); def2-SVPD (O); def2-TZVPPD (N)). This highlights the importance of treating the heteroatoms with triple zeta valence basis sets and diffuse functions. BS6 and BS7 differ from BS1 on the selective absence of doubly polarized basis sets on the heteroatoms (BS6: def2-SVP (C,H); def2-TZVPD (O); def2-TZVPPD (N); BS7: def2-SVP (C,H); def2-TZVPD (O,N)) with BS7 still giving identical results to BS1.

Table S24 Comparison of key parameters for a model urea-azide system based on choice of the basis set.

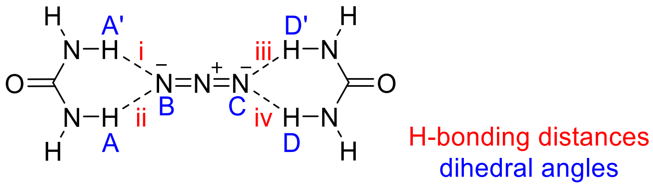

M062X	BS1	BS2	BS3	BS4	BS5	BS6	BS7	BS8
i,ii,iii,iv	2.09 Å	2.08 Å	2.09 Å	2.09 Å	2.09 Å	2.09 Å	2.09 Å	2.07 Å
θ(ABCD)	148.1°	91.2°	128.3°	118.4°	116.7°	148.1°	148.1°	84.5°
θ(A'BCD')	24.7°	89.8°	20.1°	4.8°	14.0°	24.7°	24.7°	83.5°

Method: g09 (rev.D.01) M062X /CPCM(CH_2Cl_2). Basis sets: BS1 (def2-SVP(TZVPPD)); BS2 (def2-SVP (C,H); def2-TZVP (O,N)); BS3 (def2-SVP (C,H); def2-TZVP (O); def2-TZVPPD (N)); BS4 (def2-SVP (C,H,O); def2-TZVPPD (N)); BS5 def2-SVP (C,H); def2-SVPD (O); def2-TZVPPD (N)); BS6 (def2-SVP (C,H); def2-TZVPD (O); def2-TZVPPD (N)); BS7 (def2-SVP (C,H); def2-TZVPD (O,N)); BS8 def2-SVP (C,H); def2-SVPD (O,N)).

Therefore, we chose BS7 as the basis set combination for our geometry optimizations.

Unlike our model system, both the experimentally employed achiral Schreiner's Urea **1a** and the chiral (*S*)-**1k** catalyst contain fluorine atoms. To investigate the effects of modifications at the basis set level, such as the necessity for diffuse functions, we optimized the identical [(*S*)-**1k**·N_3]$^-$ complex (conf10, see below) with different basis sets on fluorine (**Figure S22**).

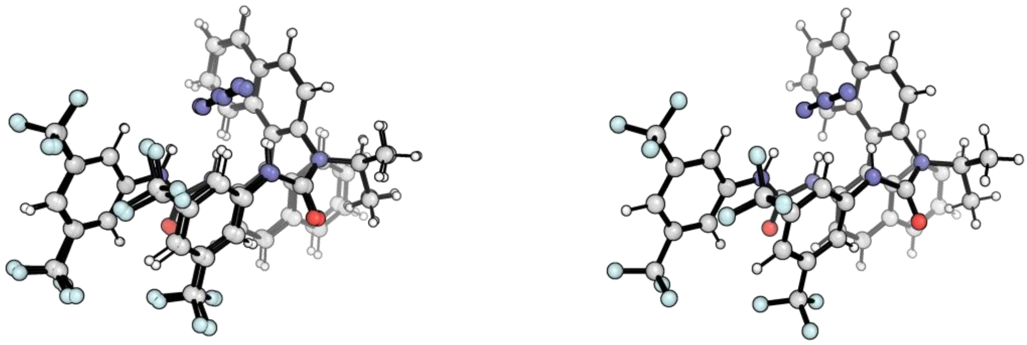

Figure S22 Superposition of the [(*S*)-**1k**·N_3]$^-$ conformer conf10 (see below), optimized with different basis sets on F. Left-hand side: def2-TZVPPD vs def2-SVP on F; Right hand side: def2-TZVPPD vs def2-TZVP on F. Method: g09 (rev.D.01) M062X/def2-SVP(C,H)/def2-TZVPD (N,O)/CPCM(CH_2Cl_2)

Following our investigation on the effects of the choice of basis set on the geometries of the discussed structures, we decided to use the M062X / def2-SVP (C,H) / def2-TZVP (F) / def2-TZVPD (N,O,Cl) level of theory for geometry optimizations, providing the best accuracy/time ratio of the studied combinations.

Integration grid: An ultrafine grid was used throughout. Using a fine grid for numerical integration, while being a factor of 3.1 times faster for geometry optimization, led to changes (e.g. to dihedral angles by several degrees) to the optimized structures, so we used a more expensive ultrafine grid.

Single Point Energies: Electronic energies from single point calculations were obtained via the ωB97x-D3 / (ma)-def2-TZVPP (N,O,F,Cl) / def2-TZVPP (C,H) level of theory as implemented in ORCA 4.2.0 and validated on our model system against the DLPNO-CCSD(T)[46] / aug-cc-pVTZ[47,48] / aug-cc-pVTZ/C / RIJCOSX[49] / def2/J[50] / TIGHTSCF standard. Our DFT binding energetics of azide with a urea moiety were within 5.4 kJ mol^{-1} (1.3 kcal mol^{-1}) of the DLPNO-CCSD(T) reference (**Table S25**). A gas-phase comparison gave similar results, with the difference reduced to 4.9 kJ mol^{-1}.

Table S25 Comparison of electronic binding energies for a model urea-azide system to validate the choice of the method in ORCA 4.2.0.

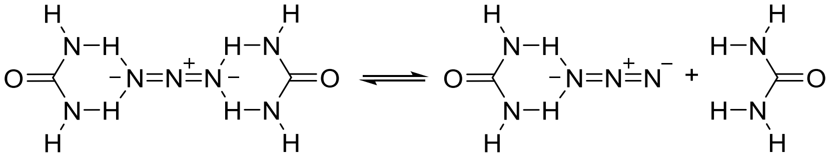

	DLPNO-CCSD(T)[a]	ωB97x-D3[b]
ΔE_{diss}	29.6	24.2

a. DLPNO-CCSD(T) / aug-cc-pVTZ / aug-cc-pVTZ/C / RIJCOSX / def2/J / TIGHTSCF / CPCM(CH_2Cl_2); b. ωB97x-D3 / (ma)-def2-TZVPP (N,O) / def2-TZVPP (C,H) / CPCM(CH_2Cl_2).

Solute cavity: We found a van der Waals solute cavity within the CPCM solvent model for $CH_2Cl_2$to be necessary. Use of default solvent-excluded surface-type solute cavity in ORCA 4.2.0 led to significant variations in conformer energies (**Table S26**) due to inconsistent smearing of surface charges.[51] Single point energies obtained on geometries optimized with different basis set mixtures, BS1 and BS7, are either identical or agree within less than 0.7 kJ mol^{-1} difference for the same conformer, which further corroborates the suitability of BS7 for geometry optimization.

Table S26 Relative energies of [(*S*)-**1k**·N_3]$^-$.

conf	$\Delta E_{CPCM(CH2Cl2)}$[a]	$\Delta E_{CPCM(CH2Cl2)}$[b] vdW surfacetype	$\Delta E_{CPCM(CH2Cl2)}$[c] vdW surfacetype
conf1	-109.5	15.0	15.4
conf2	21.6	20.6	21.1
conf3	17.5	16.5	16.5
conf4	13.2	11.0	11.1
conf5	28.5	28.4	29.1
conf6	6.3	7.0	7.1
conf7	17.2	17.4	17.3
conf8	16.2	16.3	16.3
conf9	0	0	0
conf10	13.9	14.1	14.1
conf11	16.5	17.4	17.3
conf12	16.2	16.2	16.3

Single point energies obtained via a: ωB97x-D3 / (ma)-def2-TZVPP (N,O) / def2-TZVPP (C,H) / CPCM(CH_2Cl_2), default settings for CPCM, b and c: ωB97x-D3 / (ma)-def2-TZVPP (N,O) / def2-TZVPP (C,H) / CPCM(CH_2Cl_2), vdW surface for CPCM. For a and b, structures were optimized via the M06-2X/BS1/(CPCM(CH_2Cl_2) level of theory, for c the optimization was performed at the M06-2X/BS7/(CPCM(CH_2Cl_2) level of theory, see above.

[(*S*)-1k·N_3]$^-$ conformers

CREST conformational sampling was performed in CH_2Cl_2. The 12 highest weighted structures (>0.01% weighting) were further optimized using DFT, giving ten unique conformers (**Figure S23**). We classified these structures into three categories, according to their type of binding between azide and the three N-H bond donors of the catalyst backbone, accompanied by a distinct angular orientation of the nucleophile, as discussed in the manuscript.

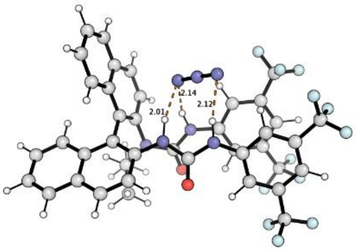
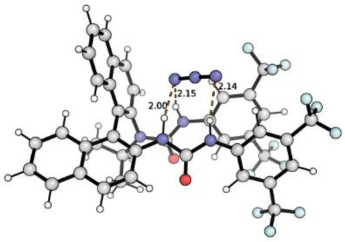
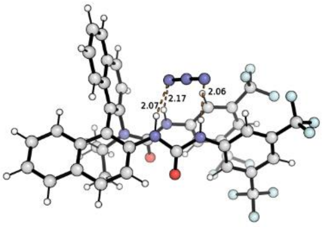

conf1: ΔΔG = 15.8 kJ mol^{-1} conf2: ΔΔG = 20.8 kJ mol^{-1} conf3: ΔΔG = 16.0 kJ mol^{-1}

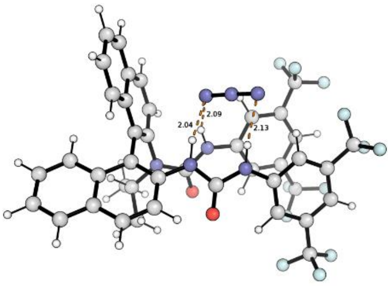
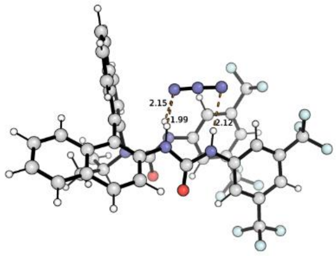
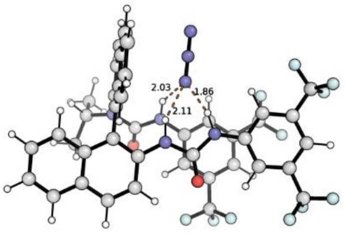

conf4: ΔΔG = 8.6 kJ mol^{-1} conf5: ΔΔG = 27.8 kJ mol^{-1} conf6: ΔΔG = 1.6 kJ mol^{-1}

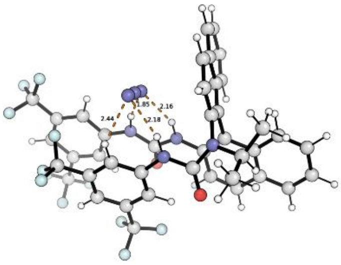
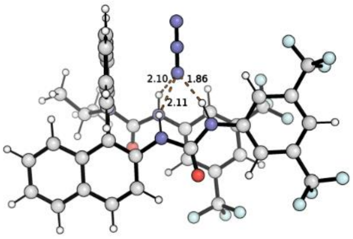
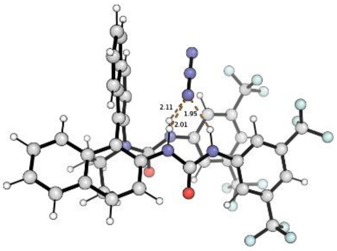

conf7: ΔΔG = 16.4 kJ mol^{-1} conf8: ΔΔG = 12.8 kJ mol^{-1} conf9: ΔΔG = 0 kJ mol^{-1}

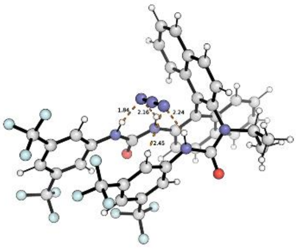

conf10: ΔΔG = 10.2 kJ mol^{-1}

Figure S23 [(*S*)-**1k**·N_3]$^-$ conformers with relative DFT-computed relative Gibbs Energies.

The results from our natural bond orbital analysis for the energetically lowest conformers of each binding type suggest that the H-bonding interaction between the catalyst and the nucleophile has a direct effect on the electron distribution of the latter, polarizing the quadrupole moment of the azide anion while charge transfer occurs from azide to the catalyst (**Table S27**). This is most pronounced for the tripodal binding of conf9. The observed geometric feature of angular promiscuity can therefore be associated with distinct electronic features, depending on the nature of the coordination sphere. The strength of the H bonding interaction, however, between the nucleophile and the catalyst backbone remains similar, irrespective of the azide's geometric

arrangement, and is therefore not considered as the decisive factor for the energy differences between the investigated conformers.

Table S27 Key properties of the most stable conformer of each type and their comparison with free azide.

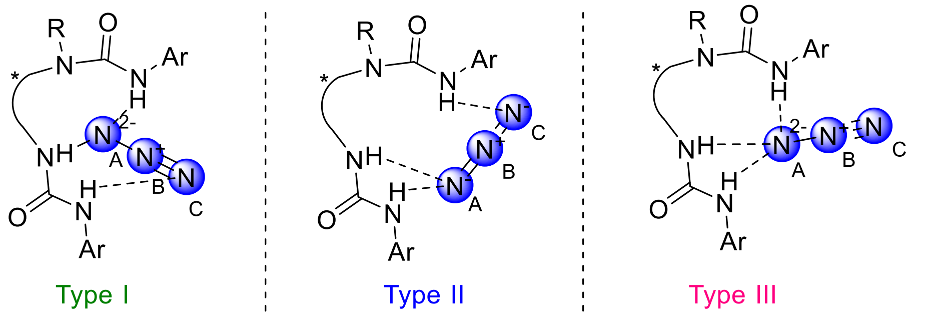

type	charge distribution	charge transfer
I (conf4)	N_A : -0.57<br>N_B : +0.20<br>N_C : -0.52	10.8%
II (conf10)	N_A : -0.58<br>N_B : +0.21<br>N_C : -0.50	11.9%
III (conf9)	N_A : -0.65<br>N_B : +0.22<br>N_C : -0.47	11.0%
free azide	N_A : -0.59<br>N_B : +0.18<br>N_C : -0.59	N/A

Urea 1a-azide conformers

We manually located three distinct low-lying conformers, representing different binding modes of azide with the achiral Schreiner's Urea HBD (1a), as discussed in the main manuscript.

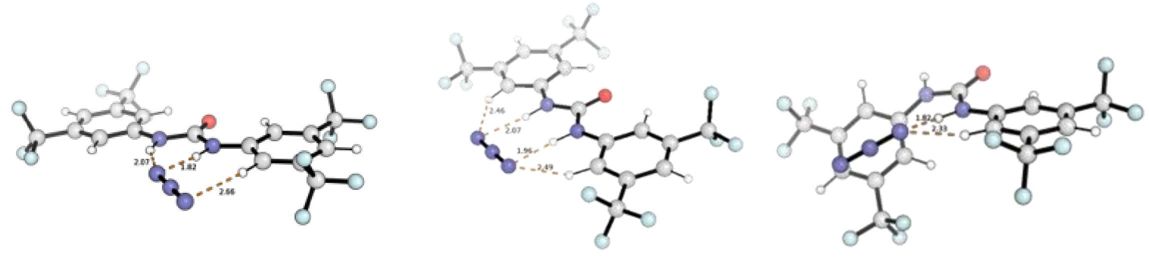

SUAconfA: 0.0 kJ/mol SUAconfB: 3.5 kJ/mol SUAconfC: 21.2 kJ/mol

Figure S25. Schreiner's Urea-azide confomers with relative Gibbs Free Energies.

For the close contact ion pairs preceding the corresponding achiral azidation transition states, we located three energetically relevant conformers. Structurally, we see two end-on coformers

(IPconfA and IPconfC) and one-side-on arrangement (IPconfB). The relative energy of IPconfC suggests that the decisive interaction takes place between azide and Schreiner's Urea, dominating potential hydrogen-bonding interactions between the azide and the quarternary ammonium electrophile.

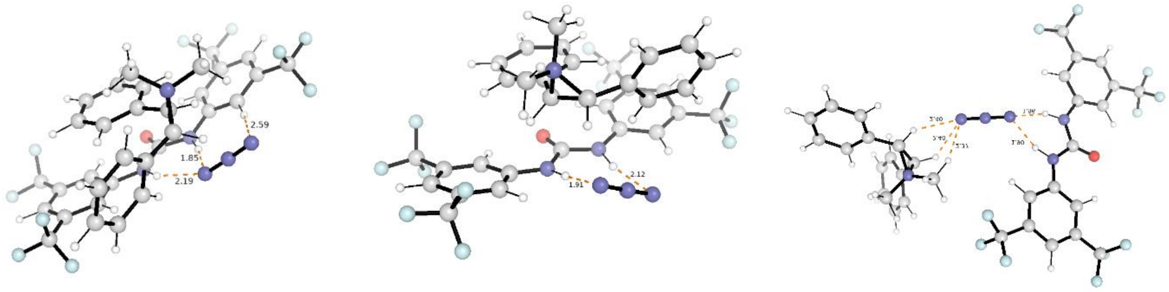

IPconfA: 0.0 kJ/mol IPconfB: 8.6 kJ/mol IPconfC: 21.9 kJ/mol

Figure S26. Close contact ion pairs with relative Gibbs Free Energies.

While the side-on arrangement (IPconfB) was energetically comparable to the lowest located ion pair conformer, as described in the preceding section, the corresponding TS (TS-C') is the energetically highest lying among the located transition states. The two end-on conformers, were very similar in energy, with an energetic difference as small as 2.0 kJ/mol depending on attack of the terminal, unbound N (TS-A') or of the hydrogen bond coordinated N (TS-B').

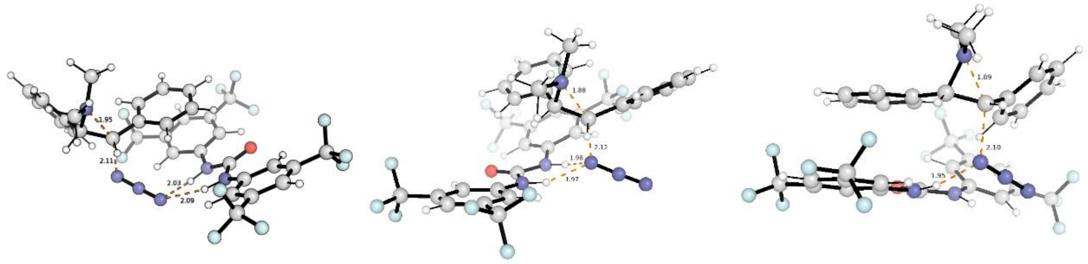

TS-A': 0.0 kJ/mol TS-B': 2.0 kJ/mol TS-C': 16.5 kJ/mol

Figure S27. Non-asymmetric transition state structures with relative Gibbs Free Energies.

Transition state structures and enantioselectivity

Input structures for subsequent conformational sampling were obtained by manually locating one central and one minor transition state at the M062X / def2-SVP (C,H) / def2-TZVP (F) / def2-TZVPD (N,O) level of theory. A constrained search with CREST was then performed to locate multiple conformers for each enantiomer - four atoms were constrained, with a default force constant of 0.5. These atoms were selected, as their relative geometric arrangement defines the distance

between the attacking N_{azide} and the electrophilic carbon C^1, the degree of ring-opening of the aziridinium (both via the bond angle of C^1-C^2-NMe_2^+ and the fixation of the relevant distances within the three-membered ring) as well as the dihedral angle N_{azide}-C^1-C^2-NMe_2.

We obtained 168 major and 219 minor structures and focussed on the 100 most stable structures for each diastereomeric parent structure. We removed duplicates (criteria were applied after visual inspection: Room-Mean-Square-Deviation (RMSD) <0.08 for minor and RMSD <0.05 for major) from the 100 lowest conformers employing an in-house Python script.

DFT refinement of the xTB conformers: to obtain a more refined picture of the constrained transition state structures and their respective energies while keeping time demands reasonable, we employed calculations with density fitting at the DF-PBE-D3/def2-SVP (C,H)/ def2-TZVPPD (N,O,F) level of theory, including the corresponding Resolution of Identity basis sets for density fitting and implicit solvation (CPCM(CH_2Cl_2)).

Optimization (M062X) and single point energy calculations (ωB97x-D3): We optimised all structures obtained after DFT-refinement that were in a 0-14 kJ mol^{-1} (corresponding to > 0.4 % weighting) window with our method of choice: M062X / def2-SVP (C,H) / def2-TZVP (F) / def2-TZVPD (N,O,Cl) / CPCM(CH_2Cl_2) /ultrafine grid. Further duplicates were removed, allowing for eight unique conformers, comprising four major and four minor transition state structures, for which single-point energies were evaluated (**Figure S24**).

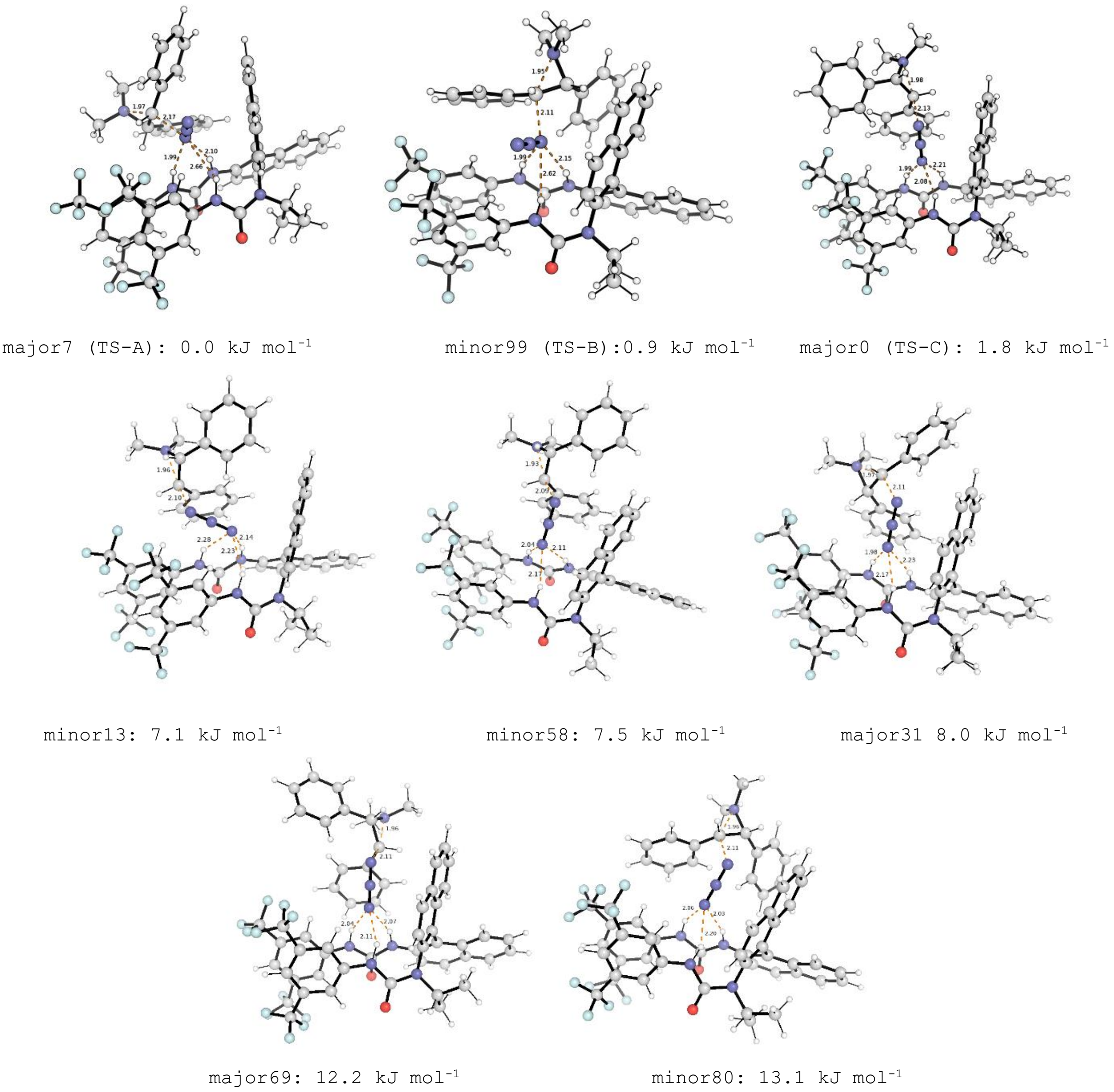

Figure S24 Eight most stable transition state structures with relative Gibbs Free Energies.

The energy decomposition analysis described in the manuscript was performed based on fragmentation of the three energetically lowest TS structures, TS-A, TS-B and TS-C as shown in **Table S28**. The charge distribution on the electrophilic carbon atom of the substrate is nearly identical for all three cases, implying a similar 'lateness' of the three TSs on the PES. We could further exclude, as discussed in the main manuscript, that the main factor for TS stability is the absence of distortion. In addition to the favorable non-covalent interactions described in the manuscript specifically for TS-A and TS-B, the polarization induced by the H bonding interaction onto the electronic distribution of the azide nucleophile (FRAG A2 and FRAG B2) is identical to the polarization observed in the transition state structures. For TS-C, however, the corresponding electronic preorganization is less favorable.

Table S28 Key properties of the three most stable transition state structures and their fragments.

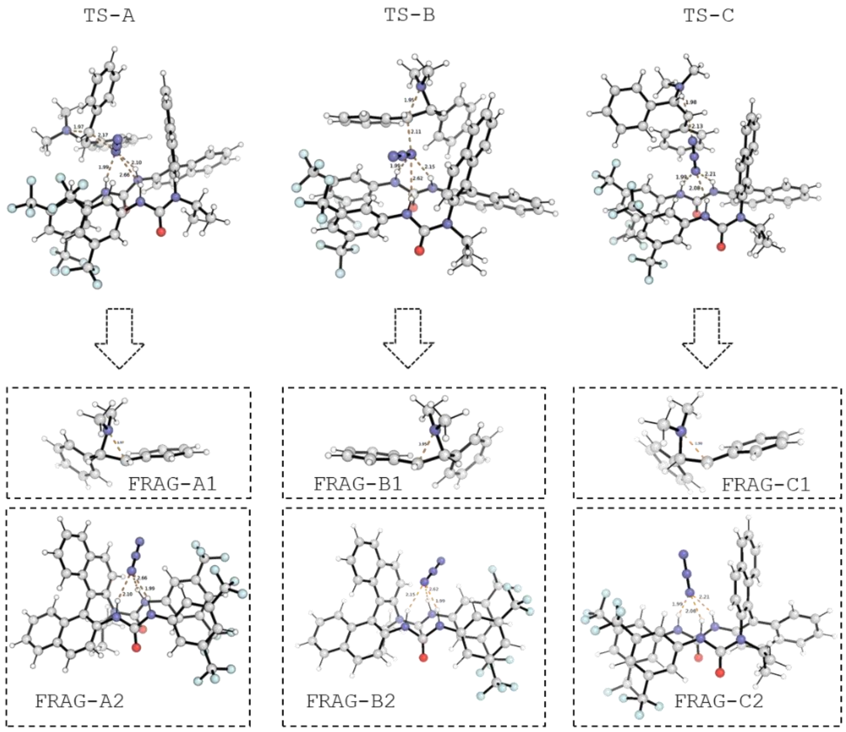

property	unit	TS-A (major7)	TS-B (minor99)	TS-C (major0)
attacking N	complex	NA	NA	NC
charge	substrate	FRAG-A1<br>CA = +0.163	FRAG-B1<br>CB = +0.156	FRAG-C1<br>CA = +0.162
distortion	[(*S*)-**1k**·N_3]$^-$	FRAG-A2<br>+ 2.8 kJ mol^{-1}	FRAG-B2<br>+ 8.3 kJ mol^{-1}	FRAG-C2<br>+ 0.0 kJ mol^{-1}
distortion	substrate	FRAG-A1<br>+ 3.1 kJ mol^{-1}	FRAG-B2<br>+ 2.4 kJ mol^{-1}	FRAG-C1<br>+ 0.0 kJ mol^{-1}

Absolute energies of computed structures

Method Validation

including CPCM(CH_2Cl_2)

	Energies (Hartree)	
	DLPNO-CCSD(T)	ωB97x-D3
dimer	-613.9879364	-615.0132289
urea monomer	-224.9507887	-225.3283298
urea-azide	-389.0258807	-389.6756995

in vacuo

	Energies (Hartree)	
	DLPNO-CCSD(T)	ωB97x-D3
dimer	-613.9089687	-614.9340603
urea monomer	-224.9327324	-225.3088552
urea-azide	-388.9417976	-389.5926221

[(*S*)-1k·N_3]$^-$ conformers

	Energies (Hartree)		
	$E_{CPCM(CH2Cl2)}{}^{a}$	$E_{CPCM(CH2Cl2)}{}^{b}$	$E_{CPCM(CH2Cl2)}{}^{c}$
conf1	-3311.827757	-3311.781112	-3311.780968
conf2	-3311.777805	-3311.778968	-3311.77883
conf3	-3311.779388	-3311.780536	-3311.780546
conf4	-3311.781006	-3311.782624	-3311.782607
conf5	-3311.775205	-3311.775994	-3311.775775
conf6	-3311.783645	-3311.784165	-3311.784159
conf7	-3311.779508	-3311.780216	-3311.78027
conf8	-3311.779864	-3311.780634	-3311.780646
conf9	-3311.786045	-3311.786828	-3311.786848
conf10	-3311.780738	-3311.781451	-3311.781486
conf11	-3311.779743	-3311.780214	-3311.780268
conf12	-3311.779878	-3311.780643	-3311.780651

*[(S)-**1k**·N_3]$^-$ conformers with corresponding electronic energies. Single point energies obtained via a: ωB97x-D3 / (ma)-def2-TZVPP (N,O) / def2-TZVPP (C,H) / CPCM(CH_2Cl_2), default settings for CPCM, b and c: ωB97x-D3 / (ma)-def2-TZVPP (N,O) / def2-TZVPP (C,H) / CPCM(CH_2Cl_2), vdW surface for CPCM. For a and b, the electronic were obtained based on structures optimized via the M06-2X/BS1/(CPCM(CH_2Cl_2) level of theory, for c the optimization was performed at the M06-2X/BS7/(CPCM(CH_2Cl_2) level of theory, see above.*

10 unique [(*S*)-1k·N_3]$^-$ conformers

	Energies (Hartree)			
	G-qh (opt)	E (opt)	E(sp)	corrected G
conf1	-3309.17597	-3309.749263	-3311.780968	-3311.207675
conf2	-3309.174887	-3309.74794	-3311.77883	-3311.205777
conf3	-3309.176001	-3309.748955	-3311.780546	-3311.207592
conf4	-3309.17821	-3309.750406	-3311.782607	-3311.210411
conf5	-3309.17209	-3309.744746	-3311.775775	-3311.203119
conf6	-3309.178768	-3309.749837	-3311.784159	-3311.21309
conf7	-3309.175516	-3309.748362	-3311.78027	-3311.207424
conf8	-3309.176092	-3309.747921	-3311.780646	-3311.208817
conf9	-3309.181237	-3309.754385	-3311.786848	-3311.2137
conf10	-3309.176693	-3309.748349	-3311.781486	-3311.20983

Transition structures with [(*S*)-1k·N_3]$^-$

PBE-D3 refinement of xTB structures (minor):

	Energies (Hartree)		
conf	E	∆E	comment
0	-3981.287378	16.8	too high in energy
2	*directly submitted for final optimization and energy calculation*		
6	-3981.290117	9.6	duplicate to 13
12	-3981.284425	24.6	too high in energy
13	-3981.290118	9.6	end-on
14	-3981.289573	11.1	end-on
15	-3981.286814	18.3	duplicate to 29
16	-3981.290986	7.4	duplicate to 19
18	-3981.290572	8.5	end-on
19	-3981.290679	8.2	duplicate to 16
24	-3981.28634	19.6	too high in energy
25	-3981.282099	30.7	too high in energy
29	-3981.286743	18.5	duplicate to 15
30	-3981.287937	15.4	too high in energy
36	-3981.290236	9.3	end-on
38	-3981.284131	25.4	too high in energy
40	-3981.29041	8.9	duplicate to 19
41	-3981.289661	10.8	duplicate to 14
43	-3981.284119	25.3	too high in energy
47	-3981.286566	19.0	too high in energy
50	-3981.285629	21.4	too high in energy
51	-3981.285666	21.3	too high in energy
52	-3981.289691	10.8	duplicate to 14
56	-3981.286656	18.7	too high in energy
57	-3981.287434	16.7	too high in energy
58	-3981.290092	9.7	end-on
59	-3981.293791	0	side-on

64	-3981.287989	15.2	too high in energy
66	-3981.287661	16.1	too high in energy
67	-3981.28786	15.6	too high in energy
68	-3981.287759	15.8	too high in energy
69	-3981.280974	33.7	too high in energy
71	-3981.289334	11.7	duplicate to 14
72	-3981.287229	17.2	too high in energy
73	-3981.292854	2.5	side-on
75	-3981.285291	22.3	too high in energy
77	-3981.287004	17.8	too high in energy
78	-3981.286997	17.8	too high in energy
80	-3981.28974	10.6	end-on
81	-3981.282394	29.9	too high in energy
86	-3981.292268	4.0	duplicate to 99
88	-3981.288116	14.9	too high in energy
89	-3981.289414	11.5	duplicate to 80
93	-3981.285857	20.8	too high in energy
94	-3981.287154	17.4	too high in energy
95	-3981.288927	12.8	end-on/side-on
97	-3981.285868	20.8	too high in energy
99	-3981.293403	1.0	side-on

PBE-D3 refinement of xTB structures (major):

	Energies (Hartree)		
conf	E	ΔE	comment
0	-3981.296941	0	end-on
3	-3981.287838	23.9	too high in energy
4	-3981.283588	35.1	too high in energy
7	-3981.292826	10.8	side-on
8	-3981.287456	24.9	too high in energy
10	-3981.281913	39.5	too high in energy
11	-3981.286471	27.5	too high in energy
13	-3981.295297	4.3	side-on
15	-3981.295292	4.3	duplicate to 13
16	-3981.295337	4.2	duplicate to 13
17	-3981.287979	23.5	too high in energy
18	-3981.291559	14.1	too high in energy
19	-3981.295525	3.7	duplicate to 13
20	-3981.295446	3.9	duplicate to 13
22	-3981.295393	4.1	duplicate to 13
23	-3981.295397	4.1	duplicate to 13
24	-3981.285758	29.4	too high in energy
27	-3981.29549	3.8	duplicate to 13
28	-3981.294967	5.2	duplicate to 13
29	-3981.295355	4.2	duplicate to 13
30	-3981.295347	4.2	duplicate to 13
31	-3981.292085	12.7	side-on
32	-3981.2829	36.9	too high in energy

34	-3981.295089	4.9	duplicate to 13
36	-3981.29181	13.5	duplicate to31
37	-3981.294881	5.4	duplicate to 13
40	-3981.288149	23.1	too high in energy
44	-3981.295079	4.9	duplicate to 13
46	-3981.284434	32.8	too high in energy
49	-3981.29501	5.1	duplicate to 13
52	-3981.285583	29.8	until here: 0.05 RMSD
54	-3981.292716	11.1	from here: 0.07 RMSD duplicate to 31
56	-3981.287008	26.1	too high in energy
58	-3981.283369	35.6	too high in energy
63	-3981.294923	5.3	duplicate to 13
65	-3981.294985	5.1	duplicate to 13
66	-3981.295094	4.9	duplicate to 13
69	-3981.293752	8.4	side-on
73	-3981.28856	22.0	too high in energy
74	-3981.295003	5.1	duplicate to 13
75	-3981.293872	8.1	duplicate to 69
76	-3981.287457	24.9	too high in energy
77	-3981.284547	32.5	too high in energy
80	-3981.294792	5.6	very similar to 13
82	-3981.29532	4.3	duplicate to 13
83	-3981.291106	15.3	too high in energy
84	-3981.293678	8.6	duplicate to 69
85	-3981.284962	31.5	too high in energy
86	-3981.285058	31.2	too high in energy
88	-3981.292457	11.8	duplicate to31
90	-3981.295301	4.3	duplicate to 13
92	-3981.286728	26.8	too high in energy
95	-3981.294473	6.5	duplicate to 13
97	-3981.291743	13.6	duplicate to 31
99	-3981.292646	11.3	duplicate to 31

Most stable major and minor transition state structures

		Energies (Hartree)		
minor conf	G-qh (opt)	E (opt)	E(sp)	corrected G
13	-3983.255672	-3984.115225	-3986.937713	-3986.07816
58	-3983.255471	-3984.116809	-3986.939361	-3986.078023
80	-3983.252772	-3984.115743	-3986.938867	-3986.075896
99	-3983.257673	-3984.119649	-3986.942496	-3986.08052

		Energies (Hartree)		
major conf	G-qh (opt)	E (opt)	E(sp)	corrected G
0	-3983.2576	-3984.119196	-3986.941804	-3986.080208
7	-3983.254521	-3984.11626	-3986.942618	-3986.080879
31	-3983.255499	-3984.118483	-3986.940804	-3986.07782
69	-3983.25497	-3984.117873	-3986.939123	-3986.07622

[(*S*)-1k·N_3]$^-$ binding energies

Note: The conformation of (*S*)-**1k** is based on conf9, the most stable [(*S*)-**1k**·N_3]$^-$ conformer.

	Energies (Hartree)			
major conf	G-qh (opt)	E (opt)	E(sp)	corrected G
[(*S*)-**1k**·N_3]$^-$	-3309.181237	-3309.754385	-3311.786848	-3311.2137
N_3^-	-164.329707	-164.322898	-164.3370191	-164.343828
(*S*)-**1k**	-3144.82934	-3145.392775	-3147.423213	-3146.85978
[(*S*)-**1k**·N_3]$^-$	-3605.229752	-3605.792194	-3607.830149	-3607.26771
Cl^-	-460.378886	-460.366882	-460.3840778	-460.396082

Mechanistic evaluation with achiral urea (catalyst concentration 0.1M)

Aziridinium formation (catalyzed)

		Energies (Hartree)			
		G-qh (opt)	E (opt)	E(sp)	corrected G
SM	free Schreiner (0.1 M)	-2034.823919	-2035.014754	-2035.914153	-2035.723318
	aminochloride	-1134.490349	-1134.756399	-1135.556644	-1135.290594
TS	TS cat	-3169.280659	-3169.761666	-3171.460927	-3170.97992
product	close contact IP	-3169.320004	-3169.802989	-3171.502464	-3171.019479

Aziridinium formation (uncatalyzed)

		Energies (Hartree)			
		G-qh (opt)	E (opt)	E(sp)	corrected G
SM	aminochloride	-1134.490349	-1134.756399	-1135.556644	-1135.290594
TS	TS uncat	-1134.45262	-1134.716458	-1135.520399	-1135.256561
product	close contact IP	-1134.486309	-1134.751686	-1135.557189	-1135.291812

Azidation

		Energies (Hartree)			
		G-qh (opt)	E (opt)	E(sp)	corrected G
	IPconfA	-2873.268562	-2873.762365	-2875.455	-2874.961197
SM	IPconfB	-2873.264252	-2873.758083	-2875.451735	-2874.957904
	IPconfC	-2873.257065	-2873.747067	-2875.442837	-2874.952835
	TS-A’ (end-on)	-2873.2414	-2873.731216	-2875.423041	-2874.933225
TS	TS-B’ (end-on)	-2873.23972	-2873.731172	-2875.423922	-2874.93247
	TS-C’ (side-on)	-2873.235886	-2873.727759	-2875.41883	-2874.926957
product	amino azide	-838.469512	-838.746931	-839.5411273	-839.2637083
	free Schreiner(0.1 M)	-2034.823919	-2035.014754	-2035.914153	-2035.723318

Schreiner’s Urea:azide binding energetics

	Energies (Hartree)			
	G-qh (opt)	E (opt)	E(sp)	corrected G
SUAconfA	-2199.163953	-2199.364317	-2200.27012	-2200.069756
SUAconfB	-2199,163411	-2199,363551	-2200,268423	-2200,068283
SUAconfC	-2199,156665	-2199,357473	-2200,262476	-2200,061668
azide	-164.329707	-164.322898	-164.3370191	-164.343828
free Schreiner (1M)	-2034.821745	-2035.014754	-2035.914153	-2035.721144

Schreiner chloride	-2495.216884	-2495.406959	-2496.317815	-2496.12774
chloride	-460.378886	-460.366882	-460.3840778	-460.396082
free Schreiner (1M)	-2034.821745	-2035.014754	-2035.914153	-2035.721144

NMR spectra

***N*[1],*N*[2]-bis(3,5-bis(trifluoromethyl)phenyl)oxalamide**

^{1}H NMR (400 MHz, Acetone-d_6)

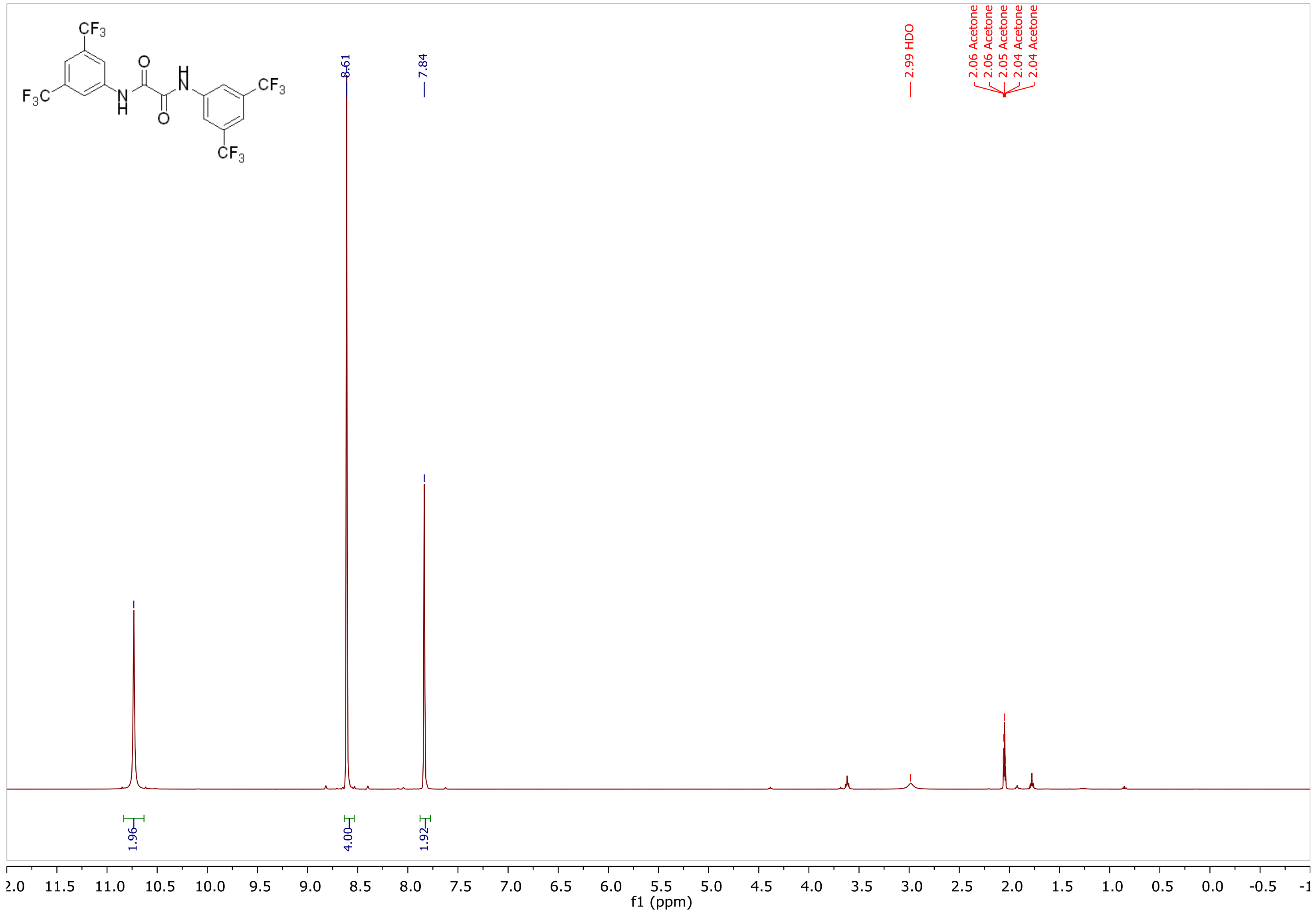

^{19}F NMR (377 MHz, Acetone-d_6)

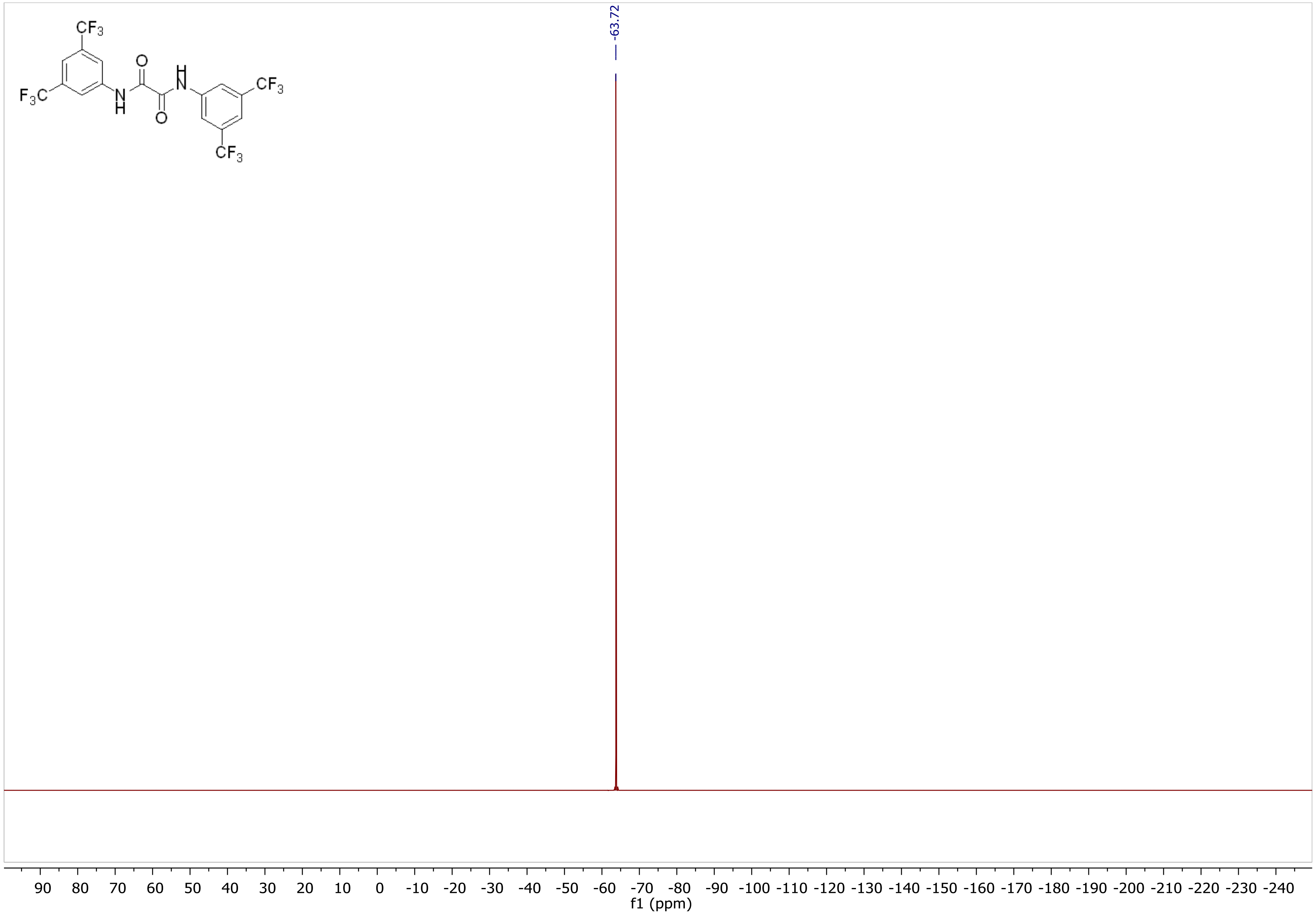

^{13}C NMR (101 MHz, Acetone-d_6)

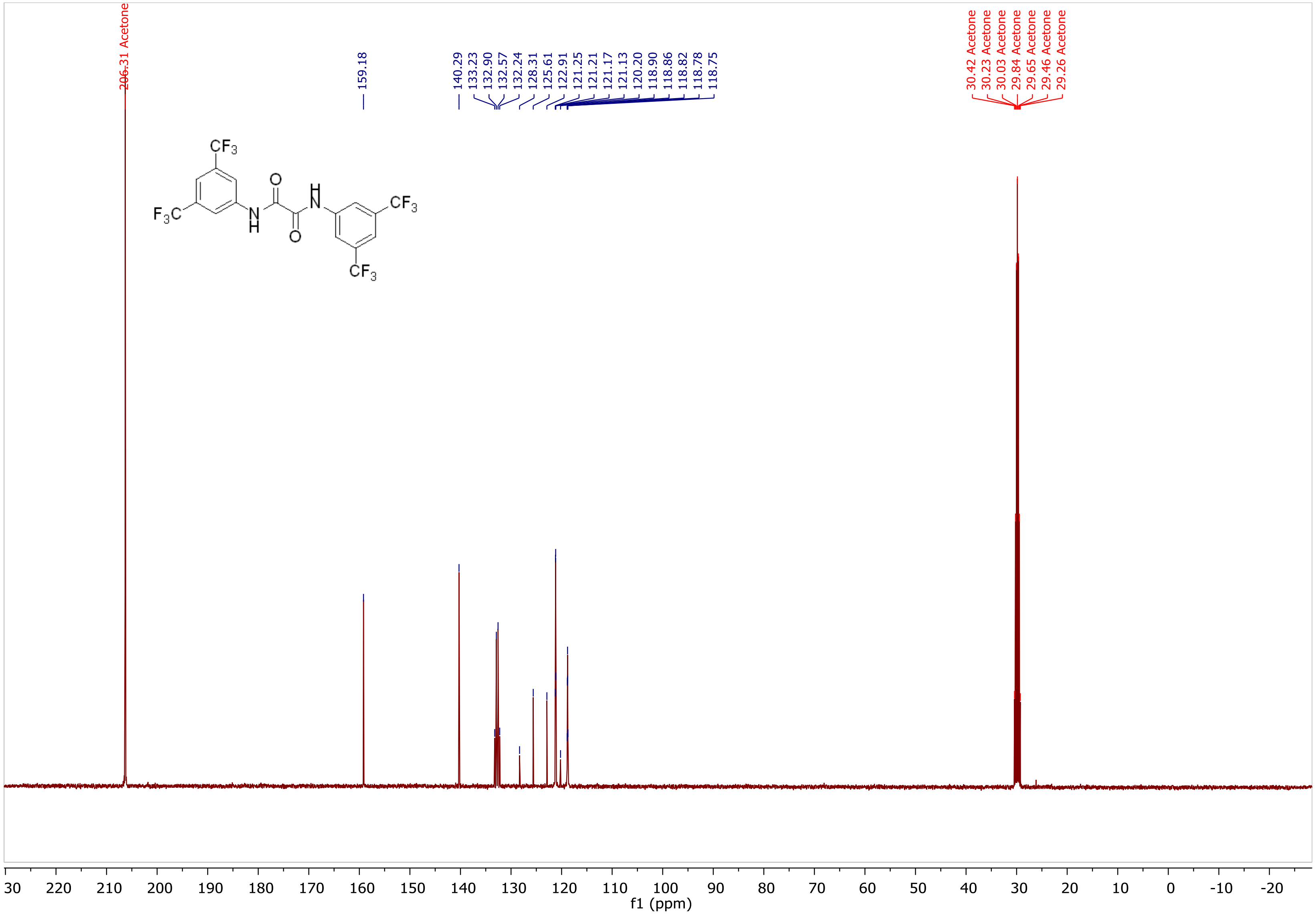

Tetrabutylammonium [1-^{15}N]-azide

^{1}H NMR (500 MHz, $CDCl_3$)

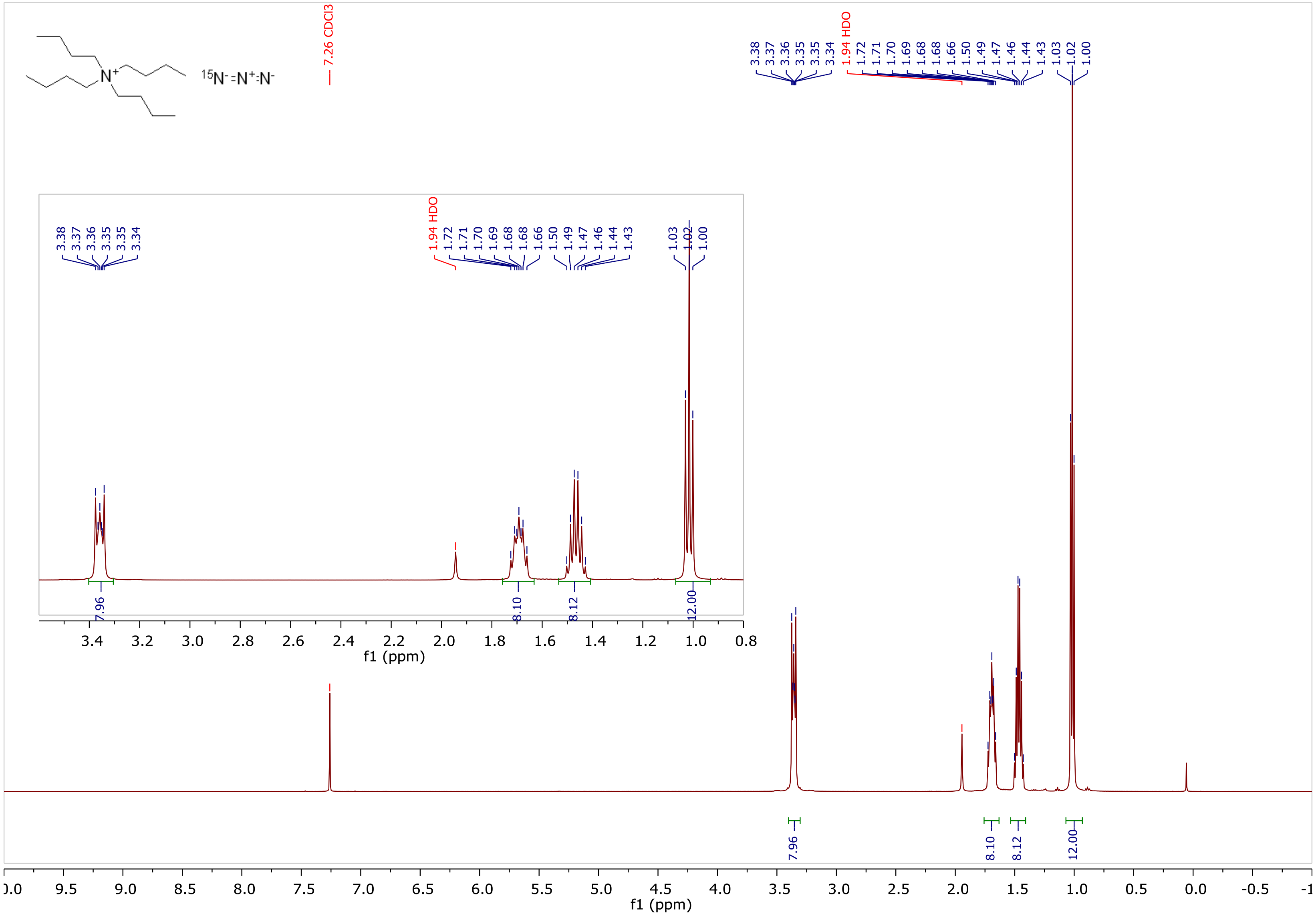

^{13}C NMR (126 MHz, $CDCl_3$)

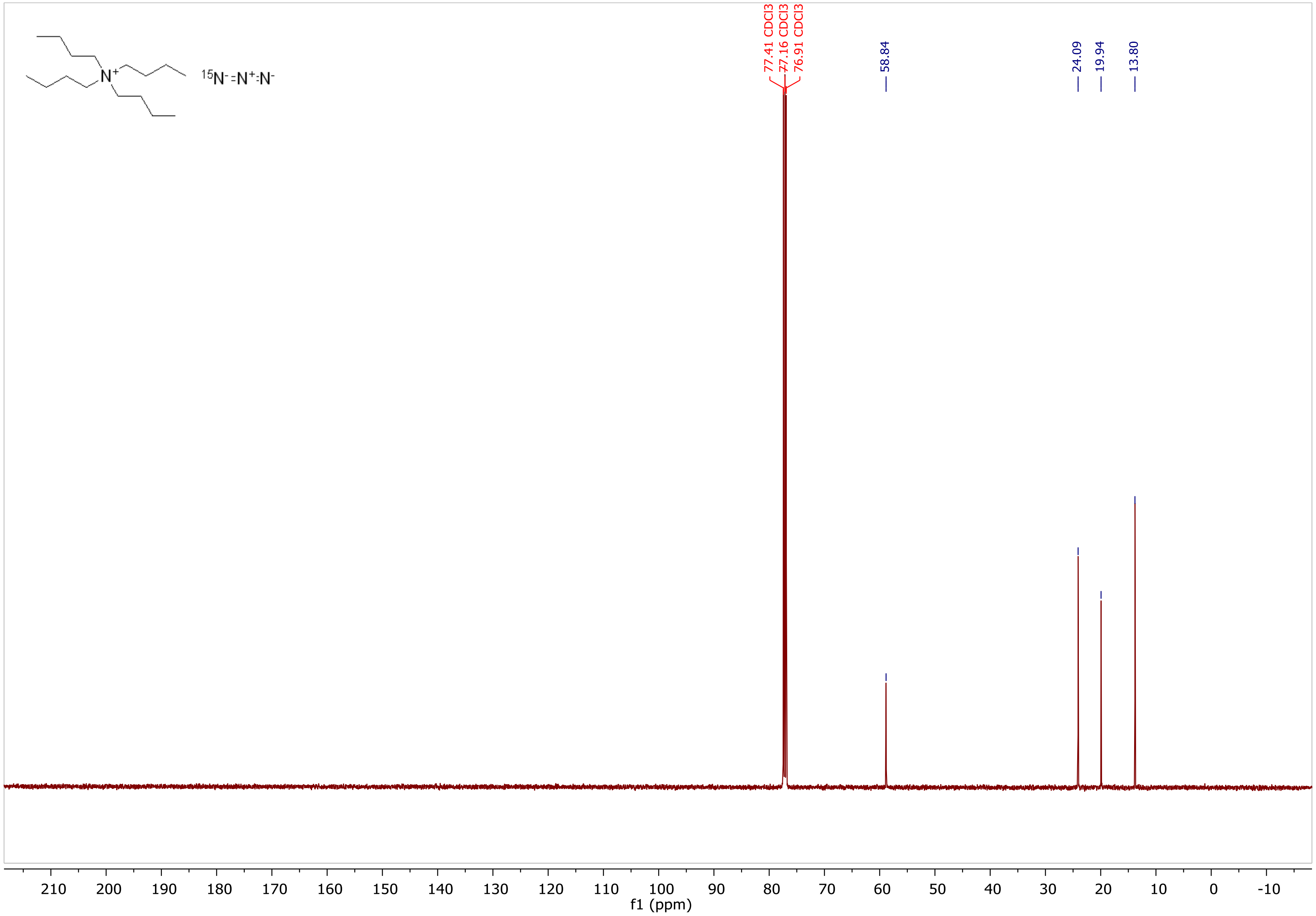

^{15}N NMR (51 MHz, $CDCl_3$)

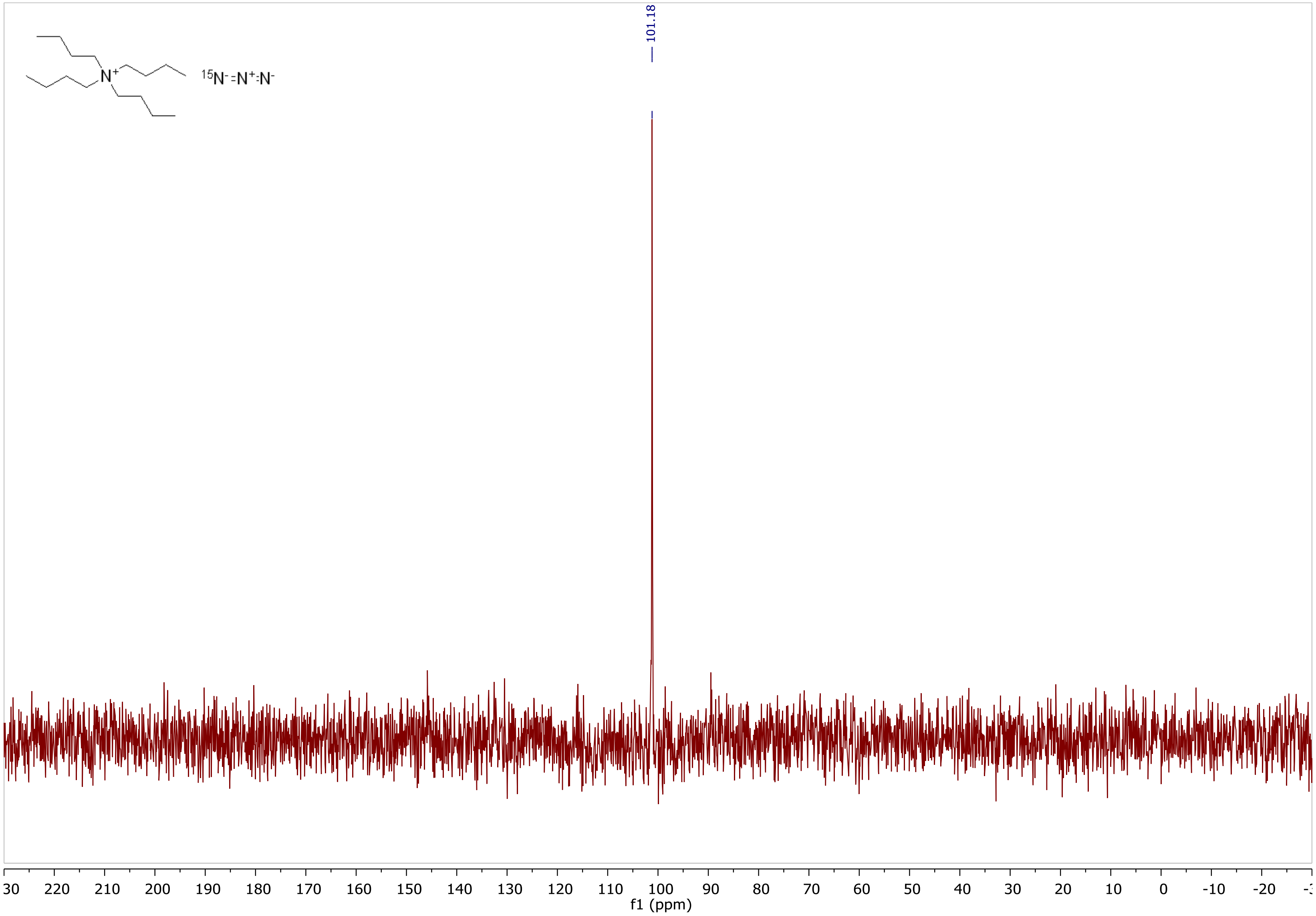

1,3-bis(3,5-bis(trifluoromethyl)phenyl)urea·tetrabutylammonium azide (1a·N_3·Bu_4N)

^{1}H NMR (400 MHz, Acetone-d_6)

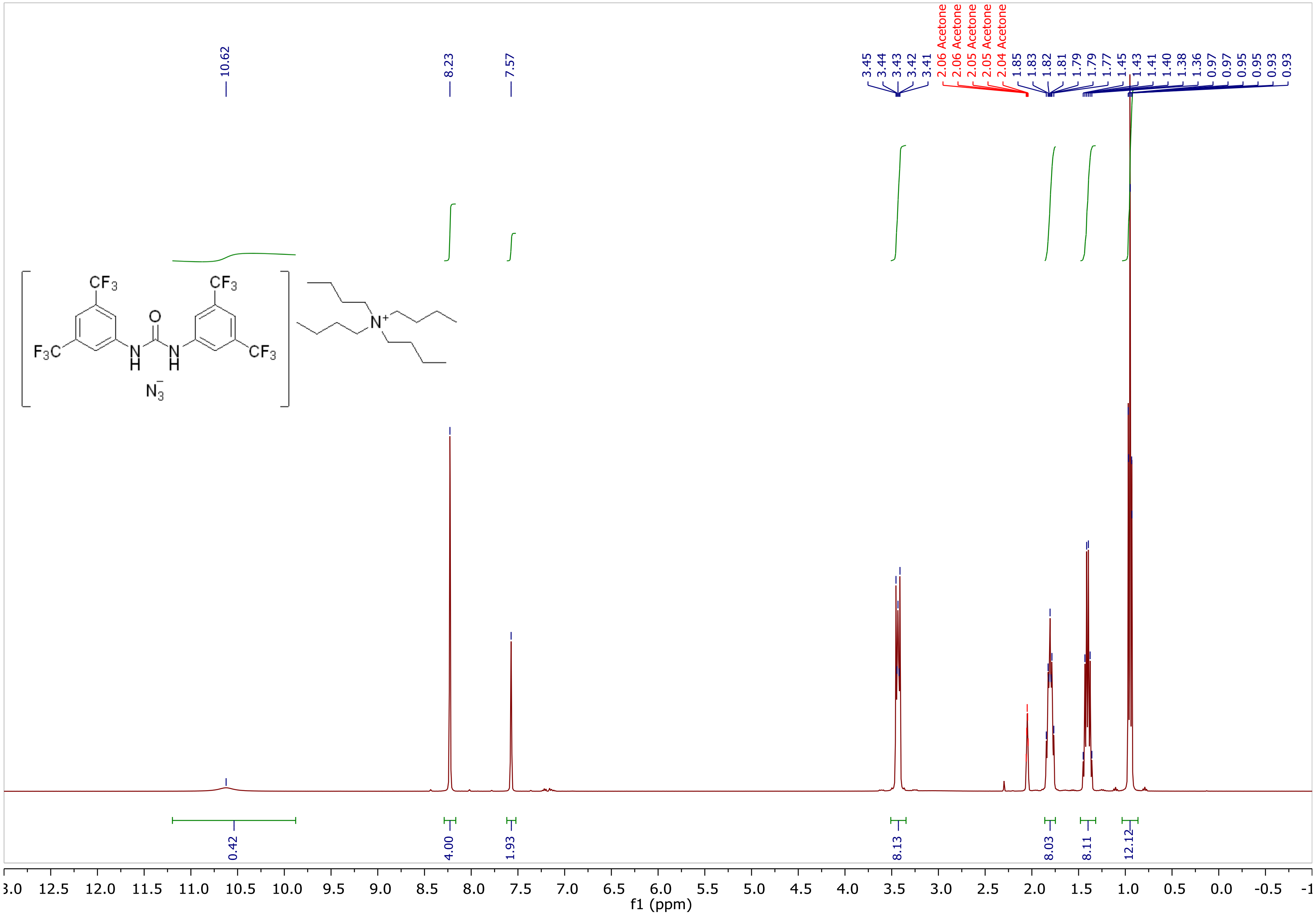

^{19}F NMR (377 MHz, Acetone-d_6)

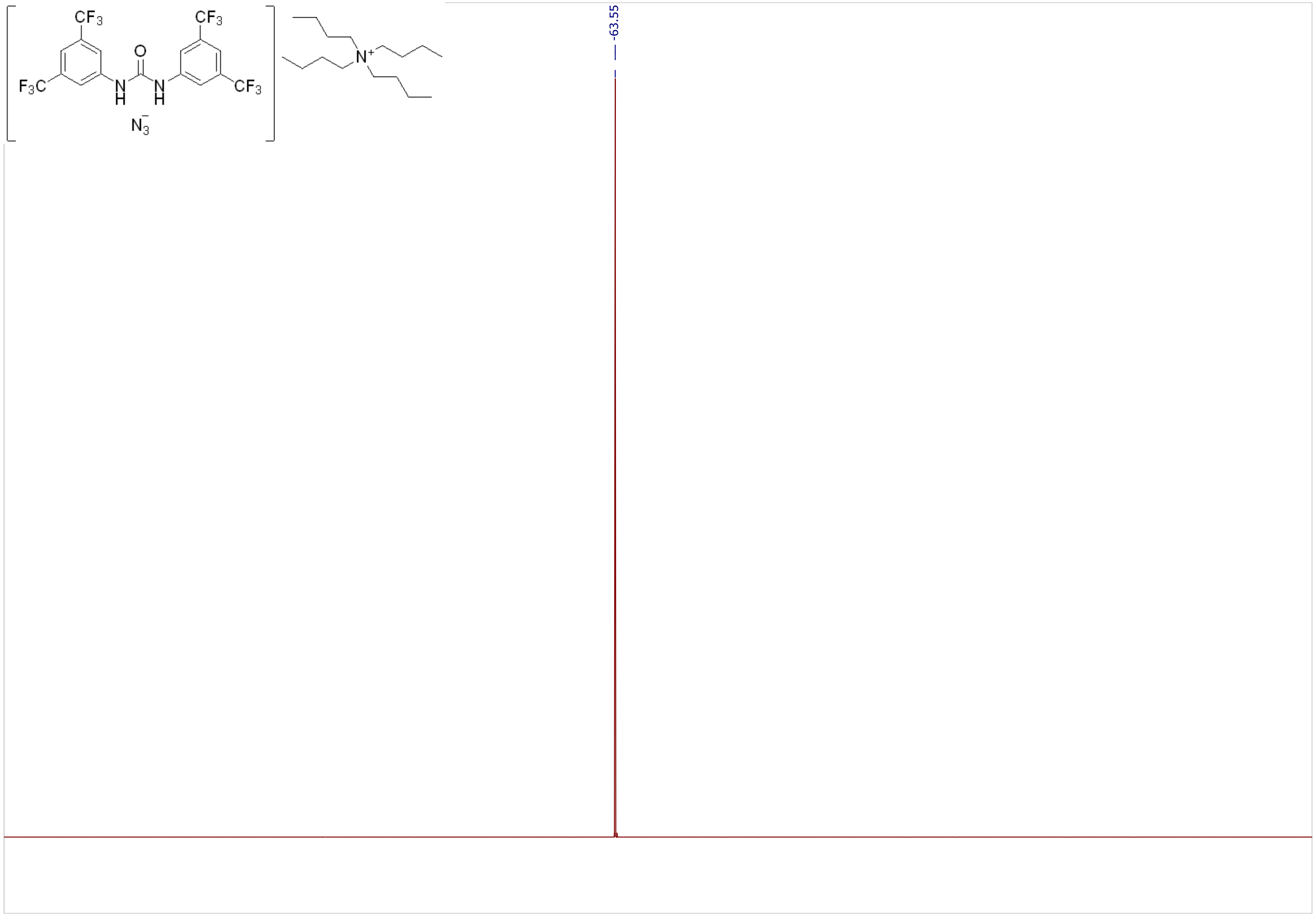

^{13}C NMR (101 MHz, Acetone-d_6)

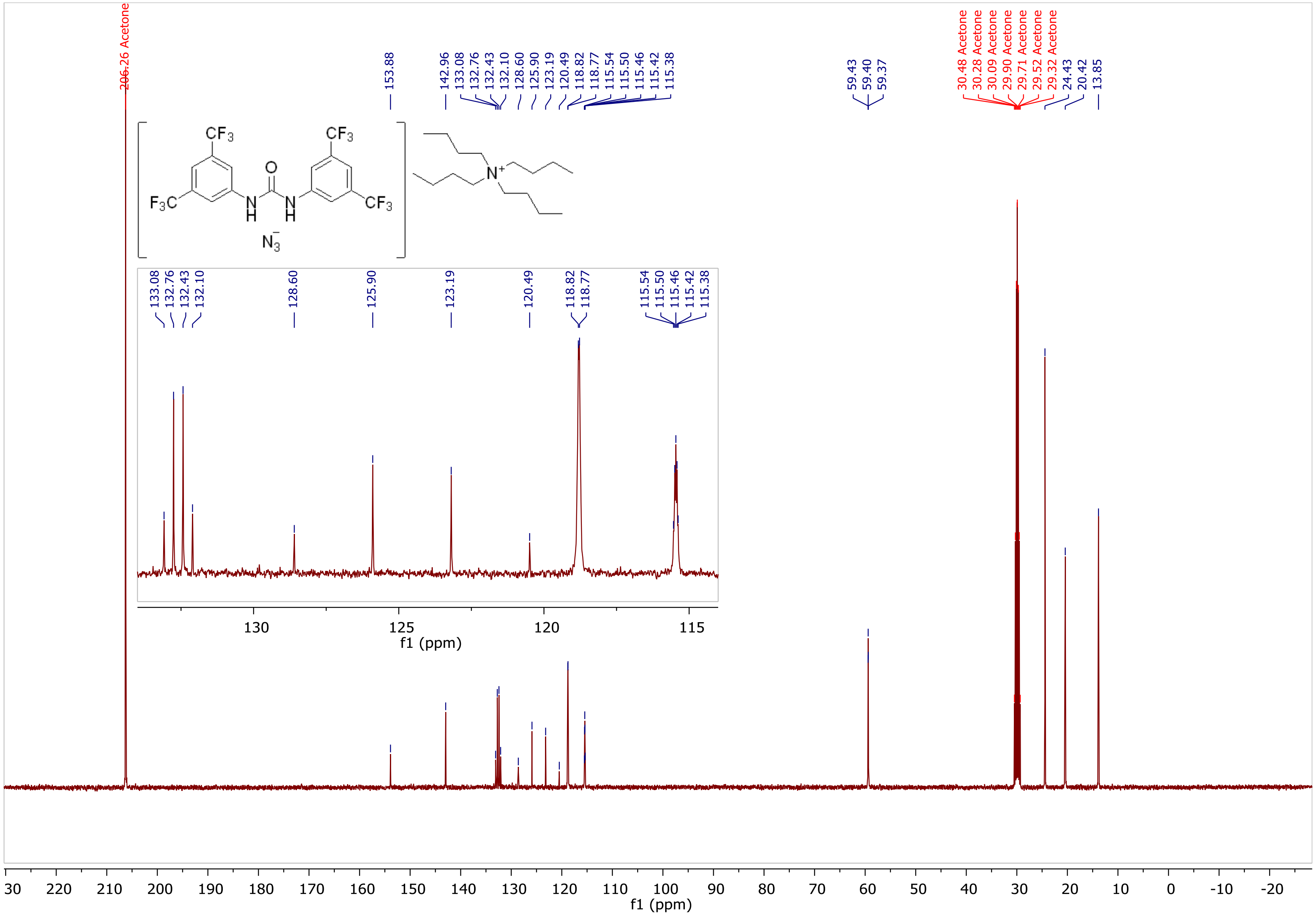

1,3-bis(3,5-bis(trifluoromethyl)phenyl)urea·sodium azide·15-crown-5 (1a·N_3·Na·15-crown-5)

^{1}H NMR (500 MHz, Acetone-d_6)

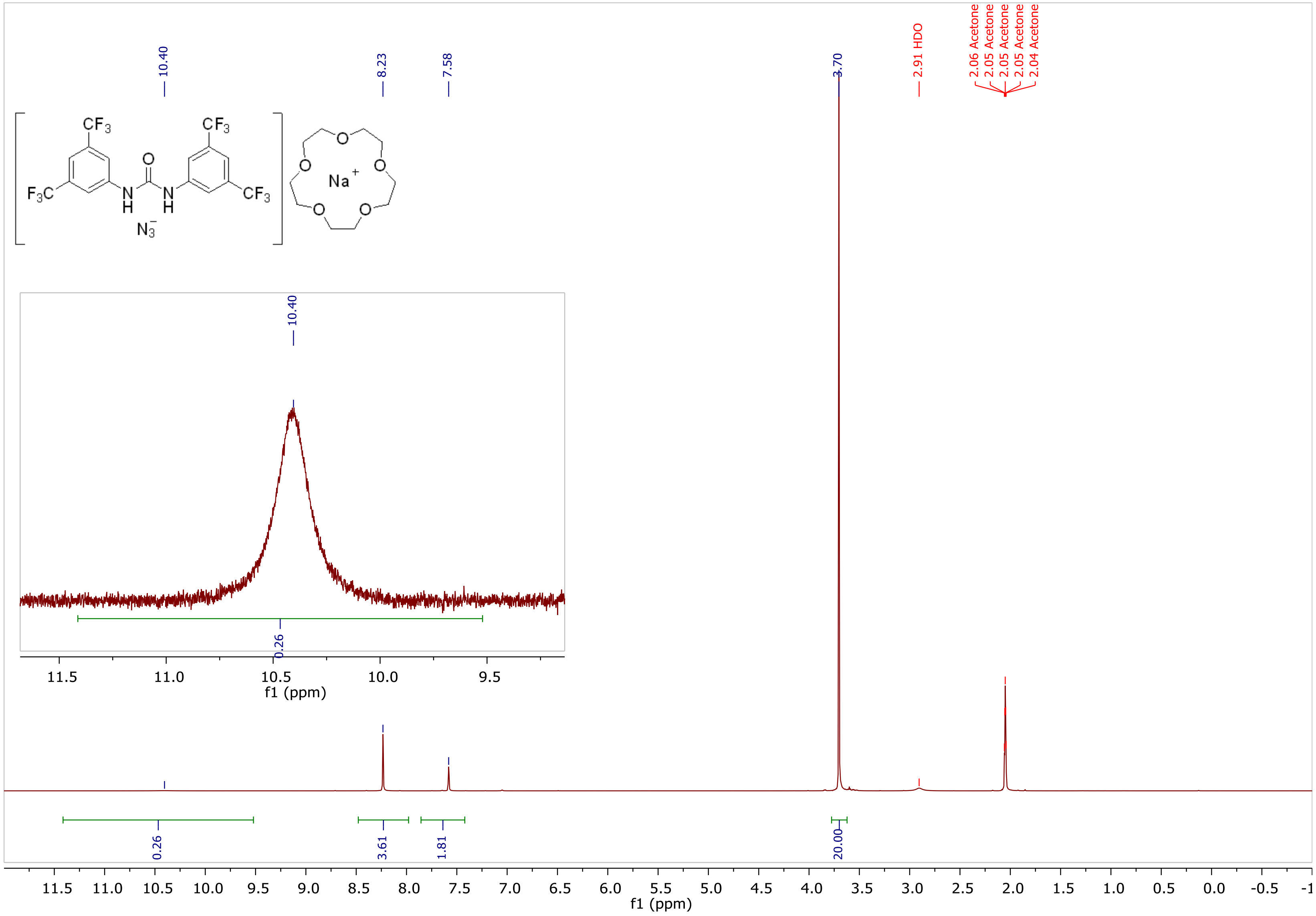

^{19}F NMR (470 MHz, Acetone-d_6)

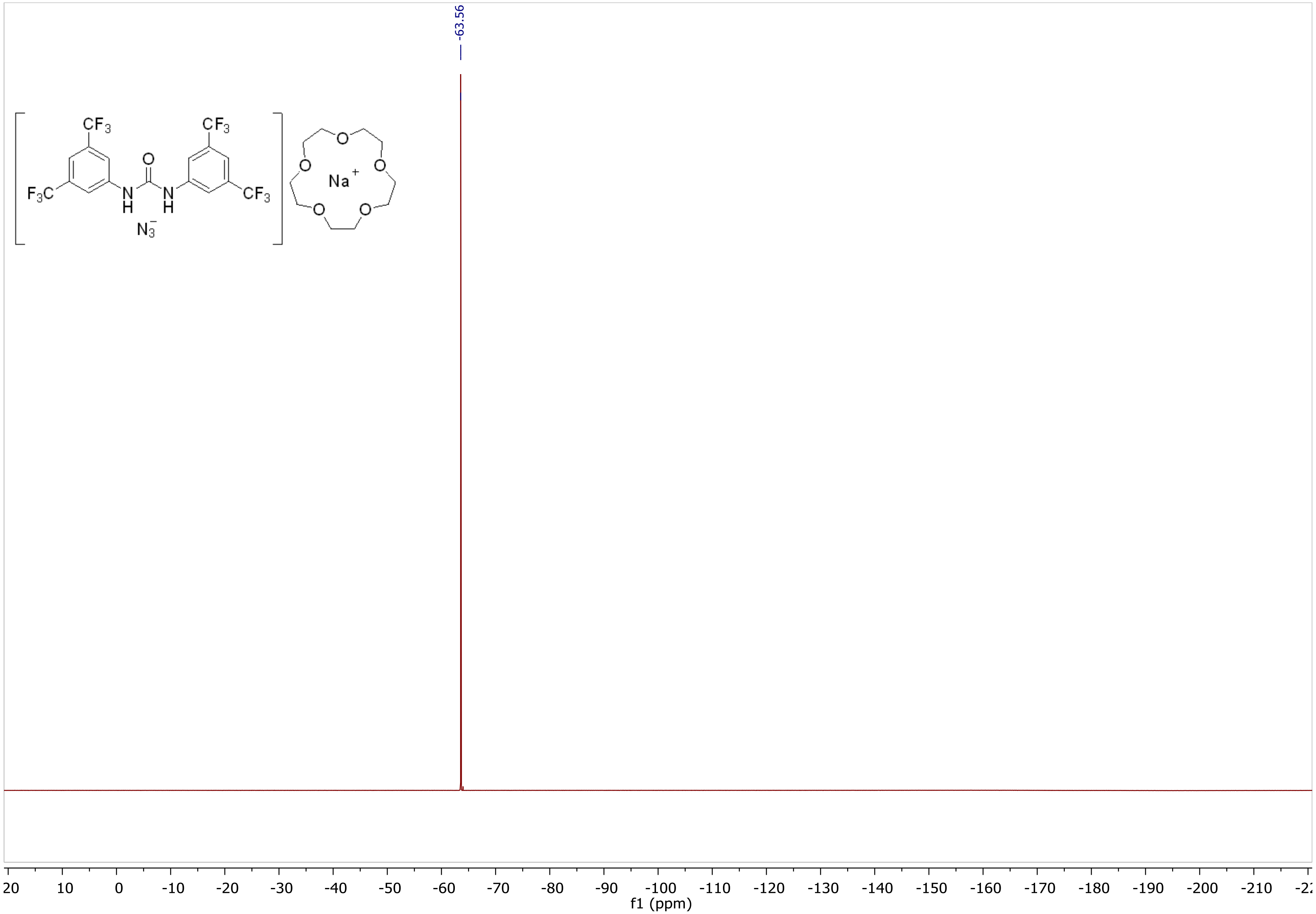

^{13}C NMR (126 MHz, Acetone-d_6)

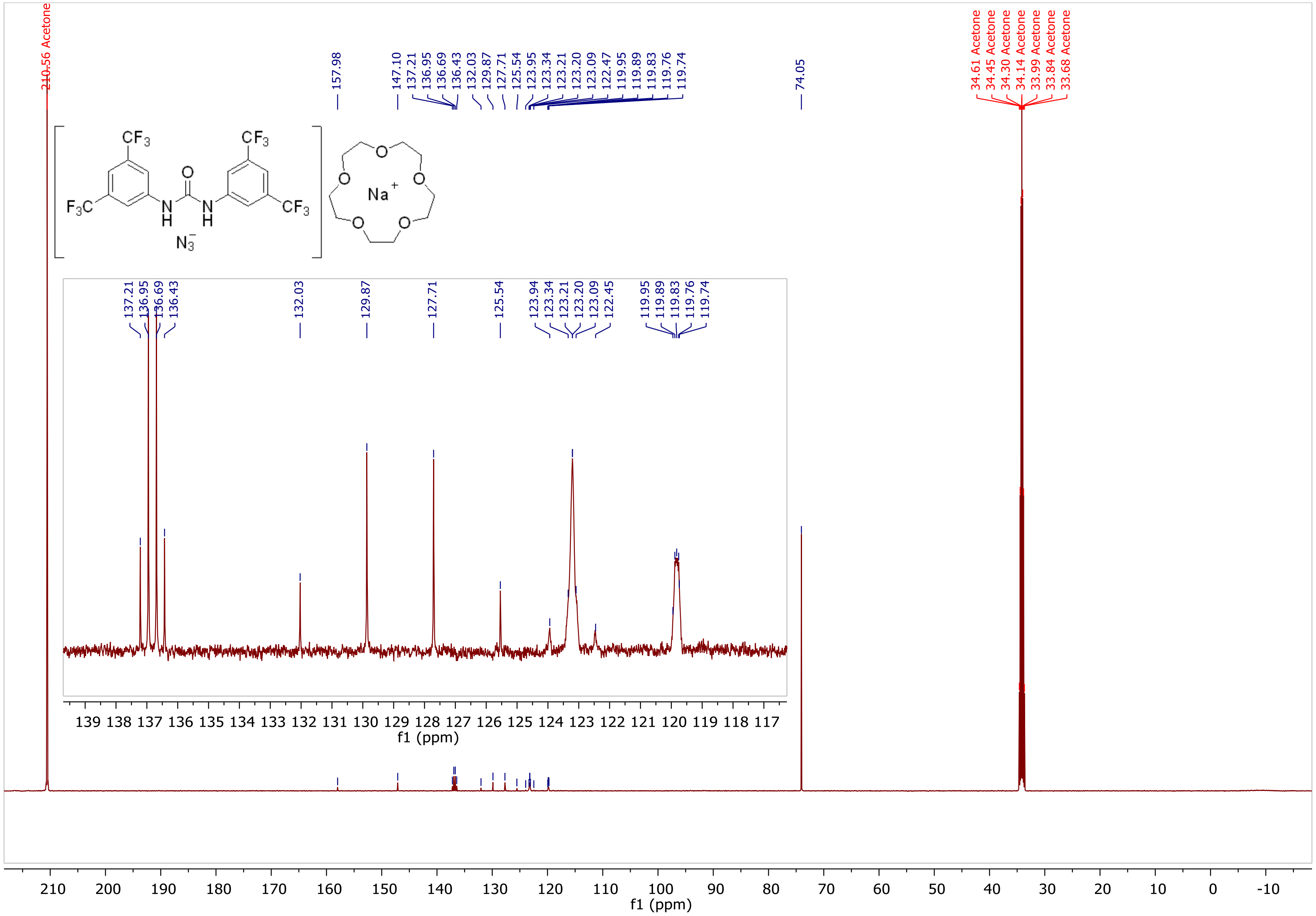

1,3-bis(3-chlorophenyl)urea·tetrabutylammonium azide (1b·N_3·Bu_4N)

^{1}H NMR (400 MHz, CD_3CN)

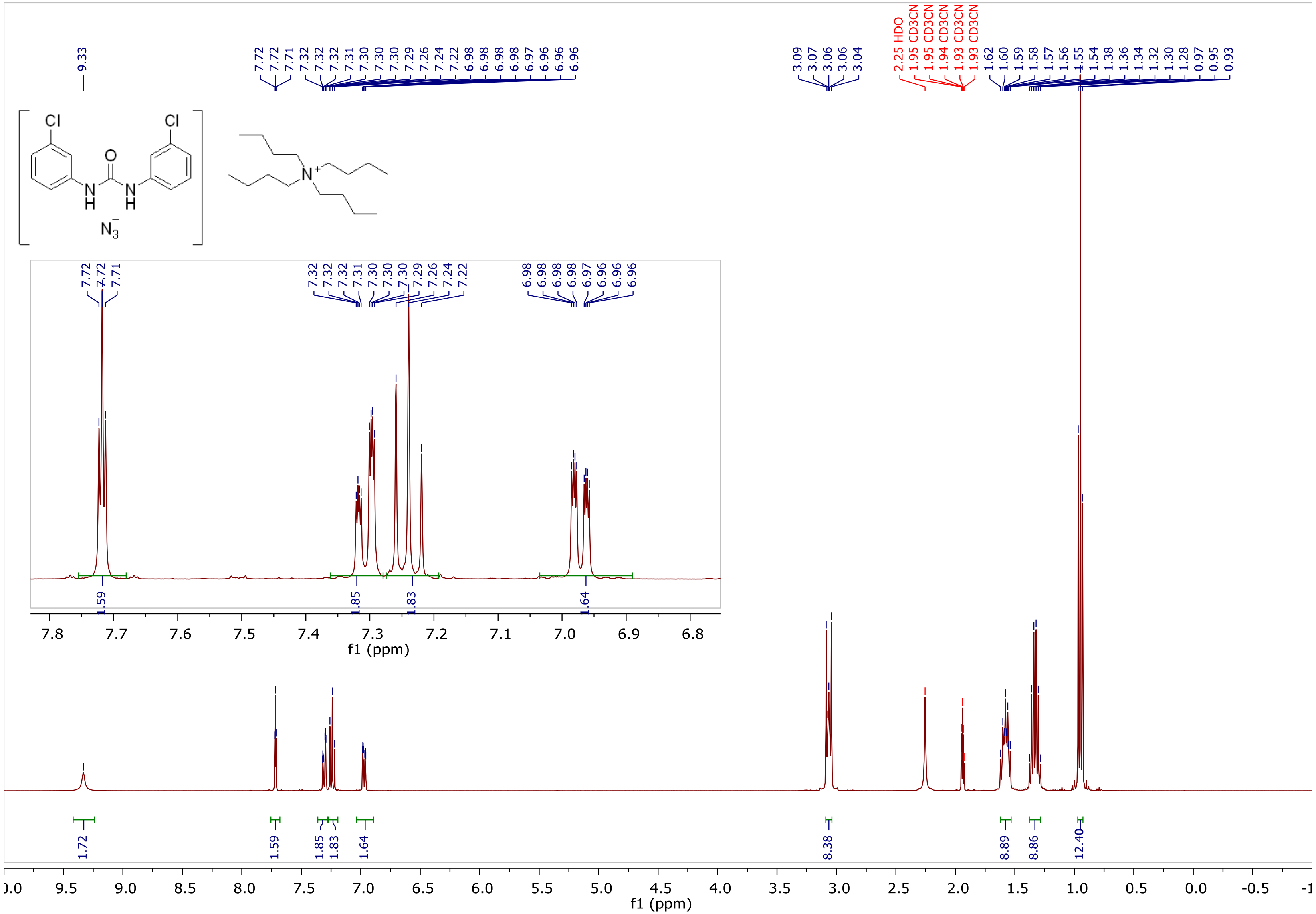

^{13}C NMR (101 MHz, CD_3CN)

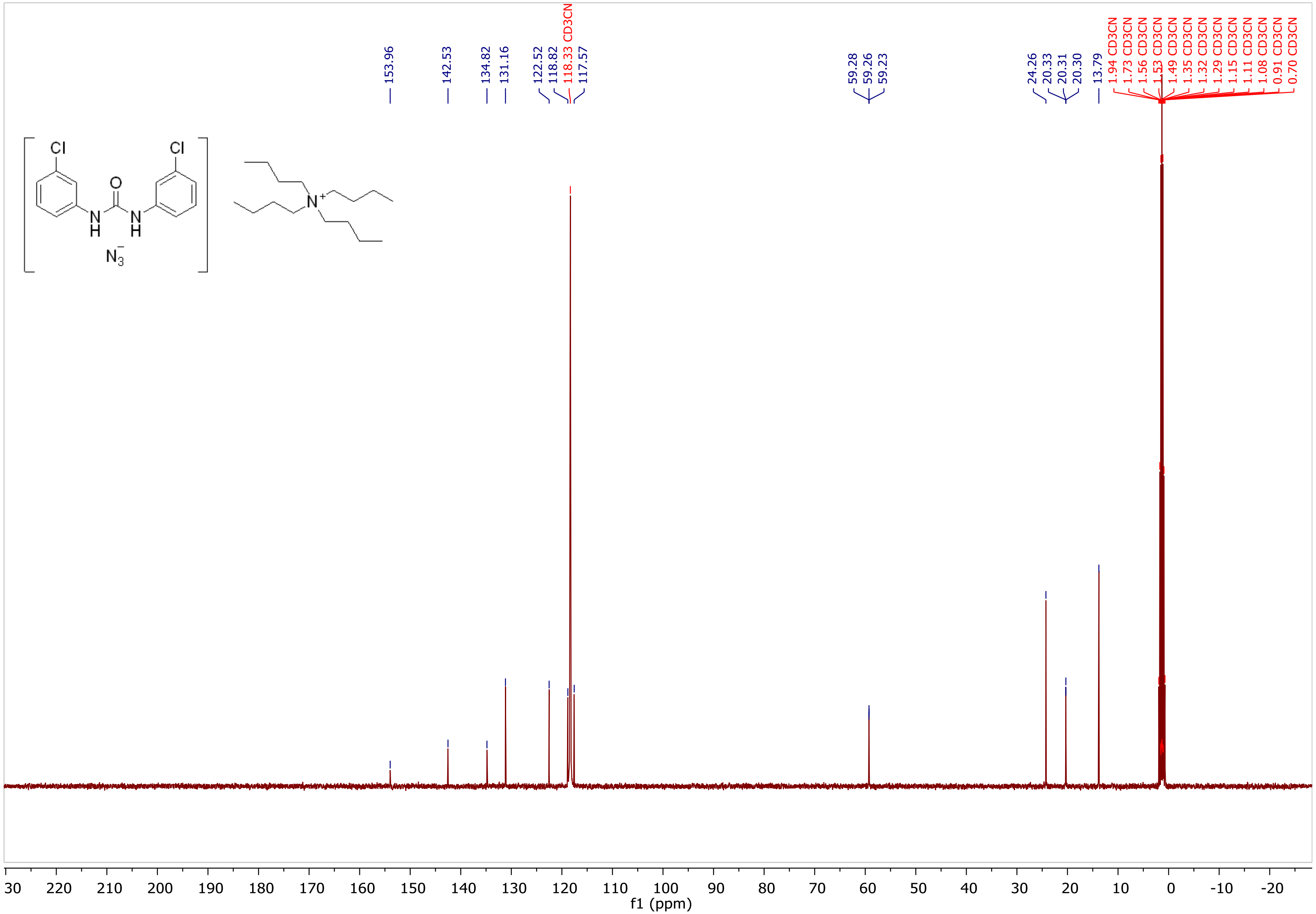

1-(3,5-bis(trifluoromethyl)phenyl)-3-phenylurea·tetrabutylammonium azide (1c·N_3·Bu_4N)

^{1}H NMR (400 MHz, CD_3CN)

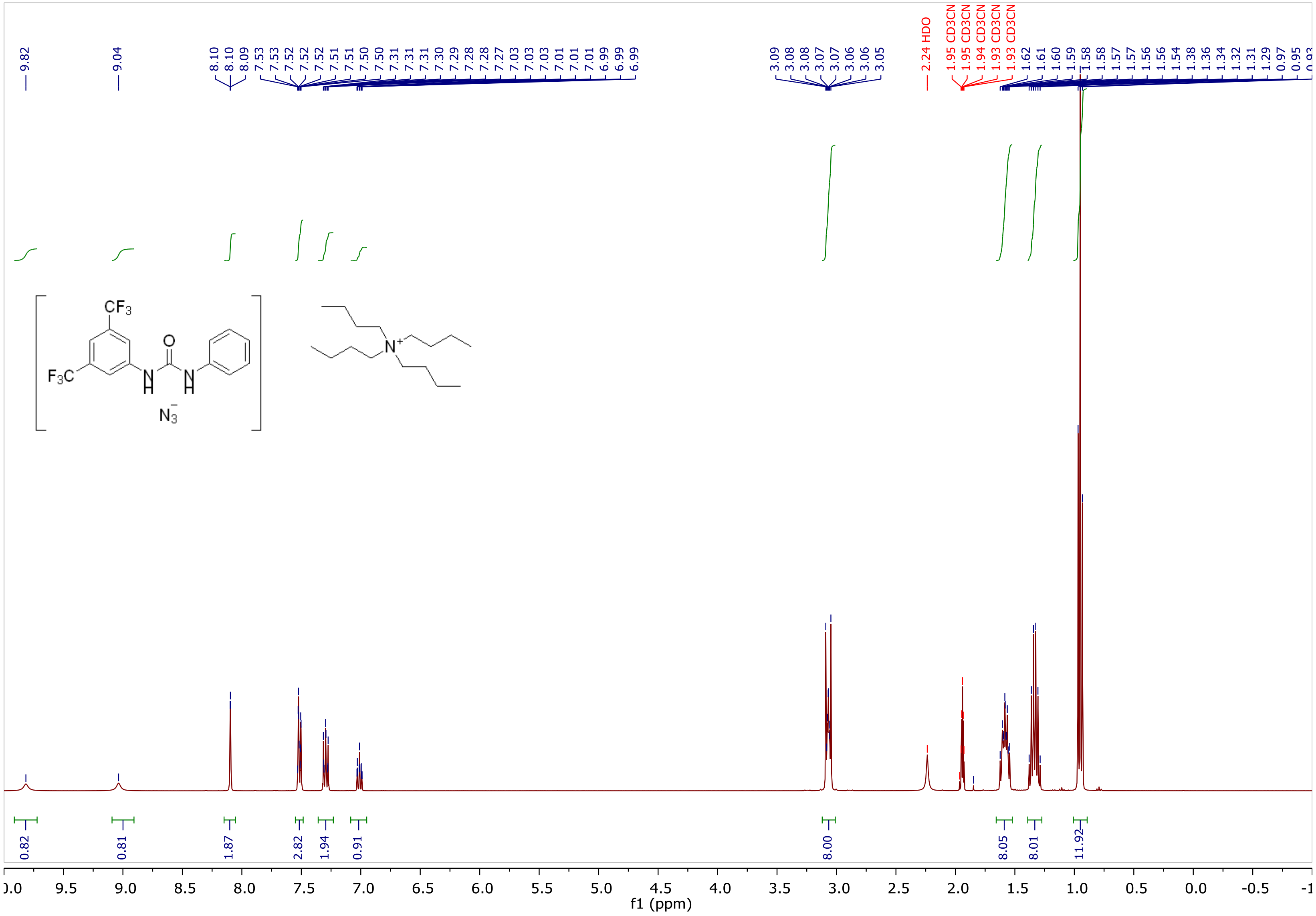

^{19}F NMR (377 MHz, CD_3CN)

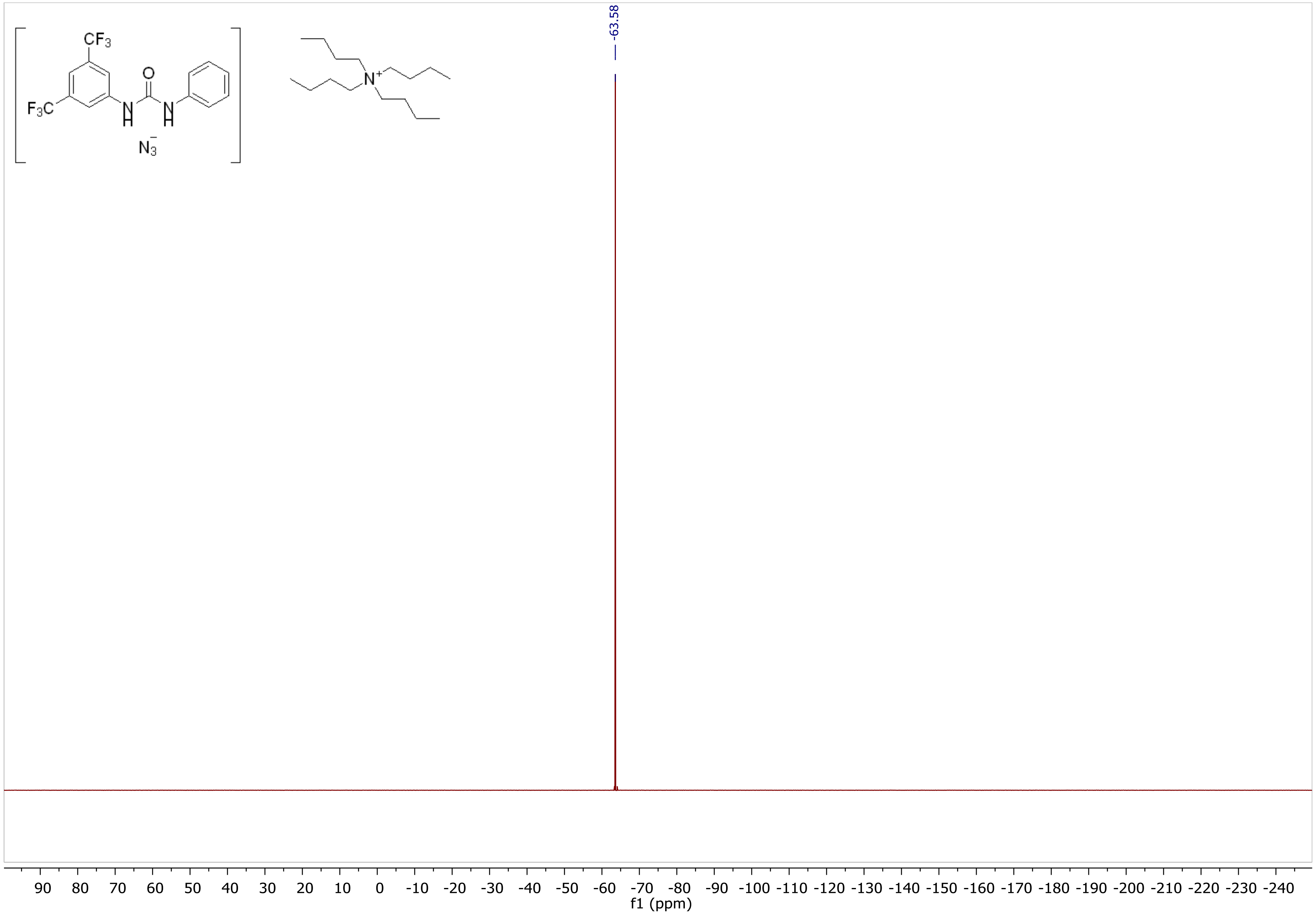

^{13}C NMR (151 MHz, CD_3CN)

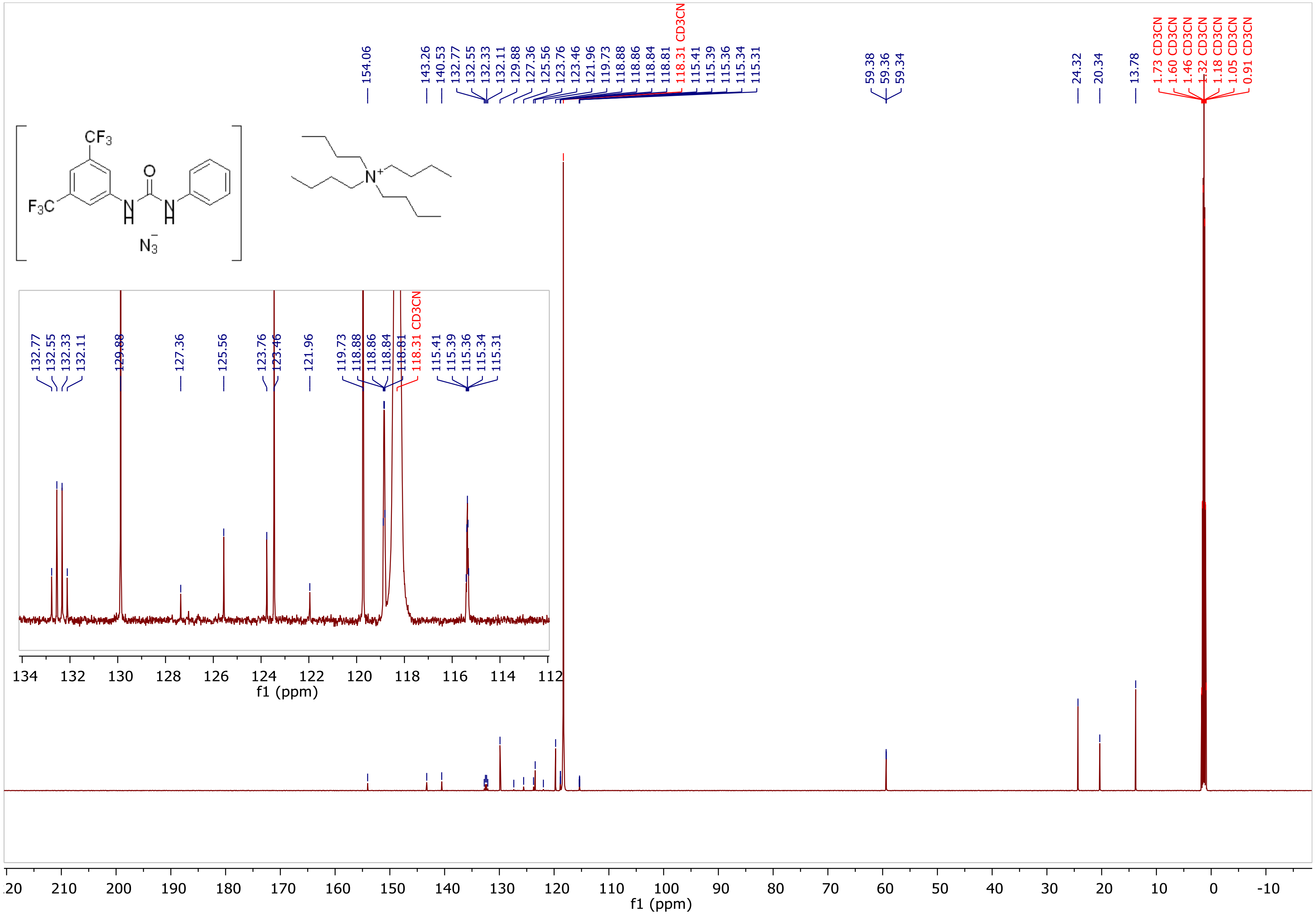

1,3-bis(4-trifluoromethylphenyl)urea·tetrabutylammonium azide (1d·N_3·Bu_4N)

^{1}H NMR (400 MHz, Acetone-d_6)

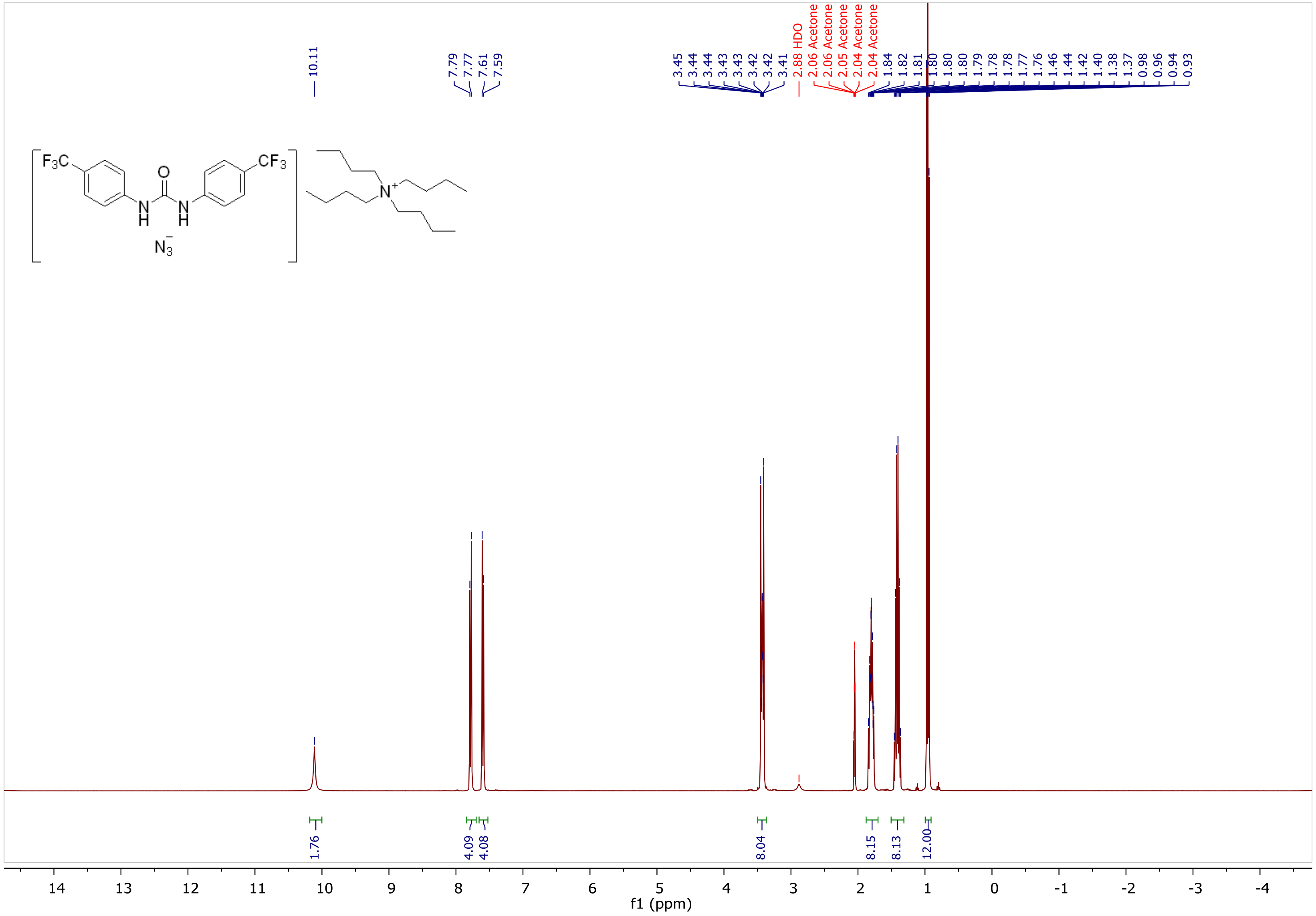

^{19}F NMR (376 MHz, Acetone-d_6)

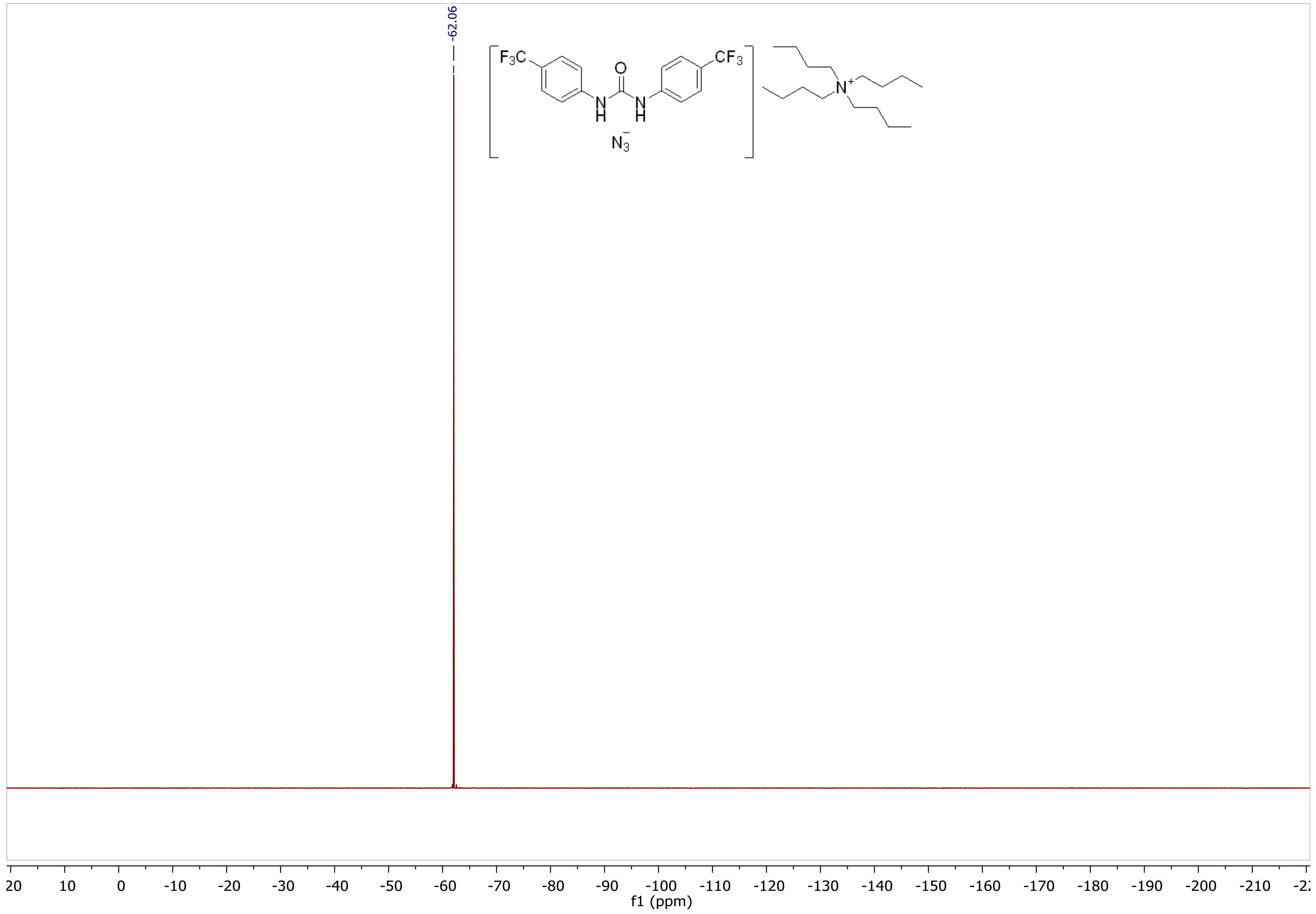

^{13}C NMR (101 MHz, Acetone-d_6)

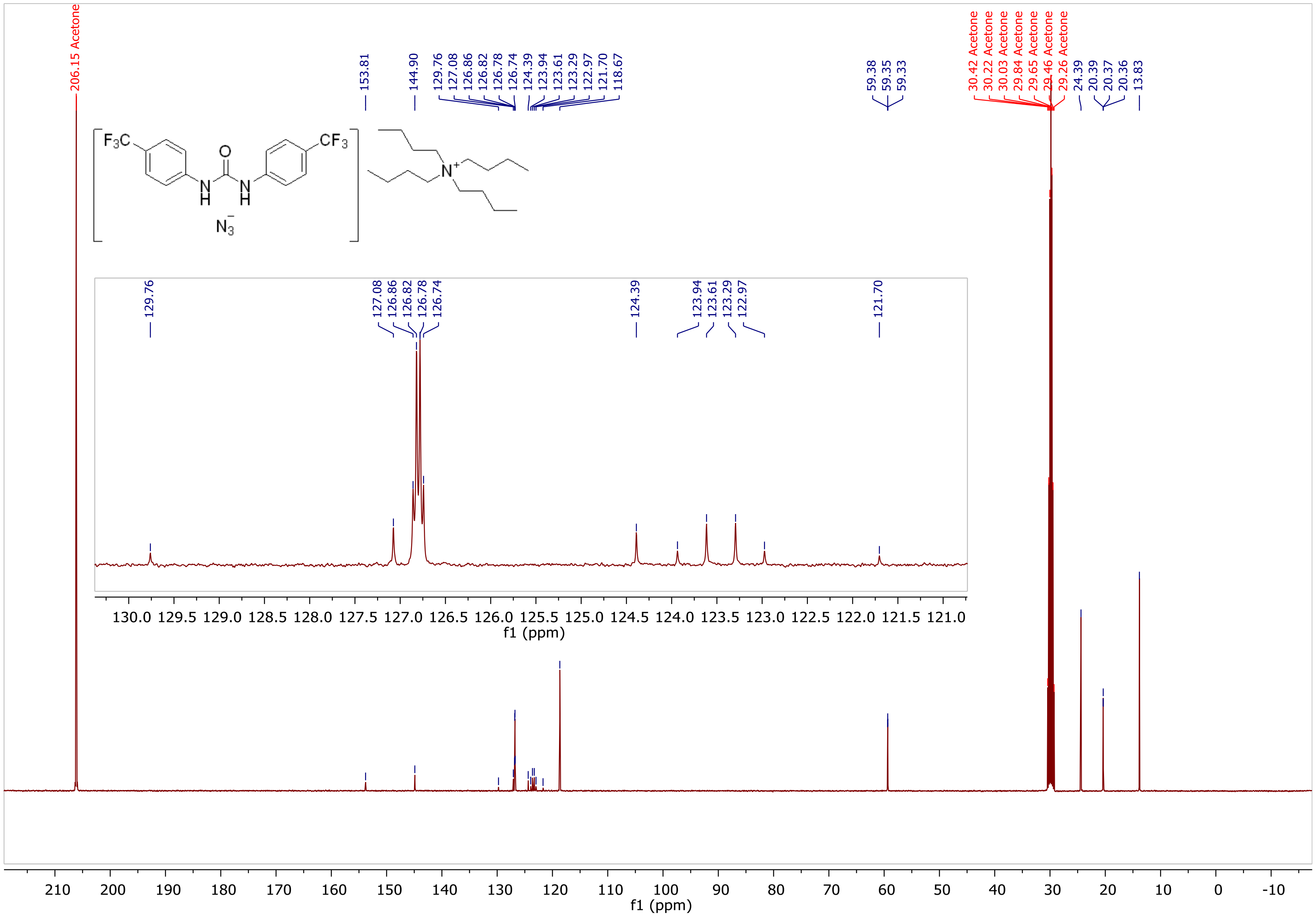

1,3-diphenylurea·tetrabutylammonium azide (1e·N_3·Bu_4N)

^{1}H NMR (400 MHz, Acetone-d_6)

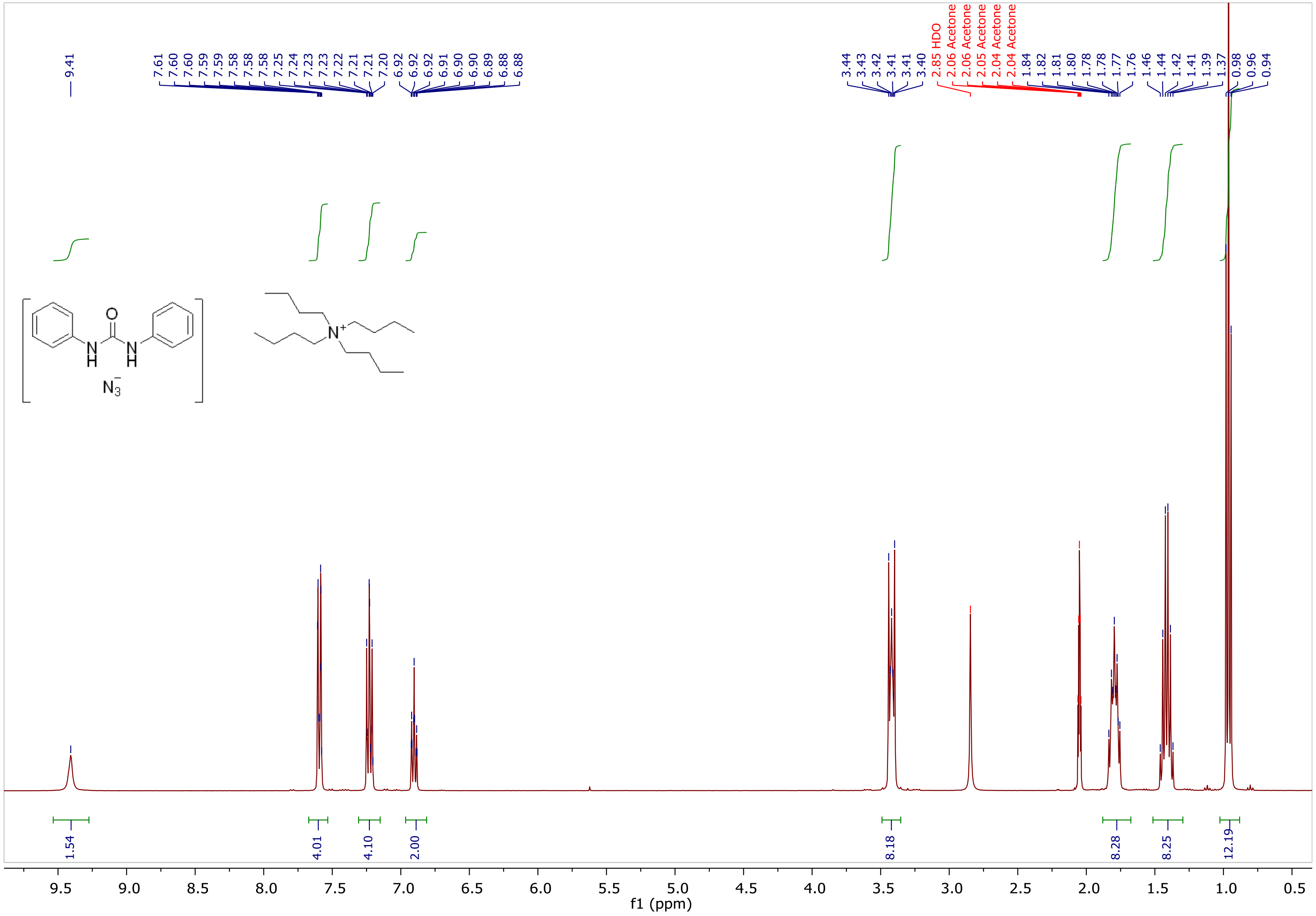

^{13}C NMR (101 MHz, Acetone-d_6)

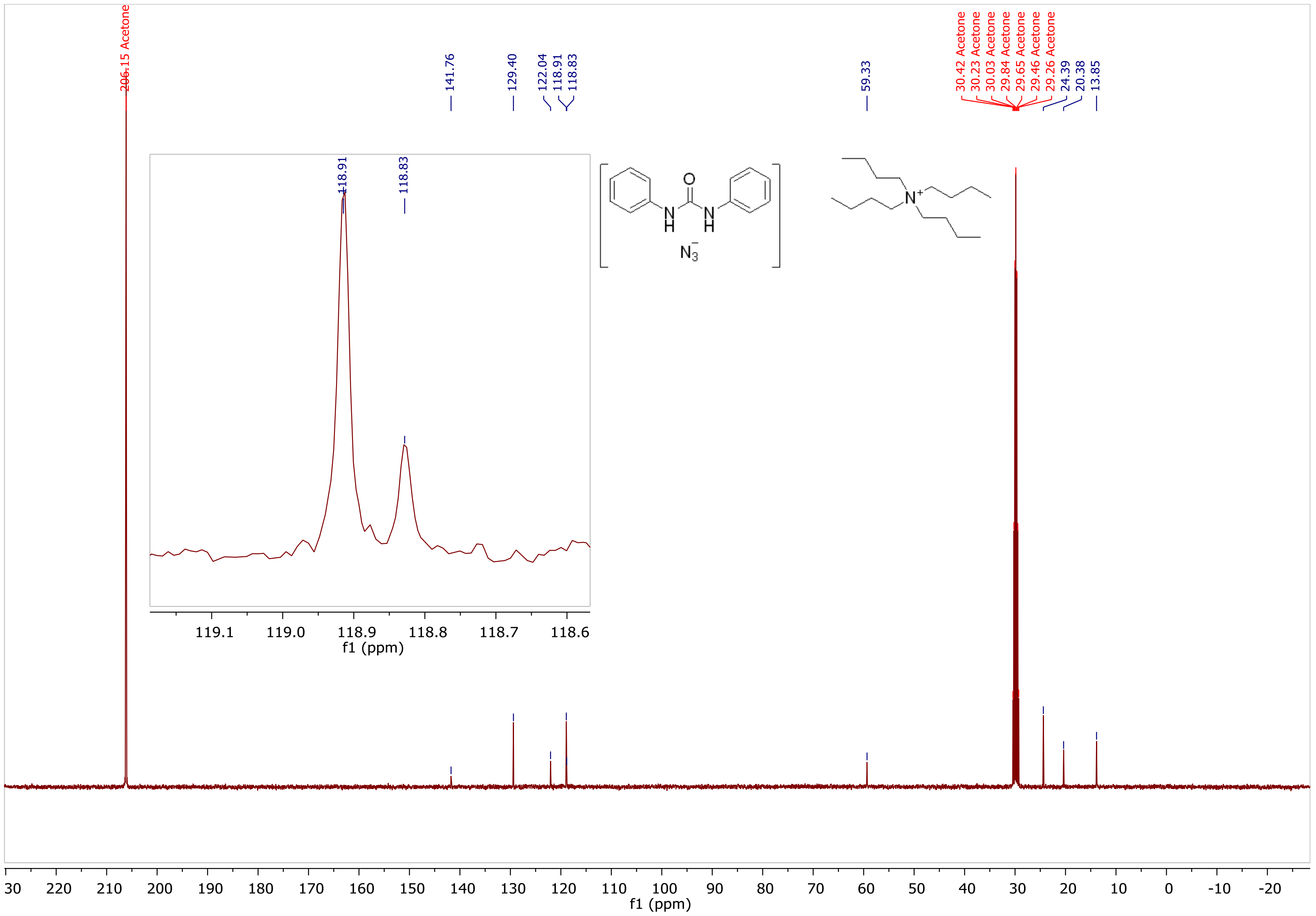

1,3-bis(4-bromophenyl)urea·tetrabutylammonium azide (1f·N_3·Bu_4N)

^{1}H NMR (400 MHz, DMSO-d_6)

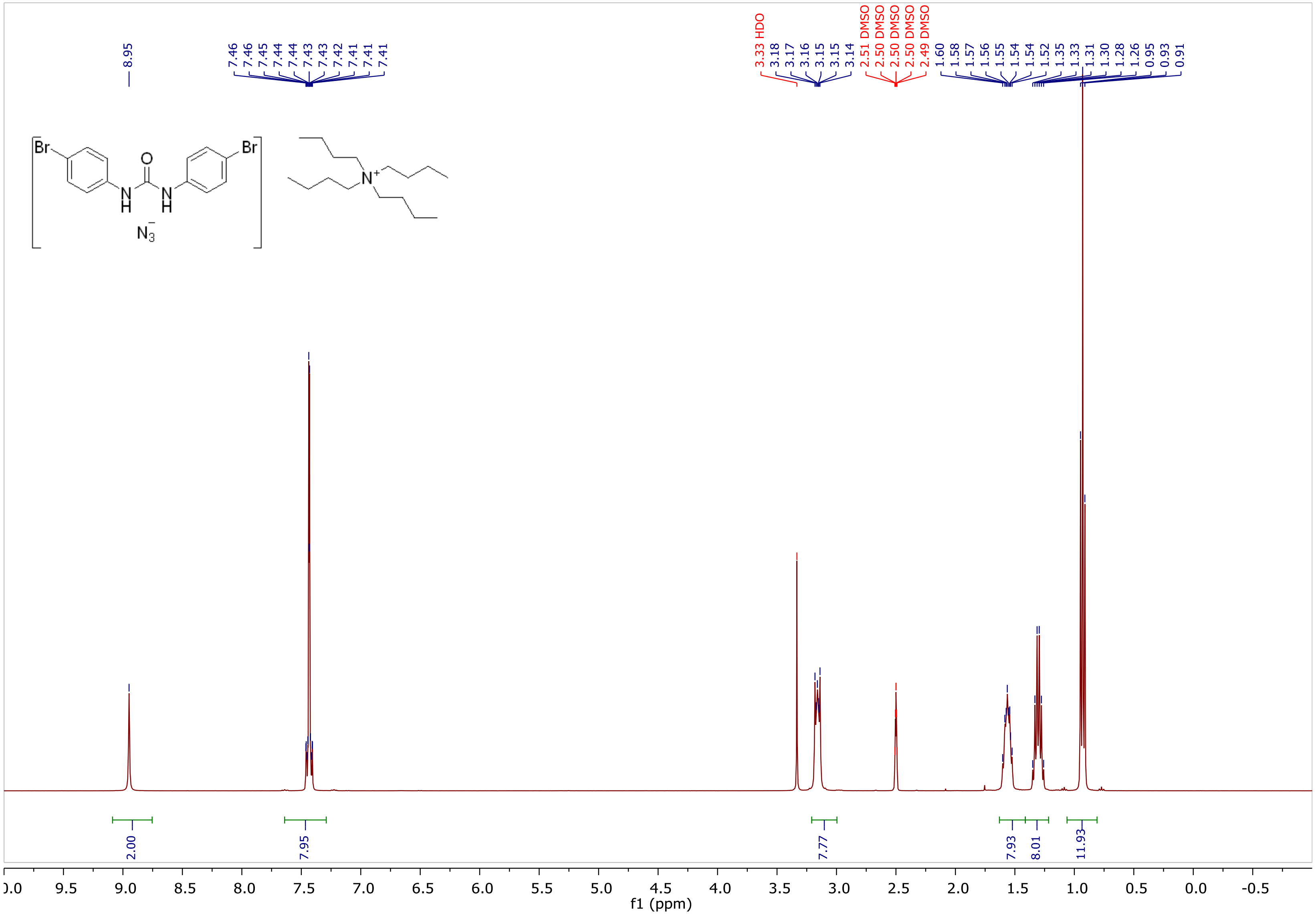

^{13}C NMR (101 MHz, DMSO-d_6)

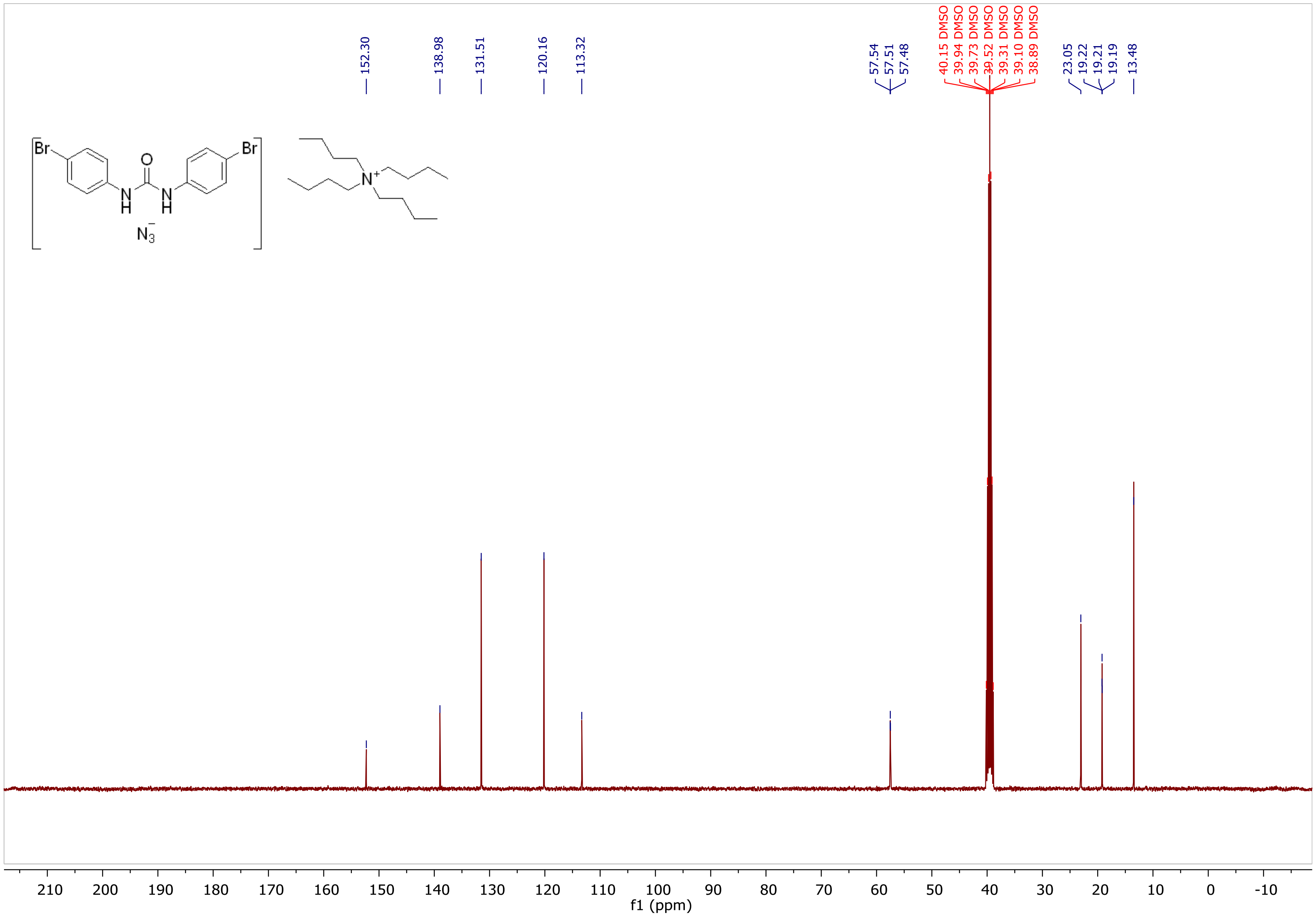

1,3-bis(4-fluorophenyl)urea·tetrabutylammonium azide (1g·N_3·Bu_4N)

^{1}H NMR (400 MHz, CD_3CN)

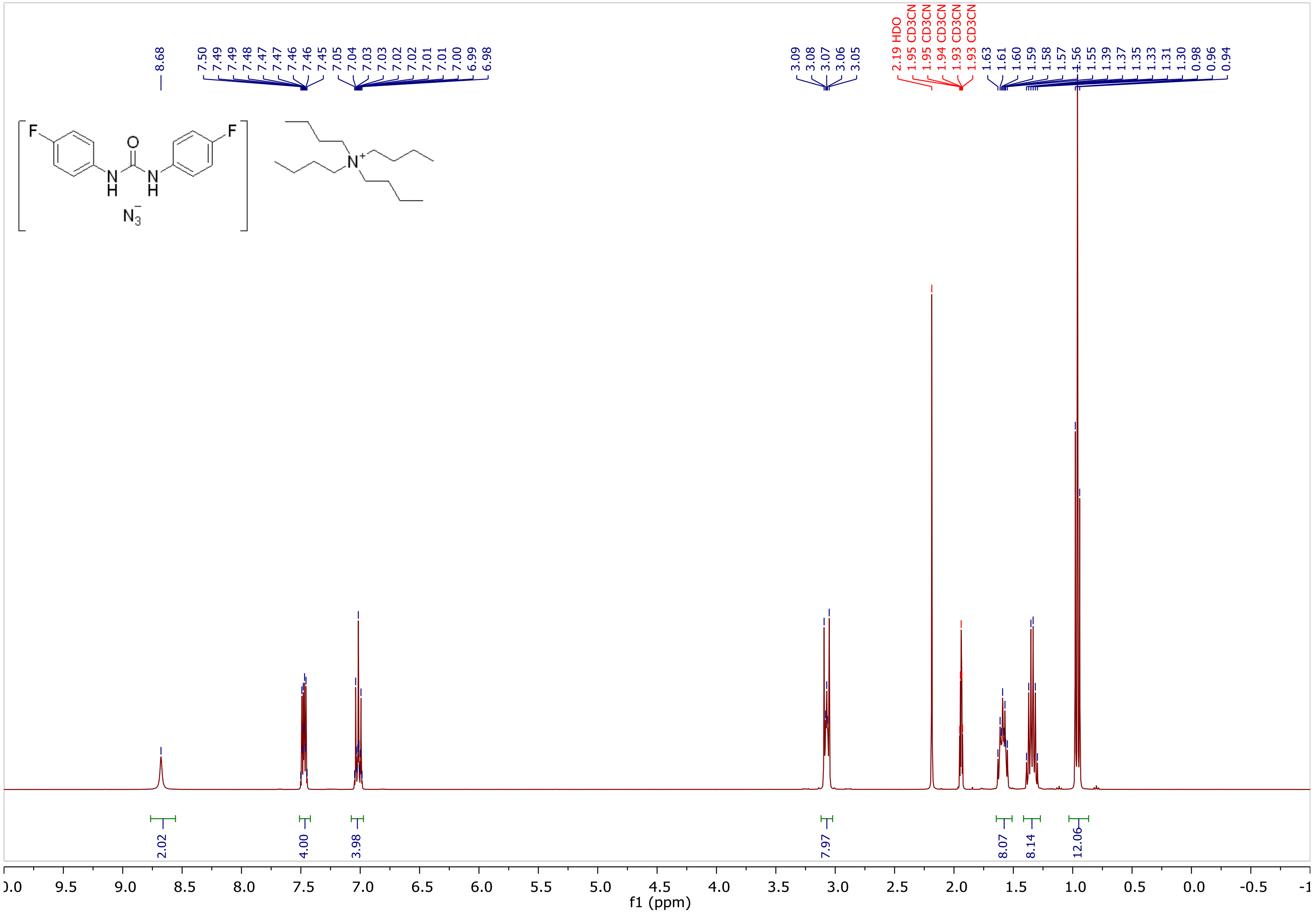

^{19}F NMR (376 MHz, CD_3CN)

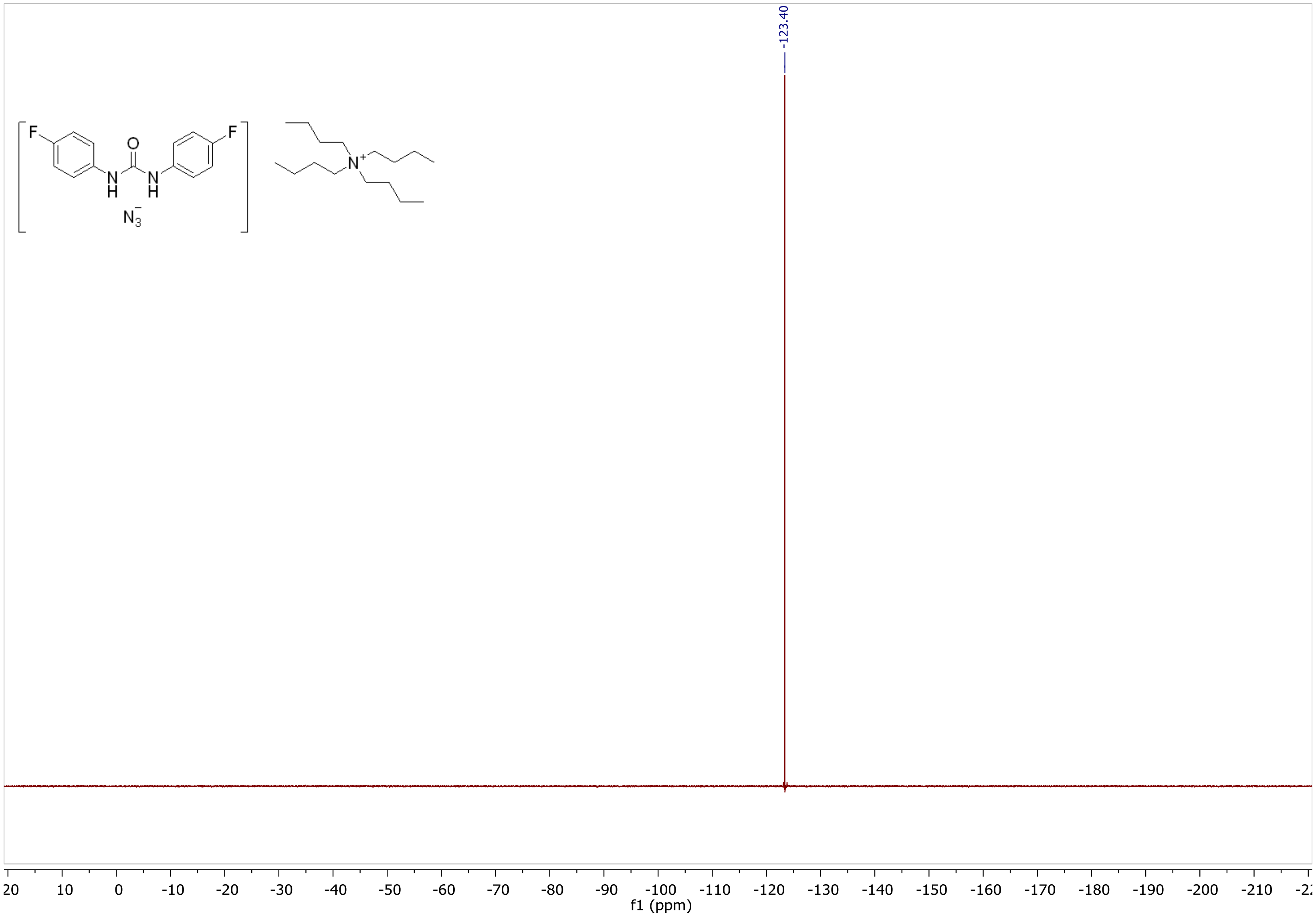

^{13}C NMR (101 MHz, CD_3CN)

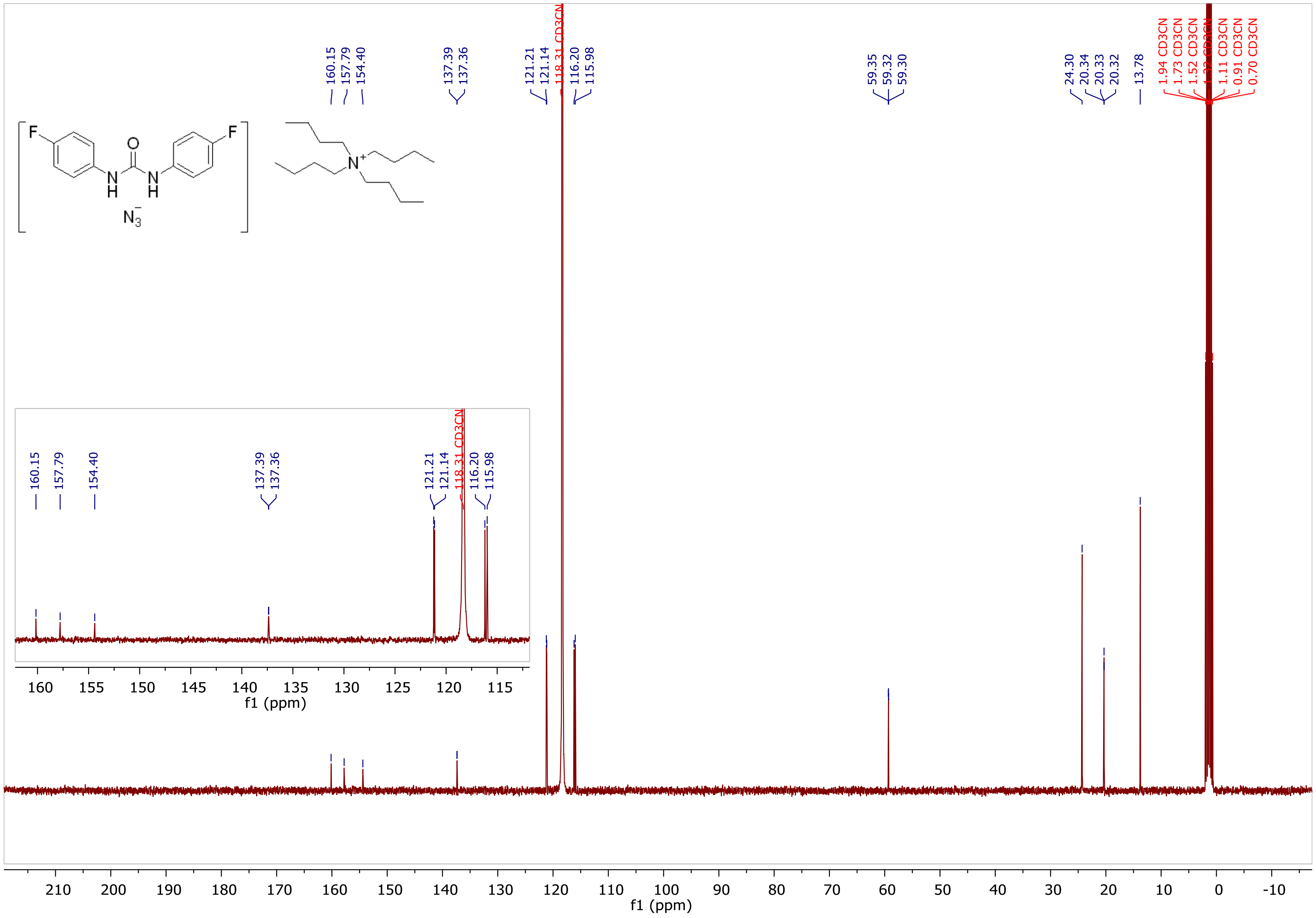

1,3-bis(4-cyanophenyl)urea·tetrabutylammonium azide (1h·N_3·Bu_4N)

^{1}H NMR (400 MHz, Acetone-d_6)

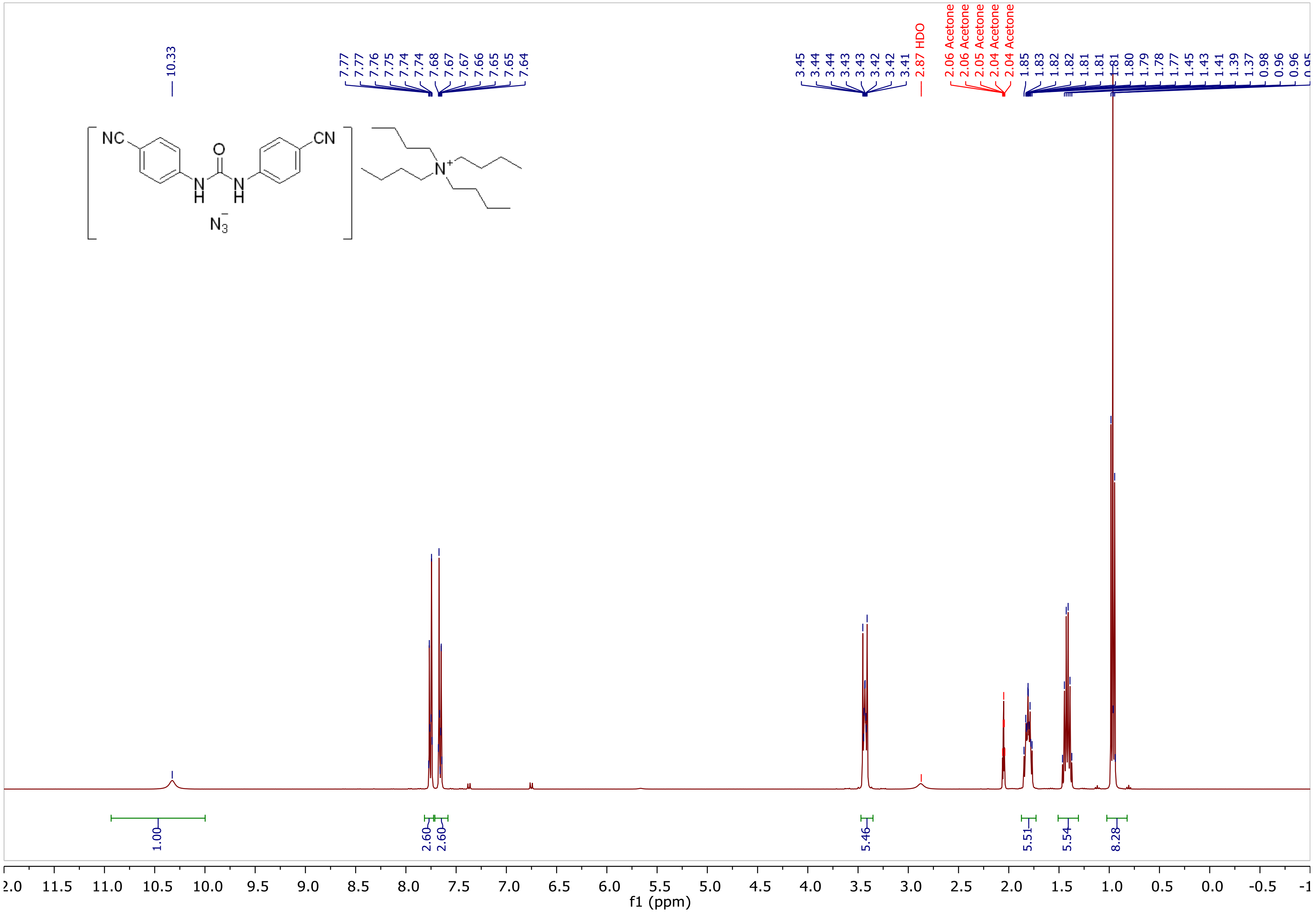

^{13}C NMR (101 MHz, Acetone-d_6)

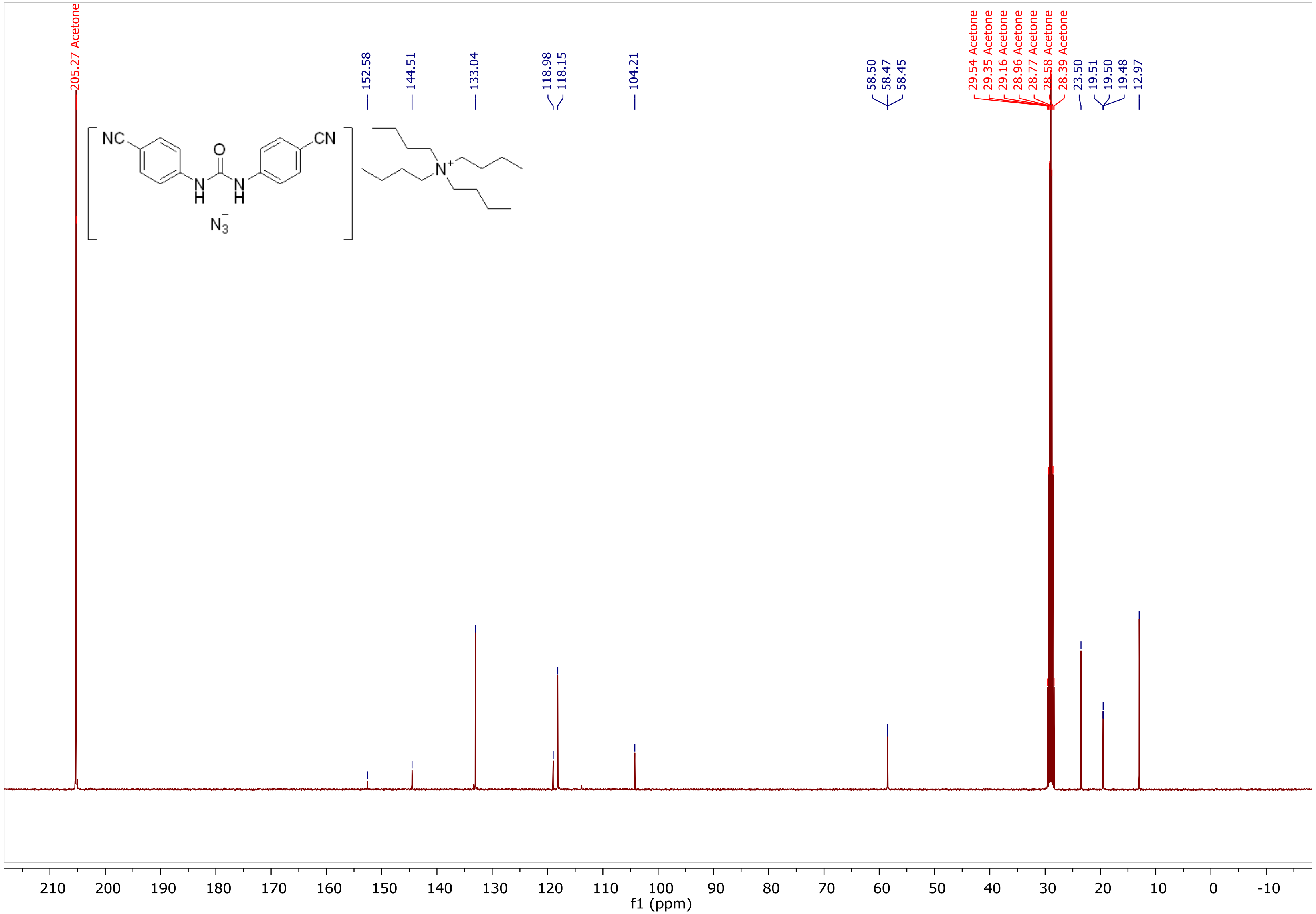

diphenylguanidine·tetrabutylammonium azide (1i·N_3·Bu_4N)

^{1}H NMR (400 MHz, $CDCl_3$)

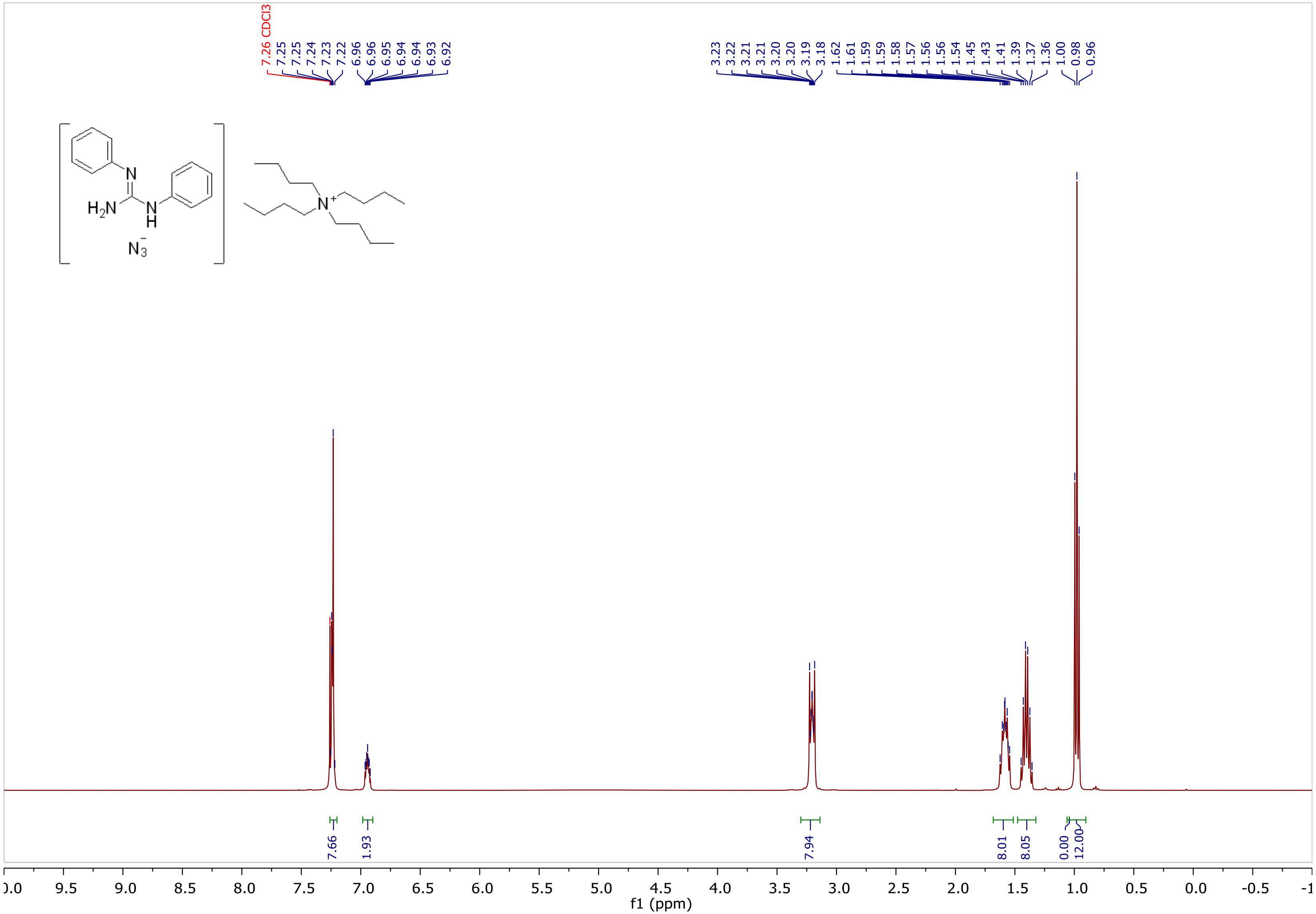

^{13}C NMR (101 MHz, $CDCl_3$)

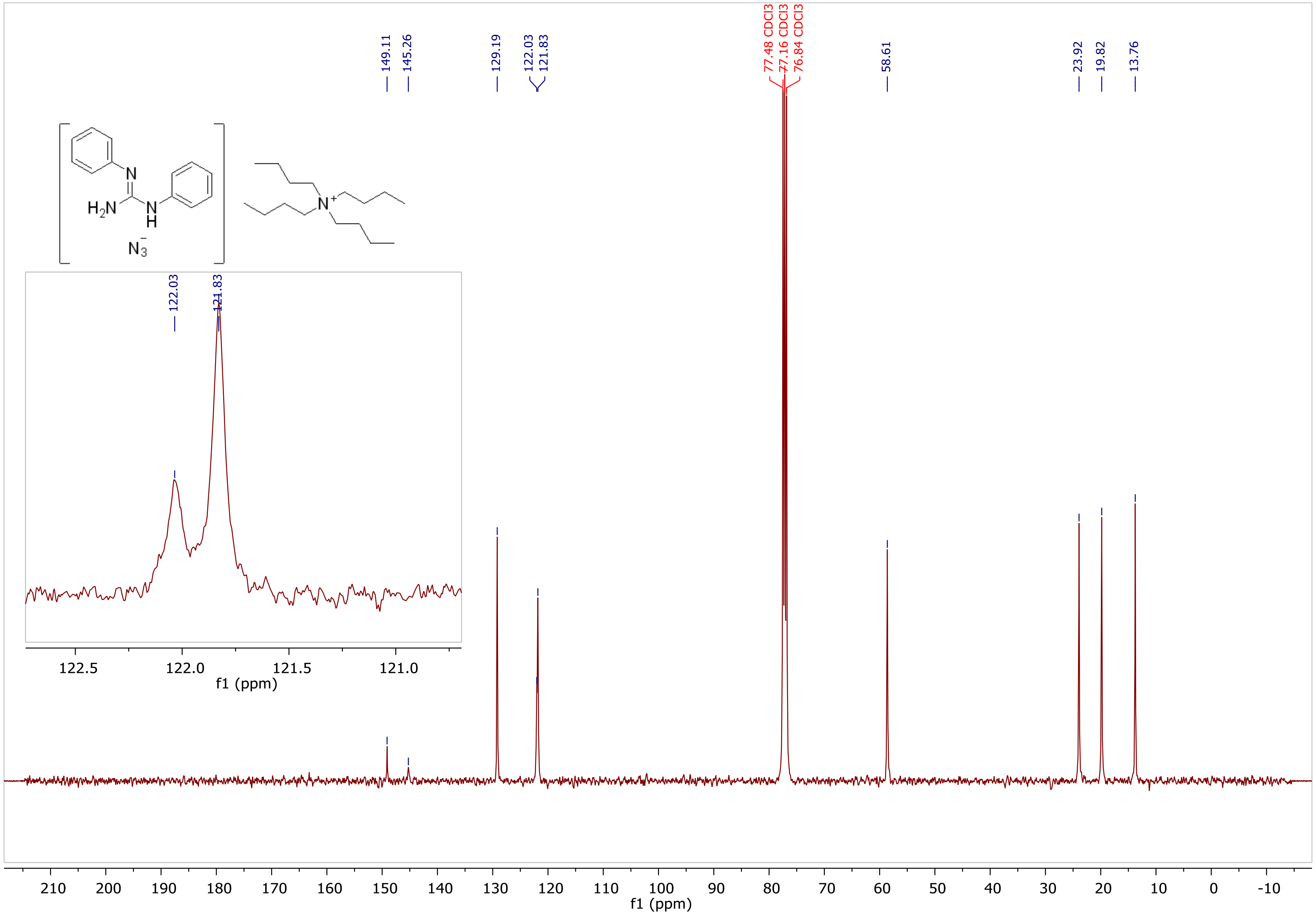

N^1,N^2-bis(3,5-bis(trifluoromethyl)phenyl)oxalamide·tetrabutylammonium azide (1j·N_3·Bu_4N)

^{1}H NMR (500 MHz, Acetone-d_6)

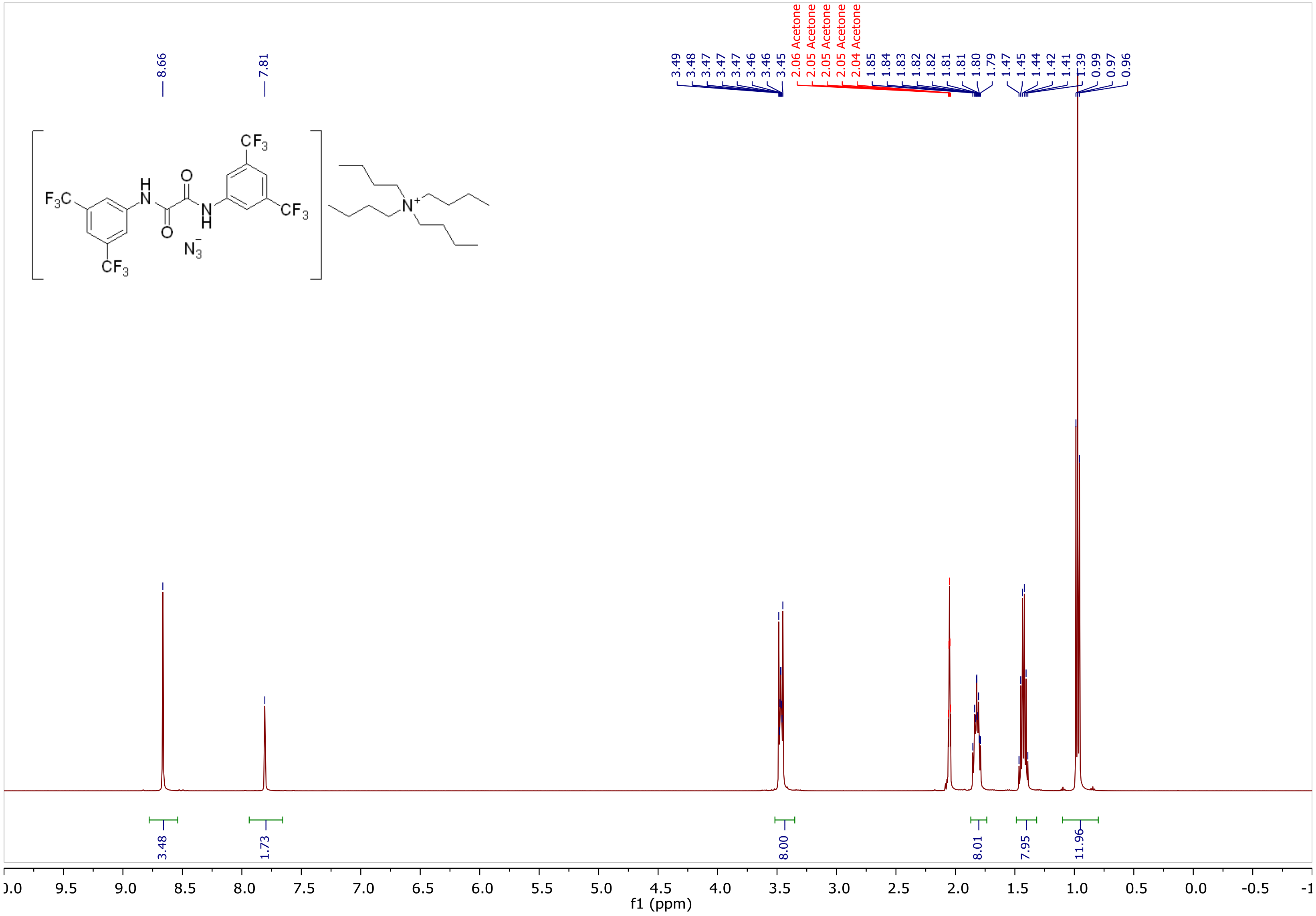

^{19}F NMR (470 MHz, Acetone-d_6)

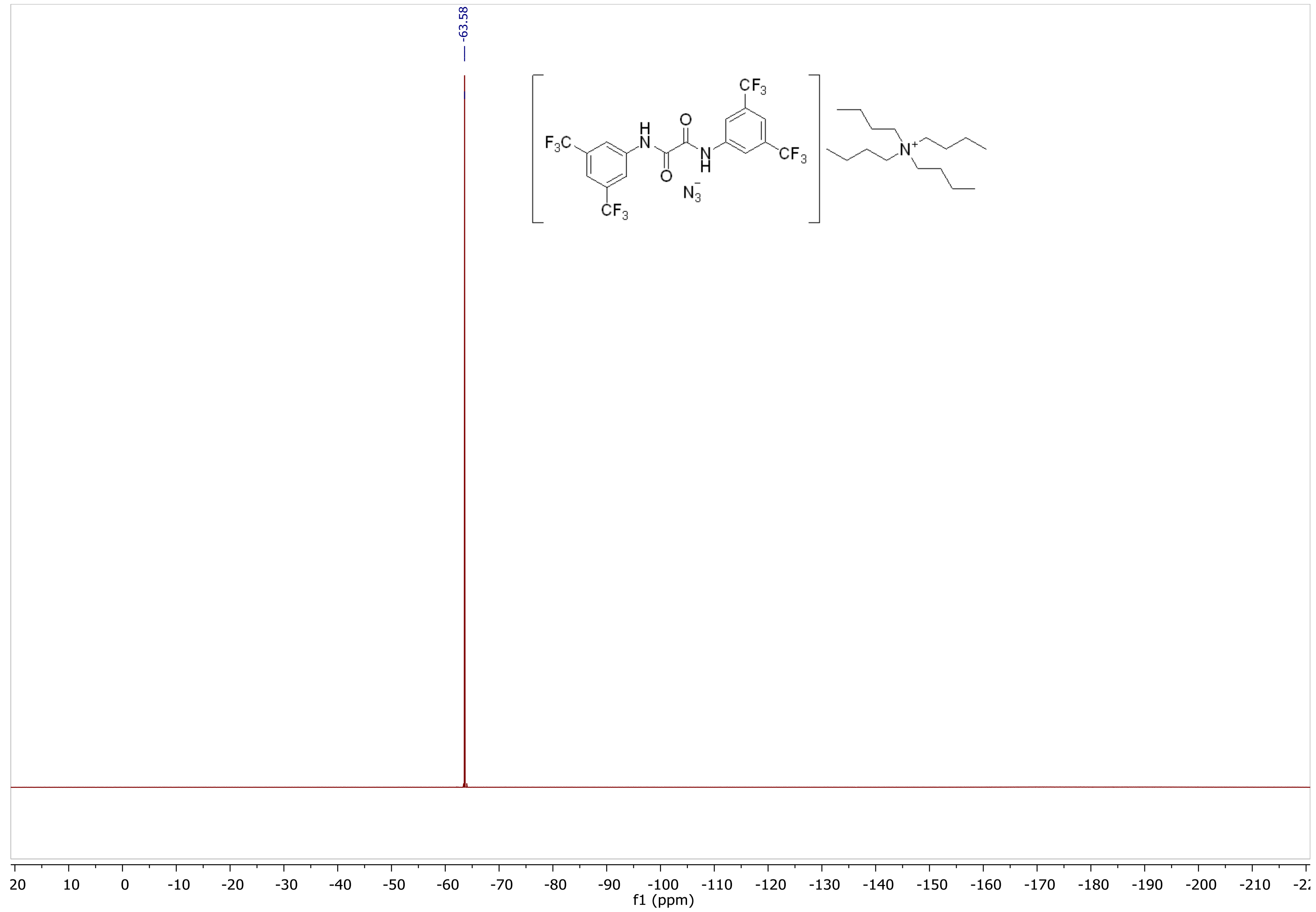

^{13}C NMR (126 MHz, Acetone-d_6)

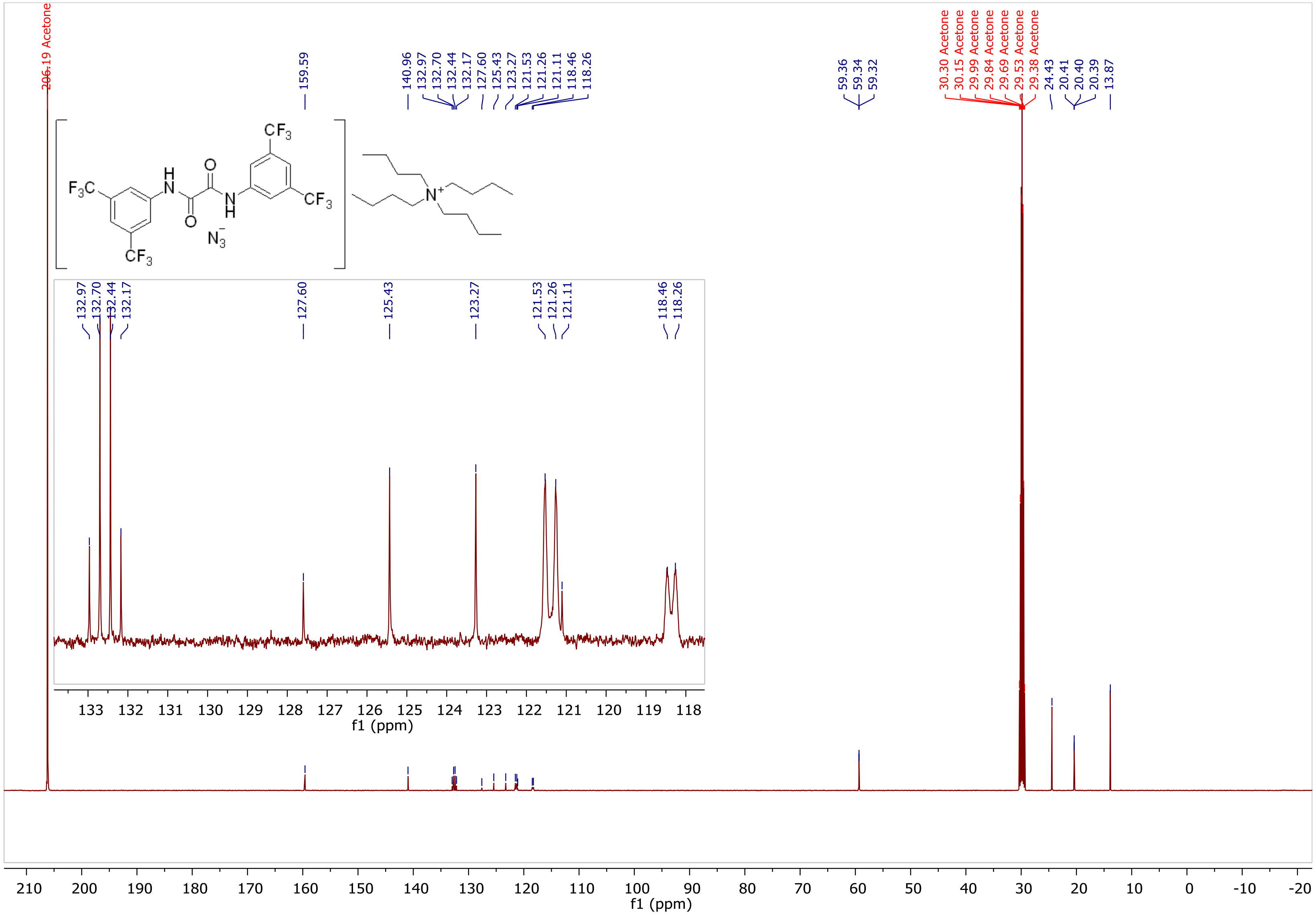

(±)-3-(3,5-bis(trifluoromethyl)phenyl)-1-(2'-(3-(3,5-bis(trifluoromethyl)phenyl)ureido)-[1,1'-binaphthalen]-2-yl)-1-isopropylurea·tetrabutylammonium azide ((±)-1k·N_3·Bu_4N)

^{1}H NMR (500 MHz, $CDCl_3$)

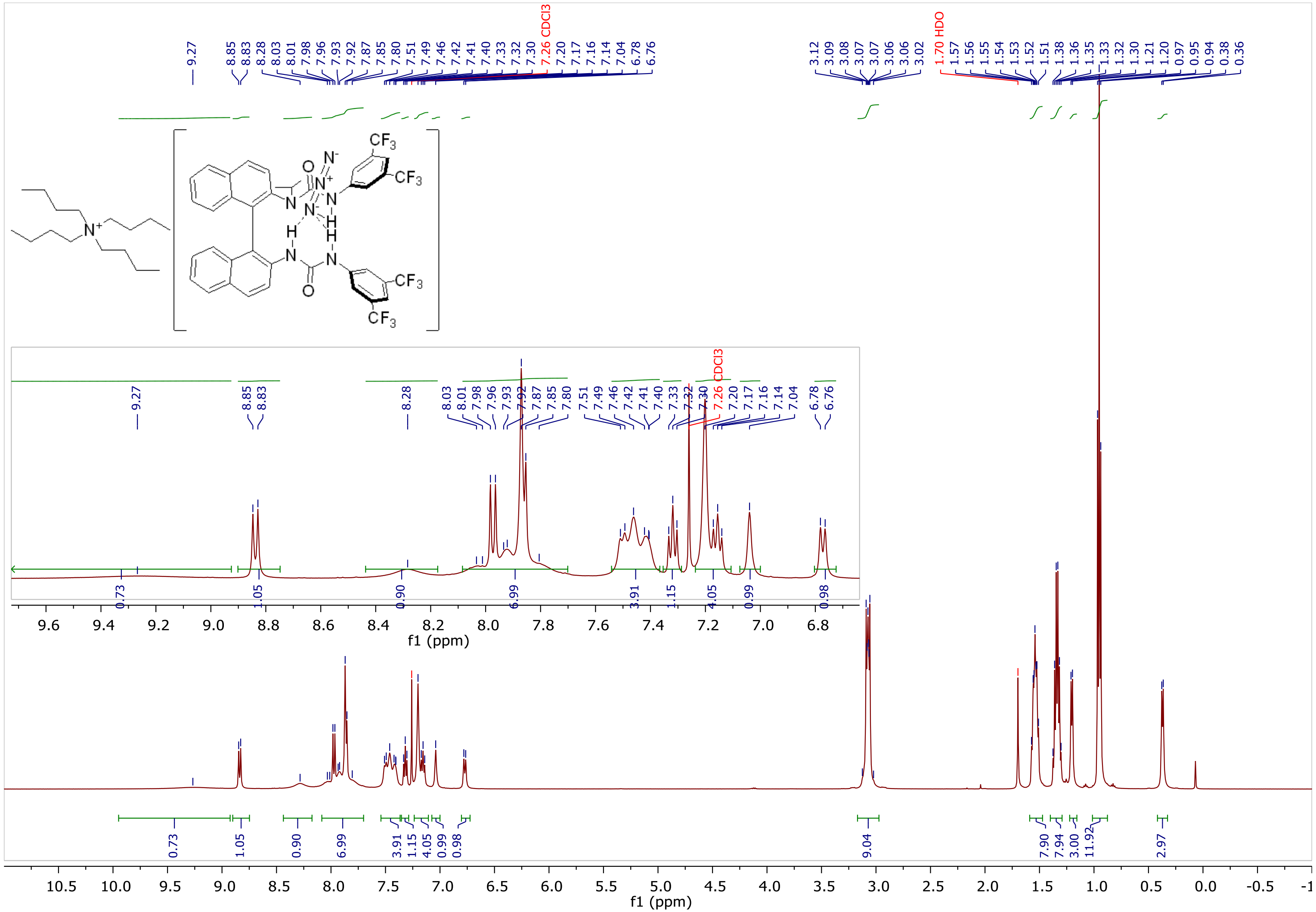

^{19}F NMR (471 MHz, $CDCl_3$)

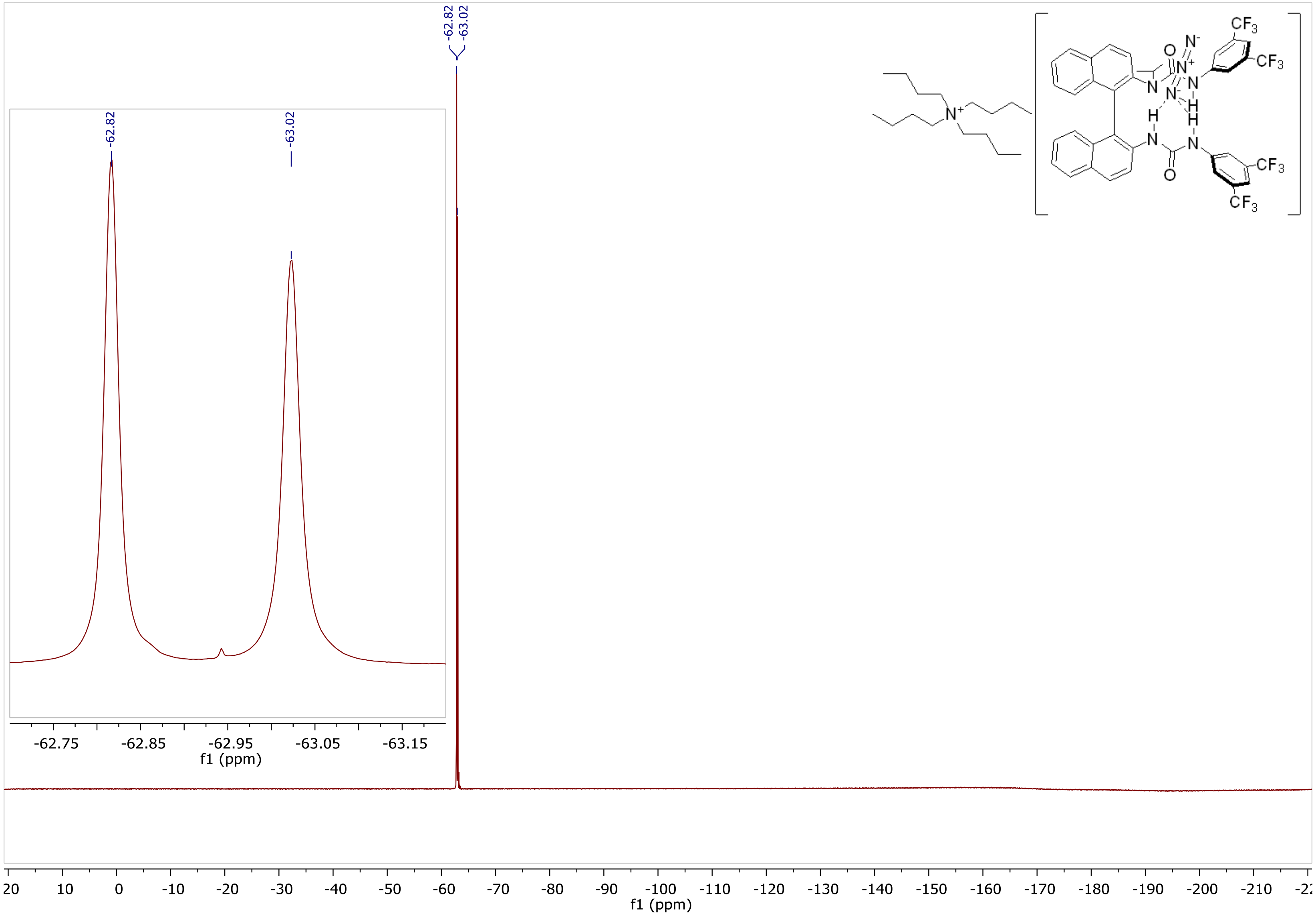

^{13}C NMR (126 MHz, $CDCl_3$)

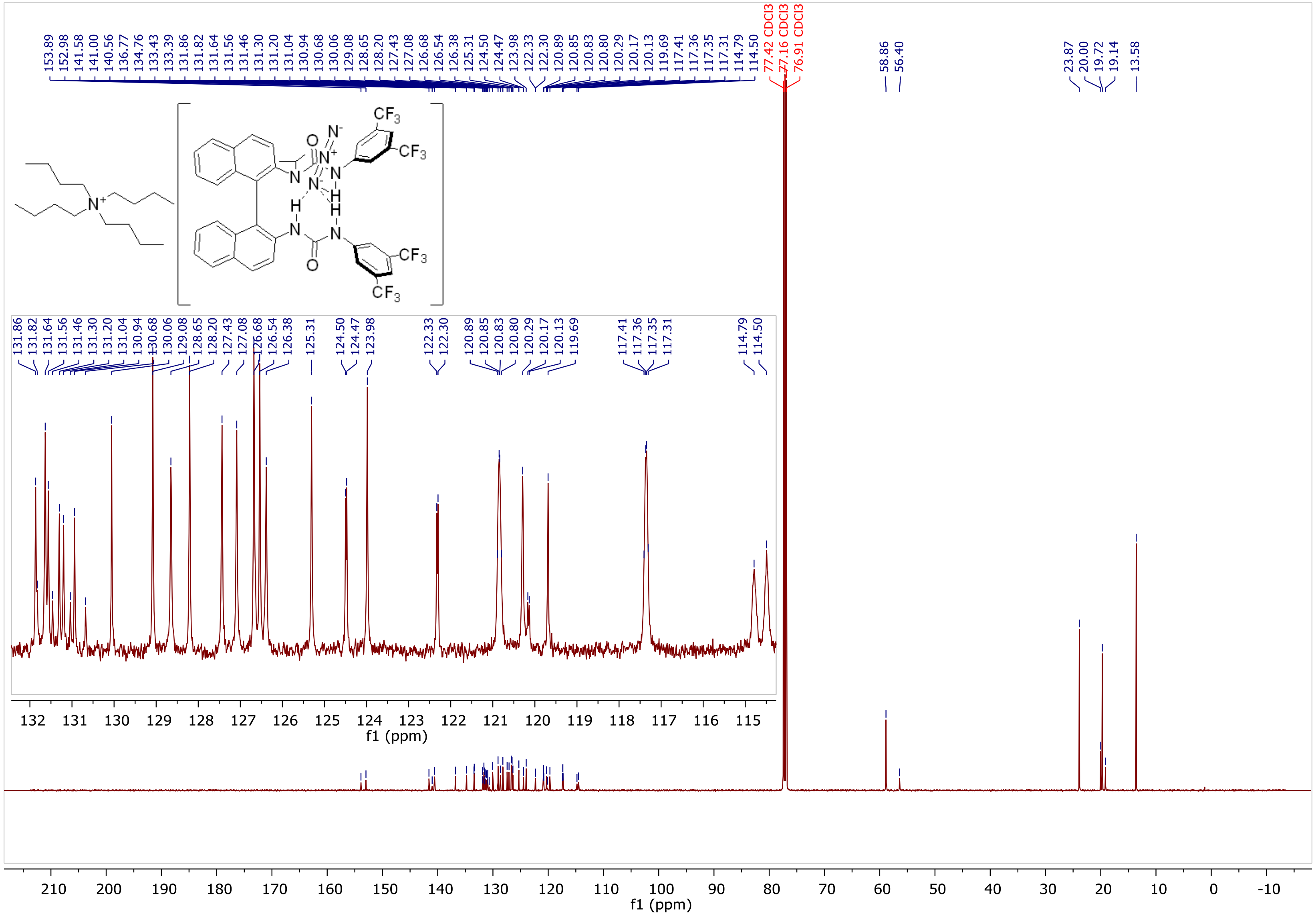

(±)-2-(4-methoxypiperidin-1-yl)-1,2-diphenylethan-1-ol

^{1}H NMR (400 MHz, $CDCl_3$)

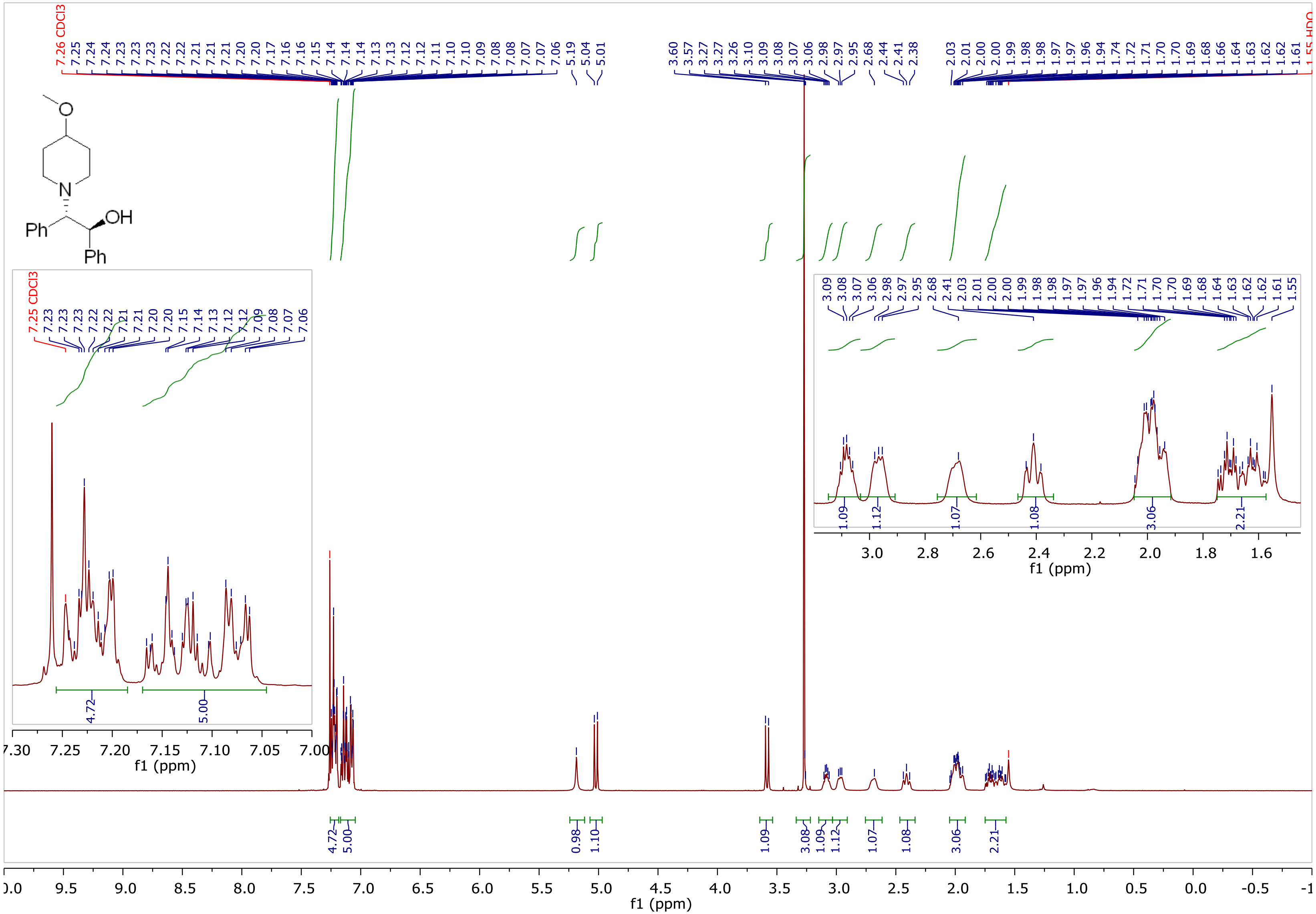

^{13}C NMR (101 MHz, $CDCl_3$)

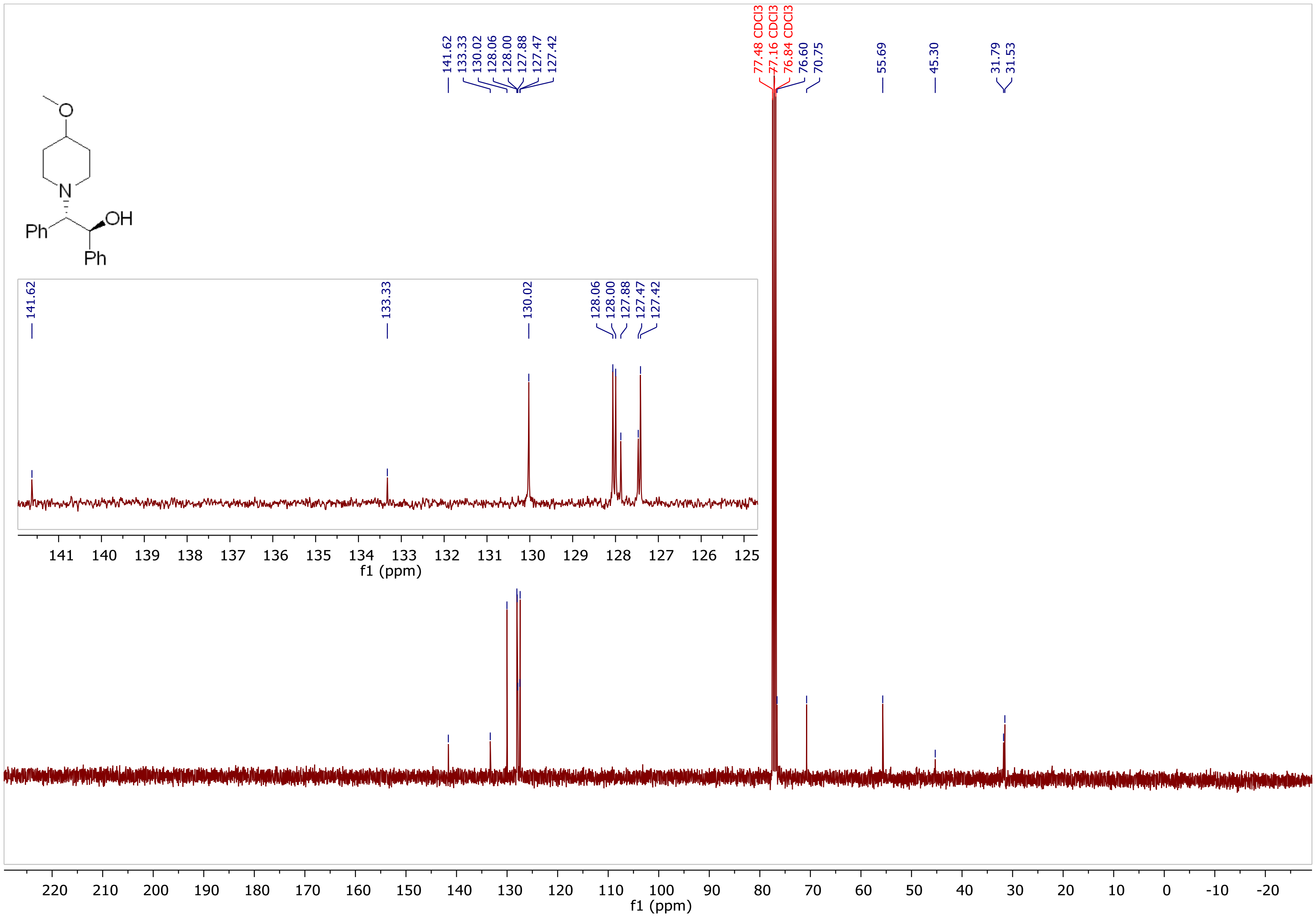
141.62
133.33
130.02
128.06
128.00
127.88
127.47
127.42
77.48 CDCl3
77.16 CDCl3
76.84 CDCl3
76.60
70.75
55.69
45.30
31.79
31.53
OH
Ph
Ph
N
O
f1 (ppm)
f1 (ppm)

(±)-1-(2-chloro-1,2-diphenylethyl)-4-methoxypiperidine (2b)

^{1}H NMR (400 MHz, $CDCl_3$)

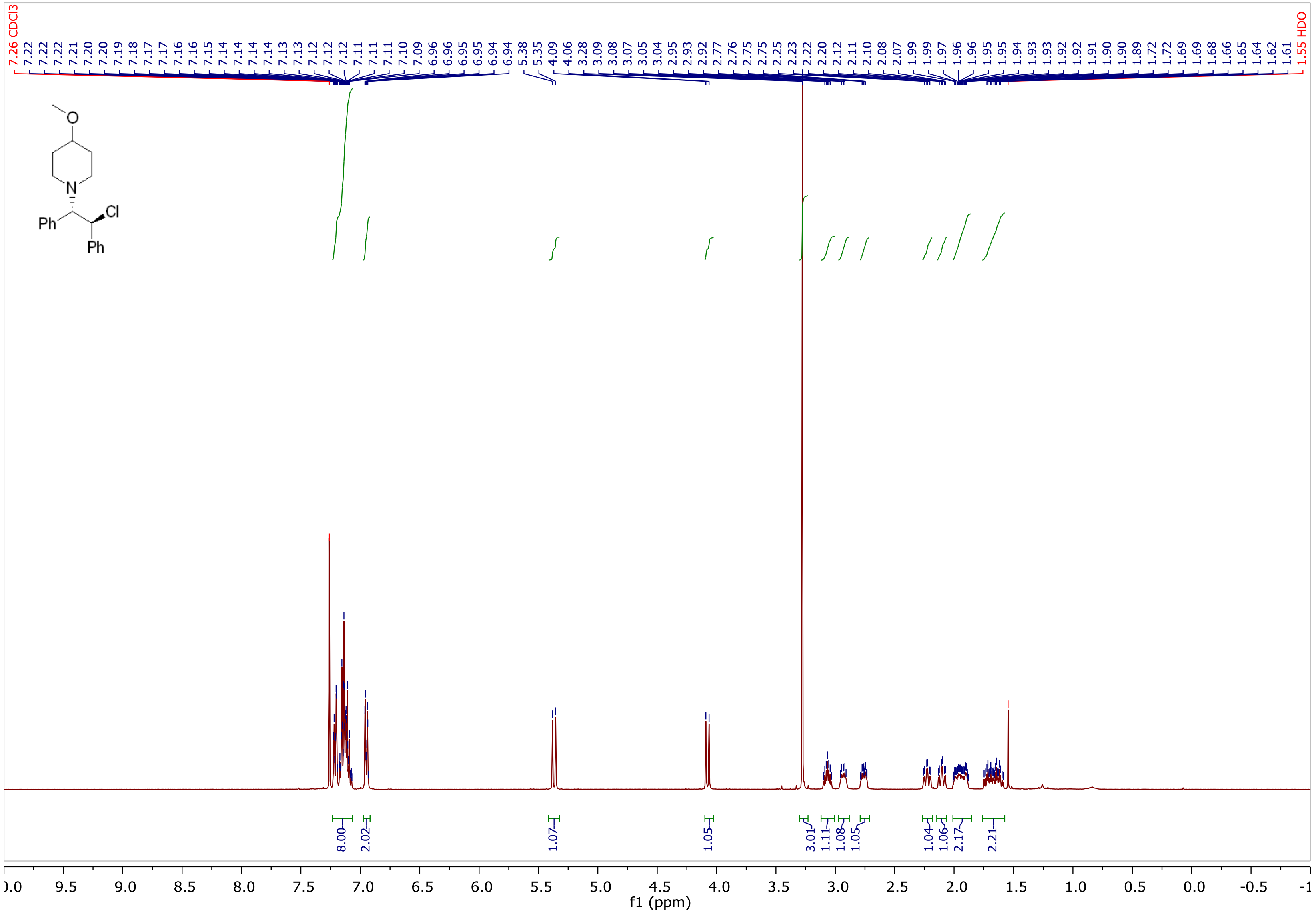

^{13}C NMR (101 MHz, $CDCl_3$)

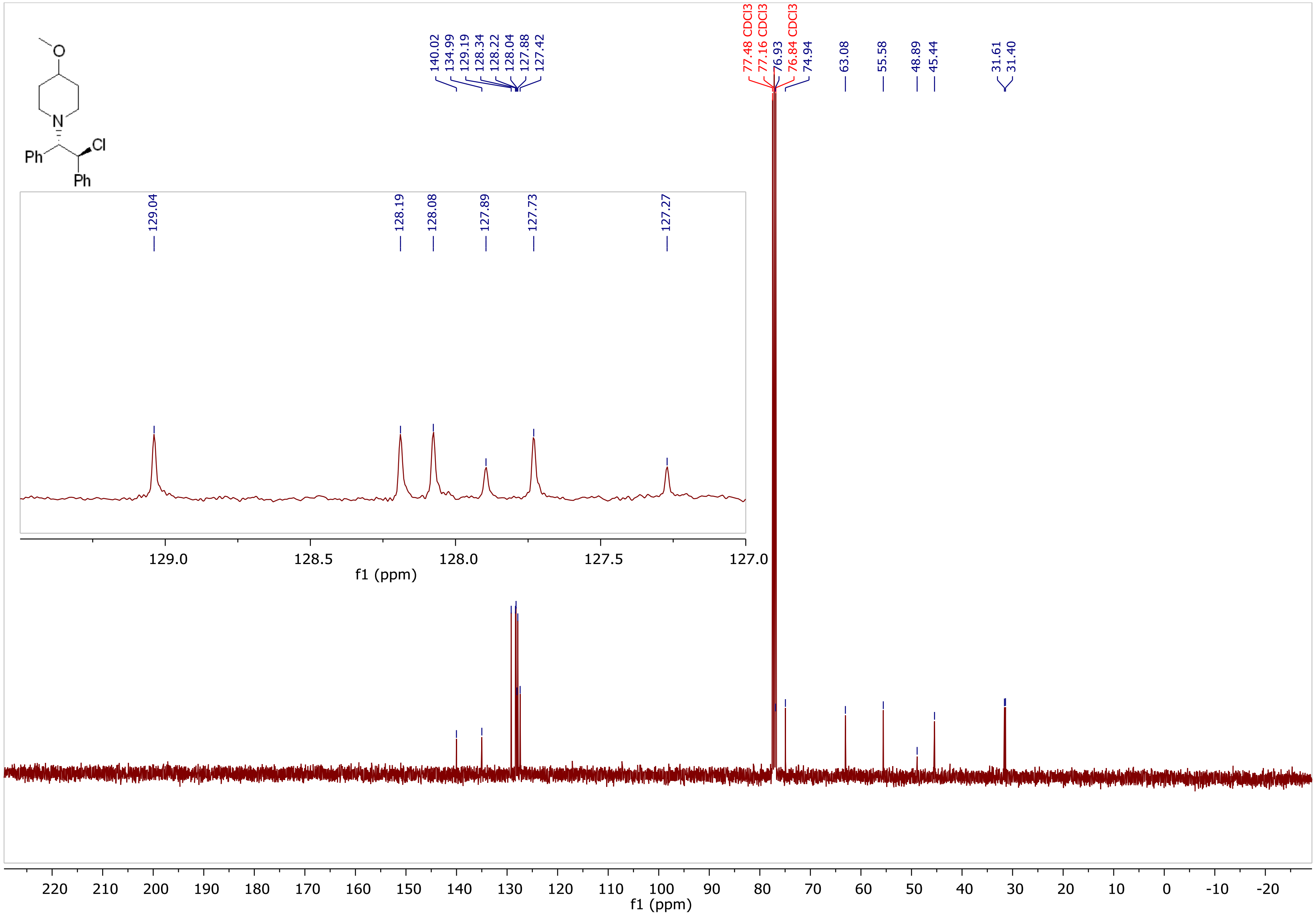

(±)-2-(4,4-difluoropiperidin-1-yl)-1,2-diphenylethan-1-ol

^{1}H NMR (500 MHz, DMSO-d_6)

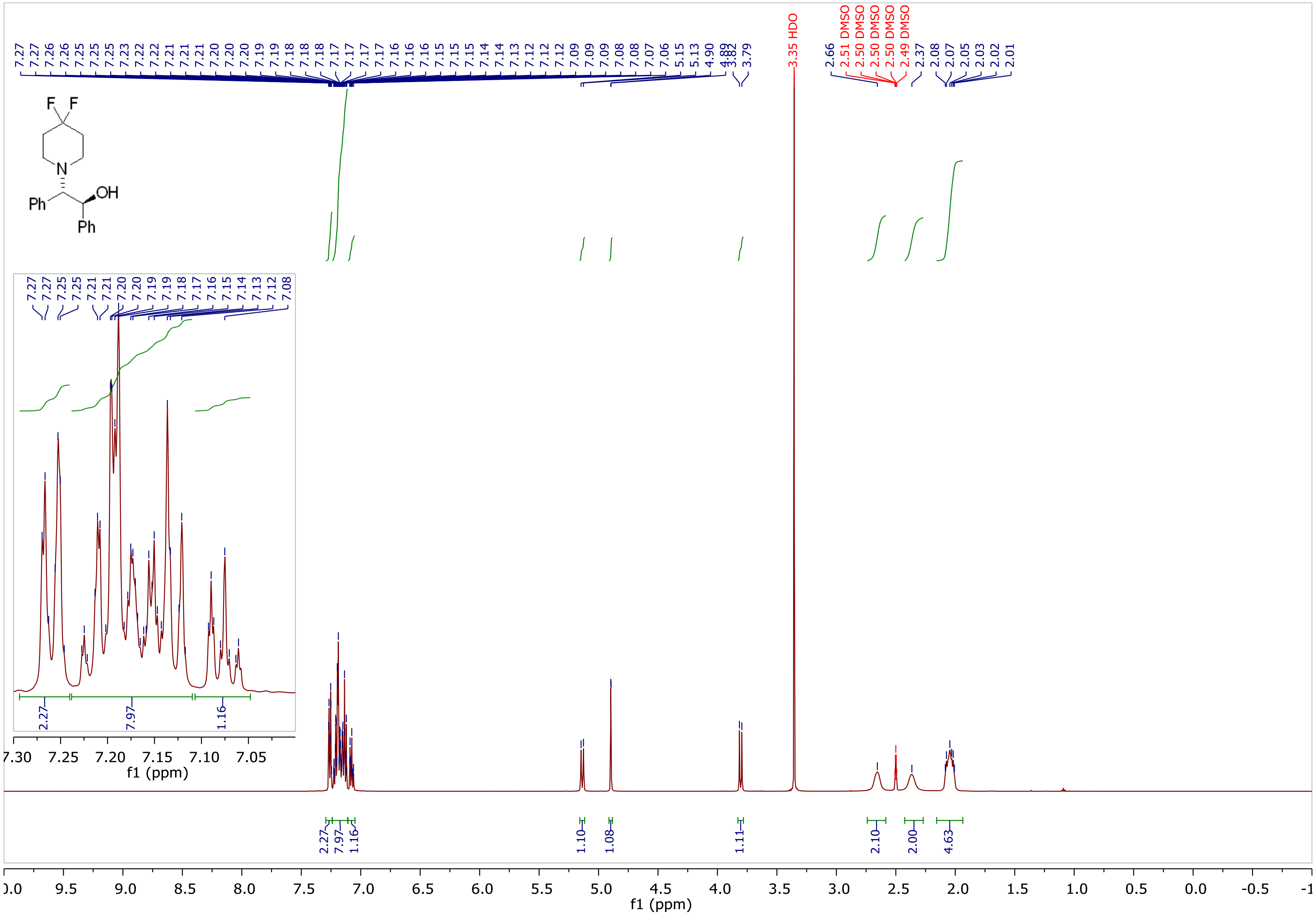

^{19}F NMR (471 MHz, DMSO-d_6)

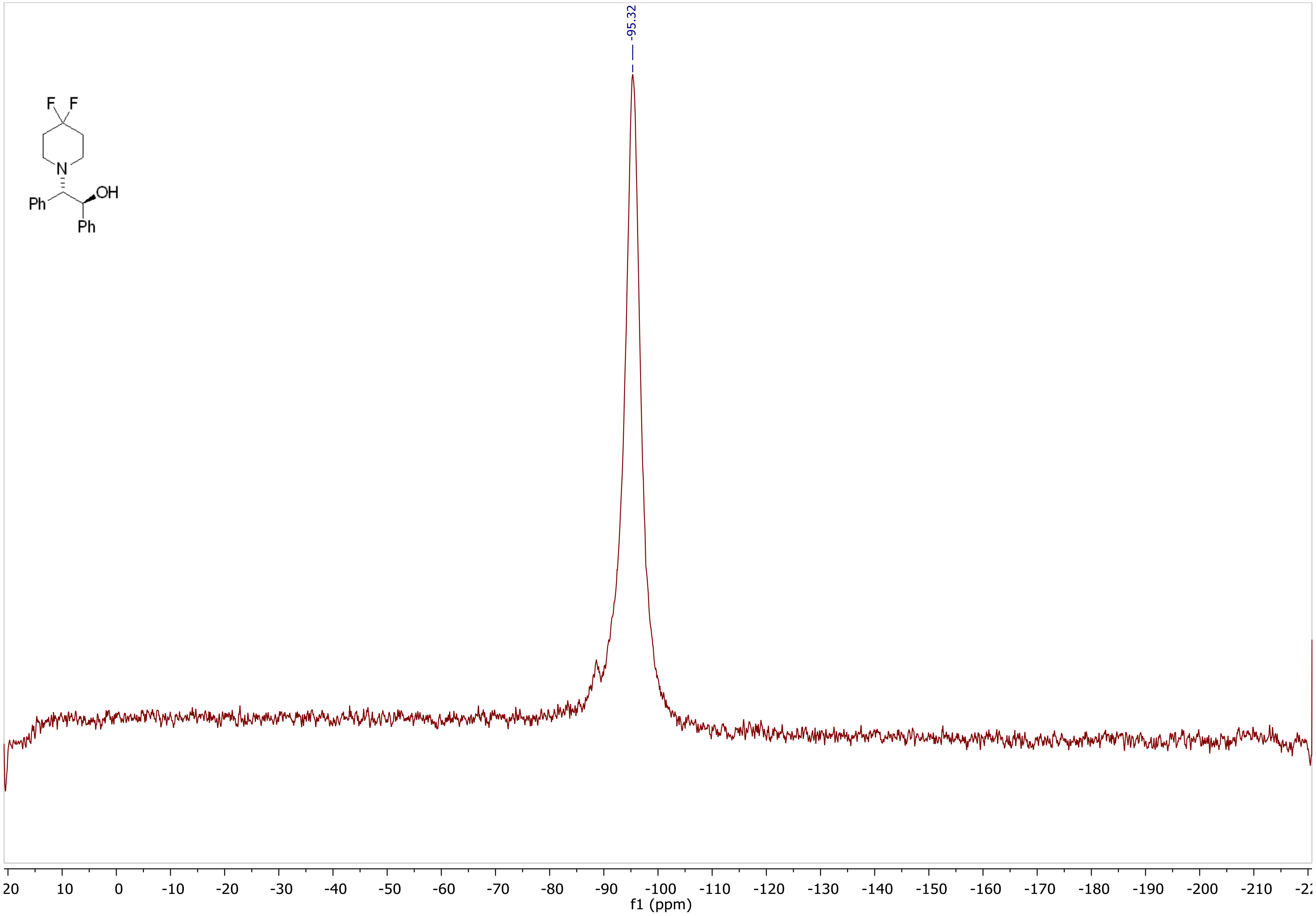

^{13}C NMR (126 MHz, DMSO-d_6)

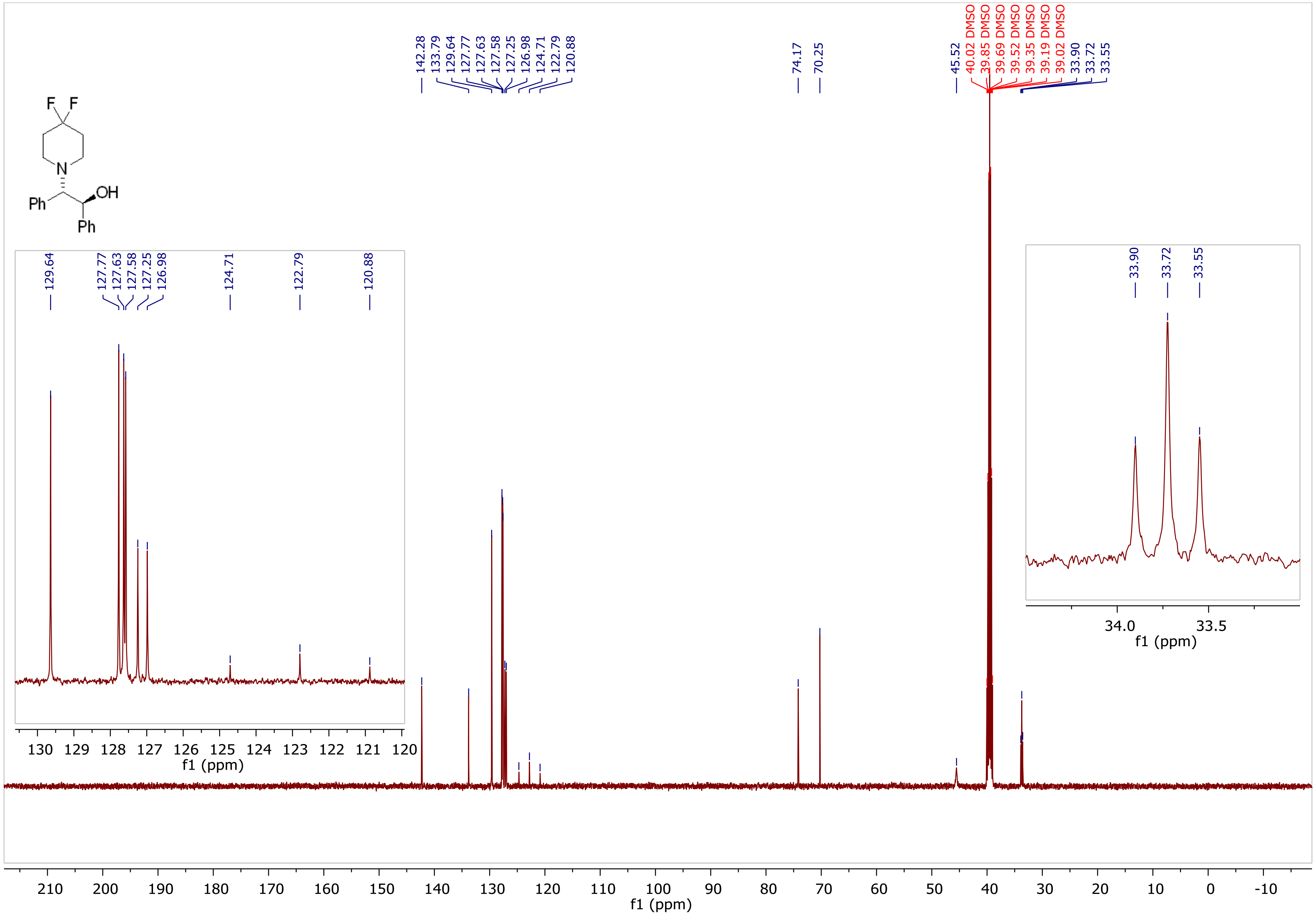

(±)-1-(2-chloro-1,2-diphenlethyl)-4,4-difluoropiperidine (2c)

^{1}H NMR (400 MHz, $CDCl_3$)

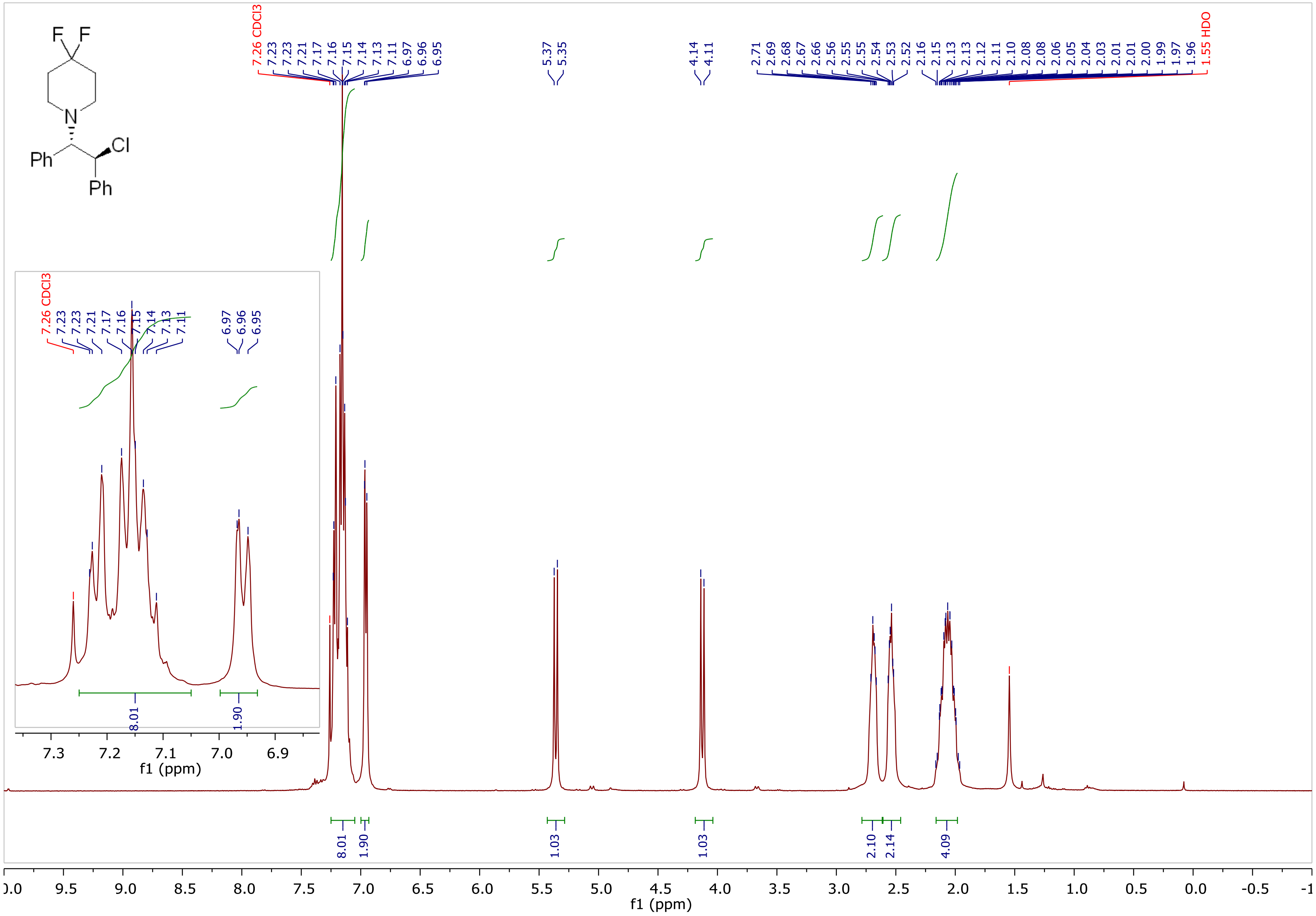

^{19}F NMR (377 MHz, $CDCl_3$)

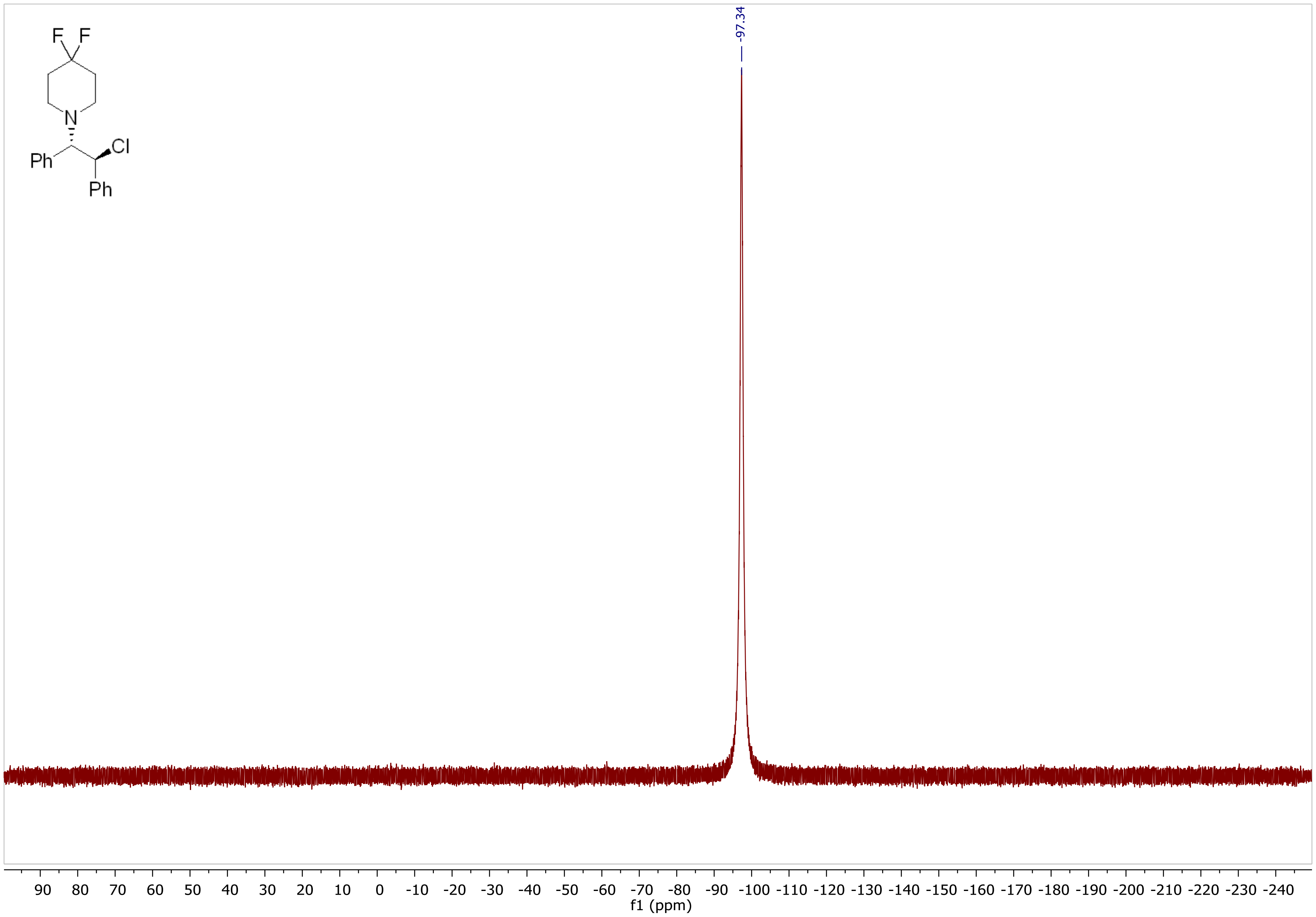

^{13}C NMR (101 MHz, $CDCl_3$)

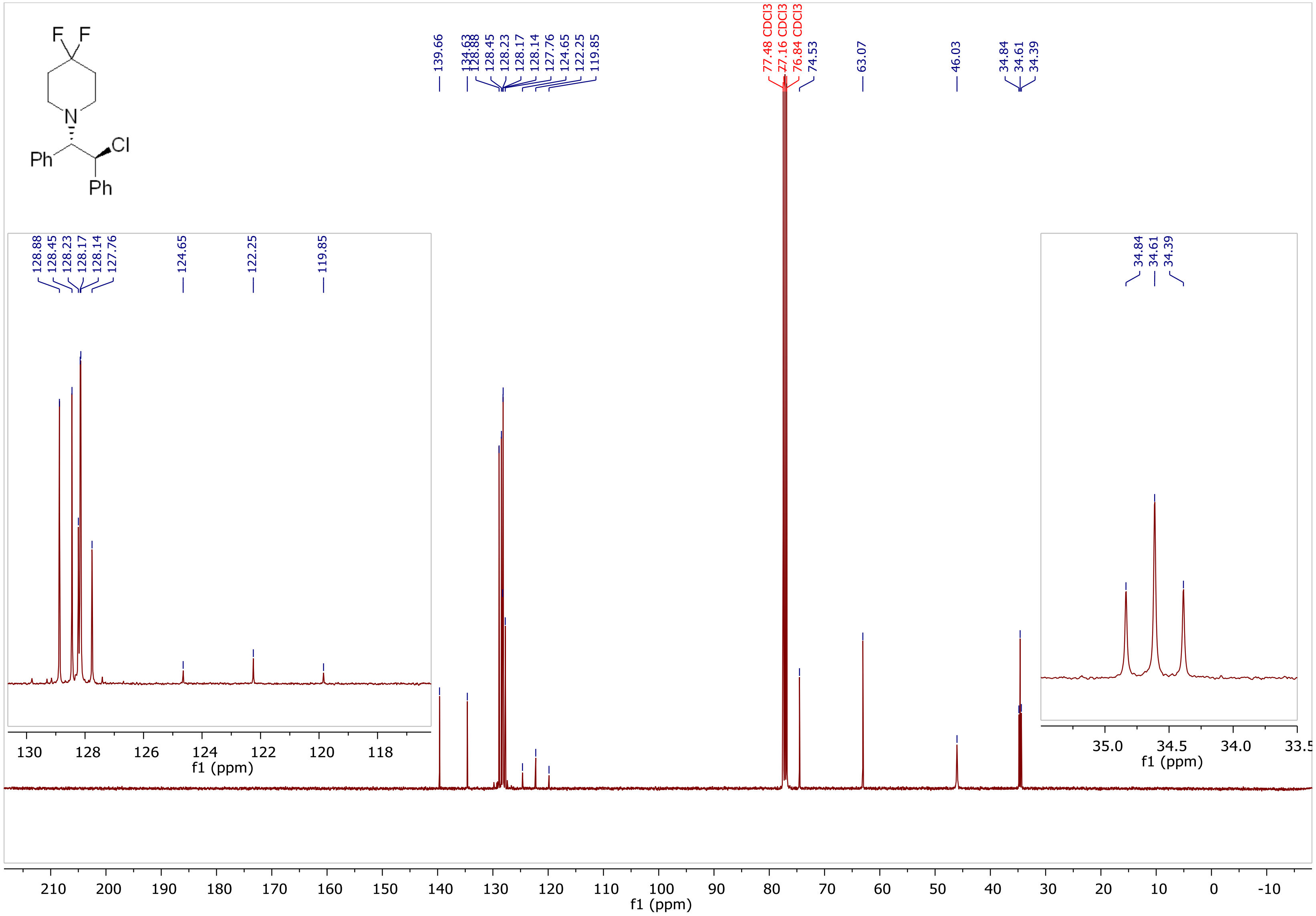

(±)-1,2-diphenyl-2-(4-phenylpiperazin-1-yl)ethan-1-ol

^{1}H NMR (400 MHz, $CDCl_3$)

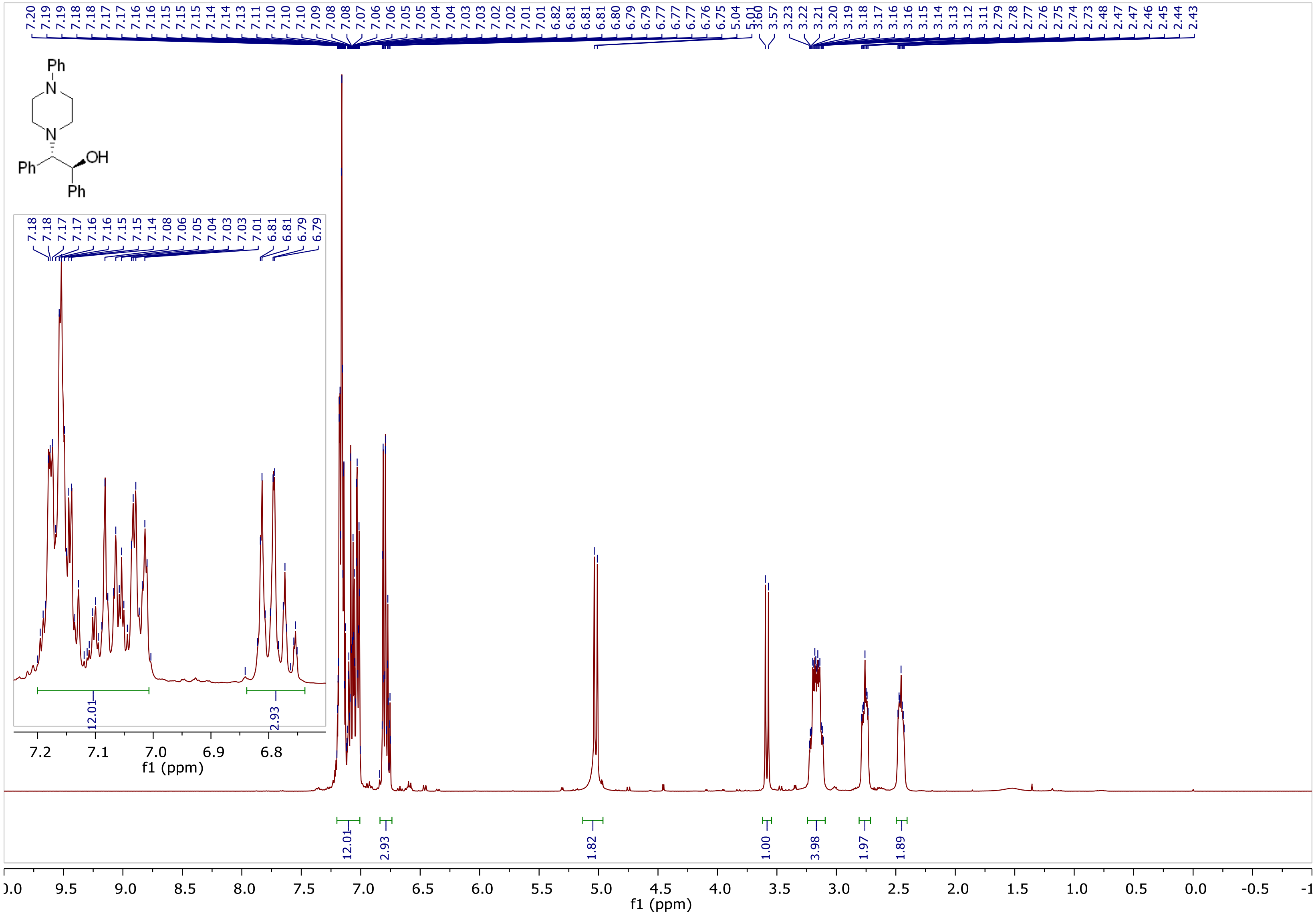

^{13}C NMR (101 MHz, $CDCl_3$)

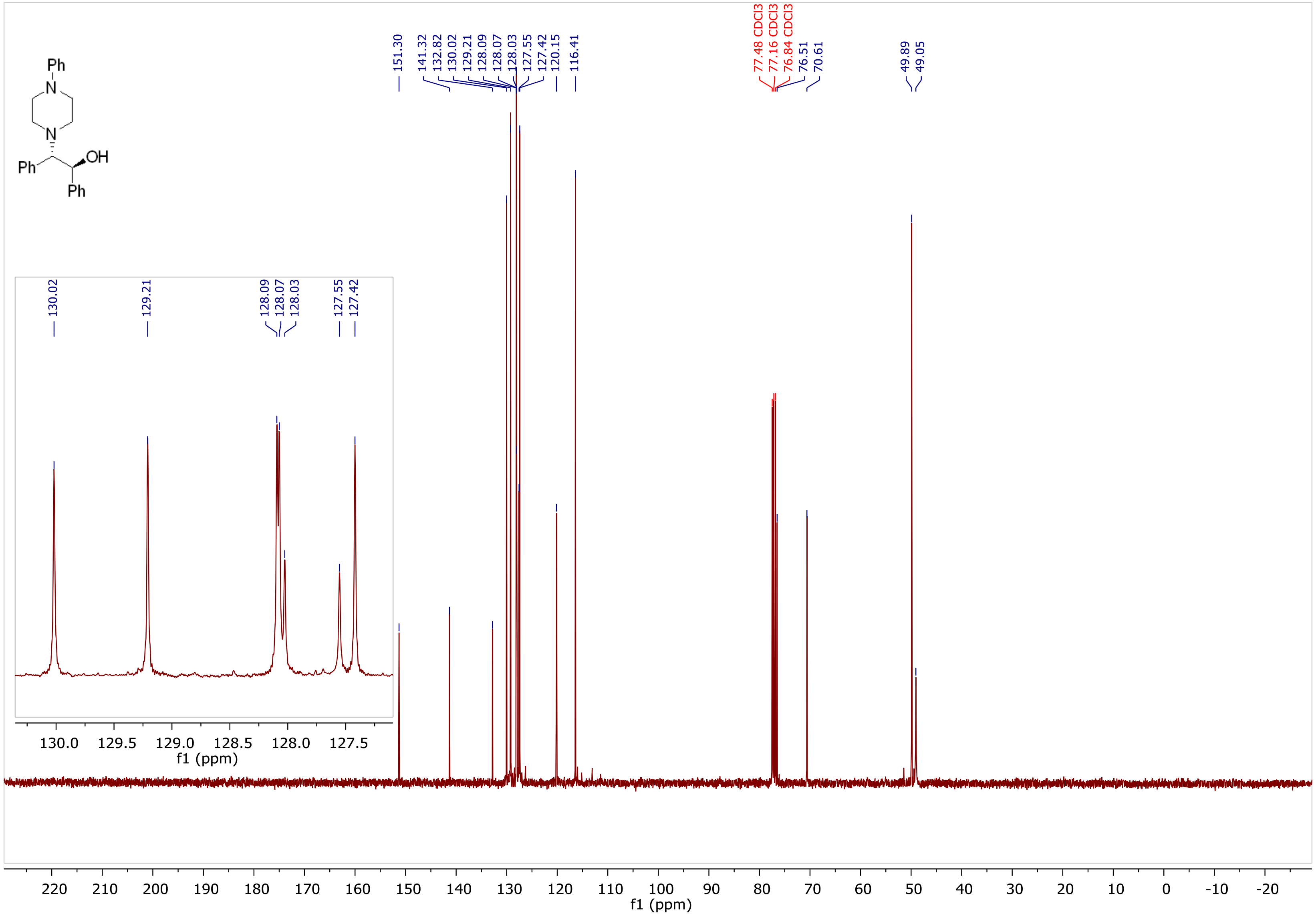

(±)-1-(2-chloro-1,2-diphenylethyl)-4-phenylpiperazine (2g)

^{1}H NMR (400 MHz, $CDCl_3$)

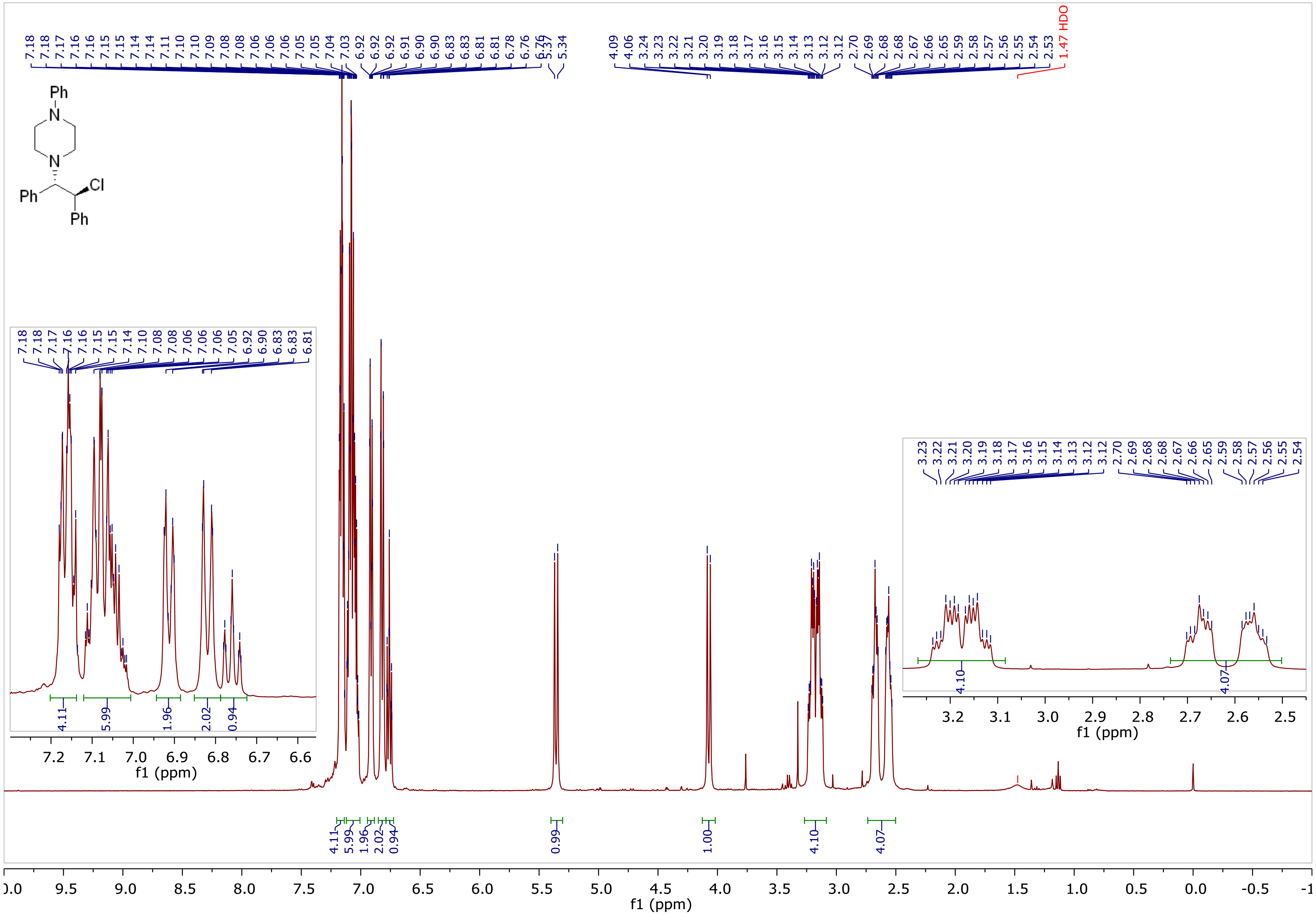

^{13}C NMR (101 MHz, $CDCl_3$)

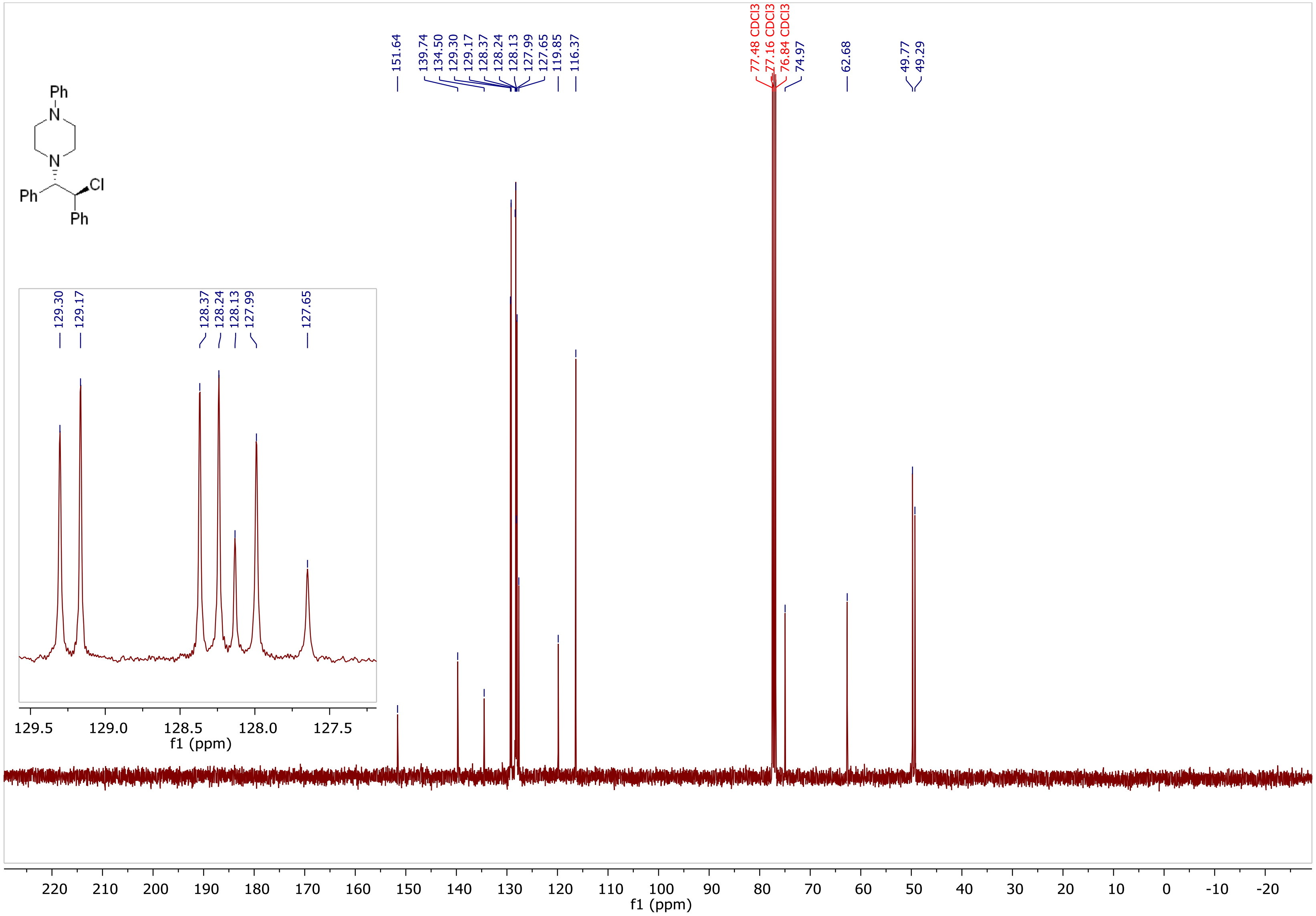

(±)-2-(benzyl(methyl)amino)-1,2-diphenylethan-1-ol

^{1}H NMR (400 MHz, $CDCl_3$)

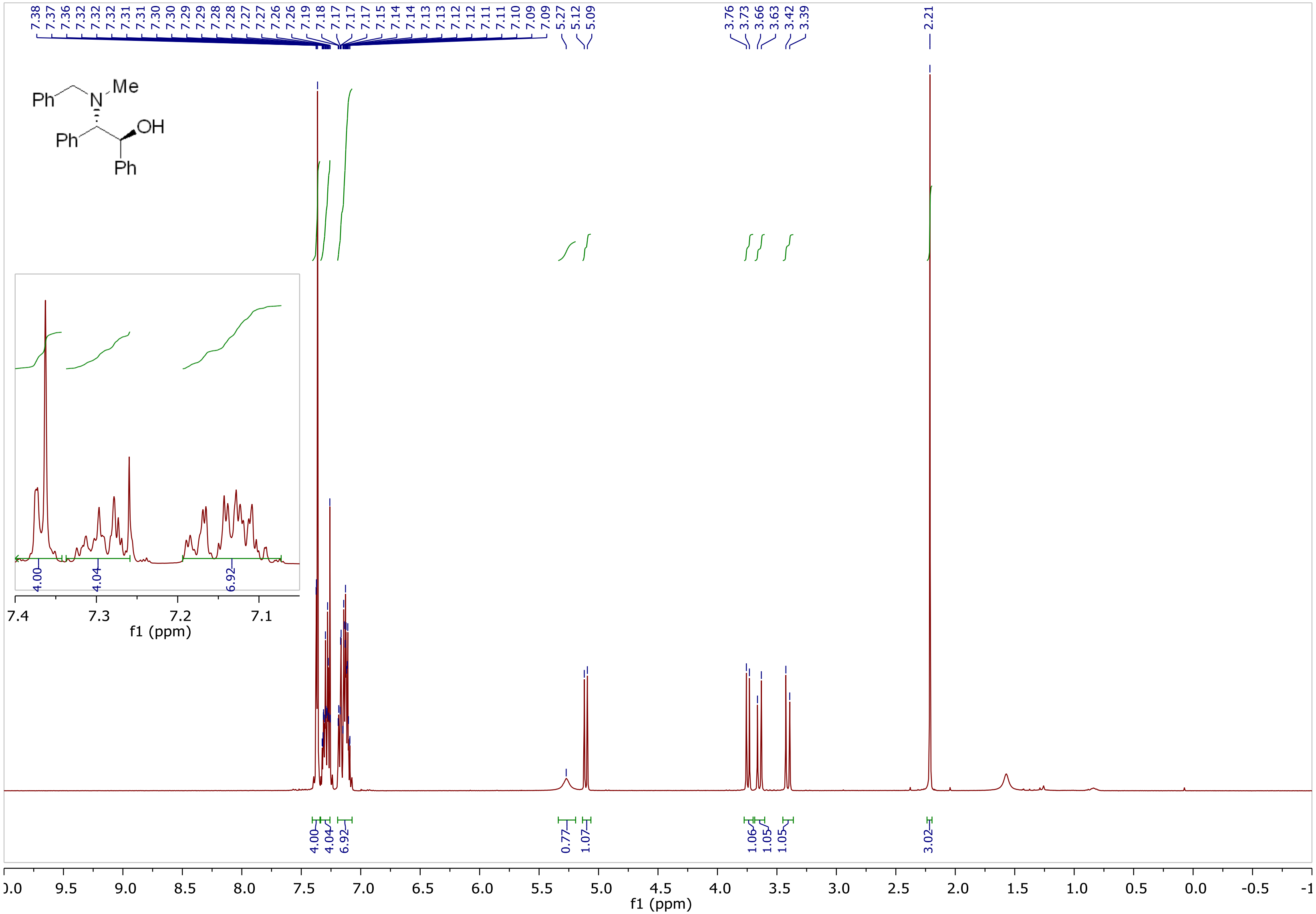

^{13}C NMR (101 MHz, $CDCl_3$)

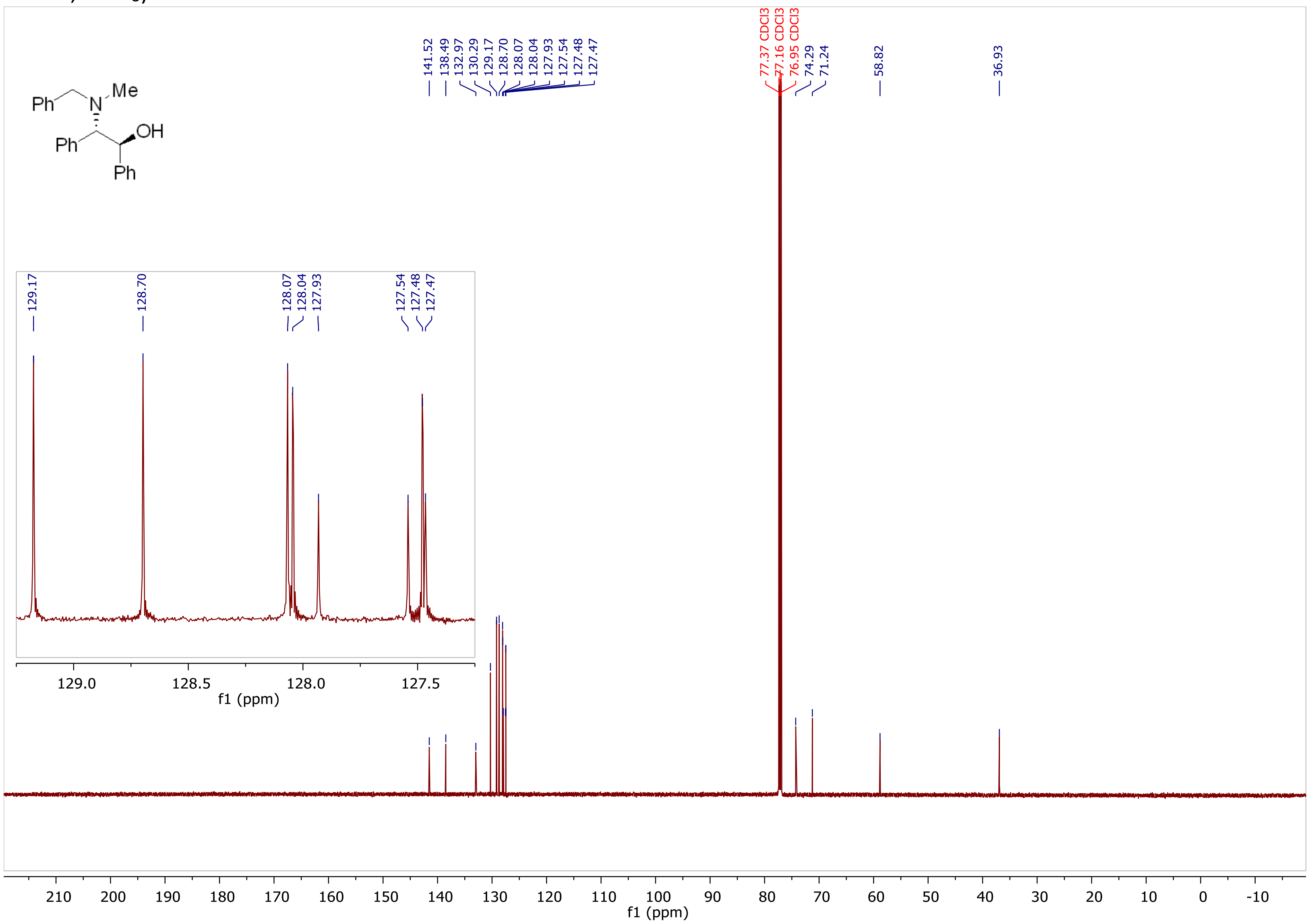

(±)-*N* -benzyl-2-chloro-*N*-methyl-1,2-diphenylethan-1-amine (2i)

^{1}H NMR (400 MHz, $CDCl_3$)

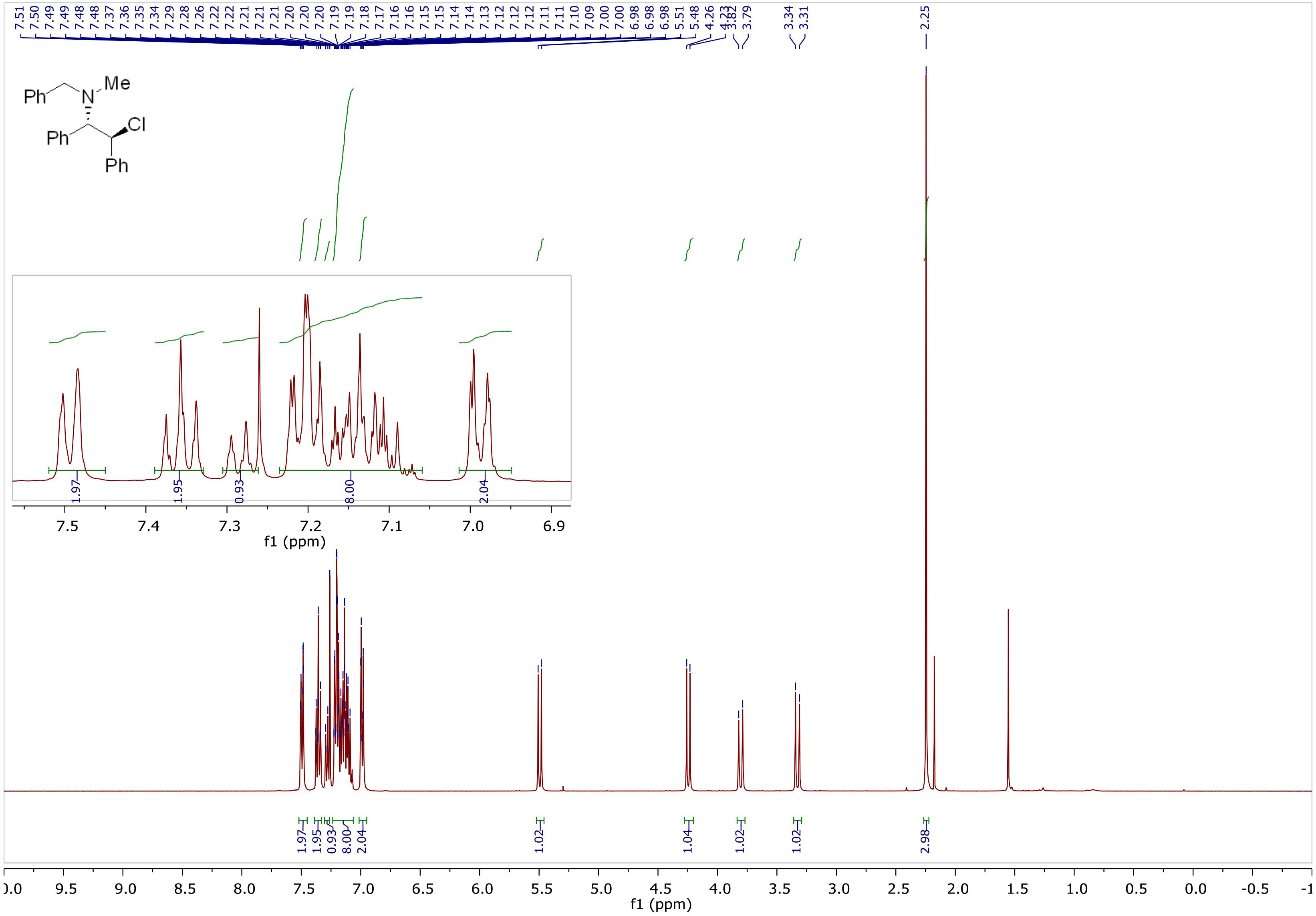

^{13}C NMR (101 MHz, $CDCl_3$)

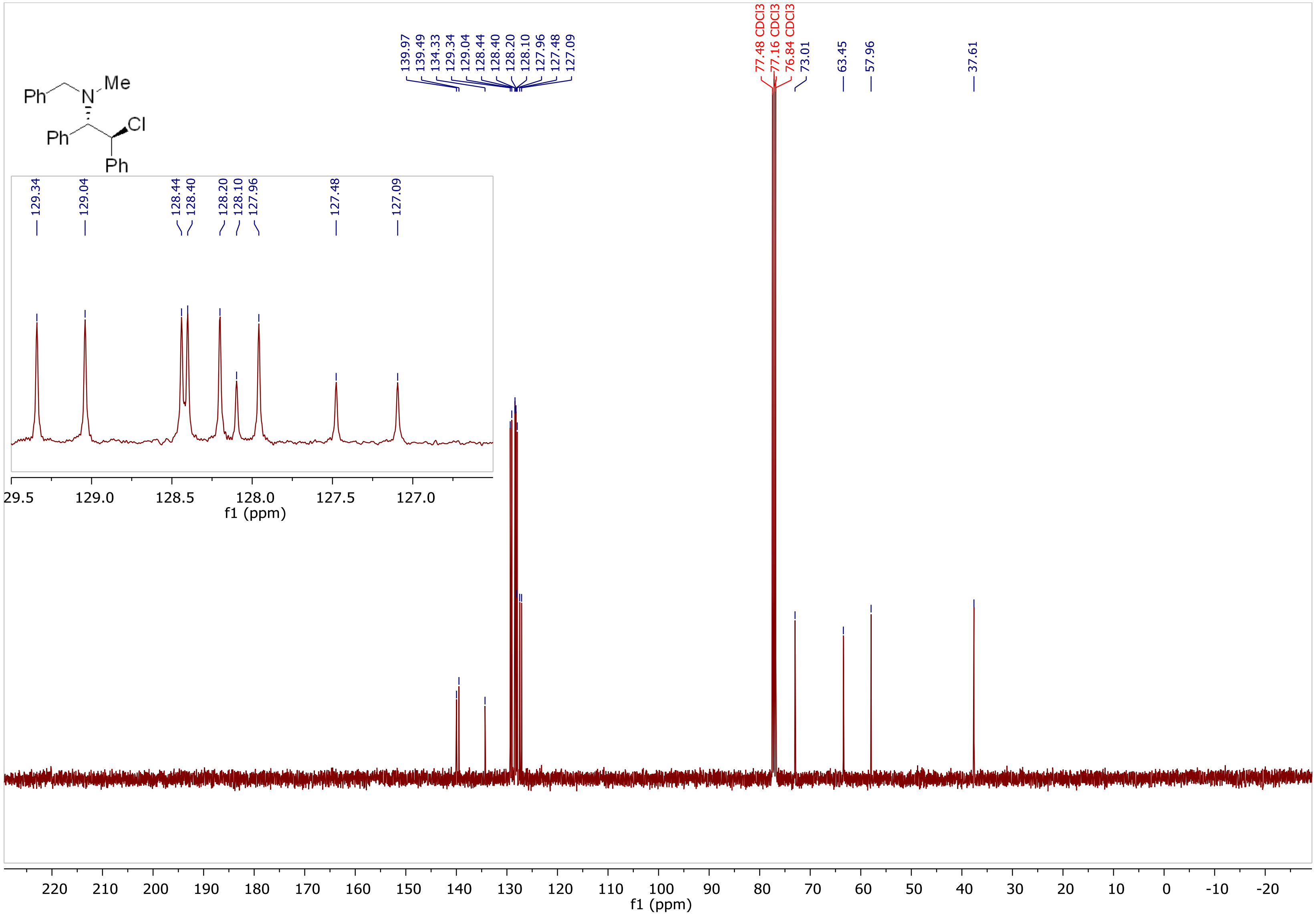

(±)-2-(azepan-1-yl)-1,2-diphenylethan-1-ol

^{1}H NMR (400 MHz, $CDCl_3$)

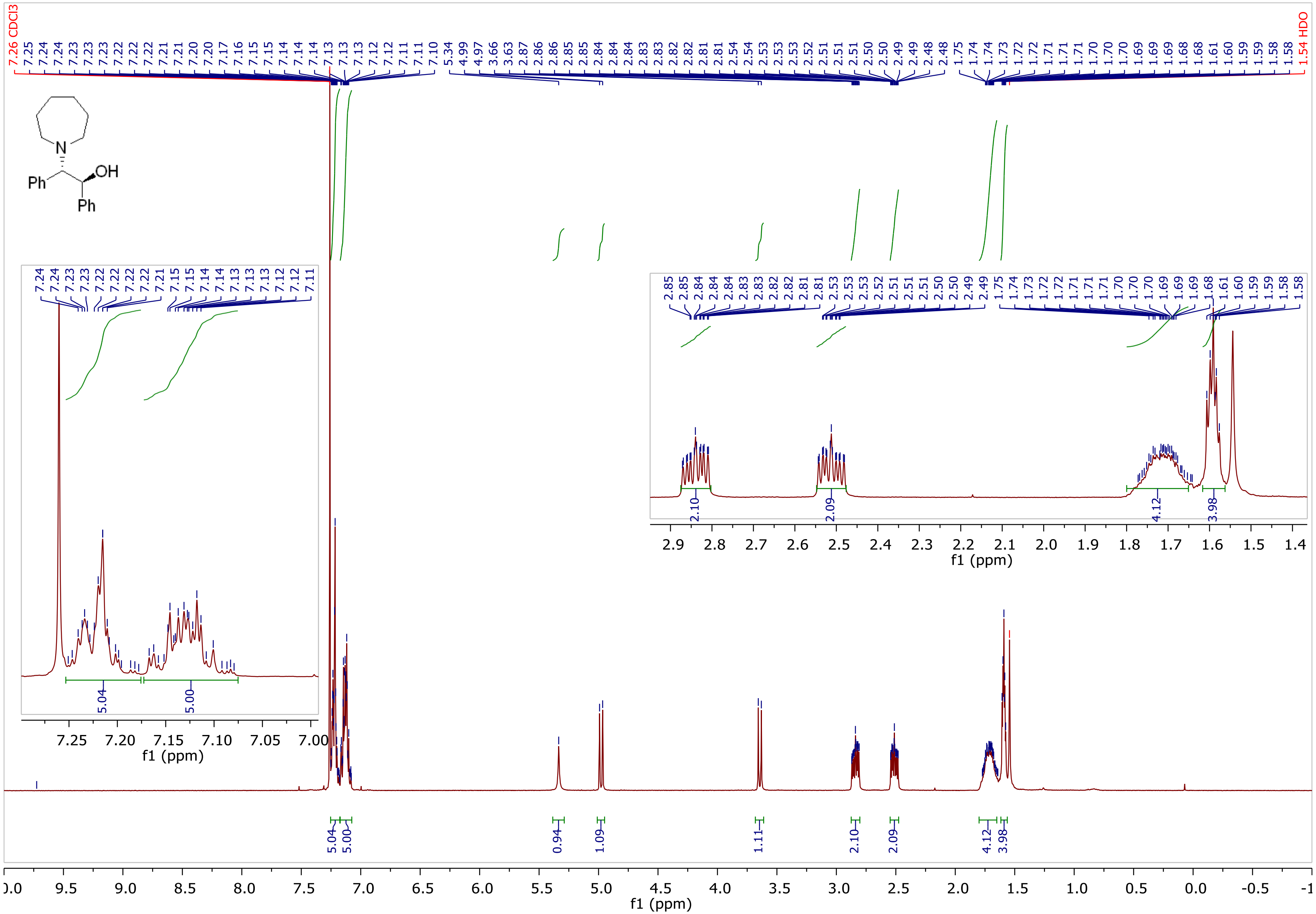

^{13}C NMR (101 MHz, $CDCl_3$)

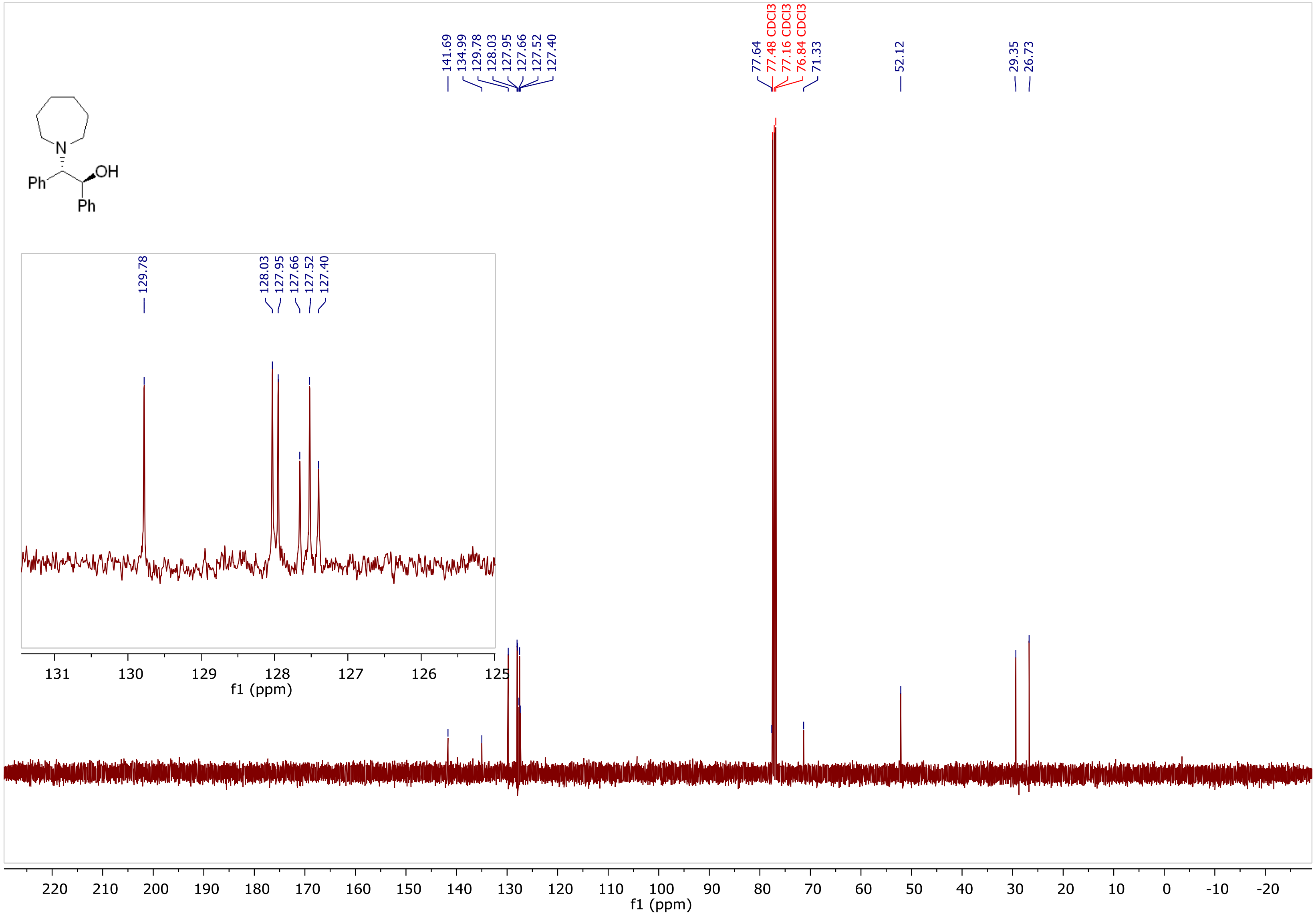

(±)-2-chloro-1,2-diphenylethyl)azepane (2k)

^{1}H NMR (400 MHz, $CDCl_3$)

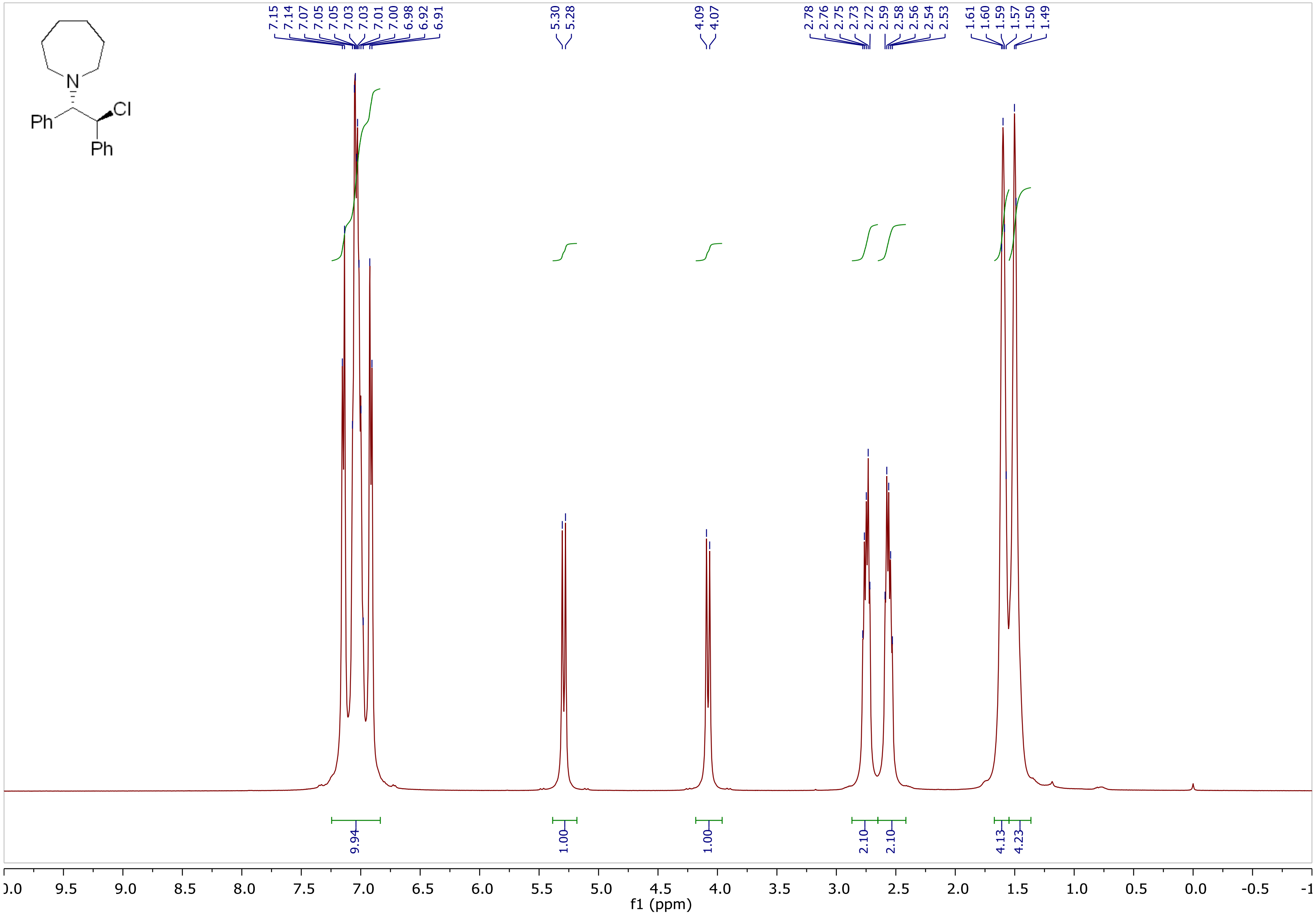

^{13}C NMR (101 MHz, $CDCl_3$)

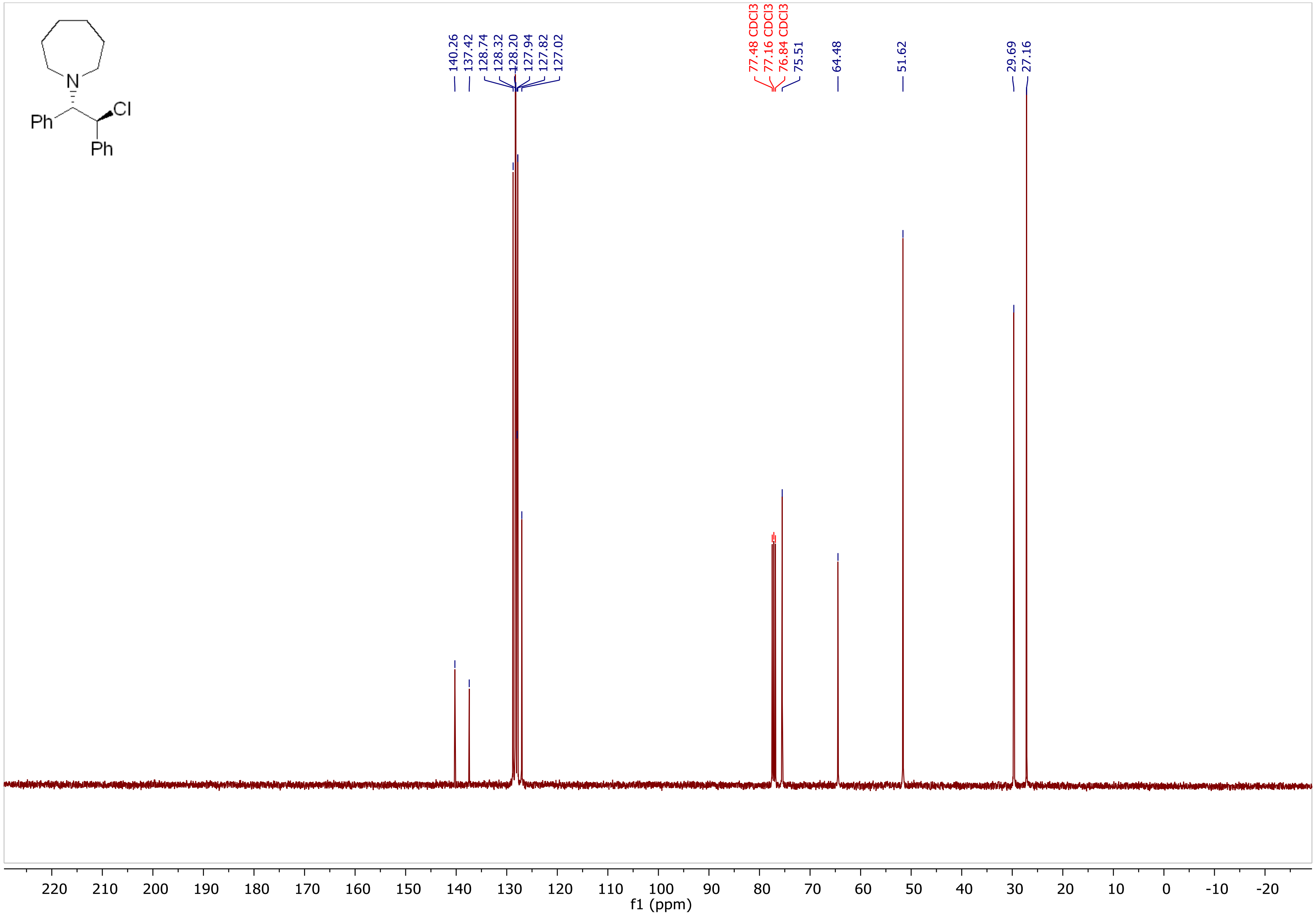

(±)-1,2-bis(3-fluorophenyl)-2-(piperidin-1-yl)ethan-1-ol

^{1}H NMR (500 MHz, $CDCl_3$)

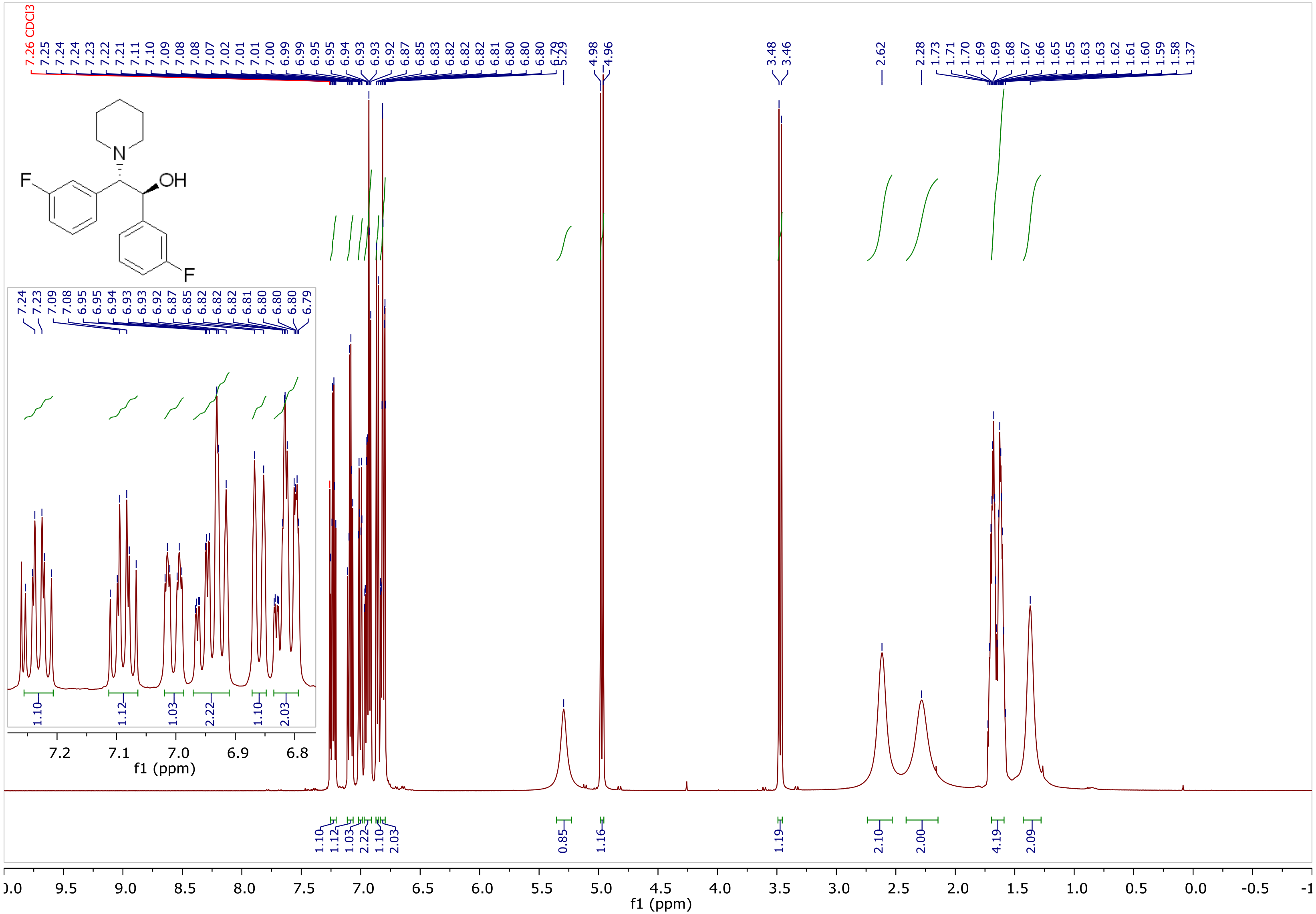

^{19}F NMR (471 MHz, $CDCl_3$)

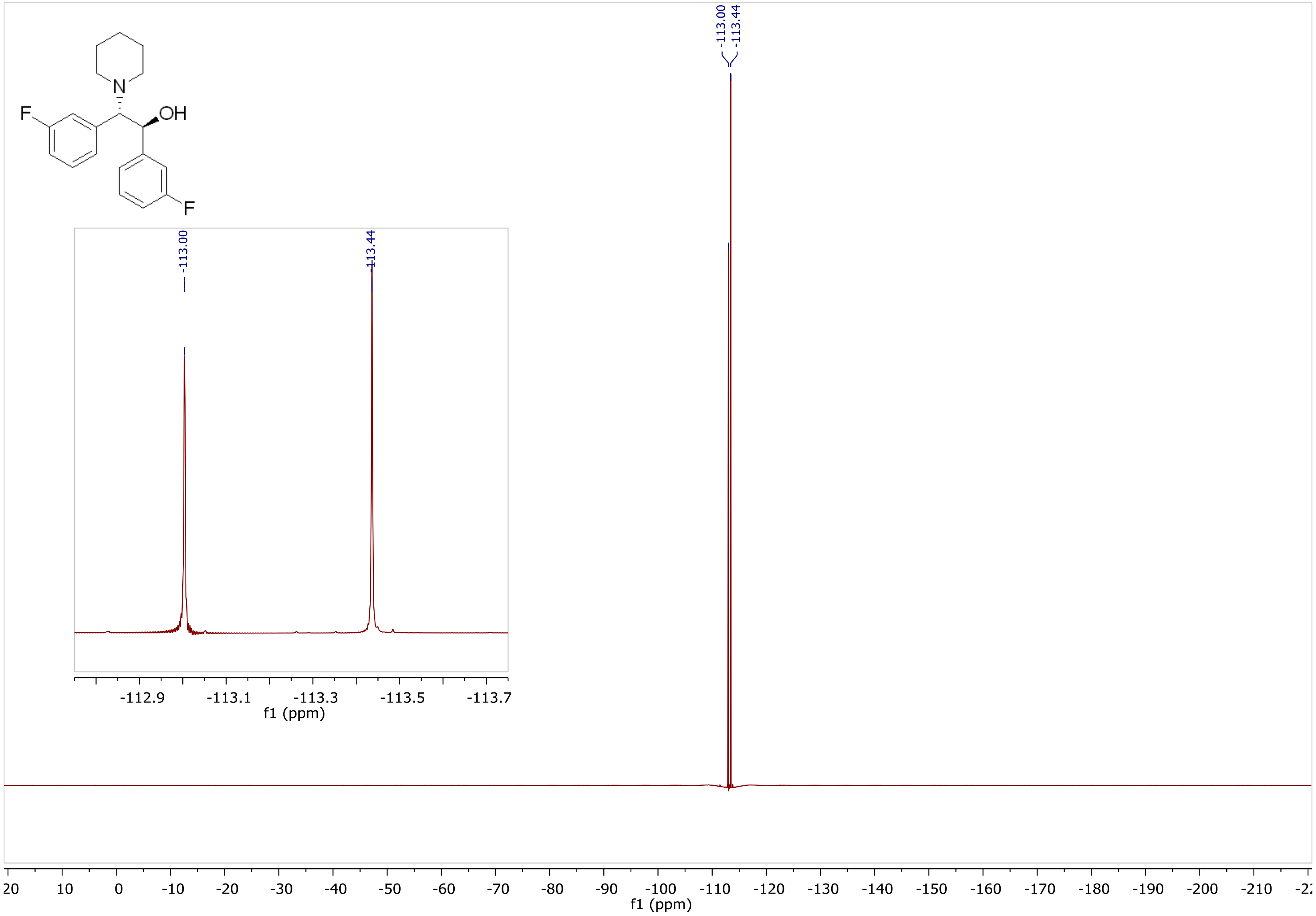

^{13}C NMR (126 MHz, $CDCl_3$)

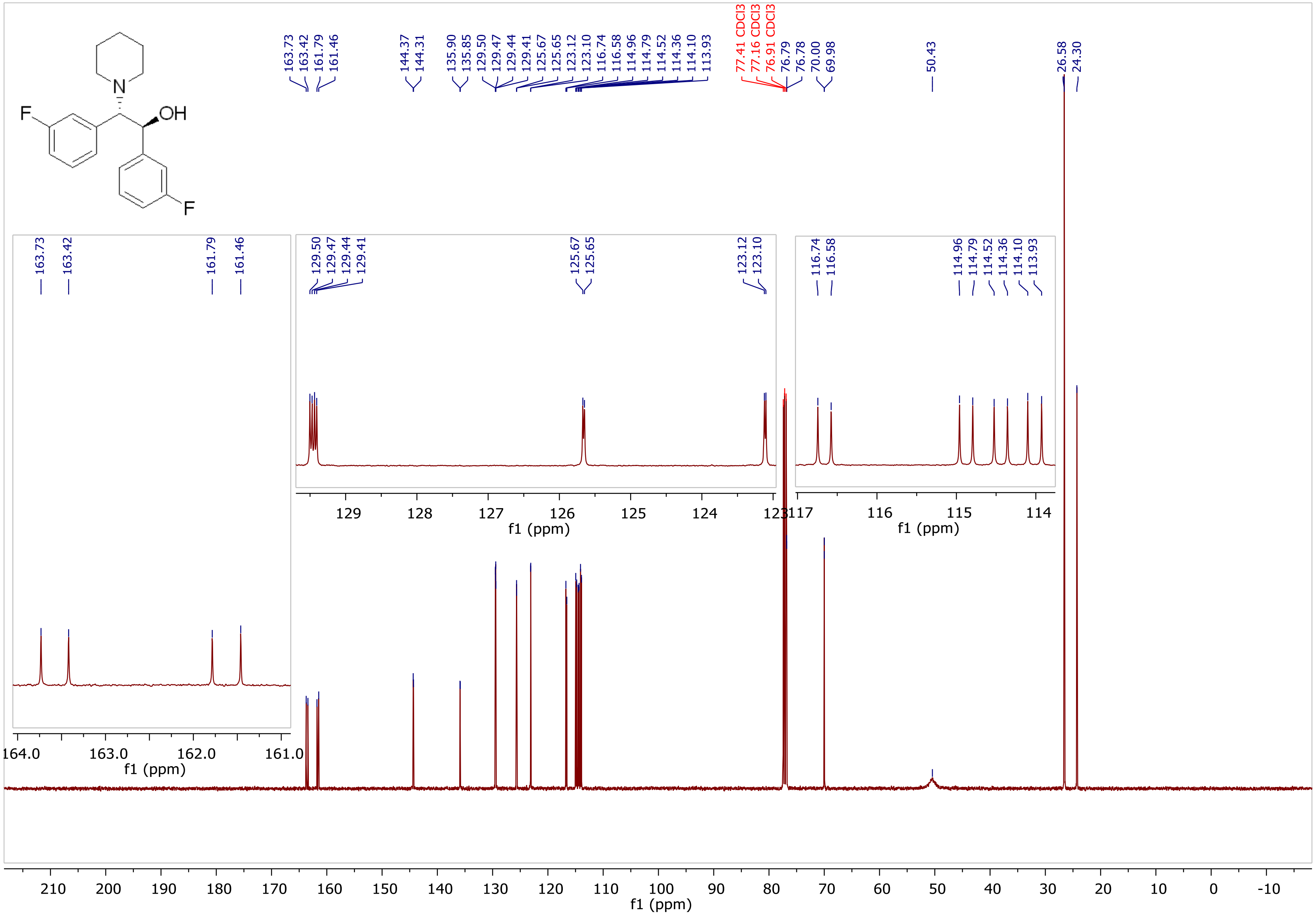

(±)-1-(2-chloro-1,2-bis(3-fluorophenyl)ethyl)piperidine (2l)

^{1}H NMR (500 MHz, CDCl$_3$)

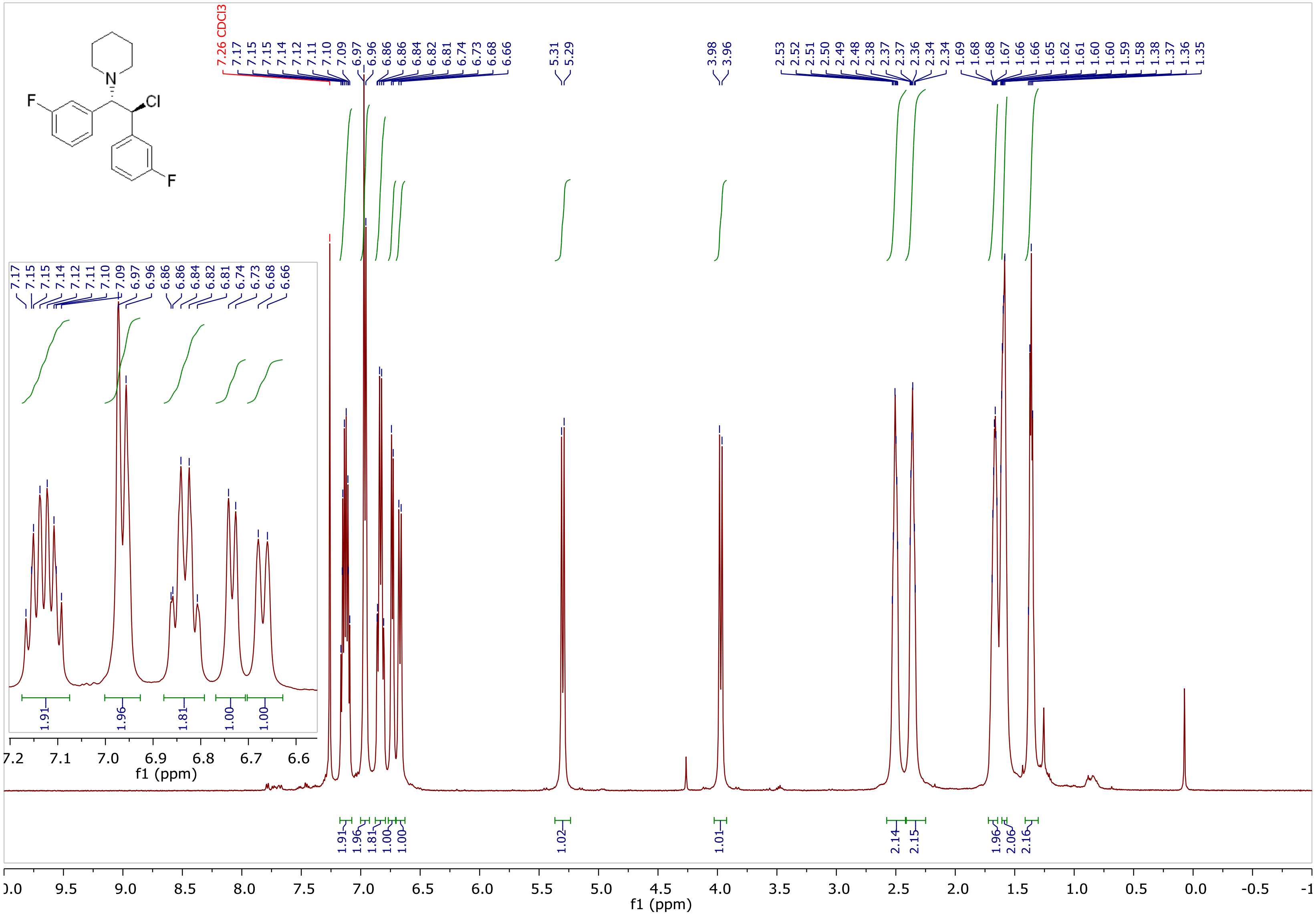

^{19}F NMR (471 MHz, $CDCl_3$)

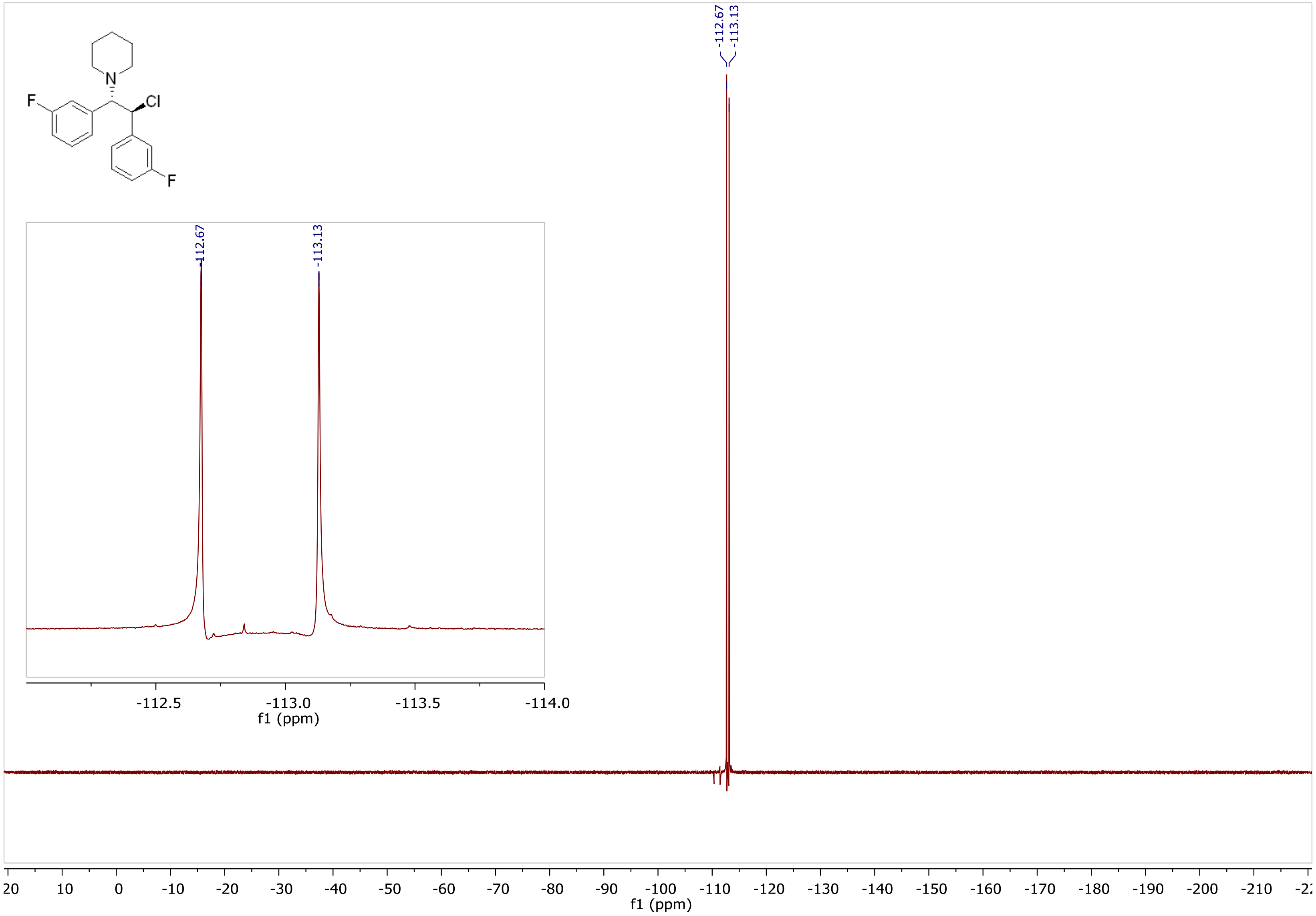

^{13}C NMR (126 MHz, $CDCl_3$)

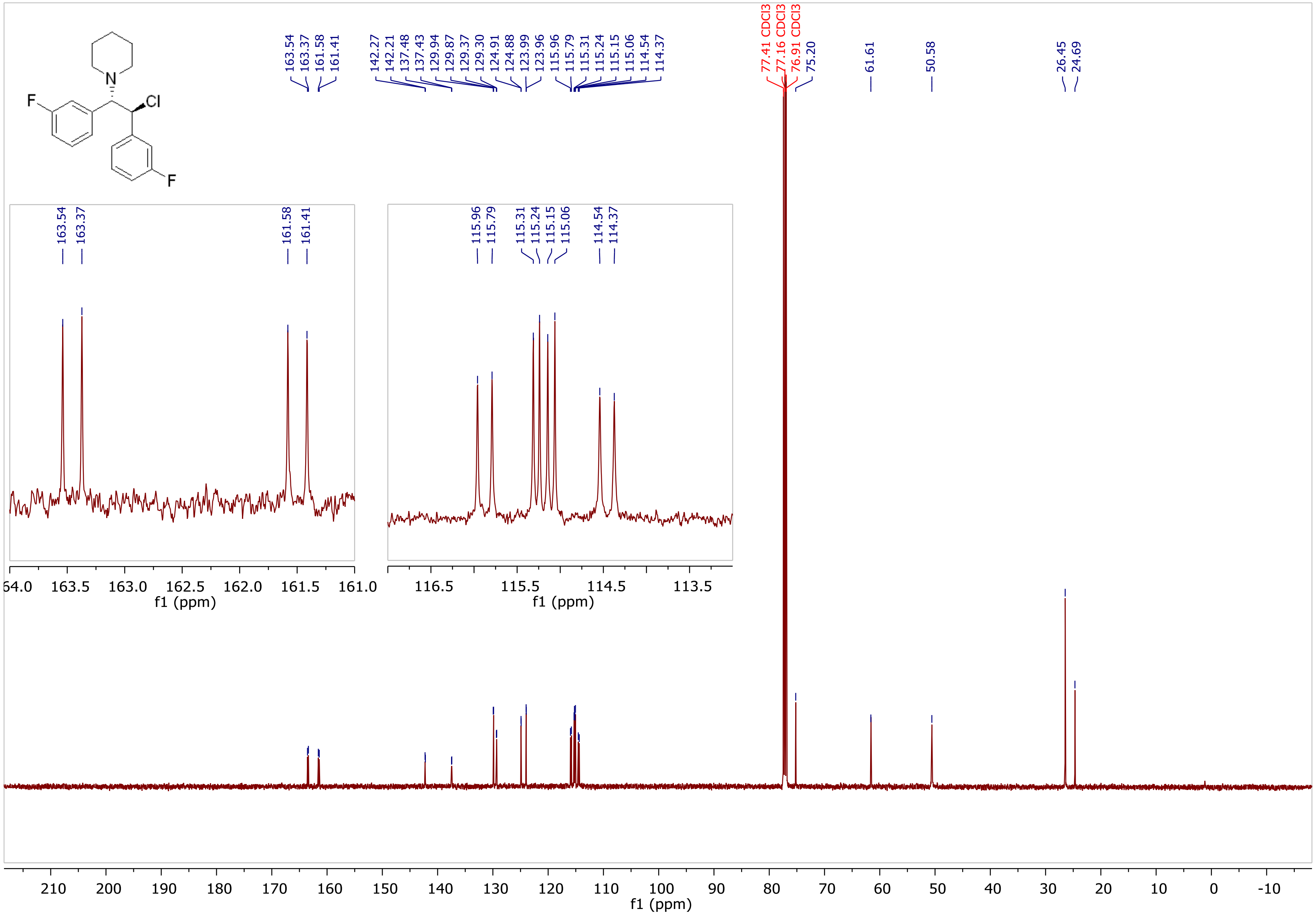

(±)-1,2-bis(3-chlorophenyl)-2-(piperidin-1-yl)ethan-1-ol

^{1}H NMR (400 MHz, $CDCl_3$)

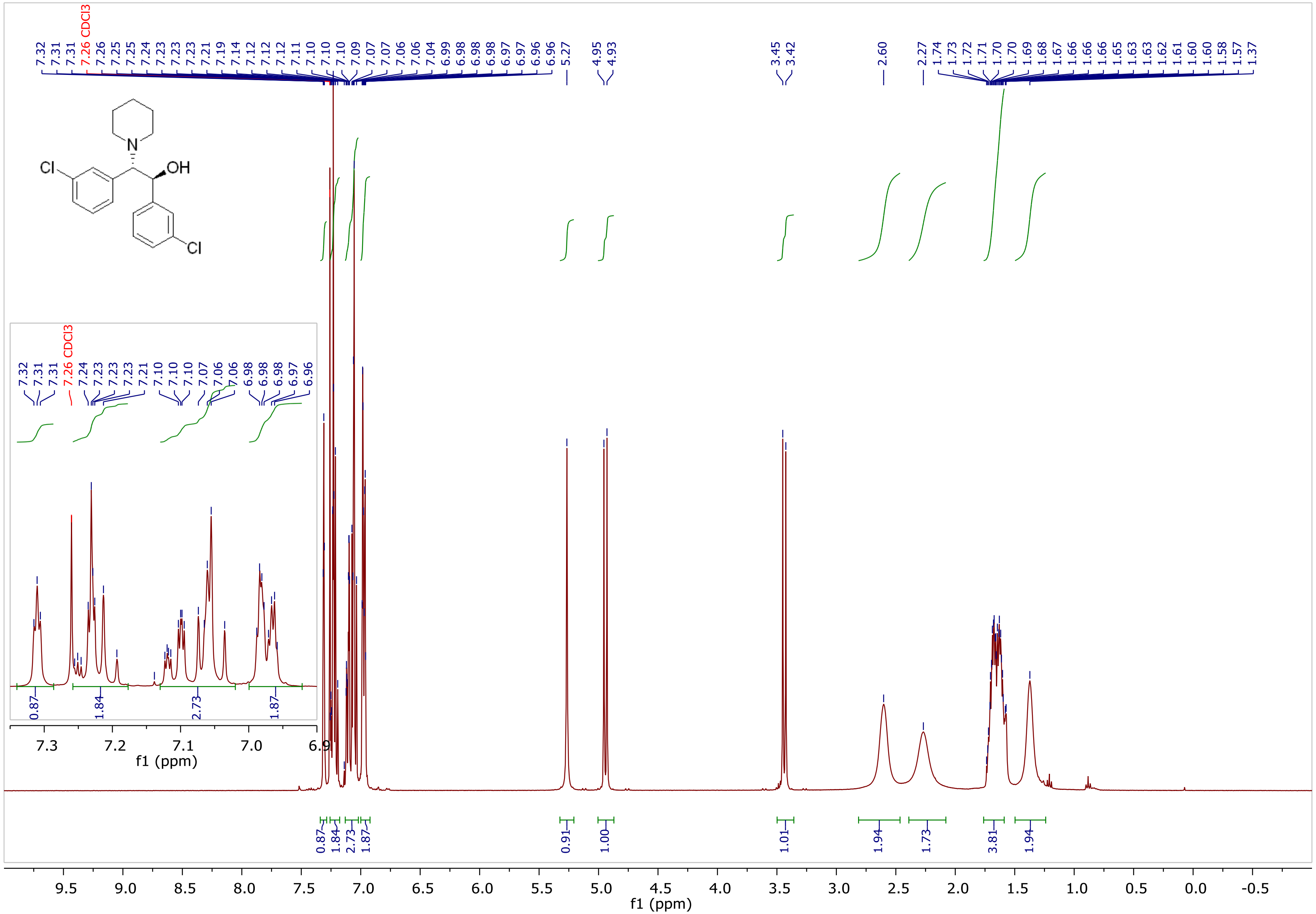

^{13}C NMR (101 MHz, $CDCl_3$)

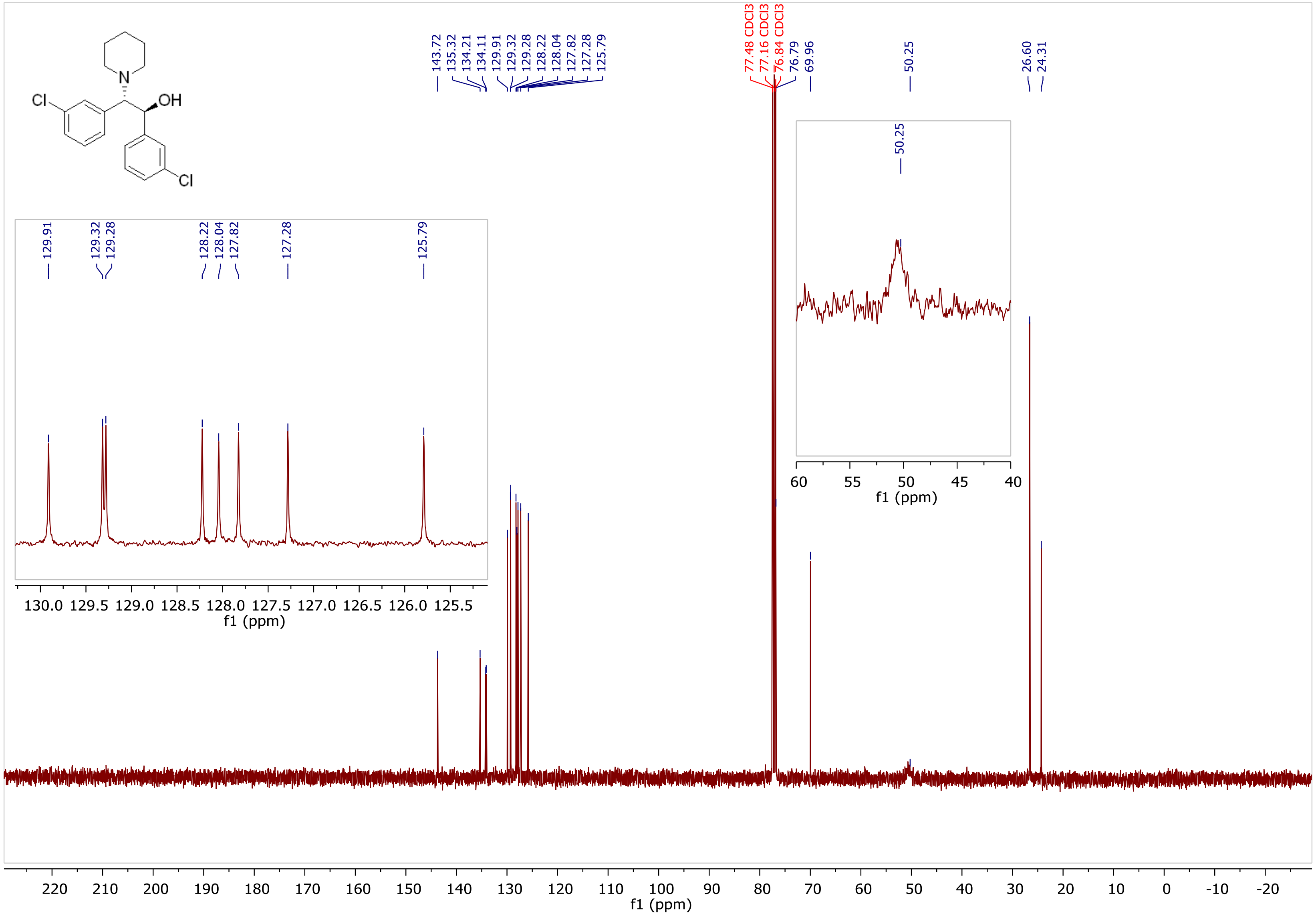

(±)-1-(2-chloro-1,2-bis(3-chlorophenyl)ethyl)piperidine (2m)

¹H NMR (400 MHz, $CDCl_3$)

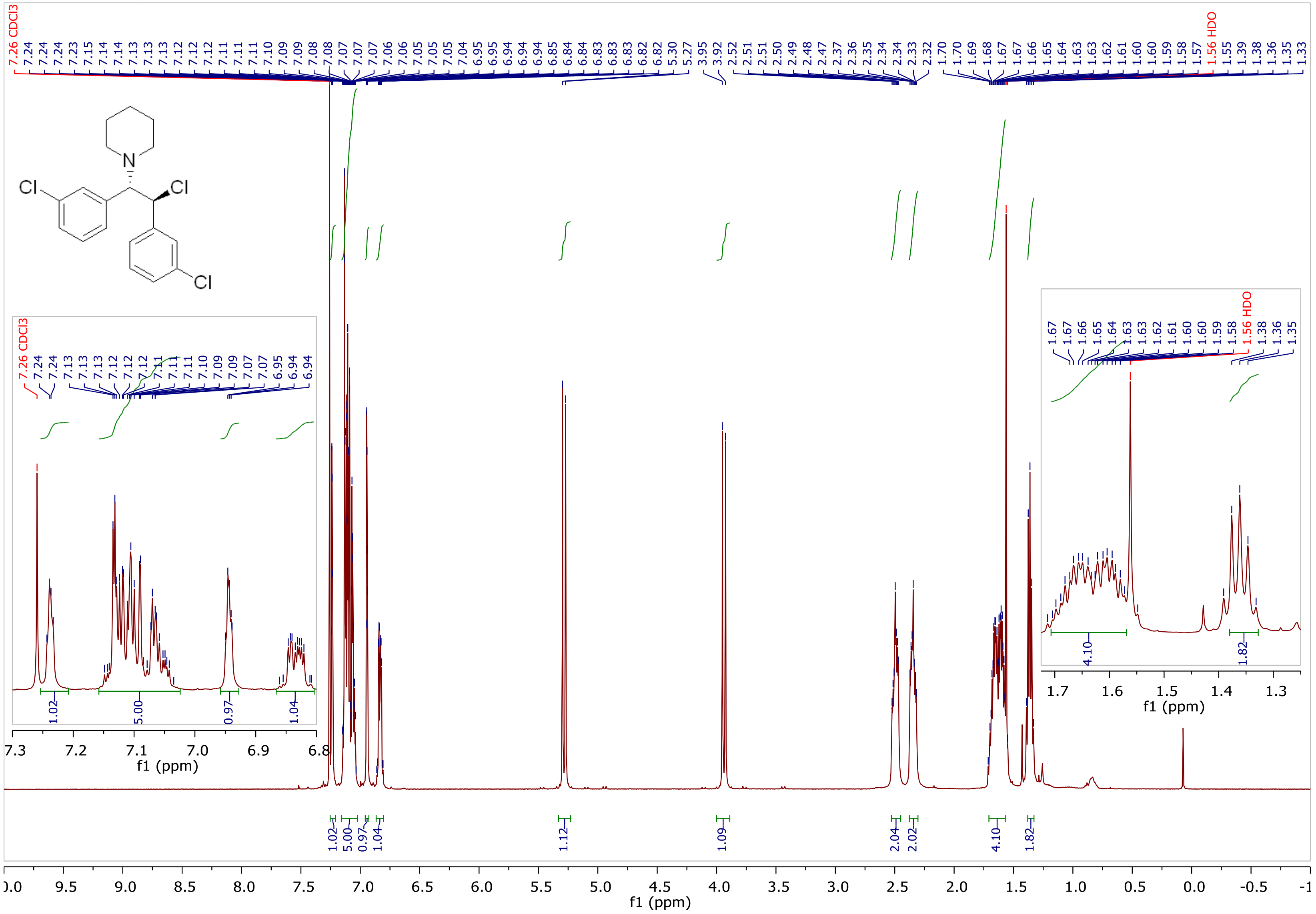

^{13}C NMR (101 MHz, $CDCl_3$)

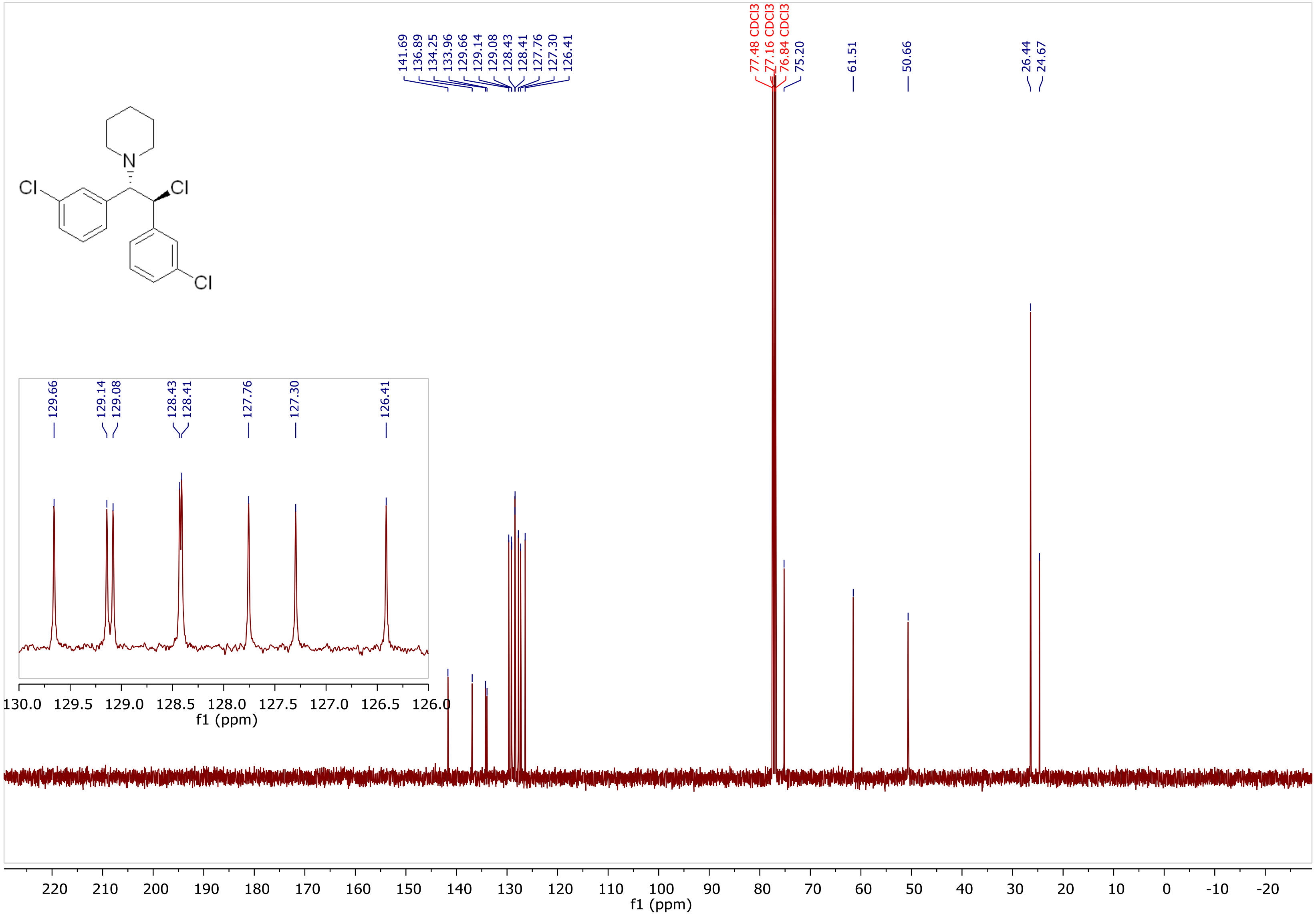

(±)-2-(piperidin-1-yl)-1,2-bis(3-(trifluoromethyl)phenyl)ethan-1-ol

^{1}H NMR (400 MHz, $CDCl_3$)

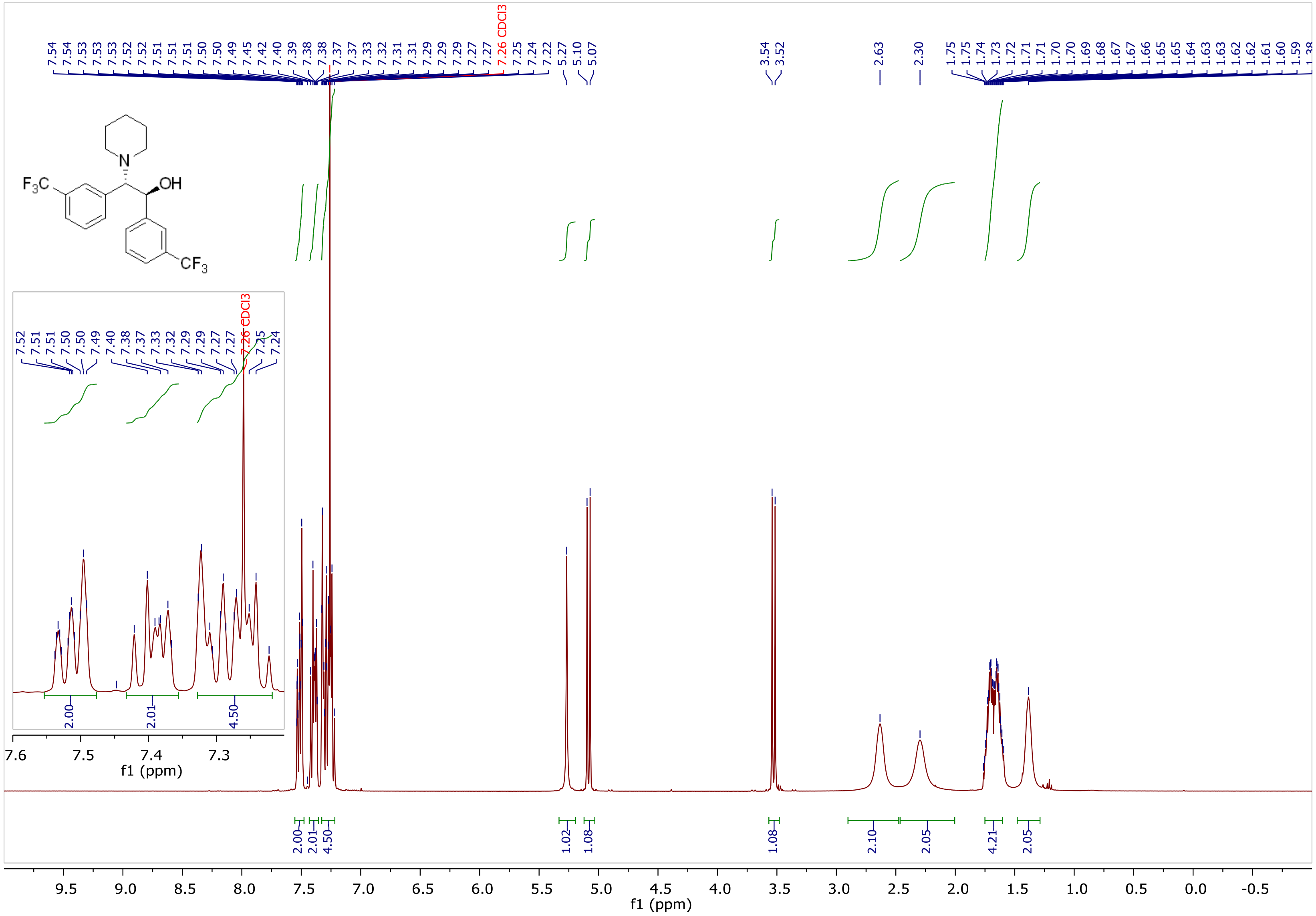

^{19}F NMR (377 MHz, $CDCl_3$)

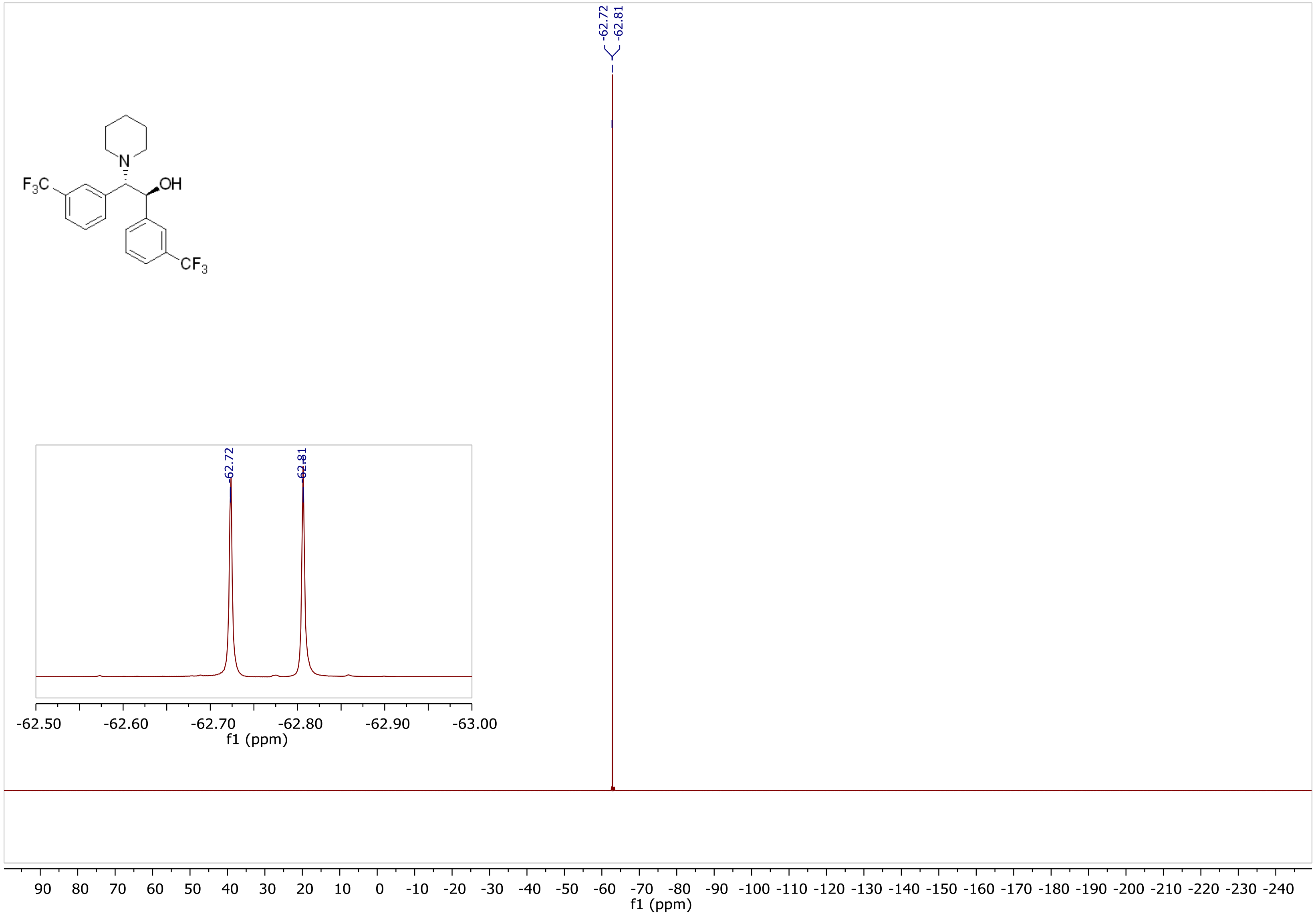

^{13}C NMR (101 MHz, $CDCl_3$)

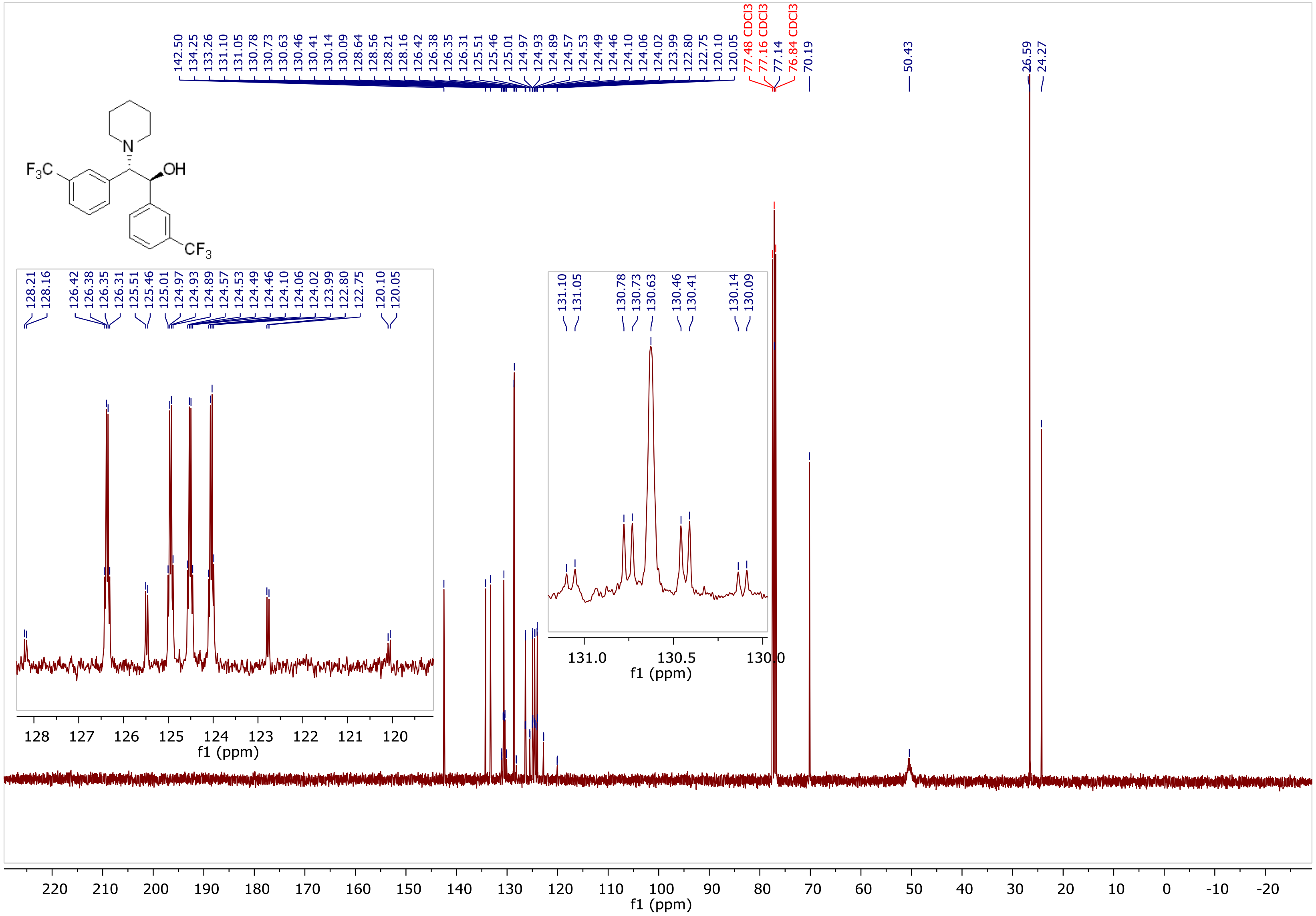

(±)-1-(2-chloro-1,2-bis(3-(trifluoromethyl)phenyl)ethyl)piperidine (2n)

^{1}H NMR (500 MHz, $CDCl_3$)

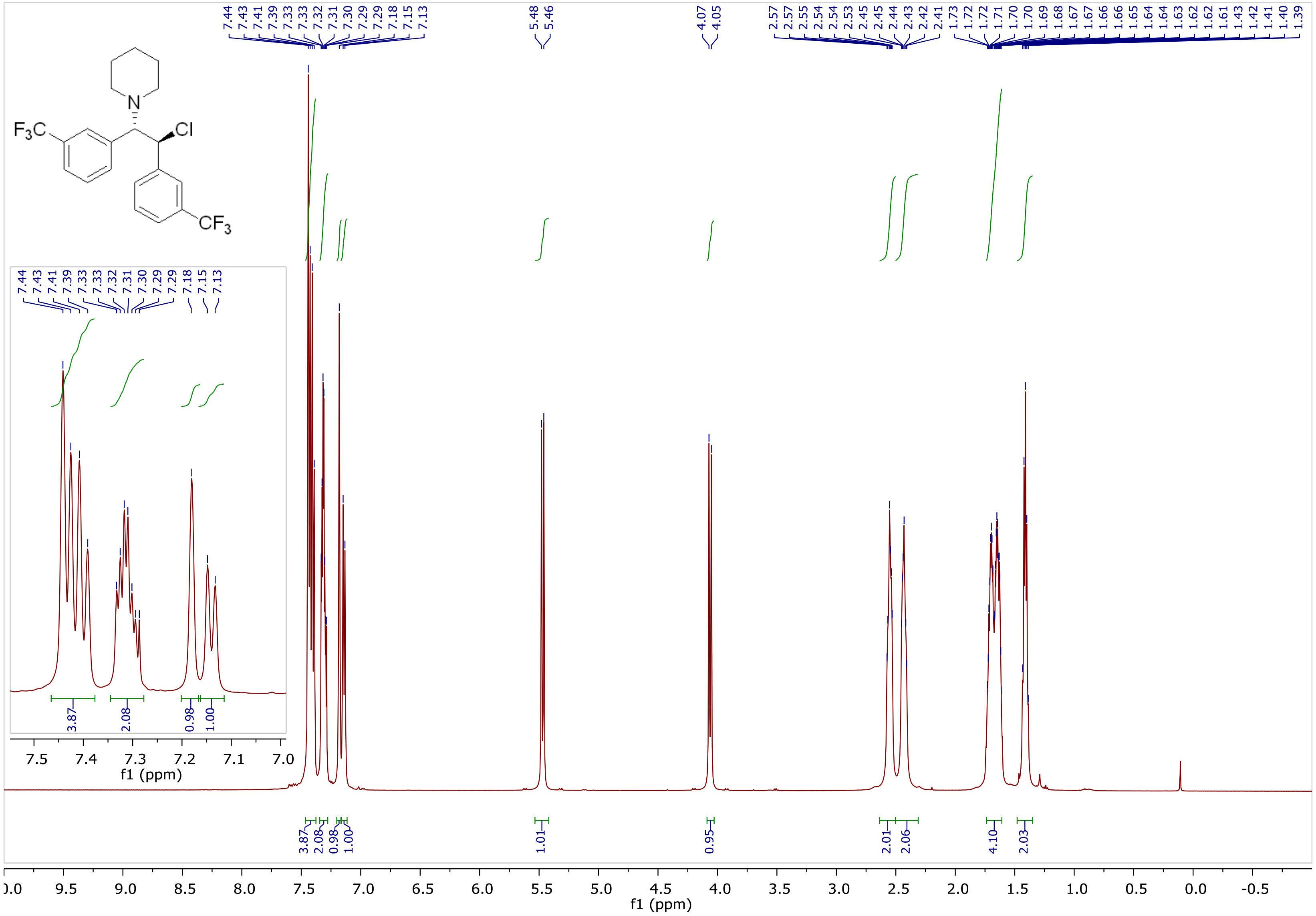

^{19}F NMR (471 MHz, $CDCl_3$)

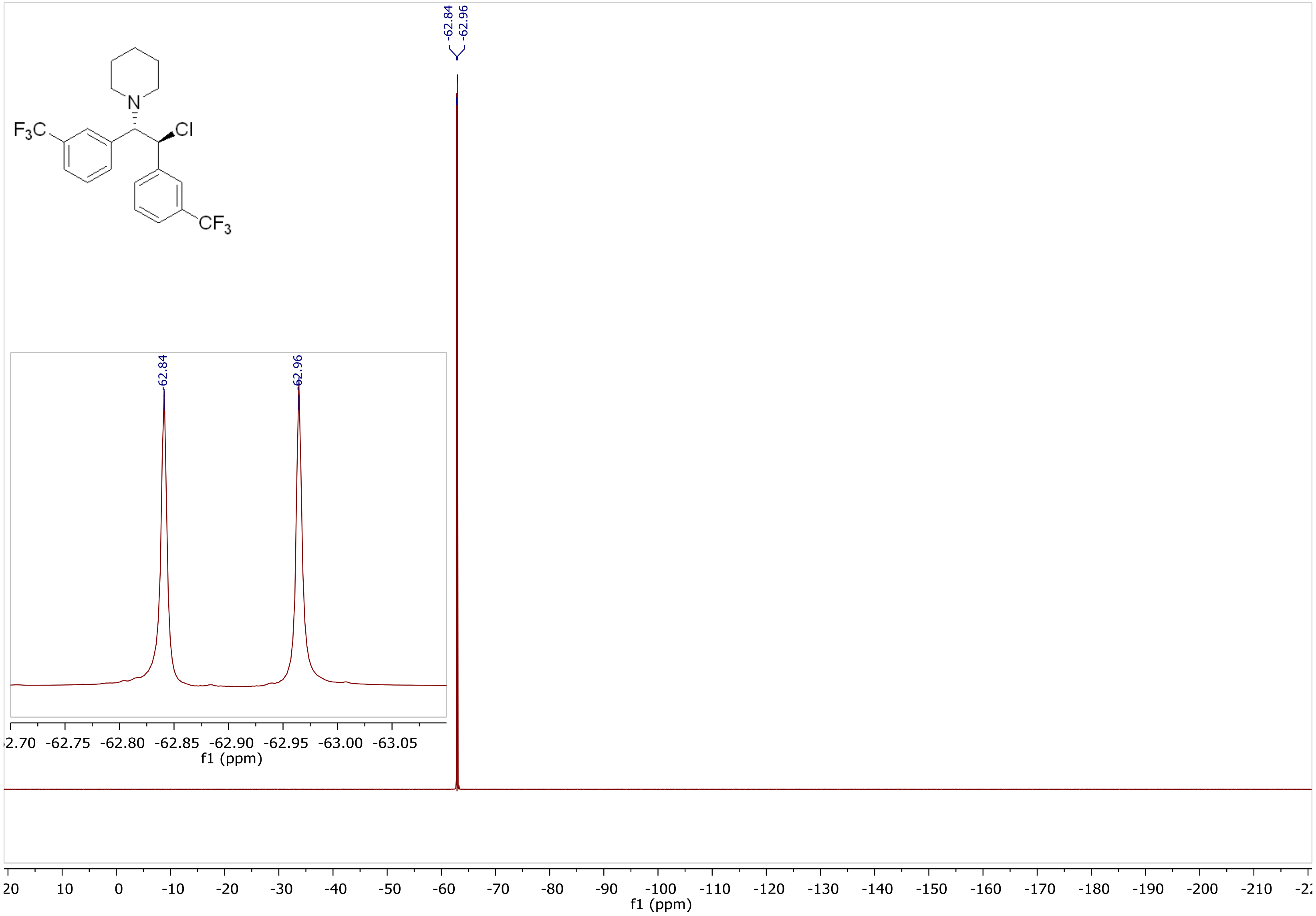

^{13}C NMR (126 MHz, $CDCl_3$)

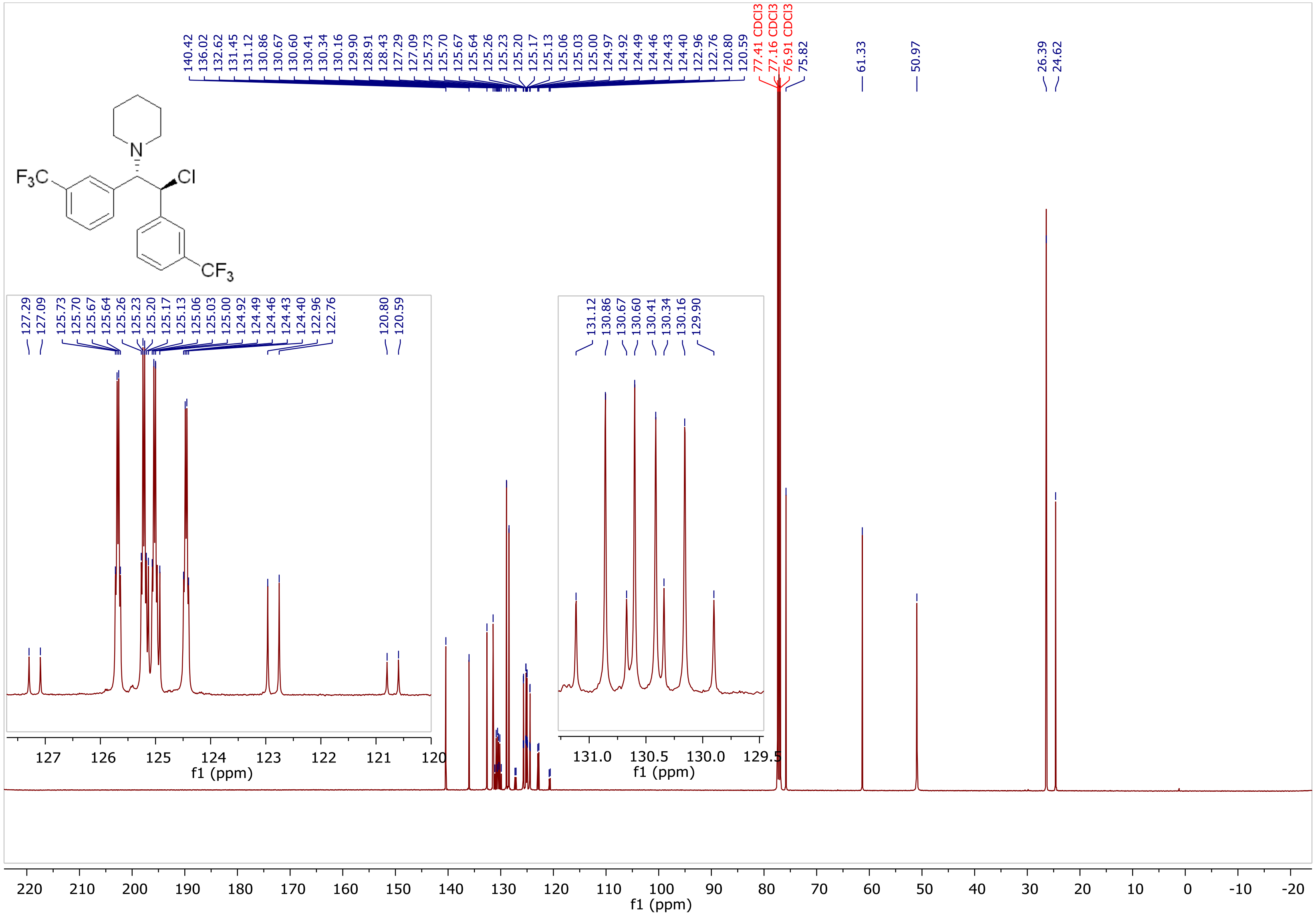

(±)-1,2-bis(4-bromophenyl)-2-(piperidin-1-yl)ethan-1-ol

^{1}H NMR (400 MHz, $CDCl_3$)

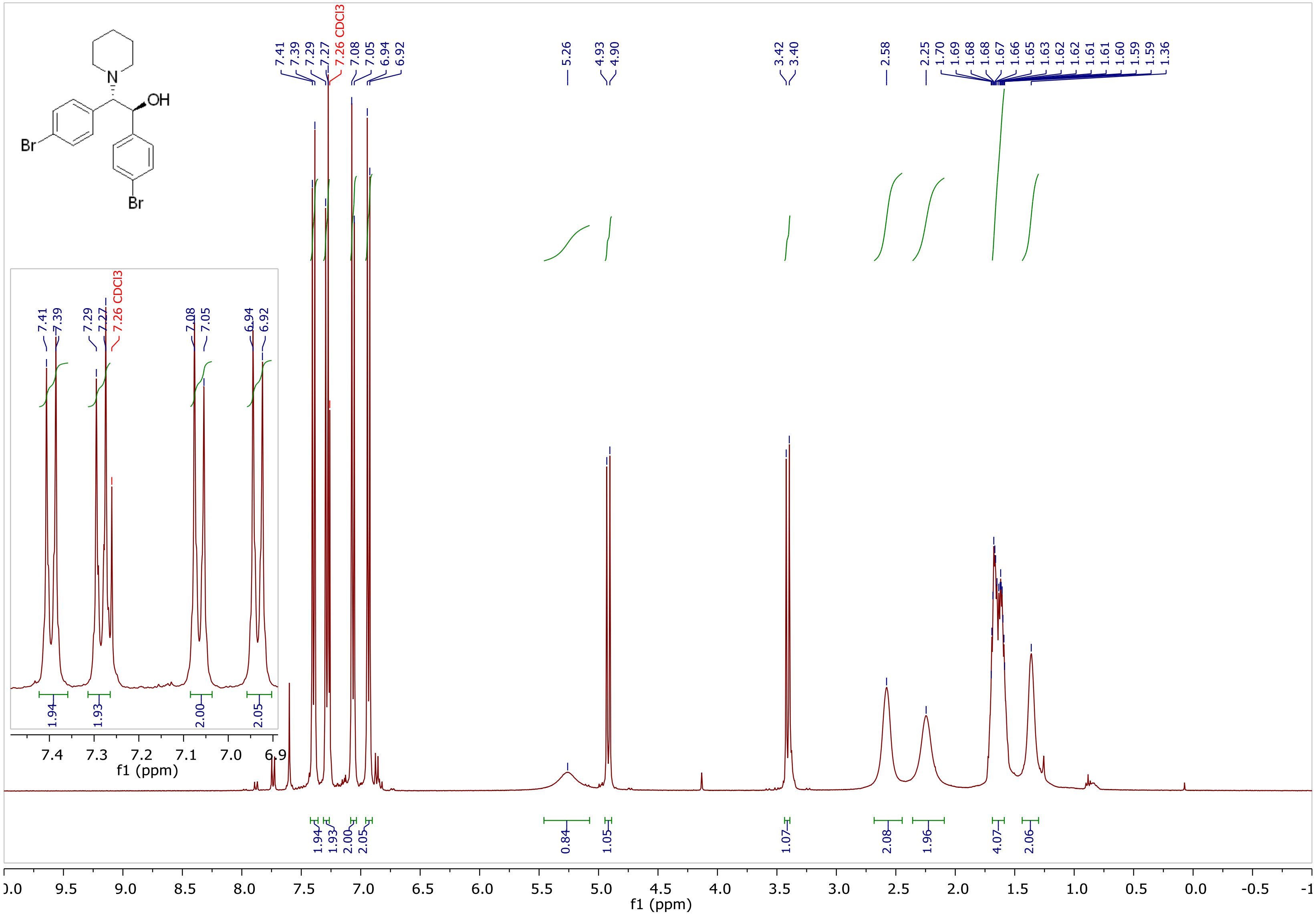

^{13}C NMR (101 MHz, $CDCl_3$)

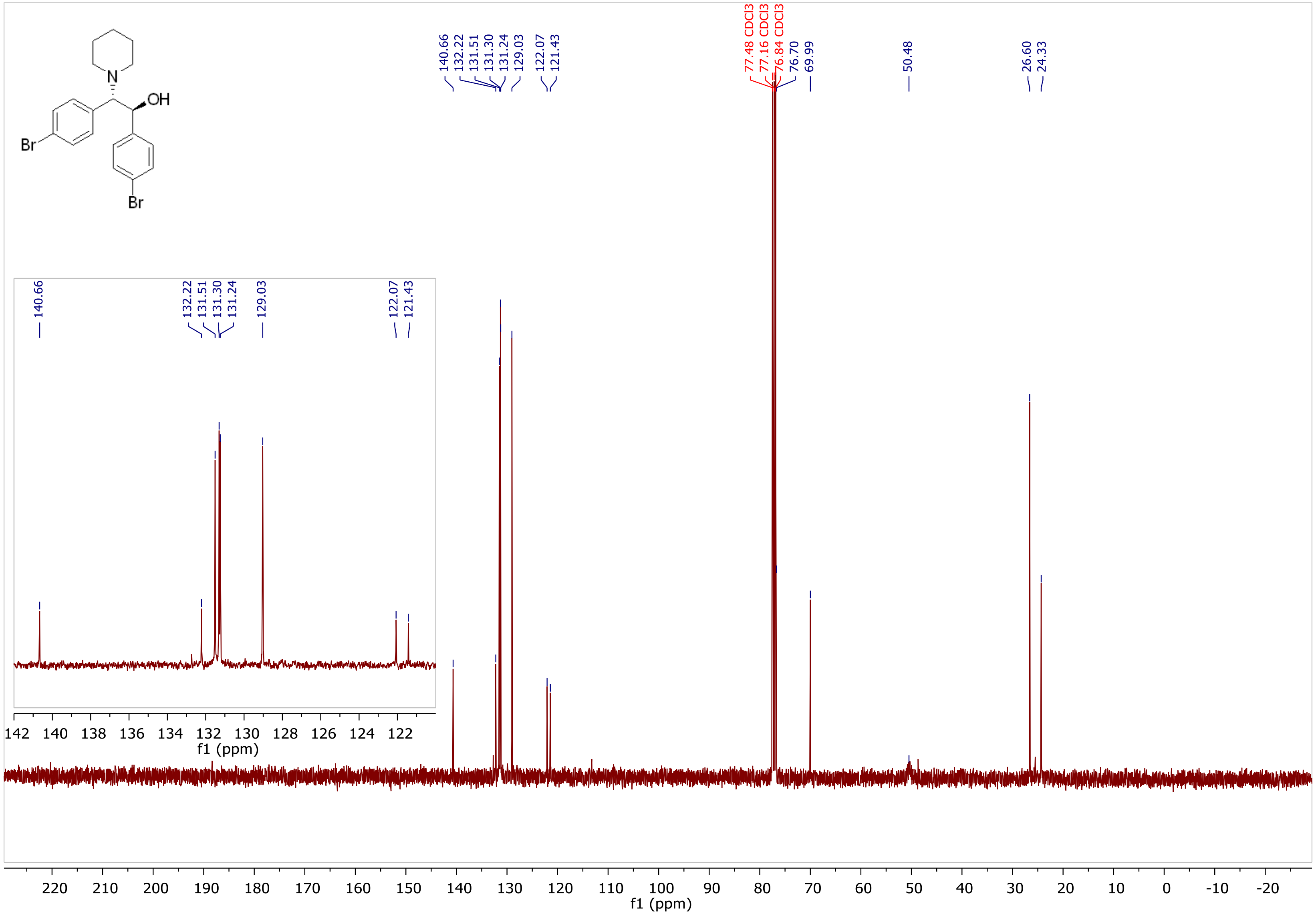

(±)-1-(1,2-bis(4-bromophenyl)-2-chloroethyl)piperidine (2o)

^{1}H NMR (400 MHz, $CDCl_3$)

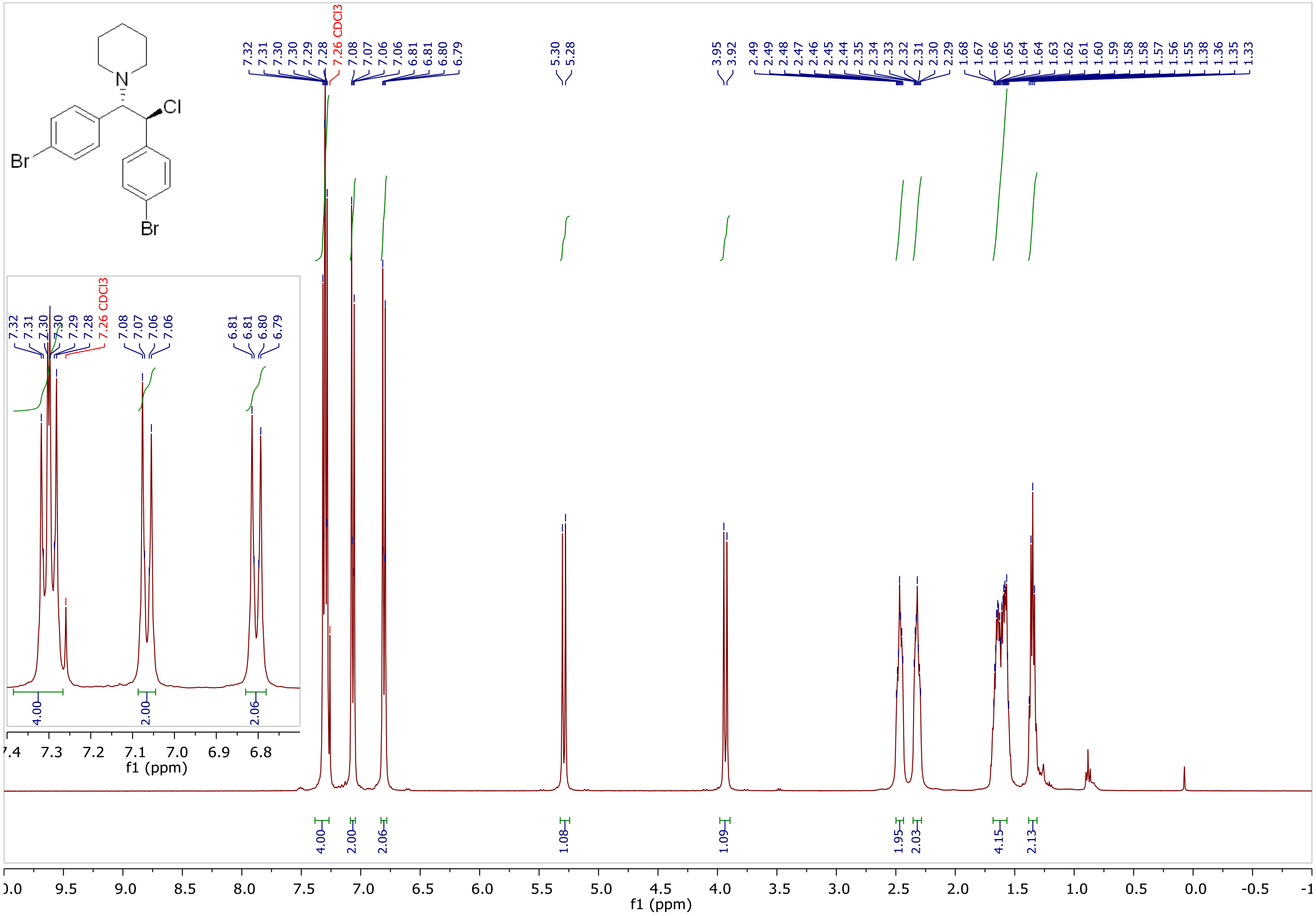

^{13}C NMR (101 MHz, $CDCl_3$)

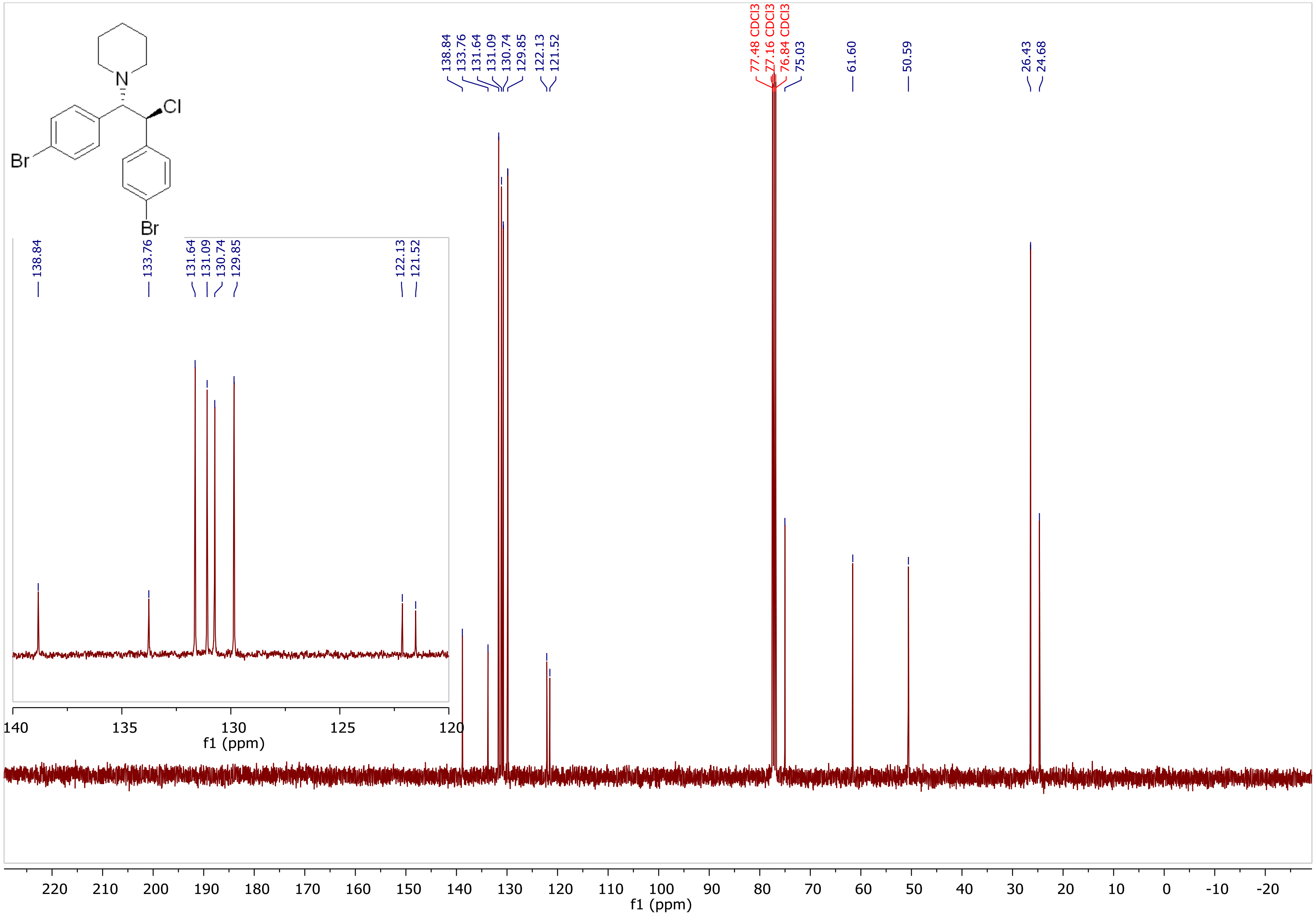

(*Z*)-1,2-bis(4-(*tert*-butyl)phenyl)ethene

^{1}H NMR (400 MHz, $CDCl_3$)

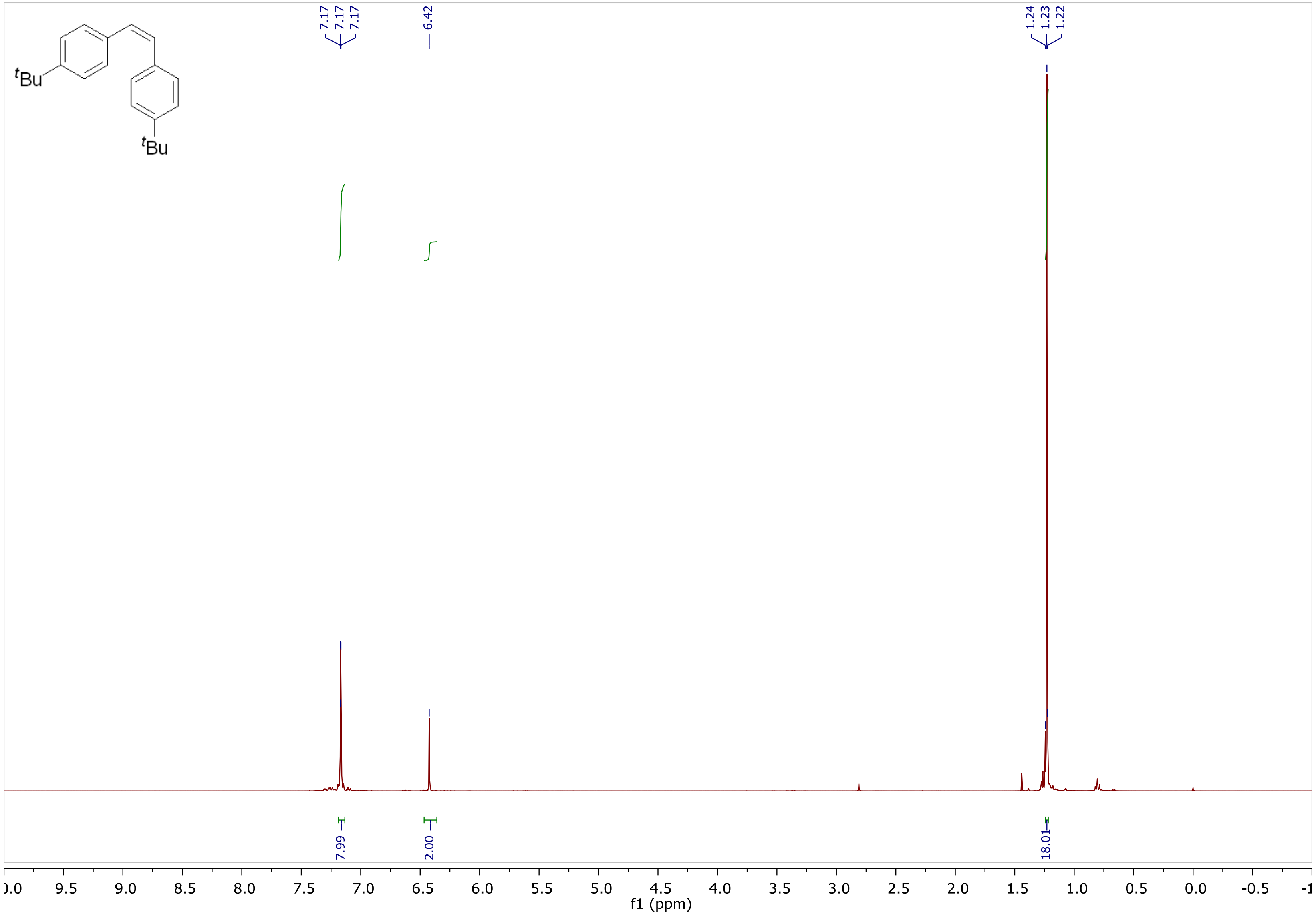

^{13}C NMR (101 MHz, $CDCl_3$)

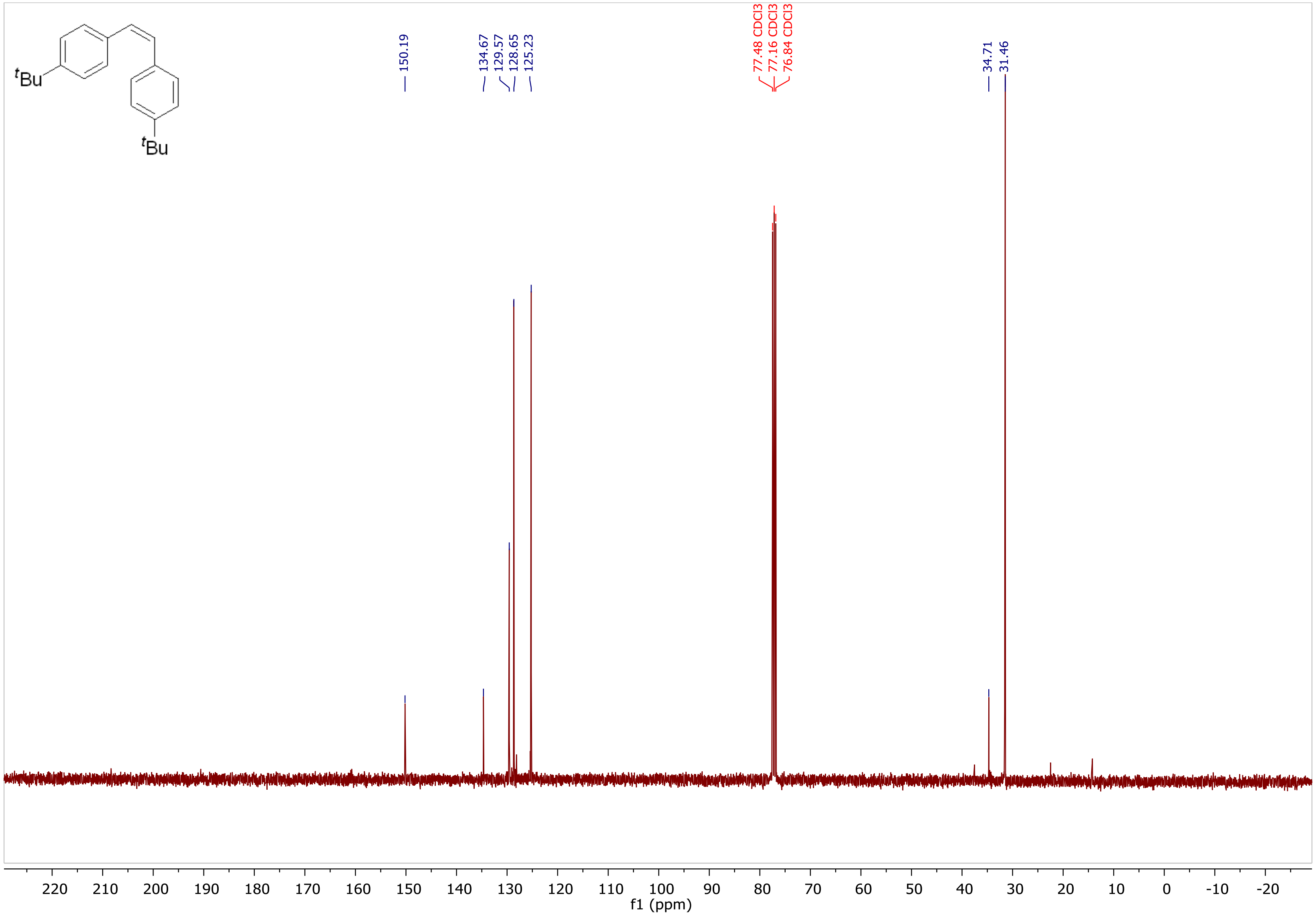

2,3-bis(4-(*tert*-butyl)phenyl)oxirane

^{1}H NMR (400 MHz, $CDCl_3$)

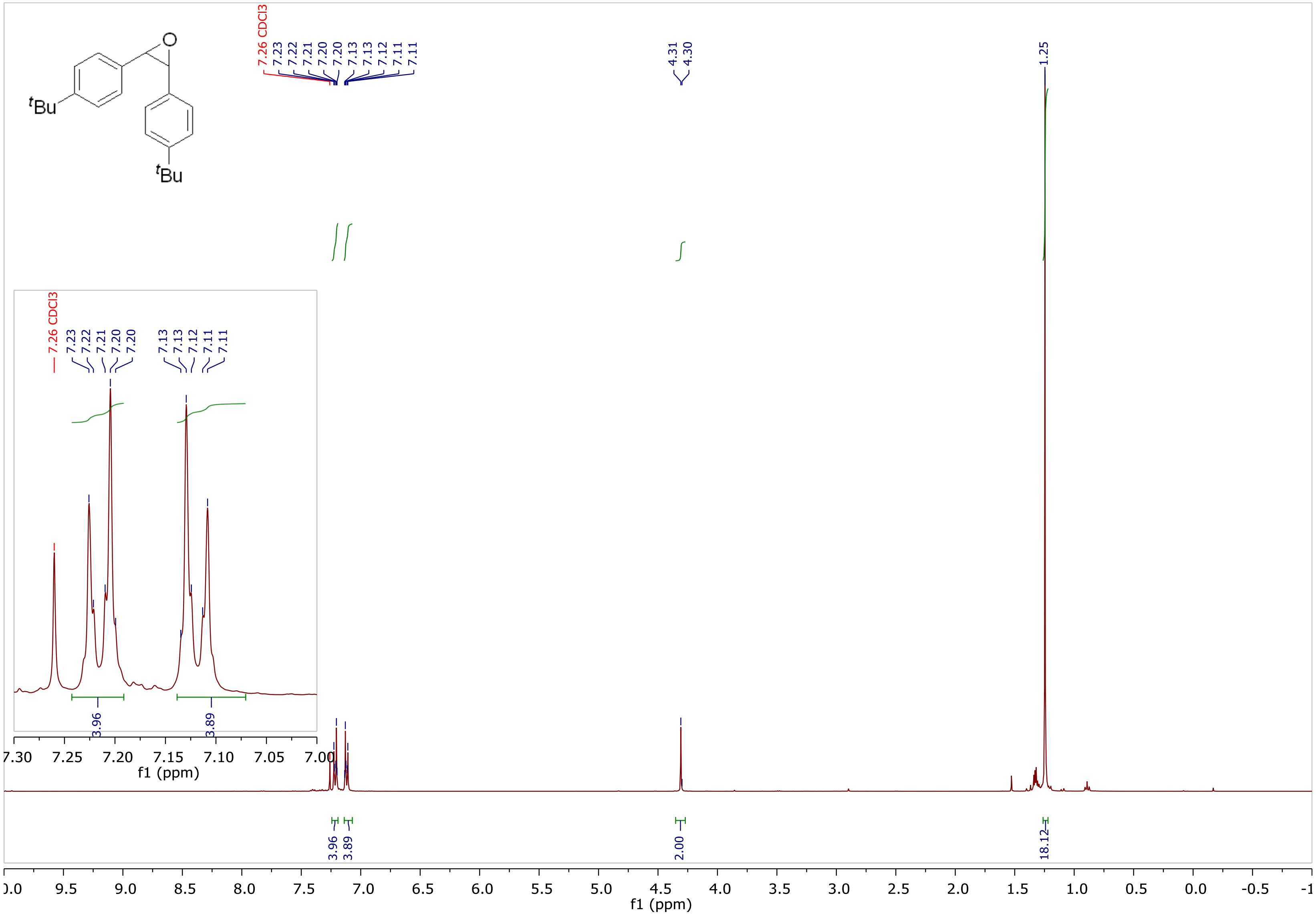

^{13}C NMR (101 MHz, $CDCl_3$)

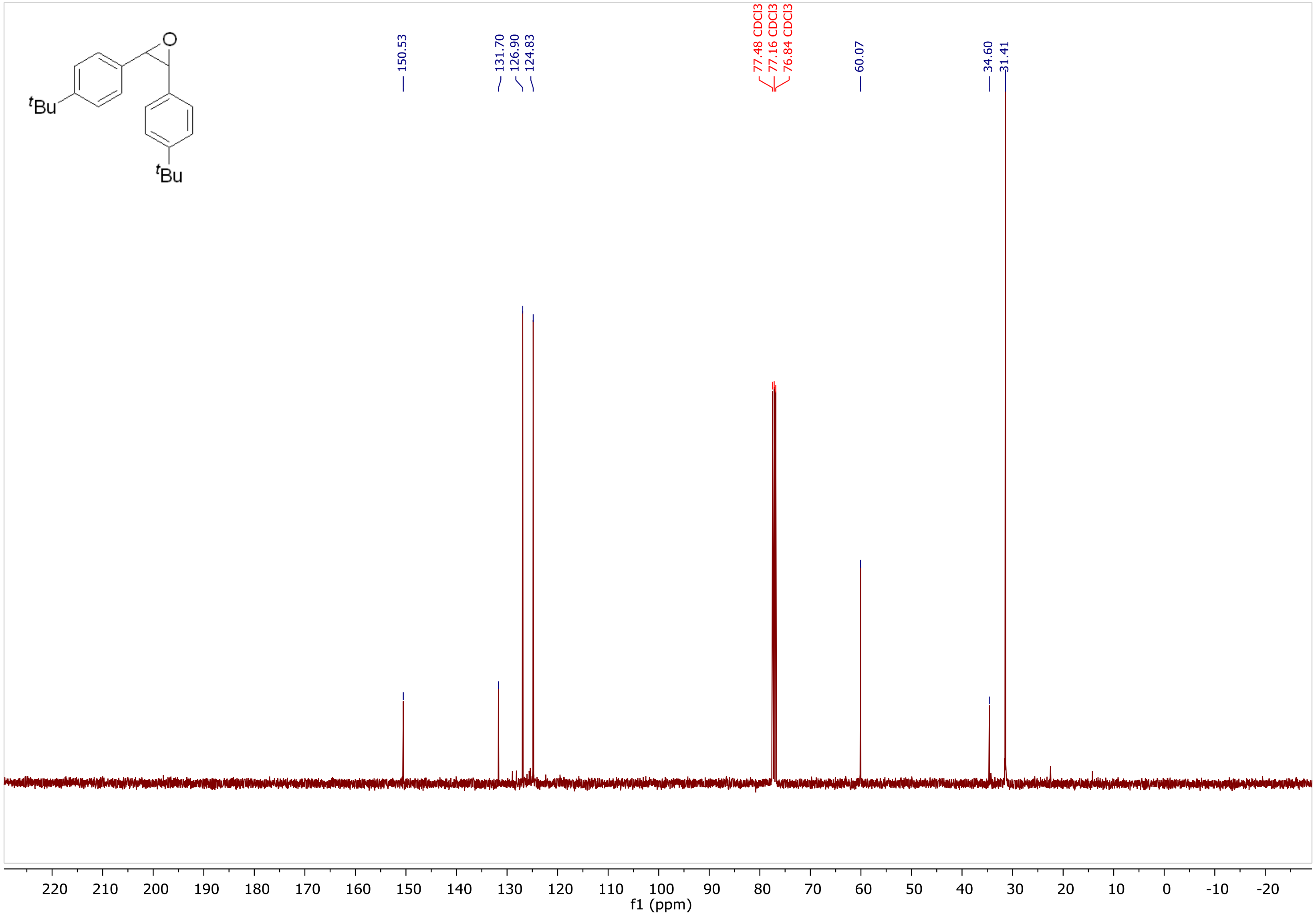

(±)-1,2-bis(4-(*tert*-butyl)phenyl)-2-(piperidin-1-yl)ethan-1-ol

^{1}H NMR (400 MHz, $CDCl_3$)

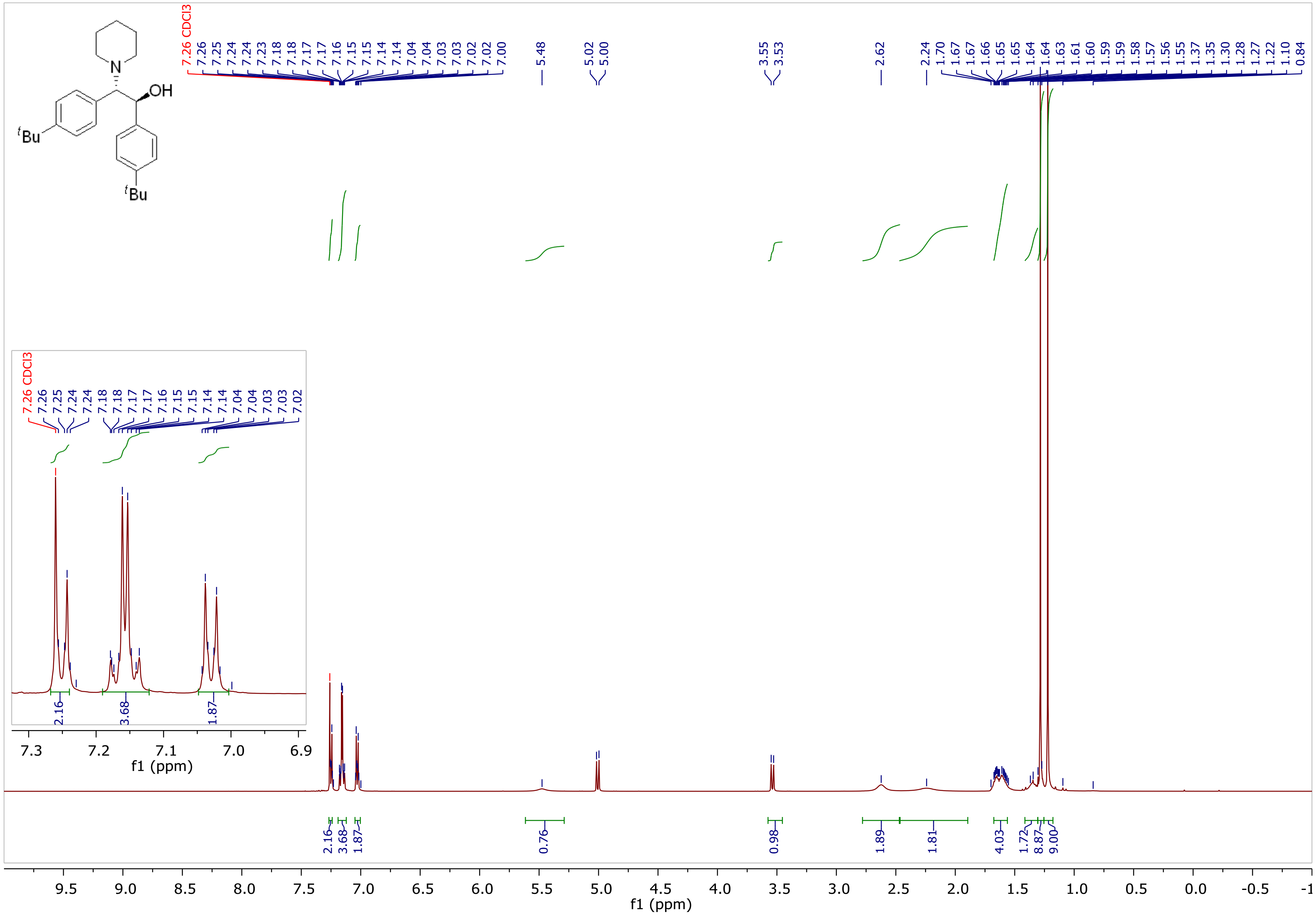

^{13}C NMR (101 MHz, $CDCl_3$)

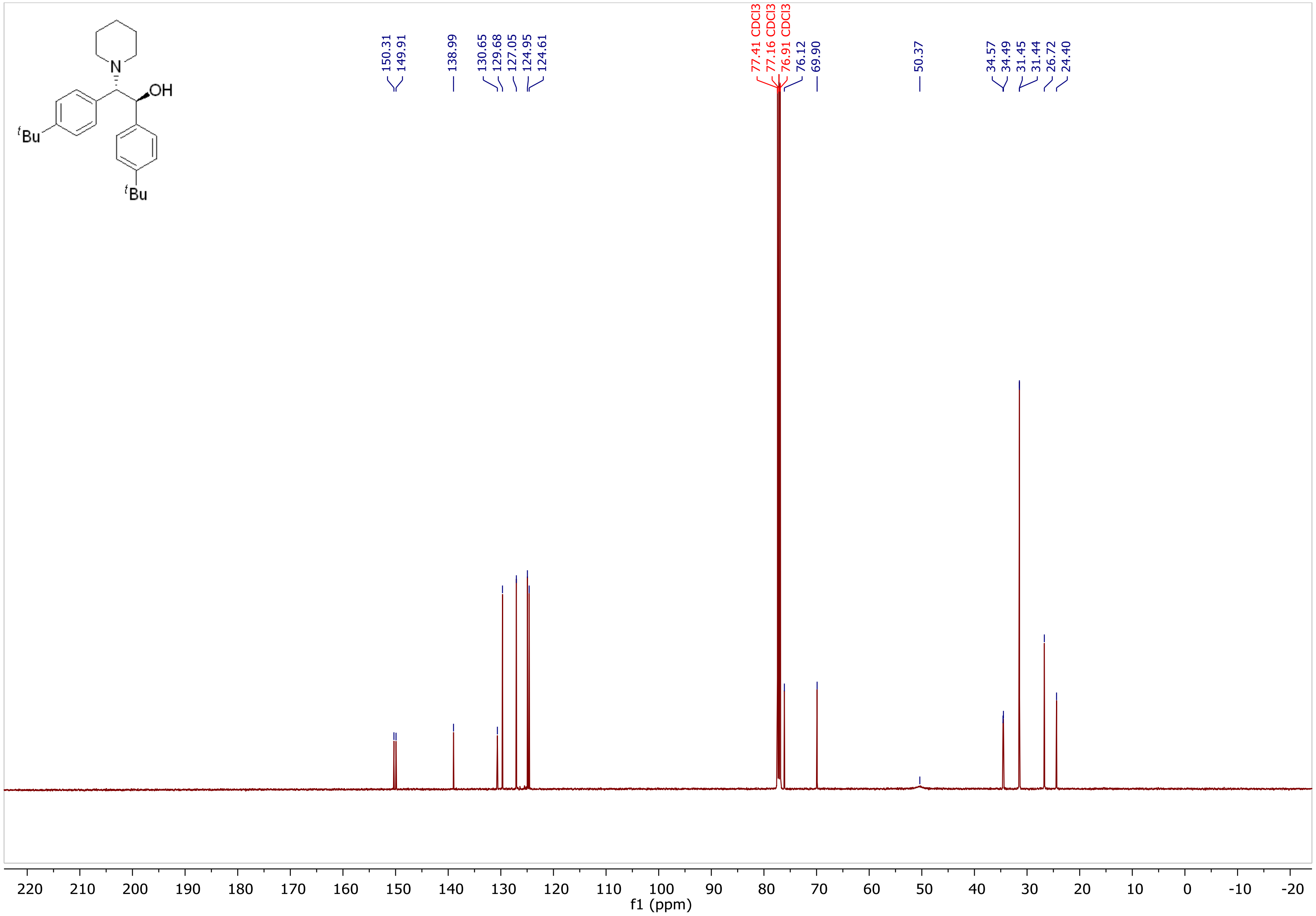

(±)-1-(1,2-bis(4-(*tert*-butyl)phenyl)-2-chloroethyl)piperidine (2p)

^{1}H NMR (400 MHz, $CDCl_3$)

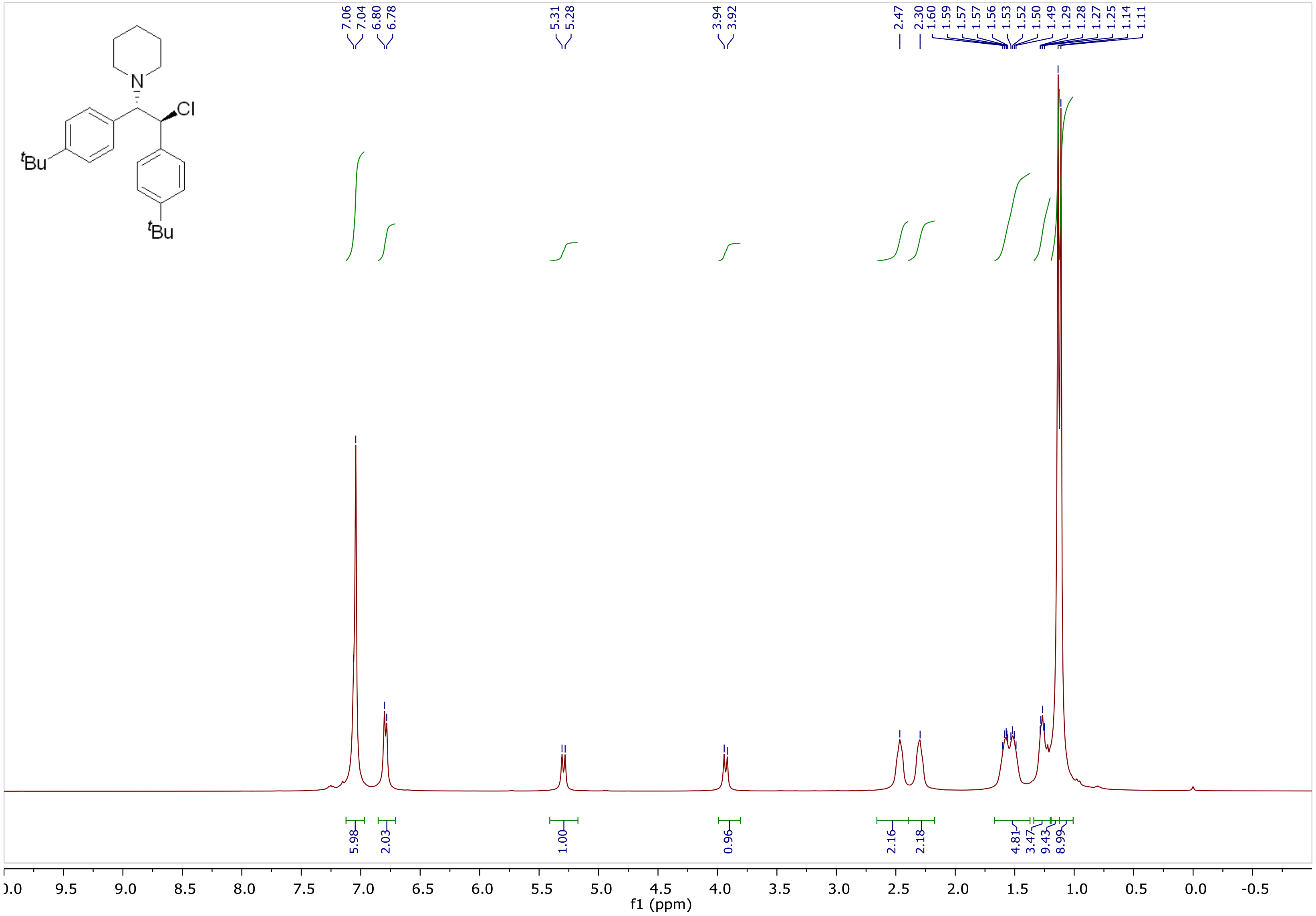

^13^C NMR (101 MHz, $CDCl_3$)

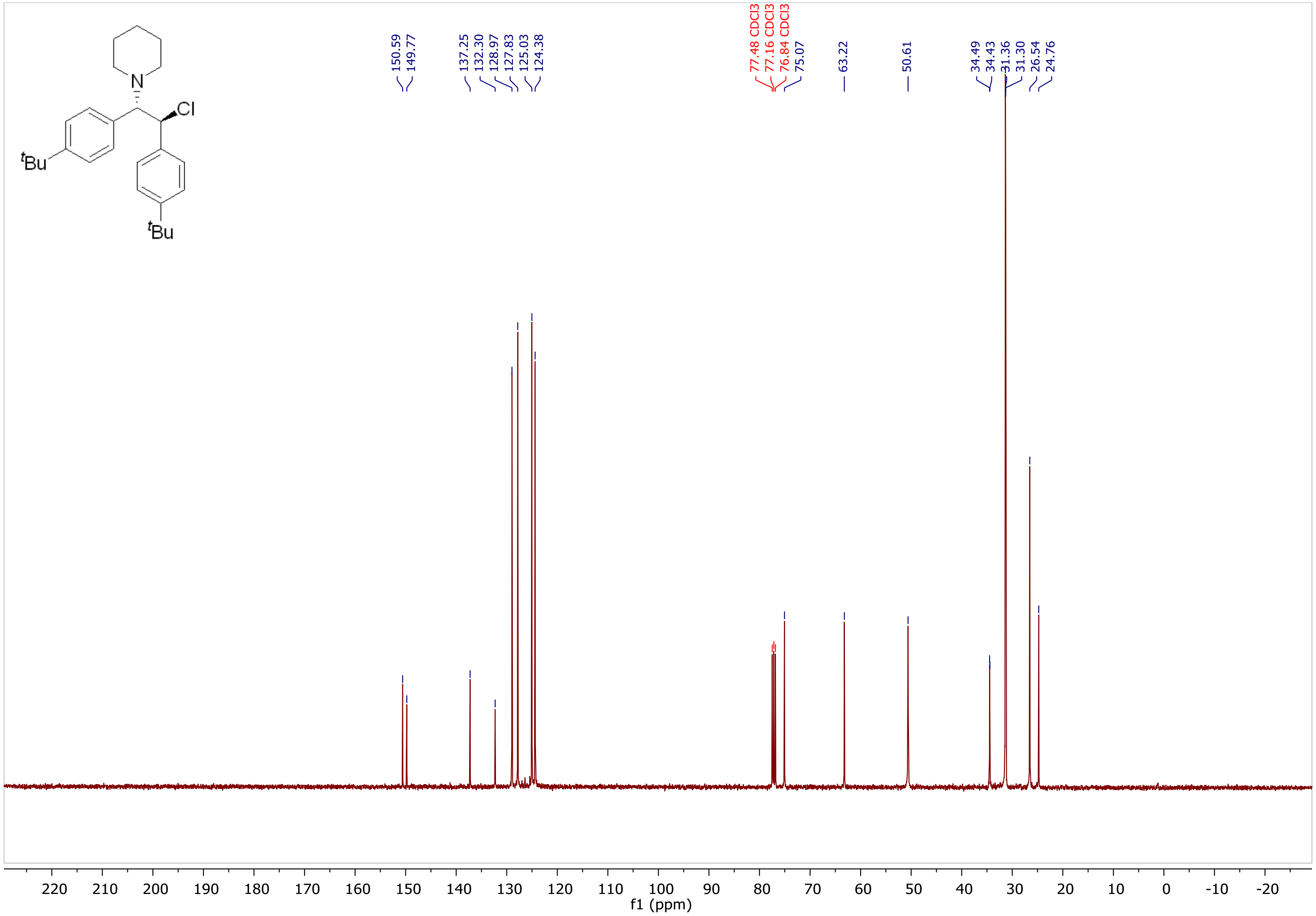

(±)-2-(piperidin-1-yl)-1,2-bis(4-(trifluoromethyl)phenyl)ethan-1-ol

^{1}H NMR (500 MHz, $CDCl_3$)

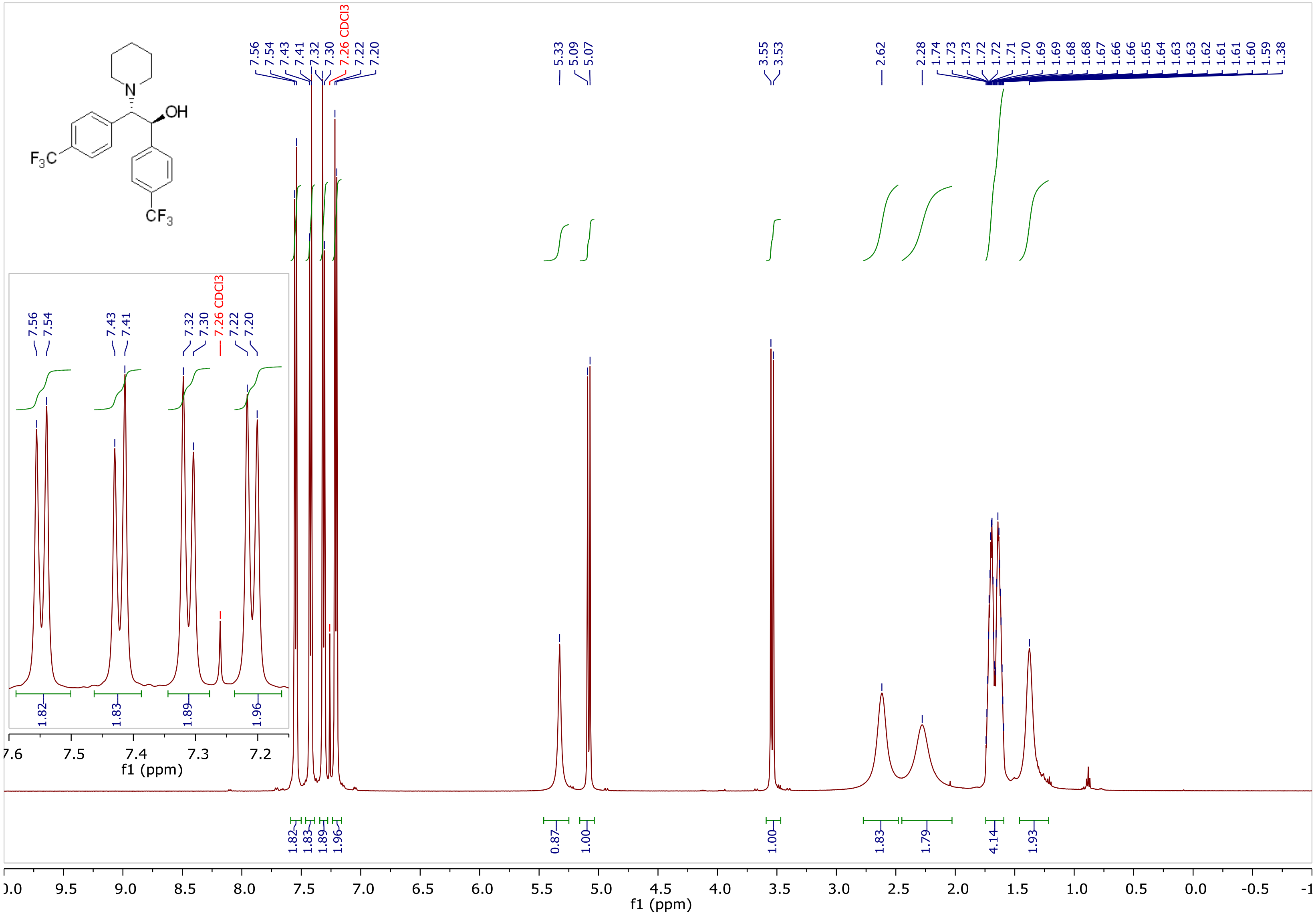

^{19}F NMR (471 MHz, $CDCl_3$)

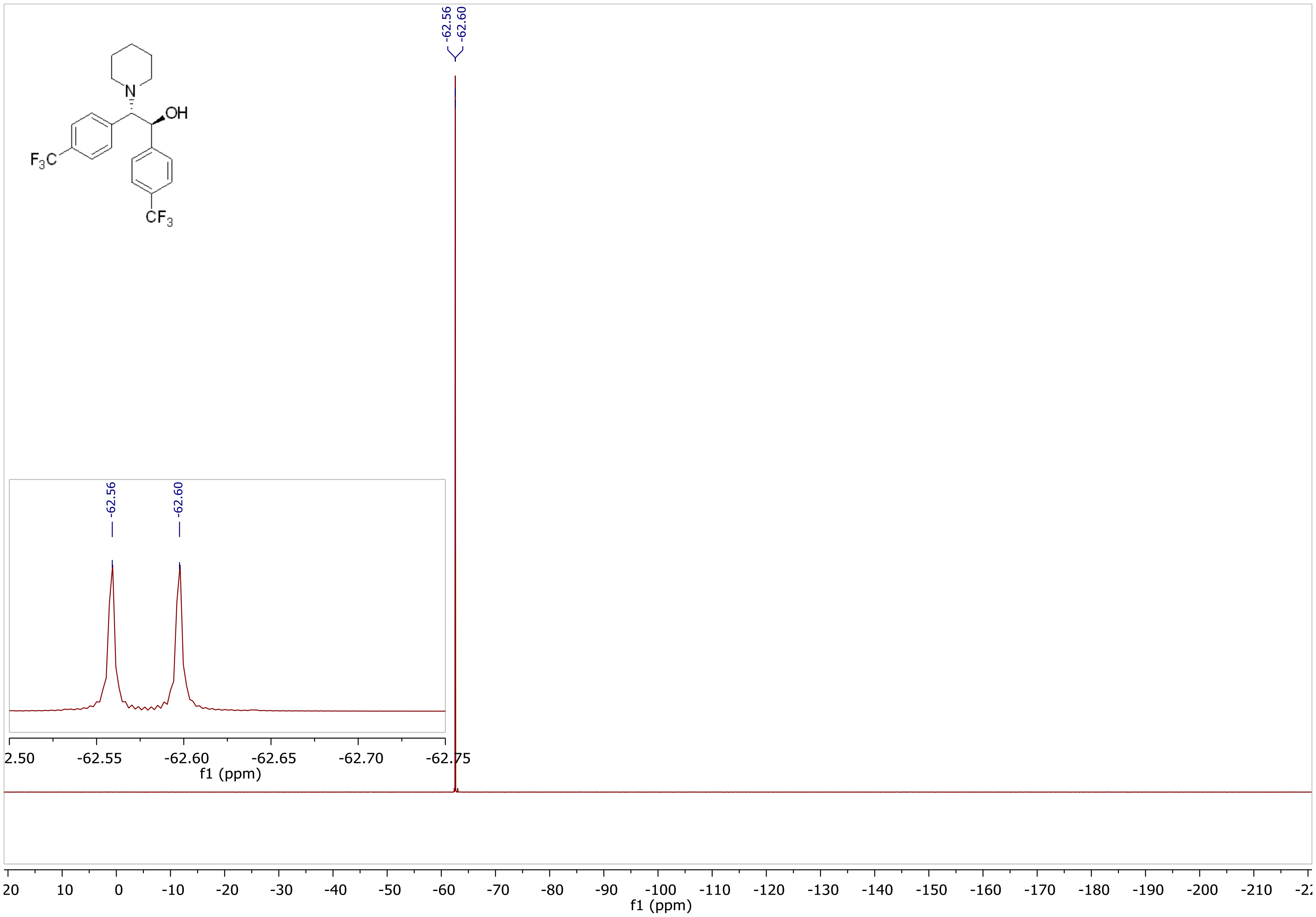

^{13}C NMR (126 MHz, $CDCl_3$)

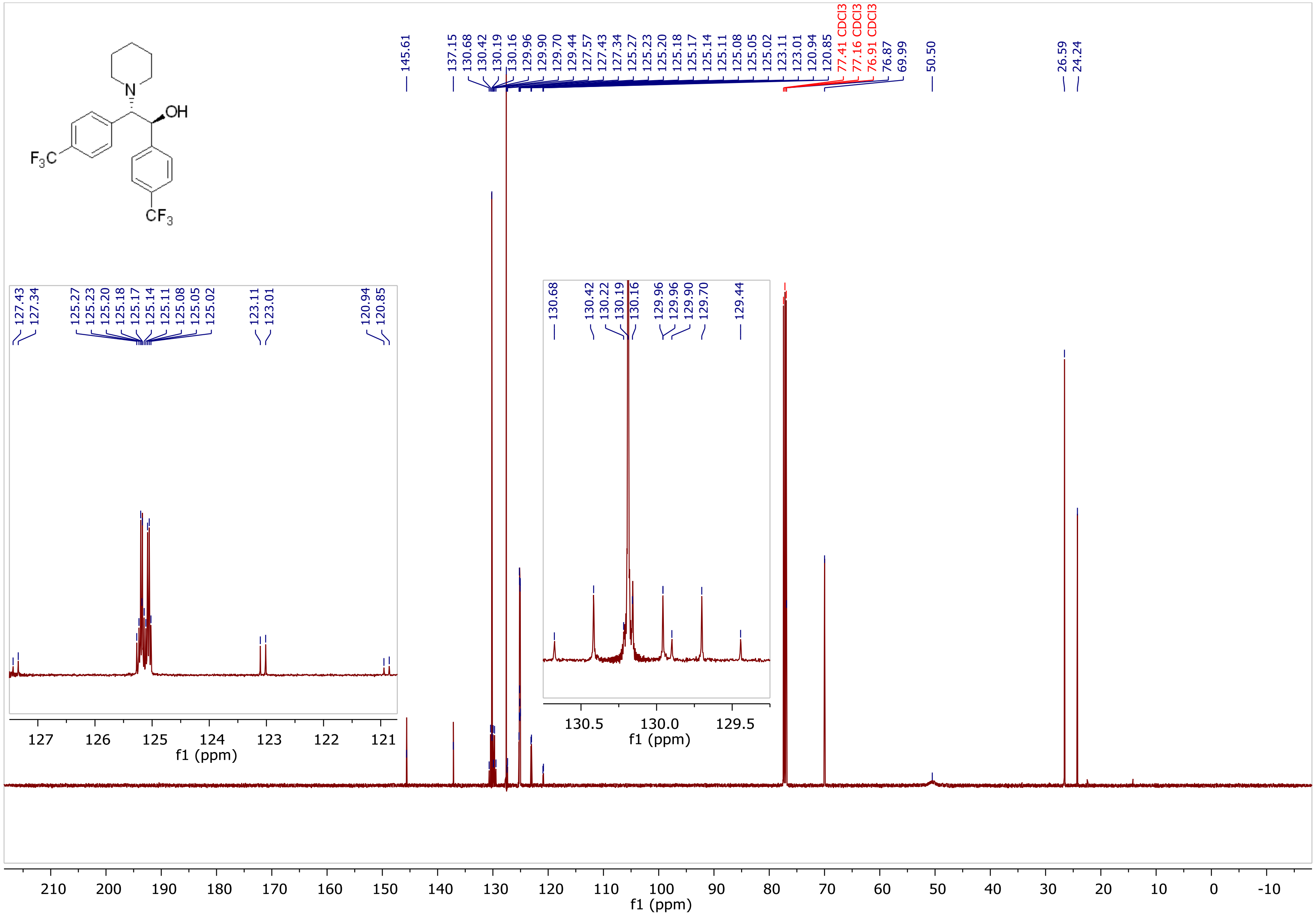

(±)-1-(2-chloro-1,2-bis(4-(trifluoromethyl)phenyl)ethyl)piperidine (2q)

^{1}H NMR (500 MHz, $CDCl_3$)

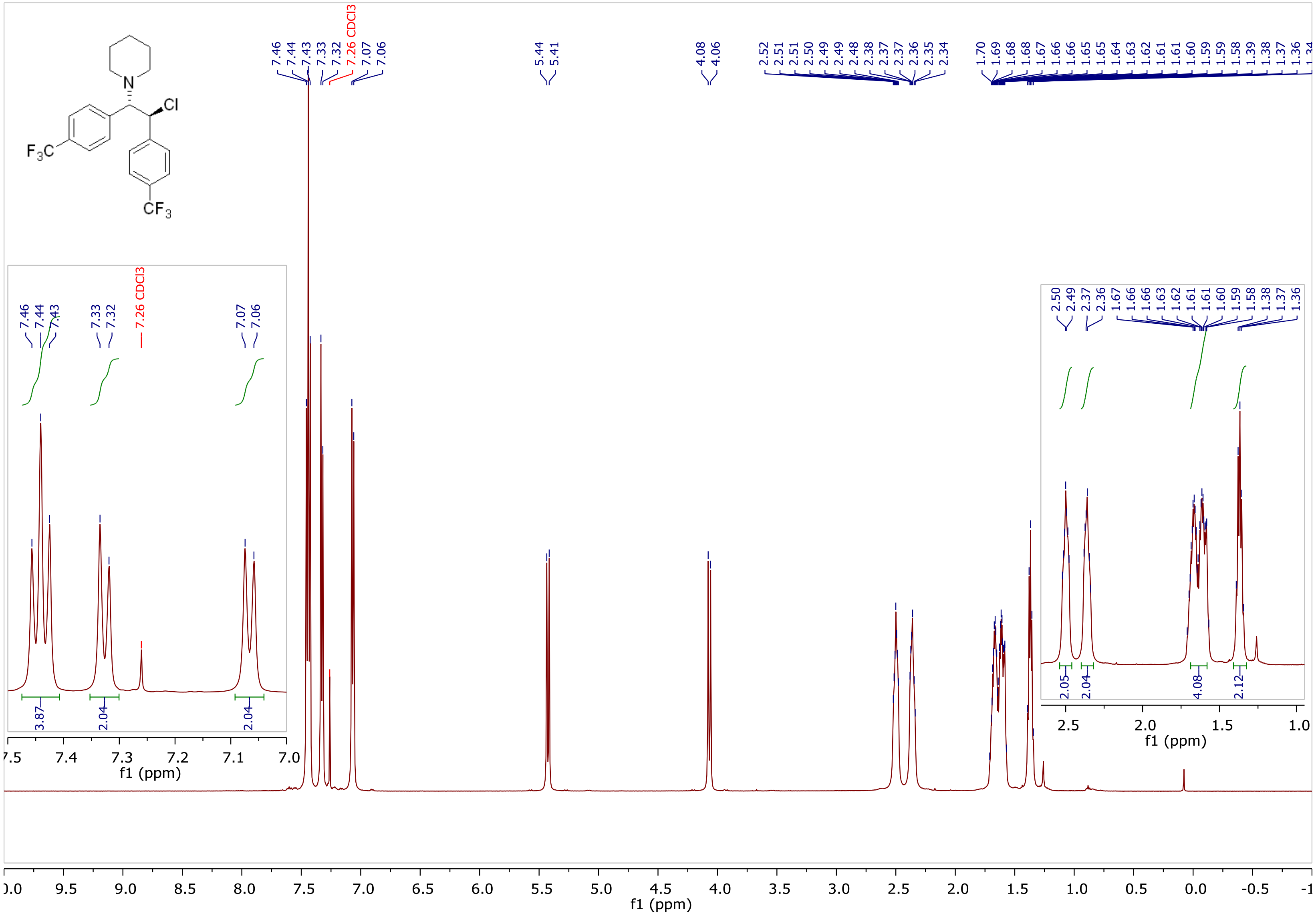

^{19}F NMR (471 MHz, $CDCl_3$)

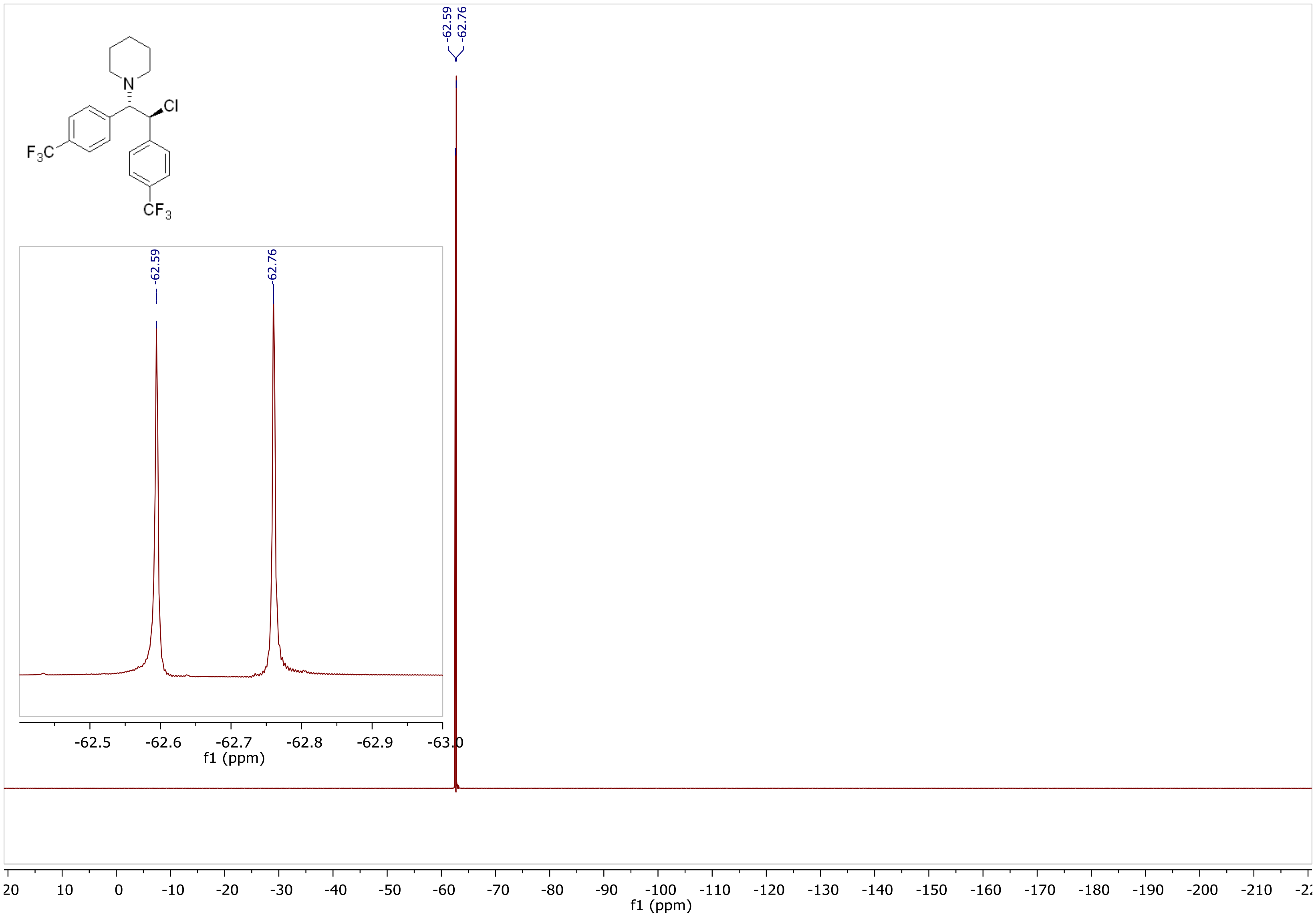

^{13}C NMR (126 MHz, $CDCl_3$)

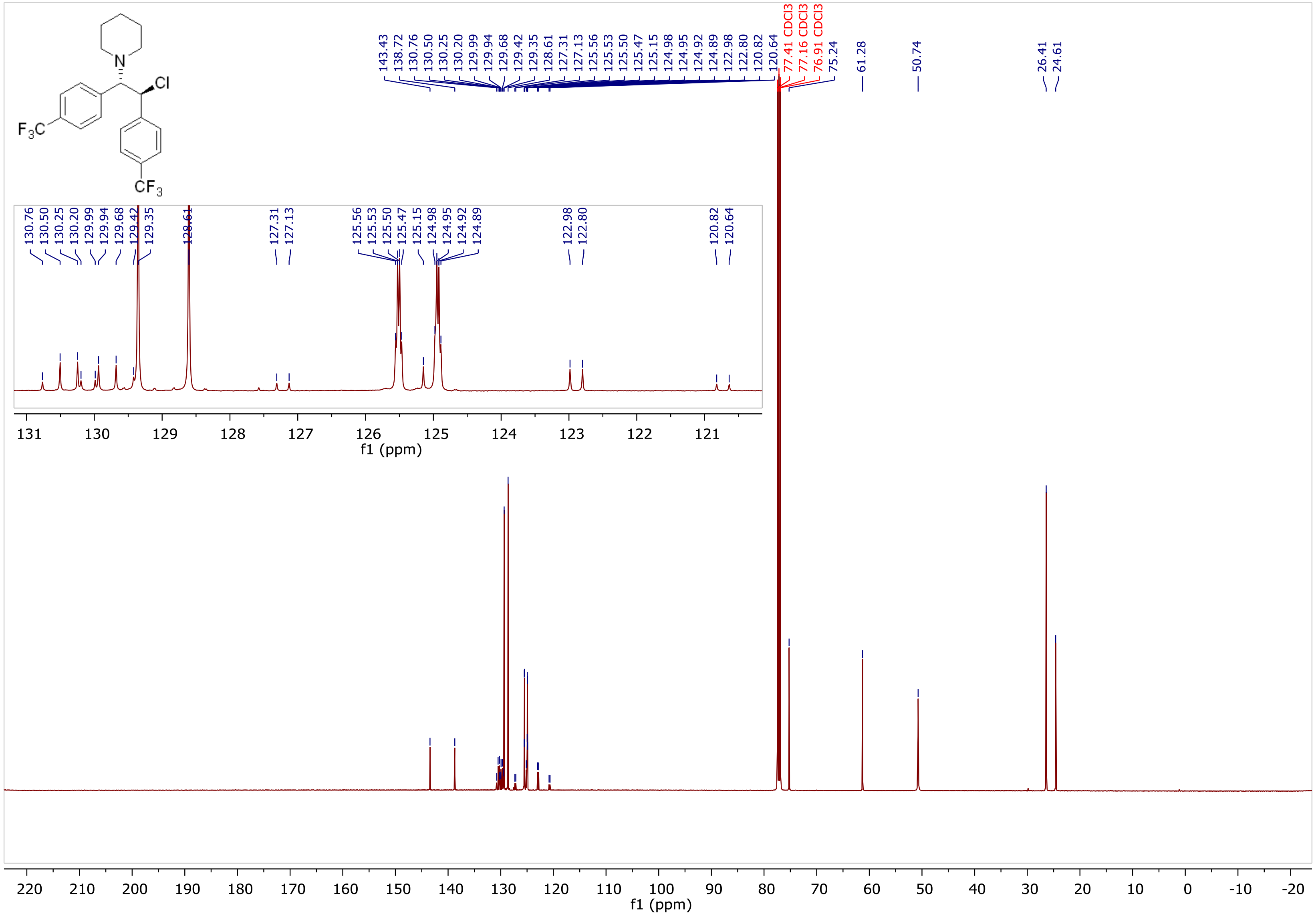

(±)-*N*-(2-chloro-1,2-di(pyridin-2-yl)ethyl)-*N*-propylpropan-1-amine (2r)

^{1}H NMR (400 MHz, $CDCl_3$)

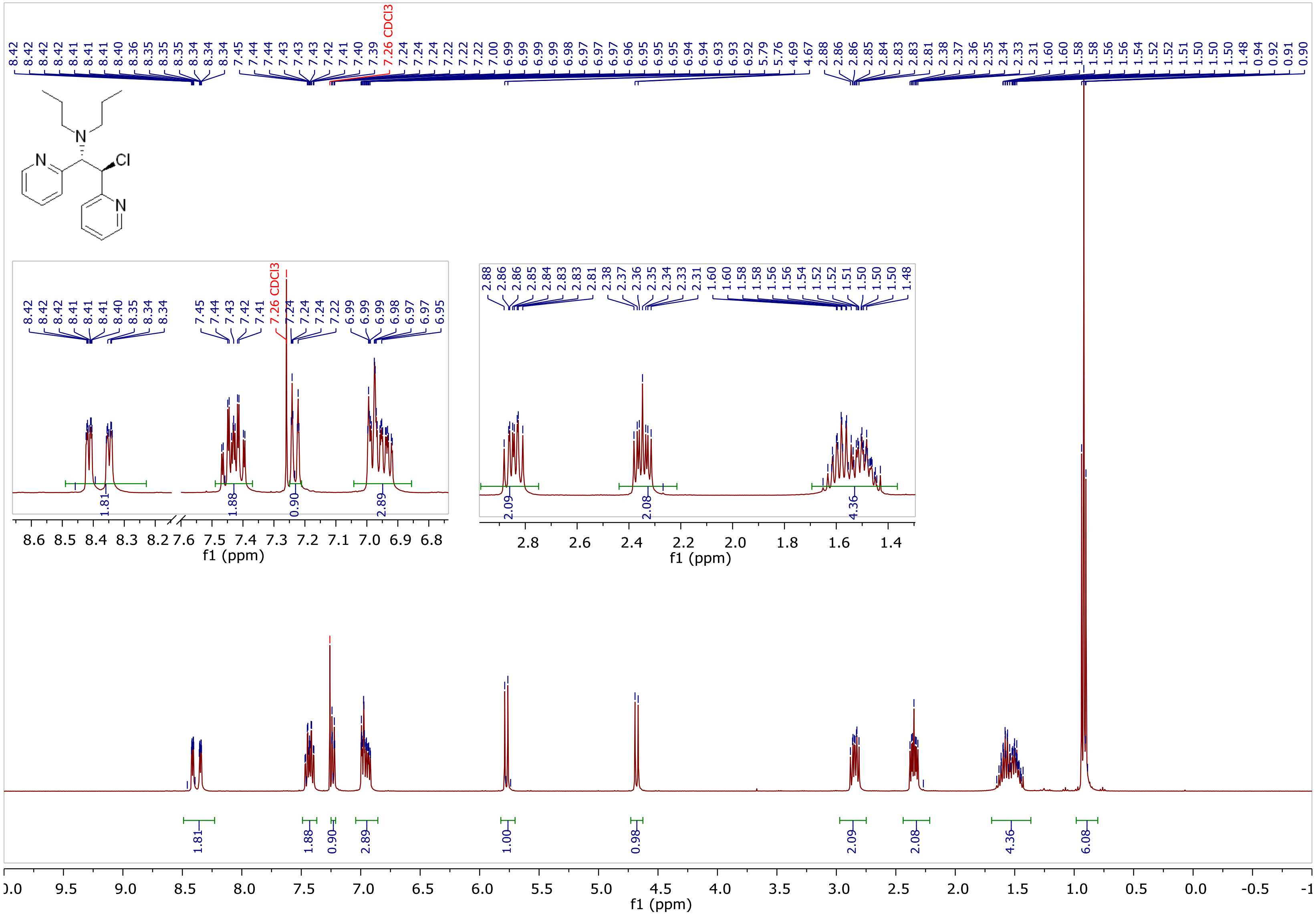

^{13}C NMR (101 MHz, $CDCl_3$)

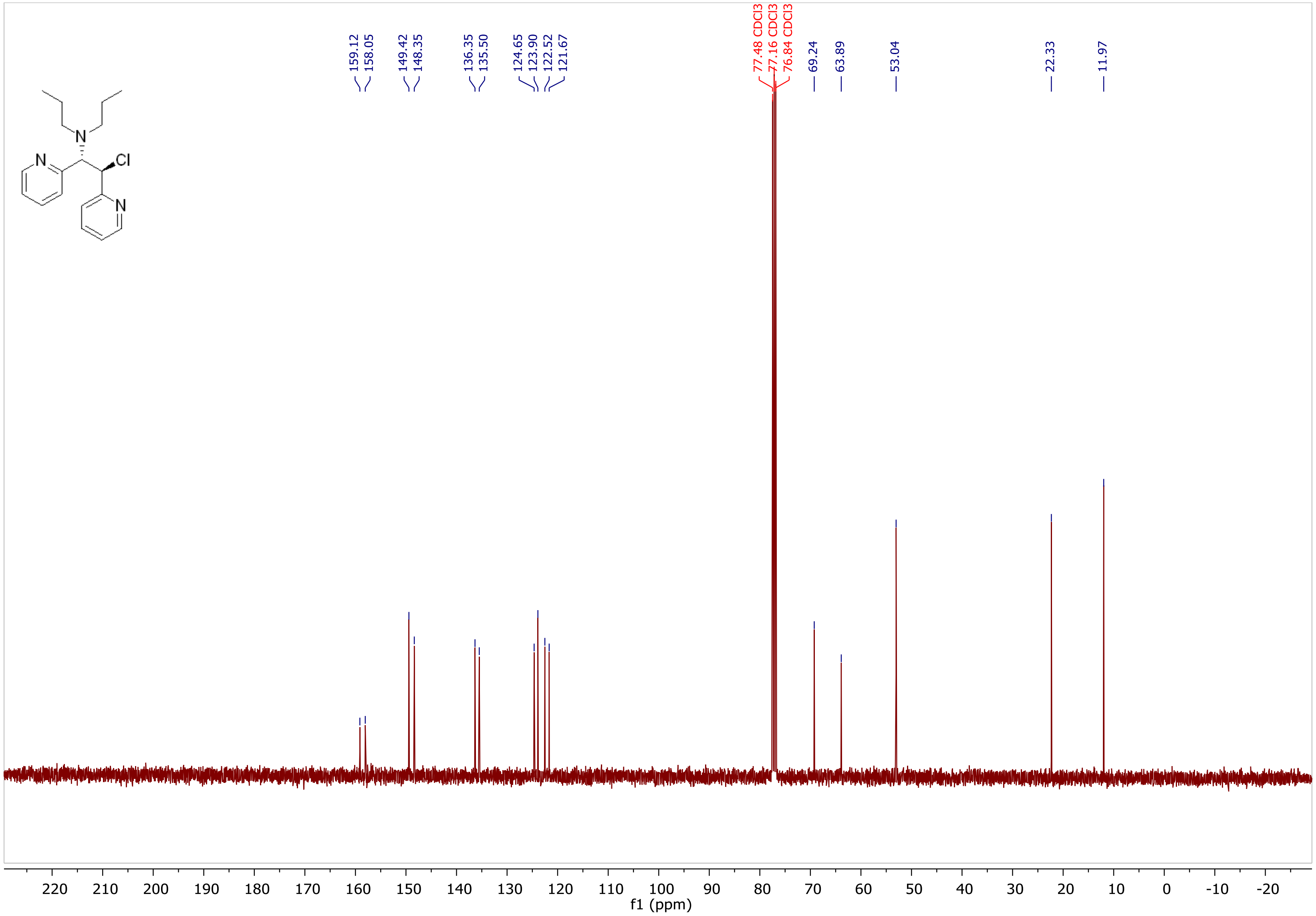

(±)-*N*-benzyl-2-chloro-*N*-methylcyclohexan-1-amine (2s)

^{1}H NMR (400 MHz, $CDCl_3$)

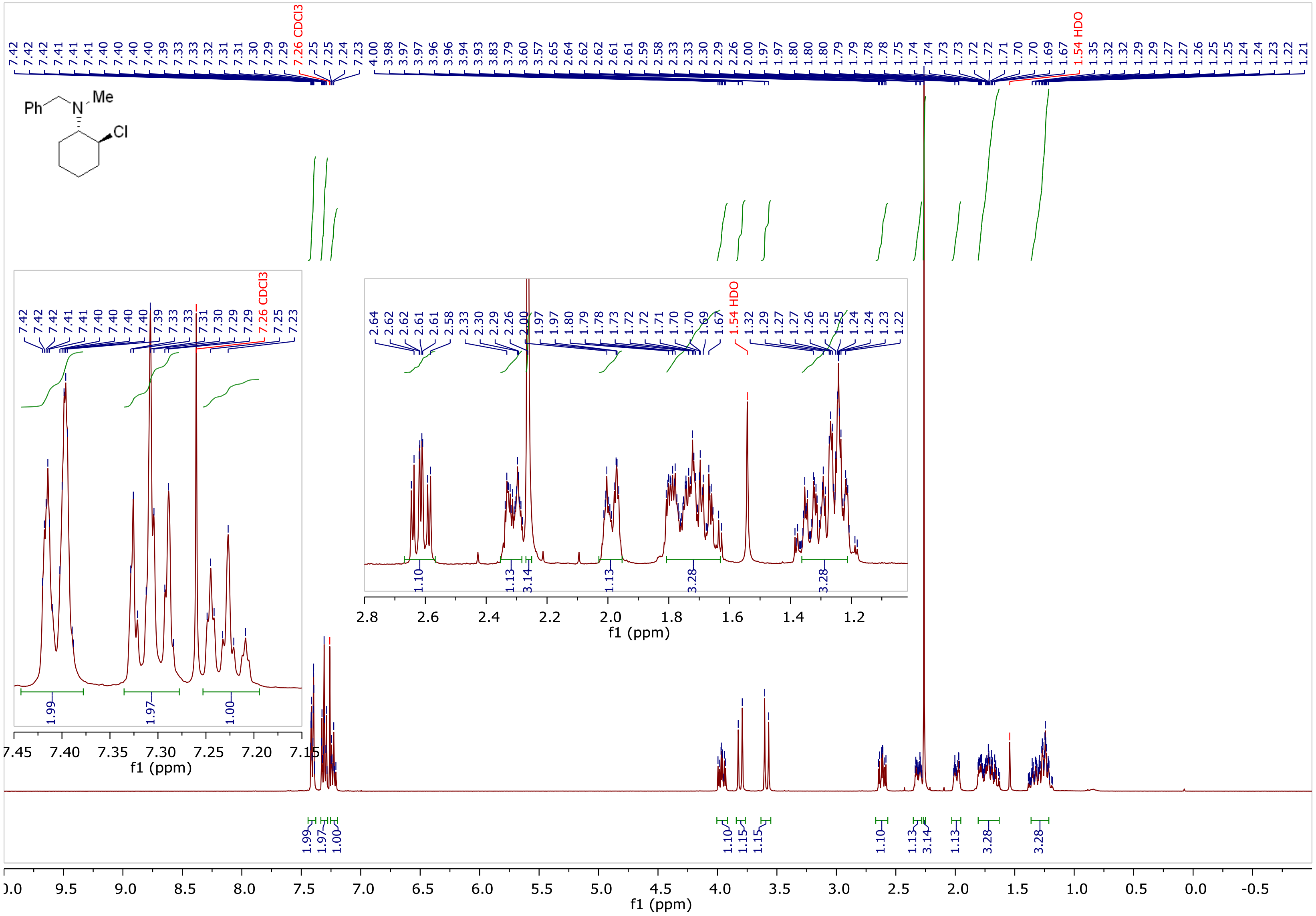

^{13}C NMR (101 MHz, $CDCl_3$)

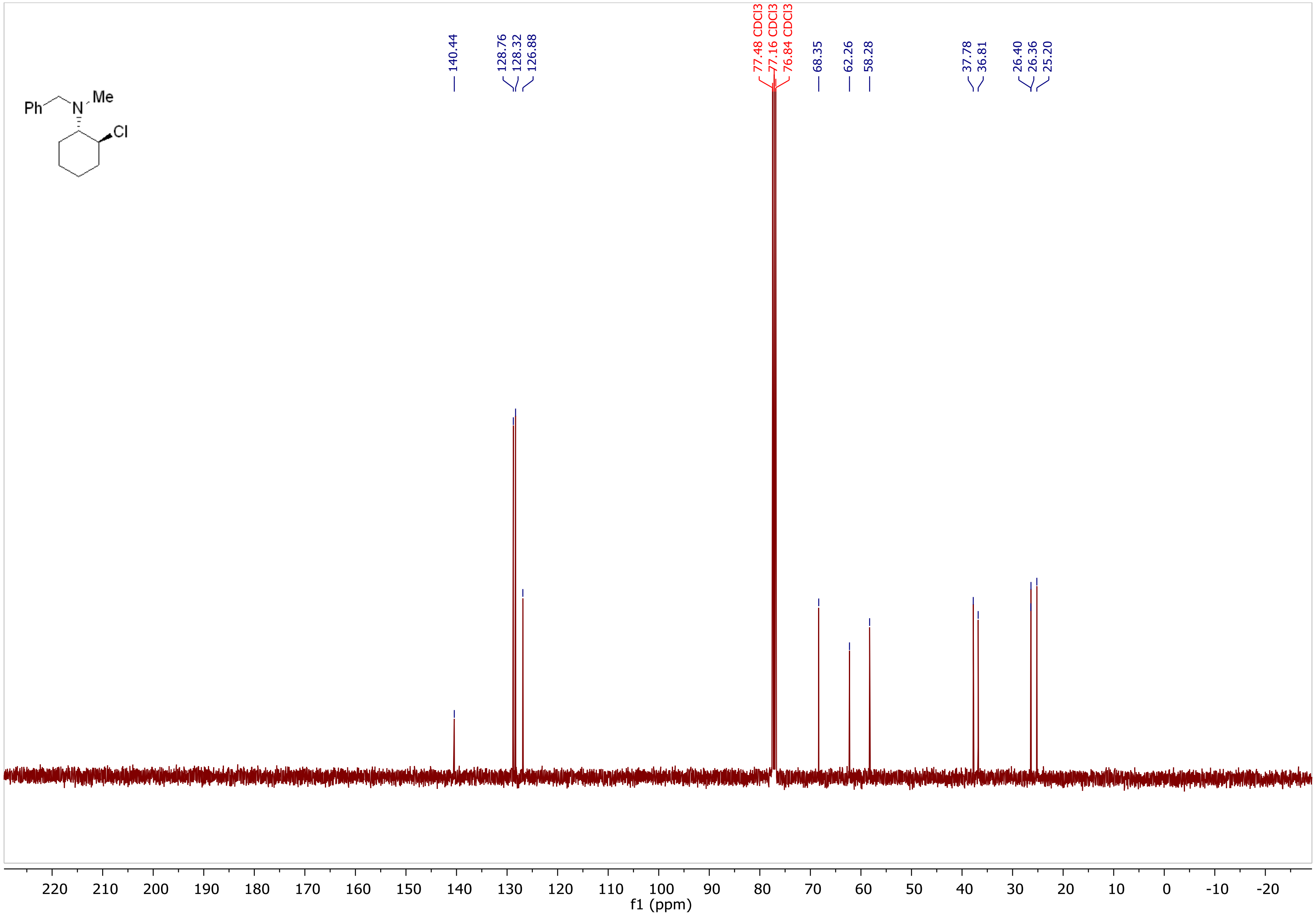

1-((1*S*,2*S*)-2-azido-1,2-diphenylethyl)piperidine (3a)

^{1}H NMR (400 MHz, $CDCl_3$)

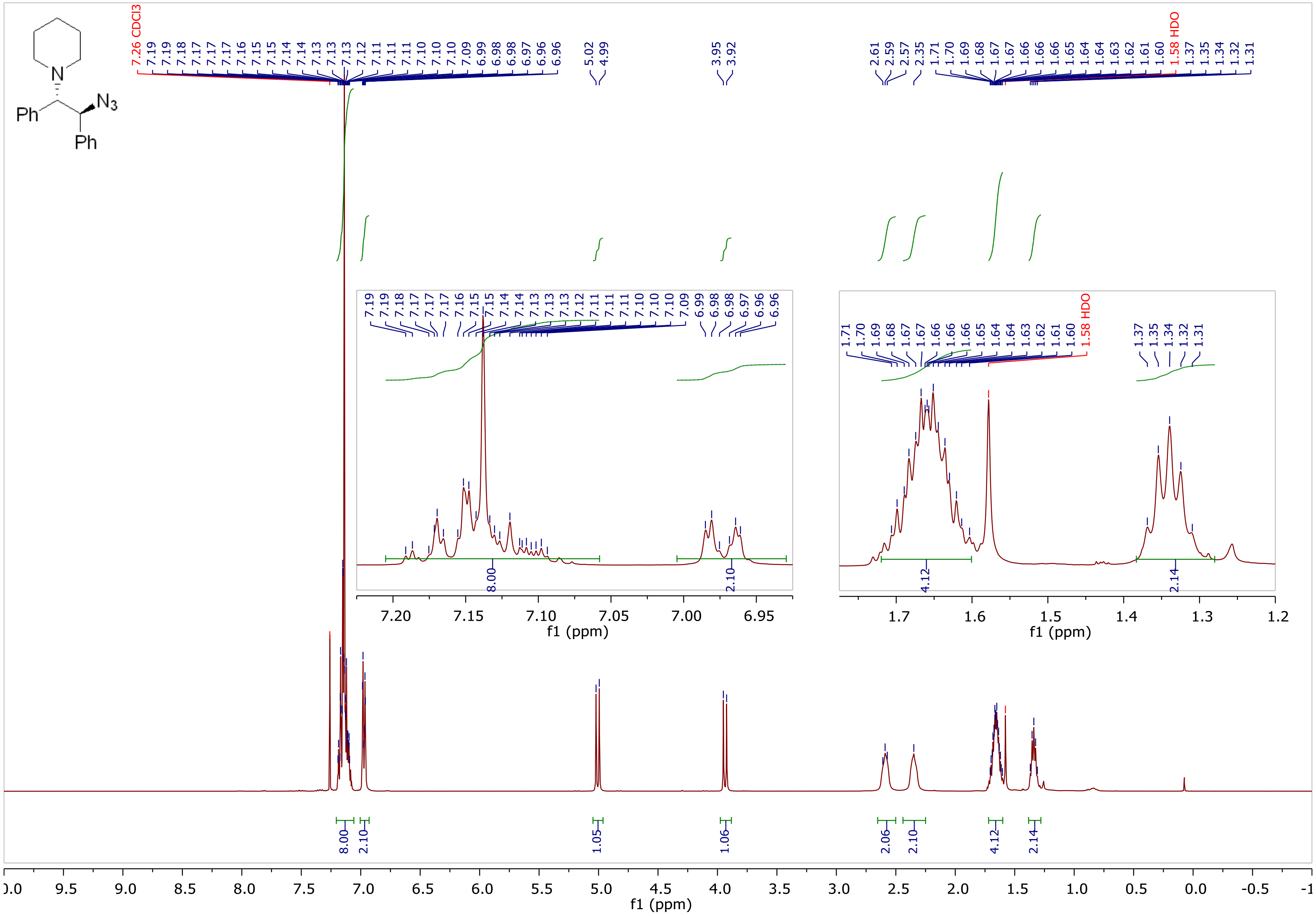

^{13}C NMR (101 MHz, $CDCl_3$)

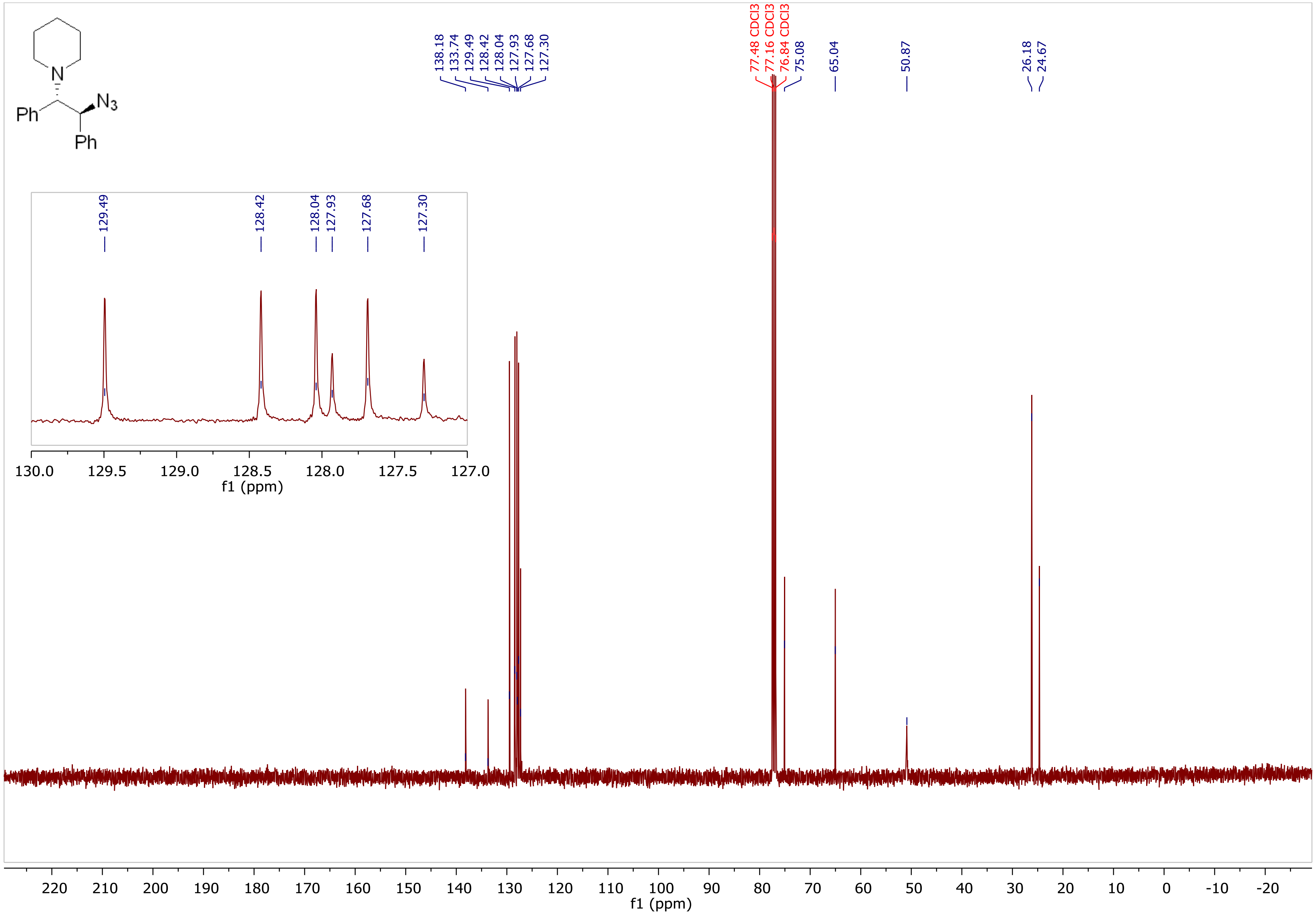

1-((1*S*,2*S*)-2-azido-1,2-diphenylethyl)-4-methoxypiperidine (3b)

^{1}H NMR (400 MHz, $CDCl_3$)

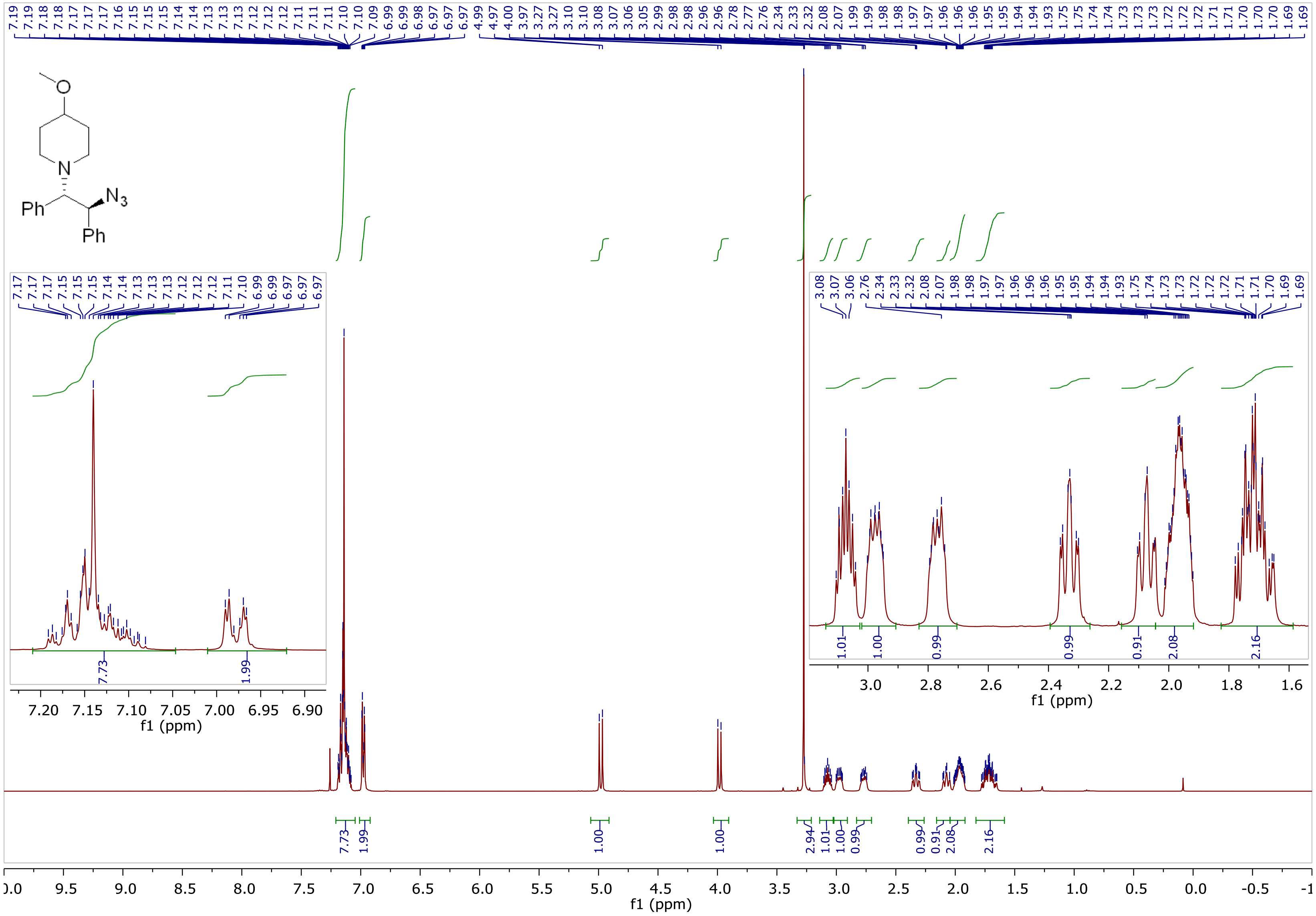

^{13}C NMR (101 MHz, $CDCl_3$)

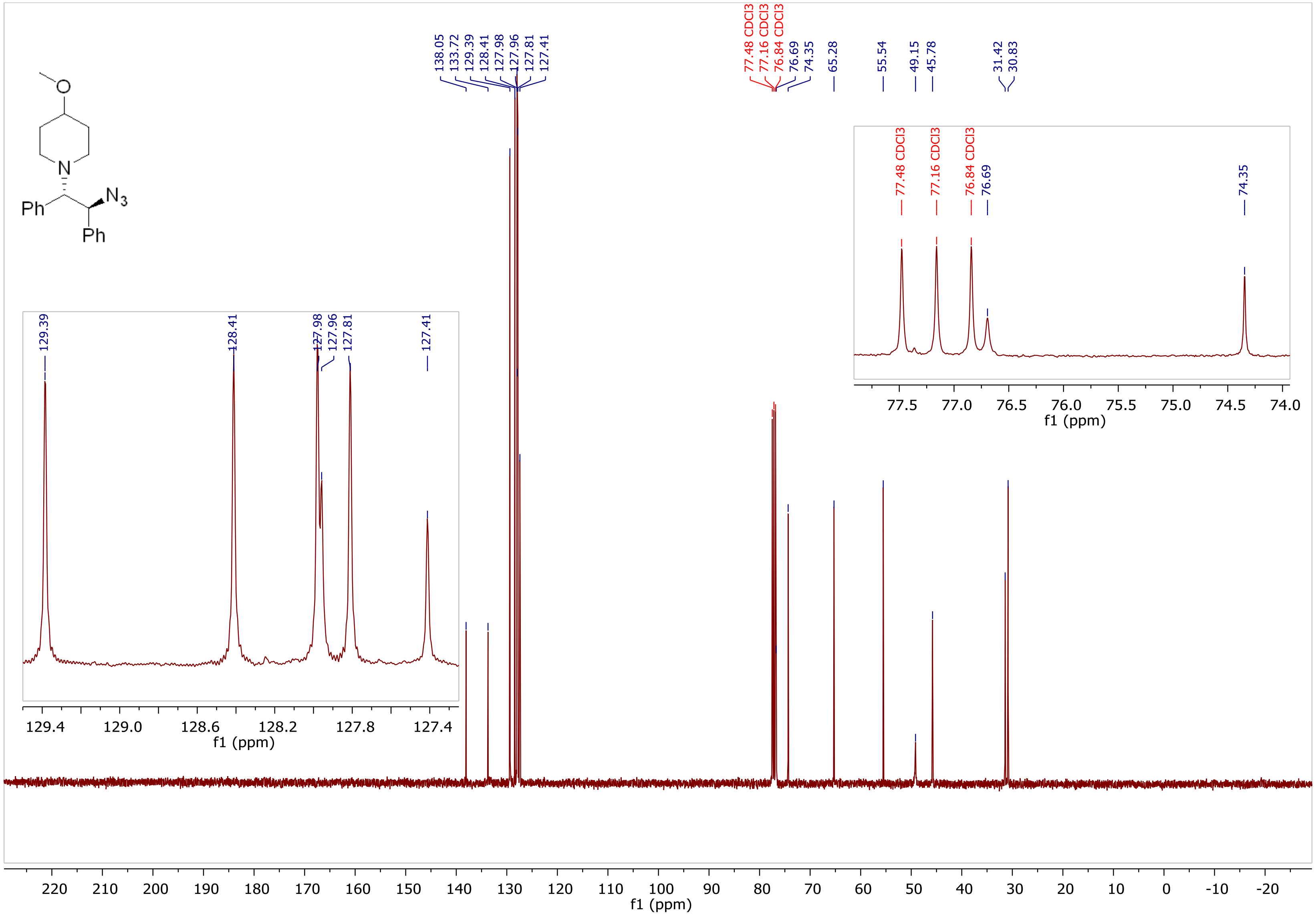

1-((1*S*,2*S*)-2-azido-1,2-diphenylethyl)-4,4-difluoropiperidine (3c)

^{1}H NMR (400 MHz, $CDCl_3$)

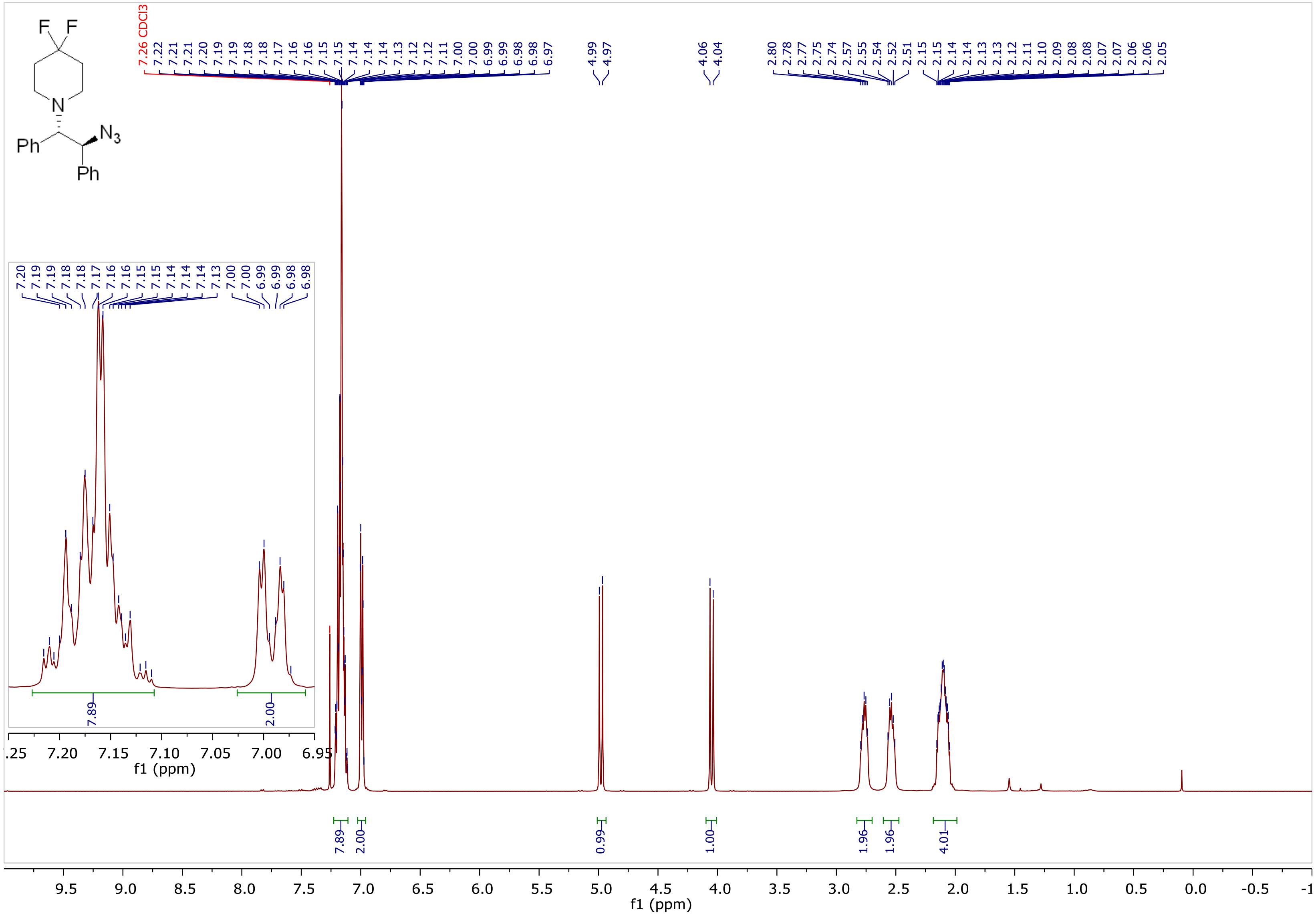

^{19}F NMR (377 MHz, $CDCl_3$)

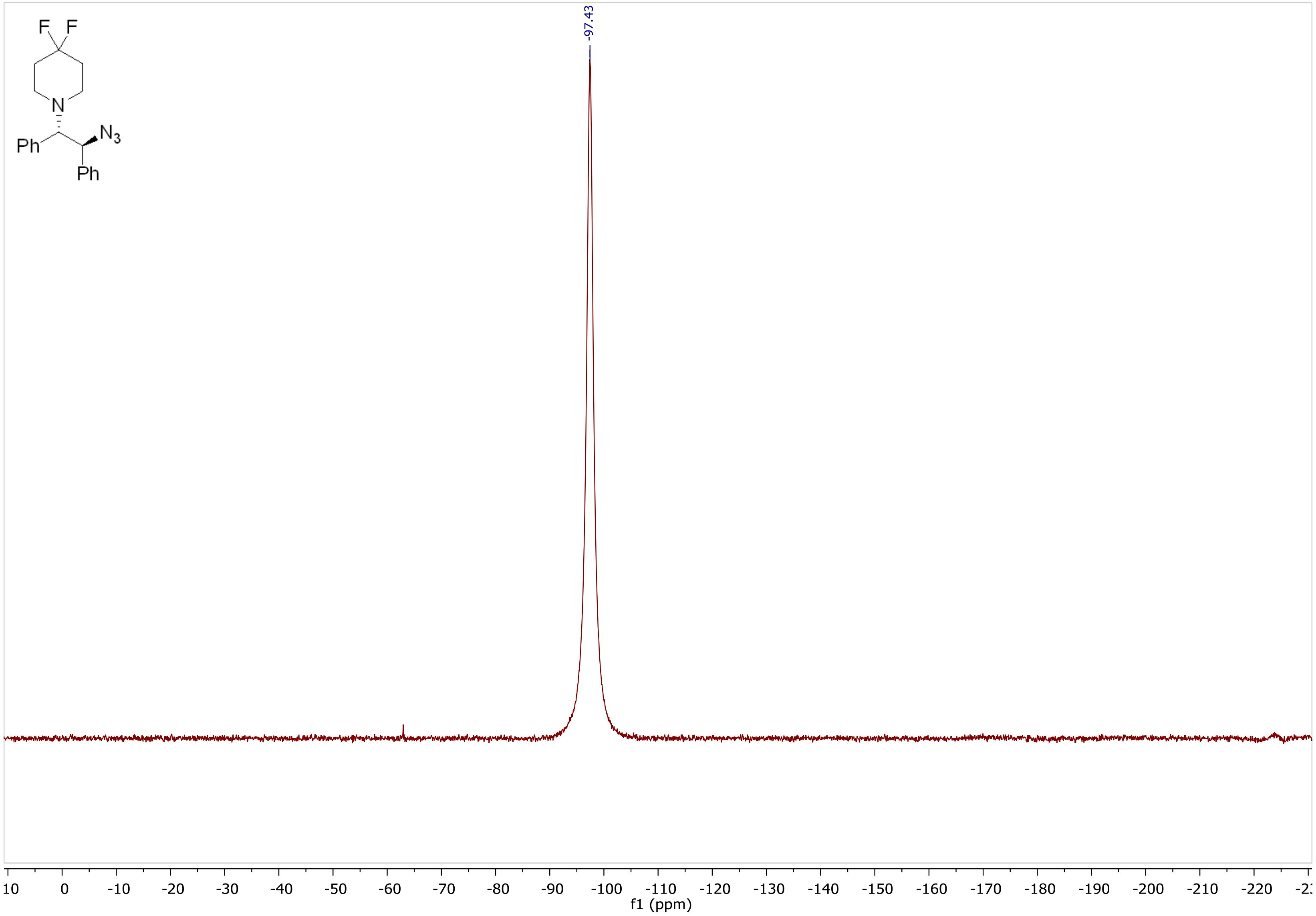

^{13}C NMR (101 MHz, $CDCl_3$)

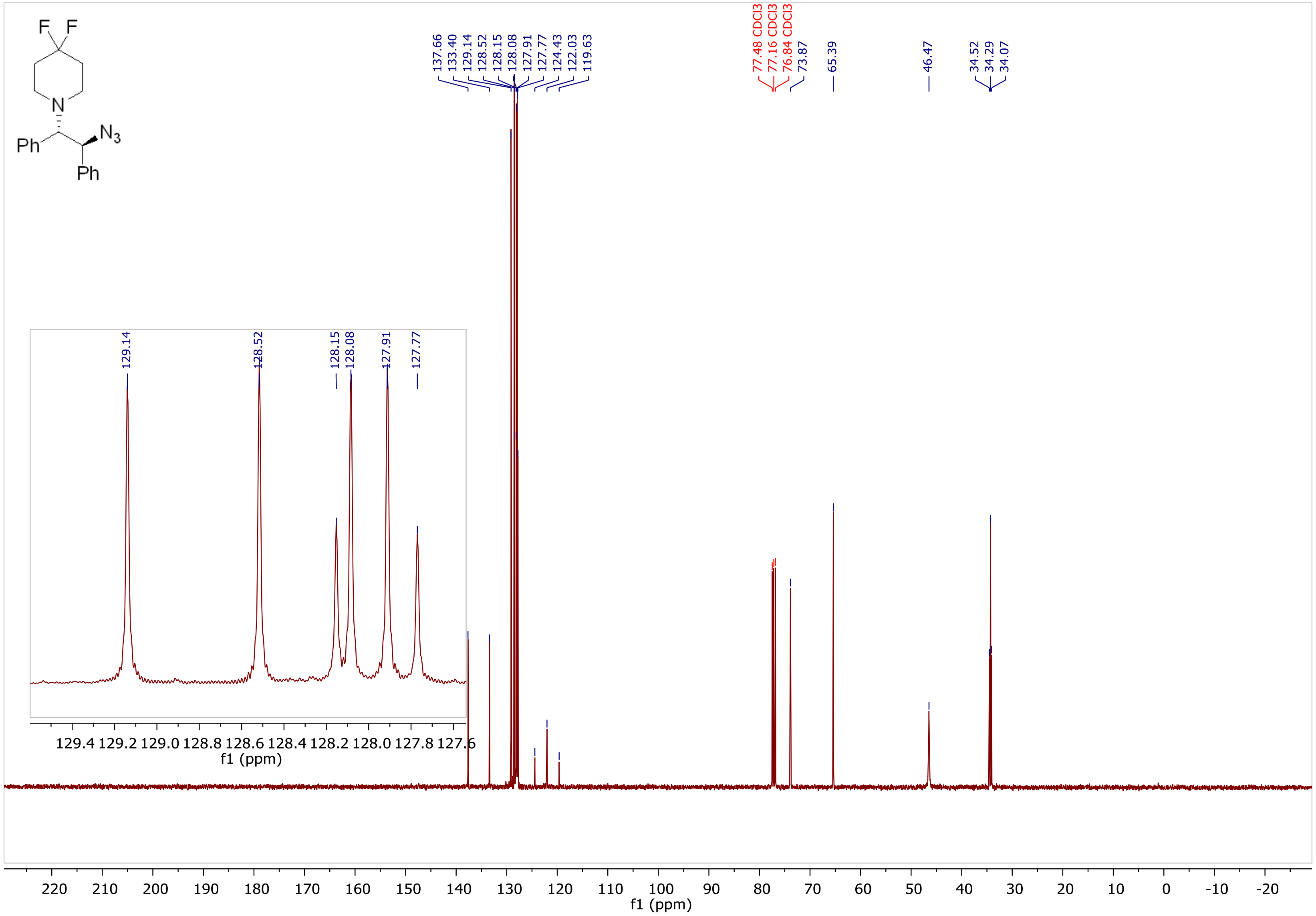

1-((1*S*,2*S*)-2-azido-1,2-diphenylethyl)pyrrolidine (3d)

^{1}H NMR (400 MHz, $CDCl_3$)

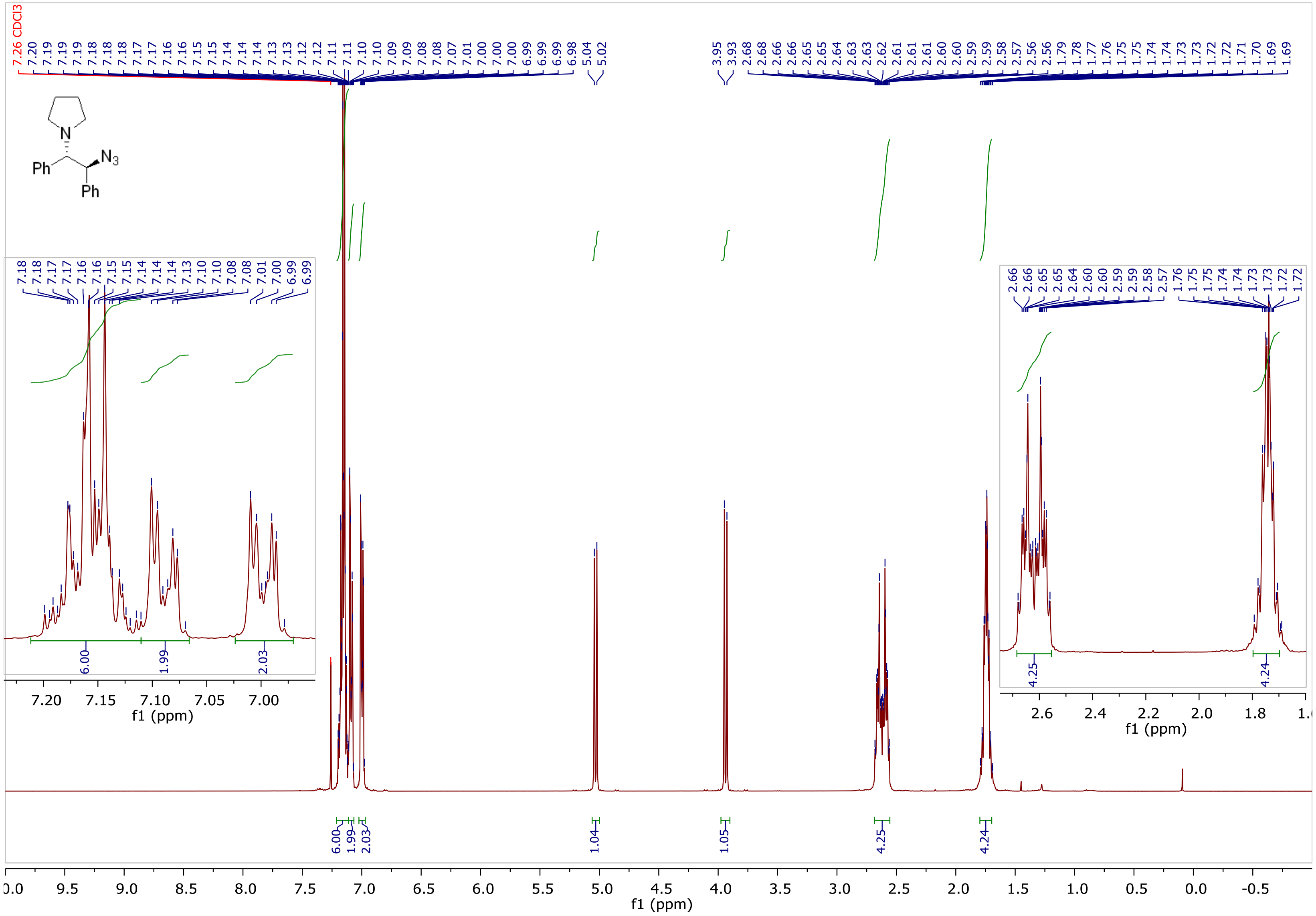

^{13}C NMR (101 MHz, $CDCl_3$)

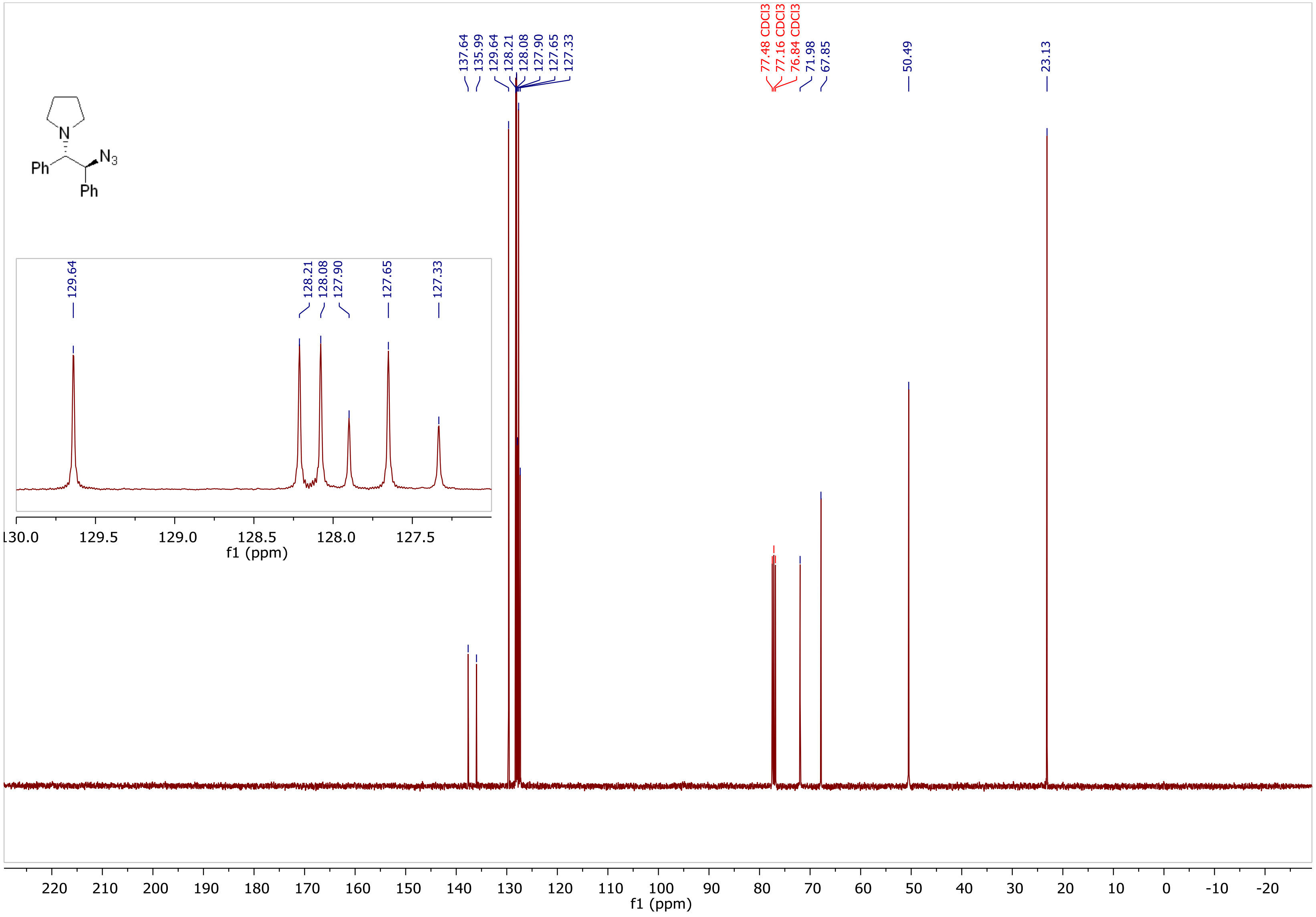

4-((1*S*,2*S*)-2-azido-1,2-diphenylethyl)morpholine (3e)

^{1}H NMR (400 MHz, $CDCl_3$)

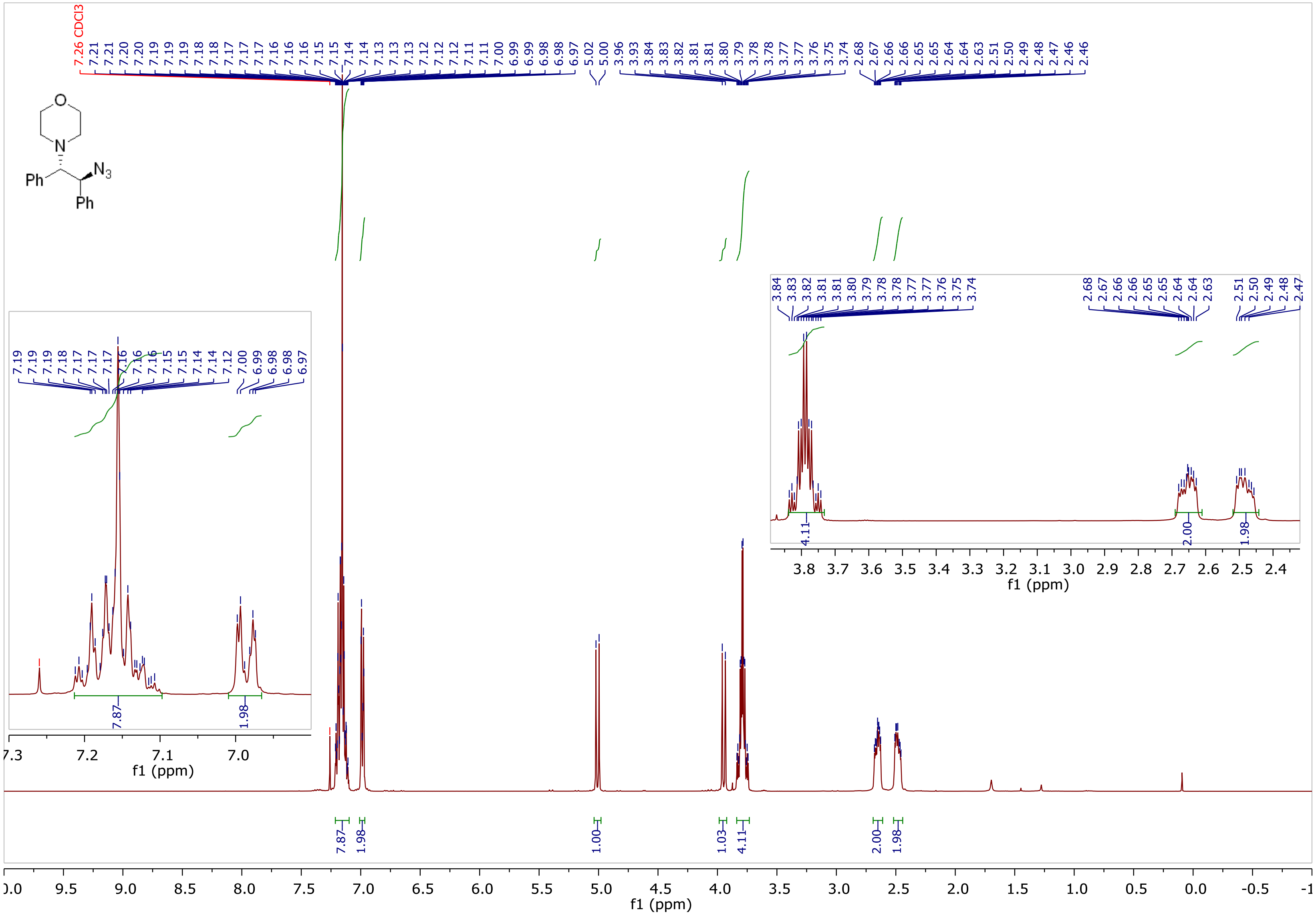

^{13}C NMR (101 MHz, $CDCl_3$)

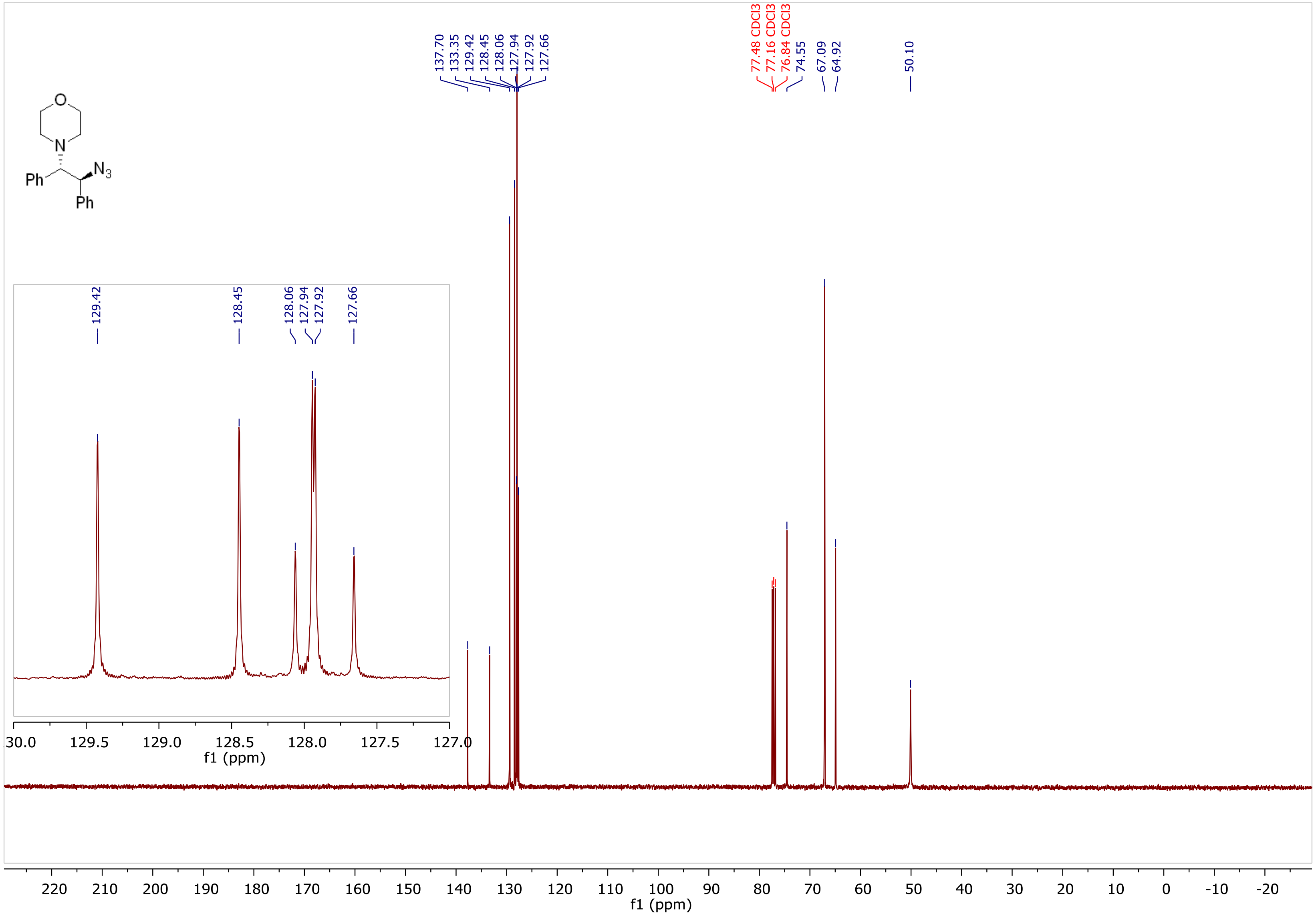

1-((1*S*,2*S*)-2-azido-1,2-diphenylethyl)-4-cyclohexylpiperazine (3f)

^{1}H NMR (500 MHz, $CDCl_3$)

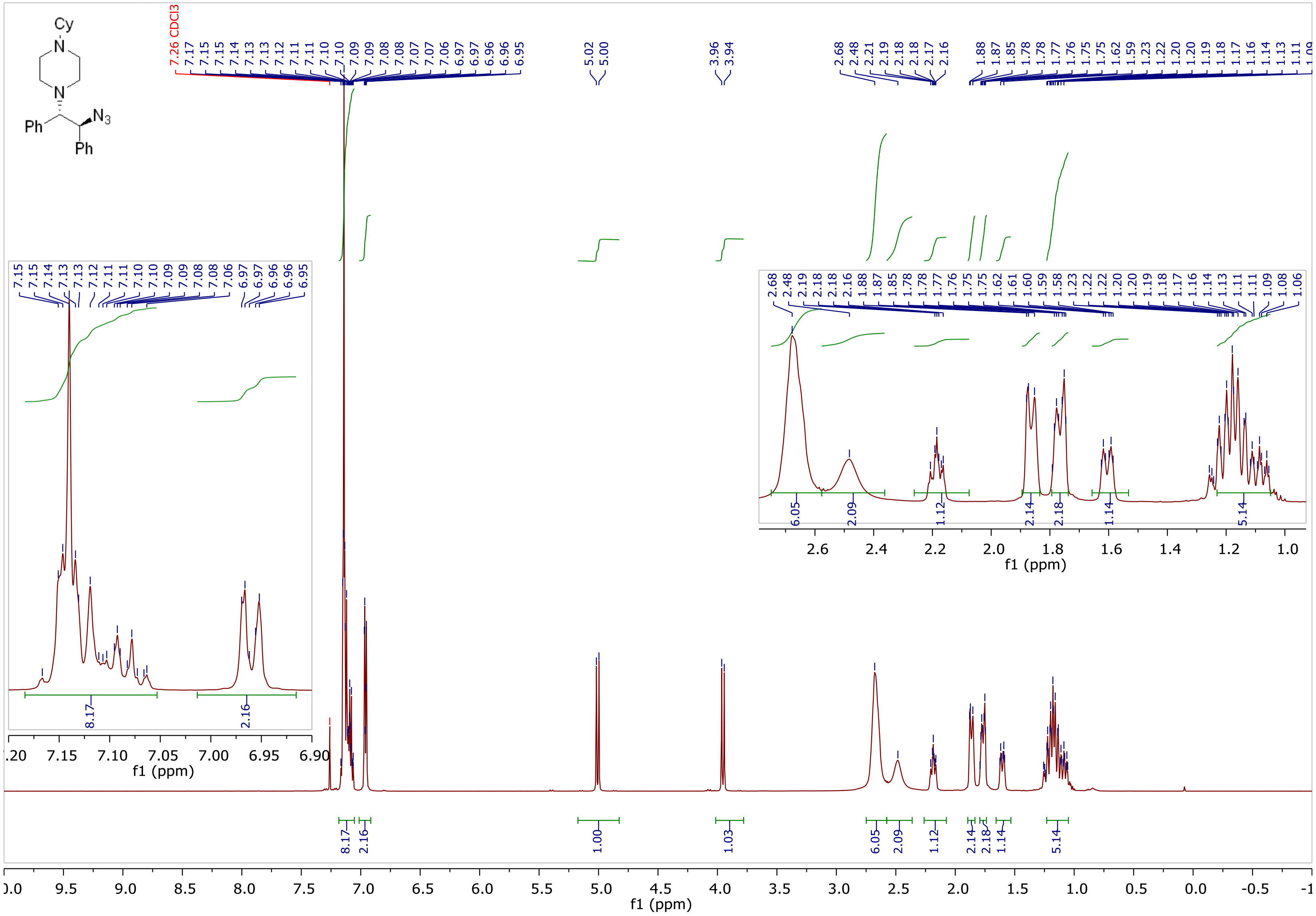

^{13}C NMR (126 MHz, $CDCl_3$)

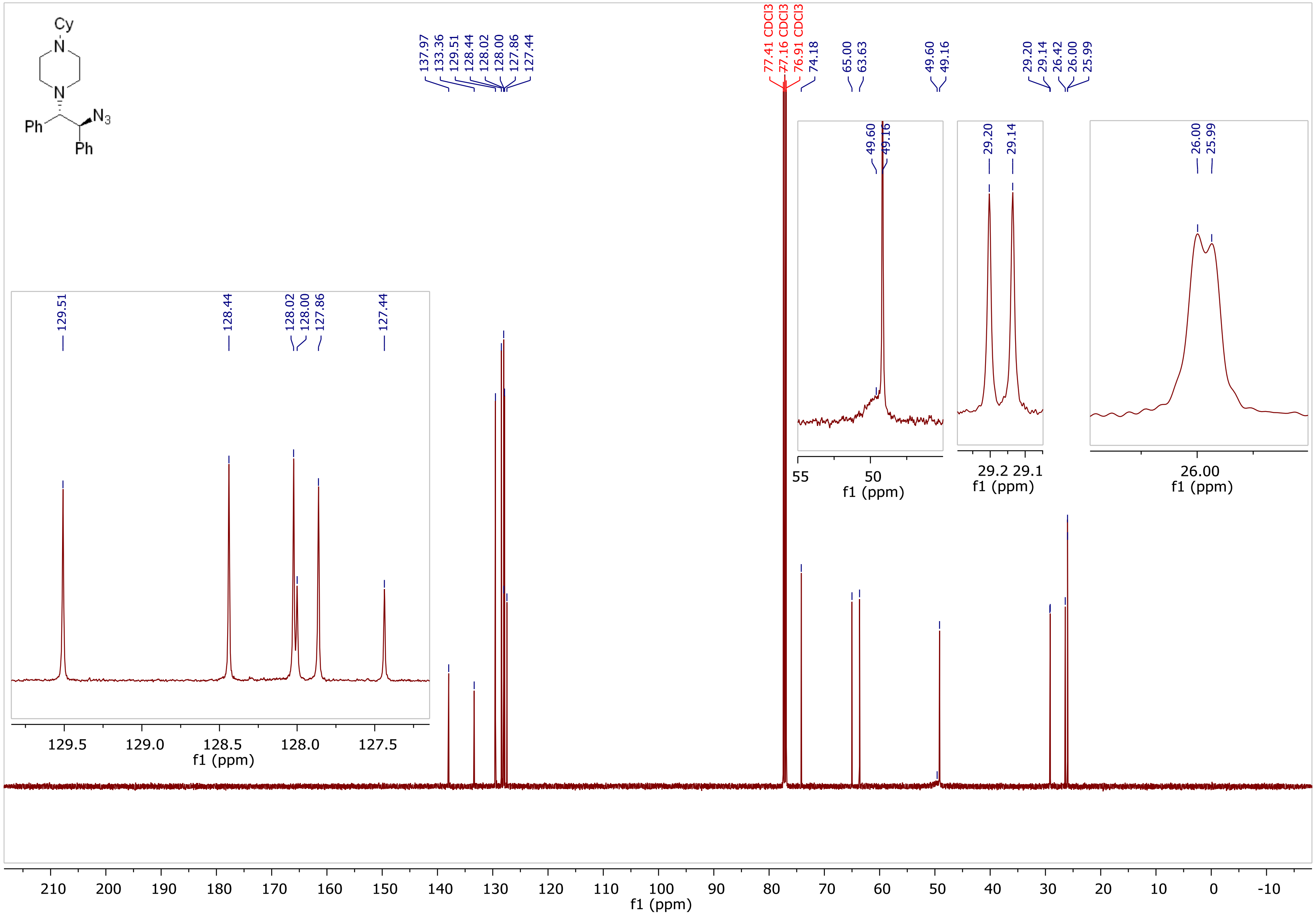

1-((1*S*,2*S*)-2-azido-1,2-diphenylethyl)-4-phenylpiperazine (3g)

^{1}H NMR (400 MHz, $CDCl_3$)

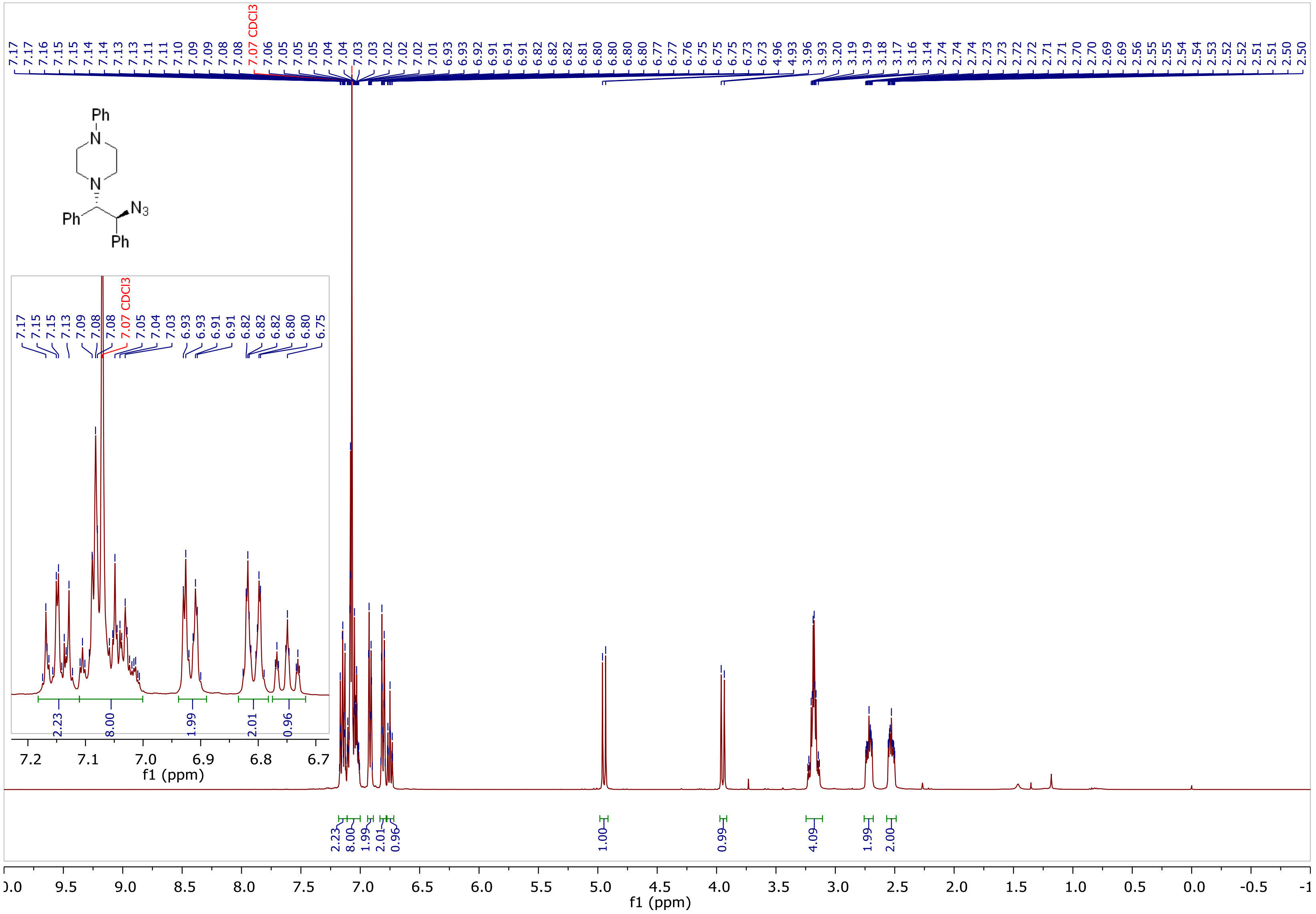

^{13}C NMR (101 MHz, $CDCl_3$)

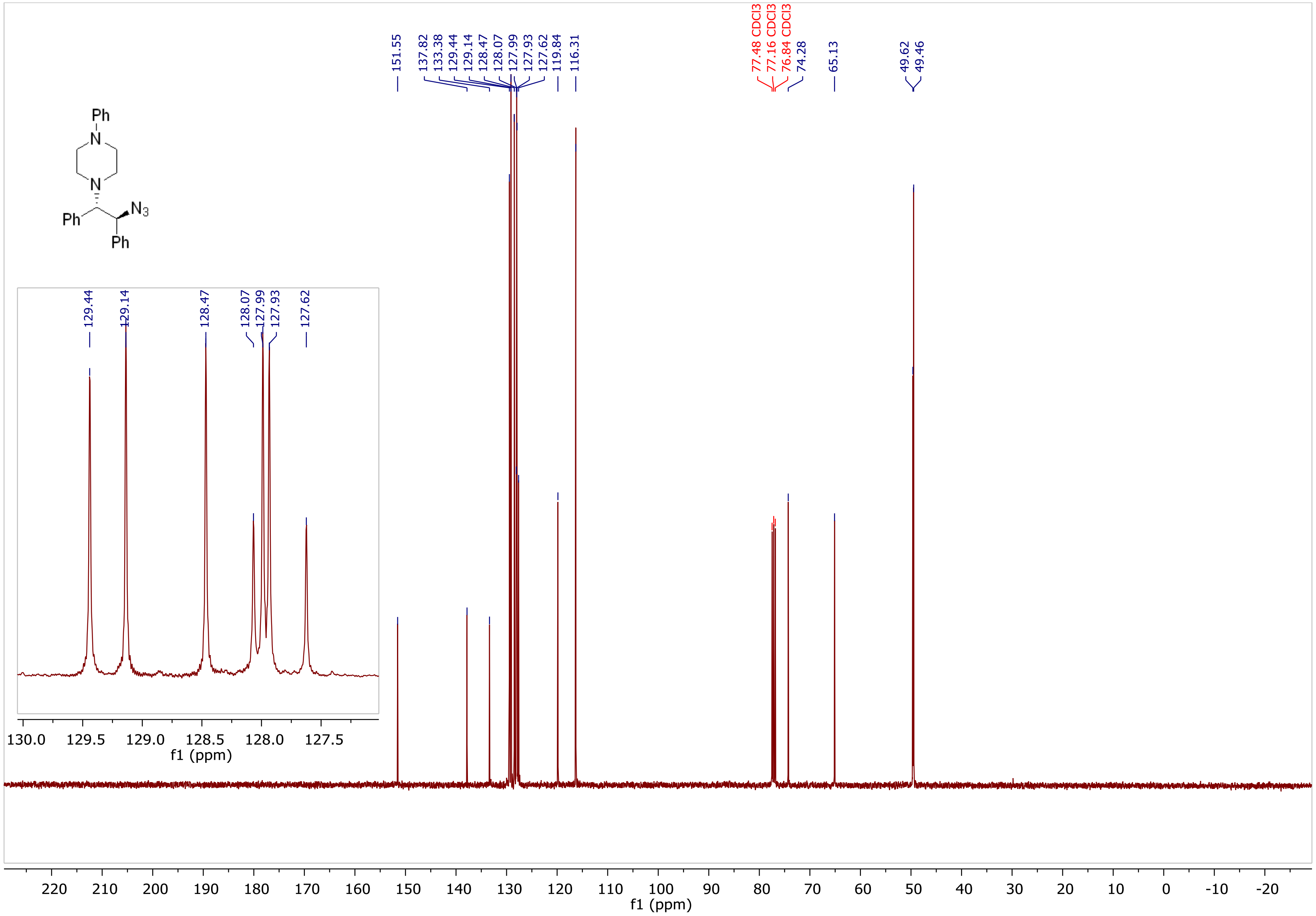

2-((1*S*,2*S*)-2-azido-1,2-diphenylethyl)-1,2,3,4-tetrahydroisoquinoline (3h)

^{1}H NMR (400 MHz, $CDCl_3$)

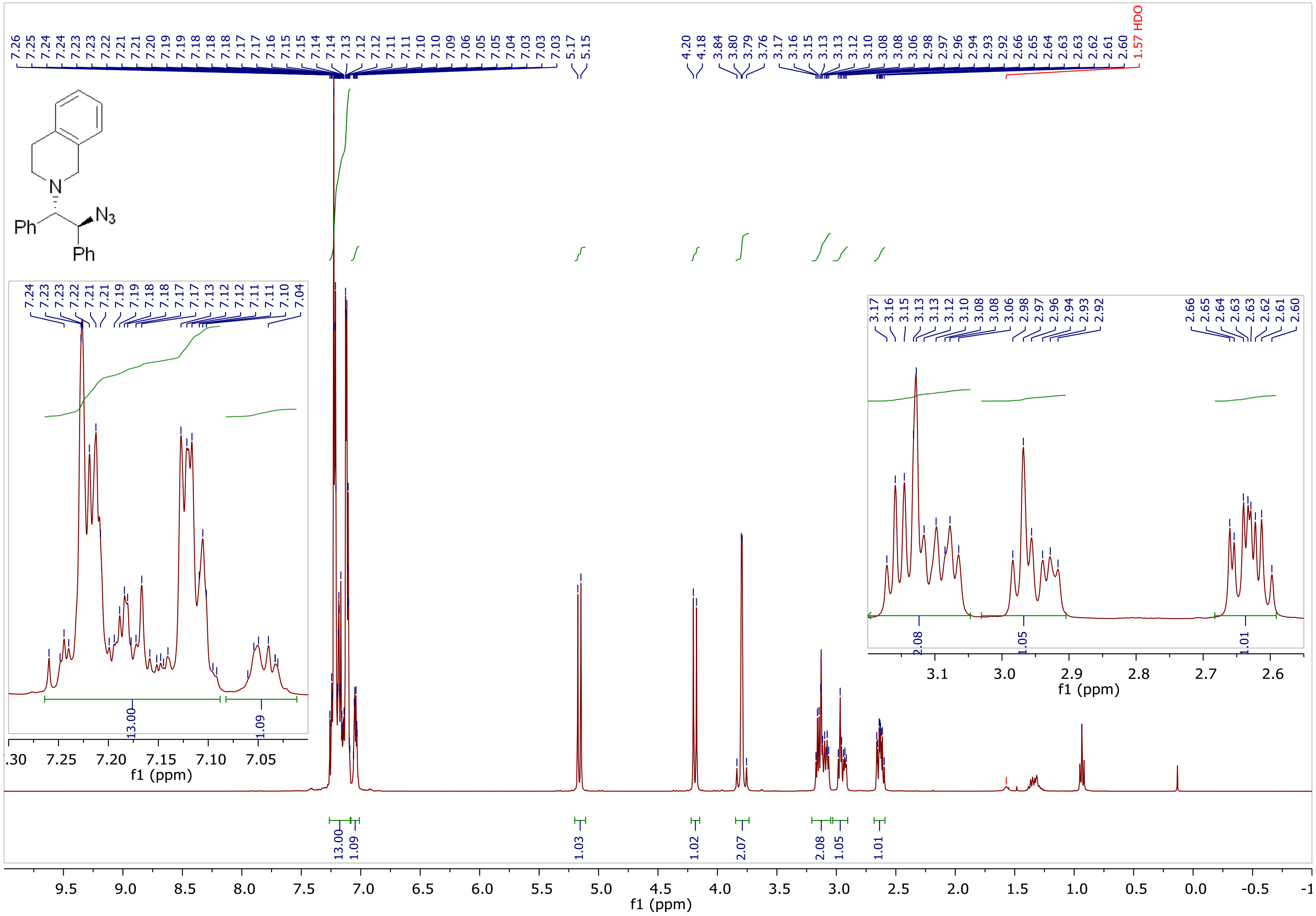

^{13}C NMR (101 MHz, $CDCl_3$)

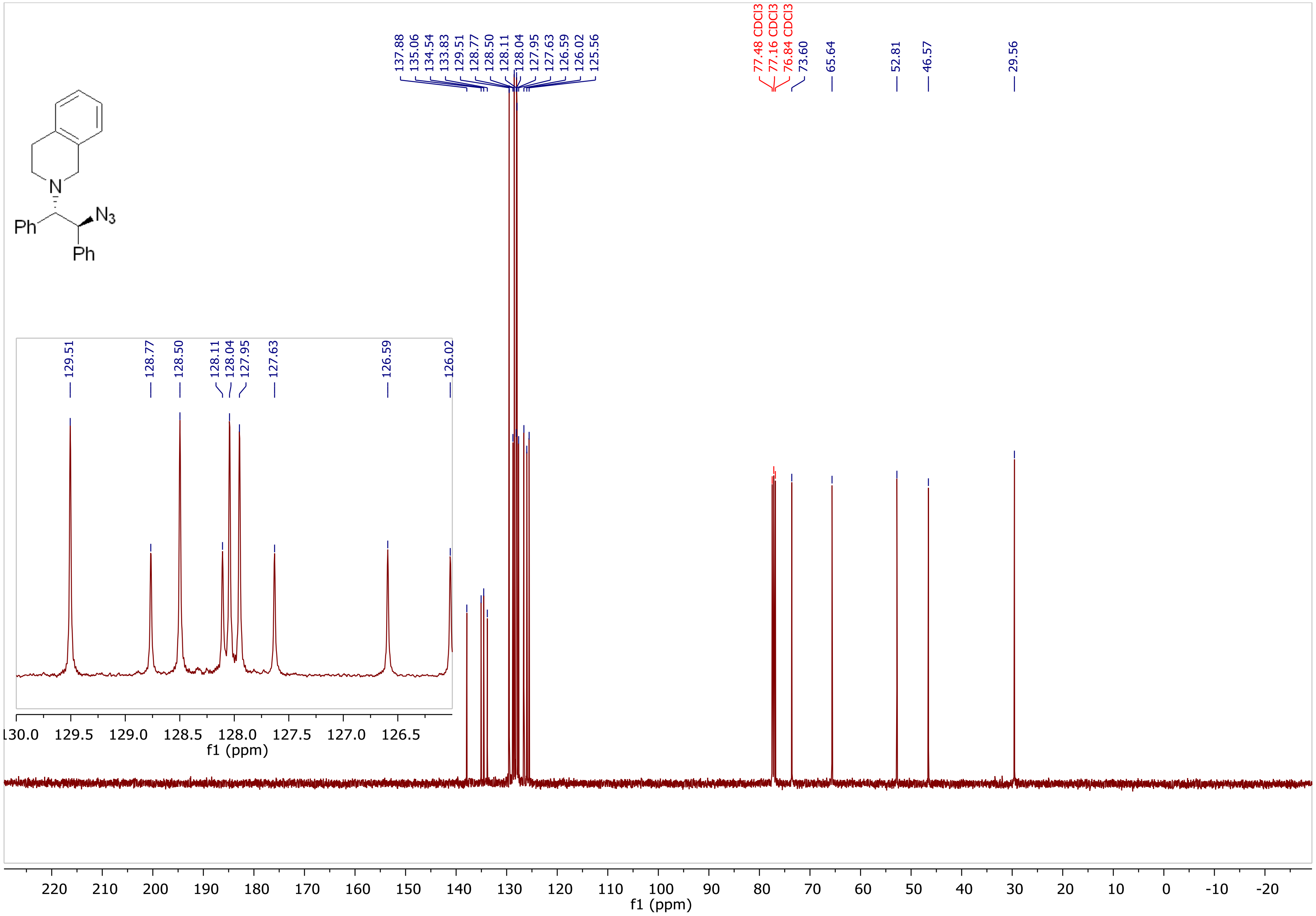

(1*S*,2*S*)-2-azido-*N*-benzyl-*N*-methyl-1,2-diphenylethan-1-amine (3i)

^{1}H NMR (500 MHz, $CDCl_3$)

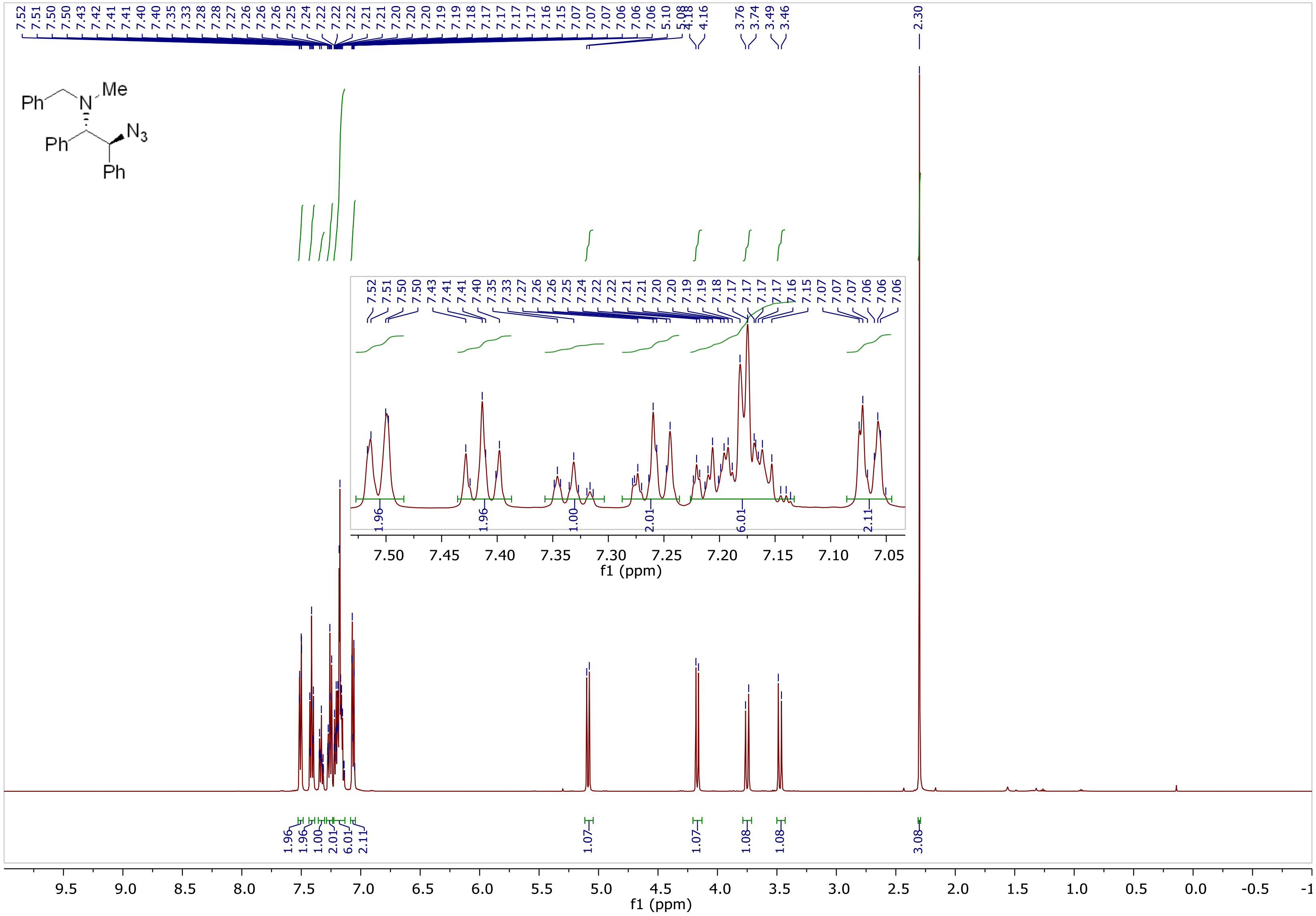

^{13}C NMR (126 MHz, $CDCl_3$)

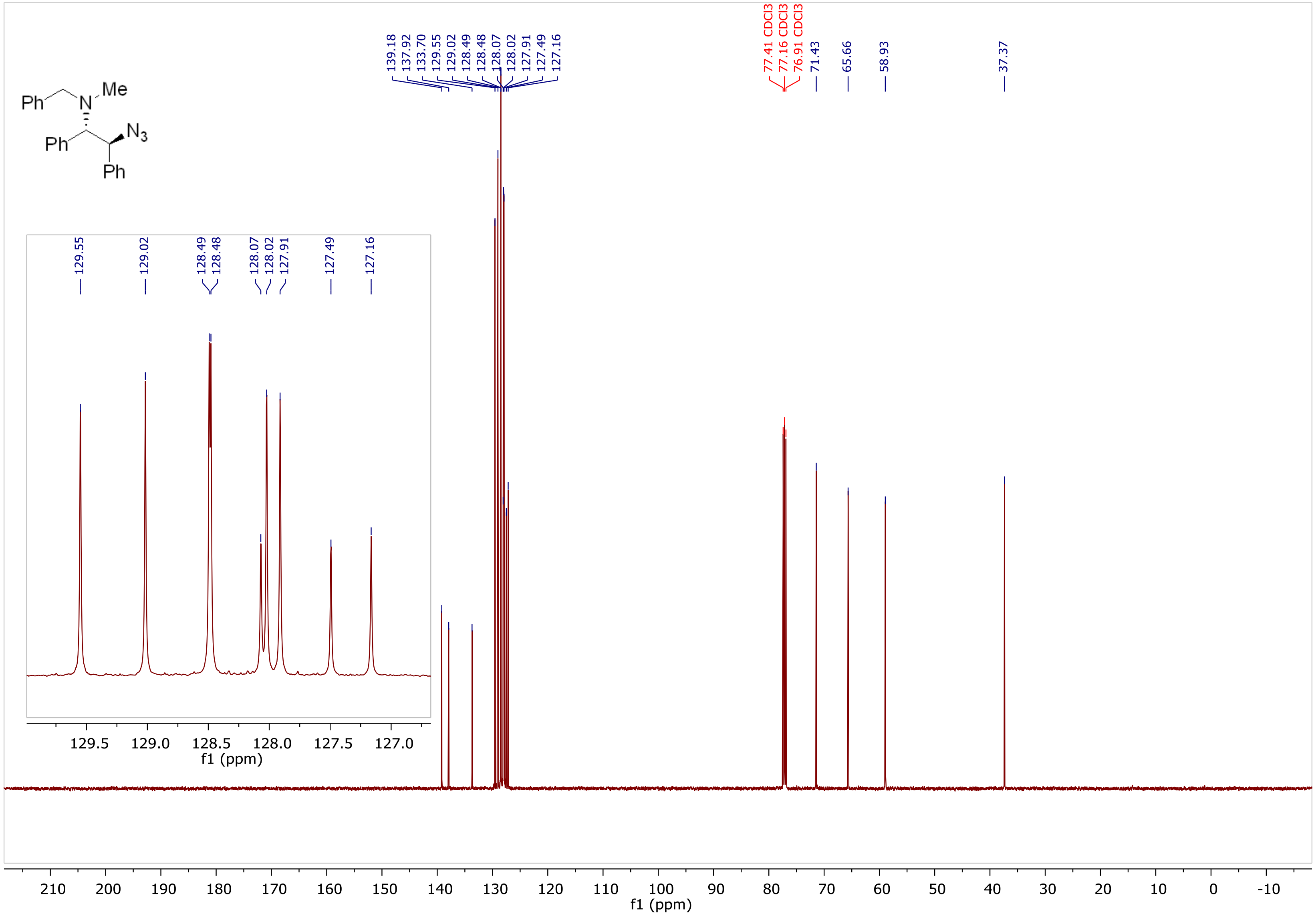

(1*S*,2*S*)-2-azido-*N*,*N*-dimethyl-1,2-diphenylethan-1-amine (3j)

^{1}H NMR (500 MHz, $CDCl_3$)

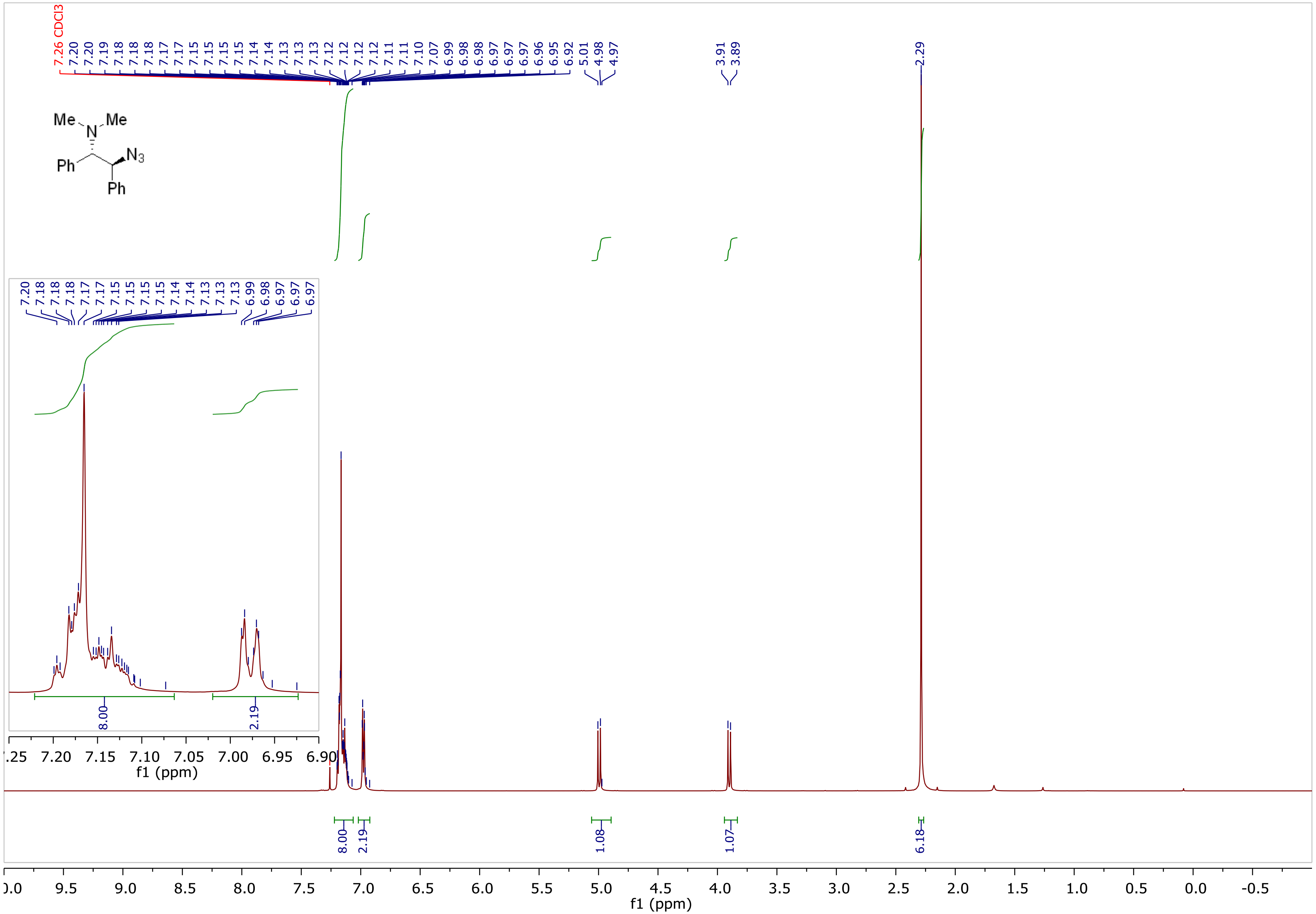

^{13}C NMR (126 MHz, $CDCl_3$)

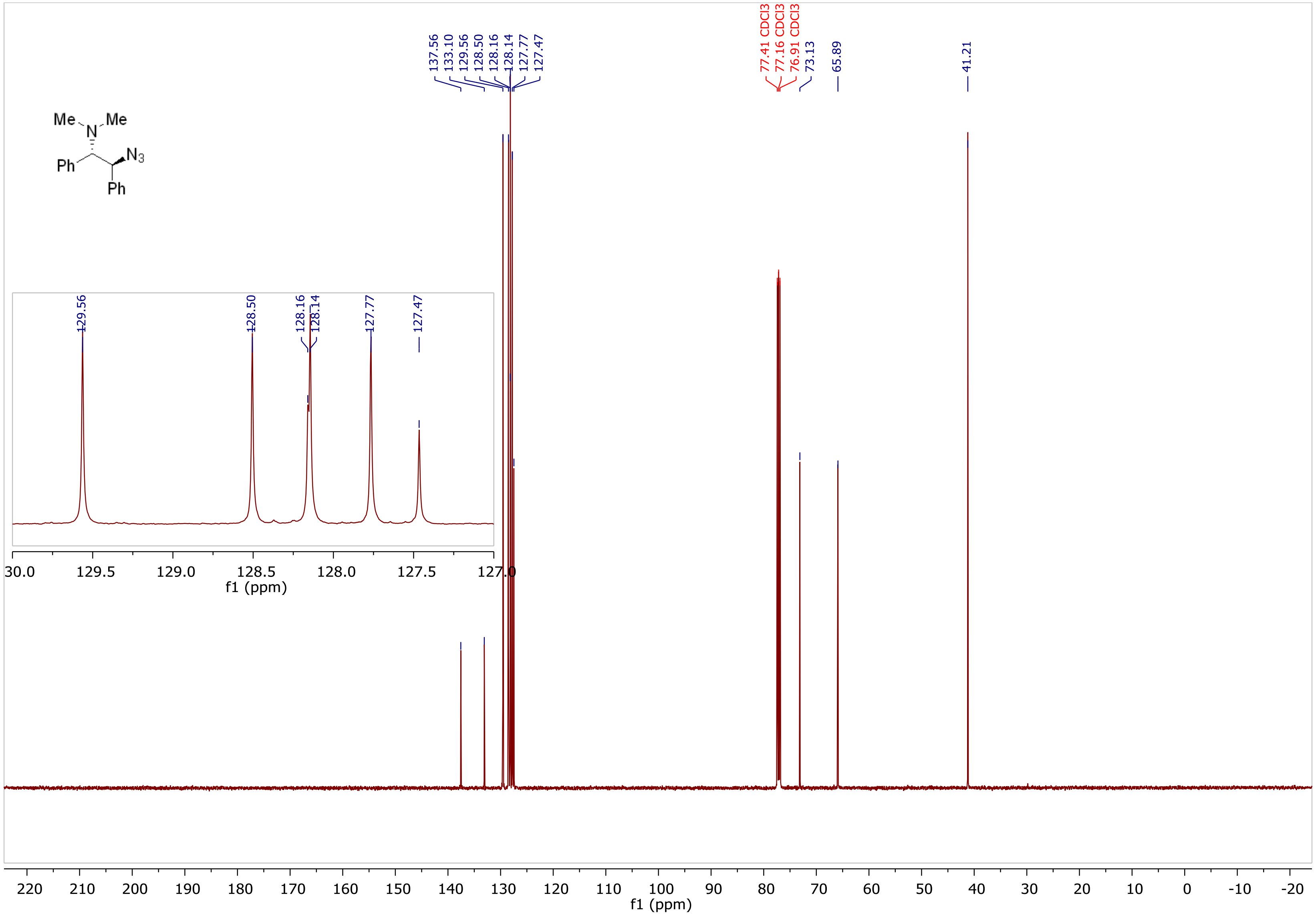

1-((1*S*,2*S*)-2-azido-1,2-diphenylethyl)azepane (3k)

^{1}H NMR (400 MHz, $CDCl_3$)

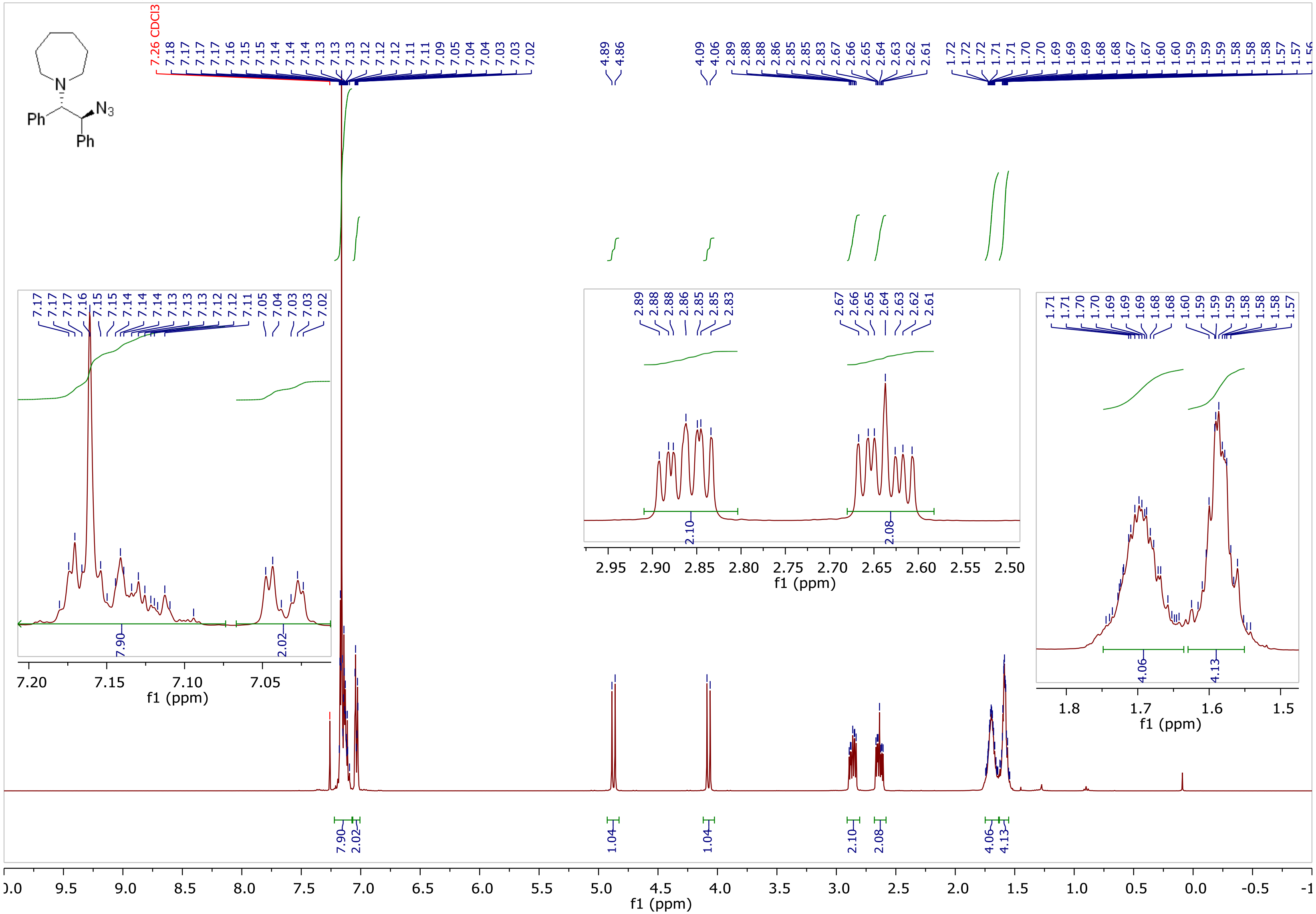

^{13}C NMR (101 MHz, $CDCl_3$)

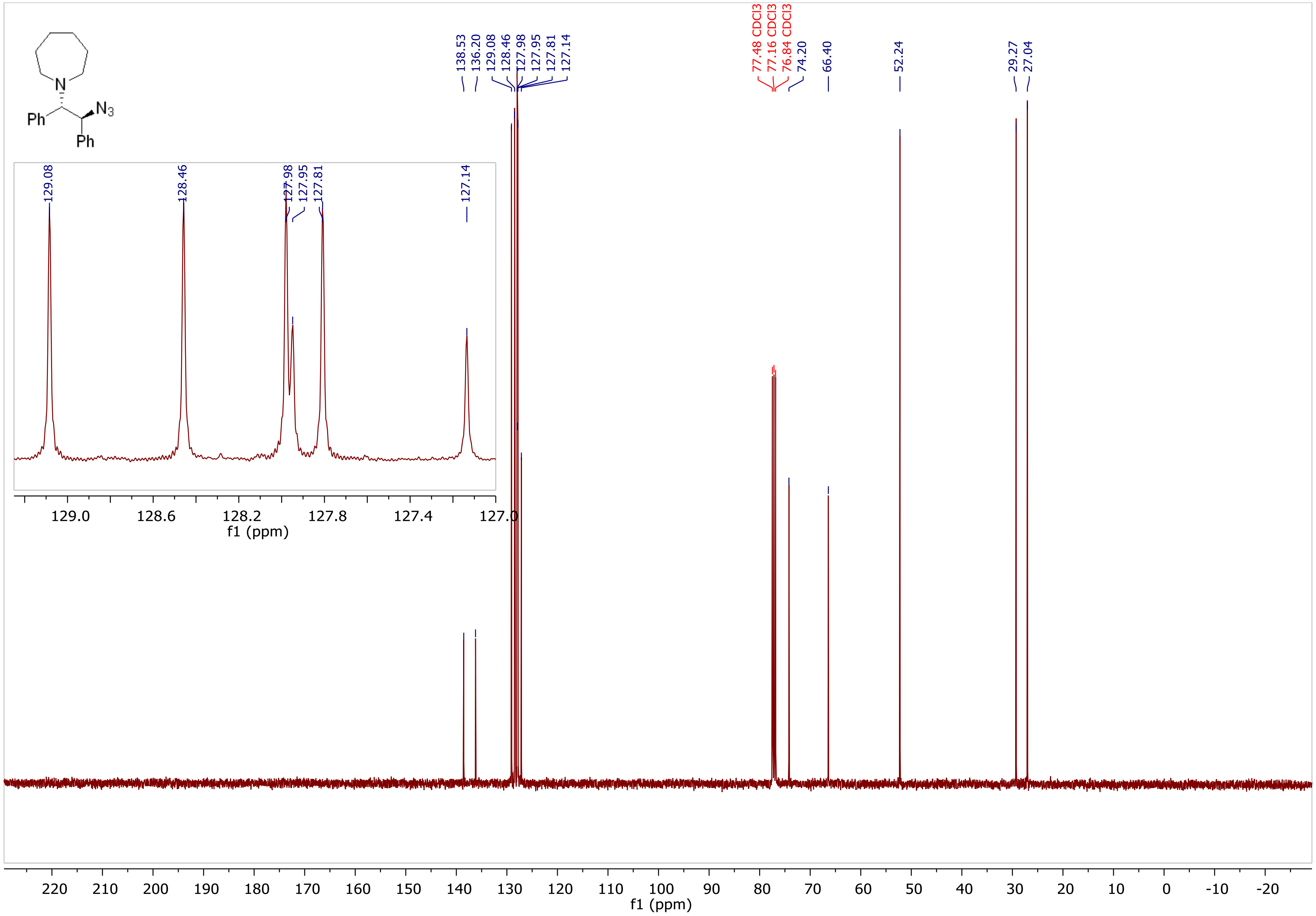

1-((1*S*,2*S*)-2-azido-1,2-bis(3-fluorophenyl)ethyl)piperidine (3l)

^{1}H NMR (500 MHz, $CDCl_3$)

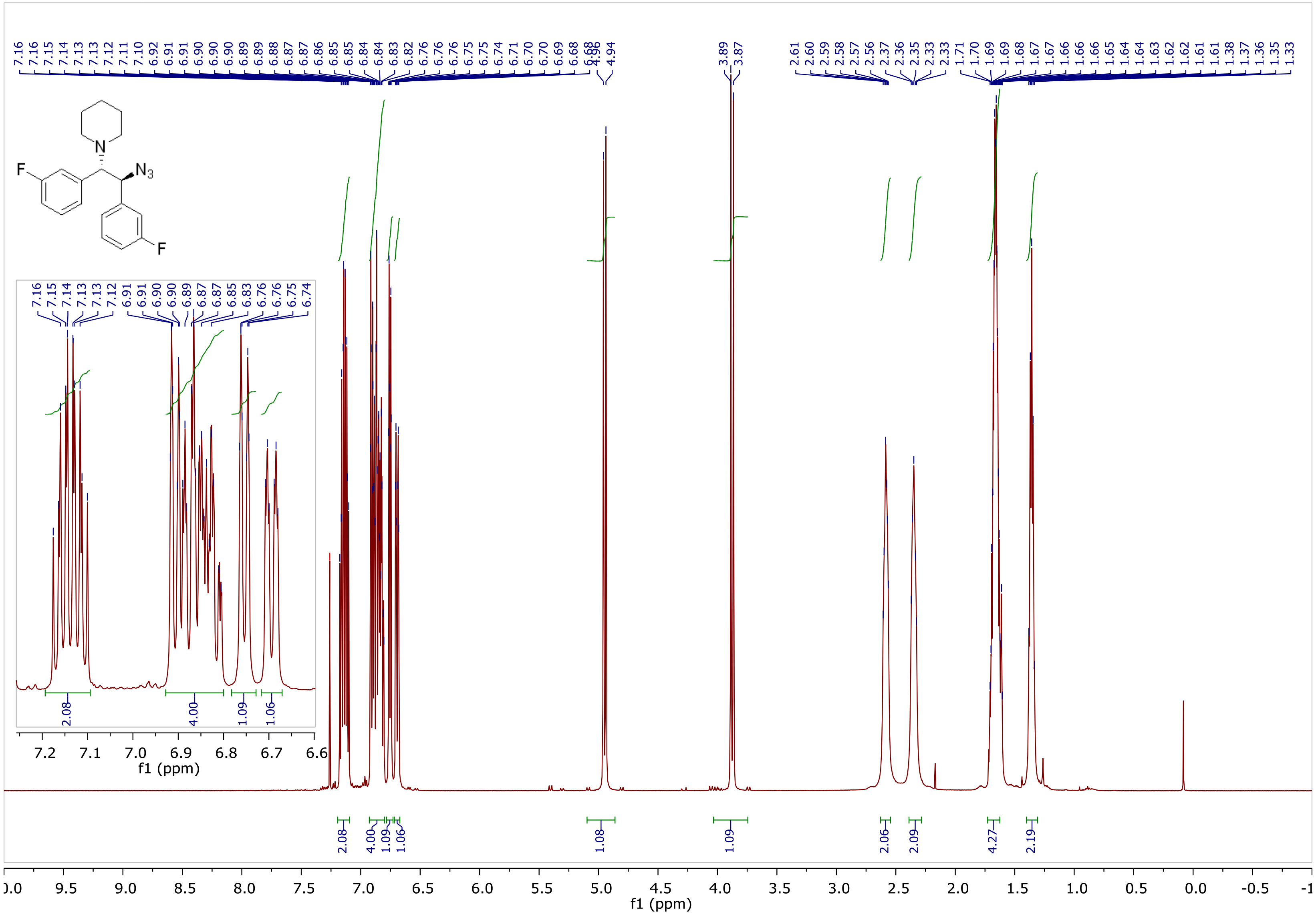

^{19}F NMR (471 MHz, $CDCl_3$)

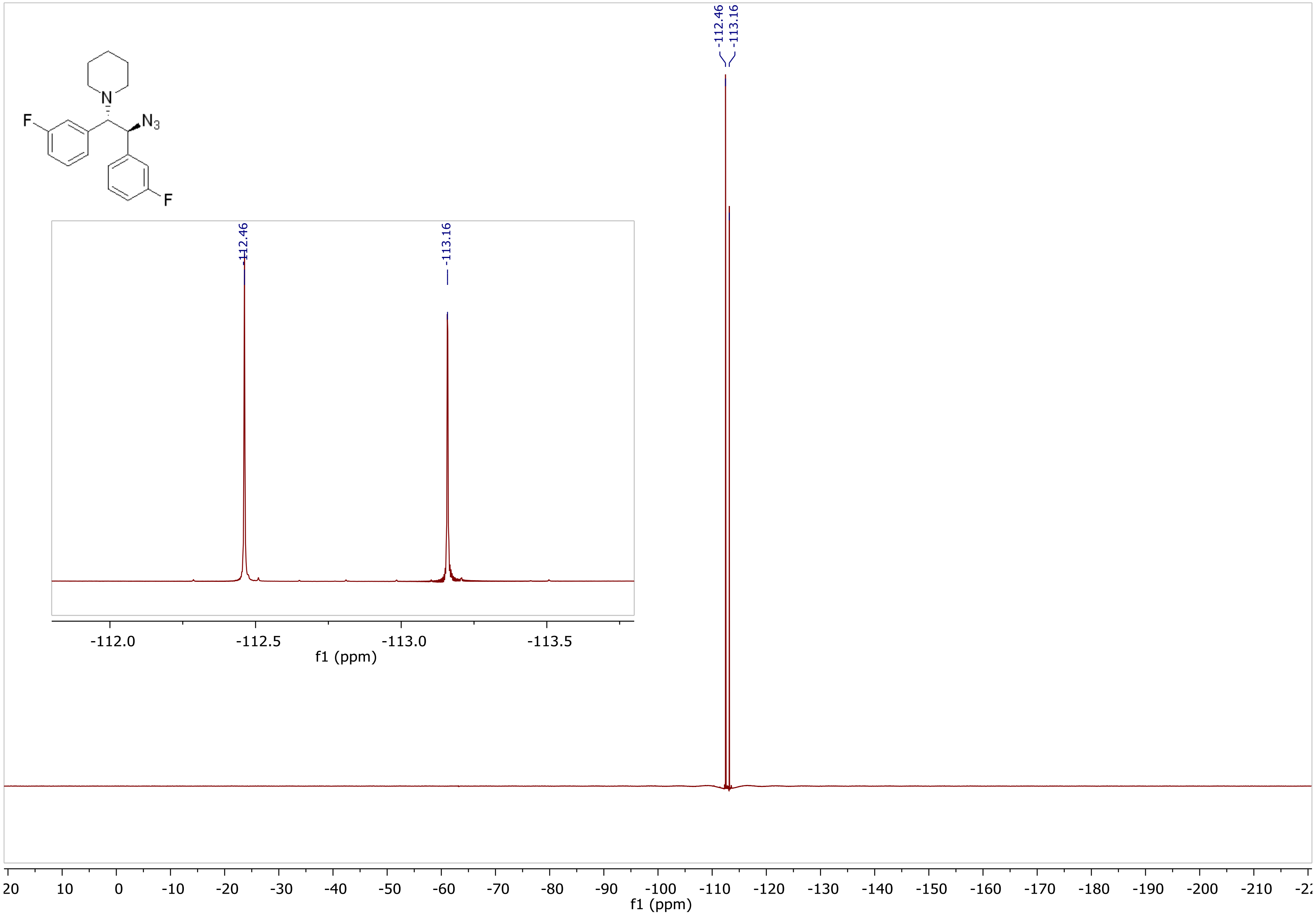

^{13}C NMR (126 MHz, $CDCl_3$)

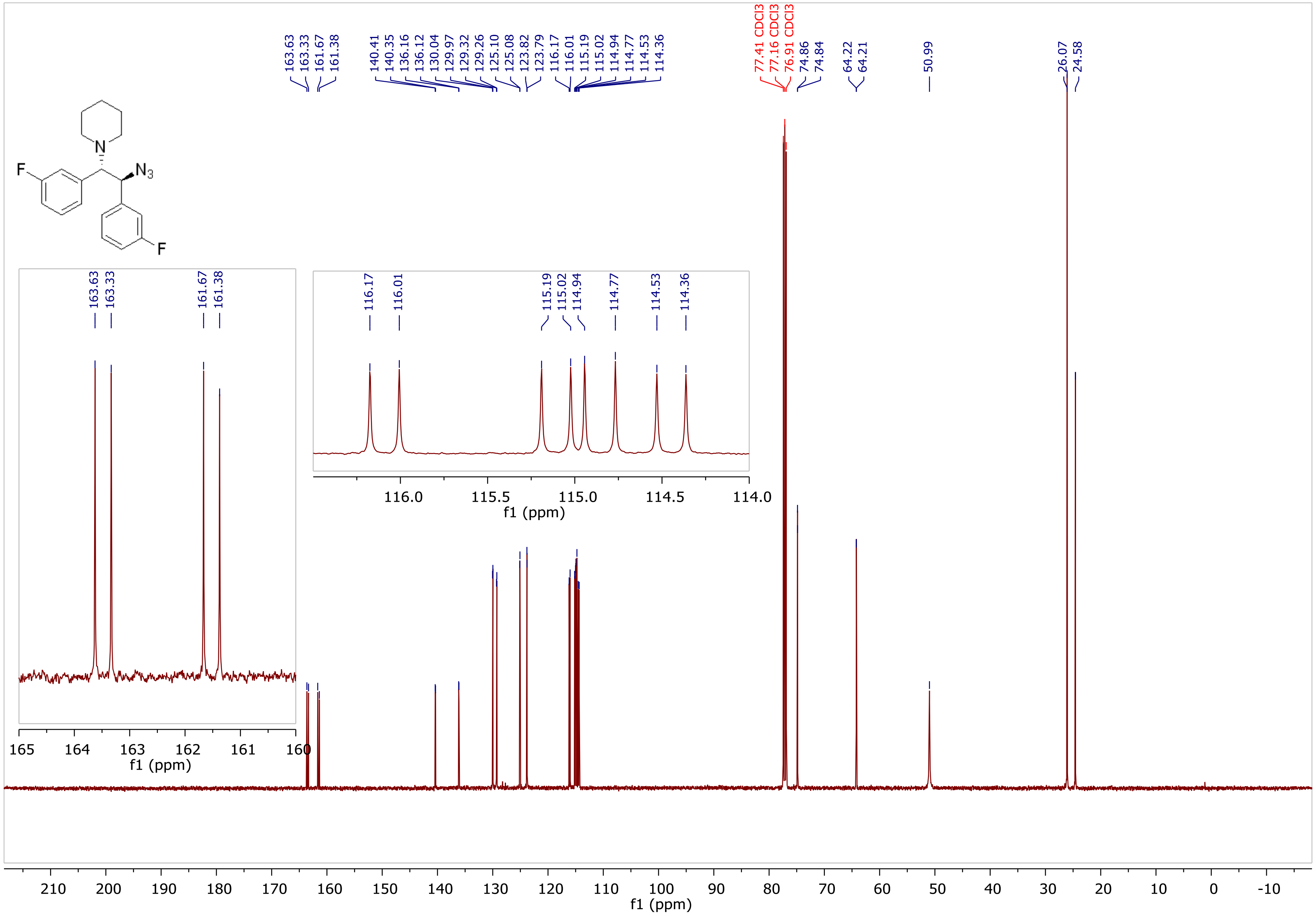

1-((1*S*,2*S*)-2-azido-1,2-bis(3-chlorophenyl)ethyl)piperidine (3m)

^{1}H NMR (400 MHz, $CDCl_3$)

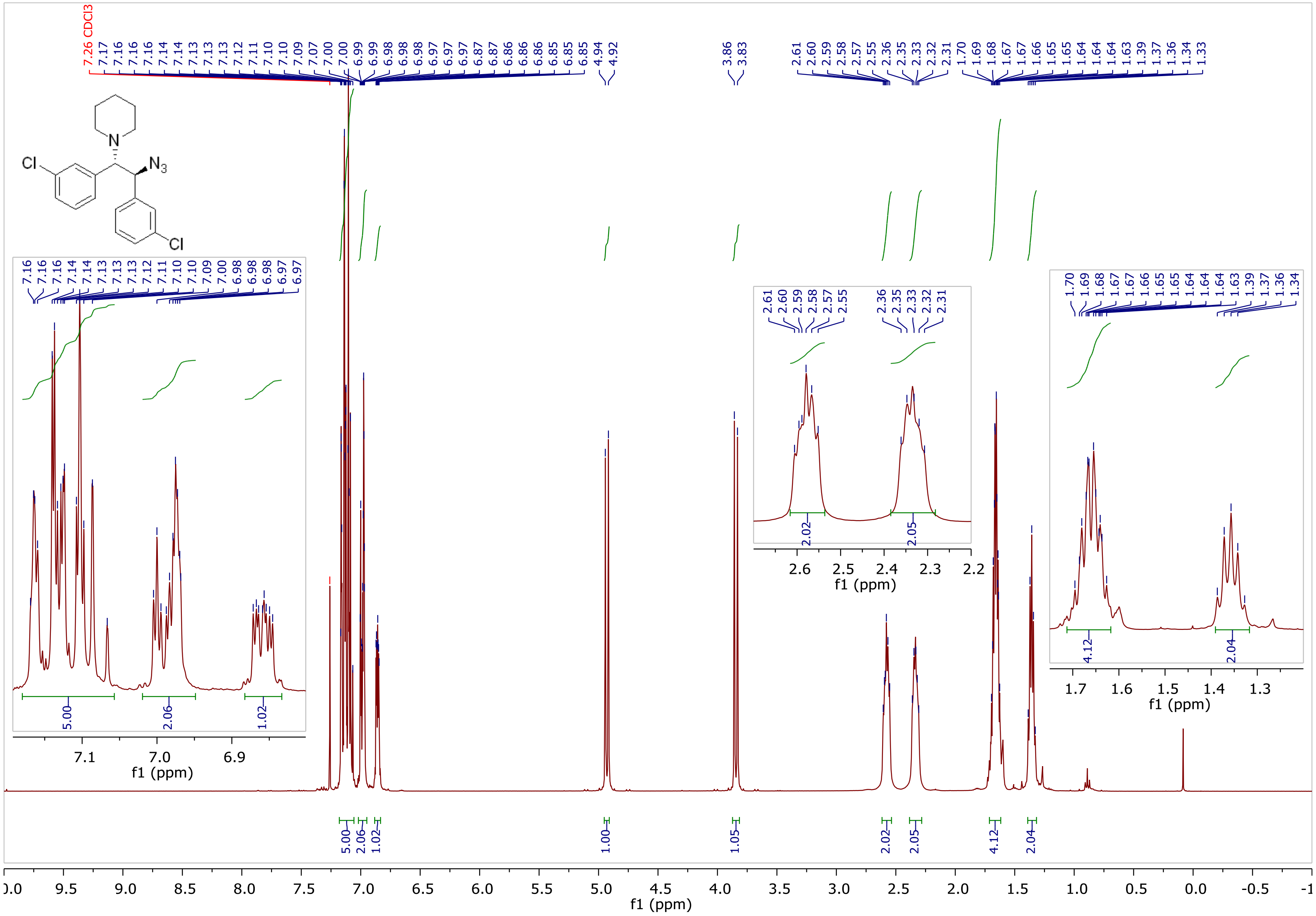

^{13}C NMR (101 MHz, $CDCl_3$)

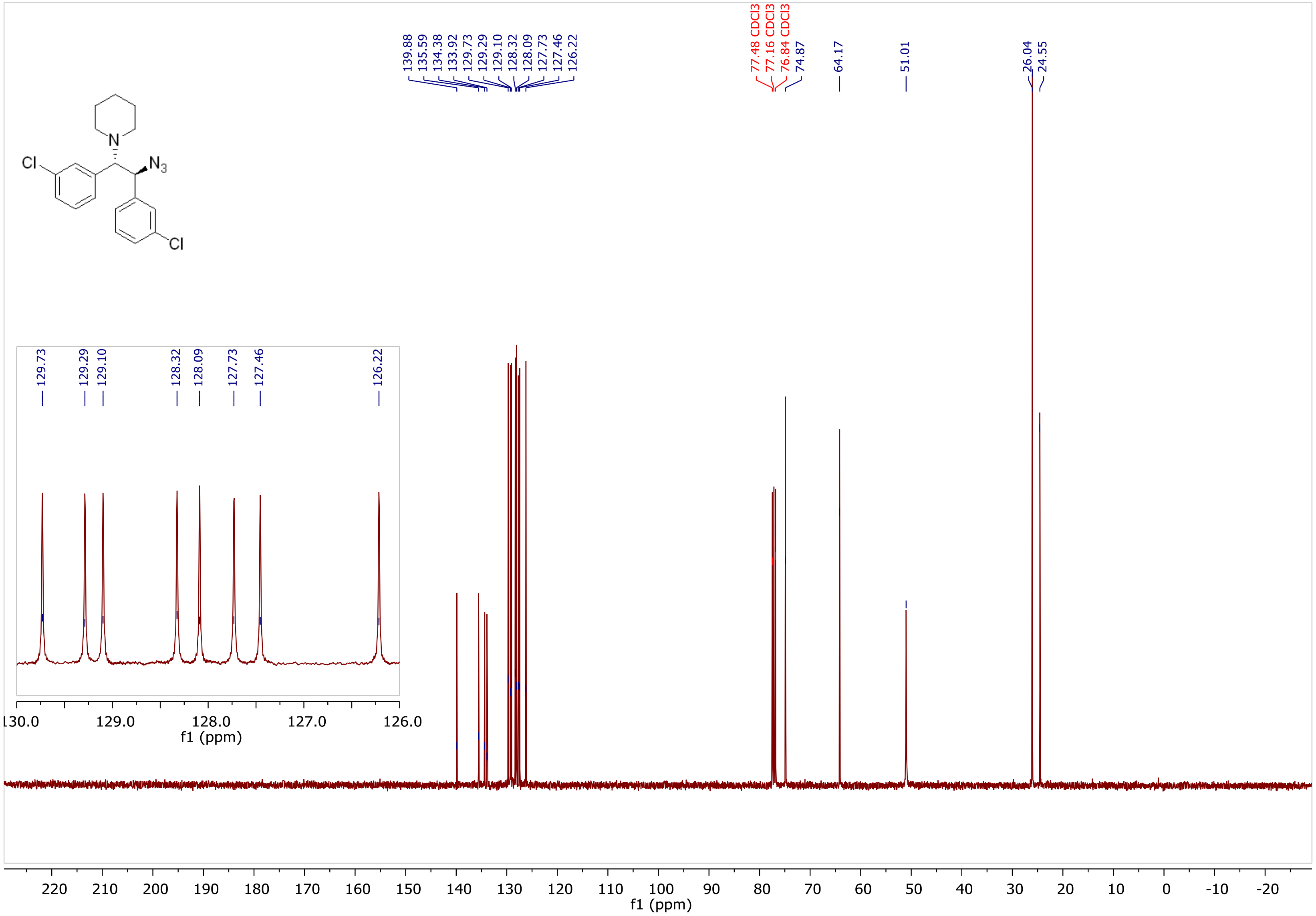

1-((1*S*,2*S*)-2-azido-1,2-bis(3-(trifluoromethyl)phenyl)ethyl)piperidine (3n)

^{1}H NMR (400 MHz, $CDCl_3$)

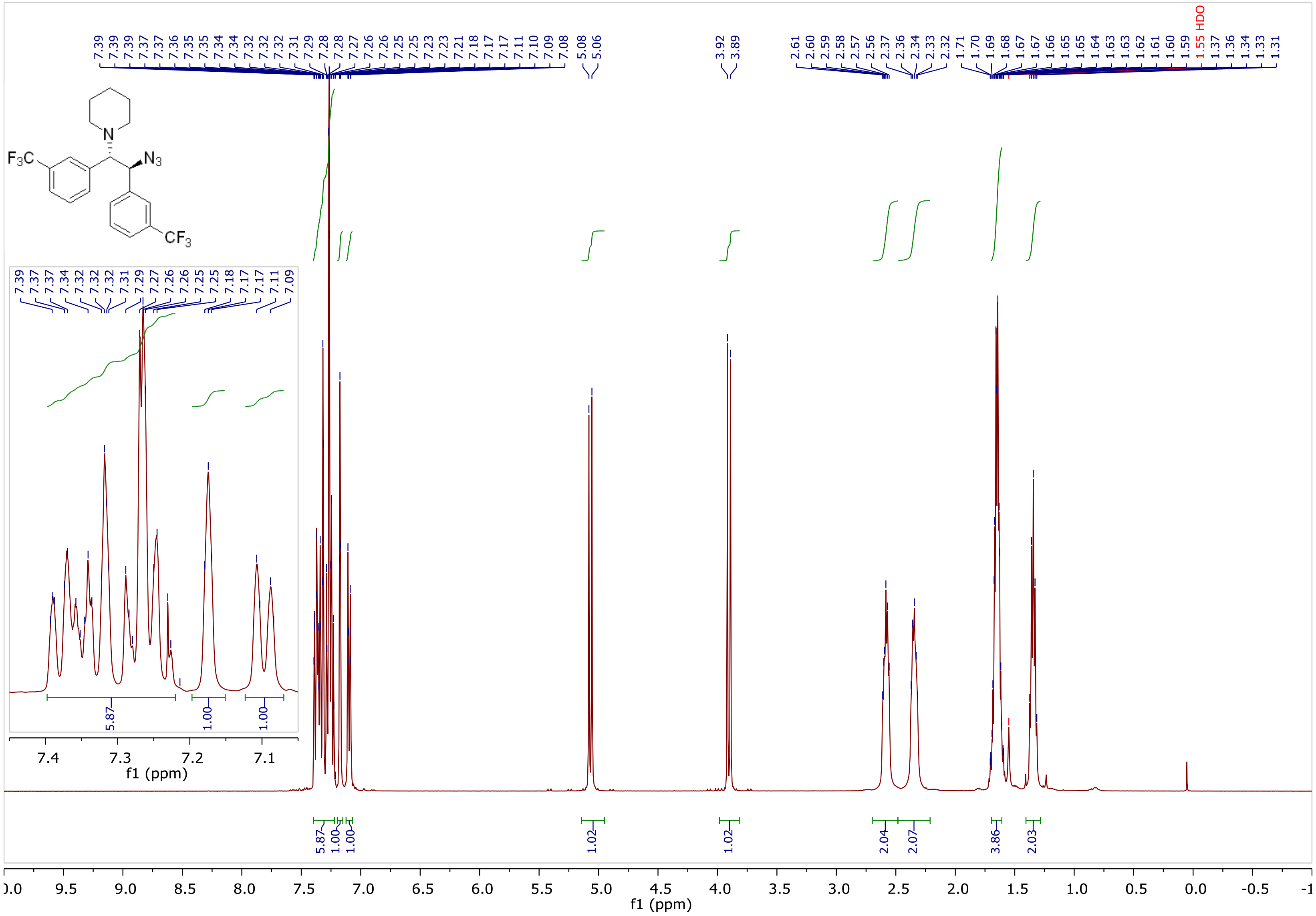

^{19}F NMR (377 MHz, $CDCl_3$)

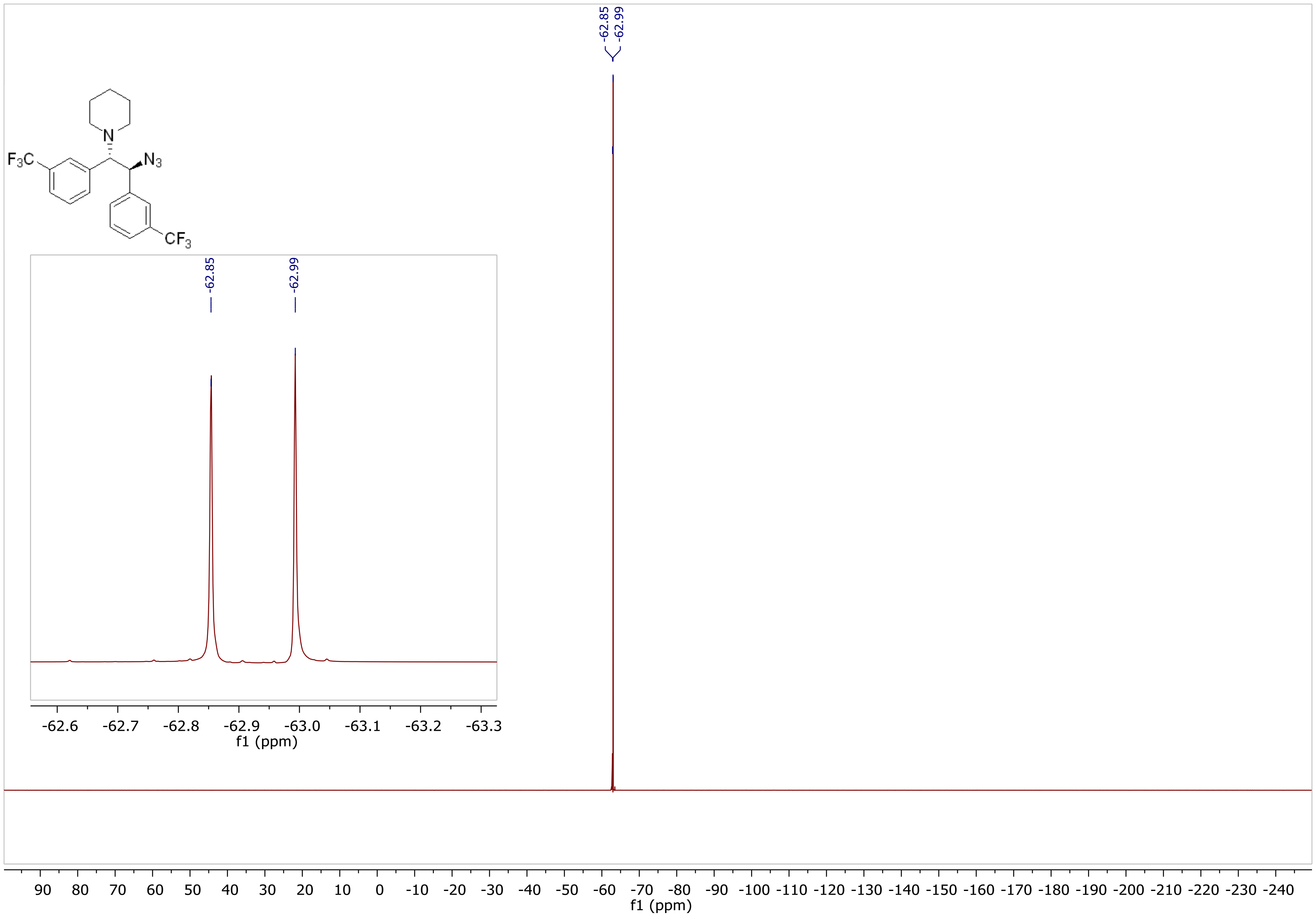

^{13}**C NMR** (101 MHz, $CDCl_3$)

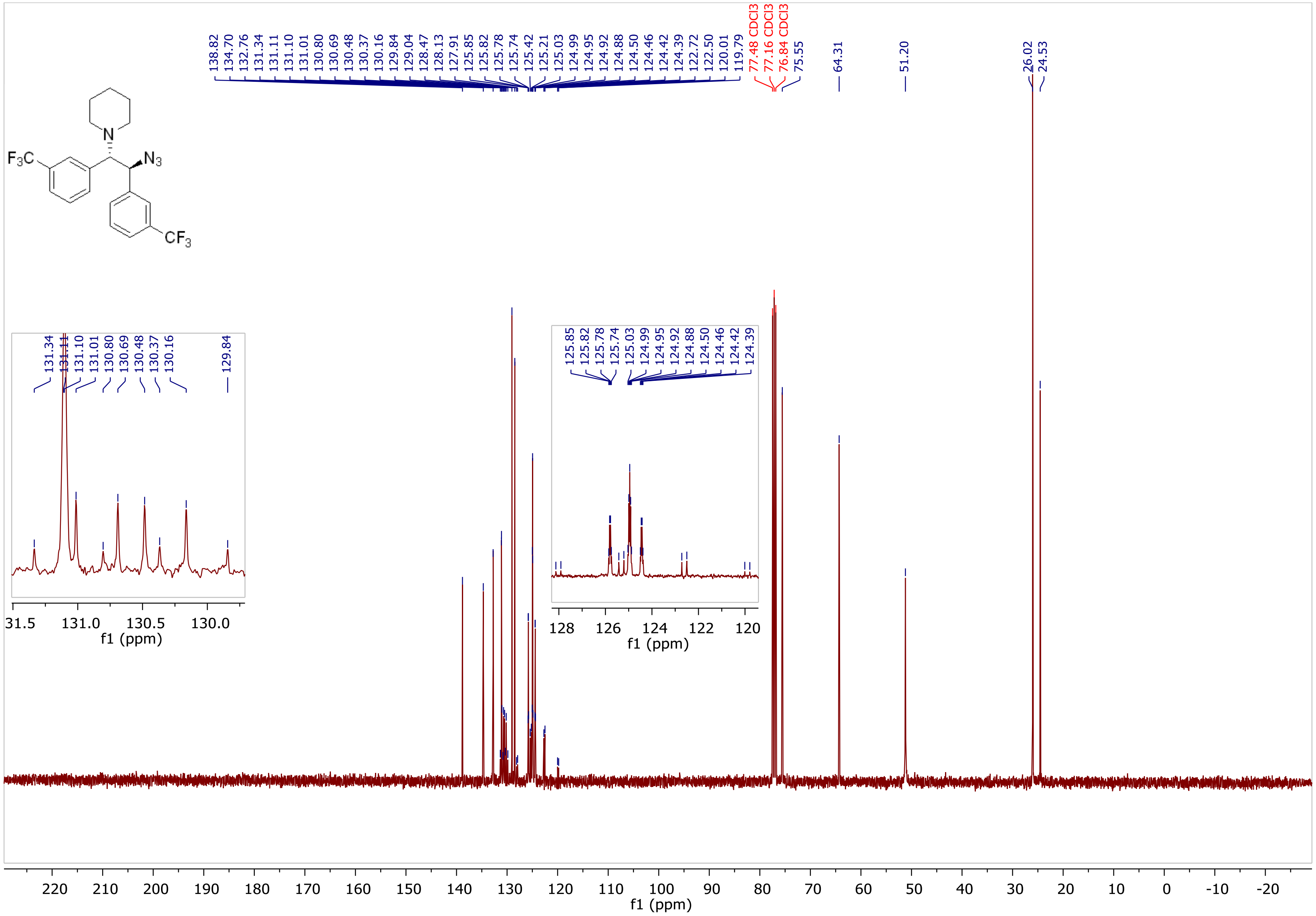

1-((1*S*,2*S*)-2-azido-1,2-bis(4-bromophenyl)ethyl)piperidine (3o)

^{1}H NMR (400 MHz, $CDCl_3$)

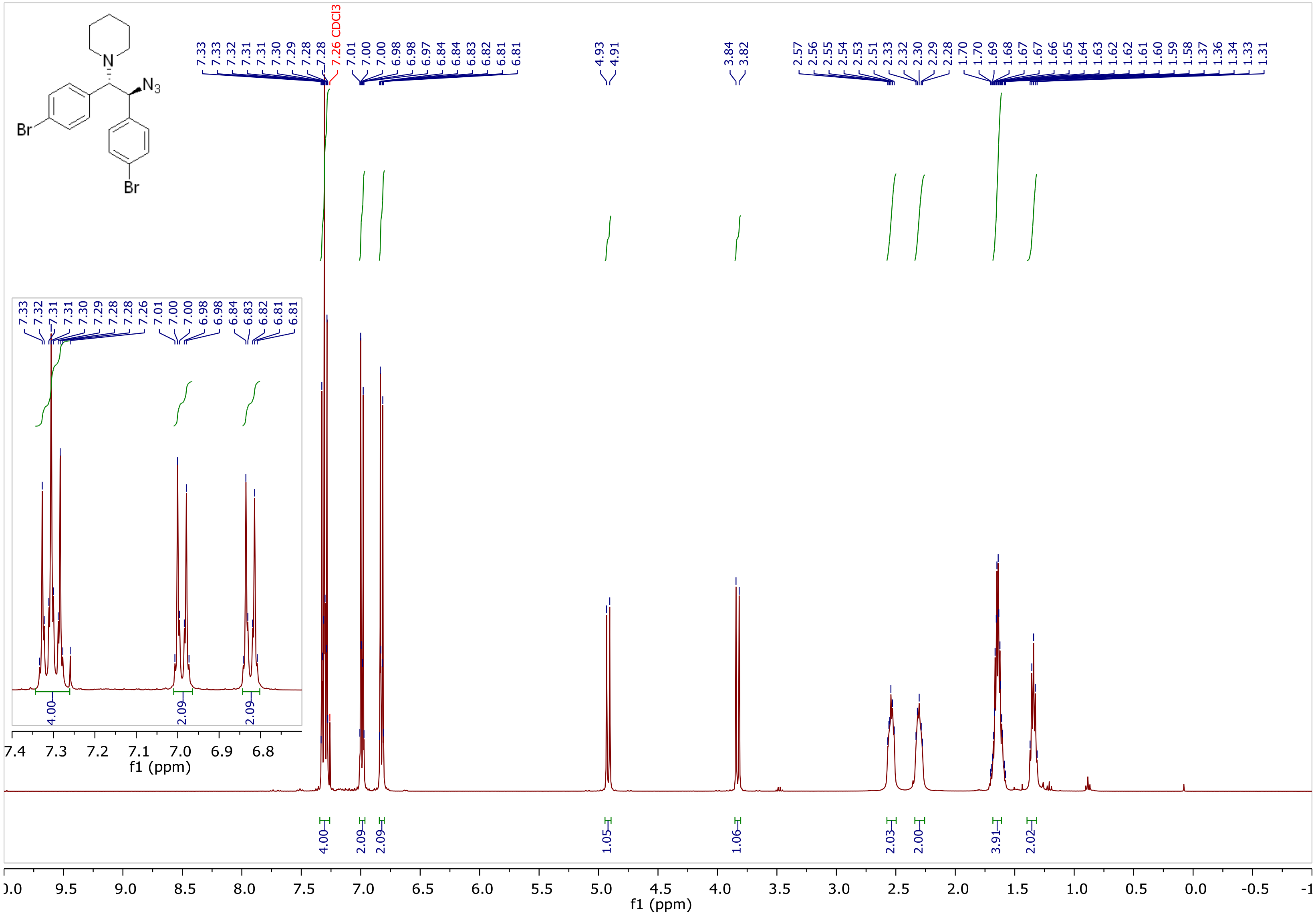

^{13}C NMR (101 MHz, $CDCl_3$)

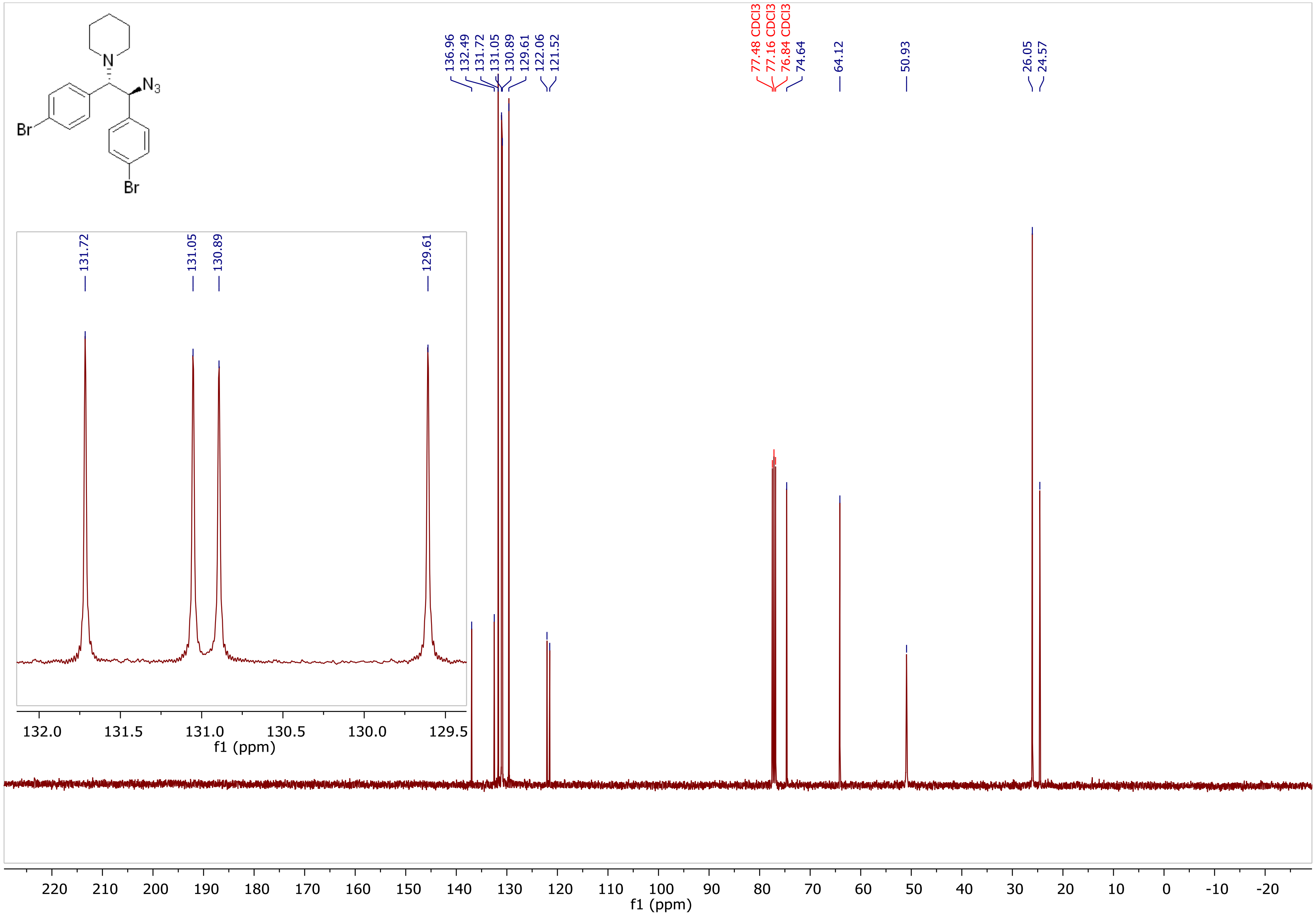

1-((1*S*,2*S*)-2-azido-1,2-bis(4-(*tert*-butyl)phenyl)ethyl)piperidine (3p)

^{1}H NMR (500 MHz, $CDCl_3$)

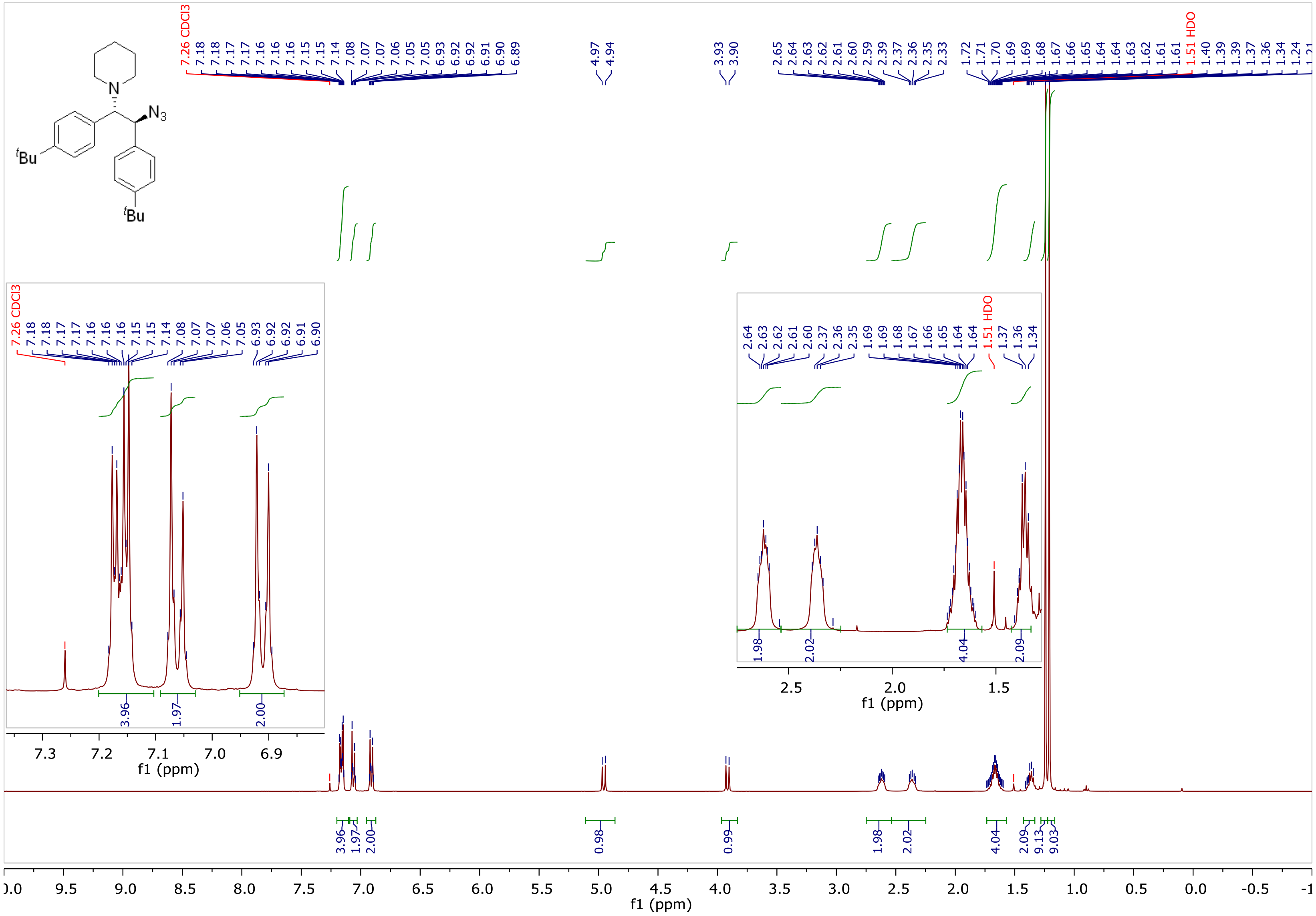

^{13}C NMR (126 MHz, $CDCl_3$)

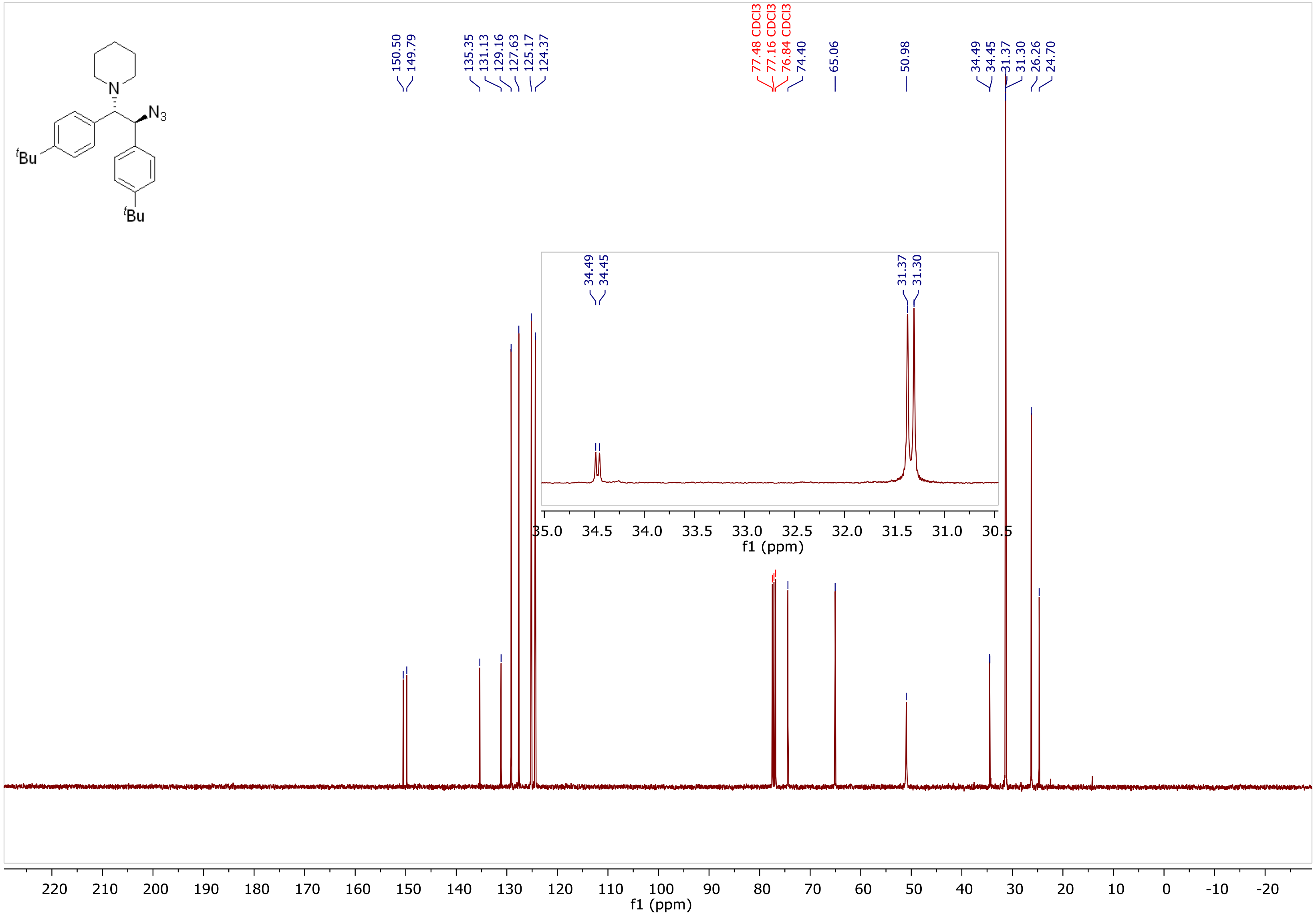

1-((1*S*,2*S*)-2-azido-1,2-bis(4-(trifluoromethyl)phenyl)ethyl)piperidine (3q)

^{1}H NMR (400 MHz, $CDCl_3$)

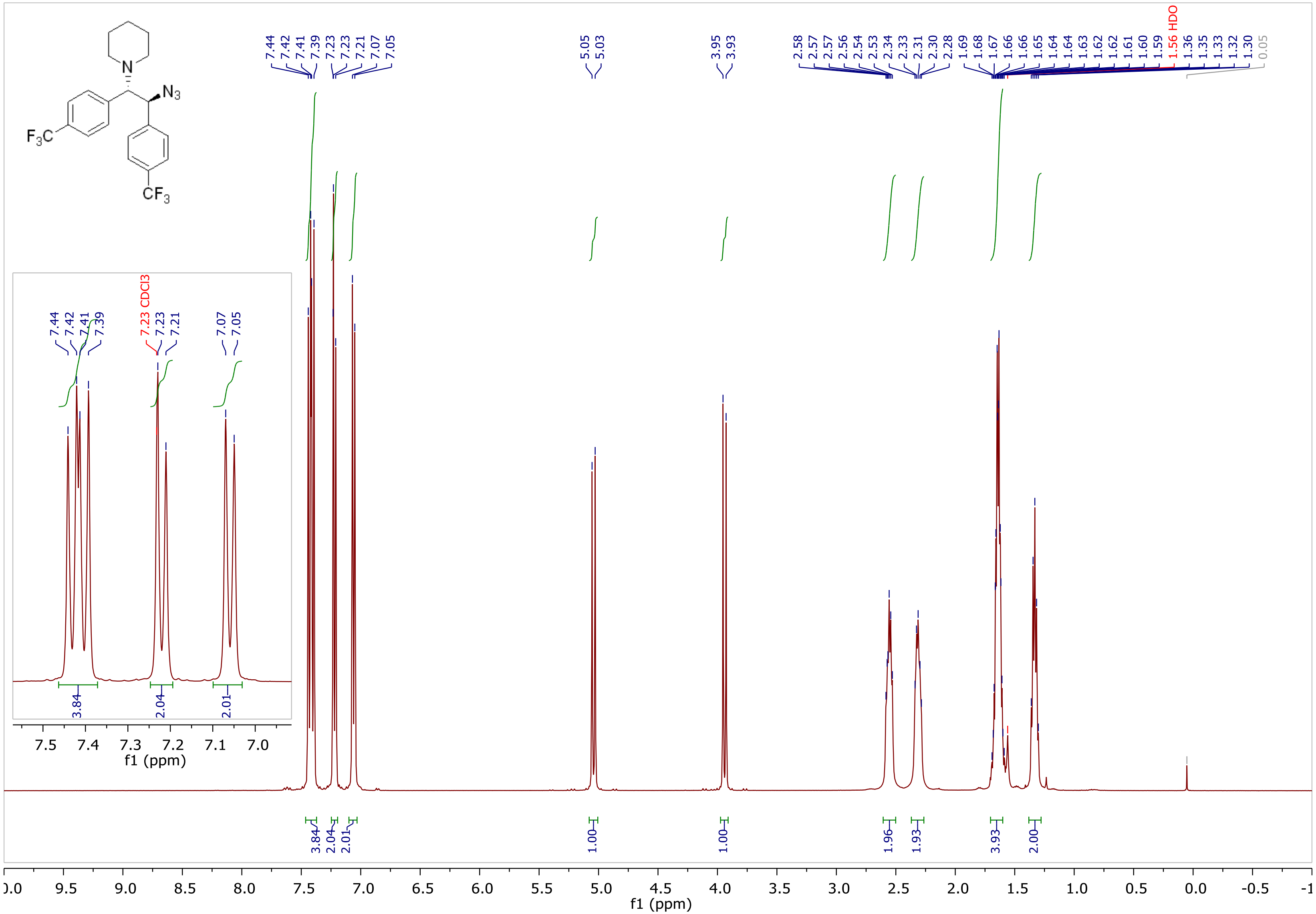

^{19}F NMR (377 MHz, $CDCl_3$)

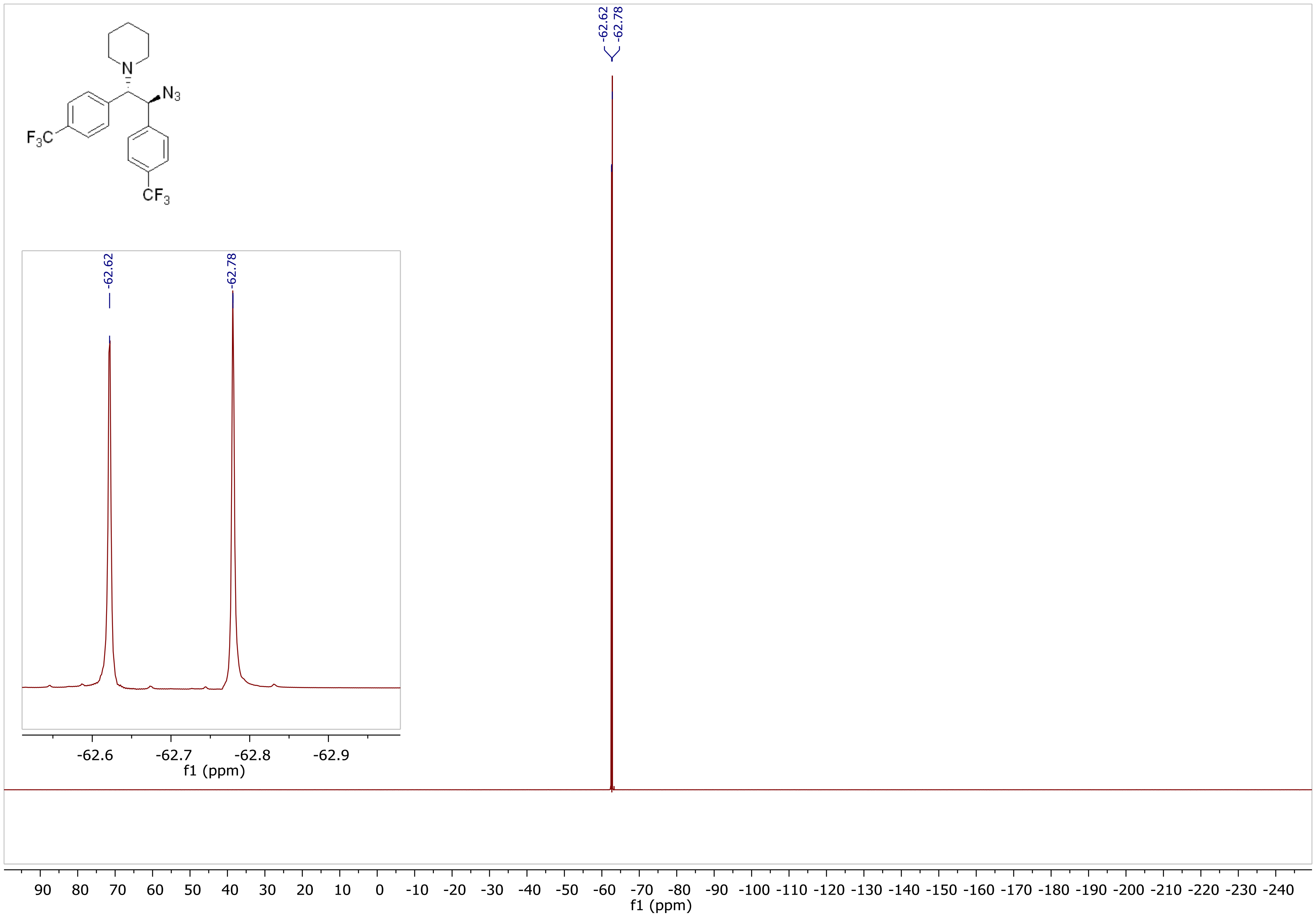

^{13}C NMR (101 MHz, $CDCl_3$)

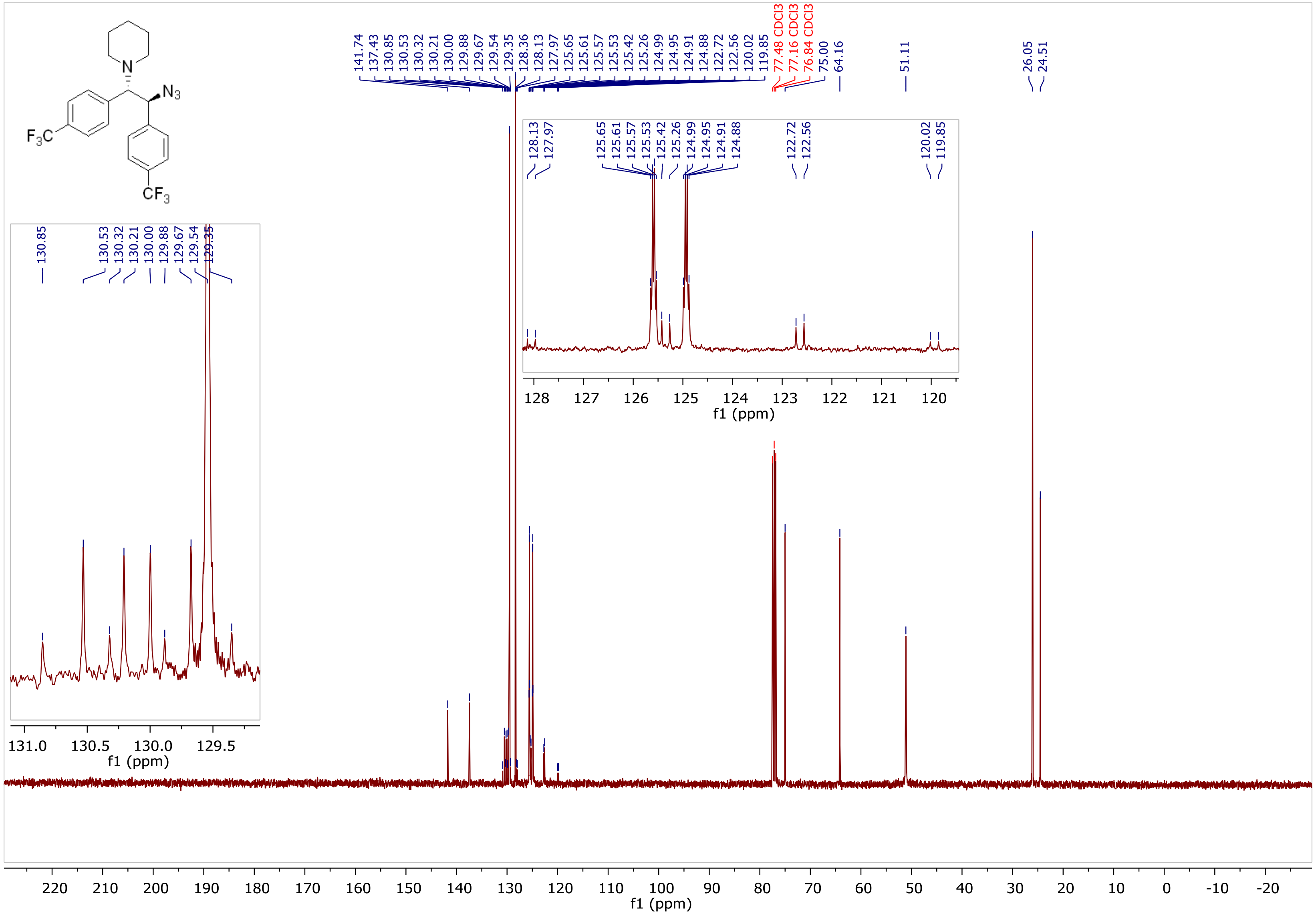

N-((1*R*,2*R*)-2-azido-1,2-di(pyridin-2-yl)ethyl)-*N*-propylpropan-1-amine (3r)

^{1}H NMR (400 MHz, $CDCl_3$)

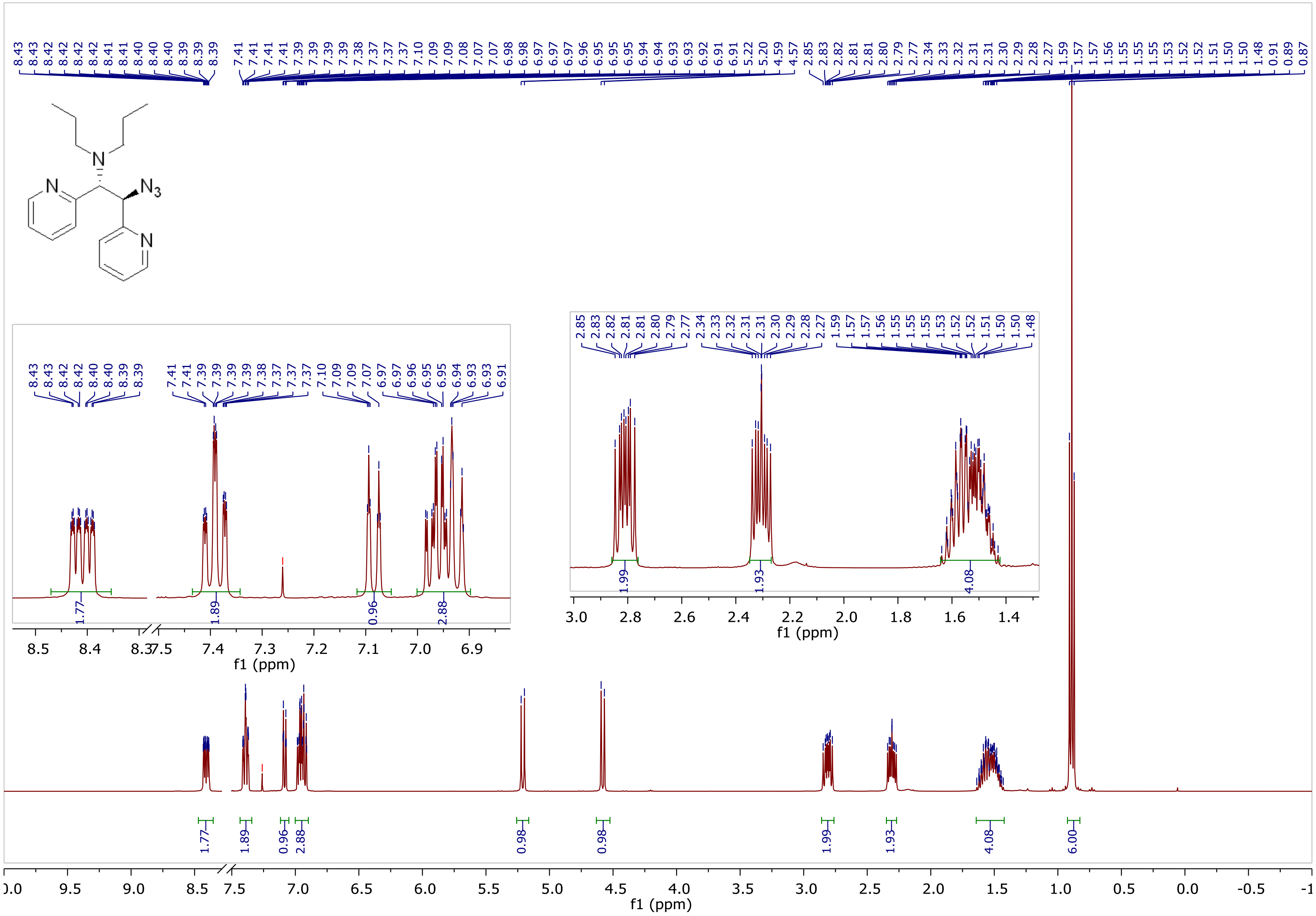

^{13}C NMR (101 MHz, $CDCl_3$)

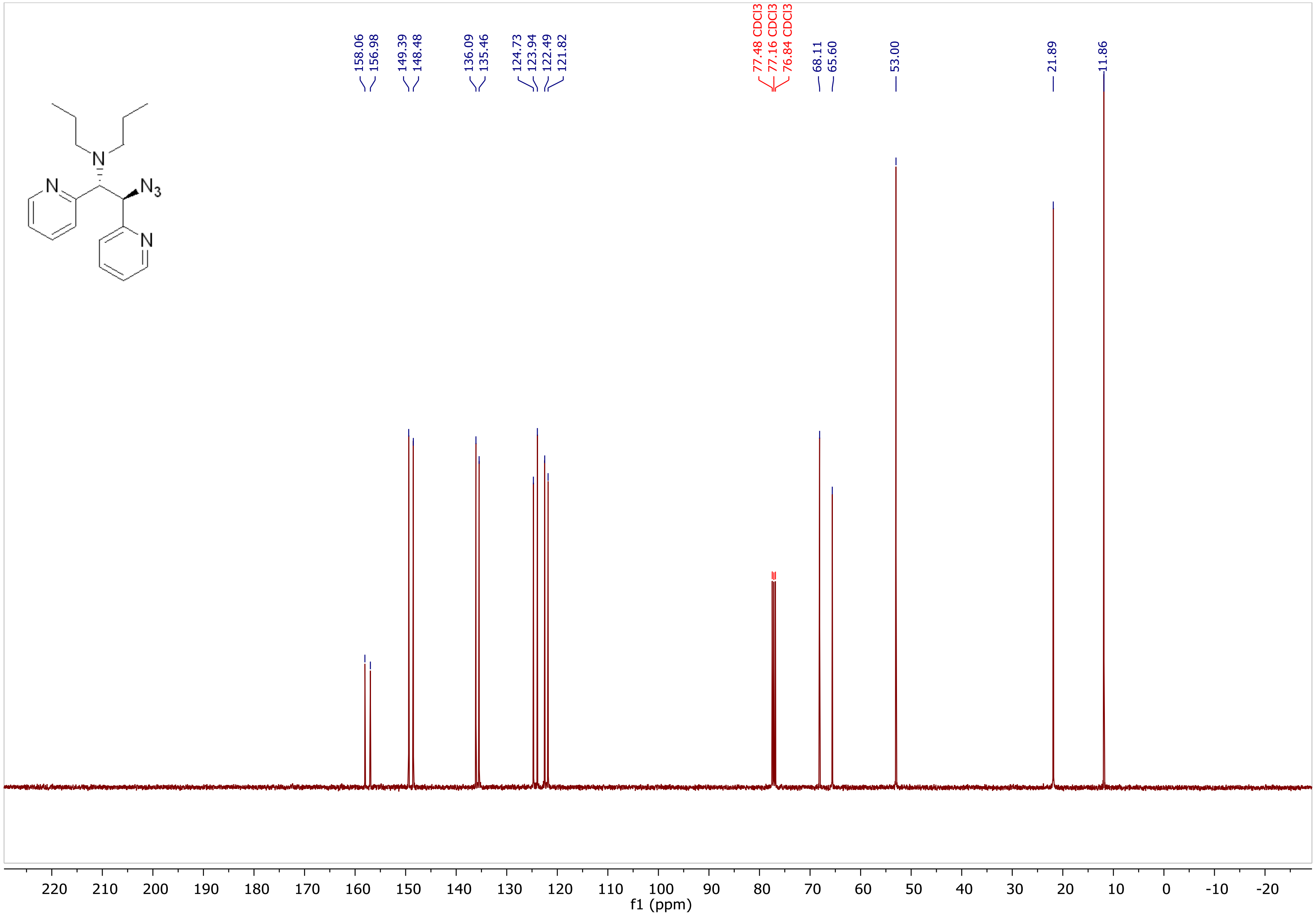

2-azido-*N*-benzyl-*N*-methylcyclohexan-1-amine (3s)

^{1}H NMR (400 MHz, $CDCl_3$)

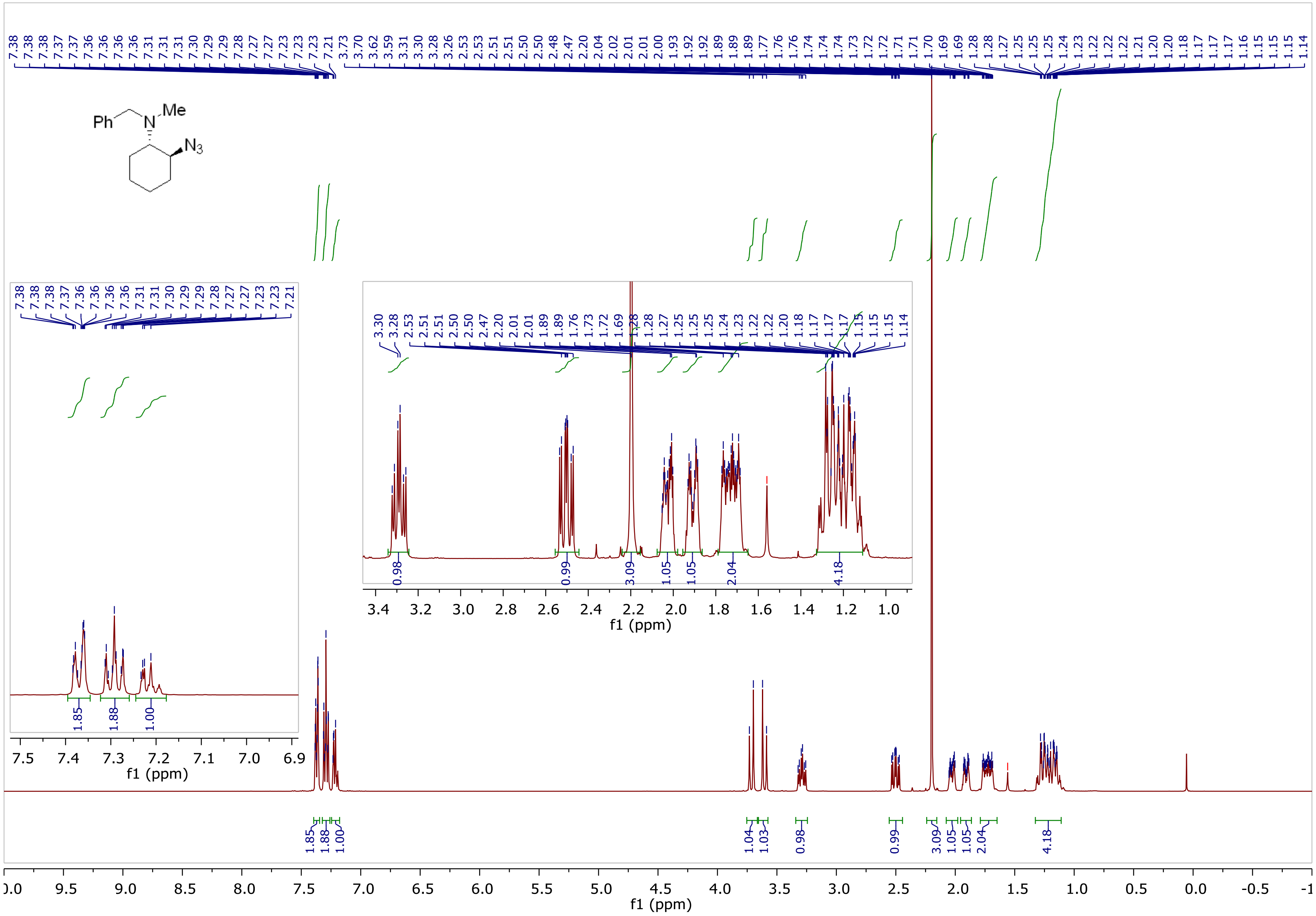

^{13}C NMR (101 MHz, $CDCl_3$)

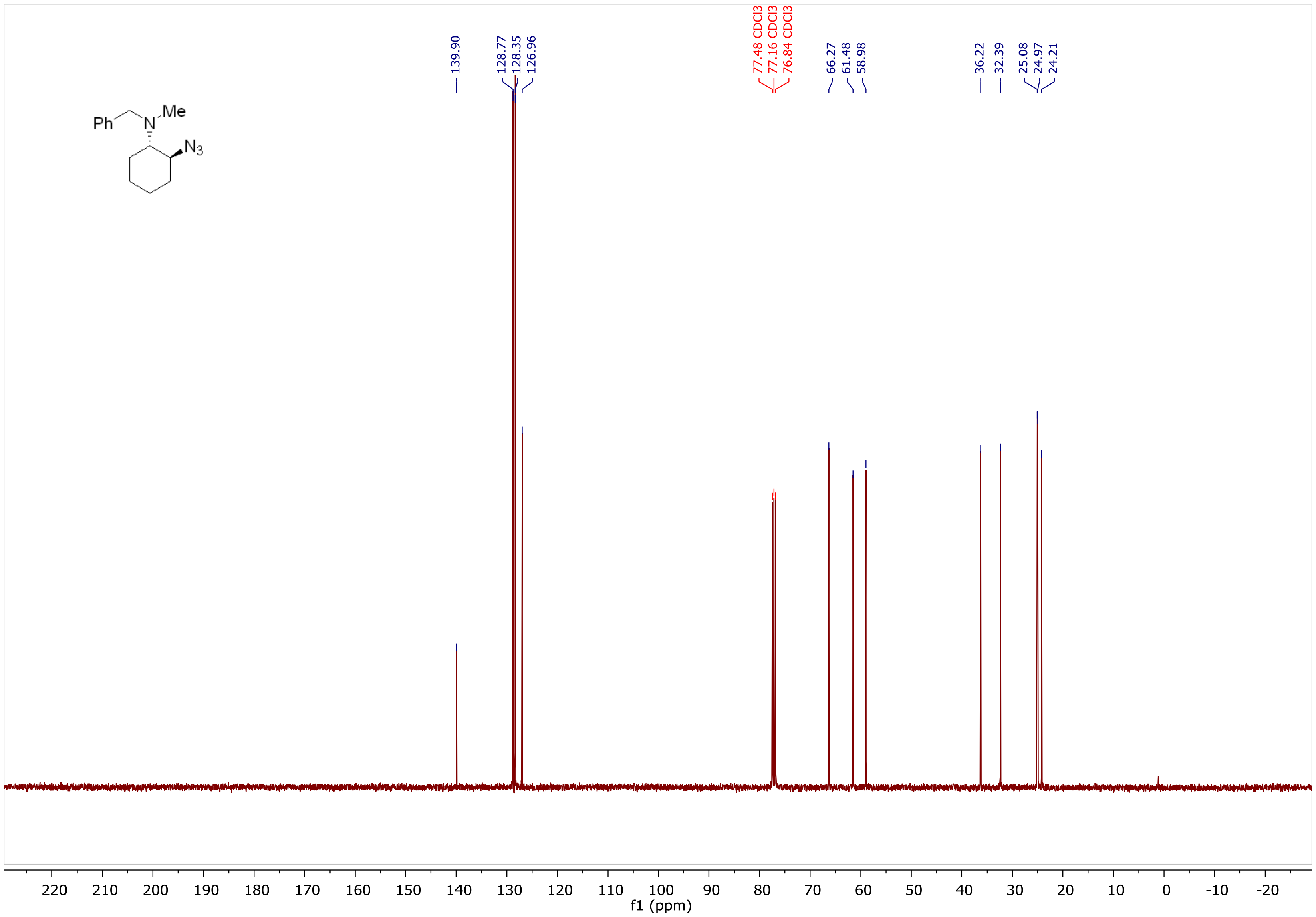

(1*S*,2*S*)-1,2-diphenyl-2-(piperidin-1-yl)ethan-1-amine

^{1}H NMR (400 MHz, $CDCl_3$)

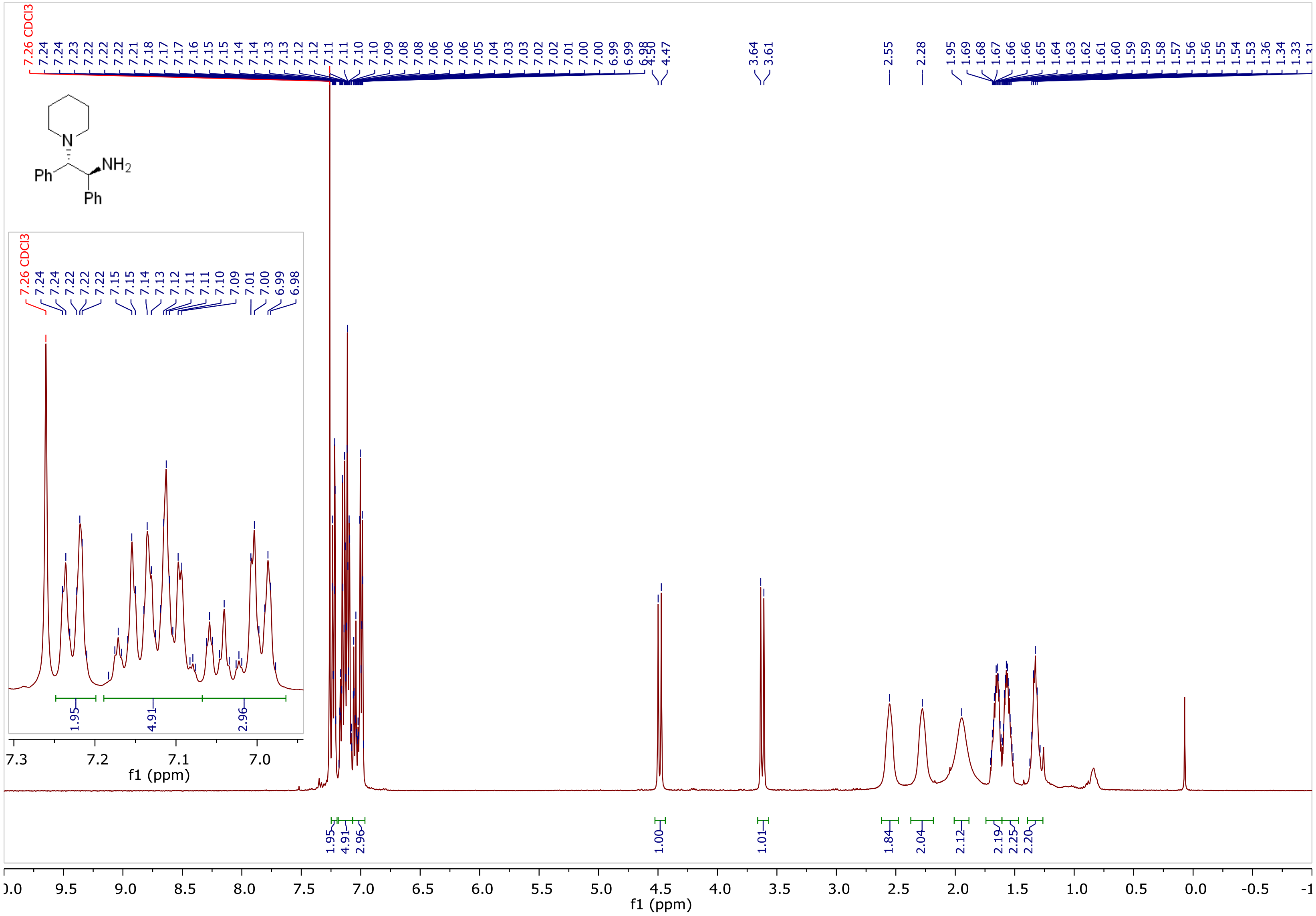

^{13}C NMR (101 MHz, $CDCl_3$)

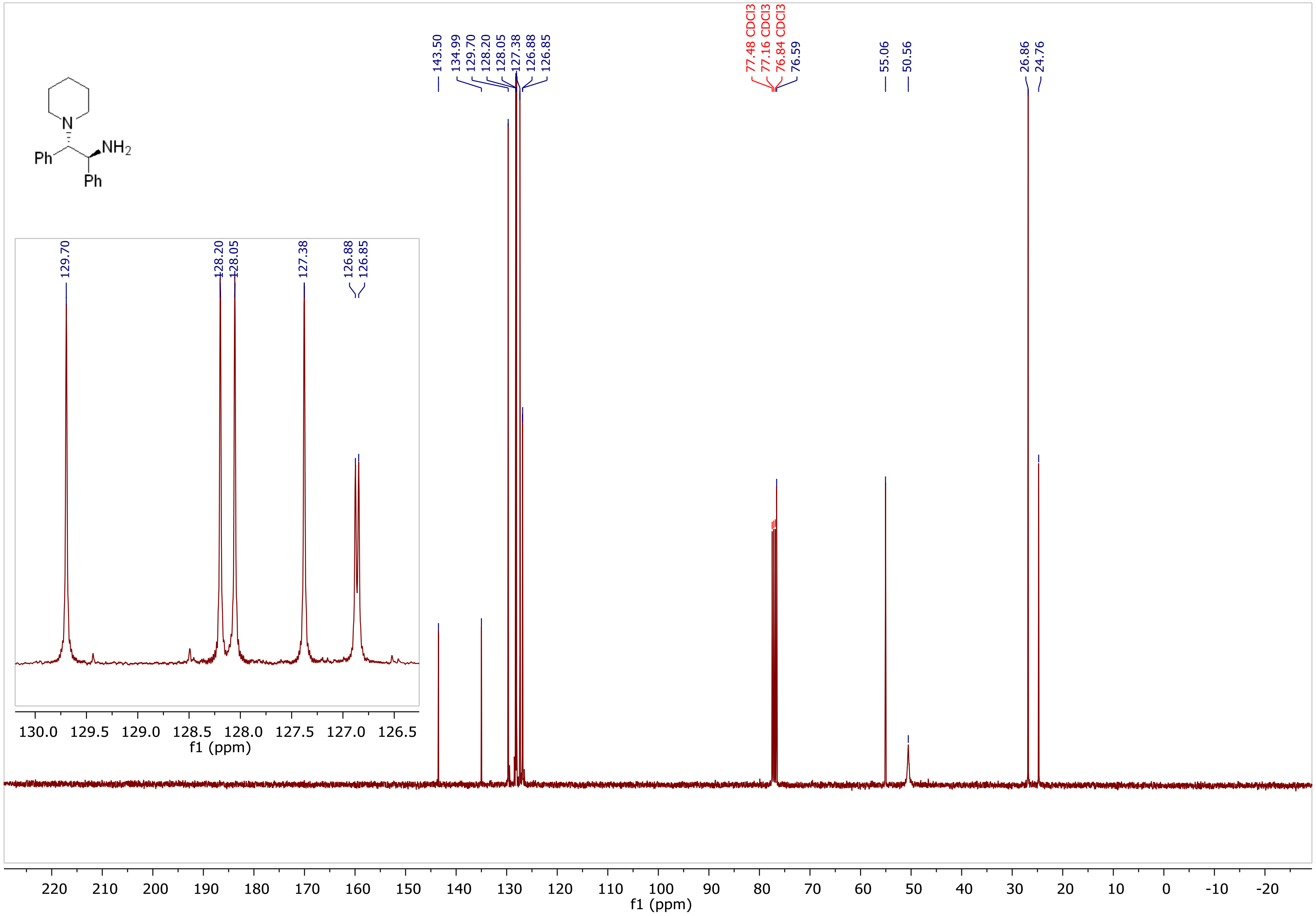

(1*S*,2*S*)-1,2-diphenyl-1,2-di(piperidin-1-yl)ethane (4)

^{1}H NMR (400 MHz, $CDCl_3$)

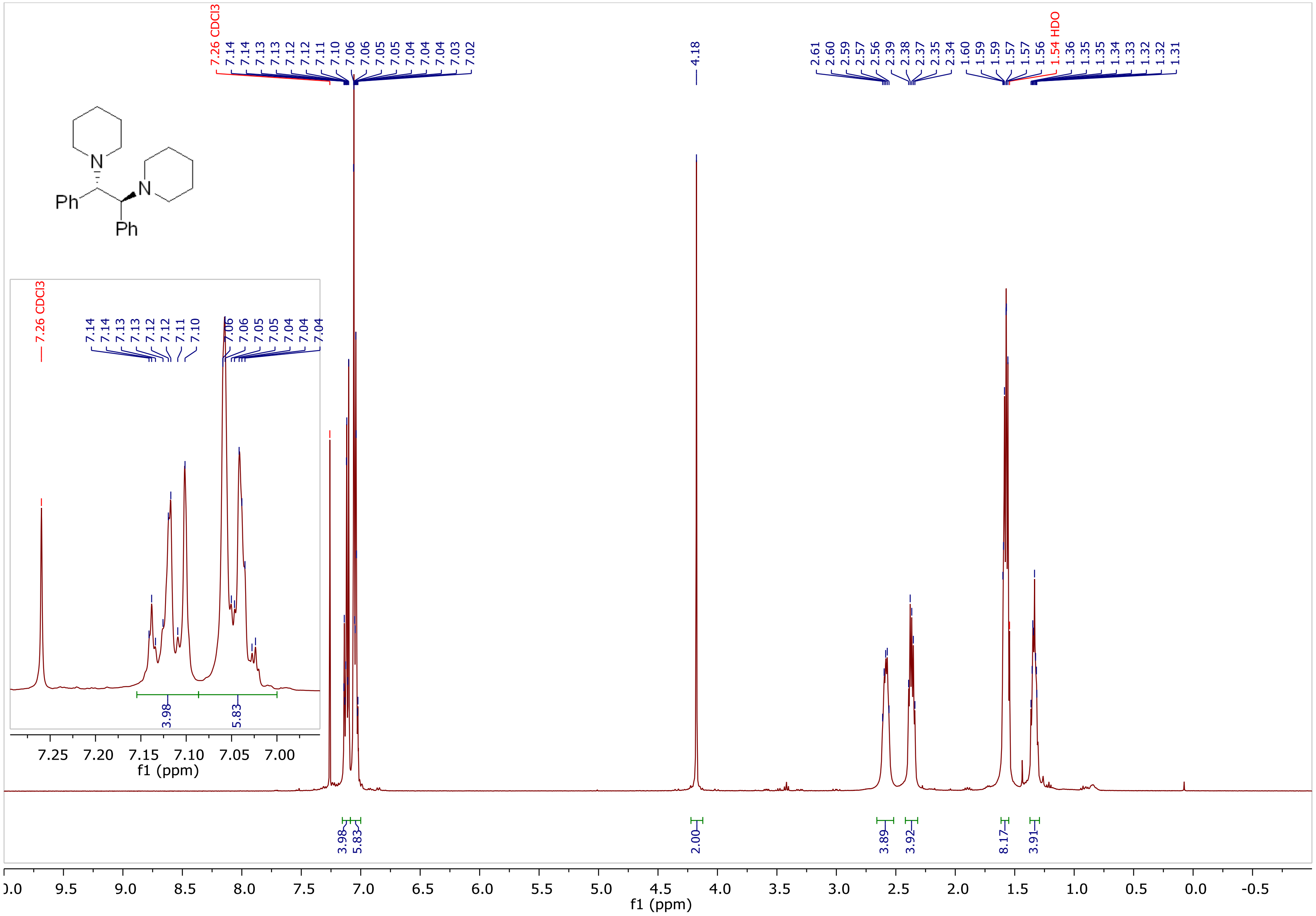

^{13}C NMR (101 MHz, $CDCl_3$)

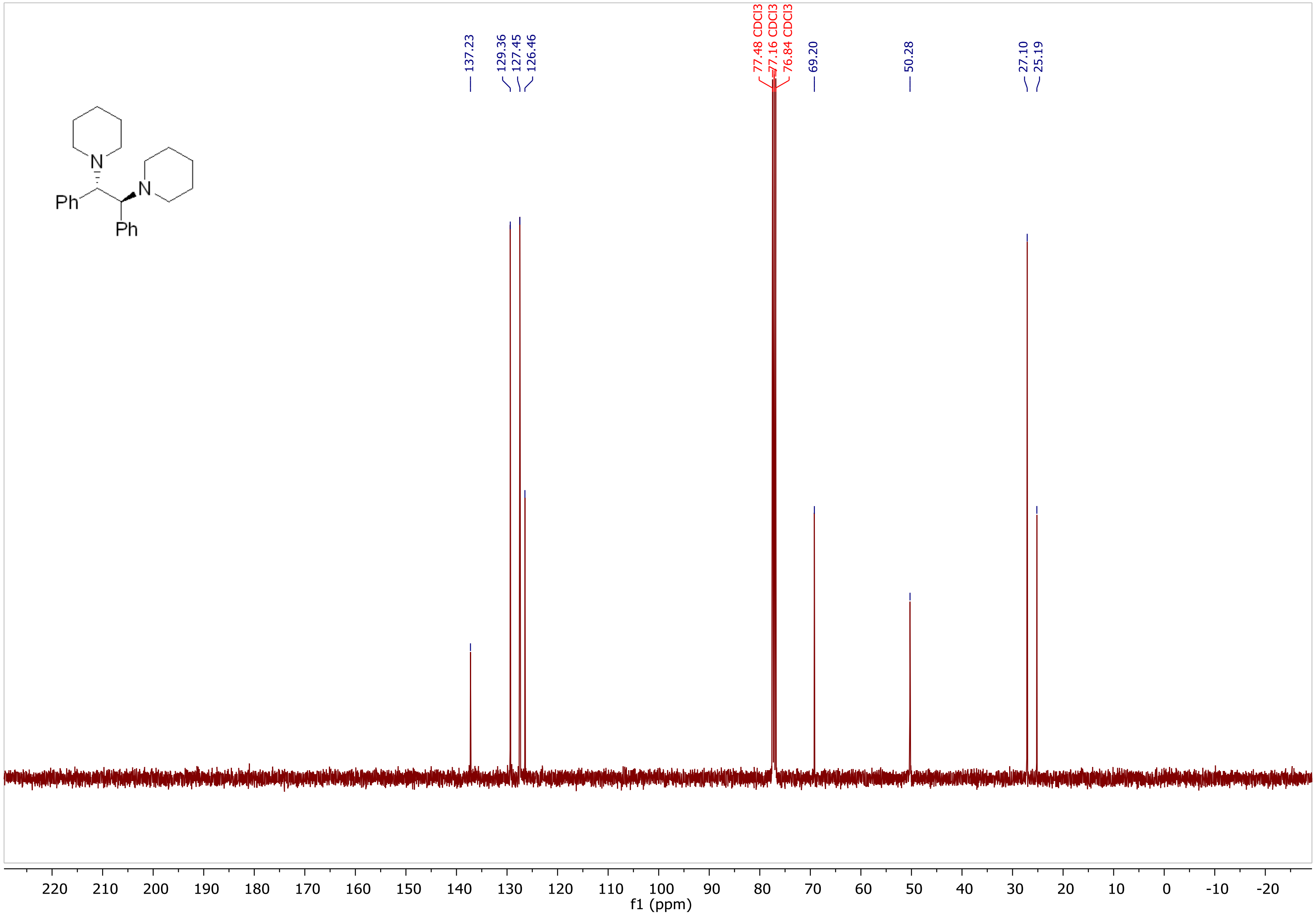

^{1}**H NMR** (500 MHz, $CDCl_3$, 233 K)

Residuals of fits in blue.

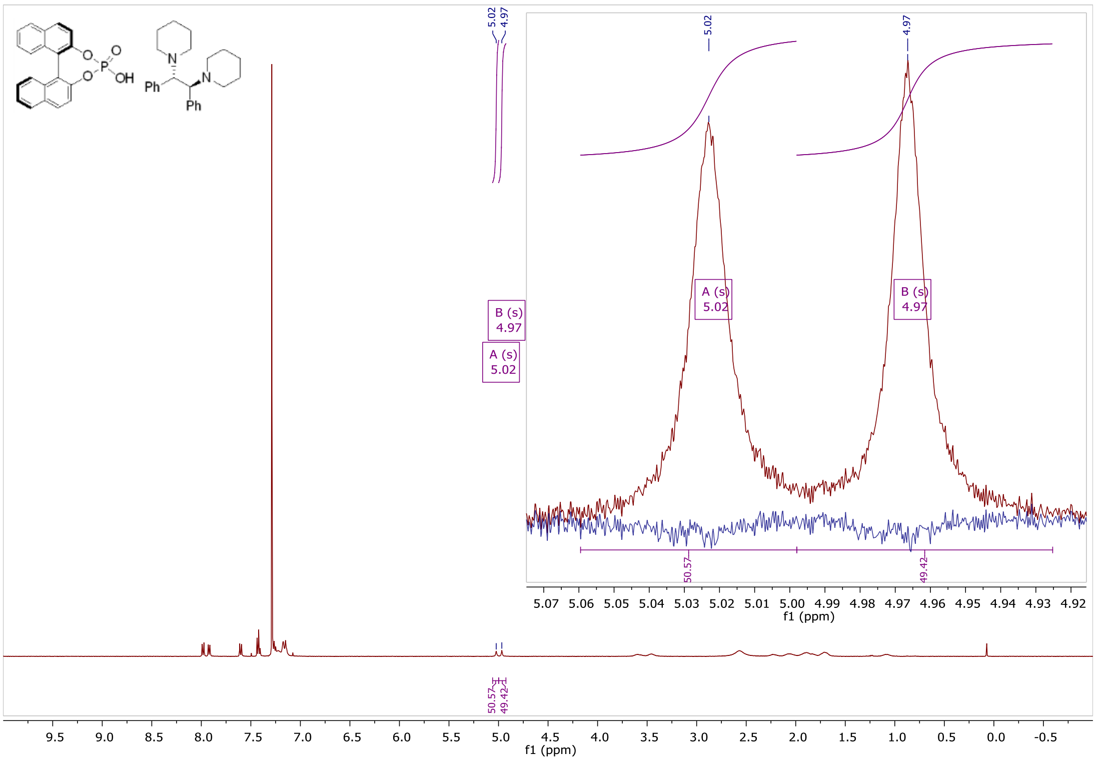

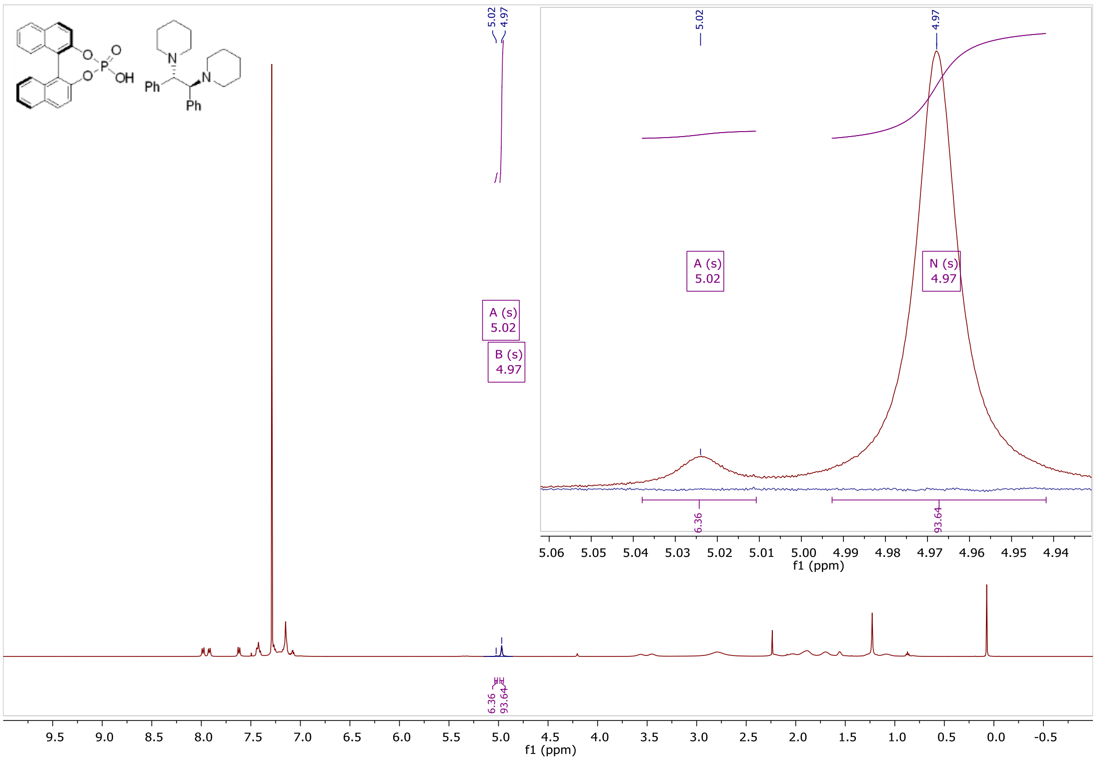

HPLC traces

1-((1*S*,2*S*)-2-azido-1,2-diphenylethyl)piperidine (3a)

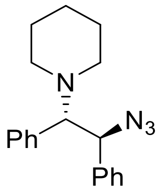

<Chromatogram>

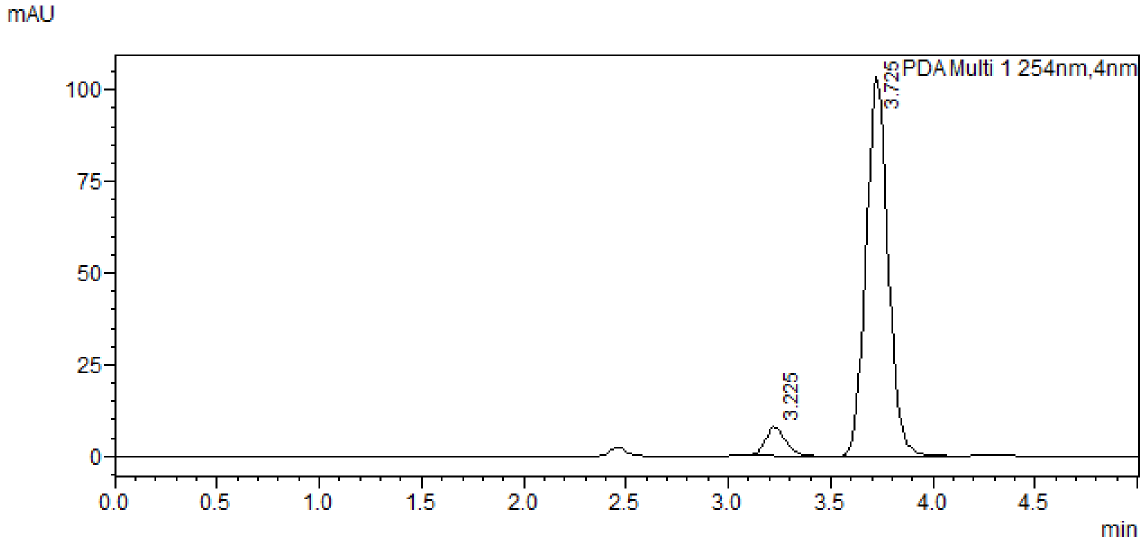

<Peak Table>

PDA Ch1 254nm

Peak#	Ret. Time	Area	Height	Conc.	Unit	Mark	Name
1	3.225	53320	7841	6.414		M	
2	3.725	777983	103332	93.586		M	
Total		831303	111173				

<Chromatogram>

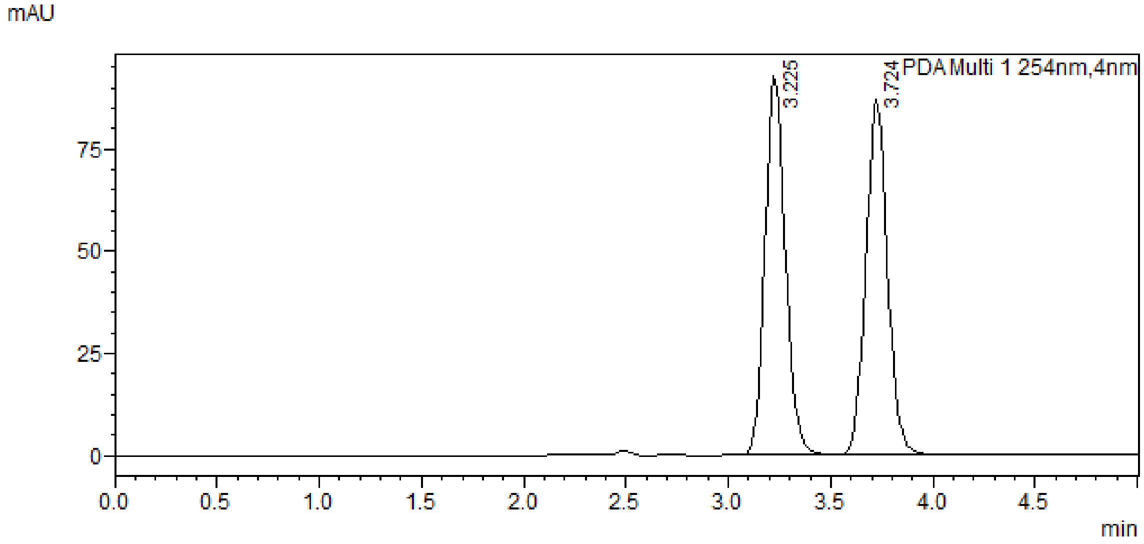

<Peak Table>

PDA Ch1 254nm

Peak#	Ret. Time	Area	Height	Conc.	Unit	Mark	Name
1	3.225	638505	92549	50.096		M	
2	3.724	636070	86952	49.904		M	
Total		1274575	179501				

1-((1*S*,2*S*)-2-azido-1,2-diphenylethyl)-4-methoxypiperidine (3b)

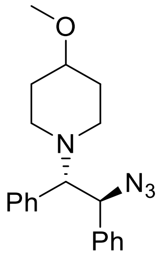

<Chromatogram>

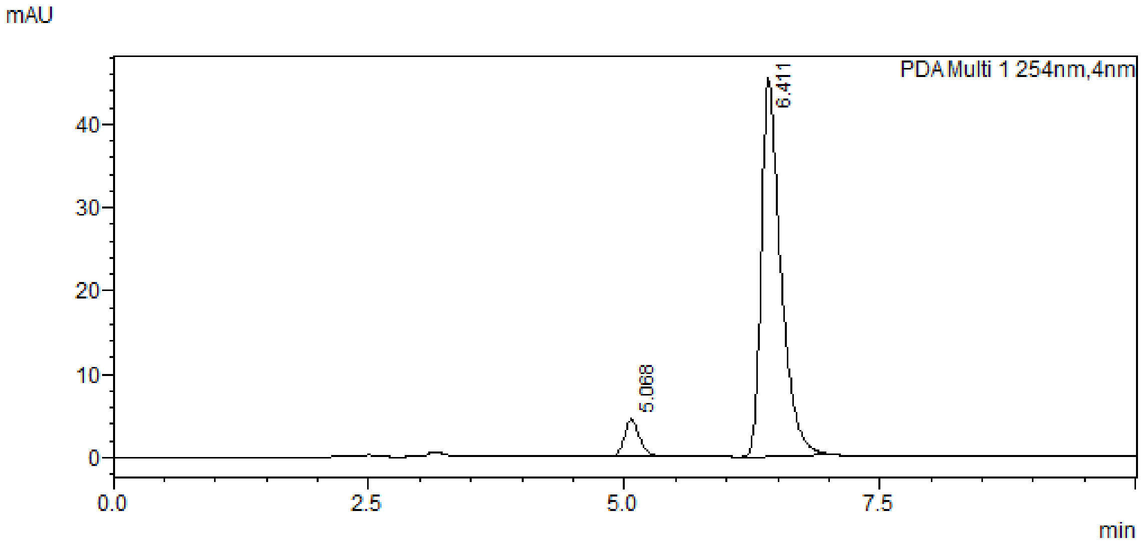

<Peak Table>

PDA Ch1 254nm

Peak#	Ret. Time	Area	Height	Conc.	Unit	Mark	Name
1	5.068	46664	4526	7.153		M	
2	6.411	605739	45457	92.847		M	
Total		652403	49983				

<Chromatogram>

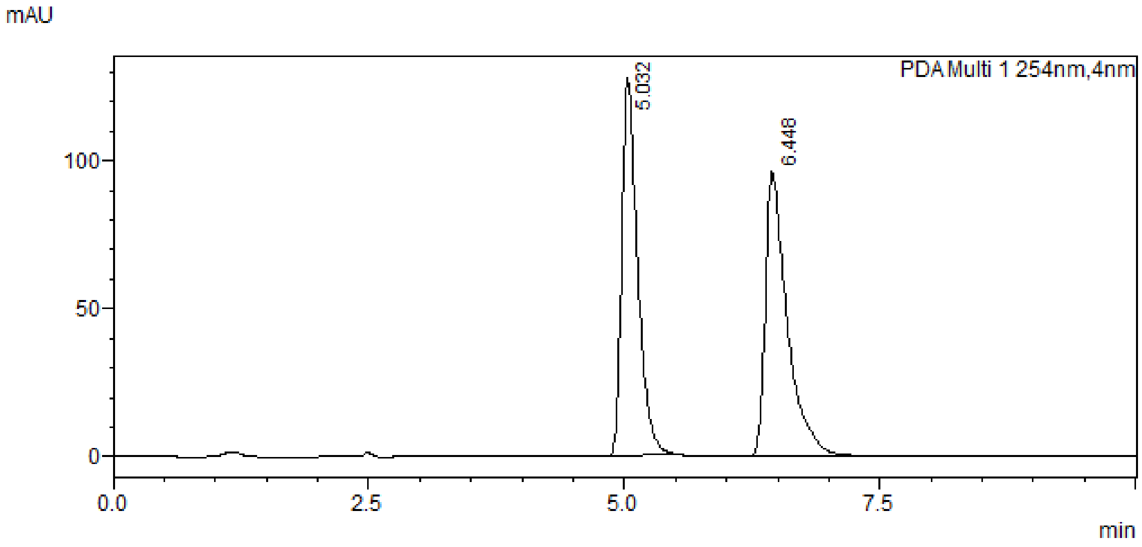

<Peak Table>

PDA Ch1 254nm

Peak#	Ret. Time	Area	Height	Conc.	Unit	Mark	Name
1	5.032	1378058	127706	49.001		M	
2	6.448	1434223	95975	50.999		M	
Total		2812280	223680				

1-((1*S*,2*S*)-2-azido-1,2-diphenylethyl)-4,4-difluoropiperidine (3c)

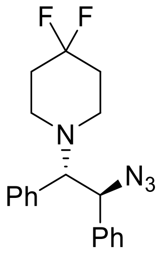

<Chromatogram>

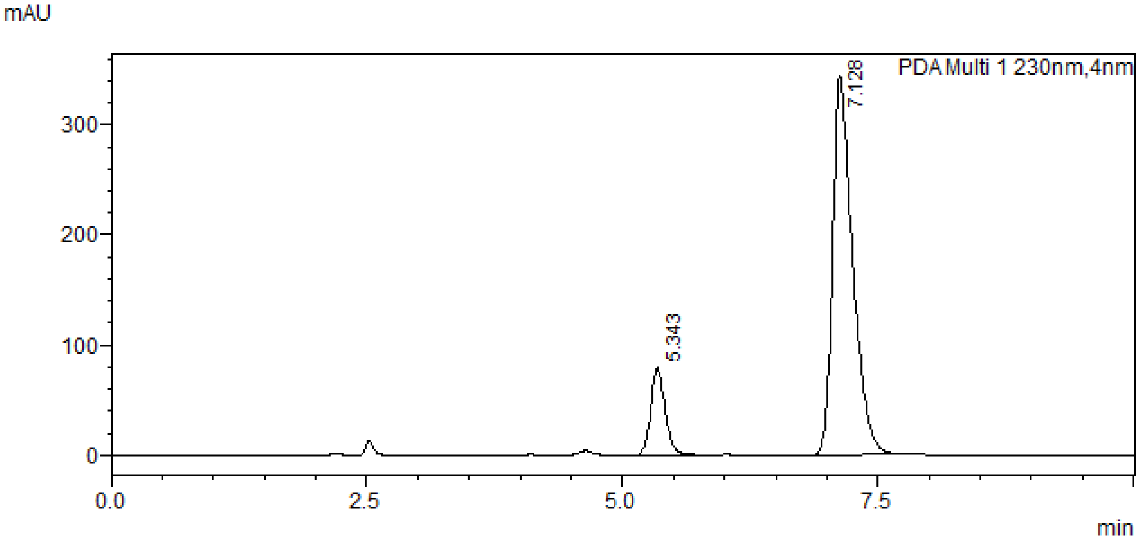

<Peak Table>

PDA Ch1 230nm

Peak#	Ret. Time	Area	Height	Conc.	Unit	Mark	Name
1	5.343	758298	79356	13.882		M	
2	7.128	4704297	344214	86.118		M	
Total		5462596	423570				

<Chromatogram>

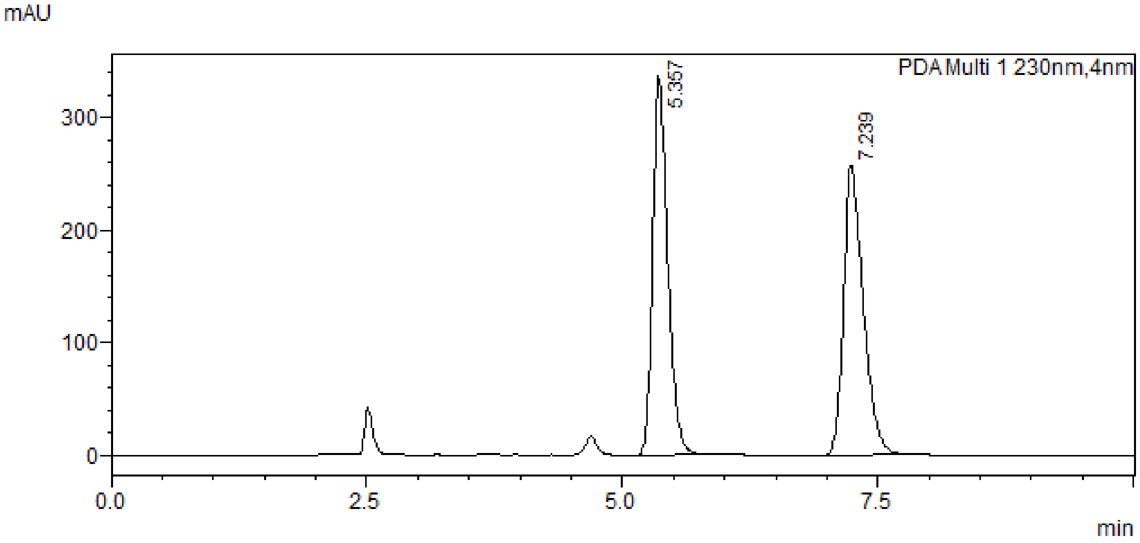

<Peak Table>

PDA Ch1 230nm

Peak#	Ret. Time	Area	Height	Conc.	Unit	Mark	Name
1	5.357	3394355	336539	49.731		M	
2	7.239	3431022	257497	50.269		M	
Total		6825377	594035				

1-((1*S*,2*S*)-2-azido-1,2-diphenylethyl)pyrrolidine (3d)

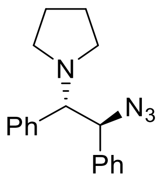

<Chromatogram>

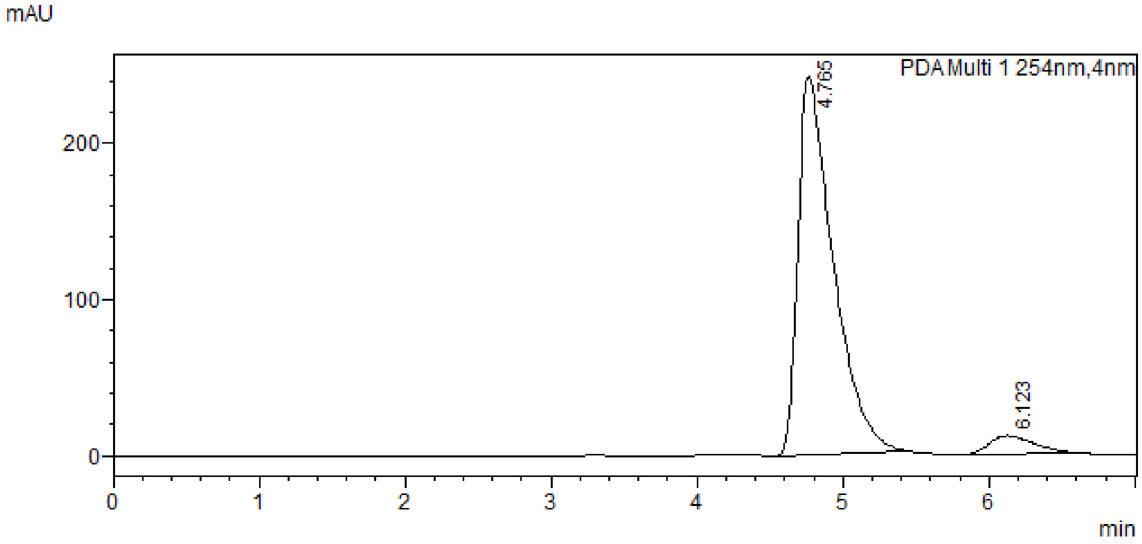

<Peak Table>

PDA Ch1 254nm

Peak#	Ret. Time	Area	Height	Conc.	Unit	Mark	Name
1	4.765	4059107	241992	93.982		M	
2	6.123	259927	11898	6.018		M	
Total		4319034	253889				

<Chromatogram>

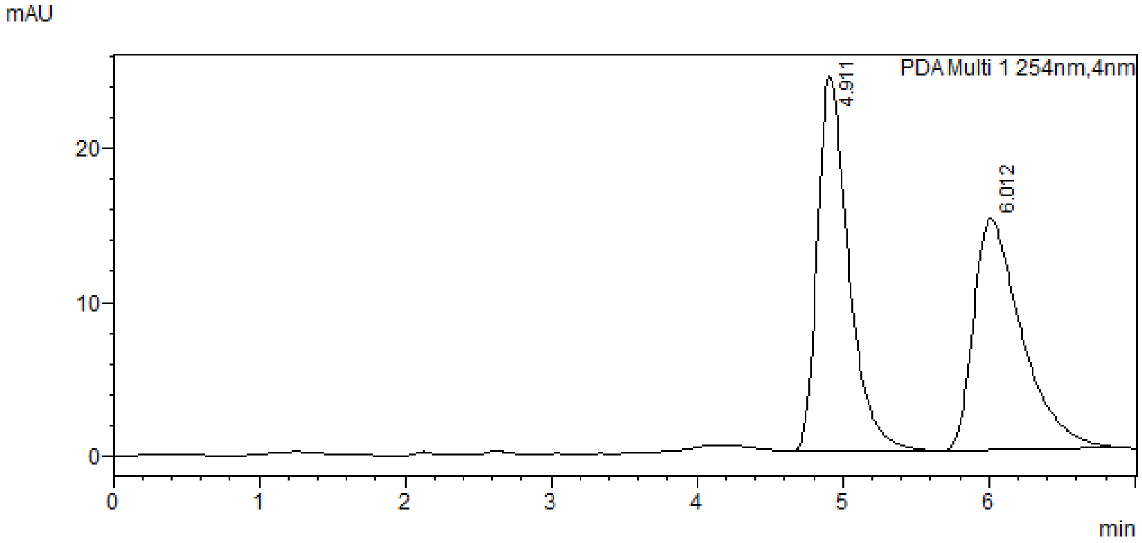

<Peak Table>

PDA Ch1 254nm

Peak#	Ret. Time	Area	Height	Conc.	Unit	Mark	Name
1	4.911	362646	24375	50.990		M	
2	6.012	348561	15050	49.010		M	
Total		711208	39425				

4-((1*S*,2*S*)-2-azido-1,2-diphenylethyl)morpholine (3e)

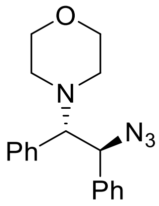

<Chromatogram>

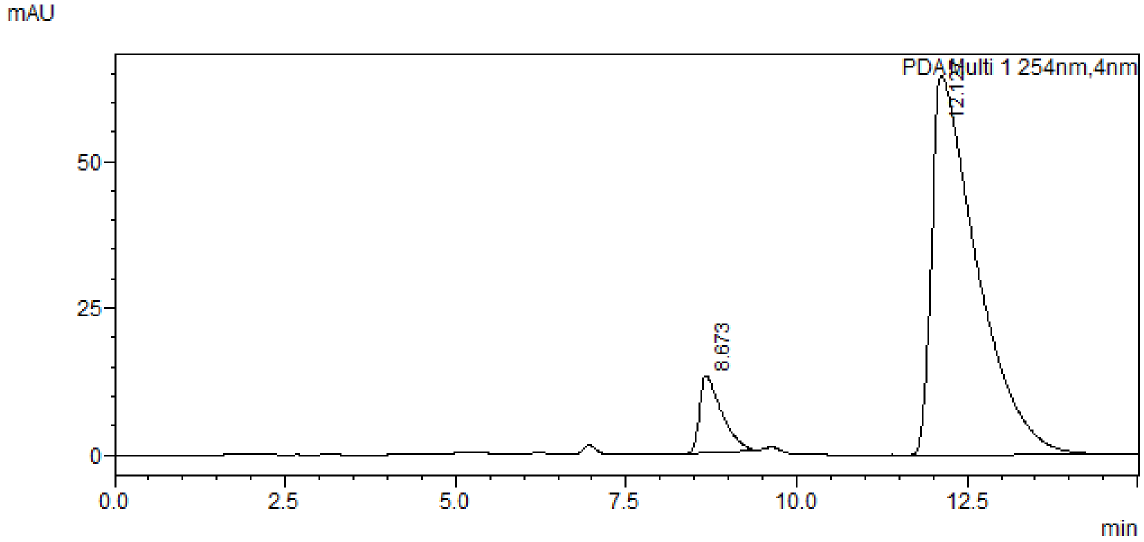

<Peak Table>

PDA Ch1 254nm

Peak#	Ret. Time	Area	Height	Conc.	Unit	Mark	Name
1	8.673	293000	13134	8.946		M	
2	12.127	2982345	64432	91.054		M	
Total		3275345	77566				

<Chromatogram>

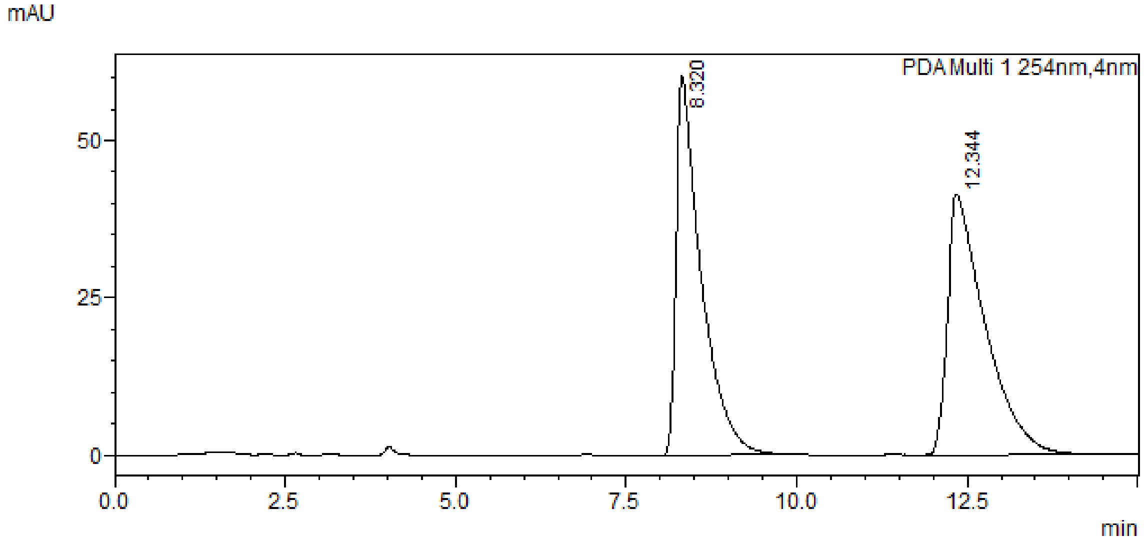

<Peak Table>

PDA Ch1 254nm

Peak#	Ret. Time	Area	Height	Conc.	Unit	Mark	Name
1	8.320	1541017	60244	49.149			
2	12.344	1594350	41387	50.851			
Total		3135367	101631				

1-((1*S*,2*S*)-2-azido-1,2-diphenylethyl)-4-cyclohexylpiperazine (3f)

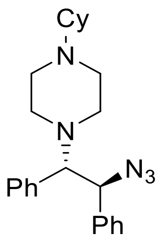

<Chromatogram>

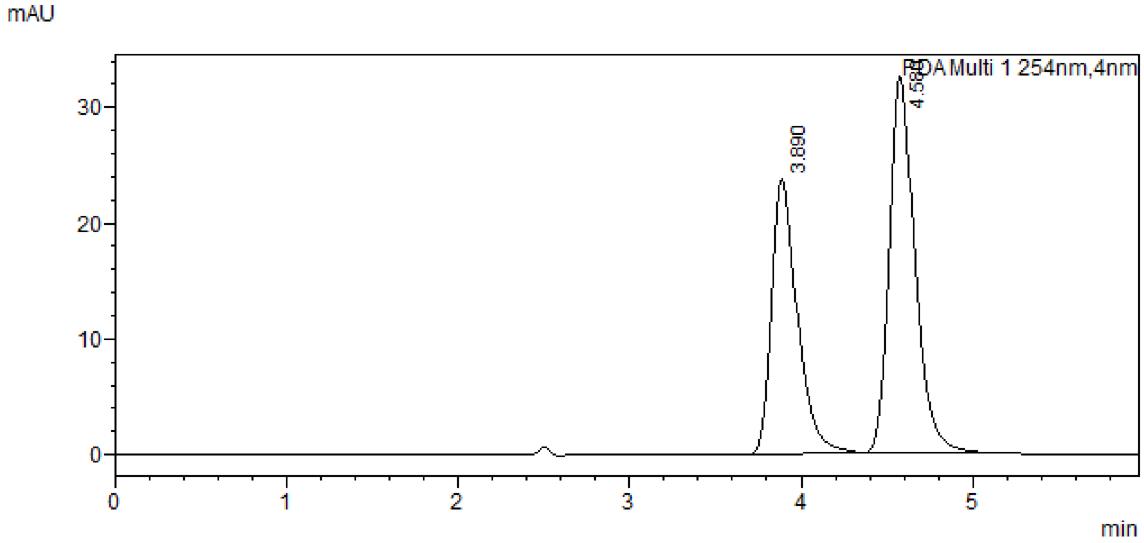

<Peak Table>

PDA Ch1 254nm

Peak#	Ret. Time	Area	Height	Conc.	Unit	Mark	Name
1	3.890	244023	23788	42.722		M	
2	4.580	327165	32462	57.278		M	
Total		571188	56250				

<Chromatogram>

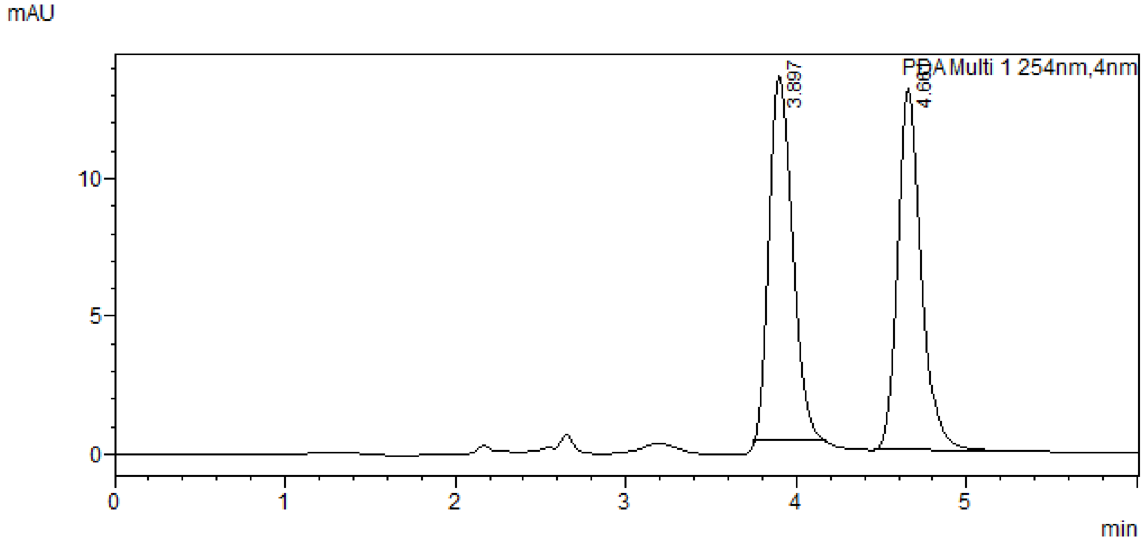

<Peak Table>

PDA Ch1 254nm

Peak#	Ret. Time	Area	Height	Conc.	Unit	Mark	Name
1	3.897	127670	13197	50.553		M	
2	4.661	124876	13074	49.447		M	
Total		252546	26272				

1-((1*S*,2*S*)-2-azido-1,2-diphenylethyl)-4-phenylpiperazine (3g)

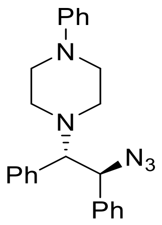

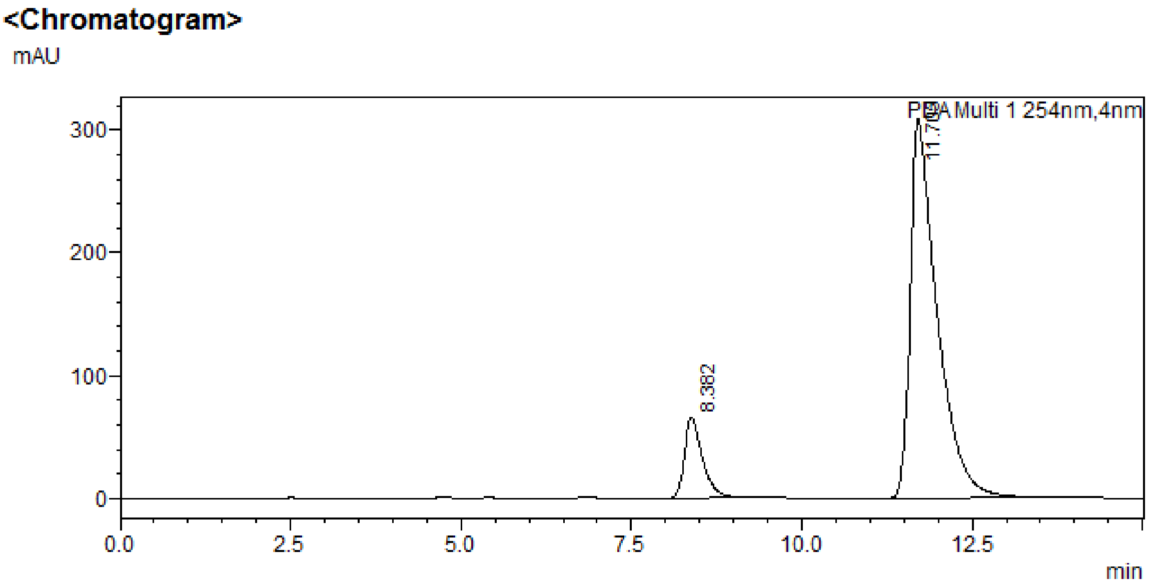

<Peak Table>

PDA Ch1 254nm

Peak#	Ret. Time	Area	Height	Conc.	Unit	Mark	Name
1	8.382	1210657	66029	12.513		M	
2	11.705	8464648	308684	87.487		M	
Total		9675305	374713				

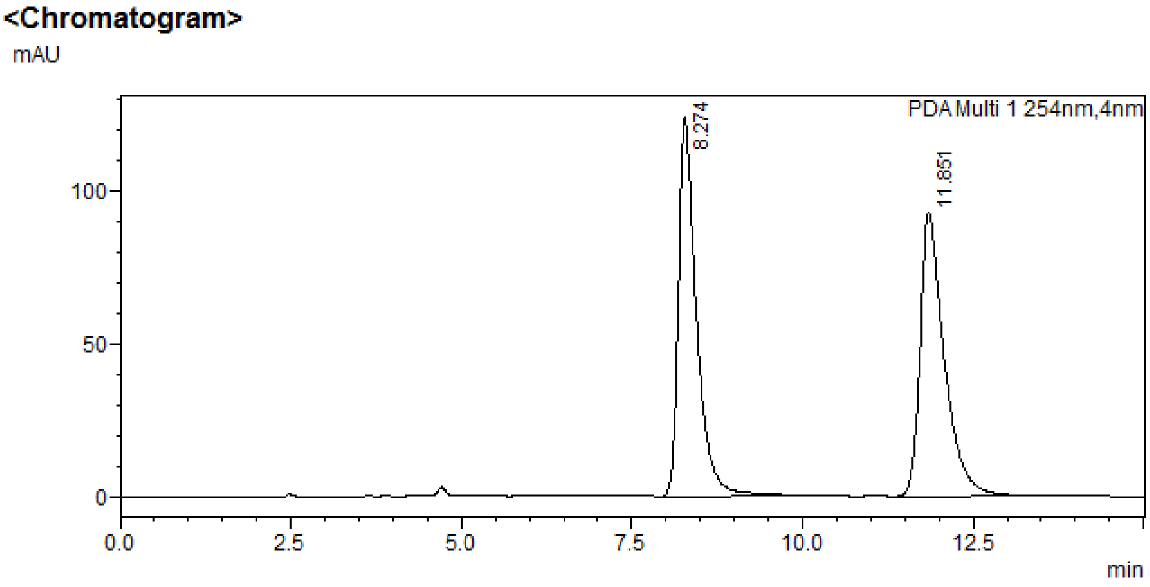

<Peak Table>

PDA Ch1 254nm

Peak#	Ret. Time	Area	Height	Conc.	Unit	Mark	Name
1	8.274	2304319	124081	50.172		M	
2	11.851	2288531	93226	49.828		M	
Total		4592849	217307				

2-((1*S*,2*S*)-2-azido-1,2-diphenylethyl)-1,2,3,4-tetrahydroisoquinoline (3h)

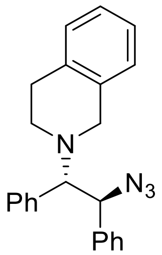

<Chromatogram>

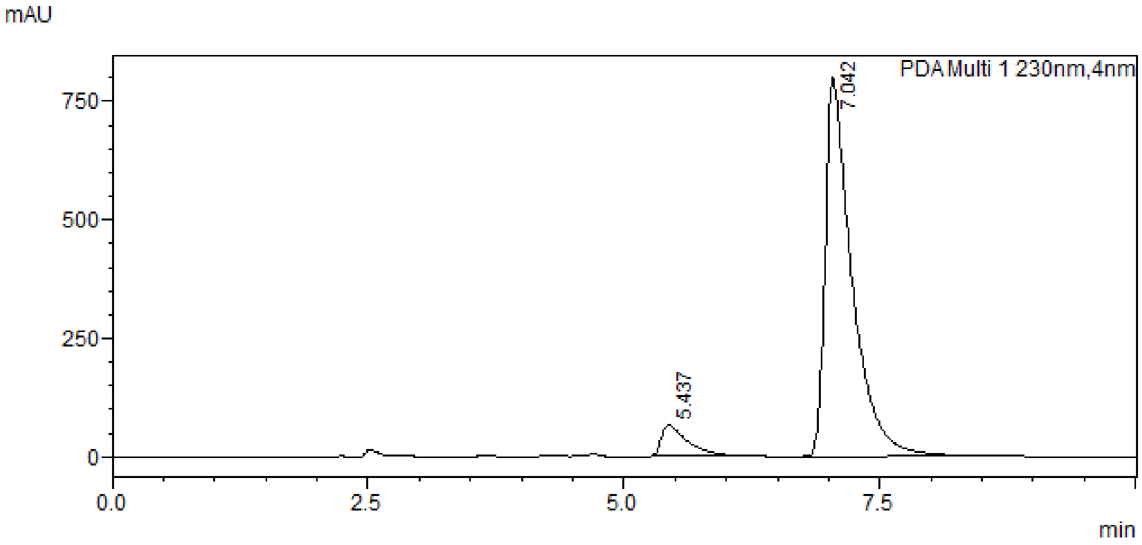

<Peak Table>

PDA Ch1 230nm

Peak#	Ret. Time	Area	Height	Conc.	Unit	Mark	Name
1	5.437	1094038	64511	7.033		M	
2	7.042	14462702	799111	92.967		M	
Total		15556740	863622				

<Chromatogram>

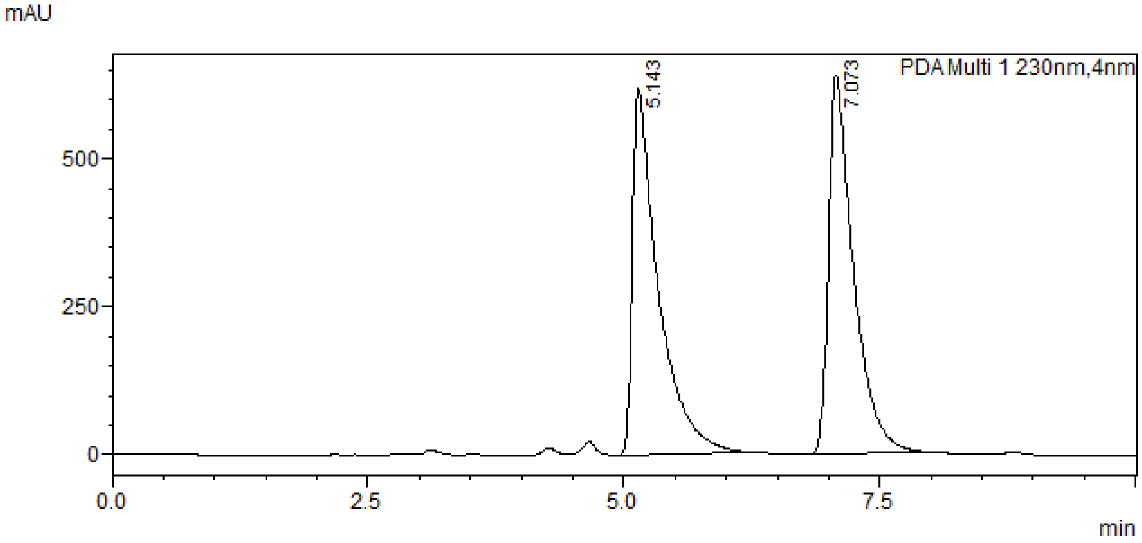

<Peak Table>

PDA Ch1 230nm

Peak#	Ret. Time	Area	Height	Conc.	Unit	Mark	Name
1	5.143	10861019	618888	50.068		M	
2	7.073	10831551	637585	49.932		M	
Total		21692571	1256473				

(1*S*,2*S*)-2-azido-*N*-benzyl-*N*-methyl-1,2-diphenylethan-1-amine (3i)

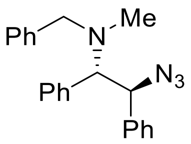

<Chromatogram>

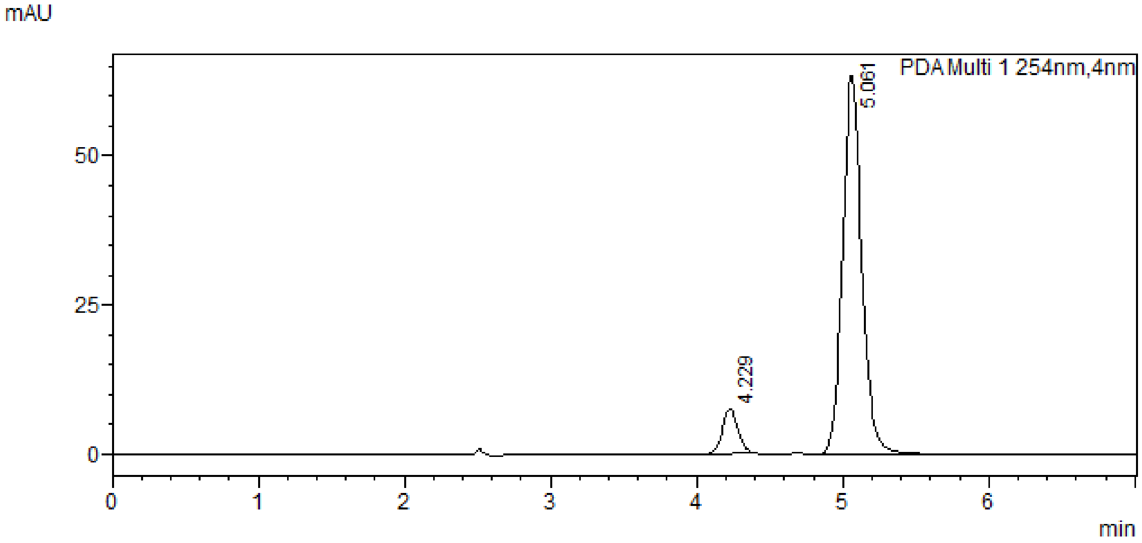

<Peak Table>

PDA Ch1 254nm

Peak#	Ret. Time	Area	Height	Conc.	Unit	Mark	Name
1	4.229	55878	7596	8.798		M	
2	5.061	579243	63247	91.202		M	
Total		635122	70842				

<Chromatogram>

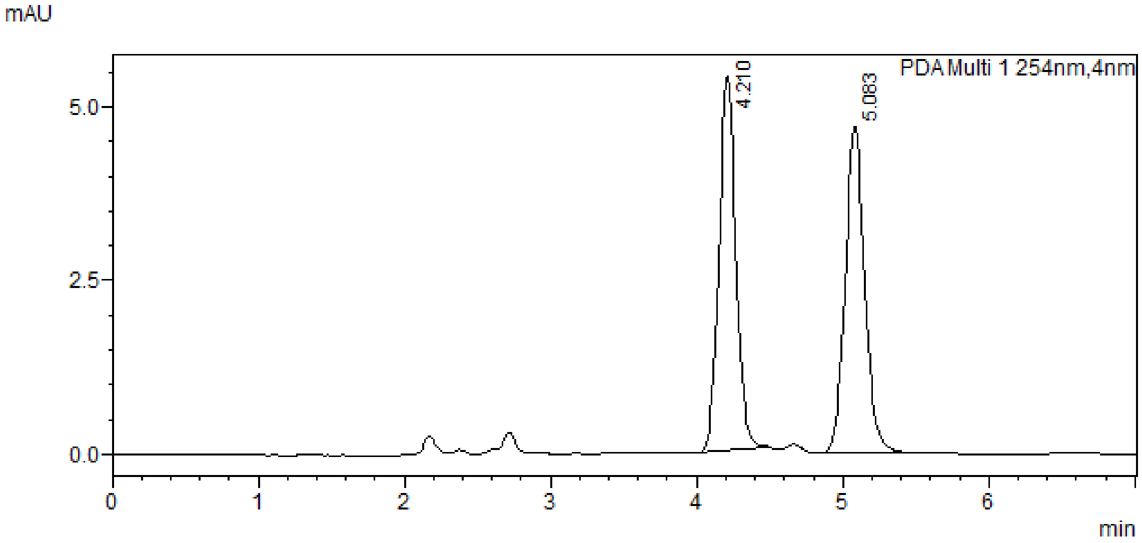

<Peak Table>

PDA Ch1 254nm

Peak#	Ret. Time	Area	Height	Conc.	Unit	Mark	Name
1	4.210	43395	5382	49.754		M	
2	5.083	43824	4697	50.246		M	
Total		87219	10079				

(1*S*,2*S*)-2-azido-*N*,*N*-dimethyl-1,2-diphenylethan-1-amine (3j)

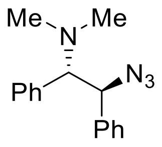

<Chromatogram>

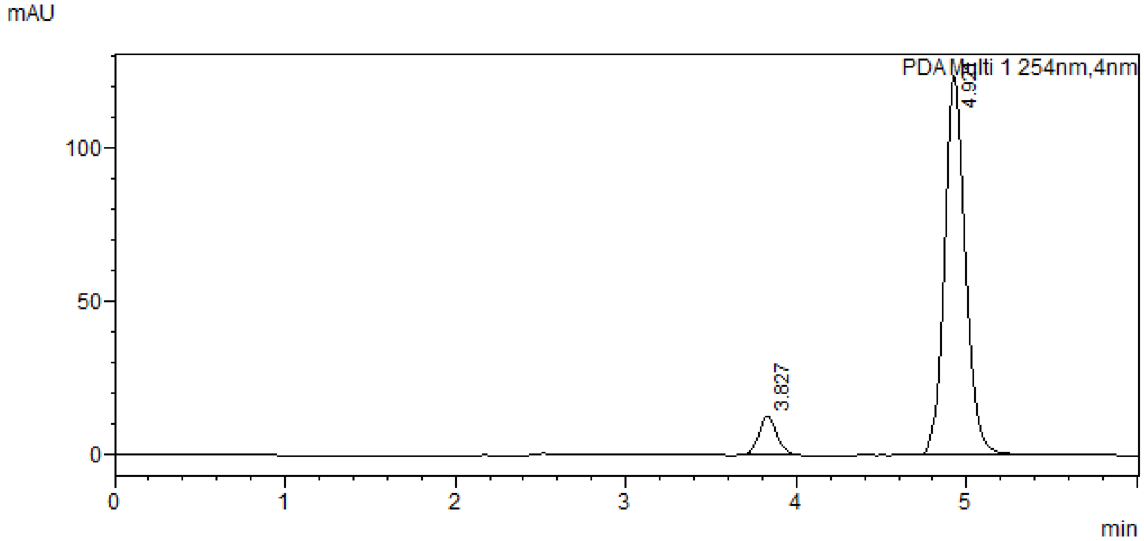

<Peak Table>

PDA Ch1 254nm

Peak#	Ret. Time	Area	Height	Conc.	Unit	Mark	Name
1	3.827	91739	12693	8.048		M	
2	4.921	1048220	123144	91.952		M	
Total		1139959	135836				

<Chromatogram>

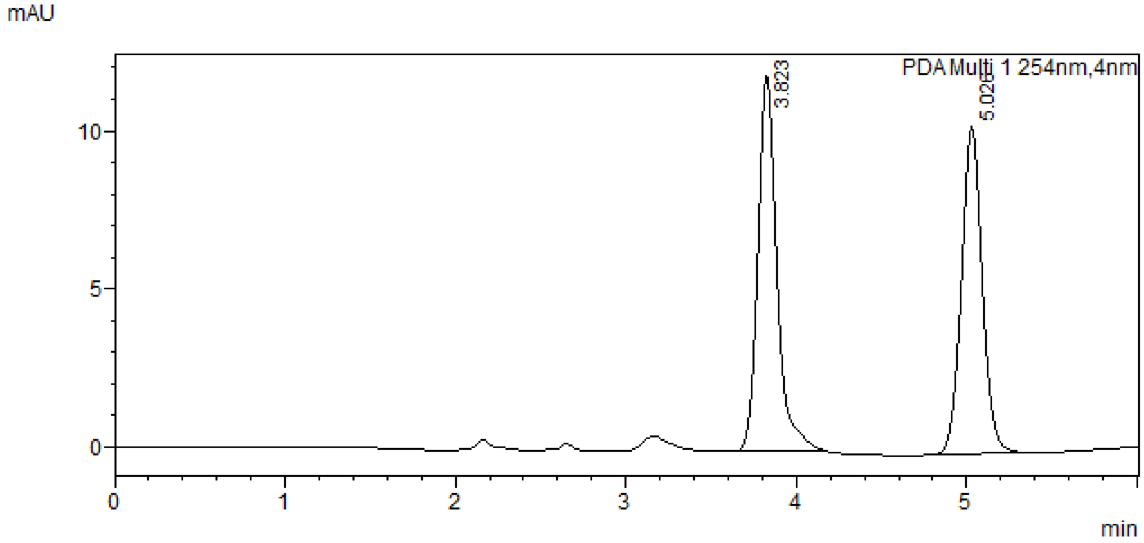

<Peak Table>

PDA Ch1 254nm

Peak#	Ret. Time	Area	Height	Conc.	Unit	Mark	Name
1	3.823	92612	11861	51.108		M	
2	5.026	88596	10368	48.892		M	
Total		181208	22229				

1-((1*S*,2*S*)-2-azido-1,2-diphenylethyl)azepane (3k)

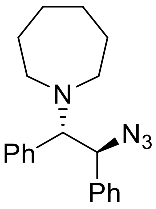

<Chromatogram>

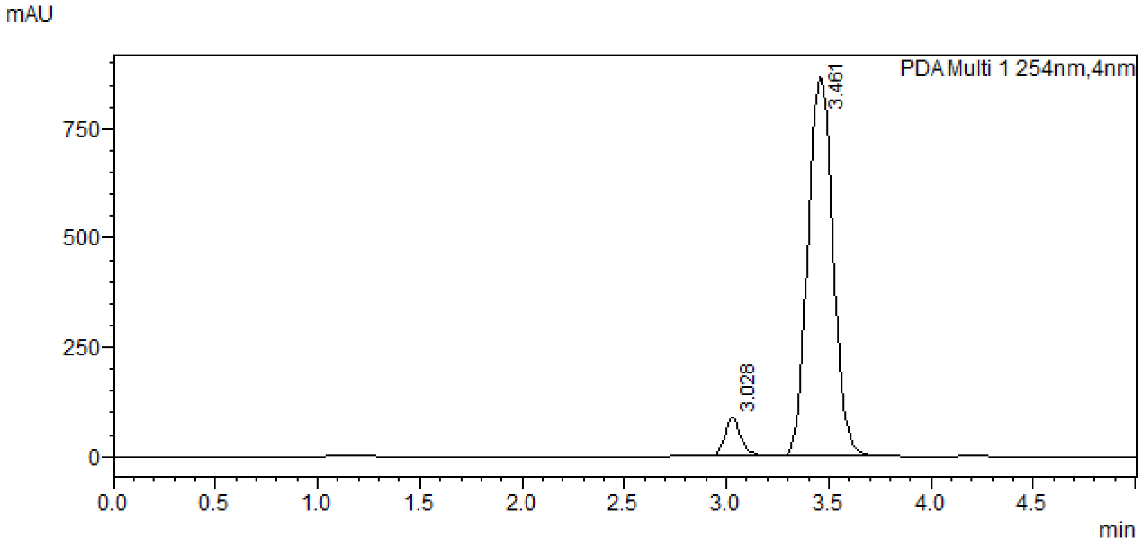

<Peak Table>

PDA Ch1 254nm

Peak#	Ret. Time	Area	Height	Conc.	Unit	Mark	Name
1	3.028	466904	88351	5.932		M	
2	3.461	7404273	866411	94.068		M	
Total		7871176	954762				

<Chromatogram>

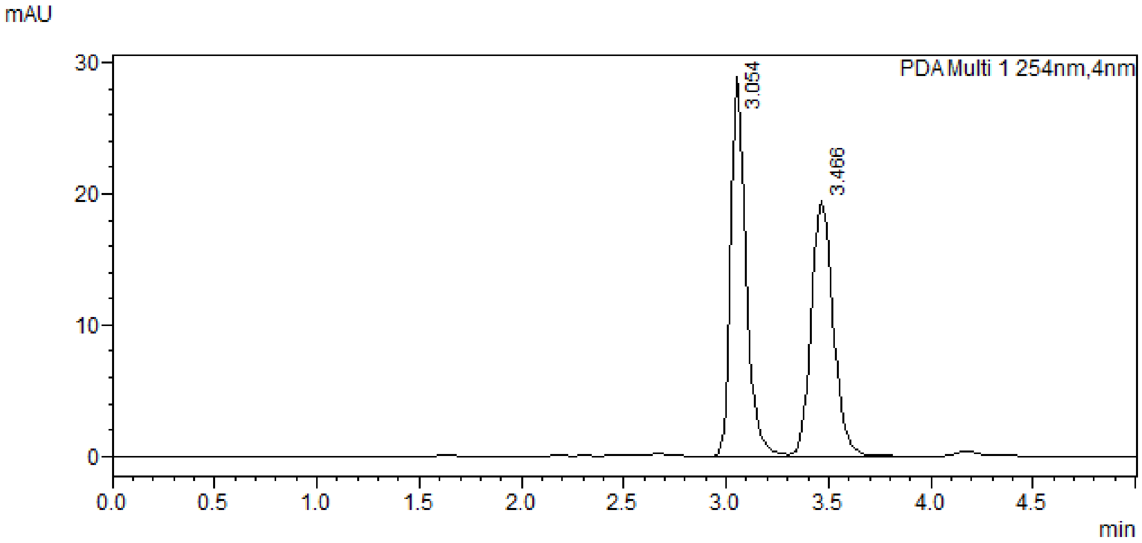

<Peak Table>

PDA Ch1 254nm

Peak#	Ret. Time	Area	Height	Conc.	Unit	Mark	Name
1	3.054	147842	28884	50.453			
2	3.466	145185	19496	49.547		V	
Total		293027	48380				

1-((1*S*,2*S*)-2-azido-1,2-bis(3-fluorophenyl)ethyl)piperidine (3l)

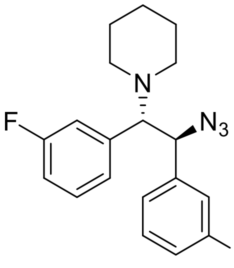

<Chromatogram>

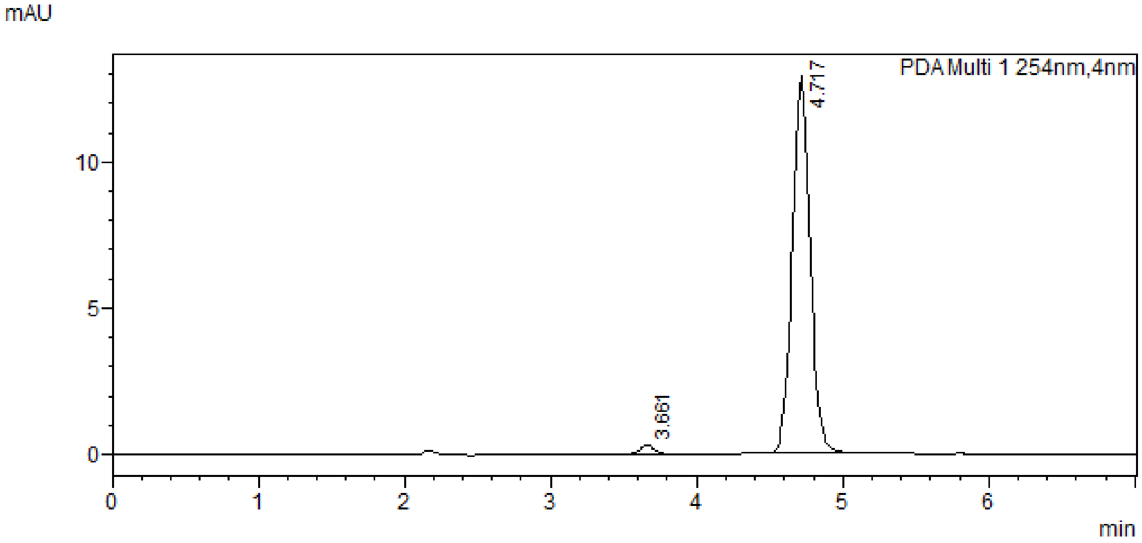

<Peak Table>

PDA Ch1 254nm

Peak#	Ret. Time	Area	Height	Conc.	Unit	Mark	Name
1	3.661	2238	309	1.995			
2	4.717	109903	12890	98.005			
Total		112141	13199				

<Chromatogram>

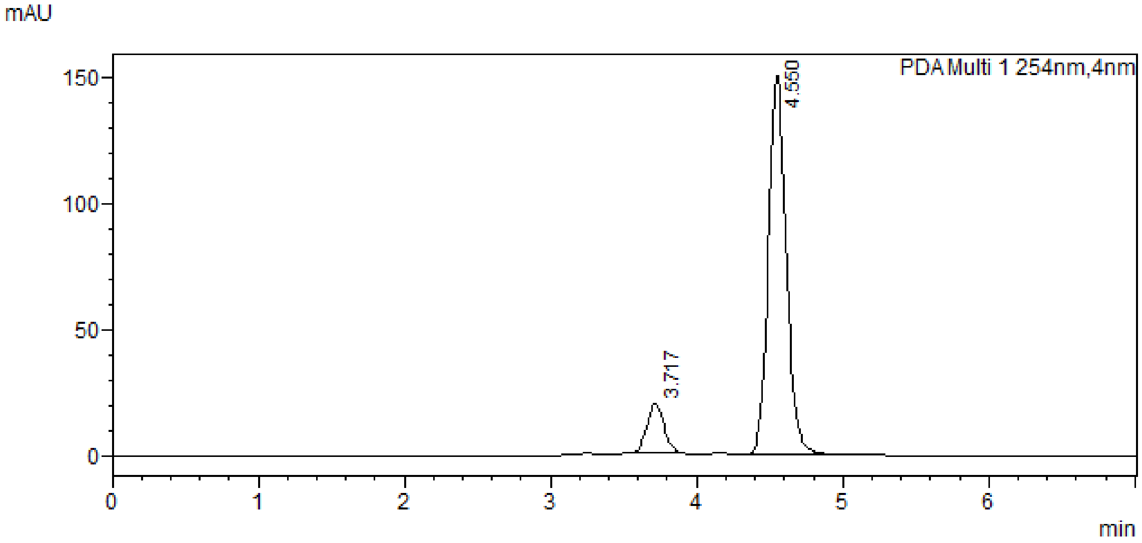

<Peak Table>

PDA Ch1 254nm

Peak#	Ret. Time	Area	Height	Conc.	Unit	Mark	Name
1	3.717	156937	19279	10.951		M	
2	4.550	1276178	150154	89.049		M	
Total		1433115	169432				

<Chromatogram>

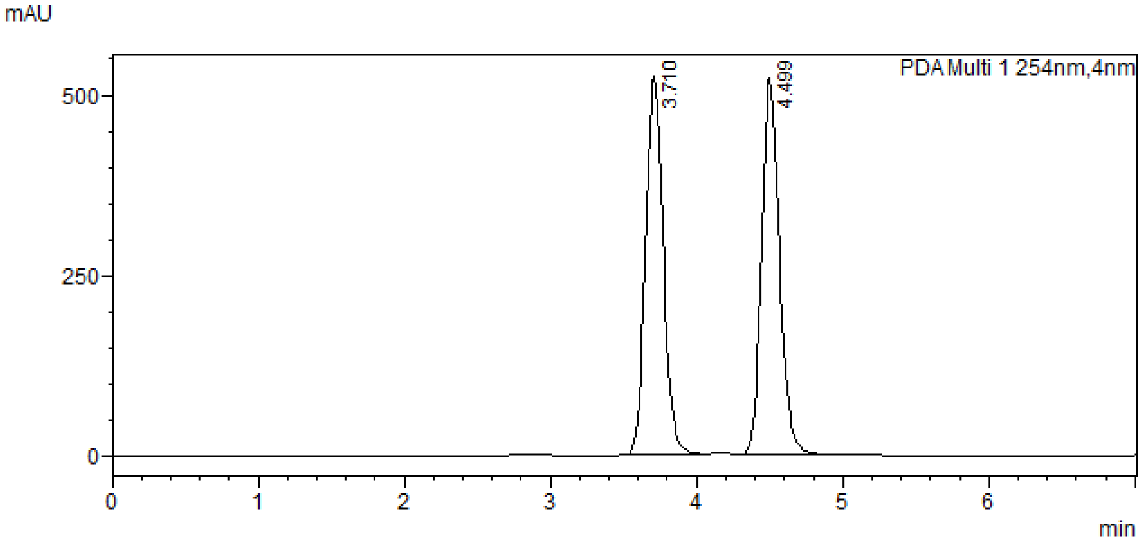

<Peak Table>

PDA Ch1 254nm

Peak#	Ret. Time	Area	Height	Conc.	Unit	Mark	Name
1	3.710	4407130	523895	49.896		M	
2	4.499	4425425	522418	50.104		M	
Total		8832555	1046314				

1-((1*S*,2*S*)-2-azido-1,2-bis(3-chlorophenyl)ethyl)piperidine (3m)

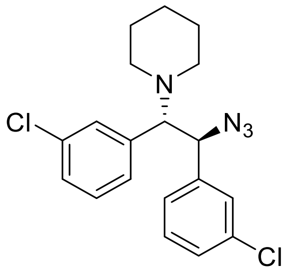

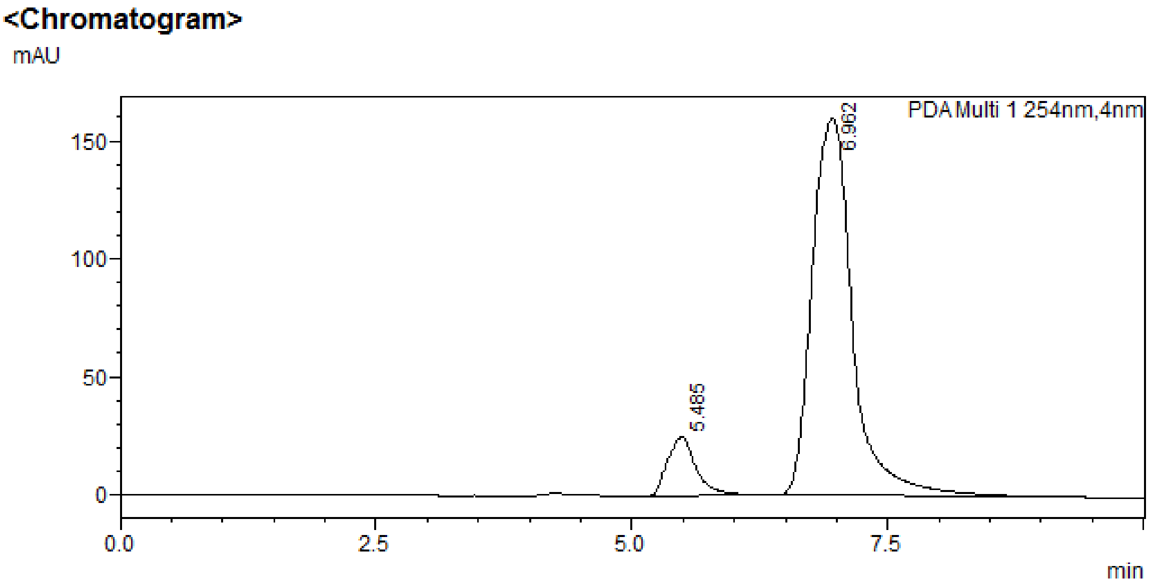

<Peak Table>

PDA Ch1 254nm

Peak#	Ret. Time	Area	Height	Conc.	Unit	Mark	Name
1	5.485	475896	24962	9.818		M	
2	6.962	4371273	159433	90.182		M	
Total		4847168	184395				

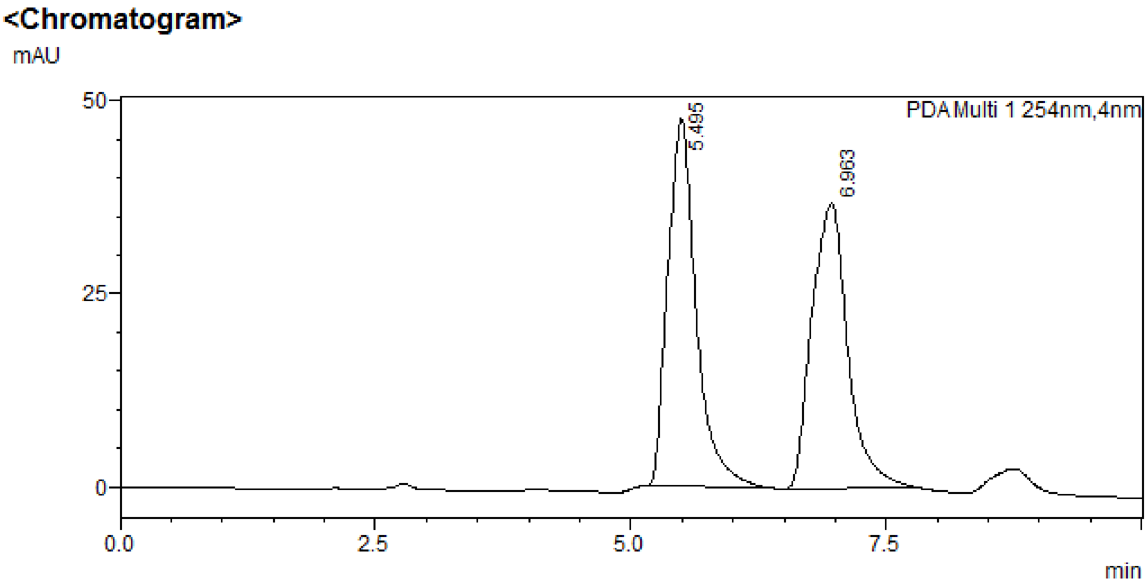

<Peak Table>

PDA Ch1 254nm

Peak#	Ret. Time	Area	Height	Conc.	Unit	Mark	Name
1	5.495	917216	47559	50.473		M	
2	6.963	900016	36860	49.527		M	
Total		1817232	84419				

1-((1*S*,2*S*)-2-azido-1,2-bis(3-(trifluoromethyl)phenyl)ethyl)piperidine (3n)

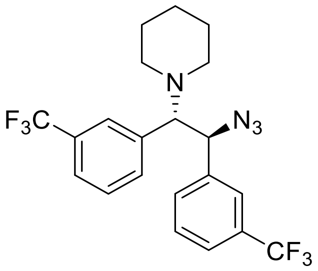

<Chromatogram>

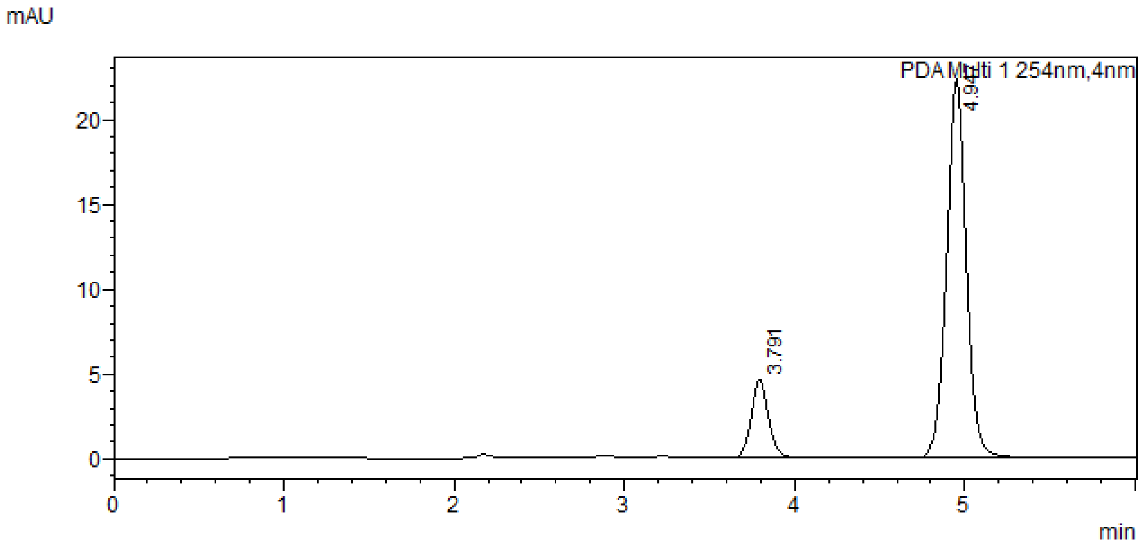

<Peak Table>

PDA Ch1 254nm

Peak#	Ret. Time	Area	Height	Conc.	Unit	Mark	Name
1	3.791	31848	4573	15.037		M	
2	4.947	179948	22322	84.963		M	
Total		211796	26896				

<Chromatogram>

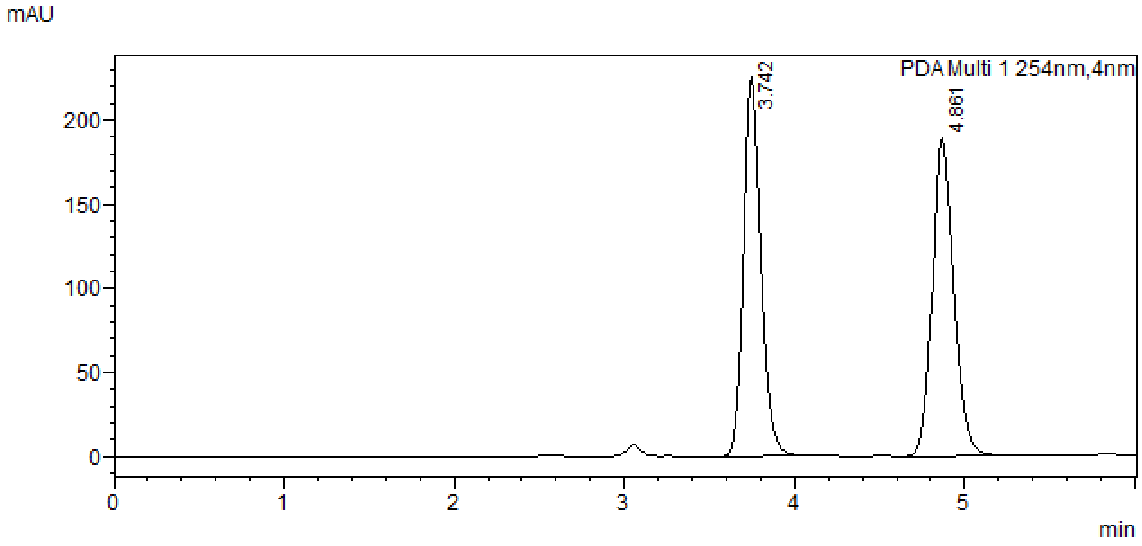

<Peak Table>

PDA Ch1 254nm

Peak#	Ret. Time	Area	Height	Conc.	Unit	Mark	Name
1	3.742	1603875	225043	49.852		M	
2	4.861	1613375	189384	50.148		M	
Total		3217250	414426				

1-((1*S*,2*S*)-2-azido-1,2-bis(4-bromophenyl)ethyl)piperidine (3o)

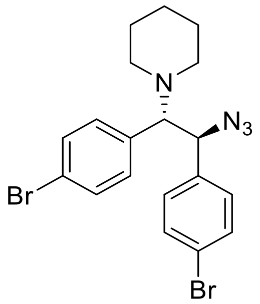

<Chromatogram>

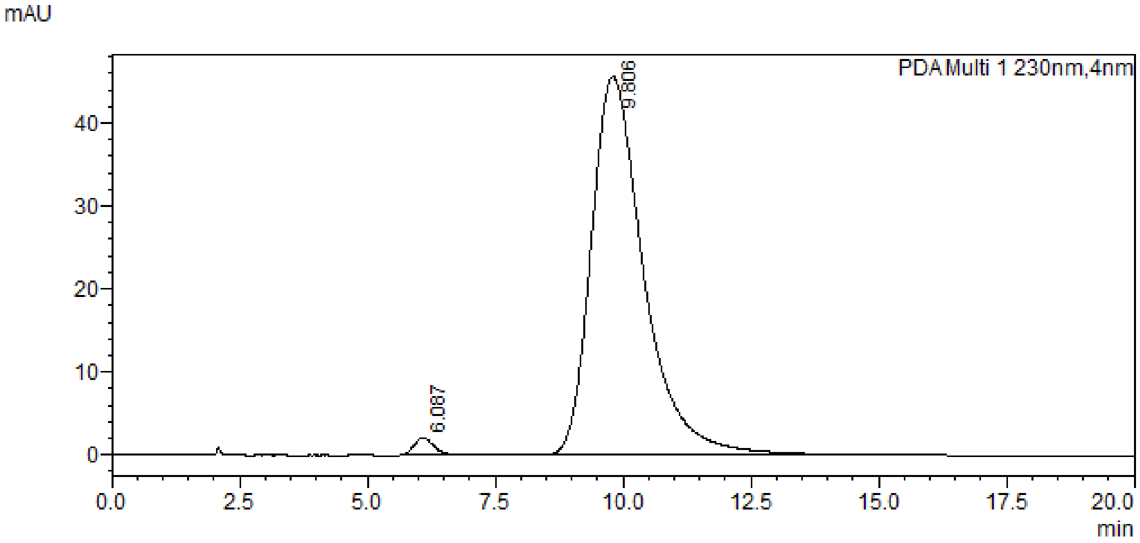

<Peak Table>

PDA Ch1 230nm

Peak#	Ret. Time	Area	Height	Conc.	Unit	Mark	Name
1	6.087	54170	2065	1.624		M	
2	9.806	3280472	45643	98.376		M	
Total		3334643	47708				

<Chromatogram>

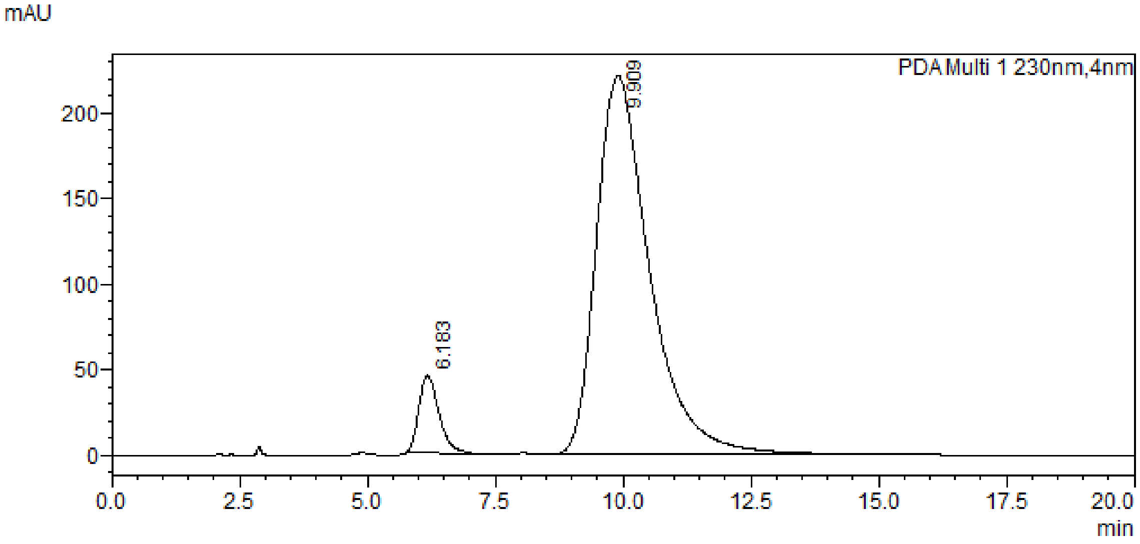

<Peak Table>

PDA Ch1 230nm

Peak#	Ret. Time	Area	Height	Conc.	Unit	Mark	Name
1	6.183	1232498	45154	7.130		M	
2	9.909	16053974	220784	92.870		M	
Total		17286472	265938				

<Chromatogram>

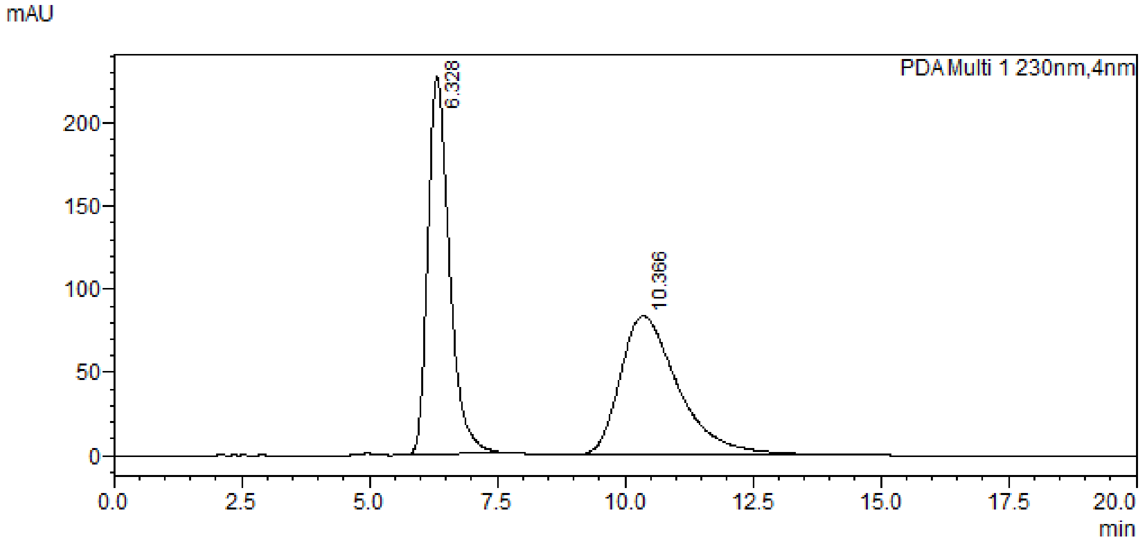

<Peak Table>

PDA Ch1 230nm

Peak#	Ret. Time	Area	Height	Conc.	Unit	Mark	Name
1	6.328	6660861	226580	50.214		M	
2	10.366	6604107	83242	49.786		M	
Total		13264968	309822				

1-((1*S*,2*S*)-2-azido-1,2-bis(4-(*tert*-butyl)phenyl)ethyl)piperidine (3p)

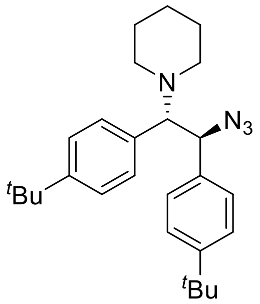

<Chromatogram>

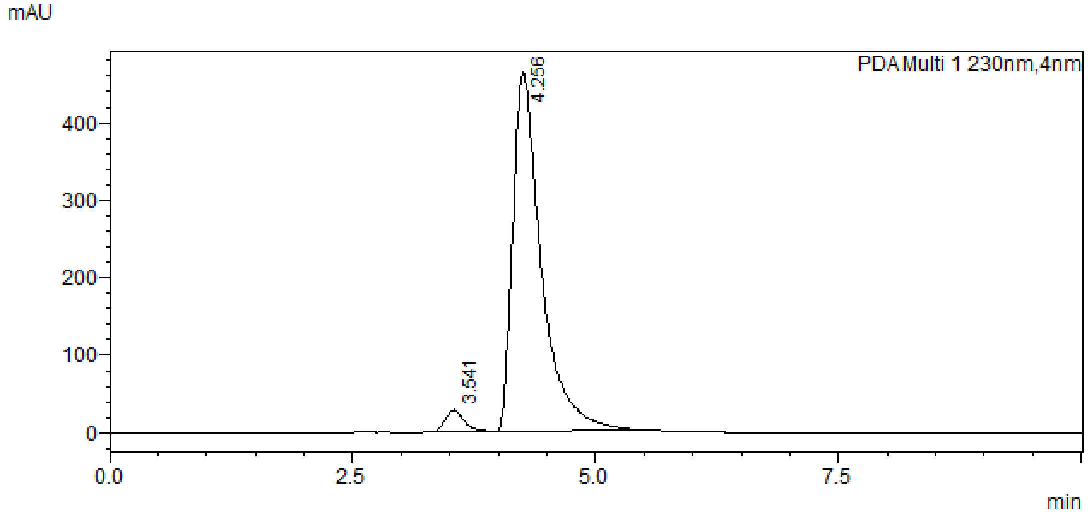

<Peak Table>

PDA Ch1 230nm

Peak#	Ret. Time	Area	Height	Conc.	Unit	Mark	Name
1	3.541	369419	27844	3.767		M	
2	4.256	9437230	462594	96.233		M	
Total		9806648	490438				

<Chromatogram>

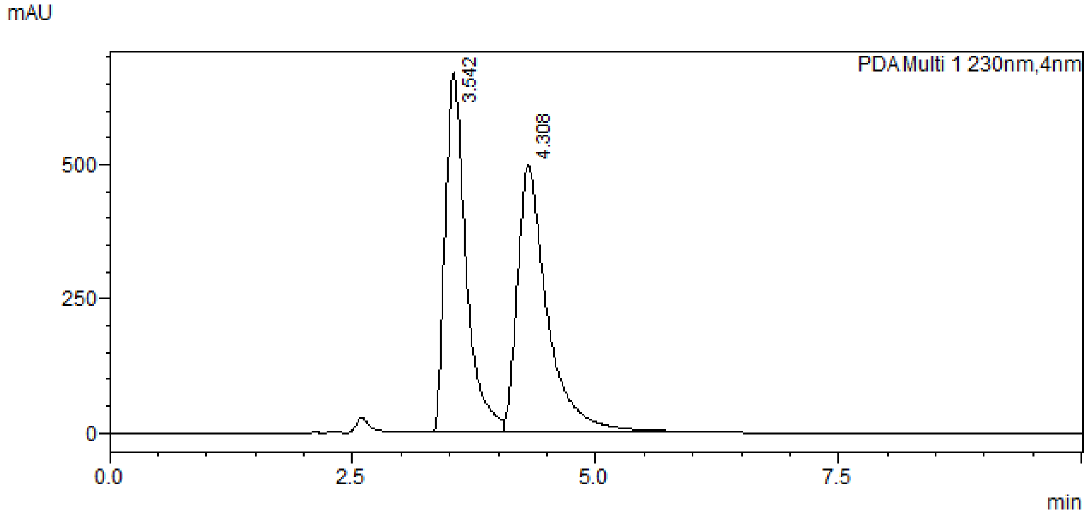

<Peak Table>

PDA Ch1 230nm

Peak#	Ret. Time	Area	Height	Conc.	Unit	Mark	Name
1	3.542	9940923	669074	48.345		M	
2	4.308	10621471	497446	51.655		V M	
Total		20562394	1166520				

1-((1*S*,2*S*)-2-azido-1,2-bis(4-(trifluoromethyl)phenyl)ethyl)piperidine (3q)

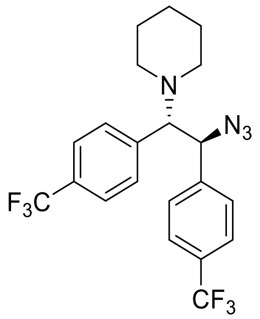

<Chromatogram>

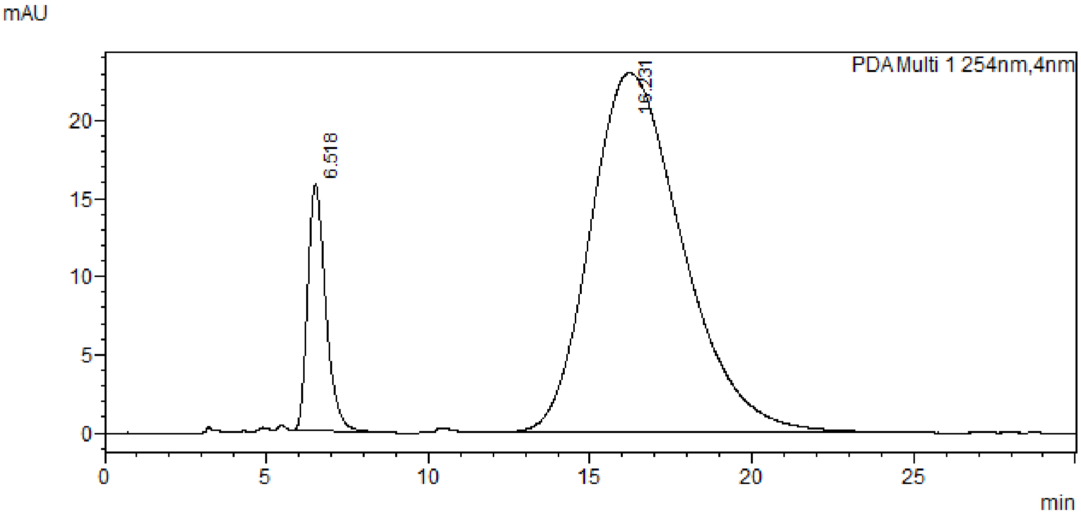

<Peak Table>

PDA Ch1 254nm

Peak#	Ret. Time	Area	Height	Conc.	Unit	Mark	Name
1	6.518	612787	15804	11.878			
2	16.231	4546028	22969	88.122		M	
Total		5158815	38773				

<Chromatogram>

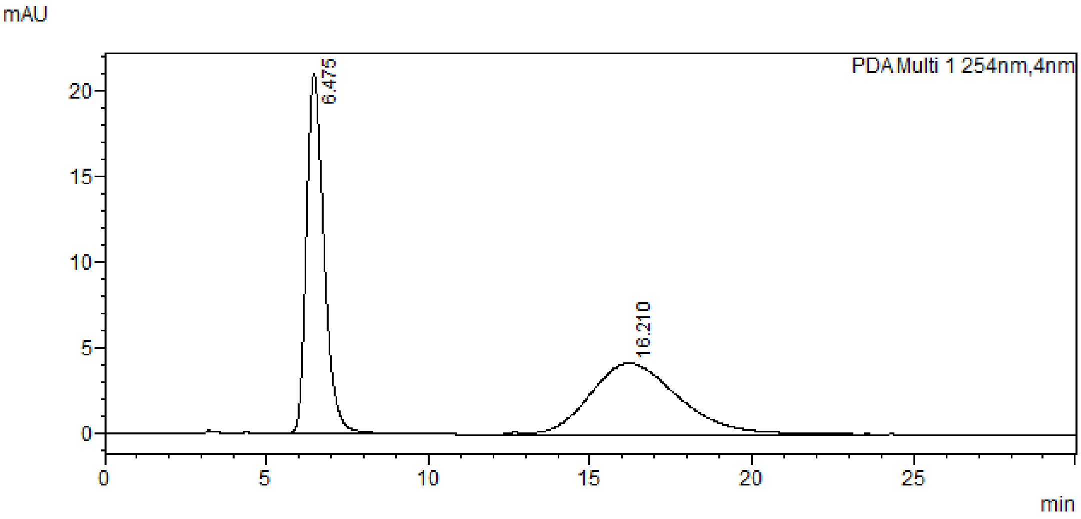

<Peak Table>

PDA Ch1 254nm

Peak#	Ret. Time	Area	Height	Conc.	Unit	Mark	Name
1	6.475	787631	20983	49.808		M	
2	16.210	793693	4158	50.192		M	
Total		1581324	25141				

_N_-((1_R_,2_R_)-2-azido-1,2-di(pyridin-2-yl)ethyl)-_N_-propylpropan-1-amine (3r)

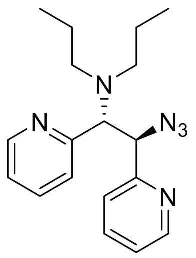

<Chromatogram>

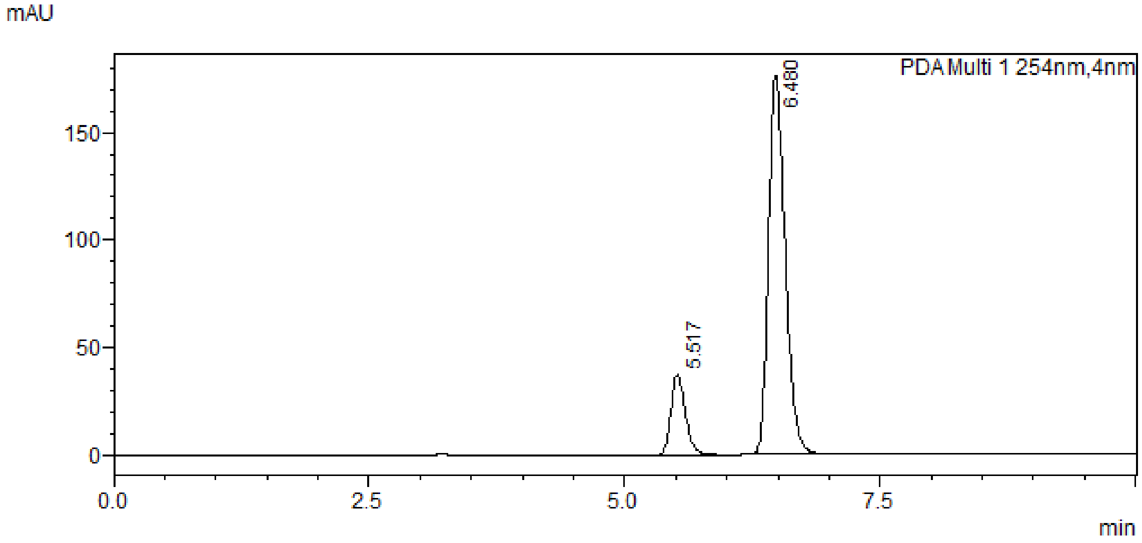

<Peak Table>

PDA Ch1 254nm

Peak#	Ret. Time	Area	Height	Conc.	Unit	Mark	Name
1	5.517	371601	37595	15.725		M	
2	6.480	1991584	176045	84.275		M	
Total		2363184	213640				

<Chromatogram>

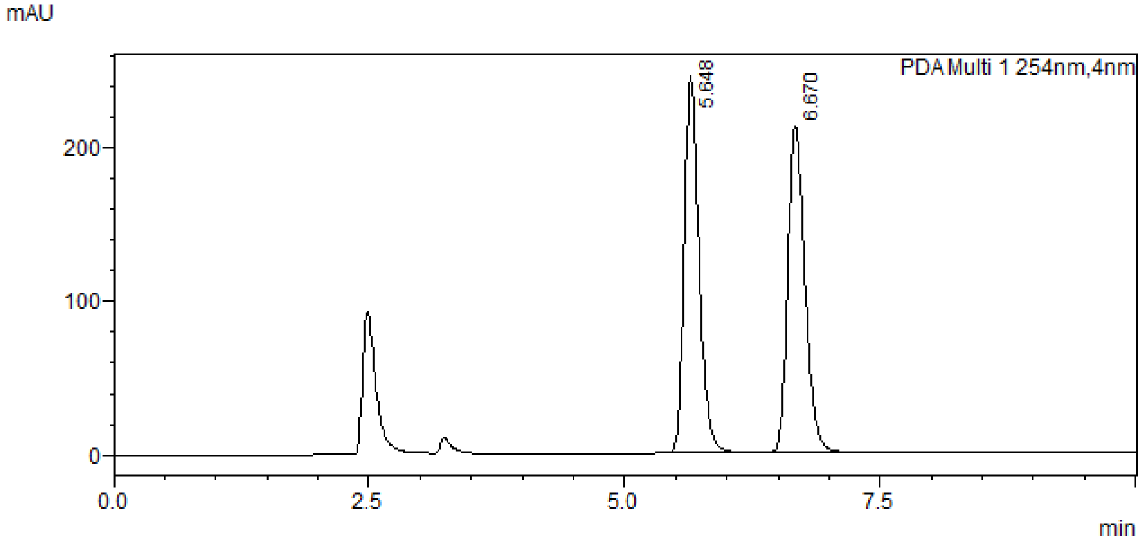

<Peak Table>

PDA Ch1 254nm

Peak#	Ret. Time	Area	Height	Conc.	Unit	Mark	Name
1	5.648	2486858	244932	50.008		M	
2	6.670	2486017	211578	49.992		M	
Total		4972875	456509				

2-azido-*N*-benzyl-*N*-methylcyclohexan-1-amine (3s)

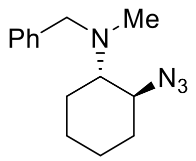

<Chromatogram>

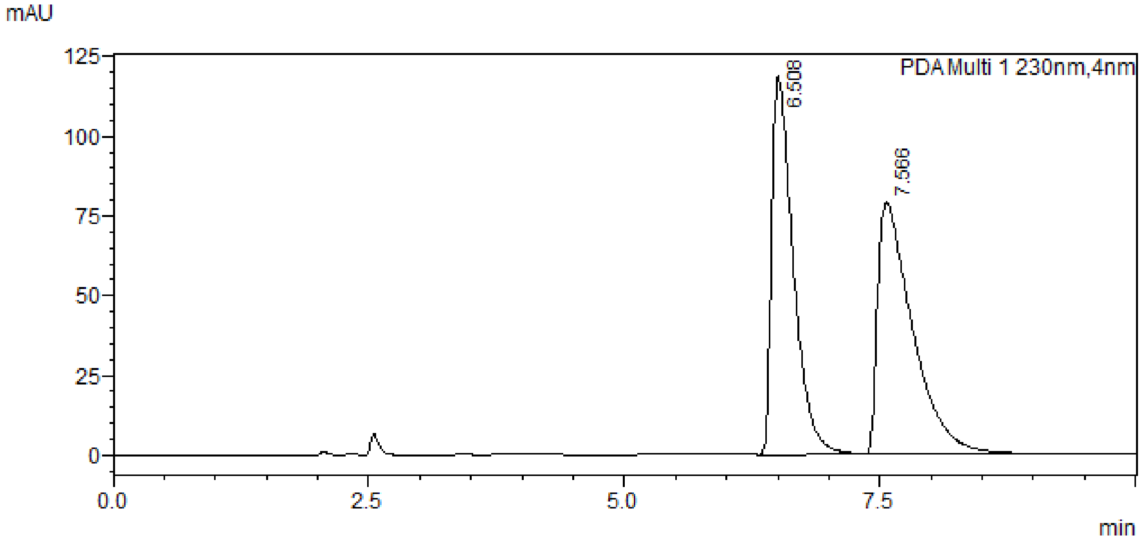

<Peak Table>

PDA Ch1 230nm

Peak#	Ret. Time	Area	Height	Conc.	Unit	Mark	Name
1	6.508	1730861	118657	48.151		M	
2	7.566	1863793	78731	51.849		M	
Total		3594654	197388				

<Chromatogram>

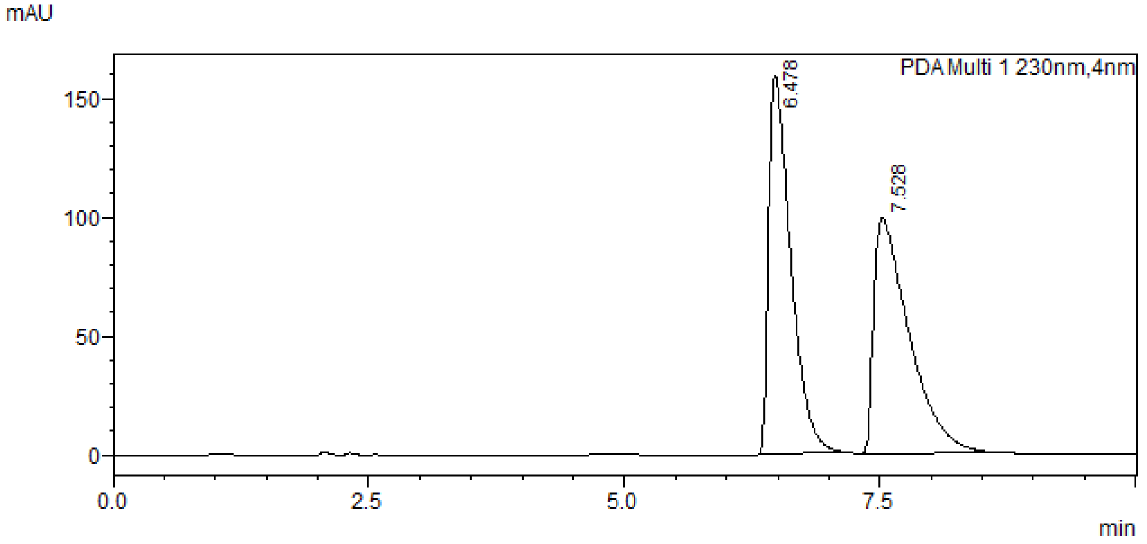

<Peak Table>

PDA Ch1 230nm

Peak#	Ret. Time	Area	Height	Conc.	Unit	Mark	Name
1	6.478	2401209	158981	50.059		M	
2	7.528	2395572	98624	49.941		M	
Total		4796781	257605				

(1*S*,2*S*)-1,2-diphenyl-2-(piperidin-1-yl)ethan-1-amine

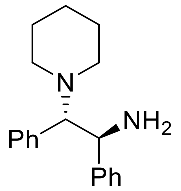

<Chromatogram>

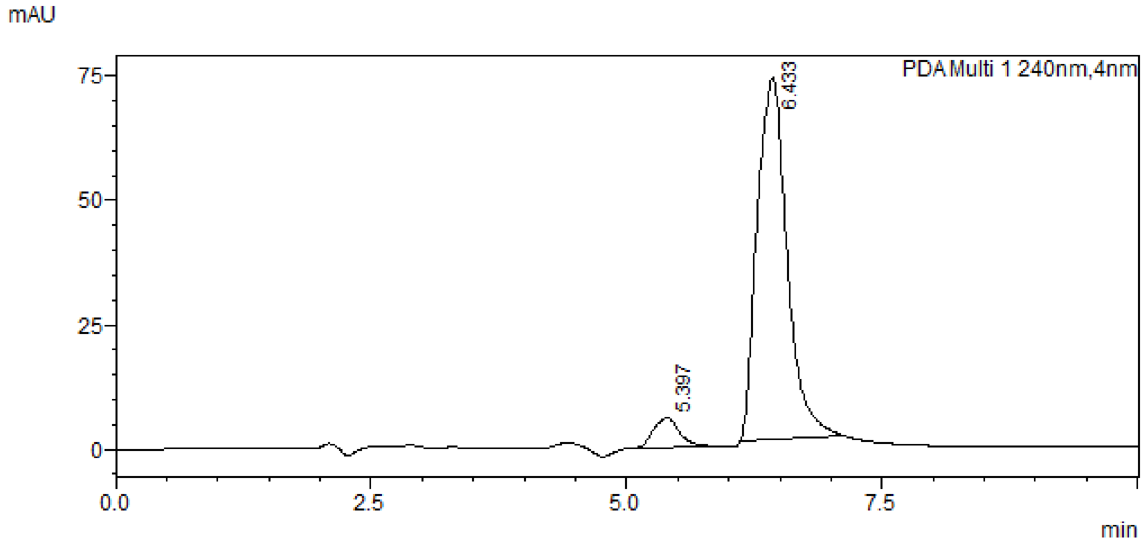

<Peak Table>

PDA Ch1 240nm

Peak#	Ret. Time	Area	Height	Conc.	Unit	Mark	Name
1	5.397	99773	5851	6.253		M	
2	6.433	1495802	72853	93.747		M	
Total		1595575	78704				

<Chromatogram>

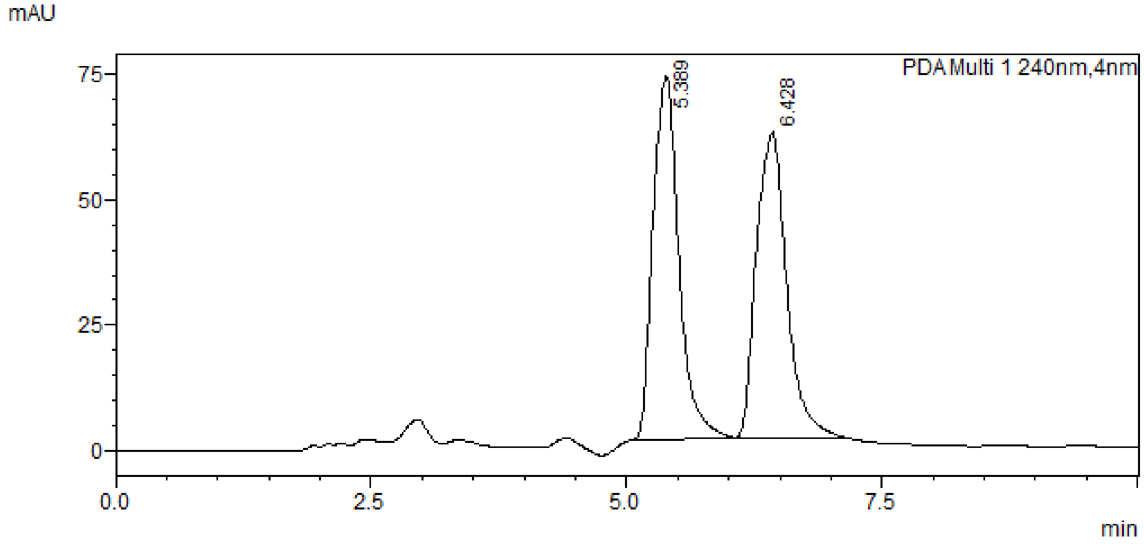

<Peak Table>

PDA Ch1 240nm

Peak#	Ret. Time	Area	Height	Conc.	Unit	Mark	Name
1	5.389	1269201	72378	50.071		M	
2	6.428	1265610	60969	49.929		M	
Total		2534812	133347				

References

(S1) Gottlieb, H. E.; Kotlyar, V.; Nudelman, A. NMR Chemical Shifts of Common Laboratory Solvents as Trace Impurities. *J. Org. Chem.* **1997**, *62* (21), 7512–7515.

(S2) *Prudent Practices for Disposal of Chemicals From Laboratories*; National Acadamy Press: Washington, D.C., 1983.

(S3) Stedman, G. 590. Mechanism of the Azide–Nitrite Reaction. Part I. *J. Chem. Soc.* **1959**, No. 2, 2943–2949.

(S4) Jakab, G.; Tancon, C.; Zhang, Z.; Lippert, K. M.; Schreiner, P. R. (Thio)Urea Organocatalyst Equilibrium Acidities in DMSO. *Org. Lett.* **2012**, *14* (7), 1724–1727.

(S5) Perveen, S.; Abdul Hai, S. M.; Khan, R. A.; Khan, K. M.; Afza, N.; Sarfaraz, T. B. Expeditious Method for Synthesis of Symmetrical 1,3-Disubstituted Ureas and Thioureas. *Synth. Commun.* **2005**, *35* (12), 1663–1674.

(S6) Pfeifer, L.; Engle, K. M.; Pidgeon, G. W.; Sparkes, H. A.; Thompson, A. L.; Brown, J. M.; Gouverneur, V. Hydrogen-Bonded Homoleptic Fluoride–Diarylurea Complexes: Structure, Reactivity, and Coordinating Power. *J. Am. Chem. Soc.* **2016**, *138* (40), 13314–13325.

(S7) Sauer, B.; Skinner-Adams, T. S.; Bouchut, A.; Chua, M. J.; Pierrot, C.; Erdmann, F.; Robaa, D.; Schmidt, M.; Khalife, J.; Andrews, K. T.; Sippl, W. Synthesis, Biological Characterisation and Structure Activity Relationships of Aromatic Bisamidines Active against Plasmodium Falciparum. *Eur. J. Med. Chem.* **2017**, *127*, 22–40.

(S8) Pupo, G.; Ibba, F.; Ascough, D. M. H.; Vicini, A. C.; Ricci, P.; Christensen, K. E.; Pfeifer, L.; Morphy, J. R.; Brown, J. M.; Paton, R. S.; Gouverneur, V. Asymmetric Nucleophilic Fluorination under Hydrogen Bonding Phase-Transfer Catalysis. *Science* **2018**, *360* (6389), 638–642.

(S9) Hendrickson, J. B.; Judelson, D. A.; Chancellor, T. A Simple Preparation of Anhydrous Trifluoromethanesulfinate (Triflinate) Salts and Synthesis of Trifluoromethyl Sulfones (Triflones). *Synthesis (Stuttg).* **1984**, *1984* (04), 320–322.

(S10) Ibba, F.; Pupo, G.; Thompson, A. L.; Brown, J. M.; Claridge, T. D. W.; Gouverneur, V. Impact of Multiple Hydrogen Bonds with Fluoride on Catalysis: Insight from NMR Spectroscopy. *J. Am. Chem. Soc.* **2020**, *142* (46), 19731–19744.

(S11) supramolecular.org http://supramolecular.org (accessed Sep 18, 2019).

(S12) Thordarson, P. Determining Association Constants from Titration Experiments in Supramolecular Chemistry. *Chem. Soc. Rev.* **2011**, *40* (3), 1305–1323.

(S13) Howe, E. N. W.; Bhadbhade, M.; Thordarson, P. Cooperativity and Complexity in the Binding

of Anions and Cations to a Tetratopic Ion-Pair Host. *J. Am. Chem. Soc.* **2014**, *136* (20), 7505–7516.

(S14) Brynn Hibbert, D.; Thordarson, P. The Death of the Job Plot, Transparency, Open Science and Online Tools, Uncertainty Estimation Methods and Other Developments in Supramolecular Chemistry Data Analysis. *Chem. Commun.* **2016**, *52* (87), 12792–12805.

(S15) Pupo, G.; Vicini, A. C.; Ascough, D. M. H.; Ibba, F.; Christensen, K. E.; Thompson, A. L.; Brown, J. M.; Paton, R. S.; Gouverneur, V. Hydrogen Bonding Phase-Transfer Catalysis with Potassium Fluoride: Enantioselective Synthesis of β-Fluoroamines. *J. Am. Chem. Soc.* **2019**, *141* (7), 2878–2883.

(S16) Broghammer, F.; Brodbeck, D.; Junge, T.; Peters, R. Cooperative Lewis Acid–Onium Salt Catalysis as Tool for the Desymmetrization of Meso-Epoxides. *Chem. Commun.* **2017**, *53* (6), 1156–1159.

(S17) Pu, X.; Qi, X.; Ready, J. M. Allenes in Asymmetric Catalysis: Asymmetric Ring Opening of Meso -Epoxides Catalyzed by Allene-Containing Phosphine Oxides. *J. Am. Chem. Soc.* **2009**, *131* (30), 10364–10365.

(S18) Kokubo, M.; Naito, T.; Kobayashi, S. Chiral Zinc(II) and Copper(II)-Catalyzed Asymmetric Ring-Opening Reactions of Meso-Epoxides with Aniline and Indole Derivatives. *Tetrahedron* **2010**, *66* (5), 1111–1118.

(S19) Ramesh, E.; Guntreddi, T.; Sahoo, A. K. AlCl3-Catalyzed Intermolecular Annulation of Thiol Derivatives and Alkynes by 1,2-Sulfur Migration: Construction of 6-Substituted Benzo[b]Thiophenes. *European J. Org. Chem.* **2017**, *2017* (30), 4405–4413.

(S20) Mio, M. J.; Kopel, L. C.; Braun, J. B.; Gadzikwa, T. L.; Hull, K. L.; Brisbois, R. G.; Markworth, C. J.; Grieco, P. A. One-Pot Synthesis of Symmetrical and Unsymmetrical Bisarylethynes by a Modification of the Sonogashira Coupling Reaction. *Org. Lett.* **2002**, *4* (19), 3199–3202.

(S21) Hong, S.; Tian, S.; Metz, M. V; Marks, T. J. C 2 -Symmetric Bis(Oxazolinato)Lanthanide Catalysts for Enantioselective Intramolecular Hydroamination/Cyclization. *J. Am. Chem. Soc.* **2003**, *125* (48), 14768–14783.

(S22) Wang, S.; Lokesh, N.; Hioe, J.; Gschwind, R. M.; König, B. Photoinitiated Carbonyl-Metathesis: Deoxygenative Reductive Olefination of Aromatic Aldehydes via Photoredox Catalysis. *Chem. Sci.* **2019**, *10* (17), 4580–4587.

(S23) Kajanus, J.; Antonsson, T.; Carlsson, L.; Jurva, U.; Pettersen, A.; Sundell, J.; Inghardt, T. Potassium Channel Blocking 1,2-Bis(Aryl)Ethane-1,2-Diamines Active as Antiarrhythmic Agents. *Bioorg. Med. Chem. Lett.* **2019**, *29* (10), 1241–1245.

(S24) González-Sabín, J.; Gotor, V.; Rebolledo, F. Chemoenzymatic Preparation of Optically Active β-Amino-Cyclohexanols and Their Application in the Enantioselective Addition of Diethylzinc to Benzaldehyde. *Tetrahedron: Asymmetry* **2004**, *15* (8), 1335–1341.

(S25) Li, J.; Fu, N.; Zhang, L.; Zhou, P.; Luo, S.; Cheng, J.-P. Chiral Primary Amine Catalyzed Asymmetric Epoxidation of α-Substituted Acroleins. *European J. Org. Chem.* **2010**, *2010* (35), 6840–6849.

(S26) Hamilton, G. L.; Kanai, T.; Toste, F. D. Chiral Anion-Mediated Asymmetric Ring Opening of Meso-Aziridinium and Episulfonium Ions. *J. Am. Chem. Soc.* **2008**, *130* (45), 14984–14986.

(S27) Wang, Z. X. An Exact Mathematical Expression for Describing Competitive Binding of Two Different Ligands to a Protein Molecule. *FEBS Lett.* **1995**, *360* (2), 111–114.

(S28) Frisch, M. J.; Trucks, G. W.; Schlegel, H. B.; Scuseria, G. E.; Robb, M. A.; Cheeseman, J. R.; Scalmani, G.; Barone, V.; Mennucci, B.; Petersson, G. A.; et al. Gaussian 09, Revision D.01. Gaussian, Inc., Wallingford CT 2009.

(S29) Zhao, Y.; Truhlar, D. G. The M06 Suite of Density Functionals for Main Group Thermochemistry, Thermochemical Kinetics, Noncovalent Interactions, Excited States, and Transition Elements: Two New Functionals and Systematic Testing of Four M06 Functionals and 12 Other Functionals. *Theor. Chem. Acc.* **2008**, *119* (5–6), 525–525.

(S30) Weigend, F.; Ahlrichs, R. Balanced Basis Sets of Split Valence, Triple Zeta Valence and Quadruple Zeta Valence Quality for H to Rn: Design and Assessment of Accuracy. *Phys. Chem. Chem. Phys.* **2005**, *7* (18), 3297.

(S31) Hellweg, A.; Rappoport, D. Development of New Auxiliary Basis Functions of the Karlsruhe Segmented Contracted Basis Sets Including Diffuse Basis Functions (Def2-SVPD, Def2-TZVPPD, and Def2-QVPPD) for RI-MP2 and RI-CC Calculations. *Phys. Chem. Chem. Phys.* **2015**, *17* (2), 1010–1017.

(S32) Cossi, M.; Rega, N.; Scalmani, G.; Barone, V. Energies, Structures, and Electronic Properties of Molecules in Solution with the C-PCM Solvation Model. *J. Comput. Chem.* **2003**, *24* (6), 669–681.

(S33) Barone, V.; Cossi, M. Quantum Calculation of Molecular Energies and Energy Gradients in Solution by a Conductor Solvent Model. *J. Phys. Chem. A* **1998**, *102* (11), 1995–2001.

(S34) Neese, F.; Wennmohs, F.; Becker, U.; Riplinger, C. The ORCA Quantum Chemistry Program Package. *J. Chem. Phys.* **2020**, *152* (22), 224108.

(S35) Chai, J.-D.; Head-Gordon, M. Long-Range Corrected Hybrid Density Functionals with Damped Atom–Atom Dispersion Corrections. *Phys. Chem. Chem. Phys.* **2008**, *10* (44), 6615.

(S36) Grimme, S.; Antony, J.; Ehrlich, S.; Krieg, H. A Consistent and Accurate Ab Initio Parametrization of Density Functional Dispersion Correction (DFT-D) for the 94 Elements H-Pu. *J. Chem. Phys.* **2010**, *132* (15), 154104.

(S37) Zheng, J.; Xu, X.; Truhlar, D. G. Minimally Augmented Karlsruhe Basis Sets. *Theor. Chem. Acc.* **2011**, *128* (3), 295–305.

(S38) Glendening, E. D.; Landis, C. R.; Weinhold, F. NBO 6.0 : Natural Bond Orbital Analysis Program. *J. Comput. Chem.* **2013**, *34* (16), 1429–1437.

(S39) Luchini, G.; Alegre-Requena, J. V.; Funes-Ardoiz, I.; Paton, R. S. GoodVibes: Automated Thermochemistry for Heterogeneous Computational Chemistry Data. *F1000Research* **2020**, *9*, 291.

(S40) Grimme, S. Supramolecular Binding Thermodynamics by Dispersion-Corrected Density Functional Theory. *Chem. - A Eur. J.* **2012**, *18* (32), 9955–9964.

(S41) Pracht, P.; Bohle, F.; Grimme, S. Automated Exploration of the Low-Energy Chemical Space with Fast Quantum Chemical Methods. *Phys. Chem. Chem. Phys.* **2020**, *22* (14), 7169–7192.

(S42) Grimme, S. Exploration of Chemical Compound, Conformer, and Reaction Space with Meta-Dynamics Simulations Based on Tight-Binding Quantum Chemical Calculations. *J. Chem. Theory Comput.* **2019**, *15* (5), 2847–2862.

(S43) Johnson, E. R.; Keinan, S.; Mori-Sánchez, P.; Contreras-García, J.; Cohen, A. J.; Yang, W. Revealing Noncovalent Interactions. *J. Am. Chem. Soc.* **2010**, *132* (18), 6498–6506.

(S44) Contreras-García, J.; Johnson, E. R.; Keinan, S.; Chaudret, R.; Piquemal, J.-P.; Beratan, D. N.; Yang, W. NCIPLOT: A Program for Plotting Noncovalent Interaction Regions. *J. Chem. Theory Comput.* **2011**, *7* (3), 625–632.

(S45) Møller, C.; Plesset, M. S. Note on an Approximation Treatment for Many-Electron Systems. *Phys. Rev.* **1934**, *46* (7), 618–622.

(S46) Liakos, D. G.; Neese, F. Is It Possible To Obtain Coupled Cluster Quality Energies at near Density Functional Theory Cost? Domain-Based Local Pair Natural Orbital Coupled Cluster vs Modern Density Functional Theory. *J. Chem. Theory Comput.* **2015**, *11* (9), 4054–4063.

(S47) Kendall, R. A.; Dunning, T. H.; Harrison, R. J. Electron Affinities of the First-row Atoms Revisited. Systematic Basis Sets and Wave Functions. *J. Chem. Phys.* **1992**, *96* (9), 6796–6806.

(S48) Woon, D. E.; Dunning, T. H. Gaussian Basis Sets for Use in Correlated Molecular Calculations. III. The Atoms Aluminum through Argon. *J. Chem. Phys.* **1993**, *98* (2), 1358–1371.

(S49) Izsák, R.; Hansen, A.; Neese, F. The Resolution of Identity and Chain of Spheres

Approximations for the LPNO-CCSD Singles Fock Term. *Mol. Phys.* **2012**, *110* (19–20), 2413–2417.

(S50) Weigend, F. Accurate Coulomb-Fitting Basis Sets for H to Rn. *Phys. Chem. Chem. Phys.* **2006**, *8* (9), 1057.

(S51) Garcia-Ratés, M.; Neese, F. Effect of the Solute Cavity on the Solvation Energy and Its Derivatives within the Framework of the Gaussian Charge Scheme. *J. Comput. Chem.* **2020**, *41* (9), 922–939.
